# Supplementary material for: High-throughput complement component 4 genomic sequence analysis with C4Investigator
Source: bioRxiv. 2023 Jul 19:2023.07.18.549551. Preprint. [Version 1] doi: 10.1101/2023.07.18.549551 (PMC10370142; doi:10.1101/2023.07.18.549551)
Supplement: Supplement 1 [file media-1.pdf]

Table_S1_suppInfo

1000Genomes project accession resource. 1000Genomes project data is downloaded from: http://s3.amazonaws.com/1000genomes	
**Accession Number**	URL Suffix
**NA06985**	1000G_2504_high_coverage/data/ERR3239276/NA06985.final.cram
**NA06986**	1000G_2504_high_coverage/data/ERR3239277/NA06986.final.cram
**NA06994**	1000G_2504_high_coverage/data/ERR3239278/NA06994.final.cram
**NA07000**	1000G_2504_high_coverage/data/ERR3239279/NA07000.final.cram
**NA07037**	1000G_2504_high_coverage/data/ERR3239280/NA07037.final.cram
**NA07051**	1000G_2504_high_coverage/data/ERR3239281/NA07051.final.cram
**NA07347**	1000G_2504_high_coverage/data/ERR3239282/NA07347.final.cram
**NA07357**	1000G_2504_high_coverage/data/ERR3239283/NA07357.final.cram
**NA10847**	1000G_2504_high_coverage/data/ERR3239284/NA10847.final.cram
**NA10851**	1000G_2504_high_coverage/data/ERR3239285/NA10851.final.cram
**NA11829**	1000G_2504_high_coverage/data/ERR3239286/NA11829.final.cram
**NA11830**	1000G_2504_high_coverage/data/ERR3239287/NA11830.final.cram
**NA11831**	1000G_2504_high_coverage/data/ERR3239288/NA11831.final.cram
**NA11832**	1000G_2504_high_coverage/data/ERR3239289/NA11832.final.cram
**NA11840**	1000G_2504_high_coverage/data/ERR3239290/NA11840.final.cram
**NA11881**	1000G_2504_high_coverage/data/ERR3239291/NA11881.final.cram
**NA11894**	1000G_2504_high_coverage/data/ERR3239292/NA11894.final.cram
**NA11918**	1000G_2504_high_coverage/data/ERR3239293/NA11918.final.cram
**NA11919**	1000G_2504_high_coverage/data/ERR3239294/NA11919.final.cram
**NA11920**	1000G_2504_high_coverage/data/ERR3239295/NA11920.final.cram
**NA11931**	1000G_2504_high_coverage/data/ERR3239296/NA11931.final.cram
**NA11992**	1000G_2504_high_coverage/data/ERR3239297/NA11992.final.cram
**NA11994**	1000G_2504_high_coverage/data/ERR3239298/NA11994.final.cram
**NA11995**	1000G_2504_high_coverage/data/ERR3239299/NA11995.final.cram
**NA12003**	1000G_2504_high_coverage/data/ERR3239300/NA12003.final.cram
**NA12004**	1000G_2504_high_coverage/data/ERR3239301/NA12004.final.cram
**NA12005**	1000G_2504_high_coverage/data/ERR3239302/NA12005.final.cram
**NA12006**	1000G_2504_high_coverage/data/ERR3239303/NA12006.final.cram
**NA12043**	1000G_2504_high_coverage/data/ERR3239304/NA12043.final.cram
**NA12044**	1000G_2504_high_coverage/data/ERR3239305/NA12044.final.cram
**NA12045**	1000G_2504_high_coverage/data/ERR3239306/NA12045.final.cram
**NA12144**	1000G_2504_high_coverage/data/ERR3239307/NA12144.final.cram
**NA12154**	1000G_2504_high_coverage/data/ERR3239308/NA12154.final.cram
**NA12155**	1000G_2504_high_coverage/data/ERR3239309/NA12155.final.cram
**NA12156**	1000G_2504_high_coverage/data/ERR3239310/NA12156.final.cram
**NA12234**	1000G_2504_high_coverage/data/ERR3239311/NA12234.final.cram
**NA12249**	1000G_2504_high_coverage/data/ERR3239312/NA12249.final.cram
**NA12287**	1000G_2504_high_coverage/data/ERR3239313/NA12287.final.cram
**NA12414**	1000G_2504_high_coverage/data/ERR3239314/NA12414.final.cram
**NA12489**	1000G_2504_high_coverage/data/ERR3239315/NA12489.final.cram
**NA12716**	1000G_2504_high_coverage/data/ERR3239316/NA12716.final.cram
**NA12717**	1000G_2504_high_coverage/data/ERR3239317/NA12717.final.cram
**NA12749**	1000G_2504_high_coverage/data/ERR3239318/NA12749.final.cram
**NA12750**	1000G_2504_high_coverage/data/ERR3239319/NA12750.final.cram
**NA12751**	1000G_2504_high_coverage/data/ERR3239320/NA12751.final.cram
**NA12760**	1000G_2504_high_coverage/data/ERR3239321/NA12760.final.cram
**NA12761**	1000G_2504_high_coverage/data/ERR3239322/NA12761.final.cram
**NA12762**	1000G_2504_high_coverage/data/ERR3239323/NA12762.final.cram
**NA12763**	1000G_2504_high_coverage/data/ERR3239324/NA12763.final.cram
**NA12776**	1000G_2504_high_coverage/data/ERR3239325/NA12776.final.cram
**NA12812**	1000G_2504_high_coverage/data/ERR3239326/NA12812.final.cram
**NA12813**	1000G_2504_high_coverage/data/ERR3239327/NA12813.final.cram
**NA12814**	1000G_2504_high_coverage/data/ERR3239328/NA12814.final.cram
**NA12815**	1000G_2504_high_coverage/data/ERR3239329/NA12815.final.cram
**NA12828**	1000G_2504_high_coverage/data/ERR3239330/NA12828.final.cram
**NA12872**	1000G_2504_high_coverage/data/ERR3239331/NA12872.final.cram
**NA12873**	1000G_2504_high_coverage/data/ERR3239332/NA12873.final.cram
**NA12874**	1000G_2504_high_coverage/data/ERR3239333/NA12874.final.cram
**NA12878**	1000G_2504_high_coverage/data/ERR3239334/NA12878.final.cram
**NA18486**	1000G_2504_high_coverage/data/ERR3239335/NA18486.final.cram
**NA18489**	1000G_2504_high_coverage/data/ERR3239336/NA18489.final.cram
**NA18498**	1000G_2504_high_coverage/data/ERR3239337/NA18498.final.cram

**NA18499**	1000G_2504_high_coverage/data/ERR3239338/NA18499.final.cram
**NA18501**	1000G_2504_high_coverage/data/ERR3239339/NA18501.final.cram
**NA18502**	1000G_2504_high_coverage/data/ERR3239340/NA18502.final.cram
**NA18504**	1000G_2504_high_coverage/data/ERR3239341/NA18504.final.cram
**NA18505**	1000G_2504_high_coverage/data/ERR3239342/NA18505.final.cram
**NA18507**	1000G_2504_high_coverage/data/ERR3239343/NA18507.final.cram
**NA18508**	1000G_2504_high_coverage/data/ERR3239344/NA18508.final.cram
**NA18510**	1000G_2504_high_coverage/data/ERR3239345/NA18510.final.cram
**NA18511**	1000G_2504_high_coverage/data/ERR3239346/NA18511.final.cram
**NA18516**	1000G_2504_high_coverage/data/ERR3239347/NA18516.final.cram
**NA18517**	1000G_2504_high_coverage/data/ERR3239348/NA18517.final.cram
**NA18519**	1000G_2504_high_coverage/data/ERR3239349/NA18519.final.cram
**NA18520**	1000G_2504_high_coverage/data/ERR3239350/NA18520.final.cram
**NA18522**	1000G_2504_high_coverage/data/ERR3239351/NA18522.final.cram
**NA18523**	1000G_2504_high_coverage/data/ERR3239352/NA18523.final.cram
**NA18526**	1000G_2504_high_coverage/data/ERR3239353/NA18526.final.cram
**NA18532**	1000G_2504_high_coverage/data/ERR3239354/NA18532.final.cram
**NA18537**	1000G_2504_high_coverage/data/ERR3239355/NA18537.final.cram
**NA18542**	1000G_2504_high_coverage/data/ERR3239356/NA18542.final.cram
**NA18545**	1000G_2504_high_coverage/data/ERR3239357/NA18545.final.cram
**NA18547**	1000G_2504_high_coverage/data/ERR3239358/NA18547.final.cram
**NA18550**	1000G_2504_high_coverage/data/ERR3239359/NA18550.final.cram
**NA18552**	1000G_2504_high_coverage/data/ERR3239360/NA18552.final.cram
**NA18555**	1000G_2504_high_coverage/data/ERR3239361/NA18555.final.cram
**NA18558**	1000G_2504_high_coverage/data/ERR3239362/NA18558.final.cram
**NA18561**	1000G_2504_high_coverage/data/ERR3239363/NA18561.final.cram
**NA18562**	1000G_2504_high_coverage/data/ERR3239364/NA18562.final.cram
**NA18563**	1000G_2504_high_coverage/data/ERR3239365/NA18563.final.cram
**NA18564**	1000G_2504_high_coverage/data/ERR3239366/NA18564.final.cram
**NA18566**	1000G_2504_high_coverage/data/ERR3239367/NA18566.final.cram
**NA18570**	1000G_2504_high_coverage/data/ERR3239368/NA18570.final.cram
**NA18571**	1000G_2504_high_coverage/data/ERR3239369/NA18571.final.cram
**NA18572**	1000G_2504_high_coverage/data/ERR3239370/NA18572.final.cram
**NA18573**	1000G_2504_high_coverage/data/ERR3239371/NA18573.final.cram
**NA18577**	1000G_2504_high_coverage/data/ERR3239372/NA18577.final.cram
**NA18579**	1000G_2504_high_coverage/data/ERR3239373/NA18579.final.cram
**NA18582**	1000G_2504_high_coverage/data/ERR3239374/NA18582.final.cram
**NA18592**	1000G_2504_high_coverage/data/ERR3239375/NA18592.final.cram
**NA18593**	1000G_2504_high_coverage/data/ERR3239376/NA18593.final.cram
**NA18603**	1000G_2504_high_coverage/data/ERR3239377/NA18603.final.cram
**NA18605**	1000G_2504_high_coverage/data/ERR3239378/NA18605.final.cram
**NA18608**	1000G_2504_high_coverage/data/ERR3239379/NA18608.final.cram
**NA18609**	1000G_2504_high_coverage/data/ERR3239380/NA18609.final.cram
**NA18638**	1000G_2504_high_coverage/data/ERR3239381/NA18638.final.cram
**NA18853**	1000G_2504_high_coverage/data/ERR3239382/NA18853.final.cram
**NA18856**	1000G_2504_high_coverage/data/ERR3239383/NA18856.final.cram
**NA18858**	1000G_2504_high_coverage/data/ERR3239384/NA18858.final.cram
**NA18861**	1000G_2504_high_coverage/data/ERR3239385/NA18861.final.cram
**NA18870**	1000G_2504_high_coverage/data/ERR3239386/NA18870.final.cram
**NA18871**	1000G_2504_high_coverage/data/ERR3239387/NA18871.final.cram
**NA18907**	1000G_2504_high_coverage/data/ERR3239388/NA18907.final.cram
**NA18909**	1000G_2504_high_coverage/data/ERR3239389/NA18909.final.cram
**NA18912**	1000G_2504_high_coverage/data/ERR3239390/NA18912.final.cram
**NA18916**	1000G_2504_high_coverage/data/ERR3239391/NA18916.final.cram
**NA18940**	1000G_2504_high_coverage/data/ERR3239392/NA18940.final.cram
**NA18942**	1000G_2504_high_coverage/data/ERR3239393/NA18942.final.cram
**NA18943**	1000G_2504_high_coverage/data/ERR3239394/NA18943.final.cram
**NA18944**	1000G_2504_high_coverage/data/ERR3239395/NA18944.final.cram
**NA18945**	1000G_2504_high_coverage/data/ERR3239396/NA18945.final.cram
**NA18947**	1000G_2504_high_coverage/data/ERR3239397/NA18947.final.cram
**NA18948**	1000G_2504_high_coverage/data/ERR3239398/NA18948.final.cram
**NA18949**	1000G_2504_high_coverage/data/ERR3239399/NA18949.final.cram
**NA18951**	1000G_2504_high_coverage/data/ERR3239400/NA18951.final.cram
**NA18952**	1000G_2504_high_coverage/data/ERR3239401/NA18952.final.cram
**NA18953**	1000G_2504_high_coverage/data/ERR3239402/NA18953.final.cram

**NA18956**	1000G_2504_high_coverage/data/ERR3239403/NA18956.final.cram
**NA18959**	1000G_2504_high_coverage/data/ERR3239404/NA18959.final.cram
**NA18960**	1000G_2504_high_coverage/data/ERR3239405/NA18960.final.cram
**NA18961**	1000G_2504_high_coverage/data/ERR3239406/NA18961.final.cram
**NA18964**	1000G_2504_high_coverage/data/ERR3239407/NA18964.final.cram
**NA18965**	1000G_2504_high_coverage/data/ERR3239408/NA18965.final.cram
**NA18967**	1000G_2504_high_coverage/data/ERR3239409/NA18967.final.cram
**NA18968**	1000G_2504_high_coverage/data/ERR3239410/NA18968.final.cram
**NA18969**	1000G_2504_high_coverage/data/ERR3239411/NA18969.final.cram
**NA18970**	1000G_2504_high_coverage/data/ERR3239412/NA18970.final.cram
**NA18971**	1000G_2504_high_coverage/data/ERR3239413/NA18971.final.cram
**NA18972**	1000G_2504_high_coverage/data/ERR3239414/NA18972.final.cram
**NA18973**	1000G_2504_high_coverage/data/ERR3239415/NA18973.final.cram
**NA18974**	1000G_2504_high_coverage/data/ERR3239416/NA18974.final.cram
**NA18975**	1000G_2504_high_coverage/data/ERR3239417/NA18975.final.cram
**NA18976**	1000G_2504_high_coverage/data/ERR3239418/NA18976.final.cram
**NA18980**	1000G_2504_high_coverage/data/ERR3239419/NA18980.final.cram
**NA18981**	1000G_2504_high_coverage/data/ERR3239420/NA18981.final.cram
**NA19005**	1000G_2504_high_coverage/data/ERR3239421/NA19005.final.cram
**NA19093**	1000G_2504_high_coverage/data/ERR3239422/NA19093.final.cram
**NA19098**	1000G_2504_high_coverage/data/ERR3239423/NA19098.final.cram
**NA19099**	1000G_2504_high_coverage/data/ERR3239424/NA19099.final.cram
**NA19102**	1000G_2504_high_coverage/data/ERR3239425/NA19102.final.cram
**NA19108**	1000G_2504_high_coverage/data/ERR3239426/NA19108.final.cram
**NA19114**	1000G_2504_high_coverage/data/ERR3239427/NA19114.final.cram
**NA19116**	1000G_2504_high_coverage/data/ERR3239428/NA19116.final.cram
**NA19119**	1000G_2504_high_coverage/data/ERR3239429/NA19119.final.cram
**NA19129**	1000G_2504_high_coverage/data/ERR3239430/NA19129.final.cram
**NA19131**	1000G_2504_high_coverage/data/ERR3239431/NA19131.final.cram
**NA19137**	1000G_2504_high_coverage/data/ERR3239432/NA19137.final.cram
**NA19138**	1000G_2504_high_coverage/data/ERR3239433/NA19138.final.cram
**NA19141**	1000G_2504_high_coverage/data/ERR3239434/NA19141.final.cram
**NA19143**	1000G_2504_high_coverage/data/ERR3239435/NA19143.final.cram
**NA19144**	1000G_2504_high_coverage/data/ERR3239436/NA19144.final.cram
**NA19147**	1000G_2504_high_coverage/data/ERR3239437/NA19147.final.cram
**NA19152**	1000G_2504_high_coverage/data/ERR3239438/NA19152.final.cram
**NA19153**	1000G_2504_high_coverage/data/ERR3239439/NA19153.final.cram
**NA19159**	1000G_2504_high_coverage/data/ERR3239440/NA19159.final.cram
**NA19160**	1000G_2504_high_coverage/data/ERR3239441/NA19160.final.cram
**NA19171**	1000G_2504_high_coverage/data/ERR3239442/NA19171.final.cram
**NA19172**	1000G_2504_high_coverage/data/ERR3239443/NA19172.final.cram
**NA19190**	1000G_2504_high_coverage/data/ERR3239444/NA19190.final.cram
**NA19200**	1000G_2504_high_coverage/data/ERR3239445/NA19200.final.cram
**NA19201**	1000G_2504_high_coverage/data/ERR3239446/NA19201.final.cram
**NA19204**	1000G_2504_high_coverage/data/ERR3239447/NA19204.final.cram
**NA19206**	1000G_2504_high_coverage/data/ERR3239448/NA19206.final.cram
**NA19207**	1000G_2504_high_coverage/data/ERR3239449/NA19207.final.cram
**NA19209**	1000G_2504_high_coverage/data/ERR3239450/NA19209.final.cram
**NA19210**	1000G_2504_high_coverage/data/ERR3239451/NA19210.final.cram
**NA19225**	1000G_2504_high_coverage/data/ERR3239452/NA19225.final.cram
**NA19238**	1000G_2504_high_coverage/data/ERR3239453/NA19238.final.cram
**NA19239**	1000G_2504_high_coverage/data/ERR3239454/NA19239.final.cram
**NA19257**	1000G_2504_high_coverage/data/ERR3239455/NA19257.final.cram
**NA07056**	1000G_2504_high_coverage/data/ERR3239456/NA07056.final.cram
**NA18614**	1000G_2504_high_coverage/data/ERR3239457/NA18614.final.cram
**NA06984**	1000G_2504_high_coverage/data/ERR3239458/NA06984.final.cram
**NA06989**	1000G_2504_high_coverage/data/ERR3239459/NA06989.final.cram
**NA11843**	1000G_2504_high_coverage/data/ERR3239460/NA11843.final.cram
**NA11892**	1000G_2504_high_coverage/data/ERR3239461/NA11892.final.cram
**NA11893**	1000G_2504_high_coverage/data/ERR3239462/NA11893.final.cram
**NA11930**	1000G_2504_high_coverage/data/ERR3239463/NA11930.final.cram
**NA12058**	1000G_2504_high_coverage/data/ERR3239464/NA12058.final.cram
**NA12272**	1000G_2504_high_coverage/data/ERR3239465/NA12272.final.cram
**NA12273**	1000G_2504_high_coverage/data/ERR3239466/NA12273.final.cram
**NA12275**	1000G_2504_high_coverage/data/ERR3239467/NA12275.final.cram

**NA12282**	1000G_2504_high_coverage/data/ERR3239468/NA12282.final.cram
**NA12283**	1000G_2504_high_coverage/data/ERR3239469/NA12283.final.cram
**NA12286**	1000G_2504_high_coverage/data/ERR3239470/NA12286.final.cram
**NA12340**	1000G_2504_high_coverage/data/ERR3239471/NA12340.final.cram
**NA12341**	1000G_2504_high_coverage/data/ERR3239472/NA12341.final.cram
**NA12342**	1000G_2504_high_coverage/data/ERR3239473/NA12342.final.cram
**NA12347**	1000G_2504_high_coverage/data/ERR3239474/NA12347.final.cram
**NA12348**	1000G_2504_high_coverage/data/ERR3239475/NA12348.final.cram
**NA12383**	1000G_2504_high_coverage/data/ERR3239476/NA12383.final.cram
**NA12399**	1000G_2504_high_coverage/data/ERR3239477/NA12399.final.cram
**NA12400**	1000G_2504_high_coverage/data/ERR3239478/NA12400.final.cram
**NA12413**	1000G_2504_high_coverage/data/ERR3239479/NA12413.final.cram
**NA12718**	1000G_2504_high_coverage/data/ERR3239480/NA12718.final.cram
**NA12748**	1000G_2504_high_coverage/data/ERR3239481/NA12748.final.cram
**NA12775**	1000G_2504_high_coverage/data/ERR3239482/NA12775.final.cram
**NA12777**	1000G_2504_high_coverage/data/ERR3239483/NA12777.final.cram
**NA12778**	1000G_2504_high_coverage/data/ERR3239484/NA12778.final.cram
**NA12827**	1000G_2504_high_coverage/data/ERR3239485/NA12827.final.cram
**NA12829**	1000G_2504_high_coverage/data/ERR3239486/NA12829.final.cram
**NA12842**	1000G_2504_high_coverage/data/ERR3239487/NA12842.final.cram
**NA12843**	1000G_2504_high_coverage/data/ERR3239488/NA12843.final.cram
**NA12889**	1000G_2504_high_coverage/data/ERR3239489/NA12889.final.cram
**NA12890**	1000G_2504_high_coverage/data/ERR3239490/NA12890.final.cram
**NA18488**	1000G_2504_high_coverage/data/ERR3239491/NA18488.final.cram
**NA18530**	1000G_2504_high_coverage/data/ERR3239492/NA18530.final.cram
**NA18534**	1000G_2504_high_coverage/data/ERR3239493/NA18534.final.cram
**NA18536**	1000G_2504_high_coverage/data/ERR3239494/NA18536.final.cram
**NA18543**	1000G_2504_high_coverage/data/ERR3239495/NA18543.final.cram
**NA18544**	1000G_2504_high_coverage/data/ERR3239496/NA18544.final.cram
**NA18546**	1000G_2504_high_coverage/data/ERR3239497/NA18546.final.cram
**NA18548**	1000G_2504_high_coverage/data/ERR3239498/NA18548.final.cram
**NA18549**	1000G_2504_high_coverage/data/ERR3239499/NA18549.final.cram
**NA18557**	1000G_2504_high_coverage/data/ERR3239500/NA18557.final.cram
**NA18559**	1000G_2504_high_coverage/data/ERR3239501/NA18559.final.cram
**NA18595**	1000G_2504_high_coverage/data/ERR3239502/NA18595.final.cram
**NA18596**	1000G_2504_high_coverage/data/ERR3239503/NA18596.final.cram
**NA18597**	1000G_2504_high_coverage/data/ERR3239504/NA18597.final.cram
**NA18599**	1000G_2504_high_coverage/data/ERR3239505/NA18599.final.cram
**NA18602**	1000G_2504_high_coverage/data/ERR3239506/NA18602.final.cram
**NA18606**	1000G_2504_high_coverage/data/ERR3239507/NA18606.final.cram
**NA18610**	1000G_2504_high_coverage/data/ERR3239508/NA18610.final.cram
**NA18611**	1000G_2504_high_coverage/data/ERR3239509/NA18611.final.cram
**NA18612**	1000G_2504_high_coverage/data/ERR3239510/NA18612.final.cram
**NA18613**	1000G_2504_high_coverage/data/ERR3239511/NA18613.final.cram
**NA18615**	1000G_2504_high_coverage/data/ERR3239512/NA18615.final.cram
**NA18616**	1000G_2504_high_coverage/data/ERR3239513/NA18616.final.cram
**NA18617**	1000G_2504_high_coverage/data/ERR3239514/NA18617.final.cram
**NA18618**	1000G_2504_high_coverage/data/ERR3239515/NA18618.final.cram
**NA18619**	1000G_2504_high_coverage/data/ERR3239516/NA18619.final.cram
**NA18620**	1000G_2504_high_coverage/data/ERR3239517/NA18620.final.cram
**NA18621**	1000G_2504_high_coverage/data/ERR3239518/NA18621.final.cram
**NA18622**	1000G_2504_high_coverage/data/ERR3239519/NA18622.final.cram
**NA18623**	1000G_2504_high_coverage/data/ERR3239520/NA18623.final.cram
**NA18624**	1000G_2504_high_coverage/data/ERR3239521/NA18624.final.cram
**NA18625**	1000G_2504_high_coverage/data/ERR3239522/NA18625.final.cram
**NA18626**	1000G_2504_high_coverage/data/ERR3239523/NA18626.final.cram
**NA18627**	1000G_2504_high_coverage/data/ERR3239524/NA18627.final.cram
**NA18628**	1000G_2504_high_coverage/data/ERR3239525/NA18628.final.cram
**NA18630**	1000G_2504_high_coverage/data/ERR3239526/NA18630.final.cram
**NA18631**	1000G_2504_high_coverage/data/ERR3239527/NA18631.final.cram
**NA18632**	1000G_2504_high_coverage/data/ERR3239528/NA18632.final.cram
**NA18633**	1000G_2504_high_coverage/data/ERR3239529/NA18633.final.cram
**NA18634**	1000G_2504_high_coverage/data/ERR3239530/NA18634.final.cram
**NA18635**	1000G_2504_high_coverage/data/ERR3239531/NA18635.final.cram
**NA18636**	1000G_2504_high_coverage/data/ERR3239532/NA18636.final.cram

**NA18637**	1000G_2504_high_coverage/data/ERR3239533/NA18637.final.cram
**NA18639**	1000G_2504_high_coverage/data/ERR3239534/NA18639.final.cram
**NA18640**	1000G_2504_high_coverage/data/ERR3239535/NA18640.final.cram
**NA18641**	1000G_2504_high_coverage/data/ERR3239536/NA18641.final.cram
**NA18642**	1000G_2504_high_coverage/data/ERR3239537/NA18642.final.cram
**NA18643**	1000G_2504_high_coverage/data/ERR3239538/NA18643.final.cram
**NA18645**	1000G_2504_high_coverage/data/ERR3239539/NA18645.final.cram
**NA18647**	1000G_2504_high_coverage/data/ERR3239540/NA18647.final.cram
**NA18740**	1000G_2504_high_coverage/data/ERR3239541/NA18740.final.cram
**NA18745**	1000G_2504_high_coverage/data/ERR3239542/NA18745.final.cram
**NA18747**	1000G_2504_high_coverage/data/ERR3239543/NA18747.final.cram
**NA18748**	1000G_2504_high_coverage/data/ERR3239544/NA18748.final.cram
**NA18749**	1000G_2504_high_coverage/data/ERR3239545/NA18749.final.cram
**NA18757**	1000G_2504_high_coverage/data/ERR3239546/NA18757.final.cram
**NA18867**	1000G_2504_high_coverage/data/ERR3239547/NA18867.final.cram
**NA18868**	1000G_2504_high_coverage/data/ERR3239548/NA18868.final.cram
**NA18873**	1000G_2504_high_coverage/data/ERR3239549/NA18873.final.cram
**NA18908**	1000G_2504_high_coverage/data/ERR3239550/NA18908.final.cram
**NA18910**	1000G_2504_high_coverage/data/ERR3239551/NA18910.final.cram
**NA18917**	1000G_2504_high_coverage/data/ERR3239552/NA18917.final.cram
**NA18923**	1000G_2504_high_coverage/data/ERR3239553/NA18923.final.cram
**NA18924**	1000G_2504_high_coverage/data/ERR3239554/NA18924.final.cram
**NA18933**	1000G_2504_high_coverage/data/ERR3239555/NA18933.final.cram
**NA18934**	1000G_2504_high_coverage/data/ERR3239556/NA18934.final.cram
**NA18939**	1000G_2504_high_coverage/data/ERR3239557/NA18939.final.cram
**NA18941**	1000G_2504_high_coverage/data/ERR3239558/NA18941.final.cram
**NA18946**	1000G_2504_high_coverage/data/ERR3239559/NA18946.final.cram
**NA18954**	1000G_2504_high_coverage/data/ERR3239560/NA18954.final.cram
**NA18957**	1000G_2504_high_coverage/data/ERR3239561/NA18957.final.cram
**NA18962**	1000G_2504_high_coverage/data/ERR3239562/NA18962.final.cram
**NA18963**	1000G_2504_high_coverage/data/ERR3239563/NA18963.final.cram
**NA18966**	1000G_2504_high_coverage/data/ERR3239564/NA18966.final.cram
**NA18977**	1000G_2504_high_coverage/data/ERR3239565/NA18977.final.cram
**NA18978**	1000G_2504_high_coverage/data/ERR3239566/NA18978.final.cram
**NA18979**	1000G_2504_high_coverage/data/ERR3239567/NA18979.final.cram
**NA18987**	1000G_2504_high_coverage/data/ERR3239568/NA18987.final.cram
**NA18990**	1000G_2504_high_coverage/data/ERR3239569/NA18990.final.cram
**NA18991**	1000G_2504_high_coverage/data/ERR3239570/NA18991.final.cram
**NA18992**	1000G_2504_high_coverage/data/ERR3239571/NA18992.final.cram
**NA18993**	1000G_2504_high_coverage/data/ERR3239572/NA18993.final.cram
**NA18994**	1000G_2504_high_coverage/data/ERR3239573/NA18994.final.cram
**NA18995**	1000G_2504_high_coverage/data/ERR3239574/NA18995.final.cram
**NA18997**	1000G_2504_high_coverage/data/ERR3239575/NA18997.final.cram
**NA18998**	1000G_2504_high_coverage/data/ERR3239576/NA18998.final.cram
**NA18999**	1000G_2504_high_coverage/data/ERR3239577/NA18999.final.cram
**NA19000**	1000G_2504_high_coverage/data/ERR3239578/NA19000.final.cram
**NA19001**	1000G_2504_high_coverage/data/ERR3239579/NA19001.final.cram
**NA19002**	1000G_2504_high_coverage/data/ERR3239580/NA19002.final.cram
**NA19003**	1000G_2504_high_coverage/data/ERR3239581/NA19003.final.cram
**NA19007**	1000G_2504_high_coverage/data/ERR3239582/NA19007.final.cram
**NA19009**	1000G_2504_high_coverage/data/ERR3239583/NA19009.final.cram
**NA19010**	1000G_2504_high_coverage/data/ERR3239584/NA19010.final.cram
**NA19012**	1000G_2504_high_coverage/data/ERR3239585/NA19012.final.cram
**NA19054**	1000G_2504_high_coverage/data/ERR3239586/NA19054.final.cram
**NA19055**	1000G_2504_high_coverage/data/ERR3239587/NA19055.final.cram
**NA19056**	1000G_2504_high_coverage/data/ERR3239588/NA19056.final.cram
**NA19057**	1000G_2504_high_coverage/data/ERR3239589/NA19057.final.cram
**NA19058**	1000G_2504_high_coverage/data/ERR3239590/NA19058.final.cram
**NA19059**	1000G_2504_high_coverage/data/ERR3239591/NA19059.final.cram
**NA19060**	1000G_2504_high_coverage/data/ERR3239592/NA19060.final.cram
**NA19062**	1000G_2504_high_coverage/data/ERR3239593/NA19062.final.cram
**NA19063**	1000G_2504_high_coverage/data/ERR3239594/NA19063.final.cram
**NA19064**	1000G_2504_high_coverage/data/ERR3239595/NA19064.final.cram
**NA19065**	1000G_2504_high_coverage/data/ERR3239596/NA19065.final.cram
**NA19066**	1000G_2504_high_coverage/data/ERR3239597/NA19066.final.cram

**NA19067**	1000G_2504_high_coverage/data/ERR3239598/NA19067.final.cram
**NA19068**	1000G_2504_high_coverage/data/ERR3239599/NA19068.final.cram
**NA19070**	1000G_2504_high_coverage/data/ERR3239600/NA19070.final.cram
**NA19072**	1000G_2504_high_coverage/data/ERR3239601/NA19072.final.cram
**NA19074**	1000G_2504_high_coverage/data/ERR3239602/NA19074.final.cram
**NA19075**	1000G_2504_high_coverage/data/ERR3239603/NA19075.final.cram
**NA19076**	1000G_2504_high_coverage/data/ERR3239604/NA19076.final.cram
**NA19077**	1000G_2504_high_coverage/data/ERR3239605/NA19077.final.cram
**NA19078**	1000G_2504_high_coverage/data/ERR3239606/NA19078.final.cram
**NA19079**	1000G_2504_high_coverage/data/ERR3239607/NA19079.final.cram
**NA19080**	1000G_2504_high_coverage/data/ERR3239608/NA19080.final.cram
**NA19081**	1000G_2504_high_coverage/data/ERR3239609/NA19081.final.cram
**NA19082**	1000G_2504_high_coverage/data/ERR3239610/NA19082.final.cram
**NA19083**	1000G_2504_high_coverage/data/ERR3239611/NA19083.final.cram
**NA19084**	1000G_2504_high_coverage/data/ERR3239612/NA19084.final.cram
**NA19085**	1000G_2504_high_coverage/data/ERR3239613/NA19085.final.cram
**NA19086**	1000G_2504_high_coverage/data/ERR3239614/NA19086.final.cram
**NA19087**	1000G_2504_high_coverage/data/ERR3239615/NA19087.final.cram
**NA19088**	1000G_2504_high_coverage/data/ERR3239616/NA19088.final.cram
**NA19092**	1000G_2504_high_coverage/data/ERR3239617/NA19092.final.cram
**NA19095**	1000G_2504_high_coverage/data/ERR3239618/NA19095.final.cram
**NA19096**	1000G_2504_high_coverage/data/ERR3239619/NA19096.final.cram
**NA19107**	1000G_2504_high_coverage/data/ERR3239620/NA19107.final.cram
**NA19113**	1000G_2504_high_coverage/data/ERR3239621/NA19113.final.cram
**NA19117**	1000G_2504_high_coverage/data/ERR3239622/NA19117.final.cram
**NA19118**	1000G_2504_high_coverage/data/ERR3239623/NA19118.final.cram
**NA19121**	1000G_2504_high_coverage/data/ERR3239624/NA19121.final.cram
**NA19130**	1000G_2504_high_coverage/data/ERR3239625/NA19130.final.cram
**NA19146**	1000G_2504_high_coverage/data/ERR3239626/NA19146.final.cram
**NA19149**	1000G_2504_high_coverage/data/ERR3239627/NA19149.final.cram
**NA19175**	1000G_2504_high_coverage/data/ERR3239628/NA19175.final.cram
**NA19184**	1000G_2504_high_coverage/data/ERR3239629/NA19184.final.cram
**NA19185**	1000G_2504_high_coverage/data/ERR3239630/NA19185.final.cram
**NA19189**	1000G_2504_high_coverage/data/ERR3239631/NA19189.final.cram
**NA19197**	1000G_2504_high_coverage/data/ERR3239632/NA19197.final.cram
**NA19198**	1000G_2504_high_coverage/data/ERR3239633/NA19198.final.cram
**NA19213**	1000G_2504_high_coverage/data/ERR3239634/NA19213.final.cram
**NA19214**	1000G_2504_high_coverage/data/ERR3239635/NA19214.final.cram
**NA19223**	1000G_2504_high_coverage/data/ERR3239636/NA19223.final.cram
**NA19235**	1000G_2504_high_coverage/data/ERR3239637/NA19235.final.cram
**NA19236**	1000G_2504_high_coverage/data/ERR3239638/NA19236.final.cram
**NA19247**	1000G_2504_high_coverage/data/ERR3239639/NA19247.final.cram
**NA19248**	1000G_2504_high_coverage/data/ERR3239640/NA19248.final.cram
**NA19256**	1000G_2504_high_coverage/data/ERR3239641/NA19256.final.cram
**NA07048**	1000G_2504_high_coverage/data/ERR3239642/NA07048.final.cram
**NA11932**	1000G_2504_high_coverage/data/ERR3239643/NA11932.final.cram
**NA11933**	1000G_2504_high_coverage/data/ERR3239644/NA11933.final.cram
**NA12046**	1000G_2504_high_coverage/data/ERR3239645/NA12046.final.cram
**NA18525**	1000G_2504_high_coverage/data/ERR3239646/NA18525.final.cram
**NA18528**	1000G_2504_high_coverage/data/ERR3239647/NA18528.final.cram
**NA18531**	1000G_2504_high_coverage/data/ERR3239648/NA18531.final.cram
**NA18533**	1000G_2504_high_coverage/data/ERR3239649/NA18533.final.cram
**NA18535**	1000G_2504_high_coverage/data/ERR3239650/NA18535.final.cram
**NA18538**	1000G_2504_high_coverage/data/ERR3239651/NA18538.final.cram
**NA18539**	1000G_2504_high_coverage/data/ERR3239652/NA18539.final.cram
**NA18541**	1000G_2504_high_coverage/data/ERR3239653/NA18541.final.cram
**NA18553**	1000G_2504_high_coverage/data/ERR3239654/NA18553.final.cram
**NA18560**	1000G_2504_high_coverage/data/ERR3239655/NA18560.final.cram
**NA18565**	1000G_2504_high_coverage/data/ERR3239656/NA18565.final.cram
**NA18567**	1000G_2504_high_coverage/data/ERR3239657/NA18567.final.cram
**NA18574**	1000G_2504_high_coverage/data/ERR3239658/NA18574.final.cram
**NA18591**	1000G_2504_high_coverage/data/ERR3239659/NA18591.final.cram
**NA18629**	1000G_2504_high_coverage/data/ERR3239660/NA18629.final.cram
**NA18644**	1000G_2504_high_coverage/data/ERR3239661/NA18644.final.cram
**NA18646**	1000G_2504_high_coverage/data/ERR3239662/NA18646.final.cram

**NA18648**	1000G_2504_high_coverage/data/ERR3239663/NA18648.final.cram
**NA18864**	1000G_2504_high_coverage/data/ERR3239664/NA18864.final.cram
**NA18865**	1000G_2504_high_coverage/data/ERR3239665/NA18865.final.cram
**NA18876**	1000G_2504_high_coverage/data/ERR3239666/NA18876.final.cram
**NA18877**	1000G_2504_high_coverage/data/ERR3239667/NA18877.final.cram
**NA18878**	1000G_2504_high_coverage/data/ERR3239668/NA18878.final.cram
**NA18879**	1000G_2504_high_coverage/data/ERR3239669/NA18879.final.cram
**NA18881**	1000G_2504_high_coverage/data/ERR3239670/NA18881.final.cram
**NA18915**	1000G_2504_high_coverage/data/ERR3239671/NA18915.final.cram
**NA18950**	1000G_2504_high_coverage/data/ERR3239672/NA18950.final.cram
**NA18982**	1000G_2504_high_coverage/data/ERR3239673/NA18982.final.cram
**NA18983**	1000G_2504_high_coverage/data/ERR3239674/NA18983.final.cram
**NA18984**	1000G_2504_high_coverage/data/ERR3239675/NA18984.final.cram
**NA18985**	1000G_2504_high_coverage/data/ERR3239676/NA18985.final.cram
**NA18986**	1000G_2504_high_coverage/data/ERR3239677/NA18986.final.cram
**NA18988**	1000G_2504_high_coverage/data/ERR3239678/NA18988.final.cram
**NA18989**	1000G_2504_high_coverage/data/ERR3239679/NA18989.final.cram
**NA19004**	1000G_2504_high_coverage/data/ERR3239680/NA19004.final.cram
**NA19006**	1000G_2504_high_coverage/data/ERR3239681/NA19006.final.cram
**NA19011**	1000G_2504_high_coverage/data/ERR3239682/NA19011.final.cram
**NA19017**	1000G_2504_high_coverage/data/ERR3239683/NA19017.final.cram
**NA19019**	1000G_2504_high_coverage/data/ERR3239684/NA19019.final.cram
**NA19020**	1000G_2504_high_coverage/data/ERR3239685/NA19020.final.cram
**NA19023**	1000G_2504_high_coverage/data/ERR3239686/NA19023.final.cram
**NA19024**	1000G_2504_high_coverage/data/ERR3239687/NA19024.final.cram
**NA19025**	1000G_2504_high_coverage/data/ERR3239688/NA19025.final.cram
**NA19026**	1000G_2504_high_coverage/data/ERR3239689/NA19026.final.cram
**NA19027**	1000G_2504_high_coverage/data/ERR3239690/NA19027.final.cram
**NA19028**	1000G_2504_high_coverage/data/ERR3239691/NA19028.final.cram
**NA19030**	1000G_2504_high_coverage/data/ERR3239692/NA19030.final.cram
**NA19031**	1000G_2504_high_coverage/data/ERR3239693/NA19031.final.cram
**NA19035**	1000G_2504_high_coverage/data/ERR3239694/NA19035.final.cram
**NA19036**	1000G_2504_high_coverage/data/ERR3239695/NA19036.final.cram
**NA19037**	1000G_2504_high_coverage/data/ERR3239696/NA19037.final.cram
**NA19038**	1000G_2504_high_coverage/data/ERR3239697/NA19038.final.cram
**NA19041**	1000G_2504_high_coverage/data/ERR3239698/NA19041.final.cram
**NA19042**	1000G_2504_high_coverage/data/ERR3239699/NA19042.final.cram
**NA19043**	1000G_2504_high_coverage/data/ERR3239700/NA19043.final.cram
**NA19089**	1000G_2504_high_coverage/data/ERR3239701/NA19089.final.cram
**NA19090**	1000G_2504_high_coverage/data/ERR3239702/NA19090.final.cram
**NA19091**	1000G_2504_high_coverage/data/ERR3239703/NA19091.final.cram
**NA19307**	1000G_2504_high_coverage/data/ERR3239704/NA19307.final.cram
**NA19308**	1000G_2504_high_coverage/data/ERR3239705/NA19308.final.cram
**NA19309**	1000G_2504_high_coverage/data/ERR3239706/NA19309.final.cram
**NA19310**	1000G_2504_high_coverage/data/ERR3239707/NA19310.final.cram
**NA19312**	1000G_2504_high_coverage/data/ERR3239708/NA19312.final.cram
**NA19314**	1000G_2504_high_coverage/data/ERR3239709/NA19314.final.cram
**NA19315**	1000G_2504_high_coverage/data/ERR3239710/NA19315.final.cram
**NA19316**	1000G_2504_high_coverage/data/ERR3239711/NA19316.final.cram
**NA19317**	1000G_2504_high_coverage/data/ERR3239712/NA19317.final.cram
**NA19318**	1000G_2504_high_coverage/data/ERR3239713/NA19318.final.cram
**NA19319**	1000G_2504_high_coverage/data/ERR3239714/NA19319.final.cram
**NA19320**	1000G_2504_high_coverage/data/ERR3239715/NA19320.final.cram
**NA19321**	1000G_2504_high_coverage/data/ERR3239716/NA19321.final.cram
**NA19323**	1000G_2504_high_coverage/data/ERR3239717/NA19323.final.cram
**NA19324**	1000G_2504_high_coverage/data/ERR3239718/NA19324.final.cram
**NA19327**	1000G_2504_high_coverage/data/ERR3239719/NA19327.final.cram
**NA19328**	1000G_2504_high_coverage/data/ERR3239720/NA19328.final.cram
**NA19331**	1000G_2504_high_coverage/data/ERR3239721/NA19331.final.cram
**NA19332**	1000G_2504_high_coverage/data/ERR3239722/NA19332.final.cram
**NA19334**	1000G_2504_high_coverage/data/ERR3239723/NA19334.final.cram
**NA19338**	1000G_2504_high_coverage/data/ERR3239724/NA19338.final.cram
**NA19346**	1000G_2504_high_coverage/data/ERR3239725/NA19346.final.cram
**NA19347**	1000G_2504_high_coverage/data/ERR3239726/NA19347.final.cram
**NA19350**	1000G_2504_high_coverage/data/ERR3239727/NA19350.final.cram

**NA19351**	1000G_2504_high_coverage/data/ERR3239728/NA19351.final.cram
**NA19355**	1000G_2504_high_coverage/data/ERR3239729/NA19355.final.cram
**NA19360**	1000G_2504_high_coverage/data/ERR3239730/NA19360.final.cram
**NA19372**	1000G_2504_high_coverage/data/ERR3239731/NA19372.final.cram
**NA19374**	1000G_2504_high_coverage/data/ERR3239732/NA19374.final.cram
**NA19375**	1000G_2504_high_coverage/data/ERR3239733/NA19375.final.cram
**NA19376**	1000G_2504_high_coverage/data/ERR3239734/NA19376.final.cram
**NA19377**	1000G_2504_high_coverage/data/ERR3239735/NA19377.final.cram
**NA19378**	1000G_2504_high_coverage/data/ERR3239736/NA19378.final.cram
**NA19379**	1000G_2504_high_coverage/data/ERR3239737/NA19379.final.cram
**NA19380**	1000G_2504_high_coverage/data/ERR3239738/NA19380.final.cram
**NA19383**	1000G_2504_high_coverage/data/ERR3239739/NA19383.final.cram
**NA19384**	1000G_2504_high_coverage/data/ERR3239740/NA19384.final.cram
**NA19385**	1000G_2504_high_coverage/data/ERR3239741/NA19385.final.cram
**NA19390**	1000G_2504_high_coverage/data/ERR3239742/NA19390.final.cram
**NA19391**	1000G_2504_high_coverage/data/ERR3239743/NA19391.final.cram
**NA19393**	1000G_2504_high_coverage/data/ERR3239744/NA19393.final.cram
**NA19394**	1000G_2504_high_coverage/data/ERR3239745/NA19394.final.cram
**NA19395**	1000G_2504_high_coverage/data/ERR3239746/NA19395.final.cram
**NA19397**	1000G_2504_high_coverage/data/ERR3239747/NA19397.final.cram
**NA19399**	1000G_2504_high_coverage/data/ERR3239748/NA19399.final.cram
**NA19401**	1000G_2504_high_coverage/data/ERR3239749/NA19401.final.cram
**NA19403**	1000G_2504_high_coverage/data/ERR3239750/NA19403.final.cram
**NA19404**	1000G_2504_high_coverage/data/ERR3239751/NA19404.final.cram
**NA19428**	1000G_2504_high_coverage/data/ERR3239752/NA19428.final.cram
**NA19429**	1000G_2504_high_coverage/data/ERR3239753/NA19429.final.cram
**NA19430**	1000G_2504_high_coverage/data/ERR3239754/NA19430.final.cram
**NA19431**	1000G_2504_high_coverage/data/ERR3239755/NA19431.final.cram
**NA19434**	1000G_2504_high_coverage/data/ERR3239756/NA19434.final.cram
**NA19435**	1000G_2504_high_coverage/data/ERR3239757/NA19435.final.cram
**NA19436**	1000G_2504_high_coverage/data/ERR3239758/NA19436.final.cram
**NA19437**	1000G_2504_high_coverage/data/ERR3239759/NA19437.final.cram
**NA19438**	1000G_2504_high_coverage/data/ERR3239760/NA19438.final.cram
**NA19439**	1000G_2504_high_coverage/data/ERR3239761/NA19439.final.cram
**NA19440**	1000G_2504_high_coverage/data/ERR3239762/NA19440.final.cram
**NA19443**	1000G_2504_high_coverage/data/ERR3239763/NA19443.final.cram
**NA19445**	1000G_2504_high_coverage/data/ERR3239764/NA19445.final.cram
**NA19446**	1000G_2504_high_coverage/data/ERR3239765/NA19446.final.cram
**NA19448**	1000G_2504_high_coverage/data/ERR3239766/NA19448.final.cram
**NA19449**	1000G_2504_high_coverage/data/ERR3239767/NA19449.final.cram
**NA19451**	1000G_2504_high_coverage/data/ERR3239768/NA19451.final.cram
**NA19452**	1000G_2504_high_coverage/data/ERR3239769/NA19452.final.cram
**NA19454**	1000G_2504_high_coverage/data/ERR3239770/NA19454.final.cram
**NA19455**	1000G_2504_high_coverage/data/ERR3239771/NA19455.final.cram
**NA19456**	1000G_2504_high_coverage/data/ERR3239772/NA19456.final.cram
**NA19457**	1000G_2504_high_coverage/data/ERR3239773/NA19457.final.cram
**NA19461**	1000G_2504_high_coverage/data/ERR3239774/NA19461.final.cram
**NA19462**	1000G_2504_high_coverage/data/ERR3239775/NA19462.final.cram
**NA19463**	1000G_2504_high_coverage/data/ERR3239776/NA19463.final.cram
**NA19466**	1000G_2504_high_coverage/data/ERR3239777/NA19466.final.cram
**NA19467**	1000G_2504_high_coverage/data/ERR3239778/NA19467.final.cram
**NA19468**	1000G_2504_high_coverage/data/ERR3239779/NA19468.final.cram
**NA19471**	1000G_2504_high_coverage/data/ERR3239780/NA19471.final.cram
**NA19472**	1000G_2504_high_coverage/data/ERR3239781/NA19472.final.cram
**NA19473**	1000G_2504_high_coverage/data/ERR3239782/NA19473.final.cram
**NA19474**	1000G_2504_high_coverage/data/ERR3239783/NA19474.final.cram
**NA19475**	1000G_2504_high_coverage/data/ERR3239784/NA19475.final.cram
**NA20502**	1000G_2504_high_coverage/data/ERR3239785/NA20502.final.cram
**NA20503**	1000G_2504_high_coverage/data/ERR3239786/NA20503.final.cram
**NA20504**	1000G_2504_high_coverage/data/ERR3239787/NA20504.final.cram
**NA20505**	1000G_2504_high_coverage/data/ERR3239788/NA20505.final.cram
**NA20506**	1000G_2504_high_coverage/data/ERR3239789/NA20506.final.cram
**NA20507**	1000G_2504_high_coverage/data/ERR3239790/NA20507.final.cram
**NA20508**	1000G_2504_high_coverage/data/ERR3239791/NA20508.final.cram
**NA20509**	1000G_2504_high_coverage/data/ERR3239792/NA20509.final.cram

**NA20510**	1000G_2504_high_coverage/data/ERR3239793/NA20510.final.cram
**NA20511**	1000G_2504_high_coverage/data/ERR3239794/NA20511.final.cram
**NA20512**	1000G_2504_high_coverage/data/ERR3239795/NA20512.final.cram
**NA20513**	1000G_2504_high_coverage/data/ERR3239796/NA20513.final.cram
**NA20514**	1000G_2504_high_coverage/data/ERR3239797/NA20514.final.cram
**NA20515**	1000G_2504_high_coverage/data/ERR3239798/NA20515.final.cram
**NA20516**	1000G_2504_high_coverage/data/ERR3239799/NA20516.final.cram
**NA20517**	1000G_2504_high_coverage/data/ERR3239800/NA20517.final.cram
**NA20518**	1000G_2504_high_coverage/data/ERR3239801/NA20518.final.cram
**NA20519**	1000G_2504_high_coverage/data/ERR3239802/NA20519.final.cram
**NA20520**	1000G_2504_high_coverage/data/ERR3239803/NA20520.final.cram
**NA20521**	1000G_2504_high_coverage/data/ERR3239804/NA20521.final.cram
**NA20522**	1000G_2504_high_coverage/data/ERR3239805/NA20522.final.cram
**NA20524**	1000G_2504_high_coverage/data/ERR3239806/NA20524.final.cram
**NA20525**	1000G_2504_high_coverage/data/ERR3239807/NA20525.final.cram
**NA20527**	1000G_2504_high_coverage/data/ERR3239808/NA20527.final.cram
**NA20528**	1000G_2504_high_coverage/data/ERR3239809/NA20528.final.cram
**NA20529**	1000G_2504_high_coverage/data/ERR3239810/NA20529.final.cram
**NA20530**	1000G_2504_high_coverage/data/ERR3239811/NA20530.final.cram
**NA20531**	1000G_2504_high_coverage/data/ERR3239812/NA20531.final.cram
**NA20532**	1000G_2504_high_coverage/data/ERR3239813/NA20532.final.cram
**NA20533**	1000G_2504_high_coverage/data/ERR3239814/NA20533.final.cram
**NA20534**	1000G_2504_high_coverage/data/ERR3239815/NA20534.final.cram
**NA20535**	1000G_2504_high_coverage/data/ERR3239816/NA20535.final.cram
**NA20536**	1000G_2504_high_coverage/data/ERR3239817/NA20536.final.cram
**NA20538**	1000G_2504_high_coverage/data/ERR3239818/NA20538.final.cram
**NA20539**	1000G_2504_high_coverage/data/ERR3239819/NA20539.final.cram
**NA20540**	1000G_2504_high_coverage/data/ERR3239820/NA20540.final.cram
**NA20541**	1000G_2504_high_coverage/data/ERR3239821/NA20541.final.cram
**NA20542**	1000G_2504_high_coverage/data/ERR3239822/NA20542.final.cram
**NA20543**	1000G_2504_high_coverage/data/ERR3239823/NA20543.final.cram
**NA20544**	1000G_2504_high_coverage/data/ERR3239824/NA20544.final.cram
**NA20581**	1000G_2504_high_coverage/data/ERR3239825/NA20581.final.cram
**NA20582**	1000G_2504_high_coverage/data/ERR3239826/NA20582.final.cram
**NA20585**	1000G_2504_high_coverage/data/ERR3239827/NA20585.final.cram
**NA20586**	1000G_2504_high_coverage/data/ERR3239828/NA20586.final.cram
**NA20587**	1000G_2504_high_coverage/data/ERR3239829/NA20587.final.cram
**NA20588**	1000G_2504_high_coverage/data/ERR3239830/NA20588.final.cram
**NA20589**	1000G_2504_high_coverage/data/ERR3239831/NA20589.final.cram
**NA20752**	1000G_2504_high_coverage/data/ERR3239832/NA20752.final.cram
**NA20753**	1000G_2504_high_coverage/data/ERR3239833/NA20753.final.cram
**NA20754**	1000G_2504_high_coverage/data/ERR3239834/NA20754.final.cram
**NA20755**	1000G_2504_high_coverage/data/ERR3239835/NA20755.final.cram
**NA20756**	1000G_2504_high_coverage/data/ERR3239836/NA20756.final.cram
**NA20757**	1000G_2504_high_coverage/data/ERR3239837/NA20757.final.cram
**NA20758**	1000G_2504_high_coverage/data/ERR3239838/NA20758.final.cram
**NA20759**	1000G_2504_high_coverage/data/ERR3239839/NA20759.final.cram
**NA20760**	1000G_2504_high_coverage/data/ERR3239840/NA20760.final.cram
**NA20761**	1000G_2504_high_coverage/data/ERR3239841/NA20761.final.cram
**NA20762**	1000G_2504_high_coverage/data/ERR3239842/NA20762.final.cram
**NA20763**	1000G_2504_high_coverage/data/ERR3239843/NA20763.final.cram
**NA20764**	1000G_2504_high_coverage/data/ERR3239844/NA20764.final.cram
**NA20765**	1000G_2504_high_coverage/data/ERR3239845/NA20765.final.cram
**NA20766**	1000G_2504_high_coverage/data/ERR3239846/NA20766.final.cram
**NA20767**	1000G_2504_high_coverage/data/ERR3239847/NA20767.final.cram
**NA20768**	1000G_2504_high_coverage/data/ERR3239848/NA20768.final.cram
**NA20769**	1000G_2504_high_coverage/data/ERR3239849/NA20769.final.cram
**NA20770**	1000G_2504_high_coverage/data/ERR3239850/NA20770.final.cram
**NA20771**	1000G_2504_high_coverage/data/ERR3239851/NA20771.final.cram
**NA20772**	1000G_2504_high_coverage/data/ERR3239852/NA20772.final.cram
**NA20773**	1000G_2504_high_coverage/data/ERR3239853/NA20773.final.cram
**NA20774**	1000G_2504_high_coverage/data/ERR3239854/NA20774.final.cram
**NA20775**	1000G_2504_high_coverage/data/ERR3239855/NA20775.final.cram
**NA20778**	1000G_2504_high_coverage/data/ERR3239856/NA20778.final.cram
**NA20783**	1000G_2504_high_coverage/data/ERR3239857/NA20783.final.cram

**NA20785**	1000G_2504_high_coverage/data/ERR3239858/NA20785.final.cram
**NA20786**	1000G_2504_high_coverage/data/ERR3239859/NA20786.final.cram
**NA20787**	1000G_2504_high_coverage/data/ERR3239860/NA20787.final.cram
**NA20790**	1000G_2504_high_coverage/data/ERR3239861/NA20790.final.cram
**NA20792**	1000G_2504_high_coverage/data/ERR3239862/NA20792.final.cram
**NA20795**	1000G_2504_high_coverage/data/ERR3239863/NA20795.final.cram
**NA20796**	1000G_2504_high_coverage/data/ERR3239864/NA20796.final.cram
**NA20797**	1000G_2504_high_coverage/data/ERR3239865/NA20797.final.cram
**NA20798**	1000G_2504_high_coverage/data/ERR3239866/NA20798.final.cram
**NA20799**	1000G_2504_high_coverage/data/ERR3239867/NA20799.final.cram
**NA20800**	1000G_2504_high_coverage/data/ERR3239868/NA20800.final.cram
**NA20801**	1000G_2504_high_coverage/data/ERR3239869/NA20801.final.cram
**NA20802**	1000G_2504_high_coverage/data/ERR3239870/NA20802.final.cram
**NA20803**	1000G_2504_high_coverage/data/ERR3239871/NA20803.final.cram
**NA20804**	1000G_2504_high_coverage/data/ERR3239872/NA20804.final.cram
**NA20805**	1000G_2504_high_coverage/data/ERR3239873/NA20805.final.cram
**NA20806**	1000G_2504_high_coverage/data/ERR3239874/NA20806.final.cram
**NA20807**	1000G_2504_high_coverage/data/ERR3239875/NA20807.final.cram
**NA20808**	1000G_2504_high_coverage/data/ERR3239876/NA20808.final.cram
**NA20809**	1000G_2504_high_coverage/data/ERR3239877/NA20809.final.cram
**NA20810**	1000G_2504_high_coverage/data/ERR3239878/NA20810.final.cram
**NA20811**	1000G_2504_high_coverage/data/ERR3239879/NA20811.final.cram
**NA20812**	1000G_2504_high_coverage/data/ERR3239880/NA20812.final.cram
**NA20813**	1000G_2504_high_coverage/data/ERR3239881/NA20813.final.cram
**NA20814**	1000G_2504_high_coverage/data/ERR3239882/NA20814.final.cram
**NA20815**	1000G_2504_high_coverage/data/ERR3239883/NA20815.final.cram
**NA20818**	1000G_2504_high_coverage/data/ERR3239884/NA20818.final.cram
**NA20819**	1000G_2504_high_coverage/data/ERR3239885/NA20819.final.cram
**NA20821**	1000G_2504_high_coverage/data/ERR3239886/NA20821.final.cram
**NA20822**	1000G_2504_high_coverage/data/ERR3239887/NA20822.final.cram
**NA20826**	1000G_2504_high_coverage/data/ERR3239888/NA20826.final.cram
**NA20827**	1000G_2504_high_coverage/data/ERR3239889/NA20827.final.cram
**NA20828**	1000G_2504_high_coverage/data/ERR3239890/NA20828.final.cram
**NA20832**	1000G_2504_high_coverage/data/ERR3239891/NA20832.final.cram
**NA19222**	1000G_2504_high_coverage/data/ERR3239892/NA19222.final.cram
**NA19625**	1000G_2504_high_coverage/data/ERR3239893/NA19625.final.cram
**NA19648**	1000G_2504_high_coverage/data/ERR3239894/NA19648.final.cram
**NA19649**	1000G_2504_high_coverage/data/ERR3239895/NA19649.final.cram
**NA19651**	1000G_2504_high_coverage/data/ERR3239896/NA19651.final.cram
**NA19652**	1000G_2504_high_coverage/data/ERR3239897/NA19652.final.cram
**NA19654**	1000G_2504_high_coverage/data/ERR3239898/NA19654.final.cram
**NA19655**	1000G_2504_high_coverage/data/ERR3239899/NA19655.final.cram
**NA19657**	1000G_2504_high_coverage/data/ERR3239900/NA19657.final.cram
**NA19658**	1000G_2504_high_coverage/data/ERR3239901/NA19658.final.cram
**NA19661**	1000G_2504_high_coverage/data/ERR3239902/NA19661.final.cram
**NA19663**	1000G_2504_high_coverage/data/ERR3239903/NA19663.final.cram
**NA19664**	1000G_2504_high_coverage/data/ERR3239904/NA19664.final.cram
**NA19669**	1000G_2504_high_coverage/data/ERR3239905/NA19669.final.cram
**NA19670**	1000G_2504_high_coverage/data/ERR3239906/NA19670.final.cram
**NA19676**	1000G_2504_high_coverage/data/ERR3239907/NA19676.final.cram
**NA19678**	1000G_2504_high_coverage/data/ERR3239908/NA19678.final.cram
**NA19679**	1000G_2504_high_coverage/data/ERR3239909/NA19679.final.cram
**NA19681**	1000G_2504_high_coverage/data/ERR3239910/NA19681.final.cram
**NA19682**	1000G_2504_high_coverage/data/ERR3239911/NA19682.final.cram
**NA19684**	1000G_2504_high_coverage/data/ERR3239912/NA19684.final.cram
**NA19700**	1000G_2504_high_coverage/data/ERR3239913/NA19700.final.cram
**NA19701**	1000G_2504_high_coverage/data/ERR3239914/NA19701.final.cram
**NA19703**	1000G_2504_high_coverage/data/ERR3239915/NA19703.final.cram
**NA19704**	1000G_2504_high_coverage/data/ERR3239916/NA19704.final.cram
**NA19707**	1000G_2504_high_coverage/data/ERR3239917/NA19707.final.cram
**NA19711**	1000G_2504_high_coverage/data/ERR3239918/NA19711.final.cram
**NA19712**	1000G_2504_high_coverage/data/ERR3239919/NA19712.final.cram
**NA19713**	1000G_2504_high_coverage/data/ERR3239920/NA19713.final.cram
**NA19716**	1000G_2504_high_coverage/data/ERR3239921/NA19716.final.cram
**NA19717**	1000G_2504_high_coverage/data/ERR3239922/NA19717.final.cram

**NA19719**	1000G_2504_high_coverage/data/ERR3239923/NA19719.final.cram
**NA19720**	1000G_2504_high_coverage/data/ERR3239924/NA19720.final.cram
**NA19722**	1000G_2504_high_coverage/data/ERR3239925/NA19722.final.cram
**NA19723**	1000G_2504_high_coverage/data/ERR3239926/NA19723.final.cram
**NA19725**	1000G_2504_high_coverage/data/ERR3239927/NA19725.final.cram
**NA19726**	1000G_2504_high_coverage/data/ERR3239928/NA19726.final.cram
**NA19728**	1000G_2504_high_coverage/data/ERR3239929/NA19728.final.cram
**NA19729**	1000G_2504_high_coverage/data/ERR3239930/NA19729.final.cram
**NA19731**	1000G_2504_high_coverage/data/ERR3239931/NA19731.final.cram
**NA19732**	1000G_2504_high_coverage/data/ERR3239932/NA19732.final.cram
**NA19746**	1000G_2504_high_coverage/data/ERR3239933/NA19746.final.cram
**NA19747**	1000G_2504_high_coverage/data/ERR3239934/NA19747.final.cram
**NA19749**	1000G_2504_high_coverage/data/ERR3239935/NA19749.final.cram
**NA19750**	1000G_2504_high_coverage/data/ERR3239936/NA19750.final.cram
**NA19755**	1000G_2504_high_coverage/data/ERR3239937/NA19755.final.cram
**NA19756**	1000G_2504_high_coverage/data/ERR3239938/NA19756.final.cram
**NA19758**	1000G_2504_high_coverage/data/ERR3239939/NA19758.final.cram
**NA19759**	1000G_2504_high_coverage/data/ERR3239940/NA19759.final.cram
**NA19761**	1000G_2504_high_coverage/data/ERR3239941/NA19761.final.cram
**NA19762**	1000G_2504_high_coverage/data/ERR3239942/NA19762.final.cram
**NA19770**	1000G_2504_high_coverage/data/ERR3239943/NA19770.final.cram
**NA19771**	1000G_2504_high_coverage/data/ERR3239944/NA19771.final.cram
**NA19773**	1000G_2504_high_coverage/data/ERR3239945/NA19773.final.cram
**NA19774**	1000G_2504_high_coverage/data/ERR3239946/NA19774.final.cram
**NA19776**	1000G_2504_high_coverage/data/ERR3239947/NA19776.final.cram
**NA19777**	1000G_2504_high_coverage/data/ERR3239948/NA19777.final.cram
**NA19779**	1000G_2504_high_coverage/data/ERR3239949/NA19779.final.cram
**NA19780**	1000G_2504_high_coverage/data/ERR3239950/NA19780.final.cram
**NA19782**	1000G_2504_high_coverage/data/ERR3239951/NA19782.final.cram
**NA19783**	1000G_2504_high_coverage/data/ERR3239952/NA19783.final.cram
**NA19785**	1000G_2504_high_coverage/data/ERR3239953/NA19785.final.cram
**NA19786**	1000G_2504_high_coverage/data/ERR3239954/NA19786.final.cram
**NA19788**	1000G_2504_high_coverage/data/ERR3239955/NA19788.final.cram
**NA19789**	1000G_2504_high_coverage/data/ERR3239956/NA19789.final.cram
**NA19794**	1000G_2504_high_coverage/data/ERR3239957/NA19794.final.cram
**NA19795**	1000G_2504_high_coverage/data/ERR3239958/NA19795.final.cram
**NA19818**	1000G_2504_high_coverage/data/ERR3239959/NA19818.final.cram
**NA19819**	1000G_2504_high_coverage/data/ERR3239960/NA19819.final.cram
**NA19834**	1000G_2504_high_coverage/data/ERR3239961/NA19834.final.cram
**NA19835**	1000G_2504_high_coverage/data/ERR3239962/NA19835.final.cram
**NA19900**	1000G_2504_high_coverage/data/ERR3239963/NA19900.final.cram
**NA19901**	1000G_2504_high_coverage/data/ERR3239964/NA19901.final.cram
**NA19904**	1000G_2504_high_coverage/data/ERR3239965/NA19904.final.cram
**NA19908**	1000G_2504_high_coverage/data/ERR3239966/NA19908.final.cram
**NA19909**	1000G_2504_high_coverage/data/ERR3239967/NA19909.final.cram
**NA19914**	1000G_2504_high_coverage/data/ERR3239968/NA19914.final.cram
**NA19916**	1000G_2504_high_coverage/data/ERR3239969/NA19916.final.cram
**NA19917**	1000G_2504_high_coverage/data/ERR3239970/NA19917.final.cram
**NA19920**	1000G_2504_high_coverage/data/ERR3239971/NA19920.final.cram
**NA19921**	1000G_2504_high_coverage/data/ERR3239972/NA19921.final.cram
**NA19982**	1000G_2504_high_coverage/data/ERR3239973/NA19982.final.cram
**NA20126**	1000G_2504_high_coverage/data/ERR3239974/NA20126.final.cram
**NA20127**	1000G_2504_high_coverage/data/ERR3239975/NA20127.final.cram
**NA20276**	1000G_2504_high_coverage/data/ERR3239976/NA20276.final.cram
**NA20278**	1000G_2504_high_coverage/data/ERR3239977/NA20278.final.cram
**NA20281**	1000G_2504_high_coverage/data/ERR3239978/NA20281.final.cram
**NA20282**	1000G_2504_high_coverage/data/ERR3239979/NA20282.final.cram
**NA20287**	1000G_2504_high_coverage/data/ERR3239980/NA20287.final.cram
**NA20289**	1000G_2504_high_coverage/data/ERR3239981/NA20289.final.cram
**NA20291**	1000G_2504_high_coverage/data/ERR3239982/NA20291.final.cram
**NA20294**	1000G_2504_high_coverage/data/ERR3239983/NA20294.final.cram
**NA20296**	1000G_2504_high_coverage/data/ERR3239984/NA20296.final.cram
**NA20299**	1000G_2504_high_coverage/data/ERR3239985/NA20299.final.cram
**NA20314**	1000G_2504_high_coverage/data/ERR3239986/NA20314.final.cram
**NA20317**	1000G_2504_high_coverage/data/ERR3239987/NA20317.final.cram

**NA20332**	1000G_2504_high_coverage/data/ERR3239988/NA20332.final.cram
**NA20334**	1000G_2504_high_coverage/data/ERR3239989/NA20334.final.cram
**NA20340**	1000G_2504_high_coverage/data/ERR3239990/NA20340.final.cram
**NA20342**	1000G_2504_high_coverage/data/ERR3239991/NA20342.final.cram
**NA20346**	1000G_2504_high_coverage/data/ERR3239992/NA20346.final.cram
**NA20348**	1000G_2504_high_coverage/data/ERR3239993/NA20348.final.cram
**NA20356**	1000G_2504_high_coverage/data/ERR3239994/NA20356.final.cram
**NA20357**	1000G_2504_high_coverage/data/ERR3239995/NA20357.final.cram
**NA20359**	1000G_2504_high_coverage/data/ERR3239996/NA20359.final.cram
**NA20845**	1000G_2504_high_coverage/data/ERR3239997/NA20845.final.cram
**NA20846**	1000G_2504_high_coverage/data/ERR3239998/NA20846.final.cram
**NA20847**	1000G_2504_high_coverage/data/ERR3239999/NA20847.final.cram
**NA20849**	1000G_2504_high_coverage/data/ERR3240000/NA20849.final.cram
**NA20850**	1000G_2504_high_coverage/data/ERR3240001/NA20850.final.cram
**NA20851**	1000G_2504_high_coverage/data/ERR3240002/NA20851.final.cram
**NA20852**	1000G_2504_high_coverage/data/ERR3240003/NA20852.final.cram
**NA20853**	1000G_2504_high_coverage/data/ERR3240004/NA20853.final.cram
**NA20854**	1000G_2504_high_coverage/data/ERR3240005/NA20854.final.cram
**NA20856**	1000G_2504_high_coverage/data/ERR3240006/NA20856.final.cram
**NA20858**	1000G_2504_high_coverage/data/ERR3240007/NA20858.final.cram
**NA20859**	1000G_2504_high_coverage/data/ERR3240008/NA20859.final.cram
**NA20861**	1000G_2504_high_coverage/data/ERR3240009/NA20861.final.cram
**NA20862**	1000G_2504_high_coverage/data/ERR3240010/NA20862.final.cram
**NA20866**	1000G_2504_high_coverage/data/ERR3240011/NA20866.final.cram
**NA20869**	1000G_2504_high_coverage/data/ERR3240012/NA20869.final.cram
**NA20870**	1000G_2504_high_coverage/data/ERR3240013/NA20870.final.cram
**NA20872**	1000G_2504_high_coverage/data/ERR3240014/NA20872.final.cram
**NA20874**	1000G_2504_high_coverage/data/ERR3240015/NA20874.final.cram
**NA20875**	1000G_2504_high_coverage/data/ERR3240016/NA20875.final.cram
**NA20876**	1000G_2504_high_coverage/data/ERR3240017/NA20876.final.cram
**NA20877**	1000G_2504_high_coverage/data/ERR3240018/NA20877.final.cram
**NA20878**	1000G_2504_high_coverage/data/ERR3240019/NA20878.final.cram
**NA20881**	1000G_2504_high_coverage/data/ERR3240020/NA20881.final.cram
**NA20882**	1000G_2504_high_coverage/data/ERR3240021/NA20882.final.cram
**NA20884**	1000G_2504_high_coverage/data/ERR3240022/NA20884.final.cram
**NA20885**	1000G_2504_high_coverage/data/ERR3240023/NA20885.final.cram
**NA20886**	1000G_2504_high_coverage/data/ERR3240024/NA20886.final.cram
**NA20887**	1000G_2504_high_coverage/data/ERR3240025/NA20887.final.cram
**NA20888**	1000G_2504_high_coverage/data/ERR3240026/NA20888.final.cram
**NA20889**	1000G_2504_high_coverage/data/ERR3240027/NA20889.final.cram
**NA20890**	1000G_2504_high_coverage/data/ERR3240028/NA20890.final.cram
**NA20891**	1000G_2504_high_coverage/data/ERR3240029/NA20891.final.cram
**NA20892**	1000G_2504_high_coverage/data/ERR3240030/NA20892.final.cram
**NA20894**	1000G_2504_high_coverage/data/ERR3240031/NA20894.final.cram
**NA20895**	1000G_2504_high_coverage/data/ERR3240032/NA20895.final.cram
**NA20896**	1000G_2504_high_coverage/data/ERR3240033/NA20896.final.cram
**NA20897**	1000G_2504_high_coverage/data/ERR3240034/NA20897.final.cram
**NA20899**	1000G_2504_high_coverage/data/ERR3240035/NA20899.final.cram
**NA20900**	1000G_2504_high_coverage/data/ERR3240036/NA20900.final.cram
**NA20901**	1000G_2504_high_coverage/data/ERR3240037/NA20901.final.cram
**NA20902**	1000G_2504_high_coverage/data/ERR3240038/NA20902.final.cram
**NA20903**	1000G_2504_high_coverage/data/ERR3240039/NA20903.final.cram
**NA20904**	1000G_2504_high_coverage/data/ERR3240040/NA20904.final.cram
**NA20906**	1000G_2504_high_coverage/data/ERR3240041/NA20906.final.cram
**NA20908**	1000G_2504_high_coverage/data/ERR3240042/NA20908.final.cram
**NA20910**	1000G_2504_high_coverage/data/ERR3240043/NA20910.final.cram
**NA20911**	1000G_2504_high_coverage/data/ERR3240044/NA20911.final.cram
**NA21086**	1000G_2504_high_coverage/data/ERR3240045/NA21086.final.cram
**NA21088**	1000G_2504_high_coverage/data/ERR3240046/NA21088.final.cram
**NA21089**	1000G_2504_high_coverage/data/ERR3240047/NA21089.final.cram
**NA21090**	1000G_2504_high_coverage/data/ERR3240048/NA21090.final.cram
**NA21091**	1000G_2504_high_coverage/data/ERR3240049/NA21091.final.cram
**NA21092**	1000G_2504_high_coverage/data/ERR3240050/NA21092.final.cram
**NA21094**	1000G_2504_high_coverage/data/ERR3240051/NA21094.final.cram
**NA21097**	1000G_2504_high_coverage/data/ERR3240052/NA21097.final.cram

**NA21098**	1000G_2504_high_coverage/data/ERR3240053/NA21098.final.cram
**NA21099**	1000G_2504_high_coverage/data/ERR3240054/NA21099.final.cram
**NA21100**	1000G_2504_high_coverage/data/ERR3240055/NA21100.final.cram
**NA21101**	1000G_2504_high_coverage/data/ERR3240056/NA21101.final.cram
**NA21102**	1000G_2504_high_coverage/data/ERR3240057/NA21102.final.cram
**NA21103**	1000G_2504_high_coverage/data/ERR3240058/NA21103.final.cram
**NA21104**	1000G_2504_high_coverage/data/ERR3240059/NA21104.final.cram
**NA21105**	1000G_2504_high_coverage/data/ERR3240060/NA21105.final.cram
**NA21106**	1000G_2504_high_coverage/data/ERR3240061/NA21106.final.cram
**NA21107**	1000G_2504_high_coverage/data/ERR3240062/NA21107.final.cram
**NA21108**	1000G_2504_high_coverage/data/ERR3240063/NA21108.final.cram
**NA21109**	1000G_2504_high_coverage/data/ERR3240064/NA21109.final.cram
**NA21110**	1000G_2504_high_coverage/data/ERR3240065/NA21110.final.cram
**NA21111**	1000G_2504_high_coverage/data/ERR3240066/NA21111.final.cram
**NA21112**	1000G_2504_high_coverage/data/ERR3240067/NA21112.final.cram
**NA21113**	1000G_2504_high_coverage/data/ERR3240068/NA21113.final.cram
**NA21115**	1000G_2504_high_coverage/data/ERR3240069/NA21115.final.cram
**NA21116**	1000G_2504_high_coverage/data/ERR3240070/NA21116.final.cram
**NA21117**	1000G_2504_high_coverage/data/ERR3240071/NA21117.final.cram
**NA21118**	1000G_2504_high_coverage/data/ERR3240072/NA21118.final.cram
**NA21119**	1000G_2504_high_coverage/data/ERR3240073/NA21119.final.cram
**NA21120**	1000G_2504_high_coverage/data/ERR3240074/NA21120.final.cram
**NA21122**	1000G_2504_high_coverage/data/ERR3240075/NA21122.final.cram
**NA21123**	1000G_2504_high_coverage/data/ERR3240076/NA21123.final.cram
**NA21125**	1000G_2504_high_coverage/data/ERR3240077/NA21125.final.cram
**NA21127**	1000G_2504_high_coverage/data/ERR3240078/NA21127.final.cram
**NA21128**	1000G_2504_high_coverage/data/ERR3240079/NA21128.final.cram
**NA21130**	1000G_2504_high_coverage/data/ERR3240080/NA21130.final.cram
**NA21133**	1000G_2504_high_coverage/data/ERR3240081/NA21133.final.cram
**NA21135**	1000G_2504_high_coverage/data/ERR3240082/NA21135.final.cram
**NA21137**	1000G_2504_high_coverage/data/ERR3240083/NA21137.final.cram
**NA21141**	1000G_2504_high_coverage/data/ERR3240084/NA21141.final.cram
**NA21142**	1000G_2504_high_coverage/data/ERR3240085/NA21142.final.cram
**NA21143**	1000G_2504_high_coverage/data/ERR3240086/NA21143.final.cram
**NA21144**	1000G_2504_high_coverage/data/ERR3240087/NA21144.final.cram
**NA19734**	1000G_2504_high_coverage/data/ERR3240088/NA19734.final.cram
**NA19735**	1000G_2504_high_coverage/data/ERR3240089/NA19735.final.cram
**NA19740**	1000G_2504_high_coverage/data/ERR3240090/NA19740.final.cram
**NA19741**	1000G_2504_high_coverage/data/ERR3240091/NA19741.final.cram
**NA19752**	1000G_2504_high_coverage/data/ERR3240092/NA19752.final.cram
**NA19764**	1000G_2504_high_coverage/data/ERR3240093/NA19764.final.cram
**NA19792**	1000G_2504_high_coverage/data/ERR3240094/NA19792.final.cram
**NA19922**	1000G_2504_high_coverage/data/ERR3240095/NA19922.final.cram
**NA19923**	1000G_2504_high_coverage/data/ERR3240096/NA19923.final.cram
**NA19984**	1000G_2504_high_coverage/data/ERR3240097/NA19984.final.cram
**NA20298**	1000G_2504_high_coverage/data/ERR3240098/NA20298.final.cram
**NA20339**	1000G_2504_high_coverage/data/ERR3240099/NA20339.final.cram
**NA20351**	1000G_2504_high_coverage/data/ERR3240100/NA20351.final.cram
**NA20412**	1000G_2504_high_coverage/data/ERR3240101/NA20412.final.cram
**NA20863**	1000G_2504_high_coverage/data/ERR3240102/NA20863.final.cram
**NA20864**	1000G_2504_high_coverage/data/ERR3240103/NA20864.final.cram
**NA20867**	1000G_2504_high_coverage/data/ERR3240104/NA20867.final.cram
**NA20868**	1000G_2504_high_coverage/data/ERR3240105/NA20868.final.cram
**NA20905**	1000G_2504_high_coverage/data/ERR3240106/NA20905.final.cram
**NA21087**	1000G_2504_high_coverage/data/ERR3240107/NA21087.final.cram
**NA21093**	1000G_2504_high_coverage/data/ERR3240108/NA21093.final.cram
**NA21095**	1000G_2504_high_coverage/data/ERR3240109/NA21095.final.cram
**NA21114**	1000G_2504_high_coverage/data/ERR3240110/NA21114.final.cram
**NA21124**	1000G_2504_high_coverage/data/ERR3240111/NA21124.final.cram
**NA21126**	1000G_2504_high_coverage/data/ERR3240112/NA21126.final.cram
**NA21129**	1000G_2504_high_coverage/data/ERR3240113/NA21129.final.cram
**HG00096**	1000G_2504_high_coverage/data/ERR3240114/HG00096.final.cram
**HG00097**	1000G_2504_high_coverage/data/ERR3240115/HG00097.final.cram
**HG00099**	1000G_2504_high_coverage/data/ERR3240116/HG00099.final.cram
**HG00100**	1000G_2504_high_coverage/data/ERR3240117/HG00100.final.cram

**HG00101**	1000G_2504_high_coverage/data/ERR3240118/HG00101.final.cram
**HG00102**	1000G_2504_high_coverage/data/ERR3240119/HG00102.final.cram
**HG00103**	1000G_2504_high_coverage/data/ERR3240120/HG00103.final.cram
**HG00105**	1000G_2504_high_coverage/data/ERR3240121/HG00105.final.cram
**HG00106**	1000G_2504_high_coverage/data/ERR3240122/HG00106.final.cram
**HG00107**	1000G_2504_high_coverage/data/ERR3240123/HG00107.final.cram
**HG00108**	1000G_2504_high_coverage/data/ERR3240124/HG00108.final.cram
**HG00109**	1000G_2504_high_coverage/data/ERR3240125/HG00109.final.cram
**HG00110**	1000G_2504_high_coverage/data/ERR3240126/HG00110.final.cram
**HG00111**	1000G_2504_high_coverage/data/ERR3240127/HG00111.final.cram
**HG00112**	1000G_2504_high_coverage/data/ERR3240128/HG00112.final.cram
**HG00113**	1000G_2504_high_coverage/data/ERR3240129/HG00113.final.cram
**HG00114**	1000G_2504_high_coverage/data/ERR3240130/HG00114.final.cram
**HG00115**	1000G_2504_high_coverage/data/ERR3240131/HG00115.final.cram
**HG00116**	1000G_2504_high_coverage/data/ERR3240132/HG00116.final.cram
**HG00117**	1000G_2504_high_coverage/data/ERR3240133/HG00117.final.cram
**HG00120**	1000G_2504_high_coverage/data/ERR3240134/HG00120.final.cram
**HG00127**	1000G_2504_high_coverage/data/ERR3240135/HG00127.final.cram
**HG00128**	1000G_2504_high_coverage/data/ERR3240136/HG00128.final.cram
**HG00129**	1000G_2504_high_coverage/data/ERR3240137/HG00129.final.cram
**HG00130**	1000G_2504_high_coverage/data/ERR3240138/HG00130.final.cram
**HG00131**	1000G_2504_high_coverage/data/ERR3240139/HG00131.final.cram
**HG00132**	1000G_2504_high_coverage/data/ERR3240140/HG00132.final.cram
**HG00133**	1000G_2504_high_coverage/data/ERR3240141/HG00133.final.cram
**HG00136**	1000G_2504_high_coverage/data/ERR3240142/HG00136.final.cram
**HG00137**	1000G_2504_high_coverage/data/ERR3240143/HG00137.final.cram
**HG00138**	1000G_2504_high_coverage/data/ERR3240144/HG00138.final.cram
**HG00139**	1000G_2504_high_coverage/data/ERR3240145/HG00139.final.cram
**HG00140**	1000G_2504_high_coverage/data/ERR3240146/HG00140.final.cram
**HG00141**	1000G_2504_high_coverage/data/ERR3240147/HG00141.final.cram
**HG00142**	1000G_2504_high_coverage/data/ERR3240148/HG00142.final.cram
**HG00143**	1000G_2504_high_coverage/data/ERR3240149/HG00143.final.cram
**HG00145**	1000G_2504_high_coverage/data/ERR3240150/HG00145.final.cram
**HG00146**	1000G_2504_high_coverage/data/ERR3240151/HG00146.final.cram
**HG00148**	1000G_2504_high_coverage/data/ERR3240152/HG00148.final.cram
**HG00149**	1000G_2504_high_coverage/data/ERR3240153/HG00149.final.cram
**HG00150**	1000G_2504_high_coverage/data/ERR3240154/HG00150.final.cram
**HG00151**	1000G_2504_high_coverage/data/ERR3240155/HG00151.final.cram
**HG00155**	1000G_2504_high_coverage/data/ERR3240156/HG00155.final.cram
**HG00157**	1000G_2504_high_coverage/data/ERR3240157/HG00157.final.cram
**HG00158**	1000G_2504_high_coverage/data/ERR3240158/HG00158.final.cram
**HG00160**	1000G_2504_high_coverage/data/ERR3240159/HG00160.final.cram
**HG00171**	1000G_2504_high_coverage/data/ERR3240160/HG00171.final.cram
**HG00173**	1000G_2504_high_coverage/data/ERR3240161/HG00173.final.cram
**HG00174**	1000G_2504_high_coverage/data/ERR3240162/HG00174.final.cram
**HG00176**	1000G_2504_high_coverage/data/ERR3240163/HG00176.final.cram
**HG00177**	1000G_2504_high_coverage/data/ERR3240164/HG00177.final.cram
**HG00178**	1000G_2504_high_coverage/data/ERR3240165/HG00178.final.cram
**HG00179**	1000G_2504_high_coverage/data/ERR3240166/HG00179.final.cram
**HG00180**	1000G_2504_high_coverage/data/ERR3240167/HG00180.final.cram
**HG00181**	1000G_2504_high_coverage/data/ERR3240168/HG00181.final.cram
**HG00182**	1000G_2504_high_coverage/data/ERR3240169/HG00182.final.cram
**HG00183**	1000G_2504_high_coverage/data/ERR3240170/HG00183.final.cram
**HG00185**	1000G_2504_high_coverage/data/ERR3240171/HG00185.final.cram
**HG00186**	1000G_2504_high_coverage/data/ERR3240172/HG00186.final.cram
**HG00187**	1000G_2504_high_coverage/data/ERR3240173/HG00187.final.cram
**HG00188**	1000G_2504_high_coverage/data/ERR3240174/HG00188.final.cram
**HG00189**	1000G_2504_high_coverage/data/ERR3240175/HG00189.final.cram
**HG00190**	1000G_2504_high_coverage/data/ERR3240176/HG00190.final.cram
**HG00233**	1000G_2504_high_coverage/data/ERR3240177/HG00233.final.cram
**HG00234**	1000G_2504_high_coverage/data/ERR3240178/HG00234.final.cram
**HG00235**	1000G_2504_high_coverage/data/ERR3240179/HG00235.final.cram
**HG00238**	1000G_2504_high_coverage/data/ERR3240180/HG00238.final.cram
**HG00240**	1000G_2504_high_coverage/data/ERR3240181/HG00240.final.cram
**HG00255**	1000G_2504_high_coverage/data/ERR3240182/HG00255.final.cram

**HG00442**	1000G_2504_high_coverage/data/ERR3240183/HG00442.final.cram
**HG00443**	1000G_2504_high_coverage/data/ERR3240184/HG00443.final.cram
**HG00445**	1000G_2504_high_coverage/data/ERR3240185/HG00445.final.cram
**HG00446**	1000G_2504_high_coverage/data/ERR3240186/HG00446.final.cram
**HG00448**	1000G_2504_high_coverage/data/ERR3240187/HG00448.final.cram
**HG00449**	1000G_2504_high_coverage/data/ERR3240188/HG00449.final.cram
**HG00451**	1000G_2504_high_coverage/data/ERR3240189/HG00451.final.cram
**HG00452**	1000G_2504_high_coverage/data/ERR3240190/HG00452.final.cram
**HG00463**	1000G_2504_high_coverage/data/ERR3240191/HG00463.final.cram
**HG00464**	1000G_2504_high_coverage/data/ERR3240192/HG00464.final.cram
**HG00118**	1000G_2504_high_coverage/data/ERR3240193/HG00118.final.cram
**HG00119**	1000G_2504_high_coverage/data/ERR3240194/HG00119.final.cram
**HG00121**	1000G_2504_high_coverage/data/ERR3240195/HG00121.final.cram
**HG00122**	1000G_2504_high_coverage/data/ERR3240196/HG00122.final.cram
**HG00123**	1000G_2504_high_coverage/data/ERR3240197/HG00123.final.cram
**HG00125**	1000G_2504_high_coverage/data/ERR3240198/HG00125.final.cram
**HG00126**	1000G_2504_high_coverage/data/ERR3240199/HG00126.final.cram
**HG00159**	1000G_2504_high_coverage/data/ERR3240200/HG00159.final.cram
**HG00231**	1000G_2504_high_coverage/data/ERR3240201/HG00231.final.cram
**HG00232**	1000G_2504_high_coverage/data/ERR3240202/HG00232.final.cram
**HG00236**	1000G_2504_high_coverage/data/ERR3240203/HG00236.final.cram
**HG00237**	1000G_2504_high_coverage/data/ERR3240204/HG00237.final.cram
**HG00239**	1000G_2504_high_coverage/data/ERR3240205/HG00239.final.cram
**HG00242**	1000G_2504_high_coverage/data/ERR3240206/HG00242.final.cram
**HG00243**	1000G_2504_high_coverage/data/ERR3240207/HG00243.final.cram
**HG00244**	1000G_2504_high_coverage/data/ERR3240208/HG00244.final.cram
**HG00245**	1000G_2504_high_coverage/data/ERR3240209/HG00245.final.cram
**HG00252**	1000G_2504_high_coverage/data/ERR3240210/HG00252.final.cram
**HG00253**	1000G_2504_high_coverage/data/ERR3240211/HG00253.final.cram
**HG00254**	1000G_2504_high_coverage/data/ERR3240212/HG00254.final.cram
**HG00256**	1000G_2504_high_coverage/data/ERR3240213/HG00256.final.cram
**HG00257**	1000G_2504_high_coverage/data/ERR3240214/HG00257.final.cram
**HG00258**	1000G_2504_high_coverage/data/ERR3240215/HG00258.final.cram
**HG00259**	1000G_2504_high_coverage/data/ERR3240216/HG00259.final.cram
**HG00260**	1000G_2504_high_coverage/data/ERR3240217/HG00260.final.cram
**HG00261**	1000G_2504_high_coverage/data/ERR3240218/HG00261.final.cram
**HG00262**	1000G_2504_high_coverage/data/ERR3240219/HG00262.final.cram
**HG00263**	1000G_2504_high_coverage/data/ERR3240220/HG00263.final.cram
**HG00264**	1000G_2504_high_coverage/data/ERR3240221/HG00264.final.cram
**HG00265**	1000G_2504_high_coverage/data/ERR3240222/HG00265.final.cram
**HG00266**	1000G_2504_high_coverage/data/ERR3240223/HG00266.final.cram
**HG00267**	1000G_2504_high_coverage/data/ERR3240224/HG00267.final.cram
**HG00268**	1000G_2504_high_coverage/data/ERR3240225/HG00268.final.cram
**HG00269**	1000G_2504_high_coverage/data/ERR3240226/HG00269.final.cram
**HG00271**	1000G_2504_high_coverage/data/ERR3240227/HG00271.final.cram
**HG00272**	1000G_2504_high_coverage/data/ERR3240228/HG00272.final.cram
**HG00273**	1000G_2504_high_coverage/data/ERR3240229/HG00273.final.cram
**HG00306**	1000G_2504_high_coverage/data/ERR3240230/HG00306.final.cram
**HG00308**	1000G_2504_high_coverage/data/ERR3240231/HG00308.final.cram
**HG00309**	1000G_2504_high_coverage/data/ERR3240232/HG00309.final.cram
**HG00311**	1000G_2504_high_coverage/data/ERR3240233/HG00311.final.cram
**HG00313**	1000G_2504_high_coverage/data/ERR3240234/HG00313.final.cram
**HG00315**	1000G_2504_high_coverage/data/ERR3240235/HG00315.final.cram
**HG00318**	1000G_2504_high_coverage/data/ERR3240236/HG00318.final.cram
**HG00319**	1000G_2504_high_coverage/data/ERR3240237/HG00319.final.cram
**HG00320**	1000G_2504_high_coverage/data/ERR3240238/HG00320.final.cram
**HG00321**	1000G_2504_high_coverage/data/ERR3240239/HG00321.final.cram
**HG00323**	1000G_2504_high_coverage/data/ERR3240240/HG00323.final.cram
**HG00327**	1000G_2504_high_coverage/data/ERR3240241/HG00327.final.cram
**HG00328**	1000G_2504_high_coverage/data/ERR3240242/HG00328.final.cram
**HG00335**	1000G_2504_high_coverage/data/ERR3240243/HG00335.final.cram
**HG00336**	1000G_2504_high_coverage/data/ERR3240244/HG00336.final.cram
**HG00337**	1000G_2504_high_coverage/data/ERR3240245/HG00337.final.cram
**HG00338**	1000G_2504_high_coverage/data/ERR3240246/HG00338.final.cram
**HG00339**	1000G_2504_high_coverage/data/ERR3240247/HG00339.final.cram

**HG00342**	1000G_2504_high_coverage/data/ERR3240248/HG00342.final.cram
**HG00343**	1000G_2504_high_coverage/data/ERR3240249/HG00343.final.cram
**HG00344**	1000G_2504_high_coverage/data/ERR3240250/HG00344.final.cram
**HG00345**	1000G_2504_high_coverage/data/ERR3240251/HG00345.final.cram
**HG00346**	1000G_2504_high_coverage/data/ERR3240252/HG00346.final.cram
**HG00349**	1000G_2504_high_coverage/data/ERR3240253/HG00349.final.cram
**HG00350**	1000G_2504_high_coverage/data/ERR3240254/HG00350.final.cram
**HG00351**	1000G_2504_high_coverage/data/ERR3240255/HG00351.final.cram
**HG00353**	1000G_2504_high_coverage/data/ERR3240256/HG00353.final.cram
**HG00355**	1000G_2504_high_coverage/data/ERR3240257/HG00355.final.cram
**HG00356**	1000G_2504_high_coverage/data/ERR3240258/HG00356.final.cram
**HG00357**	1000G_2504_high_coverage/data/ERR3240259/HG00357.final.cram
**HG00358**	1000G_2504_high_coverage/data/ERR3240260/HG00358.final.cram
**HG00360**	1000G_2504_high_coverage/data/ERR3240261/HG00360.final.cram
**HG00361**	1000G_2504_high_coverage/data/ERR3240262/HG00361.final.cram
**HG00362**	1000G_2504_high_coverage/data/ERR3240263/HG00362.final.cram
**HG00364**	1000G_2504_high_coverage/data/ERR3240264/HG00364.final.cram
**HG00365**	1000G_2504_high_coverage/data/ERR3240265/HG00365.final.cram
**HG00366**	1000G_2504_high_coverage/data/ERR3240266/HG00366.final.cram
**HG00367**	1000G_2504_high_coverage/data/ERR3240267/HG00367.final.cram
**HG00368**	1000G_2504_high_coverage/data/ERR3240268/HG00368.final.cram
**HG00369**	1000G_2504_high_coverage/data/ERR3240269/HG00369.final.cram
**HG00371**	1000G_2504_high_coverage/data/ERR3240270/HG00371.final.cram
**HG00372**	1000G_2504_high_coverage/data/ERR3240271/HG00372.final.cram
**HG00373**	1000G_2504_high_coverage/data/ERR3240272/HG00373.final.cram
**HG00375**	1000G_2504_high_coverage/data/ERR3240273/HG00375.final.cram
**HG00376**	1000G_2504_high_coverage/data/ERR3240274/HG00376.final.cram
**HG00378**	1000G_2504_high_coverage/data/ERR3241659/HG00378.final.cram
**HG00379**	1000G_2504_high_coverage/data/ERR3241660/HG00379.final.cram
**HG00380**	1000G_2504_high_coverage/data/ERR3241661/HG00380.final.cram
**HG00381**	1000G_2504_high_coverage/data/ERR3241662/HG00381.final.cram
**HG00382**	1000G_2504_high_coverage/data/ERR3241663/HG00382.final.cram
**HG00384**	1000G_2504_high_coverage/data/ERR3241664/HG00384.final.cram
**HG00403**	1000G_2504_high_coverage/data/ERR3241665/HG00403.final.cram
**HG00404**	1000G_2504_high_coverage/data/ERR3241666/HG00404.final.cram
**HG00406**	1000G_2504_high_coverage/data/ERR3241667/HG00406.final.cram
**HG00407**	1000G_2504_high_coverage/data/ERR3241668/HG00407.final.cram
**HG00419**	1000G_2504_high_coverage/data/ERR3241669/HG00419.final.cram
**HG00421**	1000G_2504_high_coverage/data/ERR3241670/HG00421.final.cram
**HG00422**	1000G_2504_high_coverage/data/ERR3241671/HG00422.final.cram
**HG00428**	1000G_2504_high_coverage/data/ERR3241672/HG00428.final.cram
**HG00436**	1000G_2504_high_coverage/data/ERR3241673/HG00436.final.cram
**HG00437**	1000G_2504_high_coverage/data/ERR3241674/HG00437.final.cram
**HG00457**	1000G_2504_high_coverage/data/ERR3241675/HG00457.final.cram
**HG00458**	1000G_2504_high_coverage/data/ERR3241676/HG00458.final.cram
**HG00472**	1000G_2504_high_coverage/data/ERR3241677/HG00472.final.cram
**HG00473**	1000G_2504_high_coverage/data/ERR3241678/HG00473.final.cram
**HG00475**	1000G_2504_high_coverage/data/ERR3241679/HG00475.final.cram
**HG00476**	1000G_2504_high_coverage/data/ERR3241680/HG00476.final.cram
**HG00478**	1000G_2504_high_coverage/data/ERR3241681/HG00478.final.cram
**HG00479**	1000G_2504_high_coverage/data/ERR3241682/HG00479.final.cram
**HG00500**	1000G_2504_high_coverage/data/ERR3241683/HG00500.final.cram
**HG00513**	1000G_2504_high_coverage/data/ERR3241684/HG00513.final.cram
**HG00524**	1000G_2504_high_coverage/data/ERR3241685/HG00524.final.cram
**HG00525**	1000G_2504_high_coverage/data/ERR3241686/HG00525.final.cram
**HG00530**	1000G_2504_high_coverage/data/ERR3241687/HG00530.final.cram
**HG00531**	1000G_2504_high_coverage/data/ERR3241688/HG00531.final.cram
**HG00533**	1000G_2504_high_coverage/data/ERR3241689/HG00533.final.cram
**HG00534**	1000G_2504_high_coverage/data/ERR3241690/HG00534.final.cram
**HG00536**	1000G_2504_high_coverage/data/ERR3241691/HG00536.final.cram
**HG00537**	1000G_2504_high_coverage/data/ERR3241692/HG00537.final.cram
**HG00542**	1000G_2504_high_coverage/data/ERR3241693/HG00542.final.cram
**HG00543**	1000G_2504_high_coverage/data/ERR3241694/HG00543.final.cram
**HG00553**	1000G_2504_high_coverage/data/ERR3241695/HG00553.final.cram
**HG00554**	1000G_2504_high_coverage/data/ERR3241696/HG00554.final.cram

**HG00556**	1000G_2504_high_coverage/data/ERR3241697/HG00556.final.cram
**HG00557**	1000G_2504_high_coverage/data/ERR3241698/HG00557.final.cram
**HG00559**	1000G_2504_high_coverage/data/ERR3241699/HG00559.final.cram
**HG00560**	1000G_2504_high_coverage/data/ERR3241700/HG00560.final.cram
**HG00565**	1000G_2504_high_coverage/data/ERR3241701/HG00565.final.cram
**HG00566**	1000G_2504_high_coverage/data/ERR3241702/HG00566.final.cram
**HG00580**	1000G_2504_high_coverage/data/ERR3241703/HG00580.final.cram
**HG00581**	1000G_2504_high_coverage/data/ERR3241704/HG00581.final.cram
**HG00583**	1000G_2504_high_coverage/data/ERR3241705/HG00583.final.cram
**HG00584**	1000G_2504_high_coverage/data/ERR3241706/HG00584.final.cram
**HG00589**	1000G_2504_high_coverage/data/ERR3241707/HG00589.final.cram
**HG00590**	1000G_2504_high_coverage/data/ERR3241708/HG00590.final.cram
**HG00592**	1000G_2504_high_coverage/data/ERR3241709/HG00592.final.cram
**HG00593**	1000G_2504_high_coverage/data/ERR3241710/HG00593.final.cram
**HG00595**	1000G_2504_high_coverage/data/ERR3241711/HG00595.final.cram
**HG00596**	1000G_2504_high_coverage/data/ERR3241712/HG00596.final.cram
**HG00607**	1000G_2504_high_coverage/data/ERR3241713/HG00607.final.cram
**HG00608**	1000G_2504_high_coverage/data/ERR3241714/HG00608.final.cram
**HG00610**	1000G_2504_high_coverage/data/ERR3241715/HG00610.final.cram
**HG00611**	1000G_2504_high_coverage/data/ERR3241716/HG00611.final.cram
**HG00613**	1000G_2504_high_coverage/data/ERR3241717/HG00613.final.cram
**HG00614**	1000G_2504_high_coverage/data/ERR3241718/HG00614.final.cram
**HG00619**	1000G_2504_high_coverage/data/ERR3241719/HG00619.final.cram
**HG00620**	1000G_2504_high_coverage/data/ERR3241720/HG00620.final.cram
**HG00625**	1000G_2504_high_coverage/data/ERR3241721/HG00625.final.cram
**HG00626**	1000G_2504_high_coverage/data/ERR3241722/HG00626.final.cram
**HG00628**	1000G_2504_high_coverage/data/ERR3241723/HG00628.final.cram
**HG00629**	1000G_2504_high_coverage/data/ERR3241724/HG00629.final.cram
**HG00634**	1000G_2504_high_coverage/data/ERR3241725/HG00634.final.cram
**HG00637**	1000G_2504_high_coverage/data/ERR3241726/HG00637.final.cram
**HG00638**	1000G_2504_high_coverage/data/ERR3241727/HG00638.final.cram
**HG00640**	1000G_2504_high_coverage/data/ERR3241728/HG00640.final.cram
**HG00641**	1000G_2504_high_coverage/data/ERR3241729/HG00641.final.cram
**HG00650**	1000G_2504_high_coverage/data/ERR3241730/HG00650.final.cram
**HG00651**	1000G_2504_high_coverage/data/ERR3241731/HG00651.final.cram
**HG00653**	1000G_2504_high_coverage/data/ERR3241732/HG00653.final.cram
**HG00654**	1000G_2504_high_coverage/data/ERR3241733/HG00654.final.cram
**HG00656**	1000G_2504_high_coverage/data/ERR3241734/HG00656.final.cram
**HG00657**	1000G_2504_high_coverage/data/ERR3241735/HG00657.final.cram
**HG00662**	1000G_2504_high_coverage/data/ERR3241736/HG00662.final.cram
**HG00663**	1000G_2504_high_coverage/data/ERR3241737/HG00663.final.cram
**HG00671**	1000G_2504_high_coverage/data/ERR3241738/HG00671.final.cram
**HG00672**	1000G_2504_high_coverage/data/ERR3241739/HG00672.final.cram
**HG00683**	1000G_2504_high_coverage/data/ERR3241740/HG00683.final.cram
**HG00684**	1000G_2504_high_coverage/data/ERR3241741/HG00684.final.cram
**HG00689**	1000G_2504_high_coverage/data/ERR3241742/HG00689.final.cram
**HG00690**	1000G_2504_high_coverage/data/ERR3241743/HG00690.final.cram
**HG00692**	1000G_2504_high_coverage/data/ERR3241744/HG00692.final.cram
**HG00693**	1000G_2504_high_coverage/data/ERR3241745/HG00693.final.cram
**HG00698**	1000G_2504_high_coverage/data/ERR3241746/HG00698.final.cram
**HG00699**	1000G_2504_high_coverage/data/ERR3241747/HG00699.final.cram
**HG00701**	1000G_2504_high_coverage/data/ERR3241748/HG00701.final.cram
**HG00704**	1000G_2504_high_coverage/data/ERR3241749/HG00704.final.cram
**HG00705**	1000G_2504_high_coverage/data/ERR3241750/HG00705.final.cram
**HG00707**	1000G_2504_high_coverage/data/ERR3241751/HG00707.final.cram
**HG00708**	1000G_2504_high_coverage/data/ERR3241752/HG00708.final.cram
**HG00717**	1000G_2504_high_coverage/data/ERR3241753/HG00717.final.cram
**HG00731**	1000G_2504_high_coverage/data/ERR3241754/HG00731.final.cram
**HG00732**	1000G_2504_high_coverage/data/ERR3241755/HG00732.final.cram
**HG00736**	1000G_2504_high_coverage/data/ERR3241756/HG00736.final.cram
**HG00737**	1000G_2504_high_coverage/data/ERR3241757/HG00737.final.cram
**HG00739**	1000G_2504_high_coverage/data/ERR3241758/HG00739.final.cram
**HG00740**	1000G_2504_high_coverage/data/ERR3241759/HG00740.final.cram
**HG01047**	1000G_2504_high_coverage/data/ERR3241760/HG01047.final.cram
**HG01048**	1000G_2504_high_coverage/data/ERR3241761/HG01048.final.cram

**HG01049**	1000G_2504_high_coverage/data/ERR3241762/HG01049.final.cram
**HG01054**	1000G_2504_high_coverage/data/ERR3241763/HG01054.final.cram
**HG01055**	1000G_2504_high_coverage/data/ERR3241764/HG01055.final.cram
**HG01066**	1000G_2504_high_coverage/data/ERR3241765/HG01066.final.cram
**HG01067**	1000G_2504_high_coverage/data/ERR3241766/HG01067.final.cram
**HG01079**	1000G_2504_high_coverage/data/ERR3241767/HG01079.final.cram
**HG01080**	1000G_2504_high_coverage/data/ERR3241768/HG01080.final.cram
**HG01097**	1000G_2504_high_coverage/data/ERR3241769/HG01097.final.cram
**HG01098**	1000G_2504_high_coverage/data/ERR3241770/HG01098.final.cram
**NA19913**	1000G_2504_high_coverage/data/ERR3241771/NA19913.final.cram
**NA20274**	1000G_2504_high_coverage/data/ERR3241772/NA20274.final.cram
**NA20318**	1000G_2504_high_coverage/data/ERR3241773/NA20318.final.cram
**NA20320**	1000G_2504_high_coverage/data/ERR3241774/NA20320.final.cram
**NA20321**	1000G_2504_high_coverage/data/ERR3241775/NA20321.final.cram
**NA20355**	1000G_2504_high_coverage/data/ERR3241776/NA20355.final.cram
**NA20362**	1000G_2504_high_coverage/data/ERR3241777/NA20362.final.cram
**HG00734**	1000G_2504_high_coverage/data/ERR3241778/HG00734.final.cram
**HG00154**	1000G_2504_high_coverage/data/ERR3241779/HG00154.final.cram
**HG00246**	1000G_2504_high_coverage/data/ERR3241780/HG00246.final.cram
**HG00250**	1000G_2504_high_coverage/data/ERR3241781/HG00250.final.cram
**HG00251**	1000G_2504_high_coverage/data/ERR3241782/HG00251.final.cram
**HG00274**	1000G_2504_high_coverage/data/ERR3241783/HG00274.final.cram
**HG00275**	1000G_2504_high_coverage/data/ERR3241784/HG00275.final.cram
**HG00276**	1000G_2504_high_coverage/data/ERR3241785/HG00276.final.cram
**HG00277**	1000G_2504_high_coverage/data/ERR3241786/HG00277.final.cram
**HG00278**	1000G_2504_high_coverage/data/ERR3241787/HG00278.final.cram
**HG00280**	1000G_2504_high_coverage/data/ERR3241788/HG00280.final.cram
**HG00281**	1000G_2504_high_coverage/data/ERR3241789/HG00281.final.cram
**HG00282**	1000G_2504_high_coverage/data/ERR3241790/HG00282.final.cram
**HG00284**	1000G_2504_high_coverage/data/ERR3241791/HG00284.final.cram
**HG00285**	1000G_2504_high_coverage/data/ERR3241792/HG00285.final.cram
**HG00310**	1000G_2504_high_coverage/data/ERR3241793/HG00310.final.cram
**HG00324**	1000G_2504_high_coverage/data/ERR3241794/HG00324.final.cram
**HG00325**	1000G_2504_high_coverage/data/ERR3241795/HG00325.final.cram
**HG00326**	1000G_2504_high_coverage/data/ERR3241796/HG00326.final.cram
**HG00329**	1000G_2504_high_coverage/data/ERR3241797/HG00329.final.cram
**HG00330**	1000G_2504_high_coverage/data/ERR3241798/HG00330.final.cram
**HG00331**	1000G_2504_high_coverage/data/ERR3241799/HG00331.final.cram
**HG00332**	1000G_2504_high_coverage/data/ERR3241800/HG00332.final.cram
**HG00334**	1000G_2504_high_coverage/data/ERR3241801/HG00334.final.cram
**HG00341**	1000G_2504_high_coverage/data/ERR3241802/HG00341.final.cram
**HG00383**	1000G_2504_high_coverage/data/ERR3241803/HG00383.final.cram
**HG00551**	1000G_2504_high_coverage/data/ERR3241804/HG00551.final.cram
**HG01051**	1000G_2504_high_coverage/data/ERR3241805/HG01051.final.cram
**HG01052**	1000G_2504_high_coverage/data/ERR3241806/HG01052.final.cram
**HG01060**	1000G_2504_high_coverage/data/ERR3241807/HG01060.final.cram
**HG01061**	1000G_2504_high_coverage/data/ERR3241808/HG01061.final.cram
**HG01069**	1000G_2504_high_coverage/data/ERR3241809/HG01069.final.cram
**HG01070**	1000G_2504_high_coverage/data/ERR3241810/HG01070.final.cram
**HG01072**	1000G_2504_high_coverage/data/ERR3241811/HG01072.final.cram
**HG01073**	1000G_2504_high_coverage/data/ERR3241812/HG01073.final.cram
**HG01075**	1000G_2504_high_coverage/data/ERR3241813/HG01075.final.cram
**HG01082**	1000G_2504_high_coverage/data/ERR3241814/HG01082.final.cram
**HG01083**	1000G_2504_high_coverage/data/ERR3241815/HG01083.final.cram
**HG01085**	1000G_2504_high_coverage/data/ERR3241816/HG01085.final.cram
**HG01086**	1000G_2504_high_coverage/data/ERR3241817/HG01086.final.cram
**HG01094**	1000G_2504_high_coverage/data/ERR3241818/HG01094.final.cram
**HG01095**	1000G_2504_high_coverage/data/ERR3241819/HG01095.final.cram
**HG01101**	1000G_2504_high_coverage/data/ERR3241820/HG01101.final.cram
**HG01102**	1000G_2504_high_coverage/data/ERR3241821/HG01102.final.cram
**HG01104**	1000G_2504_high_coverage/data/ERR3241822/HG01104.final.cram
**HG01105**	1000G_2504_high_coverage/data/ERR3241823/HG01105.final.cram
**HG01107**	1000G_2504_high_coverage/data/ERR3241824/HG01107.final.cram
**HG01108**	1000G_2504_high_coverage/data/ERR3241825/HG01108.final.cram
**HG01110**	1000G_2504_high_coverage/data/ERR3241826/HG01110.final.cram

**HG01111**	1000G_2504_high_coverage/data/ERR3241827/HG01111.final.cram
**HG01112**	1000G_2504_high_coverage/data/ERR3241828/HG01112.final.cram
**HG01113**	1000G_2504_high_coverage/data/ERR3241829/HG01113.final.cram
**HG01124**	1000G_2504_high_coverage/data/ERR3241830/HG01124.final.cram
**HG01125**	1000G_2504_high_coverage/data/ERR3241831/HG01125.final.cram
**HG01133**	1000G_2504_high_coverage/data/ERR3241832/HG01133.final.cram
**HG01134**	1000G_2504_high_coverage/data/ERR3241833/HG01134.final.cram
**HG01136**	1000G_2504_high_coverage/data/ERR3241834/HG01136.final.cram
**HG01137**	1000G_2504_high_coverage/data/ERR3241835/HG01137.final.cram
**HG01139**	1000G_2504_high_coverage/data/ERR3241836/HG01139.final.cram
**HG01140**	1000G_2504_high_coverage/data/ERR3241837/HG01140.final.cram
**HG01148**	1000G_2504_high_coverage/data/ERR3241838/HG01148.final.cram
**HG01149**	1000G_2504_high_coverage/data/ERR3241839/HG01149.final.cram
**HG01167**	1000G_2504_high_coverage/data/ERR3241840/HG01167.final.cram
**HG01168**	1000G_2504_high_coverage/data/ERR3241841/HG01168.final.cram
**HG01170**	1000G_2504_high_coverage/data/ERR3241842/HG01170.final.cram
**HG01171**	1000G_2504_high_coverage/data/ERR3241843/HG01171.final.cram
**HG01173**	1000G_2504_high_coverage/data/ERR3241844/HG01173.final.cram
**HG01174**	1000G_2504_high_coverage/data/ERR3241845/HG01174.final.cram
**HG01176**	1000G_2504_high_coverage/data/ERR3241846/HG01176.final.cram
**HG01177**	1000G_2504_high_coverage/data/ERR3241847/HG01177.final.cram
**HG01182**	1000G_2504_high_coverage/data/ERR3241848/HG01182.final.cram
**HG01183**	1000G_2504_high_coverage/data/ERR3241849/HG01183.final.cram
**HG01187**	1000G_2504_high_coverage/data/ERR3241850/HG01187.final.cram
**HG01188**	1000G_2504_high_coverage/data/ERR3241851/HG01188.final.cram
**HG01190**	1000G_2504_high_coverage/data/ERR3241852/HG01190.final.cram
**HG01191**	1000G_2504_high_coverage/data/ERR3241853/HG01191.final.cram
**HG01197**	1000G_2504_high_coverage/data/ERR3241854/HG01197.final.cram
**HG01198**	1000G_2504_high_coverage/data/ERR3241855/HG01198.final.cram
**HG01204**	1000G_2504_high_coverage/data/ERR3241856/HG01204.final.cram
**HG01205**	1000G_2504_high_coverage/data/ERR3241857/HG01205.final.cram
**HG01241**	1000G_2504_high_coverage/data/ERR3241858/HG01241.final.cram
**HG01242**	1000G_2504_high_coverage/data/ERR3241859/HG01242.final.cram
**HG01247**	1000G_2504_high_coverage/data/ERR3241860/HG01247.final.cram
**HG01248**	1000G_2504_high_coverage/data/ERR3241861/HG01248.final.cram
**HG01250**	1000G_2504_high_coverage/data/ERR3241862/HG01250.final.cram
**HG01251**	1000G_2504_high_coverage/data/ERR3241863/HG01251.final.cram
**HG01253**	1000G_2504_high_coverage/data/ERR3241864/HG01253.final.cram
**HG01254**	1000G_2504_high_coverage/data/ERR3241865/HG01254.final.cram
**HG01256**	1000G_2504_high_coverage/data/ERR3241866/HG01256.final.cram
**HG01257**	1000G_2504_high_coverage/data/ERR3241867/HG01257.final.cram
**HG01259**	1000G_2504_high_coverage/data/ERR3241868/HG01259.final.cram
**HG01260**	1000G_2504_high_coverage/data/ERR3241869/HG01260.final.cram
**HG01271**	1000G_2504_high_coverage/data/ERR3241870/HG01271.final.cram
**HG01272**	1000G_2504_high_coverage/data/ERR3241871/HG01272.final.cram
**HG01275**	1000G_2504_high_coverage/data/ERR3241872/HG01275.final.cram
**HG01277**	1000G_2504_high_coverage/data/ERR3241873/HG01277.final.cram
**HG01334**	1000G_2504_high_coverage/data/ERR3241874/HG01334.final.cram
**HG01344**	1000G_2504_high_coverage/data/ERR3241875/HG01344.final.cram
**HG01345**	1000G_2504_high_coverage/data/ERR3241876/HG01345.final.cram
**HG01348**	1000G_2504_high_coverage/data/ERR3241877/HG01348.final.cram
**HG01356**	1000G_2504_high_coverage/data/ERR3241878/HG01356.final.cram
**HG01357**	1000G_2504_high_coverage/data/ERR3241879/HG01357.final.cram
**HG01377**	1000G_2504_high_coverage/data/ERR3241880/HG01377.final.cram
**HG01378**	1000G_2504_high_coverage/data/ERR3241881/HG01378.final.cram
**HG01383**	1000G_2504_high_coverage/data/ERR3241882/HG01383.final.cram
**HG01384**	1000G_2504_high_coverage/data/ERR3241883/HG01384.final.cram
**HG01341**	1000G_2504_high_coverage/data/ERR3241884/HG01341.final.cram
**HG01342**	1000G_2504_high_coverage/data/ERR3241885/HG01342.final.cram
**HG01350**	1000G_2504_high_coverage/data/ERR3241886/HG01350.final.cram
**HG01351**	1000G_2504_high_coverage/data/ERR3241887/HG01351.final.cram
**HG01353**	1000G_2504_high_coverage/data/ERR3241888/HG01353.final.cram
**HG01354**	1000G_2504_high_coverage/data/ERR3241889/HG01354.final.cram
**HG01359**	1000G_2504_high_coverage/data/ERR3241890/HG01359.final.cram
**HG01360**	1000G_2504_high_coverage/data/ERR3241891/HG01360.final.cram

**HG01365**	1000G_2504_high_coverage/data/ERR3241892/HG01365.final.cram
**HG01366**	1000G_2504_high_coverage/data/ERR3241893/HG01366.final.cram
**HG01374**	1000G_2504_high_coverage/data/ERR3241894/HG01374.final.cram
**HG01375**	1000G_2504_high_coverage/data/ERR3241895/HG01375.final.cram
**HG01389**	1000G_2504_high_coverage/data/ERR3241896/HG01389.final.cram
**HG01390**	1000G_2504_high_coverage/data/ERR3241897/HG01390.final.cram
**HG01437**	1000G_2504_high_coverage/data/ERR3241898/HG01437.final.cram
**HG01438**	1000G_2504_high_coverage/data/ERR3241899/HG01438.final.cram
**HG01440**	1000G_2504_high_coverage/data/ERR3241900/HG01440.final.cram
**HG01441**	1000G_2504_high_coverage/data/ERR3241901/HG01441.final.cram
**HG01455**	1000G_2504_high_coverage/data/ERR3241902/HG01455.final.cram
**HG01456**	1000G_2504_high_coverage/data/ERR3241903/HG01456.final.cram
**HG01461**	1000G_2504_high_coverage/data/ERR3241904/HG01461.final.cram
**HG01462**	1000G_2504_high_coverage/data/ERR3241905/HG01462.final.cram
**HG01464**	1000G_2504_high_coverage/data/ERR3241906/HG01464.final.cram
**HG01465**	1000G_2504_high_coverage/data/ERR3241907/HG01465.final.cram
**HG01488**	1000G_2504_high_coverage/data/ERR3241908/HG01488.final.cram
**HG01489**	1000G_2504_high_coverage/data/ERR3241909/HG01489.final.cram
**HG01491**	1000G_2504_high_coverage/data/ERR3241910/HG01491.final.cram
**HG01492**	1000G_2504_high_coverage/data/ERR3241911/HG01492.final.cram
**HG01494**	1000G_2504_high_coverage/data/ERR3241912/HG01494.final.cram
**HG01495**	1000G_2504_high_coverage/data/ERR3241913/HG01495.final.cram
**HG01497**	1000G_2504_high_coverage/data/ERR3241914/HG01497.final.cram
**HG01498**	1000G_2504_high_coverage/data/ERR3241915/HG01498.final.cram
**HG01500**	1000G_2504_high_coverage/data/ERR3241916/HG01500.final.cram
**HG01501**	1000G_2504_high_coverage/data/ERR3241917/HG01501.final.cram
**HG01503**	1000G_2504_high_coverage/data/ERR3241918/HG01503.final.cram
**HG01504**	1000G_2504_high_coverage/data/ERR3241919/HG01504.final.cram
**HG01506**	1000G_2504_high_coverage/data/ERR3241920/HG01506.final.cram
**HG01507**	1000G_2504_high_coverage/data/ERR3241921/HG01507.final.cram
**HG01509**	1000G_2504_high_coverage/data/ERR3241922/HG01509.final.cram
**HG01510**	1000G_2504_high_coverage/data/ERR3241923/HG01510.final.cram
**HG01512**	1000G_2504_high_coverage/data/ERR3241924/HG01512.final.cram
**HG01513**	1000G_2504_high_coverage/data/ERR3241925/HG01513.final.cram
**HG01515**	1000G_2504_high_coverage/data/ERR3241926/HG01515.final.cram
**HG01516**	1000G_2504_high_coverage/data/ERR3241927/HG01516.final.cram
**HG01518**	1000G_2504_high_coverage/data/ERR3241928/HG01518.final.cram
**HG01519**	1000G_2504_high_coverage/data/ERR3241929/HG01519.final.cram
**HG01521**	1000G_2504_high_coverage/data/ERR3241930/HG01521.final.cram
**HG01522**	1000G_2504_high_coverage/data/ERR3241931/HG01522.final.cram
**HG01524**	1000G_2504_high_coverage/data/ERR3241932/HG01524.final.cram
**HG01525**	1000G_2504_high_coverage/data/ERR3241933/HG01525.final.cram
**HG01527**	1000G_2504_high_coverage/data/ERR3241934/HG01527.final.cram
**HG01528**	1000G_2504_high_coverage/data/ERR3241935/HG01528.final.cram
**HG01530**	1000G_2504_high_coverage/data/ERR3241936/HG01530.final.cram
**HG01531**	1000G_2504_high_coverage/data/ERR3241937/HG01531.final.cram
**HG01536**	1000G_2504_high_coverage/data/ERR3241938/HG01536.final.cram
**HG01537**	1000G_2504_high_coverage/data/ERR3241939/HG01537.final.cram
**HG01550**	1000G_2504_high_coverage/data/ERR3241940/HG01550.final.cram
**HG01551**	1000G_2504_high_coverage/data/ERR3241941/HG01551.final.cram
**HG01602**	1000G_2504_high_coverage/data/ERR3241942/HG01602.final.cram
**HG01603**	1000G_2504_high_coverage/data/ERR3241943/HG01603.final.cram
**HG01605**	1000G_2504_high_coverage/data/ERR3241944/HG01605.final.cram
**HG01606**	1000G_2504_high_coverage/data/ERR3241945/HG01606.final.cram
**HG01607**	1000G_2504_high_coverage/data/ERR3241946/HG01607.final.cram
**HG01608**	1000G_2504_high_coverage/data/ERR3241947/HG01608.final.cram
**HG01610**	1000G_2504_high_coverage/data/ERR3241948/HG01610.final.cram
**HG01612**	1000G_2504_high_coverage/data/ERR3241949/HG01612.final.cram
**HG01613**	1000G_2504_high_coverage/data/ERR3241950/HG01613.final.cram
**HG01615**	1000G_2504_high_coverage/data/ERR3241951/HG01615.final.cram
**HG01617**	1000G_2504_high_coverage/data/ERR3241952/HG01617.final.cram
**HG01618**	1000G_2504_high_coverage/data/ERR3241953/HG01618.final.cram
**HG01619**	1000G_2504_high_coverage/data/ERR3241954/HG01619.final.cram
**HG01620**	1000G_2504_high_coverage/data/ERR3241955/HG01620.final.cram
**HG01623**	1000G_2504_high_coverage/data/ERR3241956/HG01623.final.cram

**HG01624**	1000G_2504_high_coverage/data/ERR3241957/HG01624.final.cram
**HG01625**	1000G_2504_high_coverage/data/ERR3241958/HG01625.final.cram
**HG01626**	1000G_2504_high_coverage/data/ERR3241959/HG01626.final.cram
**HG01628**	1000G_2504_high_coverage/data/ERR3241960/HG01628.final.cram
**HG01630**	1000G_2504_high_coverage/data/ERR3241961/HG01630.final.cram
**HG01631**	1000G_2504_high_coverage/data/ERR3241962/HG01631.final.cram
**HG01632**	1000G_2504_high_coverage/data/ERR3241963/HG01632.final.cram
**HG01668**	1000G_2504_high_coverage/data/ERR3241964/HG01668.final.cram
**HG01669**	1000G_2504_high_coverage/data/ERR3241965/HG01669.final.cram
**HG01670**	1000G_2504_high_coverage/data/ERR3241966/HG01670.final.cram
**HG01672**	1000G_2504_high_coverage/data/ERR3241967/HG01672.final.cram
**HG01673**	1000G_2504_high_coverage/data/ERR3241968/HG01673.final.cram
**HG01675**	1000G_2504_high_coverage/data/ERR3241969/HG01675.final.cram
**HG01676**	1000G_2504_high_coverage/data/ERR3241970/HG01676.final.cram
**HG01678**	1000G_2504_high_coverage/data/ERR3241971/HG01678.final.cram
**HG01679**	1000G_2504_high_coverage/data/ERR3241972/HG01679.final.cram
**HG01680**	1000G_2504_high_coverage/data/ERR3241973/HG01680.final.cram
**HG01682**	1000G_2504_high_coverage/data/ERR3241974/HG01682.final.cram
**HG01684**	1000G_2504_high_coverage/data/ERR3241975/HG01684.final.cram
**HG01685**	1000G_2504_high_coverage/data/ERR3241976/HG01685.final.cram
**HG01686**	1000G_2504_high_coverage/data/ERR3241977/HG01686.final.cram
**HG01700**	1000G_2504_high_coverage/data/ERR3241978/HG01700.final.cram
**HG01702**	1000G_2504_high_coverage/data/ERR3241979/HG01702.final.cram
**HG01704**	1000G_2504_high_coverage/data/ERR3241980/HG01704.final.cram
**HG01705**	1000G_2504_high_coverage/data/ERR3241981/HG01705.final.cram
**HG01707**	1000G_2504_high_coverage/data/ERR3241982/HG01707.final.cram
**HG01708**	1000G_2504_high_coverage/data/ERR3241983/HG01708.final.cram
**HG01565**	1000G_2504_high_coverage/data/ERR3241984/HG01565.final.cram
**HG01566**	1000G_2504_high_coverage/data/ERR3241985/HG01566.final.cram
**HG01571**	1000G_2504_high_coverage/data/ERR3241986/HG01571.final.cram
**HG01572**	1000G_2504_high_coverage/data/ERR3241987/HG01572.final.cram
**HG01577**	1000G_2504_high_coverage/data/ERR3241988/HG01577.final.cram
**HG01578**	1000G_2504_high_coverage/data/ERR3241989/HG01578.final.cram
**HG01917**	1000G_2504_high_coverage/data/ERR3241990/HG01917.final.cram
**HG01918**	1000G_2504_high_coverage/data/ERR3241991/HG01918.final.cram
**HG01892**	1000G_2504_high_coverage/data/ERR3241992/HG01892.final.cram
**HG01893**	1000G_2504_high_coverage/data/ERR3241993/HG01893.final.cram
**HG01920**	1000G_2504_high_coverage/data/ERR3241994/HG01920.final.cram
**HG01921**	1000G_2504_high_coverage/data/ERR3241995/HG01921.final.cram
**HG01923**	1000G_2504_high_coverage/data/ERR3241996/HG01923.final.cram
**HG01924**	1000G_2504_high_coverage/data/ERR3241997/HG01924.final.cram
**HG01926**	1000G_2504_high_coverage/data/ERR3241998/HG01926.final.cram
**HG01927**	1000G_2504_high_coverage/data/ERR3241999/HG01927.final.cram
**HG01932**	1000G_2504_high_coverage/data/ERR3242000/HG01932.final.cram
**HG01933**	1000G_2504_high_coverage/data/ERR3242001/HG01933.final.cram
**HG01935**	1000G_2504_high_coverage/data/ERR3242002/HG01935.final.cram
**HG01936**	1000G_2504_high_coverage/data/ERR3242003/HG01936.final.cram
**HG01938**	1000G_2504_high_coverage/data/ERR3242004/HG01938.final.cram
**HG01939**	1000G_2504_high_coverage/data/ERR3242005/HG01939.final.cram
**HG01941**	1000G_2504_high_coverage/data/ERR3242006/HG01941.final.cram
**HG01942**	1000G_2504_high_coverage/data/ERR3242007/HG01942.final.cram
**HG01944**	1000G_2504_high_coverage/data/ERR3242008/HG01944.final.cram
**HG01945**	1000G_2504_high_coverage/data/ERR3242009/HG01945.final.cram
**HG01947**	1000G_2504_high_coverage/data/ERR3242010/HG01947.final.cram
**HG01948**	1000G_2504_high_coverage/data/ERR3242011/HG01948.final.cram
**HG01950**	1000G_2504_high_coverage/data/ERR3242012/HG01950.final.cram
**HG01951**	1000G_2504_high_coverage/data/ERR3242013/HG01951.final.cram
**HG01953**	1000G_2504_high_coverage/data/ERR3242014/HG01953.final.cram
**HG01954**	1000G_2504_high_coverage/data/ERR3242015/HG01954.final.cram
**HG01967**	1000G_2504_high_coverage/data/ERR3242016/HG01967.final.cram
**HG01968**	1000G_2504_high_coverage/data/ERR3242017/HG01968.final.cram
**HG01970**	1000G_2504_high_coverage/data/ERR3242018/HG01970.final.cram
**HG01971**	1000G_2504_high_coverage/data/ERR3242019/HG01971.final.cram
**HG01974**	1000G_2504_high_coverage/data/ERR3242020/HG01974.final.cram
**HG01973**	1000G_2504_high_coverage/data/ERR3242021/HG01973.final.cram

**HG01977**	1000G_2504_high_coverage/data/ERR3242022/HG01977.final.cram
**HG01976**	1000G_2504_high_coverage/data/ERR3242023/HG01976.final.cram
**HG01979**	1000G_2504_high_coverage/data/ERR3242024/HG01979.final.cram
**HG01980**	1000G_2504_high_coverage/data/ERR3242025/HG01980.final.cram
**HG01991**	1000G_2504_high_coverage/data/ERR3242026/HG01991.final.cram
**HG01992**	1000G_2504_high_coverage/data/ERR3242027/HG01992.final.cram
**HG02008**	1000G_2504_high_coverage/data/ERR3242028/HG02008.final.cram
**HG01997**	1000G_2504_high_coverage/data/ERR3242029/HG01997.final.cram
**HG02002**	1000G_2504_high_coverage/data/ERR3242030/HG02002.final.cram
**HG02003**	1000G_2504_high_coverage/data/ERR3242031/HG02003.final.cram
**HG02090**	1000G_2504_high_coverage/data/ERR3242032/HG02090.final.cram
**HG02089**	1000G_2504_high_coverage/data/ERR3242033/HG02089.final.cram
**HG02104**	1000G_2504_high_coverage/data/ERR3242034/HG02104.final.cram
**HG02105**	1000G_2504_high_coverage/data/ERR3242035/HG02105.final.cram
**HG02017**	1000G_2504_high_coverage/data/ERR3242036/HG02017.final.cram
**HG02016**	1000G_2504_high_coverage/data/ERR3242037/HG02016.final.cram
**HG02020**	1000G_2504_high_coverage/data/ERR3242038/HG02020.final.cram
**HG02019**	1000G_2504_high_coverage/data/ERR3242039/HG02019.final.cram
**HG02023**	1000G_2504_high_coverage/data/ERR3242040/HG02023.final.cram
**HG02026**	1000G_2504_high_coverage/data/ERR3242041/HG02026.final.cram
**HG02025**	1000G_2504_high_coverage/data/ERR3242042/HG02025.final.cram
**HG02029**	1000G_2504_high_coverage/data/ERR3242043/HG02029.final.cram
**HG02028**	1000G_2504_high_coverage/data/ERR3242044/HG02028.final.cram
**HG02058**	1000G_2504_high_coverage/data/ERR3242045/HG02058.final.cram
**HG02057**	1000G_2504_high_coverage/data/ERR3242046/HG02057.final.cram
**HG02070**	1000G_2504_high_coverage/data/ERR3242047/HG02070.final.cram
**HG02069**	1000G_2504_high_coverage/data/ERR3242048/HG02069.final.cram
**HG02073**	1000G_2504_high_coverage/data/ERR3242049/HG02073.final.cram
**HG02072**	1000G_2504_high_coverage/data/ERR3242050/HG02072.final.cram
**HG02076**	1000G_2504_high_coverage/data/ERR3242051/HG02076.final.cram
**HG02075**	1000G_2504_high_coverage/data/ERR3242052/HG02075.final.cram
**HG02079**	1000G_2504_high_coverage/data/ERR3242053/HG02079.final.cram
**HG02078**	1000G_2504_high_coverage/data/ERR3242054/HG02078.final.cram
**HG02082**	1000G_2504_high_coverage/data/ERR3242055/HG02082.final.cram
**HG02081**	1000G_2504_high_coverage/data/ERR3242056/HG02081.final.cram
**HG02085**	1000G_2504_high_coverage/data/ERR3242057/HG02085.final.cram
**HG02084**	1000G_2504_high_coverage/data/ERR3242058/HG02084.final.cram
**HG02134**	1000G_2504_high_coverage/data/ERR3242059/HG02134.final.cram
**HG02133**	1000G_2504_high_coverage/data/ERR3242060/HG02133.final.cram
**HG01841**	1000G_2504_high_coverage/data/ERR3242061/HG01841.final.cram
**HG01595**	1000G_2504_high_coverage/data/ERR3242062/HG01595.final.cram
**HG01596**	1000G_2504_high_coverage/data/ERR3242063/HG01596.final.cram
**HG01597**	1000G_2504_high_coverage/data/ERR3242064/HG01597.final.cram
**HG01598**	1000G_2504_high_coverage/data/ERR3242065/HG01598.final.cram
**HG01599**	1000G_2504_high_coverage/data/ERR3242066/HG01599.final.cram
**HG01600**	1000G_2504_high_coverage/data/ERR3242067/HG01600.final.cram
**HG01840**	1000G_2504_high_coverage/data/ERR3242068/HG01840.final.cram
**HG01842**	1000G_2504_high_coverage/data/ERR3242069/HG01842.final.cram
**HG01843**	1000G_2504_high_coverage/data/ERR3242070/HG01843.final.cram
**HG01844**	1000G_2504_high_coverage/data/ERR3242071/HG01844.final.cram
**HG01845**	1000G_2504_high_coverage/data/ERR3242072/HG01845.final.cram
**HG01846**	1000G_2504_high_coverage/data/ERR3242073/HG01846.final.cram
**HG01847**	1000G_2504_high_coverage/data/ERR3242074/HG01847.final.cram
**HG01848**	1000G_2504_high_coverage/data/ERR3242075/HG01848.final.cram
**HG01849**	1000G_2504_high_coverage/data/ERR3242076/HG01849.final.cram
**HG01850**	1000G_2504_high_coverage/data/ERR3242077/HG01850.final.cram
**HG01851**	1000G_2504_high_coverage/data/ERR3242078/HG01851.final.cram
**HG01852**	1000G_2504_high_coverage/data/ERR3242079/HG01852.final.cram
**HG01853**	1000G_2504_high_coverage/data/ERR3242080/HG01853.final.cram
**HG01855**	1000G_2504_high_coverage/data/ERR3242081/HG01855.final.cram
**HG01857**	1000G_2504_high_coverage/data/ERR3242082/HG01857.final.cram
**HG01858**	1000G_2504_high_coverage/data/ERR3242083/HG01858.final.cram
**HG01859**	1000G_2504_high_coverage/data/ERR3242084/HG01859.final.cram
**HG01860**	1000G_2504_high_coverage/data/ERR3242085/HG01860.final.cram
**HG01861**	1000G_2504_high_coverage/data/ERR3242086/HG01861.final.cram

**HG01862**	1000G_2504_high_coverage/data/ERR3242087/HG01862.final.cram
**HG01863**	1000G_2504_high_coverage/data/ERR3242088/HG01863.final.cram
**HG01864**	1000G_2504_high_coverage/data/ERR3242089/HG01864.final.cram
**HG01865**	1000G_2504_high_coverage/data/ERR3242090/HG01865.final.cram
**HG01866**	1000G_2504_high_coverage/data/ERR3242091/HG01866.final.cram
**HG01867**	1000G_2504_high_coverage/data/ERR3242092/HG01867.final.cram
**HG01868**	1000G_2504_high_coverage/data/ERR3242093/HG01868.final.cram
**HG01869**	1000G_2504_high_coverage/data/ERR3242094/HG01869.final.cram
**HG01870**	1000G_2504_high_coverage/data/ERR3242095/HG01870.final.cram
**HG01871**	1000G_2504_high_coverage/data/ERR3242096/HG01871.final.cram
**HG01872**	1000G_2504_high_coverage/data/ERR3242097/HG01872.final.cram
**HG01873**	1000G_2504_high_coverage/data/ERR3242098/HG01873.final.cram
**HG01874**	1000G_2504_high_coverage/data/ERR3242099/HG01874.final.cram
**HG01878**	1000G_2504_high_coverage/data/ERR3242100/HG01878.final.cram
**HG02048**	1000G_2504_high_coverage/data/ERR3242101/HG02048.final.cram
**HG02049**	1000G_2504_high_coverage/data/ERR3242102/HG02049.final.cram
**HG02086**	1000G_2504_high_coverage/data/ERR3242103/HG02086.final.cram
**HG02087**	1000G_2504_high_coverage/data/ERR3242104/HG02087.final.cram
**HG02088**	1000G_2504_high_coverage/data/ERR3242105/HG02088.final.cram
**HG02138**	1000G_2504_high_coverage/data/ERR3242106/HG02138.final.cram
**HG02139**	1000G_2504_high_coverage/data/ERR3242107/HG02139.final.cram
**HG02140**	1000G_2504_high_coverage/data/ERR3242108/HG02140.final.cram
**HG02142**	1000G_2504_high_coverage/data/ERR3242109/HG02142.final.cram
**HG01694**	1000G_2504_high_coverage/data/ERR3242110/HG01694.final.cram
**HG01695**	1000G_2504_high_coverage/data/ERR3242111/HG01695.final.cram
**HG01709**	1000G_2504_high_coverage/data/ERR3242112/HG01709.final.cram
**HG01710**	1000G_2504_high_coverage/data/ERR3242113/HG01710.final.cram
**HG01747**	1000G_2504_high_coverage/data/ERR3242114/HG01747.final.cram
**HG01746**	1000G_2504_high_coverage/data/ERR3242115/HG01746.final.cram
**HG01756**	1000G_2504_high_coverage/data/ERR3242116/HG01756.final.cram
**HG01757**	1000G_2504_high_coverage/data/ERR3242117/HG01757.final.cram
**HG01761**	1000G_2504_high_coverage/data/ERR3242118/HG01761.final.cram
**HG01762**	1000G_2504_high_coverage/data/ERR3242119/HG01762.final.cram
**HG01771**	1000G_2504_high_coverage/data/ERR3242120/HG01771.final.cram
**HG01770**	1000G_2504_high_coverage/data/ERR3242121/HG01770.final.cram
**HG01775**	1000G_2504_high_coverage/data/ERR3242122/HG01775.final.cram
**HG01773**	1000G_2504_high_coverage/data/ERR3242123/HG01773.final.cram
**HG01777**	1000G_2504_high_coverage/data/ERR3242124/HG01777.final.cram
**HG01776**	1000G_2504_high_coverage/data/ERR3242125/HG01776.final.cram
**HG01781**	1000G_2504_high_coverage/data/ERR3242126/HG01781.final.cram
**HG01779**	1000G_2504_high_coverage/data/ERR3242127/HG01779.final.cram
**HG00759**	1000G_2504_high_coverage/data/ERR3242128/HG00759.final.cram
**HG00766**	1000G_2504_high_coverage/data/ERR3242129/HG00766.final.cram
**HG00844**	1000G_2504_high_coverage/data/ERR3242130/HG00844.final.cram
**HG00851**	1000G_2504_high_coverage/data/ERR3242131/HG00851.final.cram
**HG00864**	1000G_2504_high_coverage/data/ERR3242132/HG00864.final.cram
**HG00867**	1000G_2504_high_coverage/data/ERR3242133/HG00867.final.cram
**HG00879**	1000G_2504_high_coverage/data/ERR3242134/HG00879.final.cram
**HG00881**	1000G_2504_high_coverage/data/ERR3242135/HG00881.final.cram
**HG00956**	1000G_2504_high_coverage/data/ERR3242136/HG00956.final.cram
**HG00978**	1000G_2504_high_coverage/data/ERR3242137/HG00978.final.cram
**HG00982**	1000G_2504_high_coverage/data/ERR3242138/HG00982.final.cram
**HG01028**	1000G_2504_high_coverage/data/ERR3242139/HG01028.final.cram
**HG01029**	1000G_2504_high_coverage/data/ERR3242140/HG01029.final.cram
**HG01031**	1000G_2504_high_coverage/data/ERR3242141/HG01031.final.cram
**HG01046**	1000G_2504_high_coverage/data/ERR3242142/HG01046.final.cram
**HG01794**	1000G_2504_high_coverage/data/ERR3242143/HG01794.final.cram
**HG01795**	1000G_2504_high_coverage/data/ERR3242144/HG01795.final.cram
**HG01796**	1000G_2504_high_coverage/data/ERR3242145/HG01796.final.cram
**HG01797**	1000G_2504_high_coverage/data/ERR3242146/HG01797.final.cram
**HG01798**	1000G_2504_high_coverage/data/ERR3242147/HG01798.final.cram
**HG01799**	1000G_2504_high_coverage/data/ERR3242148/HG01799.final.cram
**HG01800**	1000G_2504_high_coverage/data/ERR3242149/HG01800.final.cram
**HG01801**	1000G_2504_high_coverage/data/ERR3242150/HG01801.final.cram
**HG01802**	1000G_2504_high_coverage/data/ERR3242151/HG01802.final.cram

**HG01804**	1000G_2504_high_coverage/data/ERR3242152/HG01804.final.cram
**HG01805**	1000G_2504_high_coverage/data/ERR3242153/HG01805.final.cram
**HG01806**	1000G_2504_high_coverage/data/ERR3242154/HG01806.final.cram
**HG01807**	1000G_2504_high_coverage/data/ERR3242155/HG01807.final.cram
**HG01808**	1000G_2504_high_coverage/data/ERR3242156/HG01808.final.cram
**HG01809**	1000G_2504_high_coverage/data/ERR3242157/HG01809.final.cram
**HG01810**	1000G_2504_high_coverage/data/ERR3242158/HG01810.final.cram
**HG01811**	1000G_2504_high_coverage/data/ERR3242159/HG01811.final.cram
**HG01812**	1000G_2504_high_coverage/data/ERR3242160/HG01812.final.cram
**HG01813**	1000G_2504_high_coverage/data/ERR3242161/HG01813.final.cram
**HG02151**	1000G_2504_high_coverage/data/ERR3242162/HG02151.final.cram
**HG01815**	1000G_2504_high_coverage/data/ERR3242163/HG01815.final.cram
**HG01816**	1000G_2504_high_coverage/data/ERR3242164/HG01816.final.cram
**HG01817**	1000G_2504_high_coverage/data/ERR3242165/HG01817.final.cram
**HG02152**	1000G_2504_high_coverage/data/ERR3242166/HG02152.final.cram
**HG02153**	1000G_2504_high_coverage/data/ERR3242167/HG02153.final.cram
**HG02154**	1000G_2504_high_coverage/data/ERR3242168/HG02154.final.cram
**HG02155**	1000G_2504_high_coverage/data/ERR3242169/HG02155.final.cram
**HG02156**	1000G_2504_high_coverage/data/ERR3242170/HG02156.final.cram
**HG02164**	1000G_2504_high_coverage/data/ERR3242171/HG02164.final.cram
**HG02165**	1000G_2504_high_coverage/data/ERR3242172/HG02165.final.cram
**HG02166**	1000G_2504_high_coverage/data/ERR3242173/HG02166.final.cram
**HG02178**	1000G_2504_high_coverage/data/ERR3242174/HG02178.final.cram
**HG02179**	1000G_2504_high_coverage/data/ERR3242175/HG02179.final.cram
**HG02180**	1000G_2504_high_coverage/data/ERR3242176/HG02180.final.cram
**HG02181**	1000G_2504_high_coverage/data/ERR3242177/HG02181.final.cram
**HG02182**	1000G_2504_high_coverage/data/ERR3242178/HG02182.final.cram
**HG02184**	1000G_2504_high_coverage/data/ERR3242179/HG02184.final.cram
**HG02185**	1000G_2504_high_coverage/data/ERR3242180/HG02185.final.cram
**HG02186**	1000G_2504_high_coverage/data/ERR3242181/HG02186.final.cram
**HG02187**	1000G_2504_high_coverage/data/ERR3242182/HG02187.final.cram
**HG02188**	1000G_2504_high_coverage/data/ERR3242183/HG02188.final.cram
**HG02190**	1000G_2504_high_coverage/data/ERR3242184/HG02190.final.cram
**HG01789**	1000G_2504_high_coverage/data/ERR3242185/HG01789.final.cram
**HG01790**	1000G_2504_high_coverage/data/ERR3242186/HG01790.final.cram
**HG01791**	1000G_2504_high_coverage/data/ERR3242187/HG01791.final.cram
**HG02215**	1000G_2504_high_coverage/data/ERR3242188/HG02215.final.cram
**HG01882**	1000G_2504_high_coverage/data/ERR3242189/HG01882.final.cram
**HG01883**	1000G_2504_high_coverage/data/ERR3242190/HG01883.final.cram
**HG01885**	1000G_2504_high_coverage/data/ERR3242191/HG01885.final.cram
**HG01956**	1000G_2504_high_coverage/data/ERR3242192/HG01956.final.cram
**HG02014**	1000G_2504_high_coverage/data/ERR3242193/HG02014.final.cram
**HG01886**	1000G_2504_high_coverage/data/ERR3242194/HG01886.final.cram
**HG01890**	1000G_2504_high_coverage/data/ERR3242195/HG01890.final.cram
**HG01889**	1000G_2504_high_coverage/data/ERR3242196/HG01889.final.cram
**HG02009**	1000G_2504_high_coverage/data/ERR3242197/HG02009.final.cram
**HG02010**	1000G_2504_high_coverage/data/ERR3242198/HG02010.final.cram
**HG02285**	1000G_2504_high_coverage/data/ERR3242199/HG02285.final.cram
**HG02286**	1000G_2504_high_coverage/data/ERR3242200/HG02286.final.cram
**HG02301**	1000G_2504_high_coverage/data/ERR3242201/HG02301.final.cram
**HG01783**	1000G_2504_high_coverage/data/ERR3242202/HG01783.final.cram
**HG01784**	1000G_2504_high_coverage/data/ERR3242203/HG01784.final.cram
**HG02221**	1000G_2504_high_coverage/data/ERR3242204/HG02221.final.cram
**HG02220**	1000G_2504_high_coverage/data/ERR3242205/HG02220.final.cram
**HG02236**	1000G_2504_high_coverage/data/ERR3242206/HG02236.final.cram
**HG02235**	1000G_2504_high_coverage/data/ERR3242207/HG02235.final.cram
**HG02113**	1000G_2504_high_coverage/data/ERR3242208/HG02113.final.cram
**HG02141**	1000G_2504_high_coverage/data/ERR3242209/HG02141.final.cram
**HG02122**	1000G_2504_high_coverage/data/ERR3242210/HG02122.final.cram
**HG02121**	1000G_2504_high_coverage/data/ERR3242211/HG02121.final.cram
**HG02128**	1000G_2504_high_coverage/data/ERR3242212/HG02128.final.cram
**HG02127**	1000G_2504_high_coverage/data/ERR3242213/HG02127.final.cram
**HG02373**	1000G_2504_high_coverage/data/ERR3242214/HG02373.final.cram
**HG02394**	1000G_2504_high_coverage/data/ERR3242215/HG02394.final.cram
**HG02396**	1000G_2504_high_coverage/data/ERR3242216/HG02396.final.cram

**HG02397**	1000G_2504_high_coverage/data/ERR3242217/HG02397.final.cram
**HG01914**	1000G_2504_high_coverage/data/ERR3242218/HG01914.final.cram
**HG01915**	1000G_2504_high_coverage/data/ERR3242219/HG01915.final.cram
**HG01986**	1000G_2504_high_coverage/data/ERR3242220/HG01986.final.cram
**HG01958**	1000G_2504_high_coverage/data/ERR3242221/HG01958.final.cram
**HG01990**	1000G_2504_high_coverage/data/ERR3242222/HG01990.final.cram
**HG02012**	1000G_2504_high_coverage/data/ERR3242223/HG02012.final.cram
**HG02053**	1000G_2504_high_coverage/data/ERR3242224/HG02053.final.cram
**HG02054**	1000G_2504_high_coverage/data/ERR3242225/HG02054.final.cram
**HG02255**	1000G_2504_high_coverage/data/ERR3242226/HG02255.final.cram
**HG02256**	1000G_2504_high_coverage/data/ERR3242227/HG02256.final.cram
**HG02314**	1000G_2504_high_coverage/data/ERR3242228/HG02314.final.cram
**HG02315**	1000G_2504_high_coverage/data/ERR3242229/HG02315.final.cram
**HG02281**	1000G_2504_high_coverage/data/ERR3242230/HG02281.final.cram
**HG02282**	1000G_2504_high_coverage/data/ERR3242231/HG02282.final.cram
**HG02317**	1000G_2504_high_coverage/data/ERR3242232/HG02317.final.cram
**HG02318**	1000G_2504_high_coverage/data/ERR3242233/HG02318.final.cram
**HG01912**	1000G_2504_high_coverage/data/ERR3242234/HG01912.final.cram
**HG01894**	1000G_2504_high_coverage/data/ERR3242235/HG01894.final.cram
**HG01982**	1000G_2504_high_coverage/data/ERR3242236/HG01982.final.cram
**HG02146**	1000G_2504_high_coverage/data/ERR3242237/HG02146.final.cram
**HG02147**	1000G_2504_high_coverage/data/ERR3242238/HG02147.final.cram
**HG02259**	1000G_2504_high_coverage/data/ERR3242239/HG02259.final.cram
**HG02260**	1000G_2504_high_coverage/data/ERR3242240/HG02260.final.cram
**HG02271**	1000G_2504_high_coverage/data/ERR3242241/HG02271.final.cram
**HG02272**	1000G_2504_high_coverage/data/ERR3242242/HG02272.final.cram
**HG02277**	1000G_2504_high_coverage/data/ERR3242243/HG02277.final.cram
**HG02278**	1000G_2504_high_coverage/data/ERR3242244/HG02278.final.cram
**HG02291**	1000G_2504_high_coverage/data/ERR3242245/HG02291.final.cram
**HG02292**	1000G_2504_high_coverage/data/ERR3242246/HG02292.final.cram
**HG02299**	1000G_2504_high_coverage/data/ERR3242247/HG02299.final.cram
**HG02298**	1000G_2504_high_coverage/data/ERR3242248/HG02298.final.cram
**HG01699**	1000G_2504_high_coverage/data/ERR3242249/HG01699.final.cram
**HG01697**	1000G_2504_high_coverage/data/ERR3242250/HG01697.final.cram
**HG02224**	1000G_2504_high_coverage/data/ERR3242251/HG02224.final.cram
**HG02223**	1000G_2504_high_coverage/data/ERR3242252/HG02223.final.cram
**HG02231**	1000G_2504_high_coverage/data/ERR3242253/HG02231.final.cram
**HG02230**	1000G_2504_high_coverage/data/ERR3242254/HG02230.final.cram
**HG02238**	1000G_2504_high_coverage/data/ERR3242255/HG02238.final.cram
**HG02239**	1000G_2504_high_coverage/data/ERR3242256/HG02239.final.cram
**HG02032**	1000G_2504_high_coverage/data/ERR3242257/HG02032.final.cram
**HG02031**	1000G_2504_high_coverage/data/ERR3242258/HG02031.final.cram
**HG02050**	1000G_2504_high_coverage/data/ERR3242259/HG02050.final.cram
**HG02250**	1000G_2504_high_coverage/data/ERR3242260/HG02250.final.cram
**HG02351**	1000G_2504_high_coverage/data/ERR3242261/HG02351.final.cram
**HG02353**	1000G_2504_high_coverage/data/ERR3242262/HG02353.final.cram
**HG02355**	1000G_2504_high_coverage/data/ERR3242263/HG02355.final.cram
**HG02356**	1000G_2504_high_coverage/data/ERR3242264/HG02356.final.cram
**HG02360**	1000G_2504_high_coverage/data/ERR3242265/HG02360.final.cram
**HG02364**	1000G_2504_high_coverage/data/ERR3242266/HG02364.final.cram
**HG02367**	1000G_2504_high_coverage/data/ERR3242267/HG02367.final.cram
**HG02371**	1000G_2504_high_coverage/data/ERR3242268/HG02371.final.cram
**HG02374**	1000G_2504_high_coverage/data/ERR3242269/HG02374.final.cram
**HG02375**	1000G_2504_high_coverage/data/ERR3242270/HG02375.final.cram
**HG02379**	1000G_2504_high_coverage/data/ERR3242271/HG02379.final.cram
**HG02380**	1000G_2504_high_coverage/data/ERR3242272/HG02380.final.cram
**HG02382**	1000G_2504_high_coverage/data/ERR3242273/HG02382.final.cram
**HG02383**	1000G_2504_high_coverage/data/ERR3242274/HG02383.final.cram
**HG02384**	1000G_2504_high_coverage/data/ERR3242275/HG02384.final.cram
**HG02385**	1000G_2504_high_coverage/data/ERR3242276/HG02385.final.cram
**HG02386**	1000G_2504_high_coverage/data/ERR3242277/HG02386.final.cram
**HG02389**	1000G_2504_high_coverage/data/ERR3242278/HG02389.final.cram
**HG02390**	1000G_2504_high_coverage/data/ERR3242279/HG02390.final.cram
**HG02391**	1000G_2504_high_coverage/data/ERR3242280/HG02391.final.cram
**HG02392**	1000G_2504_high_coverage/data/ERR3242281/HG02392.final.cram

**HG02395**	1000G_2504_high_coverage/data/ERR3242282/HG02395.final.cram
**HG02398**	1000G_2504_high_coverage/data/ERR3242283/HG02398.final.cram
**HG02399**	1000G_2504_high_coverage/data/ERR3242284/HG02399.final.cram
**HG02401**	1000G_2504_high_coverage/data/ERR3242285/HG02401.final.cram
**HG02402**	1000G_2504_high_coverage/data/ERR3242286/HG02402.final.cram
**HG02406**	1000G_2504_high_coverage/data/ERR3242287/HG02406.final.cram
**HG02407**	1000G_2504_high_coverage/data/ERR3242288/HG02407.final.cram
**HG02409**	1000G_2504_high_coverage/data/ERR3242289/HG02409.final.cram
**HG02233**	1000G_2504_high_coverage/data/ERR3242290/HG02233.final.cram
**HG02232**	1000G_2504_high_coverage/data/ERR3242291/HG02232.final.cram
**HG01879**	1000G_2504_high_coverage/data/ERR3242292/HG01879.final.cram
**HG01880**	1000G_2504_high_coverage/data/ERR3242293/HG01880.final.cram
**HG02013**	1000G_2504_high_coverage/data/ERR3242294/HG02013.final.cram
**HG01896**	1000G_2504_high_coverage/data/ERR3242295/HG01896.final.cram
**HG01985**	1000G_2504_high_coverage/data/ERR3242296/HG01985.final.cram
**HG01988**	1000G_2504_high_coverage/data/ERR3242297/HG01988.final.cram
**HG01989**	1000G_2504_high_coverage/data/ERR3242298/HG01989.final.cram
**HG02051**	1000G_2504_high_coverage/data/ERR3242299/HG02051.final.cram
**HG02052**	1000G_2504_high_coverage/data/ERR3242300/HG02052.final.cram
**HG02095**	1000G_2504_high_coverage/data/ERR3242301/HG02095.final.cram
**HG02332**	1000G_2504_high_coverage/data/ERR3242302/HG02332.final.cram
**HG02107**	1000G_2504_high_coverage/data/ERR3242303/HG02107.final.cram
**HG02108**	1000G_2504_high_coverage/data/ERR3242304/HG02108.final.cram
**HG02111**	1000G_2504_high_coverage/data/ERR3242305/HG02111.final.cram
**HG02143**	1000G_2504_high_coverage/data/ERR3242306/HG02143.final.cram
**HG02144**	1000G_2504_high_coverage/data/ERR3242307/HG02144.final.cram
**HG02307**	1000G_2504_high_coverage/data/ERR3242308/HG02307.final.cram
**HG02308**	1000G_2504_high_coverage/data/ERR3242309/HG02308.final.cram
**HG02283**	1000G_2504_high_coverage/data/ERR3242310/HG02283.final.cram
**HG02309**	1000G_2504_high_coverage/data/ERR3242311/HG02309.final.cram
**HG02284**	1000G_2504_high_coverage/data/ERR3242312/HG02284.final.cram
**HG02322**	1000G_2504_high_coverage/data/ERR3242313/HG02322.final.cram
**HG02323**	1000G_2504_high_coverage/data/ERR3242314/HG02323.final.cram
**HG02325**	1000G_2504_high_coverage/data/ERR3242315/HG02325.final.cram
**HG02337**	1000G_2504_high_coverage/data/ERR3242316/HG02337.final.cram
**HG02334**	1000G_2504_high_coverage/data/ERR3242317/HG02334.final.cram
**HG02330**	1000G_2504_high_coverage/data/ERR3242318/HG02330.final.cram
**HG02339**	1000G_2504_high_coverage/data/ERR3242319/HG02339.final.cram
**HG02343**	1000G_2504_high_coverage/data/ERR3242320/HG02343.final.cram
**HG02419**	1000G_2504_high_coverage/data/ERR3242321/HG02419.final.cram
**HG02420**	1000G_2504_high_coverage/data/ERR3242322/HG02420.final.cram
**HG02427**	1000G_2504_high_coverage/data/ERR3242323/HG02427.final.cram
**HG02429**	1000G_2504_high_coverage/data/ERR3242324/HG02429.final.cram
**HG02433**	1000G_2504_high_coverage/data/ERR3242325/HG02433.final.cram
**HG02442**	1000G_2504_high_coverage/data/ERR3242326/HG02442.final.cram
**HG02445**	1000G_2504_high_coverage/data/ERR3242327/HG02445.final.cram
**HG02489**	1000G_2504_high_coverage/data/ERR3242328/HG02489.final.cram
**HG02449**	1000G_2504_high_coverage/data/ERR3242329/HG02449.final.cram
**HG02450**	1000G_2504_high_coverage/data/ERR3242330/HG02450.final.cram
**HG02470**	1000G_2504_high_coverage/data/ERR3242331/HG02470.final.cram
**HG02471**	1000G_2504_high_coverage/data/ERR3242332/HG02471.final.cram
**HG02479**	1000G_2504_high_coverage/data/ERR3242333/HG02479.final.cram
**HG02484**	1000G_2504_high_coverage/data/ERR3242334/HG02484.final.cram
**HG02485**	1000G_2504_high_coverage/data/ERR3242335/HG02485.final.cram
**HG02511**	1000G_2504_high_coverage/data/ERR3242336/HG02511.final.cram
**HG02496**	1000G_2504_high_coverage/data/ERR3242337/HG02496.final.cram
**HG02497**	1000G_2504_high_coverage/data/ERR3242338/HG02497.final.cram
**HG02508**	1000G_2504_high_coverage/data/ERR3242339/HG02508.final.cram
**HG02537**	1000G_2504_high_coverage/data/ERR3242340/HG02537.final.cram
**HG02035**	1000G_2504_high_coverage/data/ERR3242341/HG02035.final.cram
**HG02040**	1000G_2504_high_coverage/data/ERR3242342/HG02040.final.cram
**HG02047**	1000G_2504_high_coverage/data/ERR3242343/HG02047.final.cram
**HG02061**	1000G_2504_high_coverage/data/ERR3242344/HG02061.final.cram
**HG02060**	1000G_2504_high_coverage/data/ERR3242345/HG02060.final.cram
**HG02064**	1000G_2504_high_coverage/data/ERR3242346/HG02064.final.cram

**HG02067**	1000G_2504_high_coverage/data/ERR3242347/HG02067.final.cram
**HG02116**	1000G_2504_high_coverage/data/ERR3242348/HG02116.final.cram
**HG02131**	1000G_2504_high_coverage/data/ERR3242349/HG02131.final.cram
**HG02130**	1000G_2504_high_coverage/data/ERR3242350/HG02130.final.cram
**HG02137**	1000G_2504_high_coverage/data/ERR3242351/HG02137.final.cram
**HG02136**	1000G_2504_high_coverage/data/ERR3242352/HG02136.final.cram
**HG02512**	1000G_2504_high_coverage/data/ERR3242353/HG02512.final.cram
**HG02513**	1000G_2504_high_coverage/data/ERR3242354/HG02513.final.cram
**HG02521**	1000G_2504_high_coverage/data/ERR3242355/HG02521.final.cram
**HG02522**	1000G_2504_high_coverage/data/ERR3242356/HG02522.final.cram
**HG02461**	1000G_2504_high_coverage/data/ERR3242357/HG02461.final.cram
**HG02462**	1000G_2504_high_coverage/data/ERR3242358/HG02462.final.cram
**HG02464**	1000G_2504_high_coverage/data/ERR3242359/HG02464.final.cram
**HG02465**	1000G_2504_high_coverage/data/ERR3242360/HG02465.final.cram
**HG02561**	1000G_2504_high_coverage/data/ERR3242361/HG02561.final.cram
**HG02562**	1000G_2504_high_coverage/data/ERR3242362/HG02562.final.cram
**HG02570**	1000G_2504_high_coverage/data/ERR3242363/HG02570.final.cram
**HG02571**	1000G_2504_high_coverage/data/ERR3242364/HG02571.final.cram
**HG02573**	1000G_2504_high_coverage/data/ERR3242365/HG02573.final.cram
**HG02574**	1000G_2504_high_coverage/data/ERR3242366/HG02574.final.cram
**HG02582**	1000G_2504_high_coverage/data/ERR3242367/HG02582.final.cram
**HG02583**	1000G_2504_high_coverage/data/ERR3242368/HG02583.final.cram
**HG02585**	1000G_2504_high_coverage/data/ERR3242369/HG02585.final.cram
**HG02586**	1000G_2504_high_coverage/data/ERR3242370/HG02586.final.cram
**HG02588**	1000G_2504_high_coverage/data/ERR3242371/HG02588.final.cram
**HG02589**	1000G_2504_high_coverage/data/ERR3242372/HG02589.final.cram
**HG02594**	1000G_2504_high_coverage/data/ERR3242373/HG02594.final.cram
**HG02595**	1000G_2504_high_coverage/data/ERR3242374/HG02595.final.cram
**HG02613**	1000G_2504_high_coverage/data/ERR3242375/HG02613.final.cram
**HG02614**	1000G_2504_high_coverage/data/ERR3242376/HG02614.final.cram
**HG02620**	1000G_2504_high_coverage/data/ERR3242377/HG02620.final.cram
**HG02621**	1000G_2504_high_coverage/data/ERR3242378/HG02621.final.cram
**HG02623**	1000G_2504_high_coverage/data/ERR3242379/HG02623.final.cram
**HG02624**	1000G_2504_high_coverage/data/ERR3242380/HG02624.final.cram
**HG02628**	1000G_2504_high_coverage/data/ERR3242381/HG02628.final.cram
**HG02629**	1000G_2504_high_coverage/data/ERR3242382/HG02629.final.cram
**HG02642**	1000G_2504_high_coverage/data/ERR3242383/HG02642.final.cram
**HG02643**	1000G_2504_high_coverage/data/ERR3242384/HG02643.final.cram
**HG02645**	1000G_2504_high_coverage/data/ERR3242385/HG02645.final.cram
**HG02646**	1000G_2504_high_coverage/data/ERR3242386/HG02646.final.cram
**HG02666**	1000G_2504_high_coverage/data/ERR3242387/HG02666.final.cram
**HG02667**	1000G_2504_high_coverage/data/ERR3242388/HG02667.final.cram
**HG02675**	1000G_2504_high_coverage/data/ERR3242389/HG02675.final.cram
**HG02676**	1000G_2504_high_coverage/data/ERR3242390/HG02676.final.cram
**HG02678**	1000G_2504_high_coverage/data/ERR3242391/HG02678.final.cram
**HG02679**	1000G_2504_high_coverage/data/ERR3242392/HG02679.final.cram
**HG02702**	1000G_2504_high_coverage/data/ERR3242393/HG02702.final.cram
**HG02703**	1000G_2504_high_coverage/data/ERR3242394/HG02703.final.cram
**HG02721**	1000G_2504_high_coverage/data/ERR3242395/HG02721.final.cram
**HG02722**	1000G_2504_high_coverage/data/ERR3242396/HG02722.final.cram
**HG02490**	1000G_2504_high_coverage/data/ERR3242397/HG02490.final.cram
**HG02491**	1000G_2504_high_coverage/data/ERR3242398/HG02491.final.cram
**HG02600**	1000G_2504_high_coverage/data/ERR3242399/HG02600.final.cram
**HG02601**	1000G_2504_high_coverage/data/ERR3242400/HG02601.final.cram
**HG02603**	1000G_2504_high_coverage/data/ERR3242401/HG02603.final.cram
**HG02604**	1000G_2504_high_coverage/data/ERR3242402/HG02604.final.cram
**HG02654**	1000G_2504_high_coverage/data/ERR3242403/HG02654.final.cram
**HG02655**	1000G_2504_high_coverage/data/ERR3242404/HG02655.final.cram
**HG02657**	1000G_2504_high_coverage/data/ERR3242405/HG02657.final.cram
**HG02658**	1000G_2504_high_coverage/data/ERR3242406/HG02658.final.cram
**HG02660**	1000G_2504_high_coverage/data/ERR3242407/HG02660.final.cram
**HG02661**	1000G_2504_high_coverage/data/ERR3242408/HG02661.final.cram
**HG02684**	1000G_2504_high_coverage/data/ERR3242409/HG02684.final.cram
**HG02685**	1000G_2504_high_coverage/data/ERR3242410/HG02685.final.cram
**HG02687**	1000G_2504_high_coverage/data/ERR3242411/HG02687.final.cram

**HG02688**	1000G_2504_high_coverage/data/ERR3242412/HG02688.final.cram
**HG02696**	1000G_2504_high_coverage/data/ERR3242413/HG02696.final.cram
**HG02697**	1000G_2504_high_coverage/data/ERR3242414/HG02697.final.cram
**HG02733**	1000G_2504_high_coverage/data/ERR3242415/HG02733.final.cram
**HG02734**	1000G_2504_high_coverage/data/ERR3242416/HG02734.final.cram
**HG02610**	1000G_2504_high_coverage/data/ERR3242417/HG02610.final.cram
**HG02611**	1000G_2504_high_coverage/data/ERR3242418/HG02611.final.cram
**HG02634**	1000G_2504_high_coverage/data/ERR3242419/HG02634.final.cram
**HG02635**	1000G_2504_high_coverage/data/ERR3242420/HG02635.final.cram
**HG02715**	1000G_2504_high_coverage/data/ERR3242421/HG02715.final.cram
**HG02716**	1000G_2504_high_coverage/data/ERR3242422/HG02716.final.cram
**HG02768**	1000G_2504_high_coverage/data/ERR3242423/HG02768.final.cram
**HG02769**	1000G_2504_high_coverage/data/ERR3242424/HG02769.final.cram
**HG02771**	1000G_2504_high_coverage/data/ERR3242425/HG02771.final.cram
**HG02772**	1000G_2504_high_coverage/data/ERR3242426/HG02772.final.cram
**HG02798**	1000G_2504_high_coverage/data/ERR3242427/HG02798.final.cram
**HG02799**	1000G_2504_high_coverage/data/ERR3242428/HG02799.final.cram
**HG02804**	1000G_2504_high_coverage/data/ERR3242429/HG02804.final.cram
**HG02805**	1000G_2504_high_coverage/data/ERR3242430/HG02805.final.cram
**HG02807**	1000G_2504_high_coverage/data/ERR3242431/HG02807.final.cram
**HG02808**	1000G_2504_high_coverage/data/ERR3242432/HG02808.final.cram
**HG02810**	1000G_2504_high_coverage/data/ERR3242433/HG02810.final.cram
**HG02811**	1000G_2504_high_coverage/data/ERR3242434/HG02811.final.cram
**HG02813**	1000G_2504_high_coverage/data/ERR3242435/HG02813.final.cram
**HG02814**	1000G_2504_high_coverage/data/ERR3242436/HG02814.final.cram
**HG02839**	1000G_2504_high_coverage/data/ERR3242437/HG02839.final.cram
**HG02840**	1000G_2504_high_coverage/data/ERR3242438/HG02840.final.cram
**HG02851**	1000G_2504_high_coverage/data/ERR3242439/HG02851.final.cram
**HG02852**	1000G_2504_high_coverage/data/ERR3242440/HG02852.final.cram
**HG02887**	1000G_2504_high_coverage/data/ERR3242441/HG02887.final.cram
**HG02888**	1000G_2504_high_coverage/data/ERR3242442/HG02888.final.cram
**HG02895**	1000G_2504_high_coverage/data/ERR3242443/HG02895.final.cram
**HG02896**	1000G_2504_high_coverage/data/ERR3242444/HG02896.final.cram
**HG03027**	1000G_2504_high_coverage/data/ERR3242445/HG03027.final.cram
**HG03028**	1000G_2504_high_coverage/data/ERR3242446/HG03028.final.cram
**HG03039**	1000G_2504_high_coverage/data/ERR3242447/HG03039.final.cram
**HG03040**	1000G_2504_high_coverage/data/ERR3242448/HG03040.final.cram
**HG03045**	1000G_2504_high_coverage/data/ERR3242449/HG03045.final.cram
**HG03046**	1000G_2504_high_coverage/data/ERR3242450/HG03046.final.cram
**HG03048**	1000G_2504_high_coverage/data/ERR3242451/HG03048.final.cram
**HG03049**	1000G_2504_high_coverage/data/ERR3242452/HG03049.final.cram
**HG03258**	1000G_2504_high_coverage/data/ERR3242453/HG03258.final.cram
**HG03259**	1000G_2504_high_coverage/data/ERR3242454/HG03259.final.cram
**HG02756**	1000G_2504_high_coverage/data/ERR3242455/HG02756.final.cram
**HG02757**	1000G_2504_high_coverage/data/ERR3242456/HG02757.final.cram
**HG02816**	1000G_2504_high_coverage/data/ERR3242457/HG02816.final.cram
**HG02817**	1000G_2504_high_coverage/data/ERR3242458/HG02817.final.cram
**HG02836**	1000G_2504_high_coverage/data/ERR3242459/HG02836.final.cram
**HG02837**	1000G_2504_high_coverage/data/ERR3242460/HG02837.final.cram
**HG02860**	1000G_2504_high_coverage/data/ERR3242461/HG02860.final.cram
**HG02861**	1000G_2504_high_coverage/data/ERR3242462/HG02861.final.cram
**HG02884**	1000G_2504_high_coverage/data/ERR3242463/HG02884.final.cram
**HG02885**	1000G_2504_high_coverage/data/ERR3242464/HG02885.final.cram
**HG02890**	1000G_2504_high_coverage/data/ERR3242465/HG02890.final.cram
**HG02891**	1000G_2504_high_coverage/data/ERR3242466/HG02891.final.cram
**HG03052**	1000G_2504_high_coverage/data/ERR3242467/HG03052.final.cram
**HG03057**	1000G_2504_high_coverage/data/ERR3242468/HG03057.final.cram
**HG03058**	1000G_2504_high_coverage/data/ERR3242469/HG03058.final.cram
**HG03078**	1000G_2504_high_coverage/data/ERR3242470/HG03078.final.cram
**HG03084**	1000G_2504_high_coverage/data/ERR3242471/HG03084.final.cram
**HG03085**	1000G_2504_high_coverage/data/ERR3242472/HG03085.final.cram
**HG03096**	1000G_2504_high_coverage/data/ERR3242473/HG03096.final.cram
**HG03097**	1000G_2504_high_coverage/data/ERR3242474/HG03097.final.cram
**HG03397**	1000G_2504_high_coverage/data/ERR3242475/HG03397.final.cram
**HG03451**	1000G_2504_high_coverage/data/ERR3242476/HG03451.final.cram

**HG03452**	1000G_2504_high_coverage/data/ERR3242477/HG03452.final.cram
**HG03464**	1000G_2504_high_coverage/data/ERR3242478/HG03464.final.cram
**HG03469**	1000G_2504_high_coverage/data/ERR3242479/HG03469.final.cram
**HG03472**	1000G_2504_high_coverage/data/ERR3242480/HG03472.final.cram
**HG02923**	1000G_2504_high_coverage/data/ERR3242481/HG02923.final.cram
**HG02922**	1000G_2504_high_coverage/data/ERR3242482/HG02922.final.cram
**HG02944**	1000G_2504_high_coverage/data/ERR3242483/HG02944.final.cram
**HG02943**	1000G_2504_high_coverage/data/ERR3242484/HG02943.final.cram
**HG02974**	1000G_2504_high_coverage/data/ERR3242485/HG02974.final.cram
**HG02973**	1000G_2504_high_coverage/data/ERR3242486/HG02973.final.cram
**HG03100**	1000G_2504_high_coverage/data/ERR3242487/HG03100.final.cram
**HG03099**	1000G_2504_high_coverage/data/ERR3242488/HG03099.final.cram
**HG03109**	1000G_2504_high_coverage/data/ERR3242489/HG03109.final.cram
**HG03108**	1000G_2504_high_coverage/data/ERR3242490/HG03108.final.cram
**HG03115**	1000G_2504_high_coverage/data/ERR3242491/HG03115.final.cram
**HG03114**	1000G_2504_high_coverage/data/ERR3242492/HG03114.final.cram
**HG03121**	1000G_2504_high_coverage/data/ERR3242493/HG03121.final.cram
**HG03120**	1000G_2504_high_coverage/data/ERR3242494/HG03120.final.cram
**HG03124**	1000G_2504_high_coverage/data/ERR3242495/HG03124.final.cram
**HG03123**	1000G_2504_high_coverage/data/ERR3242496/HG03123.final.cram
**HG03133**	1000G_2504_high_coverage/data/ERR3242497/HG03133.final.cram
**HG03132**	1000G_2504_high_coverage/data/ERR3242498/HG03132.final.cram
**HG03136**	1000G_2504_high_coverage/data/ERR3242499/HG03136.final.cram
**HG03135**	1000G_2504_high_coverage/data/ERR3242500/HG03135.final.cram
**HG02724**	1000G_2504_high_coverage/data/ERR3242501/HG02724.final.cram
**HG02725**	1000G_2504_high_coverage/data/ERR3242502/HG02725.final.cram
**HG02727**	1000G_2504_high_coverage/data/ERR3242503/HG02727.final.cram
**HG02728**	1000G_2504_high_coverage/data/ERR3242504/HG02728.final.cram
**HG02783**	1000G_2504_high_coverage/data/ERR3242505/HG02783.final.cram
**HG02784**	1000G_2504_high_coverage/data/ERR3242506/HG02784.final.cram
**HG02786**	1000G_2504_high_coverage/data/ERR3242507/HG02786.final.cram
**HG02787**	1000G_2504_high_coverage/data/ERR3242508/HG02787.final.cram
**HG02789**	1000G_2504_high_coverage/data/ERR3242509/HG02789.final.cram
**HG02790**	1000G_2504_high_coverage/data/ERR3242510/HG02790.final.cram
**HG00409**	1000G_2504_high_coverage/data/ERR3242511/HG00409.final.cram
**HG00410**	1000G_2504_high_coverage/data/ERR3242512/HG00410.final.cram
**HG00598**	1000G_2504_high_coverage/data/ERR3242513/HG00598.final.cram
**HG00599**	1000G_2504_high_coverage/data/ERR3242514/HG00599.final.cram
**HG00728**	1000G_2504_high_coverage/data/ERR3242515/HG00728.final.cram
**HG00729**	1000G_2504_high_coverage/data/ERR3242516/HG00729.final.cram
**HG00622**	1000G_2504_high_coverage/data/ERR3242517/HG00622.final.cram
**HG00623**	1000G_2504_high_coverage/data/ERR3242518/HG00623.final.cram
**HG00631**	1000G_2504_high_coverage/data/ERR3242519/HG00631.final.cram
**HG00632**	1000G_2504_high_coverage/data/ERR3242520/HG00632.final.cram
**HG00674**	1000G_2504_high_coverage/data/ERR3242521/HG00674.final.cram
**HG00675**	1000G_2504_high_coverage/data/ERR3242522/HG00675.final.cram
**HG03024**	1000G_2504_high_coverage/data/ERR3242523/HG03024.final.cram
**HG03025**	1000G_2504_high_coverage/data/ERR3242524/HG03025.final.cram
**HG03240**	1000G_2504_high_coverage/data/ERR3242525/HG03240.final.cram
**HG03241**	1000G_2504_high_coverage/data/ERR3242526/HG03241.final.cram
**HG03246**	1000G_2504_high_coverage/data/ERR3242527/HG03246.final.cram
**HG03247**	1000G_2504_high_coverage/data/ERR3242528/HG03247.final.cram
**HG03538**	1000G_2504_high_coverage/data/ERR3242529/HG03538.final.cram
**HG03539**	1000G_2504_high_coverage/data/ERR3242530/HG03539.final.cram
**HG02819**	1000G_2504_high_coverage/data/ERR3242531/HG02819.final.cram
**HG02820**	1000G_2504_high_coverage/data/ERR3242532/HG02820.final.cram
**HG02854**	1000G_2504_high_coverage/data/ERR3242533/HG02854.final.cram
**HG02855**	1000G_2504_high_coverage/data/ERR3242534/HG02855.final.cram
**HG03470**	1000G_2504_high_coverage/data/ERR3242535/HG03470.final.cram
**HG03473**	1000G_2504_high_coverage/data/ERR3242536/HG03473.final.cram
**HG03081**	1000G_2504_high_coverage/data/ERR3242537/HG03081.final.cram
**HG03088**	1000G_2504_high_coverage/data/ERR3242538/HG03088.final.cram
**HG03209**	1000G_2504_high_coverage/data/ERR3242539/HG03209.final.cram
**HG03212**	1000G_2504_high_coverage/data/ERR3242540/HG03212.final.cram
**HG03055**	1000G_2504_high_coverage/data/ERR3242541/HG03055.final.cram

**HG03376**	1000G_2504_high_coverage/data/ERR3242542/HG03376.final.cram
**HG03380**	1000G_2504_high_coverage/data/ERR3242543/HG03380.final.cram
**HG03385**	1000G_2504_high_coverage/data/ERR3242544/HG03385.final.cram
**HG03388**	1000G_2504_high_coverage/data/ERR3242545/HG03388.final.cram
**HG03401**	1000G_2504_high_coverage/data/ERR3242546/HG03401.final.cram
**HG03419**	1000G_2504_high_coverage/data/ERR3242547/HG03419.final.cram
**HG03061**	1000G_2504_high_coverage/data/ERR3242548/HG03061.final.cram
**HG03428**	1000G_2504_high_coverage/data/ERR3242549/HG03428.final.cram
**HG03433**	1000G_2504_high_coverage/data/ERR3242550/HG03433.final.cram
**HG03439**	1000G_2504_high_coverage/data/ERR3242551/HG03439.final.cram
**HG03442**	1000G_2504_high_coverage/data/ERR3242552/HG03442.final.cram
**HG03445**	1000G_2504_high_coverage/data/ERR3242553/HG03445.final.cram
**HG03446**	1000G_2504_high_coverage/data/ERR3242554/HG03446.final.cram
**HG03460**	1000G_2504_high_coverage/data/ERR3242555/HG03460.final.cram
**HG03461**	1000G_2504_high_coverage/data/ERR3242556/HG03461.final.cram
**HG03478**	1000G_2504_high_coverage/data/ERR3242557/HG03478.final.cram
**HG03479**	1000G_2504_high_coverage/data/ERR3242558/HG03479.final.cram
**HG03066**	1000G_2504_high_coverage/data/ERR3242559/HG03066.final.cram
**HG03069**	1000G_2504_high_coverage/data/ERR3242560/HG03069.final.cram
**HG03072**	1000G_2504_high_coverage/data/ERR3242561/HG03072.final.cram
**HG03410**	1000G_2504_high_coverage/data/ERR3242562/HG03410.final.cram
**HG03436**	1000G_2504_high_coverage/data/ERR3242563/HG03436.final.cram
**HG03437**	1000G_2504_high_coverage/data/ERR3242564/HG03437.final.cram
**HG03449**	1000G_2504_high_coverage/data/ERR3242565/HG03449.final.cram
**HG03458**	1000G_2504_high_coverage/data/ERR3242566/HG03458.final.cram
**HG03476**	1000G_2504_high_coverage/data/ERR3242567/HG03476.final.cram
**HG03484**	1000G_2504_high_coverage/data/ERR3242568/HG03484.final.cram
**HG03485**	1000G_2504_high_coverage/data/ERR3242569/HG03485.final.cram
**HG03547**	1000G_2504_high_coverage/data/ERR3242570/HG03547.final.cram
**HG03548**	1000G_2504_high_coverage/data/ERR3242571/HG03548.final.cram
**HG03556**	1000G_2504_high_coverage/data/ERR3242572/HG03556.final.cram
**HG03557**	1000G_2504_high_coverage/data/ERR3242573/HG03557.final.cram
**HG03391**	1000G_2504_high_coverage/data/ERR3242574/HG03391.final.cram
**HG03455**	1000G_2504_high_coverage/data/ERR3242575/HG03455.final.cram
**HG03559**	1000G_2504_high_coverage/data/ERR3242576/HG03559.final.cram
**HG03563**	1000G_2504_high_coverage/data/ERR3242577/HG03563.final.cram
**HG03565**	1000G_2504_high_coverage/data/ERR3242578/HG03565.final.cram
**HG03571**	1000G_2504_high_coverage/data/ERR3242579/HG03571.final.cram
**HG03572**	1000G_2504_high_coverage/data/ERR3242580/HG03572.final.cram
**HG03575**	1000G_2504_high_coverage/data/ERR3242581/HG03575.final.cram
**HG03577**	1000G_2504_high_coverage/data/ERR3242582/HG03577.final.cram
**HG03578**	1000G_2504_high_coverage/data/ERR3242583/HG03578.final.cram
**HG03583**	1000G_2504_high_coverage/data/ERR3242584/HG03583.final.cram
**HG03515**	1000G_2504_high_coverage/data/ERR3242585/HG03515.final.cram
**HG03514**	1000G_2504_high_coverage/data/ERR3242586/HG03514.final.cram
**HG03268**	1000G_2504_high_coverage/data/ERR3242587/HG03268.final.cram
**HG03267**	1000G_2504_high_coverage/data/ERR3242588/HG03267.final.cram
**HG03271**	1000G_2504_high_coverage/data/ERR3242589/HG03271.final.cram
**HG03270**	1000G_2504_high_coverage/data/ERR3242590/HG03270.final.cram
**HG03280**	1000G_2504_high_coverage/data/ERR3242591/HG03280.final.cram
**HG03279**	1000G_2504_high_coverage/data/ERR3242592/HG03279.final.cram
**HG03291**	1000G_2504_high_coverage/data/ERR3242593/HG03291.final.cram
**HG03295**	1000G_2504_high_coverage/data/ERR3242594/HG03295.final.cram
**HG03294**	1000G_2504_high_coverage/data/ERR3242595/HG03294.final.cram
**HG03301**	1000G_2504_high_coverage/data/ERR3242596/HG03301.final.cram
**HG03300**	1000G_2504_high_coverage/data/ERR3242597/HG03300.final.cram
**HG03367**	1000G_2504_high_coverage/data/ERR3242598/HG03367.final.cram
**HG03366**	1000G_2504_high_coverage/data/ERR3242599/HG03366.final.cram
**HG02947**	1000G_2504_high_coverage/data/ERR3242600/HG02947.final.cram
**HG02946**	1000G_2504_high_coverage/data/ERR3242601/HG02946.final.cram
**HG02971**	1000G_2504_high_coverage/data/ERR3242602/HG02971.final.cram
**HG02970**	1000G_2504_high_coverage/data/ERR3242603/HG02970.final.cram
**HG02953**	1000G_2504_high_coverage/data/ERR3242604/HG02953.final.cram
**HG02952**	1000G_2504_high_coverage/data/ERR3242605/HG02952.final.cram
**HG02977**	1000G_2504_high_coverage/data/ERR3242606/HG02977.final.cram

**HG02976**	1000G_2504_high_coverage/data/ERR3242607/HG02976.final.cram
**HG03118**	1000G_2504_high_coverage/data/ERR3242608/HG03118.final.cram
**HG03117**	1000G_2504_high_coverage/data/ERR3242609/HG03117.final.cram
**HG03130**	1000G_2504_high_coverage/data/ERR3242610/HG03130.final.cram
**HG03129**	1000G_2504_high_coverage/data/ERR3242611/HG03129.final.cram
**HG03160**	1000G_2504_high_coverage/data/ERR3242612/HG03160.final.cram
**HG03159**	1000G_2504_high_coverage/data/ERR3242613/HG03159.final.cram
**HG03163**	1000G_2504_high_coverage/data/ERR3242614/HG03163.final.cram
**HG03162**	1000G_2504_high_coverage/data/ERR3242615/HG03162.final.cram
**HG03169**	1000G_2504_high_coverage/data/ERR3242616/HG03169.final.cram
**HG03168**	1000G_2504_high_coverage/data/ERR3242617/HG03168.final.cram
**HG03172**	1000G_2504_high_coverage/data/ERR3242618/HG03172.final.cram
**HG03196**	1000G_2504_high_coverage/data/ERR3242619/HG03196.final.cram
**HG03195**	1000G_2504_high_coverage/data/ERR3242620/HG03195.final.cram
**HG03199**	1000G_2504_high_coverage/data/ERR3242621/HG03199.final.cram
**HG03198**	1000G_2504_high_coverage/data/ERR3242622/HG03198.final.cram
**HG03343**	1000G_2504_high_coverage/data/ERR3242623/HG03343.final.cram
**HG03342**	1000G_2504_high_coverage/data/ERR3242624/HG03342.final.cram
**HG03370**	1000G_2504_high_coverage/data/ERR3242625/HG03370.final.cram
**HG03369**	1000G_2504_high_coverage/data/ERR3242626/HG03369.final.cram
**HG03234**	1000G_2504_high_coverage/data/ERR3242627/HG03234.final.cram
**HG03235**	1000G_2504_high_coverage/data/ERR3242628/HG03235.final.cram
**HG03488**	1000G_2504_high_coverage/data/ERR3242629/HG03488.final.cram
**HG03490**	1000G_2504_high_coverage/data/ERR3242630/HG03490.final.cram
**HG03491**	1000G_2504_high_coverage/data/ERR3242631/HG03491.final.cram
**HG03619**	1000G_2504_high_coverage/data/ERR3242632/HG03619.final.cram
**HG03634**	1000G_2504_high_coverage/data/ERR3242633/HG03634.final.cram
**HG03667**	1000G_2504_high_coverage/data/ERR3242634/HG03667.final.cram
**HG03668**	1000G_2504_high_coverage/data/ERR3242635/HG03668.final.cram
**HG03015**	1000G_2504_high_coverage/data/ERR3242636/HG03015.final.cram
**HG03016**	1000G_2504_high_coverage/data/ERR3242637/HG03016.final.cram
**HG03702**	1000G_2504_high_coverage/data/ERR3242638/HG03702.final.cram
**HG03703**	1000G_2504_high_coverage/data/ERR3242639/HG03703.final.cram
**HG03708**	1000G_2504_high_coverage/data/ERR3242640/HG03708.final.cram
**HG03709**	1000G_2504_high_coverage/data/ERR3242641/HG03709.final.cram
**HG03006**	1000G_2504_high_coverage/data/ERR3242642/HG03006.final.cram
**HG03007**	1000G_2504_high_coverage/data/ERR3242643/HG03007.final.cram
**HG03615**	1000G_2504_high_coverage/data/ERR3242644/HG03615.final.cram
**HG03616**	1000G_2504_high_coverage/data/ERR3242645/HG03616.final.cram
**HG03793**	1000G_2504_high_coverage/data/ERR3242646/HG03793.final.cram
**HG03796**	1000G_2504_high_coverage/data/ERR3242647/HG03796.final.cram
**HG03800**	1000G_2504_high_coverage/data/ERR3242648/HG03800.final.cram
**HG03803**	1000G_2504_high_coverage/data/ERR3242649/HG03803.final.cram
**HG03802**	1000G_2504_high_coverage/data/ERR3242650/HG03802.final.cram
**HG03815**	1000G_2504_high_coverage/data/ERR3242651/HG03815.final.cram
**HG03814**	1000G_2504_high_coverage/data/ERR3242652/HG03814.final.cram
**HG03824**	1000G_2504_high_coverage/data/ERR3242653/HG03824.final.cram
**HG03823**	1000G_2504_high_coverage/data/ERR3242654/HG03823.final.cram
**HG03826**	1000G_2504_high_coverage/data/ERR3242655/HG03826.final.cram
**HG03830**	1000G_2504_high_coverage/data/ERR3242656/HG03830.final.cram
**HG03829**	1000G_2504_high_coverage/data/ERR3242657/HG03829.final.cram
**HG03833**	1000G_2504_high_coverage/data/ERR3242658/HG03833.final.cram
**HG03832**	1000G_2504_high_coverage/data/ERR3242659/HG03832.final.cram
**HG03914**	1000G_2504_high_coverage/data/ERR3242660/HG03914.final.cram
**HG03913**	1000G_2504_high_coverage/data/ERR3242661/HG03913.final.cram
**HG03941**	1000G_2504_high_coverage/data/ERR3242662/HG03941.final.cram
**HG03940**	1000G_2504_high_coverage/data/ERR3242663/HG03940.final.cram
**HG04155**	1000G_2504_high_coverage/data/ERR3242664/HG04155.final.cram
**HG04156**	1000G_2504_high_coverage/data/ERR3242665/HG04156.final.cram
**HG03644**	1000G_2504_high_coverage/data/ERR3242666/HG03644.final.cram
**HG03643**	1000G_2504_high_coverage/data/ERR3242667/HG03643.final.cram
**HG03679**	1000G_2504_high_coverage/data/ERR3242668/HG03679.final.cram
**HG03642**	1000G_2504_high_coverage/data/ERR3242669/HG03642.final.cram
**HG03673**	1000G_2504_high_coverage/data/ERR3242670/HG03673.final.cram
**HG03684**	1000G_2504_high_coverage/data/ERR3242671/HG03684.final.cram

**HG03885**	1000G_2504_high_coverage/data/ERR3242672/HG03885.final.cram
**HG03886**	1000G_2504_high_coverage/data/ERR3242673/HG03886.final.cram
**HG03692**	1000G_2504_high_coverage/data/ERR3242674/HG03692.final.cram
**HG03693**	1000G_2504_high_coverage/data/ERR3242675/HG03693.final.cram
**HG03740**	1000G_2504_high_coverage/data/ERR3242676/HG03740.final.cram
**HG03741**	1000G_2504_high_coverage/data/ERR3242677/HG03741.final.cram
**HG03884**	1000G_2504_high_coverage/data/ERR3242678/HG03884.final.cram
**HG03743**	1000G_2504_high_coverage/data/ERR3242679/HG03743.final.cram
**HG03757**	1000G_2504_high_coverage/data/ERR3242680/HG03757.final.cram
**HG03746**	1000G_2504_high_coverage/data/ERR3242681/HG03746.final.cram
**HG03888**	1000G_2504_high_coverage/data/ERR3242682/HG03888.final.cram
**HG03750**	1000G_2504_high_coverage/data/ERR3242683/HG03750.final.cram
**HG03754**	1000G_2504_high_coverage/data/ERR3242684/HG03754.final.cram
**HG03756**	1000G_2504_high_coverage/data/ERR3242685/HG03756.final.cram
**HG03844**	1000G_2504_high_coverage/data/ERR3242686/HG03844.final.cram
**HG04006**	1000G_2504_high_coverage/data/ERR3242687/HG04006.final.cram
**HG03838**	1000G_2504_high_coverage/data/ERR3242688/HG03838.final.cram
**HG03849**	1000G_2504_high_coverage/data/ERR3242689/HG03849.final.cram
**HG03950**	1000G_2504_high_coverage/data/ERR3242690/HG03950.final.cram
**HG03858**	1000G_2504_high_coverage/data/ERR3242691/HG03858.final.cram
**HG03887**	1000G_2504_high_coverage/data/ERR3242692/HG03887.final.cram
**HG03986**	1000G_2504_high_coverage/data/ERR3242693/HG03986.final.cram
**HG03896**	1000G_2504_high_coverage/data/ERR3242694/HG03896.final.cram
**HG03898**	1000G_2504_high_coverage/data/ERR3242695/HG03898.final.cram
**HG03900**	1000G_2504_high_coverage/data/ERR3242696/HG03900.final.cram
**HG03943**	1000G_2504_high_coverage/data/ERR3242697/HG03943.final.cram
**HG03713**	1000G_2504_high_coverage/data/ERR3242698/HG03713.final.cram
**HG03716**	1000G_2504_high_coverage/data/ERR3242699/HG03716.final.cram
**HG03714**	1000G_2504_high_coverage/data/ERR3242700/HG03714.final.cram
**HG03717**	1000G_2504_high_coverage/data/ERR3242701/HG03717.final.cram
**HG03722**	1000G_2504_high_coverage/data/ERR3242702/HG03722.final.cram
**HG03730**	1000G_2504_high_coverage/data/ERR3242703/HG03730.final.cram
**HG03727**	1000G_2504_high_coverage/data/ERR3242704/HG03727.final.cram
**HG03729**	1000G_2504_high_coverage/data/ERR3242705/HG03729.final.cram
**HG03731**	1000G_2504_high_coverage/data/ERR3242706/HG03731.final.cram
**HG03771**	1000G_2504_high_coverage/data/ERR3242707/HG03771.final.cram
**HG03770**	1000G_2504_high_coverage/data/ERR3242708/HG03770.final.cram
**HG03882**	1000G_2504_high_coverage/data/ERR3242709/HG03882.final.cram
**HG03781**	1000G_2504_high_coverage/data/ERR3242710/HG03781.final.cram
**HG03775**	1000G_2504_high_coverage/data/ERR3242711/HG03775.final.cram
**HG03773**	1000G_2504_high_coverage/data/ERR3242712/HG03773.final.cram
**HG03772**	1000G_2504_high_coverage/data/ERR3242713/HG03772.final.cram
**HG03786**	1000G_2504_high_coverage/data/ERR3242714/HG03786.final.cram
**HG03784**	1000G_2504_high_coverage/data/ERR3242715/HG03784.final.cram
**HG03787**	1000G_2504_high_coverage/data/ERR3242716/HG03787.final.cram
**HG03788**	1000G_2504_high_coverage/data/ERR3242717/HG03788.final.cram
**HG03789**	1000G_2504_high_coverage/data/ERR3242718/HG03789.final.cram
**HG03864**	1000G_2504_high_coverage/data/ERR3242719/HG03864.final.cram
**HG03869**	1000G_2504_high_coverage/data/ERR3242720/HG03869.final.cram
**HG03862**	1000G_2504_high_coverage/data/ERR3242721/HG03862.final.cram
**HG03874**	1000G_2504_high_coverage/data/ERR3242722/HG03874.final.cram
**HG03978**	1000G_2504_high_coverage/data/ERR3242723/HG03978.final.cram
**HG03971**	1000G_2504_high_coverage/data/ERR3242724/HG03971.final.cram
**HG03974**	1000G_2504_high_coverage/data/ERR3242725/HG03974.final.cram
**HG03976**	1000G_2504_high_coverage/data/ERR3242726/HG03976.final.cram
**HG03973**	1000G_2504_high_coverage/data/ERR3242727/HG03973.final.cram
**HG04002**	1000G_2504_high_coverage/data/ERR3242728/HG04002.final.cram
**HG04017**	1000G_2504_high_coverage/data/ERR3242729/HG04017.final.cram
**HG04026**	1000G_2504_high_coverage/data/ERR3242730/HG04026.final.cram
**HG02439**	1000G_2504_high_coverage/data/ERR3242731/HG02439.final.cram
**HG02455**	1000G_2504_high_coverage/data/ERR3242732/HG02455.final.cram
**HG02476**	1000G_2504_high_coverage/data/ERR3242733/HG02476.final.cram
**HG02477**	1000G_2504_high_coverage/data/ERR3242734/HG02477.final.cram
**HG02481**	1000G_2504_high_coverage/data/ERR3242735/HG02481.final.cram
**HG02501**	1000G_2504_high_coverage/data/ERR3242736/HG02501.final.cram

**HG02502**	1000G_2504_high_coverage/data/ERR3242737/HG02502.final.cram
**HG02505**	1000G_2504_high_coverage/data/ERR3242738/HG02505.final.cram
**HG02536**	1000G_2504_high_coverage/data/ERR3242739/HG02536.final.cram
**HG02541**	1000G_2504_high_coverage/data/ERR3242740/HG02541.final.cram
**HG02545**	1000G_2504_high_coverage/data/ERR3242741/HG02545.final.cram
**HG02546**	1000G_2504_high_coverage/data/ERR3242742/HG02546.final.cram
**HG02549**	1000G_2504_high_coverage/data/ERR3242743/HG02549.final.cram
**HG02554**	1000G_2504_high_coverage/data/ERR3242744/HG02554.final.cram
**HG02555**	1000G_2504_high_coverage/data/ERR3242745/HG02555.final.cram
**HG02557**	1000G_2504_high_coverage/data/ERR3242746/HG02557.final.cram
**HG02558**	1000G_2504_high_coverage/data/ERR3242747/HG02558.final.cram
**HG02577**	1000G_2504_high_coverage/data/ERR3242748/HG02577.final.cram
**HG02580**	1000G_2504_high_coverage/data/ERR3242749/HG02580.final.cram
**HG01088**	1000G_2504_high_coverage/data/ERR3242750/HG01088.final.cram
**HG01089**	1000G_2504_high_coverage/data/ERR3242751/HG01089.final.cram
**HG01302**	1000G_2504_high_coverage/data/ERR3242752/HG01302.final.cram
**HG01303**	1000G_2504_high_coverage/data/ERR3242753/HG01303.final.cram
**HG01311**	1000G_2504_high_coverage/data/ERR3242754/HG01311.final.cram
**HG01312**	1000G_2504_high_coverage/data/ERR3242755/HG01312.final.cram
**HG01323**	1000G_2504_high_coverage/data/ERR3242756/HG01323.final.cram
**HG01325**	1000G_2504_high_coverage/data/ERR3242757/HG01325.final.cram
**HG01326**	1000G_2504_high_coverage/data/ERR3242758/HG01326.final.cram
**HG01392**	1000G_2504_high_coverage/data/ERR3242759/HG01392.final.cram
**HG01393**	1000G_2504_high_coverage/data/ERR3242760/HG01393.final.cram
**HG01395**	1000G_2504_high_coverage/data/ERR3242761/HG01395.final.cram
**HG01396**	1000G_2504_high_coverage/data/ERR3242762/HG01396.final.cram
**HG01402**	1000G_2504_high_coverage/data/ERR3242763/HG01402.final.cram
**HG01403**	1000G_2504_high_coverage/data/ERR3242764/HG01403.final.cram
**HG01413**	1000G_2504_high_coverage/data/ERR3242765/HG01413.final.cram
**HG01414**	1000G_2504_high_coverage/data/ERR3242766/HG01414.final.cram
**HG00742**	1000G_2504_high_coverage/data/ERR3242767/HG00742.final.cram
**HG00743**	1000G_2504_high_coverage/data/ERR3242768/HG00743.final.cram
**HG01058**	1000G_2504_high_coverage/data/ERR3242769/HG01058.final.cram
**HG01063**	1000G_2504_high_coverage/data/ERR3242770/HG01063.final.cram
**HG01064**	1000G_2504_high_coverage/data/ERR3242771/HG01064.final.cram
**HG01077**	1000G_2504_high_coverage/data/ERR3242772/HG01077.final.cram
**HG01092**	1000G_2504_high_coverage/data/ERR3242773/HG01092.final.cram
**HG01161**	1000G_2504_high_coverage/data/ERR3242774/HG01161.final.cram
**HG01162**	1000G_2504_high_coverage/data/ERR3242775/HG01162.final.cram
**HG01164**	1000G_2504_high_coverage/data/ERR3242776/HG01164.final.cram
**HG01200**	1000G_2504_high_coverage/data/ERR3242777/HG01200.final.cram
**HG01286**	1000G_2504_high_coverage/data/ERR3242778/HG01286.final.cram
**HG01305**	1000G_2504_high_coverage/data/ERR3242779/HG01305.final.cram
**HG01308**	1000G_2504_high_coverage/data/ERR3242780/HG01308.final.cram
**HG01398**	1000G_2504_high_coverage/data/ERR3242781/HG01398.final.cram
**HG01405**	1000G_2504_high_coverage/data/ERR3242782/HG01405.final.cram
**HG01412**	1000G_2504_high_coverage/data/ERR3242783/HG01412.final.cram
**HG01119**	1000G_2504_high_coverage/data/ERR3242784/HG01119.final.cram
**HG01121**	1000G_2504_high_coverage/data/ERR3242785/HG01121.final.cram
**HG01122**	1000G_2504_high_coverage/data/ERR3242786/HG01122.final.cram
**HG01130**	1000G_2504_high_coverage/data/ERR3242787/HG01130.final.cram
**HG01131**	1000G_2504_high_coverage/data/ERR3242788/HG01131.final.cram
**HG01142**	1000G_2504_high_coverage/data/ERR3242789/HG01142.final.cram
**HG01269**	1000G_2504_high_coverage/data/ERR3242790/HG01269.final.cram
**HG01280**	1000G_2504_high_coverage/data/ERR3242791/HG01280.final.cram
**HG01281**	1000G_2504_high_coverage/data/ERR3242792/HG01281.final.cram
**HG01284**	1000G_2504_high_coverage/data/ERR3242793/HG01284.final.cram
**HG01362**	1000G_2504_high_coverage/data/ERR3242794/HG01362.final.cram
**HG01363**	1000G_2504_high_coverage/data/ERR3242795/HG01363.final.cram
**HG01369**	1000G_2504_high_coverage/data/ERR3242796/HG01369.final.cram
**HG01372**	1000G_2504_high_coverage/data/ERR3242797/HG01372.final.cram
**HG01431**	1000G_2504_high_coverage/data/ERR3242798/HG01431.final.cram
**HG01432**	1000G_2504_high_coverage/data/ERR3242799/HG01432.final.cram
**HG01435**	1000G_2504_high_coverage/data/ERR3242800/HG01435.final.cram
**HG01443**	1000G_2504_high_coverage/data/ERR3242801/HG01443.final.cram

HG01444	1000G_2504_high_coverage/data/ERR3242802/HG01444.final.cram
HG01447	1000G_2504_high_coverage/data/ERR3242803/HG01447.final.cram
HG01459	1000G_2504_high_coverage/data/ERR3242804/HG01459.final.cram
HG01468	1000G_2504_high_coverage/data/ERR3242805/HG01468.final.cram
HG01474	1000G_2504_high_coverage/data/ERR3242806/HG01474.final.cram
HG01479	1000G_2504_high_coverage/data/ERR3242807/HG01479.final.cram
HG01485	1000G_2504_high_coverage/data/ERR3242808/HG01485.final.cram
HG01486	1000G_2504_high_coverage/data/ERR3242809/HG01486.final.cram
HG01556	1000G_2504_high_coverage/data/ERR3242810/HG01556.final.cram
HG01961	1000G_2504_high_coverage/data/ERR3242811/HG01961.final.cram
HG01965	1000G_2504_high_coverage/data/ERR3242812/HG01965.final.cram
HG02006	1000G_2504_high_coverage/data/ERR3242813/HG02006.final.cram
HG02102	1000G_2504_high_coverage/data/ERR3242814/HG02102.final.cram
HG02150	1000G_2504_high_coverage/data/ERR3242815/HG02150.final.cram
HG02252	1000G_2504_high_coverage/data/ERR3242816/HG02252.final.cram
HG02253	1000G_2504_high_coverage/data/ERR3242817/HG02253.final.cram
HG02262	1000G_2504_high_coverage/data/ERR3242818/HG02262.final.cram
HG02265	1000G_2504_high_coverage/data/ERR3242819/HG02265.final.cram
HG02266	1000G_2504_high_coverage/data/ERR3242820/HG02266.final.cram
HG02274	1000G_2504_high_coverage/data/ERR3242821/HG02274.final.cram
HG02275	1000G_2504_high_coverage/data/ERR3242822/HG02275.final.cram
HG02304	1000G_2504_high_coverage/data/ERR3242823/HG02304.final.cram
HG02312	1000G_2504_high_coverage/data/ERR3242824/HG02312.final.cram
HG02345	1000G_2504_high_coverage/data/ERR3242825/HG02345.final.cram
HG02348	1000G_2504_high_coverage/data/ERR3242826/HG02348.final.cram
HG02425	1000G_2504_high_coverage/data/ERR3242827/HG02425.final.cram
HG02408	1000G_2504_high_coverage/data/ERR3242828/HG02408.final.cram
HG02410	1000G_2504_high_coverage/data/ERR3242829/HG02410.final.cram
HG03237	1000G_2504_high_coverage/data/ERR3242830/HG03237.final.cram
HG03238	1000G_2504_high_coverage/data/ERR3242831/HG03238.final.cram
HG03607	1000G_2504_high_coverage/data/ERR3242832/HG03607.final.cram
HG03805	1000G_2504_high_coverage/data/ERR3242833/HG03805.final.cram
HG03908	1000G_2504_high_coverage/data/ERR3242834/HG03908.final.cram
HG03907	1000G_2504_high_coverage/data/ERR3242835/HG03907.final.cram
HG03926	1000G_2504_high_coverage/data/ERR3242836/HG03926.final.cram
HG03925	1000G_2504_high_coverage/data/ERR3242837/HG03925.final.cram
HG03594	1000G_2504_high_coverage/data/ERR3242838/HG03594.final.cram
HG03595	1000G_2504_high_coverage/data/ERR3242839/HG03595.final.cram
HG03809	1000G_2504_high_coverage/data/ERR3242840/HG03809.final.cram
HG03808	1000G_2504_high_coverage/data/ERR3242841/HG03808.final.cram
HG03009	1000G_2504_high_coverage/data/ERR3242842/HG03009.final.cram
HG03812	1000G_2504_high_coverage/data/ERR3242843/HG03812.final.cram
HG03817	1000G_2504_high_coverage/data/ERR3242844/HG03817.final.cram
HG03902	1000G_2504_high_coverage/data/ERR3242845/HG03902.final.cram
HG03012	1000G_2504_high_coverage/data/ERR3242846/HG03012.final.cram
HG03905	1000G_2504_high_coverage/data/ERR3242847/HG03905.final.cram
HG03911	1000G_2504_high_coverage/data/ERR3242848/HG03911.final.cram
HG03910	1000G_2504_high_coverage/data/ERR3242849/HG03910.final.cram
HG03916	1000G_2504_high_coverage/data/ERR3242850/HG03916.final.cram
HG03919	1000G_2504_high_coverage/data/ERR3242851/HG03919.final.cram
HG03928	1000G_2504_high_coverage/data/ERR3242852/HG03928.final.cram
HG03931	1000G_2504_high_coverage/data/ERR3242853/HG03931.final.cram
HG03585	1000G_2504_high_coverage/data/ERR3242854/HG03585.final.cram
HG03937	1000G_2504_high_coverage/data/ERR3242855/HG03937.final.cram
HG03934	1000G_2504_high_coverage/data/ERR3242856/HG03934.final.cram
HG04146	1000G_2504_high_coverage/data/ERR3242857/HG04146.final.cram
HG03589	1000G_2504_high_coverage/data/ERR3242858/HG03589.final.cram
HG04159	1000G_2504_high_coverage/data/ERR3242859/HG04159.final.cram
HG03593	1000G_2504_high_coverage/data/ERR3242860/HG03593.final.cram
HG04186	1000G_2504_high_coverage/data/ERR3242861/HG04186.final.cram
HG03598	1000G_2504_high_coverage/data/ERR3242862/HG03598.final.cram
HG03755	1000G_2504_high_coverage/data/ERR3242863/HG03755.final.cram
HG03850	1000G_2504_high_coverage/data/ERR3242864/HG03850.final.cram
HG03894	1000G_2504_high_coverage/data/ERR3242865/HG03894.final.cram
HG03897	1000G_2504_high_coverage/data/ERR3242866/HG03897.final.cram

**HG03944**	1000G_2504_high_coverage/data/ERR3242867/HG03944.final.cram
**HG03646**	1000G_2504_high_coverage/data/ERR3242868/HG03646.final.cram
**HG03645**	1000G_2504_high_coverage/data/ERR3242869/HG03645.final.cram
**HG03999**	1000G_2504_high_coverage/data/ERR3242870/HG03999.final.cram
**HG03672**	1000G_2504_high_coverage/data/ERR3242871/HG03672.final.cram
**HG03680**	1000G_2504_high_coverage/data/ERR3242872/HG03680.final.cram
**HG03681**	1000G_2504_high_coverage/data/ERR3242873/HG03681.final.cram
**HG03685**	1000G_2504_high_coverage/data/ERR3242874/HG03685.final.cram
**HG03686**	1000G_2504_high_coverage/data/ERR3242875/HG03686.final.cram
**HG03687**	1000G_2504_high_coverage/data/ERR3242876/HG03687.final.cram
**HG03689**	1000G_2504_high_coverage/data/ERR3242877/HG03689.final.cram
**HG03690**	1000G_2504_high_coverage/data/ERR3242878/HG03690.final.cram
**HG03691**	1000G_2504_high_coverage/data/ERR3242879/HG03691.final.cram
**HG03694**	1000G_2504_high_coverage/data/ERR3242880/HG03694.final.cram
**HG03695**	1000G_2504_high_coverage/data/ERR3242881/HG03695.final.cram
**HG03696**	1000G_2504_high_coverage/data/ERR3242882/HG03696.final.cram
**HG03697**	1000G_2504_high_coverage/data/ERR3242883/HG03697.final.cram
**HG03698**	1000G_2504_high_coverage/data/ERR3242884/HG03698.final.cram
**HG03711**	1000G_2504_high_coverage/data/ERR3242885/HG03711.final.cram
**HG03733**	1000G_2504_high_coverage/data/ERR3242886/HG03733.final.cram
**HG03736**	1000G_2504_high_coverage/data/ERR3242887/HG03736.final.cram
**HG03738**	1000G_2504_high_coverage/data/ERR3242888/HG03738.final.cram
**HG03744**	1000G_2504_high_coverage/data/ERR3242889/HG03744.final.cram
**HG03745**	1000G_2504_high_coverage/data/ERR3242890/HG03745.final.cram
**HG03752**	1000G_2504_high_coverage/data/ERR3242891/HG03752.final.cram
**HG03753**	1000G_2504_high_coverage/data/ERR3242892/HG03753.final.cram
**HG03760**	1000G_2504_high_coverage/data/ERR3242893/HG03760.final.cram
**HG03836**	1000G_2504_high_coverage/data/ERR3242894/HG03836.final.cram
**HG03837**	1000G_2504_high_coverage/data/ERR3242895/HG03837.final.cram
**HG03846**	1000G_2504_high_coverage/data/ERR3242896/HG03846.final.cram
**HG03848**	1000G_2504_high_coverage/data/ERR3242897/HG03848.final.cram
**HG03851**	1000G_2504_high_coverage/data/ERR3242898/HG03851.final.cram
**HG03856**	1000G_2504_high_coverage/data/ERR3242899/HG03856.final.cram
**HG03895**	1000G_2504_high_coverage/data/ERR3242900/HG03895.final.cram
**HG03890**	1000G_2504_high_coverage/data/ERR3242901/HG03890.final.cram
**HG04033**	1000G_2504_high_coverage/data/ERR3242902/HG04033.final.cram
**HG03947**	1000G_2504_high_coverage/data/ERR3242903/HG03947.final.cram
**HG03953**	1000G_2504_high_coverage/data/ERR3242904/HG03953.final.cram
**HG03949**	1000G_2504_high_coverage/data/ERR3242905/HG03949.final.cram
**HG04035**	1000G_2504_high_coverage/data/ERR3242906/HG04035.final.cram
**HG04029**	1000G_2504_high_coverage/data/ERR3242907/HG04029.final.cram
**HG04042**	1000G_2504_high_coverage/data/ERR3242908/HG04042.final.cram
**HG04099**	1000G_2504_high_coverage/data/ERR3242909/HG04099.final.cram
**HG03977**	1000G_2504_high_coverage/data/ERR3242910/HG03977.final.cram
**HG03780**	1000G_2504_high_coverage/data/ERR3242911/HG03780.final.cram
**HG03718**	1000G_2504_high_coverage/data/ERR3242912/HG03718.final.cram
**HG04018**	1000G_2504_high_coverage/data/ERR3242913/HG04018.final.cram
**HG03742**	1000G_2504_high_coverage/data/ERR3242914/HG03742.final.cram
**HG03779**	1000G_2504_high_coverage/data/ERR3242915/HG03779.final.cram
**HG03777**	1000G_2504_high_coverage/data/ERR3242916/HG03777.final.cram
**HG03785**	1000G_2504_high_coverage/data/ERR3242917/HG03785.final.cram
**HG03790**	1000G_2504_high_coverage/data/ERR3242918/HG03790.final.cram
**HG03792**	1000G_2504_high_coverage/data/ERR3242919/HG03792.final.cram
**HG03782**	1000G_2504_high_coverage/data/ERR3242920/HG03782.final.cram
**HG03873**	1000G_2504_high_coverage/data/ERR3242921/HG03873.final.cram
**HG03861**	1000G_2504_high_coverage/data/ERR3242922/HG03861.final.cram
**HG03863**	1000G_2504_high_coverage/data/ERR3242923/HG03863.final.cram
**HG03872**	1000G_2504_high_coverage/data/ERR3242924/HG03872.final.cram
**HG03870**	1000G_2504_high_coverage/data/ERR3242925/HG03870.final.cram
**HG03867**	1000G_2504_high_coverage/data/ERR3242926/HG03867.final.cram
**HG03963**	1000G_2504_high_coverage/data/ERR3242927/HG03963.final.cram
**HG03960**	1000G_2504_high_coverage/data/ERR3242928/HG03960.final.cram
**HG03968**	1000G_2504_high_coverage/data/ERR3242929/HG03968.final.cram
**HG03720**	1000G_2504_high_coverage/data/ERR3242930/HG03720.final.cram
**HG04019**	1000G_2504_high_coverage/data/ERR3242931/HG04019.final.cram

**HG04025**	1000G_2504_high_coverage/data/ERR3242932/HG04025.final.cram
**HG04020**	1000G_2504_high_coverage/data/ERR3242933/HG04020.final.cram
**HG04014**	1000G_2504_high_coverage/data/ERR3242934/HG04014.final.cram
**HG04015**	1000G_2504_high_coverage/data/ERR3242935/HG04015.final.cram
**HG04022**	1000G_2504_high_coverage/data/ERR3242936/HG04022.final.cram
**HG04063**	1000G_2504_high_coverage/data/ERR3242937/HG04063.final.cram
**HG04093**	1000G_2504_high_coverage/data/ERR3242938/HG04093.final.cram
**HG04118**	1000G_2504_high_coverage/data/ERR3242939/HG04118.final.cram
**HG02568**	1000G_2504_high_coverage/data/ERR3242940/HG02568.final.cram
**HG02759**	1000G_2504_high_coverage/data/ERR3242941/HG02759.final.cram
**HG02760**	1000G_2504_high_coverage/data/ERR3242942/HG02760.final.cram
**HG02763**	1000G_2504_high_coverage/data/ERR3242943/HG02763.final.cram
**HG02870**	1000G_2504_high_coverage/data/ERR3242944/HG02870.final.cram
**HG02878**	1000G_2504_high_coverage/data/ERR3242945/HG02878.final.cram
**HG02879**	1000G_2504_high_coverage/data/ERR3242946/HG02879.final.cram
**HG02881**	1000G_2504_high_coverage/data/ERR3242947/HG02881.final.cram
**HG02882**	1000G_2504_high_coverage/data/ERR3242948/HG02882.final.cram
**HG02982**	1000G_2504_high_coverage/data/ERR3242949/HG02982.final.cram
**HG02983**	1000G_2504_high_coverage/data/ERR3242950/HG02983.final.cram
**HG03054**	1000G_2504_high_coverage/data/ERR3242951/HG03054.final.cram
**HG03060**	1000G_2504_high_coverage/data/ERR3242952/HG03060.final.cram
**HG03063**	1000G_2504_high_coverage/data/ERR3242953/HG03063.final.cram
**HG03064**	1000G_2504_high_coverage/data/ERR3242954/HG03064.final.cram
**HG03073**	1000G_2504_high_coverage/data/ERR3242955/HG03073.final.cram
**HG03074**	1000G_2504_high_coverage/data/ERR3242956/HG03074.final.cram
**HG03077**	1000G_2504_high_coverage/data/ERR3242957/HG03077.final.cram
**HG03079**	1000G_2504_high_coverage/data/ERR3242958/HG03079.final.cram
**HG03082**	1000G_2504_high_coverage/data/ERR3242959/HG03082.final.cram
**HG03086**	1000G_2504_high_coverage/data/ERR3242960/HG03086.final.cram
**HG03091**	1000G_2504_high_coverage/data/ERR3242961/HG03091.final.cram
**HG03095**	1000G_2504_high_coverage/data/ERR3242962/HG03095.final.cram
**HG03224**	1000G_2504_high_coverage/data/ERR3242963/HG03224.final.cram
**HG03225**	1000G_2504_high_coverage/data/ERR3242964/HG03225.final.cram
**HG03378**	1000G_2504_high_coverage/data/ERR3242965/HG03378.final.cram
**HG03382**	1000G_2504_high_coverage/data/ERR3242966/HG03382.final.cram
**HG03394**	1000G_2504_high_coverage/data/ERR3242967/HG03394.final.cram
**HG03432**	1000G_2504_high_coverage/data/ERR3242968/HG03432.final.cram
**HG03457**	1000G_2504_high_coverage/data/ERR3242969/HG03457.final.cram
**HG03558**	1000G_2504_high_coverage/data/ERR3242970/HG03558.final.cram
**HG03567**	1000G_2504_high_coverage/data/ERR3242971/HG03567.final.cram
**HG02938**	1000G_2504_high_coverage/data/ERR3242972/HG02938.final.cram
**HG02941**	1000G_2504_high_coverage/data/ERR3242973/HG02941.final.cram
**HG02968**	1000G_2504_high_coverage/data/ERR3242974/HG02968.final.cram
**HG02979**	1000G_2504_high_coverage/data/ERR3242975/HG02979.final.cram
**HG02981**	1000G_2504_high_coverage/data/ERR3242976/HG02981.final.cram
**HG03103**	1000G_2504_high_coverage/data/ERR3242977/HG03103.final.cram
**HG03105**	1000G_2504_high_coverage/data/ERR3242978/HG03105.final.cram
**HG03111**	1000G_2504_high_coverage/data/ERR3242979/HG03111.final.cram
**HG03112**	1000G_2504_high_coverage/data/ERR3242980/HG03112.final.cram
**HG03126**	1000G_2504_high_coverage/data/ERR3242981/HG03126.final.cram
**HG03127**	1000G_2504_high_coverage/data/ERR3242982/HG03127.final.cram
**HG03139**	1000G_2504_high_coverage/data/ERR3242983/HG03139.final.cram
**HG03157**	1000G_2504_high_coverage/data/ERR3242984/HG03157.final.cram
**HG03166**	1000G_2504_high_coverage/data/ERR3242985/HG03166.final.cram
**HG03175**	1000G_2504_high_coverage/data/ERR3242986/HG03175.final.cram
**HG03189**	1000G_2504_high_coverage/data/ERR3242987/HG03189.final.cram
**HG03190**	1000G_2504_high_coverage/data/ERR3242988/HG03190.final.cram
**HG03193**	1000G_2504_high_coverage/data/ERR3242989/HG03193.final.cram
**HG03202**	1000G_2504_high_coverage/data/ERR3242990/HG03202.final.cram
**HG03265**	1000G_2504_high_coverage/data/ERR3242991/HG03265.final.cram
**HG03297**	1000G_2504_high_coverage/data/ERR3242992/HG03297.final.cram
**HG03298**	1000G_2504_high_coverage/data/ERR3242993/HG03298.final.cram
**HG03303**	1000G_2504_high_coverage/data/ERR3242994/HG03303.final.cram
**HG03304**	1000G_2504_high_coverage/data/ERR3242995/HG03304.final.cram
**HG03311**	1000G_2504_high_coverage/data/ERR3242996/HG03311.final.cram

HG03313	1000G_2504_high_coverage/data/ERR3242997/HG03313.final.cram
HG03351	1000G_2504_high_coverage/data/ERR3242998/HG03351.final.cram
HG03352	1000G_2504_high_coverage/data/ERR3242999/HG03352.final.cram
HG03354	1000G_2504_high_coverage/data/ERR3243000/HG03354.final.cram
HG03363	1000G_2504_high_coverage/data/ERR3243001/HG03363.final.cram
HG03372	1000G_2504_high_coverage/data/ERR3243002/HG03372.final.cram
HG03499	1000G_2504_high_coverage/data/ERR3243003/HG03499.final.cram
HG03511	1000G_2504_high_coverage/data/ERR3243004/HG03511.final.cram
HG03517	1000G_2504_high_coverage/data/ERR3243005/HG03517.final.cram
HG03518	1000G_2504_high_coverage/data/ERR3243006/HG03518.final.cram
HG03520	1000G_2504_high_coverage/data/ERR3243007/HG03520.final.cram
HG03521	1000G_2504_high_coverage/data/ERR3243008/HG03521.final.cram
HG02493	1000G_2504_high_coverage/data/ERR3243009/HG02493.final.cram
HG02494	1000G_2504_high_coverage/data/ERR3243010/HG02494.final.cram
HG02648	1000G_2504_high_coverage/data/ERR3243011/HG02648.final.cram
HG02649	1000G_2504_high_coverage/data/ERR3243012/HG02649.final.cram
HG02651	1000G_2504_high_coverage/data/ERR3243013/HG02651.final.cram
HG02652	1000G_2504_high_coverage/data/ERR3243014/HG02652.final.cram
HG02681	1000G_2504_high_coverage/data/ERR3243015/HG02681.final.cram
HG02682	1000G_2504_high_coverage/data/ERR3243016/HG02682.final.cram
HG02690	1000G_2504_high_coverage/data/ERR3243017/HG02690.final.cram
HG02691	1000G_2504_high_coverage/data/ERR3243018/HG02691.final.cram
HG02699	1000G_2504_high_coverage/data/ERR3243019/HG02699.final.cram
HG02700	1000G_2504_high_coverage/data/ERR3243020/HG02700.final.cram
HG02736	1000G_2504_high_coverage/data/ERR3243021/HG02736.final.cram
HG02737	1000G_2504_high_coverage/data/ERR3243022/HG02737.final.cram
HG02774	1000G_2504_high_coverage/data/ERR3243023/HG02774.final.cram
HG02775	1000G_2504_high_coverage/data/ERR3243024/HG02775.final.cram
HG02792	1000G_2504_high_coverage/data/ERR3243025/HG02792.final.cram
HG02793	1000G_2504_high_coverage/data/ERR3243026/HG02793.final.cram
HG03228	1000G_2504_high_coverage/data/ERR3243027/HG03228.final.cram
HG03229	1000G_2504_high_coverage/data/ERR3243028/HG03229.final.cram
HG03624	1000G_2504_high_coverage/data/ERR3243029/HG03624.final.cram
HG03625	1000G_2504_high_coverage/data/ERR3243030/HG03625.final.cram
HG03640	1000G_2504_high_coverage/data/ERR3243031/HG03640.final.cram
HG03649	1000G_2504_high_coverage/data/ERR3243032/HG03649.final.cram
HG03652	1000G_2504_high_coverage/data/ERR3243033/HG03652.final.cram
HG03653	1000G_2504_high_coverage/data/ERR3243034/HG03653.final.cram
HG03705	1000G_2504_high_coverage/data/ERR3243035/HG03705.final.cram
HG03706	1000G_2504_high_coverage/data/ERR3243036/HG03706.final.cram
HG03762	1000G_2504_high_coverage/data/ERR3243037/HG03762.final.cram
HG01593	1000G_2504_high_coverage/data/ERR3243038/HG01593.final.cram
HG02597	1000G_2504_high_coverage/data/ERR3243039/HG02597.final.cram
HG02778	1000G_2504_high_coverage/data/ERR3243040/HG02778.final.cram
HG03021	1000G_2504_high_coverage/data/ERR3243041/HG03021.final.cram
HG03636	1000G_2504_high_coverage/data/ERR3243042/HG03636.final.cram
HG03765	1000G_2504_high_coverage/data/ERR3243043/HG03765.final.cram
HG03767	1000G_2504_high_coverage/data/ERR3243044/HG03767.final.cram
HG01583	1000G_2504_high_coverage/data/ERR3243045/HG01583.final.cram
HG01586	1000G_2504_high_coverage/data/ERR3243046/HG01586.final.cram
HG01589	1000G_2504_high_coverage/data/ERR3243047/HG01589.final.cram
HG02694	1000G_2504_high_coverage/data/ERR3243048/HG02694.final.cram
HG02731	1000G_2504_high_coverage/data/ERR3243049/HG02731.final.cram
HG02780	1000G_2504_high_coverage/data/ERR3243050/HG02780.final.cram
HG03018	1000G_2504_high_coverage/data/ERR3243051/HG03018.final.cram
HG03019	1000G_2504_high_coverage/data/ERR3243052/HG03019.final.cram
HG03022	1000G_2504_high_coverage/data/ERR3243053/HG03022.final.cram
HG03629	1000G_2504_high_coverage/data/ERR3243054/HG03629.final.cram
HG03631	1000G_2504_high_coverage/data/ERR3243055/HG03631.final.cram
HG03660	1000G_2504_high_coverage/data/ERR3243056/HG03660.final.cram
HG03663	1000G_2504_high_coverage/data/ERR3243057/HG03663.final.cram
HG04158	1000G_2504_high_coverage/data/ERR3243058/HG04158.final.cram
HG04185	1000G_2504_high_coverage/data/ERR3243059/HG04185.final.cram
HG04131	1000G_2504_high_coverage/data/ERR3243060/HG04131.final.cram
HG04134	1000G_2504_high_coverage/data/ERR3243061/HG04134.final.cram

**HG04140**	1000G_2504_high_coverage/data/ERR3243062/HG04140.final.cram
**HG04141**	1000G_2504_high_coverage/data/ERR3243063/HG04141.final.cram
**HG04173**	1000G_2504_high_coverage/data/ERR3243064/HG04173.final.cram
**HG04182**	1000G_2504_high_coverage/data/ERR3243065/HG04182.final.cram
**HG04183**	1000G_2504_high_coverage/data/ERR3243066/HG04183.final.cram
**HG03821**	1000G_2504_high_coverage/data/ERR3243067/HG03821.final.cram
**HG03922**	1000G_2504_high_coverage/data/ERR3243068/HG03922.final.cram
**HG04180**	1000G_2504_high_coverage/data/ERR3243069/HG04180.final.cram
**HG03600**	1000G_2504_high_coverage/data/ERR3243070/HG03600.final.cram
**HG03603**	1000G_2504_high_coverage/data/ERR3243071/HG03603.final.cram
**HG03604**	1000G_2504_high_coverage/data/ERR3243072/HG03604.final.cram
**HG03611**	1000G_2504_high_coverage/data/ERR3243073/HG03611.final.cram
**HG03917**	1000G_2504_high_coverage/data/ERR3243074/HG03917.final.cram
**HG03920**	1000G_2504_high_coverage/data/ERR3243075/HG03920.final.cram
**HG04144**	1000G_2504_high_coverage/data/ERR3243076/HG04144.final.cram
**HG04152**	1000G_2504_high_coverage/data/ERR3243077/HG04152.final.cram
**HG04153**	1000G_2504_high_coverage/data/ERR3243078/HG04153.final.cram
**HG04161**	1000G_2504_high_coverage/data/ERR3243079/HG04161.final.cram
**HG04162**	1000G_2504_high_coverage/data/ERR3243080/HG04162.final.cram
**HG04164**	1000G_2504_high_coverage/data/ERR3243081/HG04164.final.cram
**HG04171**	1000G_2504_high_coverage/data/ERR3243082/HG04171.final.cram
**HG04176**	1000G_2504_high_coverage/data/ERR3243083/HG04176.final.cram
**HG04177**	1000G_2504_high_coverage/data/ERR3243084/HG04177.final.cram
**HG04188**	1000G_2504_high_coverage/data/ERR3243085/HG04188.final.cram
**HG04189**	1000G_2504_high_coverage/data/ERR3243086/HG04189.final.cram
**HG04194**	1000G_2504_high_coverage/data/ERR3243087/HG04194.final.cram
**HG04195**	1000G_2504_high_coverage/data/ERR3243088/HG04195.final.cram
**HG03995**	1000G_2504_high_coverage/data/ERR3243089/HG03995.final.cram
**HG03899**	1000G_2504_high_coverage/data/ERR3243090/HG03899.final.cram
**HG03951**	1000G_2504_high_coverage/data/ERR3243091/HG03951.final.cram
**HG03857**	1000G_2504_high_coverage/data/ERR3243092/HG03857.final.cram
**HG03854**	1000G_2504_high_coverage/data/ERR3243093/HG03854.final.cram
**HG04100**	1000G_2504_high_coverage/data/ERR3243094/HG04100.final.cram
**HG04210**	1000G_2504_high_coverage/data/ERR3243095/HG04210.final.cram
**HG04075**	1000G_2504_high_coverage/data/ERR3243096/HG04075.final.cram
**HG04229**	1000G_2504_high_coverage/data/ERR3243097/HG04229.final.cram
**HG04227**	1000G_2504_high_coverage/data/ERR3243098/HG04227.final.cram
**HG03945**	1000G_2504_high_coverage/data/ERR3243099/HG03945.final.cram
**HG03955**	1000G_2504_high_coverage/data/ERR3243100/HG03955.final.cram
**HG03990**	1000G_2504_high_coverage/data/ERR3243101/HG03990.final.cram
**HG03991**	1000G_2504_high_coverage/data/ERR3243102/HG03991.final.cram
**HG03985**	1000G_2504_high_coverage/data/ERR3243103/HG03985.final.cram
**HG03989**	1000G_2504_high_coverage/data/ERR3243104/HG03989.final.cram
**HG03998**	1000G_2504_high_coverage/data/ERR3243105/HG03998.final.cram
**HG04003**	1000G_2504_high_coverage/data/ERR3243106/HG04003.final.cram
**HG04038**	1000G_2504_high_coverage/data/ERR3243107/HG04038.final.cram
**HG04039**	1000G_2504_high_coverage/data/ERR3243108/HG04039.final.cram
**HG04047**	1000G_2504_high_coverage/data/ERR3243109/HG04047.final.cram
**HG04107**	1000G_2504_high_coverage/data/ERR3243110/HG04107.final.cram
**HG04106**	1000G_2504_high_coverage/data/ERR3243111/HG04106.final.cram
**HG03774**	1000G_2504_high_coverage/data/ERR3243112/HG03774.final.cram
**HG04001**	1000G_2504_high_coverage/data/ERR3243113/HG04001.final.cram
**HG03866**	1000G_2504_high_coverage/data/ERR3243114/HG03866.final.cram
**HG03969**	1000G_2504_high_coverage/data/ERR3243115/HG03969.final.cram
**HG04023**	1000G_2504_high_coverage/data/ERR3243116/HG04023.final.cram
**HG04060**	1000G_2504_high_coverage/data/ERR3243117/HG04060.final.cram
**HG04090**	1000G_2504_high_coverage/data/ERR3243118/HG04090.final.cram
**HG04056**	1000G_2504_high_coverage/data/ERR3243119/HG04056.final.cram
**HG04054**	1000G_2504_high_coverage/data/ERR3243120/HG04054.final.cram
**HG04061**	1000G_2504_high_coverage/data/ERR3243121/HG04061.final.cram
**HG04062**	1000G_2504_high_coverage/data/ERR3243122/HG04062.final.cram
**HG04059**	1000G_2504_high_coverage/data/ERR3243123/HG04059.final.cram
**HG04076**	1000G_2504_high_coverage/data/ERR3243124/HG04076.final.cram
**HG04080**	1000G_2504_high_coverage/data/ERR3243125/HG04080.final.cram
**HG04094**	1000G_2504_high_coverage/data/ERR3243126/HG04094.final.cram

**HG04096**	1000G_2504_high_coverage/data/ERR3243127/HG04096.final.cram
**HG04098**	1000G_2504_high_coverage/data/ERR3243128/HG04098.final.cram
**HG03778**	1000G_2504_high_coverage/data/ERR3243129/HG03778.final.cram
**HG04209**	1000G_2504_high_coverage/data/ERR3243130/HG04209.final.cram
**HG04198**	1000G_2504_high_coverage/data/ERR3243131/HG04198.final.cram
**HG04200**	1000G_2504_high_coverage/data/ERR3243132/HG04200.final.cram
**HG04206**	1000G_2504_high_coverage/data/ERR3243133/HG04206.final.cram
**HG04202**	1000G_2504_high_coverage/data/ERR3243134/HG04202.final.cram
**HG04211**	1000G_2504_high_coverage/data/ERR3243135/HG04211.final.cram
**HG04212**	1000G_2504_high_coverage/data/ERR3243136/HG04212.final.cram
**HG04222**	1000G_2504_high_coverage/data/ERR3243137/HG04222.final.cram
**HG04214**	1000G_2504_high_coverage/data/ERR3243138/HG04214.final.cram
**HG04216**	1000G_2504_high_coverage/data/ERR3243139/HG04216.final.cram
**HG04239**	1000G_2504_high_coverage/data/ERR3243140/HG04239.final.cram
**HG04219**	1000G_2504_high_coverage/data/ERR3243141/HG04219.final.cram
**HG04225**	1000G_2504_high_coverage/data/ERR3243142/HG04225.final.cram
**HG04235**	1000G_2504_high_coverage/data/ERR3243143/HG04235.final.cram
**HG04238**	1000G_2504_high_coverage/data/ERR3243144/HG04238.final.cram
**HG03875**	1000G_2504_high_coverage/data/ERR3243145/HG03875.final.cram
**HG03965**	1000G_2504_high_coverage/data/ERR3243146/HG03965.final.cram
**HG04070**	1000G_2504_high_coverage/data/ERR3243147/HG04070.final.cram
**HG03868**	1000G_2504_high_coverage/data/ERR3243148/HG03868.final.cram
**HG03871**	1000G_2504_high_coverage/data/ERR3243149/HG03871.final.cram
**HG03967**	1000G_2504_high_coverage/data/ERR3243150/HG03967.final.cram
**HG00288**	1000G_2504_high_coverage/data/ERR3243151/HG00288.final.cram
**HG00290**	1000G_2504_high_coverage/data/ERR3243152/HG00290.final.cram
**HG00304**	1000G_2504_high_coverage/data/ERR3243153/HG00304.final.cram
**HG01765**	1000G_2504_high_coverage/data/ERR3243154/HG01765.final.cram
**HG01766**	1000G_2504_high_coverage/data/ERR3243155/HG01766.final.cram
**HG01767**	1000G_2504_high_coverage/data/ERR3243156/HG01767.final.cram
**HG01768**	1000G_2504_high_coverage/data/ERR3243157/HG01768.final.cram
**HG01785**	1000G_2504_high_coverage/data/ERR3243158/HG01785.final.cram
**HG01786**	1000G_2504_high_coverage/data/ERR3243159/HG01786.final.cram
**HG02219**	1000G_2504_high_coverage/data/ERR3243160/HG02219.final.cram
**NA18874**	1000G_2504_high_coverage/data/ERR3243161/NA18874.final.cram
**NA12830**	1000G_2504_high_coverage/data/ERR3243162/NA12830.final.cram
**NA12546**	1000G_2504_high_coverage/data/ERR3243163/NA12546.final.cram
**HG00405**	1000G_2504_high_coverage/additional_698_related/data/ERR398876
**HG00408**	1000G_2504_high_coverage/additional_698_related/data/ERR398876
**HG00418**	1000G_2504_high_coverage/additional_698_related/data/ERR398876
**HG00420**	1000G_2504_high_coverage/additional_698_related/data/ERR398876
**HG00423**	1000G_2504_high_coverage/additional_698_related/data/ERR398876
**HG00427**	1000G_2504_high_coverage/additional_698_related/data/ERR398876
**HG00429**	1000G_2504_high_coverage/additional_698_related/data/ERR398876
**HG00438**	1000G_2504_high_coverage/additional_698_related/data/ERR398876
**HG00444**	1000G_2504_high_coverage/additional_698_related/data/ERR398876
**HG00447**	1000G_2504_high_coverage/additional_698_related/data/ERR398877
**HG00450**	1000G_2504_high_coverage/additional_698_related/data/ERR398877
**HG00453**	1000G_2504_high_coverage/additional_698_related/data/ERR398877
**HG00459**	1000G_2504_high_coverage/additional_698_related/data/ERR398877
**HG00465**	1000G_2504_high_coverage/additional_698_related/data/ERR398877
**HG00474**	1000G_2504_high_coverage/additional_698_related/data/ERR398877
**HG00477**	1000G_2504_high_coverage/additional_698_related/data/ERR398877
**HG00480**	1000G_2504_high_coverage/additional_698_related/data/ERR398877
**HG00501**	1000G_2504_high_coverage/additional_698_related/data/ERR398877
**HG00502**	1000G_2504_high_coverage/additional_698_related/data/ERR398877
**HG00512**	1000G_2504_high_coverage/additional_698_related/data/ERR398878
**HG00514**	1000G_2504_high_coverage/additional_698_related/data/ERR398878
**HG00526**	1000G_2504_high_coverage/additional_698_related/data/ERR398878
**HG00532**	1000G_2504_high_coverage/additional_698_related/data/ERR398878
**HG00535**	1000G_2504_high_coverage/additional_698_related/data/ERR398878
**HG00538**	1000G_2504_high_coverage/additional_698_related/data/ERR398878
**HG00544**	1000G_2504_high_coverage/additional_698_related/data/ERR398878
**HG00552**	1000G_2504_high_coverage/additional_698_related/data/ERR398878
**HG00555**	1000G_2504_high_coverage/additional_698_related/data/ERR398878

**HG00558**	1000G_2504_high_coverage/additional_698_related/data/ERR398878
**HG00561**	1000G_2504_high_coverage/additional_698_related/data/ERR398879
**HG00567**	1000G_2504_high_coverage/additional_698_related/data/ERR398879
**HG00577**	1000G_2504_high_coverage/additional_698_related/data/ERR398879
**HG00578**	1000G_2504_high_coverage/additional_698_related/data/ERR398879
**HG00579**	1000G_2504_high_coverage/additional_698_related/data/ERR398879
**HG00582**	1000G_2504_high_coverage/additional_698_related/data/ERR398879
**HG00585**	1000G_2504_high_coverage/additional_698_related/data/ERR398879
**HG00591**	1000G_2504_high_coverage/additional_698_related/data/ERR398879
**HG00594**	1000G_2504_high_coverage/additional_698_related/data/ERR398879
**HG00597**	1000G_2504_high_coverage/additional_698_related/data/ERR398879
**HG00609**	1000G_2504_high_coverage/additional_698_related/data/ERR398880
**HG00612**	1000G_2504_high_coverage/additional_698_related/data/ERR398880
**HG00615**	1000G_2504_high_coverage/additional_698_related/data/ERR398880
**HG00621**	1000G_2504_high_coverage/additional_698_related/data/ERR398880
**HG00627**	1000G_2504_high_coverage/additional_698_related/data/ERR398880
**HG00630**	1000G_2504_high_coverage/additional_698_related/data/ERR398880
**HG00635**	1000G_2504_high_coverage/additional_698_related/data/ERR398880
**HG00636**	1000G_2504_high_coverage/additional_698_related/data/ERR398880
**HG00639**	1000G_2504_high_coverage/additional_698_related/data/ERR398880
**HG00642**	1000G_2504_high_coverage/additional_698_related/data/ERR398880
**HG00652**	1000G_2504_high_coverage/additional_698_related/data/ERR398881
**HG00655**	1000G_2504_high_coverage/additional_698_related/data/ERR398881
**HG00658**	1000G_2504_high_coverage/additional_698_related/data/ERR398881
**HG00664**	1000G_2504_high_coverage/additional_698_related/data/ERR398881
**HG00673**	1000G_2504_high_coverage/additional_698_related/data/ERR398881
**HG00685**	1000G_2504_high_coverage/additional_698_related/data/ERR398881
**HG00691**	1000G_2504_high_coverage/additional_698_related/data/ERR398881
**HG00694**	1000G_2504_high_coverage/additional_698_related/data/ERR398881
**HG00700**	1000G_2504_high_coverage/additional_698_related/data/ERR398881
**HG00702**	1000G_2504_high_coverage/additional_698_related/data/ERR398881
**HG00703**	1000G_2504_high_coverage/additional_698_related/data/ERR398882
**HG00706**	1000G_2504_high_coverage/additional_698_related/data/ERR398882
**HG00709**	1000G_2504_high_coverage/additional_698_related/data/ERR398882
**HG00733**	1000G_2504_high_coverage/additional_698_related/data/ERR398882
**HG00735**	1000G_2504_high_coverage/additional_698_related/data/ERR398882
**HG00738**	1000G_2504_high_coverage/additional_698_related/data/ERR398882
**HG00741**	1000G_2504_high_coverage/additional_698_related/data/ERR398882
**HG01050**	1000G_2504_high_coverage/additional_698_related/data/ERR398882
**HG01053**	1000G_2504_high_coverage/additional_698_related/data/ERR398882
**HG01056**	1000G_2504_high_coverage/additional_698_related/data/ERR398882
**HG01062**	1000G_2504_high_coverage/additional_698_related/data/ERR398883
**HG01068**	1000G_2504_high_coverage/additional_698_related/data/ERR398883
**HG01071**	1000G_2504_high_coverage/additional_698_related/data/ERR398883
**HG01074**	1000G_2504_high_coverage/additional_698_related/data/ERR398883
**HG01081**	1000G_2504_high_coverage/additional_698_related/data/ERR398883
**HG01084**	1000G_2504_high_coverage/additional_698_related/data/ERR398883
**HG01087**	1000G_2504_high_coverage/additional_698_related/data/ERR398883
**HG01096**	1000G_2504_high_coverage/additional_698_related/data/ERR398883
**HG01099**	1000G_2504_high_coverage/additional_698_related/data/ERR398883
**HG01100**	1000G_2504_high_coverage/additional_698_related/data/ERR398883
**HG01103**	1000G_2504_high_coverage/additional_698_related/data/ERR398884
**HG01106**	1000G_2504_high_coverage/additional_698_related/data/ERR398884
**HG01109**	1000G_2504_high_coverage/additional_698_related/data/ERR398884
**HG01114**	1000G_2504_high_coverage/additional_698_related/data/ERR398884
**HG01126**	1000G_2504_high_coverage/additional_698_related/data/ERR398884
**HG01135**	1000G_2504_high_coverage/additional_698_related/data/ERR398884
**HG01138**	1000G_2504_high_coverage/additional_698_related/data/ERR398884
**HG01141**	1000G_2504_high_coverage/additional_698_related/data/ERR398884
**HG01150**	1000G_2504_high_coverage/additional_698_related/data/ERR398884
**HG01169**	1000G_2504_high_coverage/additional_698_related/data/ERR398884
**HG01172**	1000G_2504_high_coverage/additional_698_related/data/ERR398885
**HG01175**	1000G_2504_high_coverage/additional_698_related/data/ERR398885
**HG01178**	1000G_2504_high_coverage/additional_698_related/data/ERR398885
**HG01184**	1000G_2504_high_coverage/additional_698_related/data/ERR398885

**HG01189**	1000G_2504_high_coverage/additional_698_related/data/ERR398885
**HG01192**	1000G_2504_high_coverage/additional_698_related/data/ERR398885
**HG01199**	1000G_2504_high_coverage/additional_698_related/data/ERR398885
**HG01206**	1000G_2504_high_coverage/additional_698_related/data/ERR398885
**HG01243**	1000G_2504_high_coverage/additional_698_related/data/ERR398885
**HG01249**	1000G_2504_high_coverage/additional_698_related/data/ERR398885
**HG01252**	1000G_2504_high_coverage/additional_698_related/data/ERR398886
**HG01255**	1000G_2504_high_coverage/additional_698_related/data/ERR398886
**HG01258**	1000G_2504_high_coverage/additional_698_related/data/ERR398886
**HG01261**	1000G_2504_high_coverage/additional_698_related/data/ERR398886
**HG01273**	1000G_2504_high_coverage/additional_698_related/data/ERR398886
**HG01274**	1000G_2504_high_coverage/additional_698_related/data/ERR398886
**HG01276**	1000G_2504_high_coverage/additional_698_related/data/ERR398886
**HG01278**	1000G_2504_high_coverage/additional_698_related/data/ERR398886
**HG01279**	1000G_2504_high_coverage/additional_698_related/data/ERR398886
**HG01343**	1000G_2504_high_coverage/additional_698_related/data/ERR398886
**HG01346**	1000G_2504_high_coverage/additional_698_related/data/ERR398887
**HG01347**	1000G_2504_high_coverage/additional_698_related/data/ERR398887
**HG01349**	1000G_2504_high_coverage/additional_698_related/data/ERR398887
**HG01352**	1000G_2504_high_coverage/additional_698_related/data/ERR398887
**HG01355**	1000G_2504_high_coverage/additional_698_related/data/ERR398887
**HG01358**	1000G_2504_high_coverage/additional_698_related/data/ERR398887
**HG01361**	1000G_2504_high_coverage/additional_698_related/data/ERR398887
**HG01367**	1000G_2504_high_coverage/additional_698_related/data/ERR398887
**HG01376**	1000G_2504_high_coverage/additional_698_related/data/ERR398887
**HG01379**	1000G_2504_high_coverage/additional_698_related/data/ERR398887
**HG01385**	1000G_2504_high_coverage/additional_698_related/data/ERR398888
**HG01391**	1000G_2504_high_coverage/additional_698_related/data/ERR398888
**HG01433**	1000G_2504_high_coverage/additional_698_related/data/ERR398888
**HG01439**	1000G_2504_high_coverage/additional_698_related/data/ERR398888
**HG01457**	1000G_2504_high_coverage/additional_698_related/data/ERR398888
**HG01463**	1000G_2504_high_coverage/additional_698_related/data/ERR398888
**HG01466**	1000G_2504_high_coverage/additional_698_related/data/ERR398888
**HG01490**	1000G_2504_high_coverage/additional_698_related/data/ERR398888
**HG01493**	1000G_2504_high_coverage/additional_698_related/data/ERR398888
**HG01496**	1000G_2504_high_coverage/additional_698_related/data/ERR398888
**HG01499**	1000G_2504_high_coverage/additional_698_related/data/ERR398889
**HG01502**	1000G_2504_high_coverage/additional_698_related/data/ERR398889
**HG01505**	1000G_2504_high_coverage/additional_698_related/data/ERR398889
**HG01508**	1000G_2504_high_coverage/additional_698_related/data/ERR398889
**HG01511**	1000G_2504_high_coverage/additional_698_related/data/ERR398889
**HG01514**	1000G_2504_high_coverage/additional_698_related/data/ERR398889
**HG01517**	1000G_2504_high_coverage/additional_698_related/data/ERR398889
**HG01520**	1000G_2504_high_coverage/additional_698_related/data/ERR398889
**HG01523**	1000G_2504_high_coverage/additional_698_related/data/ERR398889
**HG01526**	1000G_2504_high_coverage/additional_698_related/data/ERR398889
**HG01529**	1000G_2504_high_coverage/additional_698_related/data/ERR398890
**HG01532**	1000G_2504_high_coverage/additional_698_related/data/ERR398890
**HG01538**	1000G_2504_high_coverage/additional_698_related/data/ERR398890
**HG01552**	1000G_2504_high_coverage/additional_698_related/data/ERR398890
**HG01567**	1000G_2504_high_coverage/additional_698_related/data/ERR398890
**HG01573**	1000G_2504_high_coverage/additional_698_related/data/ERR398890
**HG01579**	1000G_2504_high_coverage/additional_698_related/data/ERR398890
**HG01601**	1000G_2504_high_coverage/additional_698_related/data/ERR398890
**HG01604**	1000G_2504_high_coverage/additional_698_related/data/ERR398890
**HG01609**	1000G_2504_high_coverage/additional_698_related/data/ERR398890
**HG01611**	1000G_2504_high_coverage/additional_698_related/data/ERR398891
**HG01614**	1000G_2504_high_coverage/additional_698_related/data/ERR398891
**HG01616**	1000G_2504_high_coverage/additional_698_related/data/ERR398891
**HG01621**	1000G_2504_high_coverage/additional_698_related/data/ERR398891
**HG01622**	1000G_2504_high_coverage/additional_698_related/data/ERR398891
**HG01627**	1000G_2504_high_coverage/additional_698_related/data/ERR398891
**HG01629**	1000G_2504_high_coverage/additional_698_related/data/ERR398891
**HG01633**	1000G_2504_high_coverage/additional_698_related/data/ERR398891
**HG01667**	1000G_2504_high_coverage/additional_698_related/data/ERR398891

**HG01671**	1000G_2504_high_coverage/additional_698_related/data/ERR398891
**HG01674**	1000G_2504_high_coverage/additional_698_related/data/ERR398892
**HG01677**	1000G_2504_high_coverage/additional_698_related/data/ERR398892
**HG01681**	1000G_2504_high_coverage/additional_698_related/data/ERR398892
**HG01683**	1000G_2504_high_coverage/additional_698_related/data/ERR398892
**HG01687**	1000G_2504_high_coverage/additional_698_related/data/ERR398892
**HG01696**	1000G_2504_high_coverage/additional_698_related/data/ERR398892
**HG01698**	1000G_2504_high_coverage/additional_698_related/data/ERR398892
**HG01701**	1000G_2504_high_coverage/additional_698_related/data/ERR398892
**HG01703**	1000G_2504_high_coverage/additional_698_related/data/ERR398892
**HG01706**	1000G_2504_high_coverage/additional_698_related/data/ERR398892
**HG01711**	1000G_2504_high_coverage/additional_698_related/data/ERR398893
**HG01748**	1000G_2504_high_coverage/additional_698_related/data/ERR398893
**HG01755**	1000G_2504_high_coverage/additional_698_related/data/ERR398893
**HG01763**	1000G_2504_high_coverage/additional_698_related/data/ERR398893
**HG01772**	1000G_2504_high_coverage/additional_698_related/data/ERR398893
**HG01774**	1000G_2504_high_coverage/additional_698_related/data/ERR398893
**HG01778**	1000G_2504_high_coverage/additional_698_related/data/ERR398893
**HG01780**	1000G_2504_high_coverage/additional_698_related/data/ERR398893
**HG01782**	1000G_2504_high_coverage/additional_698_related/data/ERR398893
**HG01881**	1000G_2504_high_coverage/additional_698_related/data/ERR398893
**HG01884**	1000G_2504_high_coverage/additional_698_related/data/ERR398894
**HG01887**	1000G_2504_high_coverage/additional_698_related/data/ERR398894
**HG01888**	1000G_2504_high_coverage/additional_698_related/data/ERR398894
**HG01891**	1000G_2504_high_coverage/additional_698_related/data/ERR398894
**HG01895**	1000G_2504_high_coverage/additional_698_related/data/ERR398894
**HG01897**	1000G_2504_high_coverage/additional_698_related/data/ERR398894
**HG01898**	1000G_2504_high_coverage/additional_698_related/data/ERR398894
**HG01916**	1000G_2504_high_coverage/additional_698_related/data/ERR398894
**HG01919**	1000G_2504_high_coverage/additional_698_related/data/ERR398894
**HG01922**	1000G_2504_high_coverage/additional_698_related/data/ERR398894
**HG01925**	1000G_2504_high_coverage/additional_698_related/data/ERR398895
**HG01928**	1000G_2504_high_coverage/additional_698_related/data/ERR398895
**HG01934**	1000G_2504_high_coverage/additional_698_related/data/ERR398895
**HG01937**	1000G_2504_high_coverage/additional_698_related/data/ERR398895
**HG01940**	1000G_2504_high_coverage/additional_698_related/data/ERR398895
**HG01943**	1000G_2504_high_coverage/additional_698_related/data/ERR398895
**HG01946**	1000G_2504_high_coverage/additional_698_related/data/ERR398895
**HG01949**	1000G_2504_high_coverage/additional_698_related/data/ERR398895
**HG01952**	1000G_2504_high_coverage/additional_698_related/data/ERR398895
**HG01955**	1000G_2504_high_coverage/additional_698_related/data/ERR398895
**HG01959**	1000G_2504_high_coverage/additional_698_related/data/ERR398896
**HG01960**	1000G_2504_high_coverage/additional_698_related/data/ERR398896
**HG01969**	1000G_2504_high_coverage/additional_698_related/data/ERR398896
**HG01972**	1000G_2504_high_coverage/additional_698_related/data/ERR398896
**HG01975**	1000G_2504_high_coverage/additional_698_related/data/ERR398896
**HG01978**	1000G_2504_high_coverage/additional_698_related/data/ERR398896
**HG01981**	1000G_2504_high_coverage/additional_698_related/data/ERR398896
**HG01983**	1000G_2504_high_coverage/additional_698_related/data/ERR398896
**HG01984**	1000G_2504_high_coverage/additional_698_related/data/ERR398896
**HG01987**	1000G_2504_high_coverage/additional_698_related/data/ERR398896
**HG01993**	1000G_2504_high_coverage/additional_698_related/data/ERR398897
**HG01998**	1000G_2504_high_coverage/additional_698_related/data/ERR398897
**HG02004**	1000G_2504_high_coverage/additional_698_related/data/ERR398897
**HG02011**	1000G_2504_high_coverage/additional_698_related/data/ERR398897
**HG02015**	1000G_2504_high_coverage/additional_698_related/data/ERR398897
**HG02018**	1000G_2504_high_coverage/additional_698_related/data/ERR398897
**HG02024**	1000G_2504_high_coverage/additional_698_related/data/ERR398897
**HG02027**	1000G_2504_high_coverage/additional_698_related/data/ERR398897
**HG02030**	1000G_2504_high_coverage/additional_698_related/data/ERR398897
**HG02055**	1000G_2504_high_coverage/additional_698_related/data/ERR398897
**HG02056**	1000G_2504_high_coverage/additional_698_related/data/ERR398898
**HG02059**	1000G_2504_high_coverage/additional_698_related/data/ERR398898
**HG02068**	1000G_2504_high_coverage/additional_698_related/data/ERR398898
**HG02071**	1000G_2504_high_coverage/additional_698_related/data/ERR398898

**HG02074**	1000G_2504_high_coverage/additional_698_related/data/ERR3988898
**HG02077**	1000G_2504_high_coverage/additional_698_related/data/ERR3988898
**HG02080**	1000G_2504_high_coverage/additional_698_related/data/ERR3988898
**HG02083**	1000G_2504_high_coverage/additional_698_related/data/ERR3988898
**HG02091**	1000G_2504_high_coverage/additional_698_related/data/ERR3988898
**HG02106**	1000G_2504_high_coverage/additional_698_related/data/ERR3988898
**HG02120**	1000G_2504_high_coverage/additional_698_related/data/ERR3988899
**HG02126**	1000G_2504_high_coverage/additional_698_related/data/ERR3988899
**HG02129**	1000G_2504_high_coverage/additional_698_related/data/ERR3988899
**HG02132**	1000G_2504_high_coverage/additional_698_related/data/ERR3988899
**HG02135**	1000G_2504_high_coverage/additional_698_related/data/ERR3988899
**HG02145**	1000G_2504_high_coverage/additional_698_related/data/ERR3988899
**HG02148**	1000G_2504_high_coverage/additional_698_related/data/ERR3988899
**HG02222**	1000G_2504_high_coverage/additional_698_related/data/ERR3988899
**HG02225**	1000G_2504_high_coverage/additional_698_related/data/ERR3988899
**HG02229**	1000G_2504_high_coverage/additional_698_related/data/ERR3988899
**HG02234**	1000G_2504_high_coverage/additional_698_related/data/ERR3989000
**HG02237**	1000G_2504_high_coverage/additional_698_related/data/ERR3989000
**HG02240**	1000G_2504_high_coverage/additional_698_related/data/ERR3989000
**HG02257**	1000G_2504_high_coverage/additional_698_related/data/ERR3989000
**HG02258**	1000G_2504_high_coverage/additional_698_related/data/ERR3989000
**HG02261**	1000G_2504_high_coverage/additional_698_related/data/ERR3989000
**HG02273**	1000G_2504_high_coverage/additional_698_related/data/ERR3989000
**HG02279**	1000G_2504_high_coverage/additional_698_related/data/ERR3989000
**HG02280**	1000G_2504_high_coverage/additional_698_related/data/ERR3989000
**HG02287**	1000G_2504_high_coverage/additional_698_related/data/ERR3989000
**HG02293**	1000G_2504_high_coverage/additional_698_related/data/ERR3989011
**HG02300**	1000G_2504_high_coverage/additional_698_related/data/ERR3989011
**HG02302**	1000G_2504_high_coverage/additional_698_related/data/ERR3989011
**HG02303**	1000G_2504_high_coverage/additional_698_related/data/ERR3989011
**HG02316**	1000G_2504_high_coverage/additional_698_related/data/ERR3989011
**HG02321**	1000G_2504_high_coverage/additional_698_related/data/ERR3989011
**HG02451**	1000G_2504_high_coverage/additional_698_related/data/ERR3989011
**HG02463**	1000G_2504_high_coverage/additional_698_related/data/ERR3989011
**HG02466**	1000G_2504_high_coverage/additional_698_related/data/ERR3989011
**HG02492**	1000G_2504_high_coverage/additional_698_related/data/ERR3989011
**HG02495**	1000G_2504_high_coverage/additional_698_related/data/ERR3989022
**HG02514**	1000G_2504_high_coverage/additional_698_related/data/ERR3989022
**HG02523**	1000G_2504_high_coverage/additional_698_related/data/ERR3989022
**HG02524**	1000G_2504_high_coverage/additional_698_related/data/ERR3989022
**HG02525**	1000G_2504_high_coverage/additional_698_related/data/ERR3989022
**HG02526**	1000G_2504_high_coverage/additional_698_related/data/ERR3989022
**HG02563**	1000G_2504_high_coverage/additional_698_related/data/ERR3989022
**HG02569**	1000G_2504_high_coverage/additional_698_related/data/ERR3989022
**HG02572**	1000G_2504_high_coverage/additional_698_related/data/ERR3989022
**HG02575**	1000G_2504_high_coverage/additional_698_related/data/ERR3989022
**HG02584**	1000G_2504_high_coverage/additional_698_related/data/ERR3989033
**HG02587**	1000G_2504_high_coverage/additional_698_related/data/ERR3989033
**HG02590**	1000G_2504_high_coverage/additional_698_related/data/ERR3989033
**HG02596**	1000G_2504_high_coverage/additional_698_related/data/ERR3989033
**HG02602**	1000G_2504_high_coverage/additional_698_related/data/ERR3989033
**HG02605**	1000G_2504_high_coverage/additional_698_related/data/ERR3989033
**HG02612**	1000G_2504_high_coverage/additional_698_related/data/ERR3989033
**HG02615**	1000G_2504_high_coverage/additional_698_related/data/ERR3989033
**HG02622**	1000G_2504_high_coverage/additional_698_related/data/ERR3989033
**HG02625**	1000G_2504_high_coverage/additional_698_related/data/ERR3989033
**HG02630**	1000G_2504_high_coverage/additional_698_related/data/ERR3989044
**HG02636**	1000G_2504_high_coverage/additional_698_related/data/ERR3989044
**HG02644**	1000G_2504_high_coverage/additional_698_related/data/ERR3989044
**HG02647**	1000G_2504_high_coverage/additional_698_related/data/ERR3989044
**HG02650**	1000G_2504_high_coverage/additional_698_related/data/ERR3989044
**HG02653**	1000G_2504_high_coverage/additional_698_related/data/ERR3989044
**HG02656**	1000G_2504_high_coverage/additional_698_related/data/ERR3989044
**HG02659**	1000G_2504_high_coverage/additional_698_related/data/ERR3989044
**HG02662**	1000G_2504_high_coverage/additional_698_related/data/ERR3989044

**HG02668**	1000G_2504_high_coverage/additional_698_related/data/ERR398904
**HG02677**	1000G_2504_high_coverage/additional_698_related/data/ERR398905
**HG02680**	1000G_2504_high_coverage/additional_698_related/data/ERR398905
**HG02683**	1000G_2504_high_coverage/additional_698_related/data/ERR398905
**HG02686**	1000G_2504_high_coverage/additional_698_related/data/ERR398905
**HG02689**	1000G_2504_high_coverage/additional_698_related/data/ERR398905
**HG02692**	1000G_2504_high_coverage/additional_698_related/data/ERR398905
**HG02698**	1000G_2504_high_coverage/additional_698_related/data/ERR398905
**HG02701**	1000G_2504_high_coverage/additional_698_related/data/ERR398905
**HG02704**	1000G_2504_high_coverage/additional_698_related/data/ERR398905
**HG02717**	1000G_2504_high_coverage/additional_698_related/data/ERR398905
**HG02723**	1000G_2504_high_coverage/additional_698_related/data/ERR398906
**HG02726**	1000G_2504_high_coverage/additional_698_related/data/ERR398906
**HG02729**	1000G_2504_high_coverage/additional_698_related/data/ERR398906
**HG02735**	1000G_2504_high_coverage/additional_698_related/data/ERR398906
**HG02738**	1000G_2504_high_coverage/additional_698_related/data/ERR398906
**HG02761**	1000G_2504_high_coverage/additional_698_related/data/ERR398906
**HG02762**	1000G_2504_high_coverage/additional_698_related/data/ERR398906
**HG02764**	1000G_2504_high_coverage/additional_698_related/data/ERR398906
**HG02770**	1000G_2504_high_coverage/additional_698_related/data/ERR398906
**HG02773**	1000G_2504_high_coverage/additional_698_related/data/ERR398906
**HG02776**	1000G_2504_high_coverage/additional_698_related/data/ERR398907
**HG02785**	1000G_2504_high_coverage/additional_698_related/data/ERR398907
**HG02788**	1000G_2504_high_coverage/additional_698_related/data/ERR398907
**HG02791**	1000G_2504_high_coverage/additional_698_related/data/ERR398907
**HG02794**	1000G_2504_high_coverage/additional_698_related/data/ERR398907
**HG02800**	1000G_2504_high_coverage/additional_698_related/data/ERR398907
**HG02806**	1000G_2504_high_coverage/additional_698_related/data/ERR398907
**HG02809**	1000G_2504_high_coverage/additional_698_related/data/ERR398907
**HG02812**	1000G_2504_high_coverage/additional_698_related/data/ERR398907
**HG02815**	1000G_2504_high_coverage/additional_698_related/data/ERR398907
**HG02818**	1000G_2504_high_coverage/additional_698_related/data/ERR398908
**HG02821**	1000G_2504_high_coverage/additional_698_related/data/ERR398908
**HG02838**	1000G_2504_high_coverage/additional_698_related/data/ERR398908
**HG02841**	1000G_2504_high_coverage/additional_698_related/data/ERR398908
**HG02853**	1000G_2504_high_coverage/additional_698_related/data/ERR398908
**HG02856**	1000G_2504_high_coverage/additional_698_related/data/ERR398908
**HG02862**	1000G_2504_high_coverage/additional_698_related/data/ERR398908
**HG02869**	1000G_2504_high_coverage/additional_698_related/data/ERR398908
**HG02871**	1000G_2504_high_coverage/additional_698_related/data/ERR398908
**HG02880**	1000G_2504_high_coverage/additional_698_related/data/ERR398908
**HG02883**	1000G_2504_high_coverage/additional_698_related/data/ERR398909
**HG02886**	1000G_2504_high_coverage/additional_698_related/data/ERR398909
**HG02889**	1000G_2504_high_coverage/additional_698_related/data/ERR398909
**HG02892**	1000G_2504_high_coverage/additional_698_related/data/ERR398909
**HG02897**	1000G_2504_high_coverage/additional_698_related/data/ERR398909
**HG02924**	1000G_2504_high_coverage/additional_698_related/data/ERR398909
**HG02945**	1000G_2504_high_coverage/additional_698_related/data/ERR398909
**HG02948**	1000G_2504_high_coverage/additional_698_related/data/ERR398909
**HG02954**	1000G_2504_high_coverage/additional_698_related/data/ERR398909
**HG02964**	1000G_2504_high_coverage/additional_698_related/data/ERR398909
**HG02965**	1000G_2504_high_coverage/additional_698_related/data/ERR398910
**HG02966**	1000G_2504_high_coverage/additional_698_related/data/ERR398910
**HG02972**	1000G_2504_high_coverage/additional_698_related/data/ERR398910
**HG02975**	1000G_2504_high_coverage/additional_698_related/data/ERR398910
**HG02978**	1000G_2504_high_coverage/additional_698_related/data/ERR398910
**HG02980**	1000G_2504_high_coverage/additional_698_related/data/ERR398910
**HG02984**	1000G_2504_high_coverage/additional_698_related/data/ERR398910
**HG03008**	1000G_2504_high_coverage/additional_698_related/data/ERR398910
**HG03017**	1000G_2504_high_coverage/additional_698_related/data/ERR398910
**HG03026**	1000G_2504_high_coverage/additional_698_related/data/ERR398910
**HG03029**	1000G_2504_high_coverage/additional_698_related/data/ERR398911
**HG03033**	1000G_2504_high_coverage/additional_698_related/data/ERR398911
**HG03034**	1000G_2504_high_coverage/additional_698_related/data/ERR398911
**HG03035**	1000G_2504_high_coverage/additional_698_related/data/ERR398911

**HG03041**	1000G_2504_high_coverage/additional_698_related/data/ERR398911
**HG03047**	1000G_2504_high_coverage/additional_698_related/data/ERR398911
**HG03050**	1000G_2504_high_coverage/additional_698_related/data/ERR398911
**HG03056**	1000G_2504_high_coverage/additional_698_related/data/ERR398911
**HG03065**	1000G_2504_high_coverage/additional_698_related/data/ERR398911
**HG03098**	1000G_2504_high_coverage/additional_698_related/data/ERR398911
**HG03101**	1000G_2504_high_coverage/additional_698_related/data/ERR398912
**HG03110**	1000G_2504_high_coverage/additional_698_related/data/ERR398912
**HG03113**	1000G_2504_high_coverage/additional_698_related/data/ERR398912
**HG03116**	1000G_2504_high_coverage/additional_698_related/data/ERR398912
**HG03119**	1000G_2504_high_coverage/additional_698_related/data/ERR398912
**HG03122**	1000G_2504_high_coverage/additional_698_related/data/ERR398912
**HG03125**	1000G_2504_high_coverage/additional_698_related/data/ERR398912
**HG03128**	1000G_2504_high_coverage/additional_698_related/data/ERR398912
**HG03131**	1000G_2504_high_coverage/additional_698_related/data/ERR398912
**HG03134**	1000G_2504_high_coverage/additional_698_related/data/ERR398912
**HG03137**	1000G_2504_high_coverage/additional_698_related/data/ERR398913
**HG03161**	1000G_2504_high_coverage/additional_698_related/data/ERR398913
**HG03164**	1000G_2504_high_coverage/additional_698_related/data/ERR398913
**HG03170**	1000G_2504_high_coverage/additional_698_related/data/ERR398913
**HG03191**	1000G_2504_high_coverage/additional_698_related/data/ERR398913
**HG03197**	1000G_2504_high_coverage/additional_698_related/data/ERR398913
**HG03200**	1000G_2504_high_coverage/additional_698_related/data/ERR398913
**HG03230**	1000G_2504_high_coverage/additional_698_related/data/ERR398913
**HG03236**	1000G_2504_high_coverage/additional_698_related/data/ERR398913
**HG03239**	1000G_2504_high_coverage/additional_698_related/data/ERR398913
**HG03242**	1000G_2504_high_coverage/additional_698_related/data/ERR398914
**HG03248**	1000G_2504_high_coverage/additional_698_related/data/ERR398914
**HG03249**	1000G_2504_high_coverage/additional_698_related/data/ERR398914
**HG03250**	1000G_2504_high_coverage/additional_698_related/data/ERR398914
**HG03251**	1000G_2504_high_coverage/additional_698_related/data/ERR398914
**HG03260**	1000G_2504_high_coverage/additional_698_related/data/ERR398914
**HG03269**	1000G_2504_high_coverage/additional_698_related/data/ERR398914
**HG03272**	1000G_2504_high_coverage/additional_698_related/data/ERR398914
**HG03296**	1000G_2504_high_coverage/additional_698_related/data/ERR398914
**HG03299**	1000G_2504_high_coverage/additional_698_related/data/ERR398914
**HG03302**	1000G_2504_high_coverage/additional_698_related/data/ERR398915
**HG03305**	1000G_2504_high_coverage/additional_698_related/data/ERR398915
**HG03306**	1000G_2504_high_coverage/additional_698_related/data/ERR398915
**HG03307**	1000G_2504_high_coverage/additional_698_related/data/ERR398915
**HG03308**	1000G_2504_high_coverage/additional_698_related/data/ERR398915
**HG03309**	1000G_2504_high_coverage/additional_698_related/data/ERR398915
**HG03310**	1000G_2504_high_coverage/additional_698_related/data/ERR398915
**HG03312**	1000G_2504_high_coverage/additional_698_related/data/ERR398915
**HG03314**	1000G_2504_high_coverage/additional_698_related/data/ERR398915
**HG03344**	1000G_2504_high_coverage/additional_698_related/data/ERR398915
**HG03350**	1000G_2504_high_coverage/additional_698_related/data/ERR398916
**HG03368**	1000G_2504_high_coverage/additional_698_related/data/ERR398916
**HG03371**	1000G_2504_high_coverage/additional_698_related/data/ERR398916
**HG03373**	1000G_2504_high_coverage/additional_698_related/data/ERR398916
**HG03374**	1000G_2504_high_coverage/additional_698_related/data/ERR398916
**HG03438**	1000G_2504_high_coverage/additional_698_related/data/ERR398916
**HG03453**	1000G_2504_high_coverage/additional_698_related/data/ERR398916
**HG03454**	1000G_2504_high_coverage/additional_698_related/data/ERR398916
**HG03456**	1000G_2504_high_coverage/additional_698_related/data/ERR398916
**HG03480**	1000G_2504_high_coverage/additional_698_related/data/ERR398916
**HG03486**	1000G_2504_high_coverage/additional_698_related/data/ERR398917
**HG03487**	1000G_2504_high_coverage/additional_698_related/data/ERR398917
**HG03489**	1000G_2504_high_coverage/additional_698_related/data/ERR398917
**HG03492**	1000G_2504_high_coverage/additional_698_related/data/ERR398917
**HG03516**	1000G_2504_high_coverage/additional_698_related/data/ERR398917
**HG03519**	1000G_2504_high_coverage/additional_698_related/data/ERR398917
**HG03522**	1000G_2504_high_coverage/additional_698_related/data/ERR398917
**HG03540**	1000G_2504_high_coverage/additional_698_related/data/ERR398917
**HG03574**	1000G_2504_high_coverage/additional_698_related/data/ERR398917

**HG03576**	1000G_2504_high_coverage/additional_698_related/data/ERR398917
**HG03579**	1000G_2504_high_coverage/additional_698_related/data/ERR398918
**HG03582**	1000G_2504_high_coverage/additional_698_related/data/ERR398918
**HG03584**	1000G_2504_high_coverage/additional_698_related/data/ERR398918
**HG03596**	1000G_2504_high_coverage/additional_698_related/data/ERR398918
**HG03605**	1000G_2504_high_coverage/additional_698_related/data/ERR398918
**HG03606**	1000G_2504_high_coverage/additional_698_related/data/ERR398918
**HG03617**	1000G_2504_high_coverage/additional_698_related/data/ERR398918
**HG03618**	1000G_2504_high_coverage/additional_698_related/data/ERR398918
**HG03620**	1000G_2504_high_coverage/additional_698_related/data/ERR398918
**HG03626**	1000G_2504_high_coverage/additional_698_related/data/ERR398918
**HG03633**	1000G_2504_high_coverage/additional_698_related/data/ERR398919
**HG03635**	1000G_2504_high_coverage/additional_698_related/data/ERR398919
**HG03639**	1000G_2504_high_coverage/additional_698_related/data/ERR398919
**HG03641**	1000G_2504_high_coverage/additional_698_related/data/ERR398919
**HG03650**	1000G_2504_high_coverage/additional_698_related/data/ERR398919
**HG03651**	1000G_2504_high_coverage/additional_698_related/data/ERR398919
**HG03654**	1000G_2504_high_coverage/additional_698_related/data/ERR398919
**HG03669**	1000G_2504_high_coverage/additional_698_related/data/ERR398919
**HG03682**	1000G_2504_high_coverage/additional_698_related/data/ERR398919
**HG03683**	1000G_2504_high_coverage/additional_698_related/data/ERR398919
**HG03688**	1000G_2504_high_coverage/additional_698_related/data/ERR398920
**HG03699**	1000G_2504_high_coverage/additional_698_related/data/ERR398920
**HG03700**	1000G_2504_high_coverage/additional_698_related/data/ERR398920
**HG03701**	1000G_2504_high_coverage/additional_698_related/data/ERR398920
**HG03704**	1000G_2504_high_coverage/additional_698_related/data/ERR398920
**HG03707**	1000G_2504_high_coverage/additional_698_related/data/ERR398920
**HG03710**	1000G_2504_high_coverage/additional_698_related/data/ERR398920
**HG03719**	1000G_2504_high_coverage/additional_698_related/data/ERR398920
**HG03721**	1000G_2504_high_coverage/additional_698_related/data/ERR398920
**HG03725**	1000G_2504_high_coverage/additional_698_related/data/ERR398920
**HG03732**	1000G_2504_high_coverage/additional_698_related/data/ERR398921
**HG03761**	1000G_2504_high_coverage/additional_698_related/data/ERR398921
**HG03763**	1000G_2504_high_coverage/additional_698_related/data/ERR398921
**HG03794**	1000G_2504_high_coverage/additional_698_related/data/ERR398921
**HG03795**	1000G_2504_high_coverage/additional_698_related/data/ERR398921
**HG03797**	1000G_2504_high_coverage/additional_698_related/data/ERR398921
**HG03798**	1000G_2504_high_coverage/additional_698_related/data/ERR398921
**HG03799**	1000G_2504_high_coverage/additional_698_related/data/ERR398921
**HG03801**	1000G_2504_high_coverage/additional_698_related/data/ERR398921
**HG03804**	1000G_2504_high_coverage/additional_698_related/data/ERR398921
**HG03806**	1000G_2504_high_coverage/additional_698_related/data/ERR398922
**HG03807**	1000G_2504_high_coverage/additional_698_related/data/ERR398922
**HG03816**	1000G_2504_high_coverage/additional_698_related/data/ERR398922
**HG03825**	1000G_2504_high_coverage/additional_698_related/data/ERR398922
**HG03831**	1000G_2504_high_coverage/additional_698_related/data/ERR398922
**HG03834**	1000G_2504_high_coverage/additional_698_related/data/ERR398922
**HG03904**	1000G_2504_high_coverage/additional_698_related/data/ERR398922
**HG03906**	1000G_2504_high_coverage/additional_698_related/data/ERR398922
**HG03909**	1000G_2504_high_coverage/additional_698_related/data/ERR398922
**HG03915**	1000G_2504_high_coverage/additional_698_related/data/ERR398922
**HG03927**	1000G_2504_high_coverage/additional_698_related/data/ERR398923
**HG03929**	1000G_2504_high_coverage/additional_698_related/data/ERR398923
**HG03930**	1000G_2504_high_coverage/additional_698_related/data/ERR398923
**HG03942**	1000G_2504_high_coverage/additional_698_related/data/ERR398923
**HG03992**	1000G_2504_high_coverage/additional_698_related/data/ERR398923
**HG04036**	1000G_2504_high_coverage/additional_698_related/data/ERR398923
**HG04115**	1000G_2504_high_coverage/additional_698_related/data/ERR398923
**HG04122**	1000G_2504_high_coverage/additional_698_related/data/ERR398923
**HG04127**	1000G_2504_high_coverage/additional_698_related/data/ERR398923
**HG04132**	1000G_2504_high_coverage/additional_698_related/data/ERR398923
**HG04133**	1000G_2504_high_coverage/additional_698_related/data/ERR398924
**HG04135**	1000G_2504_high_coverage/additional_698_related/data/ERR398924
**HG04136**	1000G_2504_high_coverage/additional_698_related/data/ERR398924
**HG04142**	1000G_2504_high_coverage/additional_698_related/data/ERR398924

**HG04147**	1000G_2504_high_coverage/additional_698_related/data/ERR398924
**HG04148**	1000G_2504_high_coverage/additional_698_related/data/ERR398924
**HG04149**	1000G_2504_high_coverage/additional_698_related/data/ERR398924
**HG04150**	1000G_2504_high_coverage/additional_698_related/data/ERR398924
**HG04151**	1000G_2504_high_coverage/additional_698_related/data/ERR398924
**HG04157**	1000G_2504_high_coverage/additional_698_related/data/ERR398924
**HG04160**	1000G_2504_high_coverage/additional_698_related/data/ERR398925
**HG04174**	1000G_2504_high_coverage/additional_698_related/data/ERR398925
**HG04175**	1000G_2504_high_coverage/additional_698_related/data/ERR398925
**HG04184**	1000G_2504_high_coverage/additional_698_related/data/ERR398925
**HG04187**	1000G_2504_high_coverage/additional_698_related/data/ERR398925
**HG04191**	1000G_2504_high_coverage/additional_698_related/data/ERR398925
**HG04192**	1000G_2504_high_coverage/additional_698_related/data/ERR398925
**HG04193**	1000G_2504_high_coverage/additional_698_related/data/ERR398925
**HG04199**	1000G_2504_high_coverage/additional_698_related/data/ERR398925
**HG04204**	1000G_2504_high_coverage/additional_698_related/data/ERR398925
**HG04215**	1000G_2504_high_coverage/additional_698_related/data/ERR398926
**HG04217**	1000G_2504_high_coverage/additional_698_related/data/ERR398926
**HG04228**	1000G_2504_high_coverage/additional_698_related/data/ERR398926
**NA06991**	1000G_2504_high_coverage/additional_698_related/data/ERR398926
**NA06993**	1000G_2504_high_coverage/additional_698_related/data/ERR398926
**NA06995**	1000G_2504_high_coverage/additional_698_related/data/ERR398926
**NA06997**	1000G_2504_high_coverage/additional_698_related/data/ERR398926
**NA07014**	1000G_2504_high_coverage/additional_698_related/data/ERR398926
**NA07019**	1000G_2504_high_coverage/additional_698_related/data/ERR398926
**NA07022**	1000G_2504_high_coverage/additional_698_related/data/ERR398926
**NA07029**	1000G_2504_high_coverage/additional_698_related/data/ERR398927
**NA07031**	1000G_2504_high_coverage/additional_698_related/data/ERR398927
**NA07034**	1000G_2504_high_coverage/additional_698_related/data/ERR398927
**NA07045**	1000G_2504_high_coverage/additional_698_related/data/ERR398927
**NA07055**	1000G_2504_high_coverage/additional_698_related/data/ERR398927
**NA07345**	1000G_2504_high_coverage/additional_698_related/data/ERR398927
**NA07346**	1000G_2504_high_coverage/additional_698_related/data/ERR398927
**NA07348**	1000G_2504_high_coverage/additional_698_related/data/ERR398927
**NA07349**	1000G_2504_high_coverage/additional_698_related/data/ERR398927
**NA07435**	1000G_2504_high_coverage/additional_698_related/data/ERR398927
**NA10830**	1000G_2504_high_coverage/additional_698_related/data/ERR398928
**NA10831**	1000G_2504_high_coverage/additional_698_related/data/ERR398928
**NA10835**	1000G_2504_high_coverage/additional_698_related/data/ERR398928
**NA10836**	1000G_2504_high_coverage/additional_698_related/data/ERR398928
**NA10837**	1000G_2504_high_coverage/additional_698_related/data/ERR398928
**NA10838**	1000G_2504_high_coverage/additional_698_related/data/ERR398928
**NA10839**	1000G_2504_high_coverage/additional_698_related/data/ERR398928
**NA10840**	1000G_2504_high_coverage/additional_698_related/data/ERR398928
**NA10842**	1000G_2504_high_coverage/additional_698_related/data/ERR398928
**NA10843**	1000G_2504_high_coverage/additional_698_related/data/ERR398928
**NA10845**	1000G_2504_high_coverage/additional_698_related/data/ERR398929
**NA10846**	1000G_2504_high_coverage/additional_698_related/data/ERR398929
**NA10852**	1000G_2504_high_coverage/additional_698_related/data/ERR398929
**NA10854**	1000G_2504_high_coverage/additional_698_related/data/ERR398929
**NA10855**	1000G_2504_high_coverage/additional_698_related/data/ERR398929
**NA10856**	1000G_2504_high_coverage/additional_698_related/data/ERR398929
**NA10857**	1000G_2504_high_coverage/additional_698_related/data/ERR398929
**NA10859**	1000G_2504_high_coverage/additional_698_related/data/ERR398929
**NA10860**	1000G_2504_high_coverage/additional_698_related/data/ERR398929
**NA10861**	1000G_2504_high_coverage/additional_698_related/data/ERR398929
**NA10863**	1000G_2504_high_coverage/additional_698_related/data/ERR398930
**NA10864**	1000G_2504_high_coverage/additional_698_related/data/ERR398930
**NA10865**	1000G_2504_high_coverage/additional_698_related/data/ERR398930
**NA11839**	1000G_2504_high_coverage/additional_698_related/data/ERR398930
**NA11882**	1000G_2504_high_coverage/additional_698_related/data/ERR398930
**NA11891**	1000G_2504_high_coverage/additional_698_related/data/ERR398930
**NA11917**	1000G_2504_high_coverage/additional_698_related/data/ERR398930
**NA11993**	1000G_2504_high_coverage/additional_698_related/data/ERR398930
**NA12056**	1000G_2504_high_coverage/additional_698_related/data/ERR398930

**NA12057**	1000G_2504_high_coverage/additional_698_related/data/ERR398930
**NA12145**	1000G_2504_high_coverage/additional_698_related/data/ERR398931
**NA12146**	1000G_2504_high_coverage/additional_698_related/data/ERR398931
**NA12236**	1000G_2504_high_coverage/additional_698_related/data/ERR398931
**NA12239**	1000G_2504_high_coverage/additional_698_related/data/ERR398931
**NA12248**	1000G_2504_high_coverage/additional_698_related/data/ERR398931
**NA12264**	1000G_2504_high_coverage/additional_698_related/data/ERR398931
**NA12274**	1000G_2504_high_coverage/additional_698_related/data/ERR398931
**NA12329**	1000G_2504_high_coverage/additional_698_related/data/ERR398931
**NA12335**	1000G_2504_high_coverage/additional_698_related/data/ERR398931
**NA12336**	1000G_2504_high_coverage/additional_698_related/data/ERR398931
**NA12343**	1000G_2504_high_coverage/additional_698_related/data/ERR398932
**NA12344**	1000G_2504_high_coverage/additional_698_related/data/ERR398932
**NA12376**	1000G_2504_high_coverage/additional_698_related/data/ERR398932
**NA12386**	1000G_2504_high_coverage/additional_698_related/data/ERR398932
**NA12485**	1000G_2504_high_coverage/additional_698_related/data/ERR398932
**NA12707**	1000G_2504_high_coverage/additional_698_related/data/ERR398932
**NA12739**	1000G_2504_high_coverage/additional_698_related/data/ERR398932
**NA12740**	1000G_2504_high_coverage/additional_698_related/data/ERR398932
**NA12752**	1000G_2504_high_coverage/additional_698_related/data/ERR398932
**NA12753**	1000G_2504_high_coverage/additional_698_related/data/ERR398932
**NA12766**	1000G_2504_high_coverage/additional_698_related/data/ERR398933
**NA12767**	1000G_2504_high_coverage/additional_698_related/data/ERR398933
**NA12801**	1000G_2504_high_coverage/additional_698_related/data/ERR398933
**NA12802**	1000G_2504_high_coverage/additional_698_related/data/ERR398933
**NA12817**	1000G_2504_high_coverage/additional_698_related/data/ERR398933
**NA12818**	1000G_2504_high_coverage/additional_698_related/data/ERR398933
**NA12832**	1000G_2504_high_coverage/additional_698_related/data/ERR398933
**NA12864**	1000G_2504_high_coverage/additional_698_related/data/ERR398933
**NA12865**	1000G_2504_high_coverage/additional_698_related/data/ERR398933
**NA12875**	1000G_2504_high_coverage/additional_698_related/data/ERR398933
**NA12877**	1000G_2504_high_coverage/additional_698_related/data/ERR398934
**NA12891**	1000G_2504_high_coverage/additional_698_related/data/ERR398934
**NA12892**	1000G_2504_high_coverage/additional_698_related/data/ERR398934
**NA18484**	1000G_2504_high_coverage/additional_698_related/data/ERR398934
**NA18485**	1000G_2504_high_coverage/additional_698_related/data/ERR398934
**NA18487**	1000G_2504_high_coverage/additional_698_related/data/ERR398934
**NA18497**	1000G_2504_high_coverage/additional_698_related/data/ERR398934
**NA18500**	1000G_2504_high_coverage/additional_698_related/data/ERR398934
**NA18503**	1000G_2504_high_coverage/additional_698_related/data/ERR398934
**NA18506**	1000G_2504_high_coverage/additional_698_related/data/ERR398934
**NA18515**	1000G_2504_high_coverage/additional_698_related/data/ERR398935
**NA18518**	1000G_2504_high_coverage/additional_698_related/data/ERR398935
**NA18521**	1000G_2504_high_coverage/additional_698_related/data/ERR398935
**NA18852**	1000G_2504_high_coverage/additional_698_related/data/ERR398935
**NA18854**	1000G_2504_high_coverage/additional_698_related/data/ERR398935
**NA18855**	1000G_2504_high_coverage/additional_698_related/data/ERR398935
**NA18857**	1000G_2504_high_coverage/additional_698_related/data/ERR398935
**NA18859**	1000G_2504_high_coverage/additional_698_related/data/ERR398935
**NA18860**	1000G_2504_high_coverage/additional_698_related/data/ERR398935
**NA18862**	1000G_2504_high_coverage/additional_698_related/data/ERR398935
**NA18863**	1000G_2504_high_coverage/additional_698_related/data/ERR398936
**NA18869**	1000G_2504_high_coverage/additional_698_related/data/ERR398936
**NA18872**	1000G_2504_high_coverage/additional_698_related/data/ERR398936
**NA18875**	1000G_2504_high_coverage/additional_698_related/data/ERR398936
**NA18906**	1000G_2504_high_coverage/additional_698_related/data/ERR398936
**NA18911**	1000G_2504_high_coverage/additional_698_related/data/ERR398936
**NA18913**	1000G_2504_high_coverage/additional_698_related/data/ERR398936
**NA18914**	1000G_2504_high_coverage/additional_698_related/data/ERR398936
**NA18925**	1000G_2504_high_coverage/additional_698_related/data/ERR398936
**NA18930**	1000G_2504_high_coverage/additional_698_related/data/ERR398936
**NA18935**	1000G_2504_high_coverage/additional_698_related/data/ERR398937
**NA19094**	1000G_2504_high_coverage/additional_698_related/data/ERR398937
**NA19097**	1000G_2504_high_coverage/additional_698_related/data/ERR398937
**NA19100**	1000G_2504_high_coverage/additional_698_related/data/ERR398937

**NA19101**	1000G_2504_high_coverage/additional_698_related/data/ERR398937
**NA19103**	1000G_2504_high_coverage/additional_698_related/data/ERR398937
**NA19109**	1000G_2504_high_coverage/additional_698_related/data/ERR398937
**NA19115**	1000G_2504_high_coverage/additional_698_related/data/ERR398937
**NA19120**	1000G_2504_high_coverage/additional_698_related/data/ERR398937
**NA19122**	1000G_2504_high_coverage/additional_698_related/data/ERR398937
**NA19123**	1000G_2504_high_coverage/additional_698_related/data/ERR398938
**NA19127**	1000G_2504_high_coverage/additional_698_related/data/ERR398938
**NA19128**	1000G_2504_high_coverage/additional_698_related/data/ERR398938
**NA19132**	1000G_2504_high_coverage/additional_698_related/data/ERR398938
**NA19139**	1000G_2504_high_coverage/additional_698_related/data/ERR398938
**NA19140**	1000G_2504_high_coverage/additional_698_related/data/ERR398938
**NA19142**	1000G_2504_high_coverage/additional_698_related/data/ERR398938
**NA19145**	1000G_2504_high_coverage/additional_698_related/data/ERR398938
**NA19148**	1000G_2504_high_coverage/additional_698_related/data/ERR398938
**NA19150**	1000G_2504_high_coverage/additional_698_related/data/ERR398938
**NA19151**	1000G_2504_high_coverage/additional_698_related/data/ERR398939
**NA19154**	1000G_2504_high_coverage/additional_698_related/data/ERR398939
**NA19161**	1000G_2504_high_coverage/additional_698_related/data/ERR398939
**NA19173**	1000G_2504_high_coverage/additional_698_related/data/ERR398939
**NA19174**	1000G_2504_high_coverage/additional_698_related/data/ERR398939
**NA19176**	1000G_2504_high_coverage/additional_698_related/data/ERR398939
**NA19177**	1000G_2504_high_coverage/additional_698_related/data/ERR398939
**NA19186**	1000G_2504_high_coverage/additional_698_related/data/ERR398939
**NA19191**	1000G_2504_high_coverage/additional_698_related/data/ERR398939
**NA19199**	1000G_2504_high_coverage/additional_698_related/data/ERR398939
**NA19202**	1000G_2504_high_coverage/additional_698_related/data/ERR398940
**NA19203**	1000G_2504_high_coverage/additional_698_related/data/ERR398940
**NA19205**	1000G_2504_high_coverage/additional_698_related/data/ERR398940
**NA19208**	1000G_2504_high_coverage/additional_698_related/data/ERR398940
**NA19211**	1000G_2504_high_coverage/additional_698_related/data/ERR398940
**NA19215**	1000G_2504_high_coverage/additional_698_related/data/ERR398940
**NA19221**	1000G_2504_high_coverage/additional_698_related/data/ERR398940
**NA19224**	1000G_2504_high_coverage/additional_698_related/data/ERR398940
**NA19226**	1000G_2504_high_coverage/additional_698_related/data/ERR398940
**NA19237**	1000G_2504_high_coverage/additional_698_related/data/ERR398940
**NA19240**	1000G_2504_high_coverage/additional_698_related/data/ERR398941
**NA19249**	1000G_2504_high_coverage/additional_698_related/data/ERR398941
**NA19258**	1000G_2504_high_coverage/additional_698_related/data/ERR398941
**NA19650**	1000G_2504_high_coverage/additional_698_related/data/ERR398941
**NA19653**	1000G_2504_high_coverage/additional_698_related/data/ERR398941
**NA19656**	1000G_2504_high_coverage/additional_698_related/data/ERR398941
**NA19659**	1000G_2504_high_coverage/additional_698_related/data/ERR398941
**NA19660**	1000G_2504_high_coverage/additional_698_related/data/ERR398941
**NA19662**	1000G_2504_high_coverage/additional_698_related/data/ERR398941
**NA19665**	1000G_2504_high_coverage/additional_698_related/data/ERR398941
**NA19671**	1000G_2504_high_coverage/additional_698_related/data/ERR398942
**NA19675**	1000G_2504_high_coverage/additional_698_related/data/ERR398942
**NA19677**	1000G_2504_high_coverage/additional_698_related/data/ERR398942
**NA19680**	1000G_2504_high_coverage/additional_698_related/data/ERR398942
**NA19683**	1000G_2504_high_coverage/additional_698_related/data/ERR398942
**NA19685**	1000G_2504_high_coverage/additional_698_related/data/ERR398942
**NA19686**	1000G_2504_high_coverage/additional_698_related/data/ERR398942
**NA19702**	1000G_2504_high_coverage/additional_698_related/data/ERR398942
**NA19705**	1000G_2504_high_coverage/additional_698_related/data/ERR398942
**NA19718**	1000G_2504_high_coverage/additional_698_related/data/ERR398942
**NA19721**	1000G_2504_high_coverage/additional_698_related/data/ERR398943
**NA19724**	1000G_2504_high_coverage/additional_698_related/data/ERR398943
**NA19727**	1000G_2504_high_coverage/additional_698_related/data/ERR398943
**NA19730**	1000G_2504_high_coverage/additional_698_related/data/ERR398943
**NA19733**	1000G_2504_high_coverage/additional_698_related/data/ERR398943
**NA19748**	1000G_2504_high_coverage/additional_698_related/data/ERR398943
**NA19751**	1000G_2504_high_coverage/additional_698_related/data/ERR398943
**NA19757**	1000G_2504_high_coverage/additional_698_related/data/ERR398943
**NA19760**	1000G_2504_high_coverage/additional_698_related/data/ERR398943

**NA19763**	1000G_2504_high_coverage/additional_698_related/data/ERR398943
**NA19772**	1000G_2504_high_coverage/additional_698_related/data/ERR398944
**NA19775**	1000G_2504_high_coverage/additional_698_related/data/ERR398944
**NA19778**	1000G_2504_high_coverage/additional_698_related/data/ERR398944
**NA19781**	1000G_2504_high_coverage/additional_698_related/data/ERR398944
**NA19784**	1000G_2504_high_coverage/additional_698_related/data/ERR398944
**NA19787**	1000G_2504_high_coverage/additional_698_related/data/ERR398944
**NA19790**	1000G_2504_high_coverage/additional_698_related/data/ERR398944
**NA19796**	1000G_2504_high_coverage/additional_698_related/data/ERR398944
**NA19828**	1000G_2504_high_coverage/additional_698_related/data/ERR398944
**NA19836**	1000G_2504_high_coverage/additional_698_related/data/ERR398944
**NA19902**	1000G_2504_high_coverage/additional_698_related/data/ERR398945
**NA19918**	1000G_2504_high_coverage/additional_698_related/data/ERR398945
**NA19919**	1000G_2504_high_coverage/additional_698_related/data/ERR398945
**NA19924**	1000G_2504_high_coverage/additional_698_related/data/ERR398945
**NA19983**	1000G_2504_high_coverage/additional_698_related/data/ERR398945
**NA20128**	1000G_2504_high_coverage/additional_698_related/data/ERR398945
**NA20129**	1000G_2504_high_coverage/additional_698_related/data/ERR398945
**NA20279**	1000G_2504_high_coverage/additional_698_related/data/ERR398945
**NA20358**	1000G_2504_high_coverage/additional_698_related/data/ERR398945
