# Supplementary material for: High-throughput complement component 4 genomic sequence analysis with C4Investigator
Source: bioRxiv. 2023 Jul 19:2023.07.18.549551. Preprint. [Version 1] doi: 10.1101/2023.07.18.549551 (PMC10370142; doi:10.1101/2023.07.18.549551)
Supplement: Supplement 2 [file media-2.pdf]

Table_S2_suppInfo

Sample name	Sex	Biosample ID	Population code	Population name	Superpopulation code	Superpopulation name	Population elastic ID	Data collections
HG00315	female	SAME124335	FIN	Finnish	EUR	European Ancestry	FIN	1000 Genomes on GRCh38,1000 Genomes 30x on GRCh38,1000 Genomes phase 3 release,1000 Genomes phase 1 release,Geuvadis
HG00327	female	SAME123791	FIN	Finnish	EUR	European Ancestry	FIN	1000 Genomes on GRCh38,1000 Genomes 30x on GRCh38,1000 Genomes phase 3 release,1000 Genomes phase 1 release,Geuvadis
HG00334	female	SAME123981	FIN	Finnish	EUR	European Ancestry	FIN	1000 Genomes on GRCh38,1000 Genomes 30x on GRCh38,1000 Genomes phase 3 release,1000 Genomes phase 1 release,Geuvadis
HG00339	female	SAME123991	FIN	Finnish	EUR	European Ancestry	FIN	1000 Genomes on GRCh38,1000 Genomes 30x on GRCh38,1000 Genomes phase 3 release,1000 Genomes phase 1 release,Geuvadis
HG00341	male	SAME124922	FIN	Finnish	EUR	European Ancestry	FIN	1000 Genomes on GRCh38,1000 Genomes 30x on GRCh38,1000 Genomes phase 3 release,1000 Genomes phase 1 release,Geuvadis
HG00346	female	SAME125264	FIN	Finnish	EUR	European Ancestry	FIN	1000 Genomes on GRCh38,1000 Genomes 30x on GRCh38,1000 Genomes phase 3 release,1000 Genomes phase 1 release,Geuvadis
HG00353	female	SAME125120	FIN	Finnish	EUR	European Ancestry	FIN	1000 Genomes on GRCh38,1000 Genomes 30x on GRCh38,1000 Genomes phase 3 release,1000 Genomes phase 1 release,Geuvadis
HG00358	male	SAME125126	FIN	Finnish	EUR	European Ancestry	FIN	1000 Genomes on GRCh38,1000 Genomes 30x on GRCh38,1000 Genomes phase 3 release,1000 Genomes phase 1 release,Geuvadis
HG00360	male	SAME124539	FIN	Finnish,Finnish	EUR	European Ancestry,West Eurasia (SGDP)	FIN,FinnishSGDP	1000 Genomes on GRCh38,Simons Genome Diversity Project,1000 Genomes 30x on GRCh38,1000 Genomes phase 3 release,1000 Genomes phase 1 release,Geuvadis
HG00365	female	SAME124542	FIN	Finnish	EUR	European Ancestry	FIN	1000 Genomes on GRCh38,1000 Genomes 30x on GRCh38,1000 Genomes phase 3 release,1000 Genomes phase 1 release,Geuvadis
HG00372	male	SAME124748	FIN	Finnish	EUR	European Ancestry	FIN	1000 Genomes on GRCh38,1000 Genomes 30x on GRCh38,1000 Genomes phase 3 release,1000 Genomes phase 1 release,Geuvadis
HG00384	female	SAME123034	FIN	Finnish	EUR	European Ancestry	FIN	1000 Genomes on GRCh38,1000 Genomes 30x on GRCh38,1000 Genomes phase 3 release,1000 Genomes phase 1 release,Geuvadis
HG00404	female	SAME123158	CHS	Southern Han Chinese	EAS	East Asian Ancestry	CHS	1000 Genomes on GRCh38,1000 Genomes 30x on GRCh38,1000 Genomes phase 3 release,1000 Genomes phase 1 release
HG00409	male	SAME1840026	CHS	Southern Han Chinese	EAS	East Asian Ancestry	CHS	1000 Genomes on GRCh38,1000 Genomes 30x on GRCh38,1000 Genomes phase 3 release
HG00106	female	SAME123948	GBR	British	EUR	European Ancestry	GBR	1000 Genomes on GRCh38,1000 Genomes 30x on GRCh38,1000 Genomes phase 3 release,1000 Genomes phase 1 release,Geuvadis
HG00113	male	SAME125342	GBR	British	EUR	European Ancestry	GBR	1000 Genomes on GRCh38,1000 Genomes 30x on GRCh38,1000 Genomes phase 3 release,1000 Genomes phase 1 release
HG00118	female	SAME125347	GBR	British	EUR	European Ancestry	GBR	1000 Genomes on GRCh38,1000 Genomes 30x on GRCh38,1000 Genomes phase 3 release,1000 Genomes phase 1 release,Geuvadis
HG00120	female	SAME122874	GBR	British	EUR	European Ancestry	GBR	1000 Genomes on GRCh38,1000 Genomes 30x on GRCh38,1000 Genomes phase 3 release,1000 Genomes phase 1 release,Geuvadis
HG00125	female	SAME122869	GBR	British	EUR	European Ancestry	GBR	1000 Genomes on GRCh38,1000 Genomes 30x on GRCh38,1000 Genomes phase 3 release,1000 Genomes phase 1 release,Geuvadis
HG00132	female	SAME123061	GBR	British	EUR	European Ancestry	GBR	1000 Genomes on GRCh38,1000 Genomes 30x on GRCh38,1000 Genomes phase 3 release,1000 Genomes phase 1 release,Geuvadis
HG00137	female	SAME123066	GBR	British	EUR	European Ancestry	GBR	1000 Genomes on GRCh38,1000 Genomes 30x on GRCh38,1000 Genomes phase 3 release,1000 Genomes phase 1 release,Geuvadis
HG00149	male	SAME124387	GBR	British	EUR	European Ancestry	GBR	1000 Genomes on GRCh38,1000 Genomes 30x on GRCh38,1000 Genomes phase 3 release,1000 Genomes phase 1 release,Geuvadis
HG00151	male	SAME124592	GBR	British	EUR	European Ancestry	GBR	1000 Genomes on GRCh38,1000 Genomes 30x on GRCh38,1000 Genomes phase 3 release,1000 Genomes phase 1 release,Geuvadis
HG00182	male	SAME123643	FIN	Finnish	EUR	European Ancestry	FIN	1000 Genomes on GRCh38,1000 Genomes 30x on GRCh38,1000 Genomes phase 3 release,1000 Genomes phase 1 release,Geuvadis
HG00187	male	SAME123646	FIN	Finnish	EUR	European Ancestry	FIN	1000 Genomes on GRCh38,1000 Genomes 30x on GRCh38,1000 Genomes phase 3 release,1000 Genomes phase 1 release,Geuvadis
HG00233	female	SAME124127	GBR	British	EUR	European Ancestry	GBR	1000 Genomes on GRCh38,1000 Genomes 30x on GRCh38,1000 Genomes phase 3 release,1000 Genomes phase 1 release,Geuvadis
HG00238	female	SAME124131	GBR	British	EUR	European Ancestry	GBR	1000 Genomes on GRCh38,1000 Genomes 30x on GRCh38,1000 Genomes phase 3 release,1000 Genomes phase 1 release,Geuvadis
HG00240	female	SAME123221	GBR	British	EUR	European Ancestry	GBR	1000 Genomes on GRCh38,1000 Genomes 30x on GRCh38,1000 Genomes phase 3 release,1000 Genomes phase 1 release,Geuvadis
HG00245	female	SAME123218	GBR	British	EUR	European Ancestry	GBR	1000 Genomes on GRCh38,1000 Genomes 30x on GRCh38,1000 Genomes phase 3 release,1000 Genomes phase 1 release,Geuvadis
HG00252	male	SAME123052	GBR	British	EUR	European Ancestry	GBR	1000 Genomes on GRCh38,1000 Genomes 30x on GRCh38,1000 Genomes phase 3 release,1000 Genomes phase 1 release,Geuvadis
HG00257	female	SAME123047	GBR	British	EUR	European Ancestry	GBR	1000 Genomes on GRCh38,1000 Genomes 30x on GRCh38,1000 Genomes phase 3 release,1000 Genomes phase 1 release,Geuvadis
HG00097	female	SAME123267	GBR	British	EUR	European Ancestry	GBR	1000 Genomes on GRCh38,1000 Genomes 30x on GRCh38,1000 Genomes phase 3 release,1000 Genomes phase 1 release,Geuvadis
HG00264	male	SAME123578	GBR	British	EUR	European Ancestry	GBR	1000 Genomes on GRCh38,1000 Genomes 30x on GRCh38,1000 Genomes phase 3 release,1000 Genomes phase 1 release,Geuvadis
HG00100	female	SAME125154	GBR	British	EUR	European Ancestry	GBR	1000 Genomes on GRCh38,1000 Genomes 30x on GRCh38,1000 Genomes phase 3 release,1000 Genomes phase 1 release,Geuvadis
HG00269	female	SAME123575	FIN	Finnish	EUR	European Ancestry	FIN	1000 Genomes on GRCh38,1000 Genomes 30x on GRCh38,1000 Genomes phase 3 release,1000 Genomes phase 1 release,Geuvadis
HG00105	male	SAME123949	GBR	British	EUR	European Ancestry	GBR	1000 Genomes on GRCh38,1000 Genomes 30x on GRCh38,1000 Genomes phase 3 release,1000 Genomes phase 1 release,Geuvadis
HG00271	male	SAME123417	FIN	Finnish	EUR	European Ancestry	FIN	1000 Genomes on GRCh38,1000 Genomes 30x on GRCh38,1000 Genomes phase 3 release,1000 Genomes phase 1 release,Geuvadis
HG00112	male	SAME125341	GBR	British	EUR	European Ancestry	GBR	1000 Genomes on GRCh38,1000 Genomes 30x on GRCh38,1000 Genomes phase 3 release,1000 Genomes phase 1 release,Geuvadis
HG00276	female	SAME123424	FIN	Finnish	EUR	European Ancestry	FIN	1000 Genomes on GRCh38,1000 Genomes 30x on GRCh38,1000 Genomes phase 3 release,1000 Genomes phase 1 release,Geuvadis
HG00117	male	SAME125346	GBR	British	EUR	European Ancestry	GBR	1000 Genomes on GRCh38,1000 Genomes 30x on GRCh38,1000 Genomes phase 3 release,1000 Genomes phase 1 release,Geuvadis
HG00288	female	SAME1839246	FIN	Finnish	EUR	European Ancestry	FIN	1000 Genomes on GRCh38,1000 Genomes 30x on GRCh38,1000 Genomes phase 3 release
HG00290	male	SAME1839057	FIN	Finnish	EUR	European Ancestry	FIN	1000 Genomes on GRCh38,1000 Genomes 30x on GRCh38,1000 Genomes phase 3 release
HG00129	male	SAME122867	GBR	British	EUR	European Ancestry	GBR	1000 Genomes on GRCh38,1000 Genomes 30x on GRCh38,1000 Genomes phase 3 release,1000 Genomes phase 1 release,Geuvadis
HG00131	male	SAME123064	GBR	British	EUR	European Ancestry	GBR	1000 Genomes on GRCh38,1000 Genomes 30x on GRCh38,1000 Genomes phase 3 release,1000 Genomes phase 1 release,Geuvadis
HG00308	male	SAME124161	FIN	Finnish	EUR	European Ancestry	FIN	1000 Genomes on GRCh38,1000 Genomes 30x on GRCh38,1000 Genomes phase 3 release,Geuvadis
HG00136	male	SAME123065	GBR	British	EUR	European Ancestry	GBR	1000 Genomes on GRCh38,1000 Genomes 30x on GRCh38,1000 Genomes phase 3 release,1000 Genomes phase 1 release,Geuvadis
HG00310	male	SAME124338	FIN	Finnish	EUR	European Ancestry	FIN	1000 Genomes on GRCh38,1000 Genomes 30x on GRCh38,1000 Genomes phase 3 release,1000 Genomes phase 1 release,Geuvadis
HG00143	male	SAME124393	GBR	British	EUR	European Ancestry	GBR	1000 Genomes on GRCh38,1000 Genomes 30x on GRCh38,1000 Genomes phase 3 release,1000 Genomes phase 1 release,Geuvadis
HG00148	male	SAME124388	GBR	British	EUR	European Ancestry	GBR	1000 Genomes on GRCh38,1000 Genomes 30x on GRCh38,1000 Genomes phase 3 release,1000 Genomes phase 1 release,Geuvadis
HG00101	male	SAME125153	GBR	British	EUR	European Ancestry	GBR	1000 Genomes on GRCh38,1000 Genomes 30x on GRCh38,1000 Genomes phase 3 release,1000 Genomes phase 1 release,Geuvadis
HG00150	female	SAME124591	GBR	British	EUR	European Ancestry	GBR	1000 Genomes on GRCh38,1000 Genomes 30x on GRCh38,1000 Genomes phase 3 release,1000 Genomes phase 1 release,Geuvadis
HG00423	female	SAME122810	CHS	Southern Han Chinese	EAS	East Asian Ancestry	CHS	1000 Genomes 30x on GRCh38,1000 Genomes phase 3 release
HG00155	male	SAME124588	GBR	British	EUR	European Ancestry	GBR	1000 Genomes on GRCh38,1000 Genomes 30x on GRCh38,1000 Genomes phase 3 release,1000 Genomes phase 1 release,Geuvadis
HG00428	female	SAME122811	CHS	Southern Han Chinese	EAS	East Asian Ancestry	CHS	1000 Genomes on GRCh38,1000 Genomes 30x on GRCh38,1000 Genomes phase 3 release,1000 Genomes phase 1 release,90 Han Chinese high coverage genomes
HG00174	female	SAME124958	FIN	Finnish,Finnish	EUR	European Ancestry,West Eurasia (SGDP)	FIN,FinnishSGDP	1000 Genomes on GRCh38,Simons Genome Diversity Project,1000 Genomes 30x on GRCh38,1000 Genomes phase 3 release,1000 Genomes phase 1 release,Geuvadis
HG00442	male	SAME124912	CHS	Southern Han Chinese	EAS	East Asian Ancestry	CHS	1000 Genomes on GRCh38,1000 Genomes 30x on GRCh38,1000 Genomes phase 3 release,1000 Genomes phase 1 release,90 Han Chinese high coverage genomes
HG00179	female	SAME124965	FIN	Finnish	EUR	European Ancestry	FIN	1000 Genomes on GRCh38,1000 Genomes 30x on GRCh38,1000 Genomes phase 3 release,1000 Genomes phase 1 release,Geuvadis
HG00447	female	SAME124953	CHS	Southern Han Chinese	EAS	East Asian Ancestry	CHS	1000 Genomes 30x on GRCh38,1000 Genomes phase 3 release
HG00181	male	SAME123644	FIN	Finnish	EUR	European Ancestry	FIN	1000 Genomes on GRCh38,1000 Genomes 30x on GRCh38,1000 Genomes phase 3 release,1000 Genomes phase 1 release,Geuvadis
HG00459	male	SAME125269	CHS	Southern Han Chinese	EAS	East Asian Ancestry	CHS	1000 Genomes 30x on GRCh38,1000 Genomes phase 3 release
HG00186	male	SAME123647	FIN	Finnish	EUR	European Ancestry	FIN	1000 Genomes on GRCh38,1000 Genomes 30x on GRCh38,1000 Genomes phase 3 release,1000 Genomes phase 1 release,Geuvadis
HG00473	female	SAME123338	CHS	Southern Han Chinese	EAS	East Asian Ancestry	CHS	1000 Genomes on GRCh38,1000 Genomes 30x on GRCh38,1000 Genomes phase 3 release,1000 Genomes phase 1 release,90 Han Chinese high coverage genomes
HG00232	female	SAME124128	GBR	British	EUR	European Ancestry	GBR	1000 Genomes on GRCh38,1000 Genomes 30x on GRCh38,1000 Genomes phase 3 release,1000 Genomes phase 1 release,Geuvadis
HG00478	male	SAME123335	CHS	Southern Han Chinese	EAS	East Asian Ancestry	CHS	1000 Genomes on GRCh38,1000 Genomes 30x on GRCh38,1000 Genomes phase 3 release,1000 Genomes phase 1 release,90 Han Chinese high coverage genomes
HG00237	female	SAME124124	GBR	British	EUR	European Ancestry	GBR	1000 Genomes on GRCh38,1000 Genomes 30x on GRCh38,1000 Genomes phase 3 release,1000 Genomes phase 1 release,Geuvadis
HG00480	male	SAME124617	CHS	Southern Han Chinese	EAS	East Asian Ancestry	CHS	1000 Genomes 30x on GRCh38,1000 Genomes phase 3 release
HG00244	male	SAME123217	GBR	British	EUR	European Ancestry	GBR	1000 Genomes on GRCh38,1000 Genomes 30x on GRCh38,1000 Genomes phase 3 release,1000 Genomes phase 1 release,Geuvadis
HG00500	male	SAME123363	CHS	Southern Han Chinese	EAS	East Asian Ancestry	CHS	1000 Genomes on GRCh38,1000 Genomes 30x on GRCh38,1000 Genomes phase 3 release,1000 Genomes phase 1 release,90 Han Chinese high coverage genomes
HG00319	female	SAME124334	FIN	Finnish	EUR	European Ancestry	FIN	1000 Genomes on GRCh38,1000 Genomes 30x on GRCh38,1000 Genomes phase 3 release,1000 Genomes phase 1 release,Geuvadis
HG00512	male	SAME123525	CHS	Southern Han Chinese	EAS	East Asian Ancestry	CHS	1000 Genomes on GRCh38,1000 Genomes 30x on GRCh38,Human Genome Structural Variation Consortium, Phase 2,The Human Genome Structural Variation Consortiu
HG00321	male	SAME123796	FIN	Finnish	EUR	European Ancestry	FIN	1000 Genomes on GRCh38,1000 Genomes 30x on GRCh38,1000 Genomes phase 3 release,1000 Genomes phase 1 release,Geuvadis
HG00524	male	SAME123724	CHS	Southern Han Chinese	EAS	East Asian Ancestry	CHS	1000 Genomes on GRCh38,1000 Genomes 30x on GRCh38,1000 Genomes phase 3 release,1000 Genomes phase 1 release,90 Han Chinese high coverage genomes
HG00326	female	SAME123792	FIN	Finnish	EUR	European Ancestry	FIN	1000 Genomes on GRCh38,1000 Genomes 30x on GRCh38,1000 Genomes phase 3 release,1000 Genomes phase 1 release,Geuvadis
HG00531	female	SAME125381	CHS	Southern Han Chinese	EAS	East Asian Ancestry	CHS	1000 Genomes on GRCh38,1000 Genomes 30x on GRCh38,1000 Genomes phase 3 release,1000 Genomes phase 1 release,90 Han Chinese high coverage genomes
HG00338	male	SAME123990	FIN	Finnish	EUR	European Ancestry	FIN	1000 Genomes on GRCh38,1000 Genomes 30x on GRCh38,1000 Genomes phase 3 release,1000 Genomes phase 1 release,Geuvadis
HG00536	male	SAME124344	CHS	Southern Han Chinese	EAS	East Asian Ancestry	CHS	1000 Genomes on GRCh38,1000 Genomes 30x on GRCh38,1000 Genomes phase 3 release,1000 Genomes phase 1 release,90 Han Chinese high coverage genomes
HG00345	male	SAME124919	FIN	Finnish	EUR	European Ancestry	FIN	1000 Genomes on GRCh38,1000 Genomes 30x on GRCh38,1000 Genomes phase 3 release,1000 Genomes phase 1 release,Geuvadis
HG00733	female	SAME125399	PUR	Puerto Rican	AMR	American Ancestry	PUR	1000 Genomes on GRCh38,1000 Genomes 30x on GRCh38,Human Genome Structural Variation Consortium, Phase 2,The Human Genome Structural Variation Consortiu
HG00357	female	SAME125124	FIN	Finnish	EUR	European Ancestry	FIN	1000 Genomes on GRCh38,1000 Genomes 30x on GRCh38,1000 Genomes phase 3 release,1000 Genomes phase 1 release
HG00738	male	SAME125368	PUR	Puerto Rican	AMR	American Ancestry	PUR	1000 Genomes 30x on GRCh38,1000 Genomes phase 3 release
HG00364	female	SAME124543	FIN	Finnish	EUR	European Ancestry	FIN	1000 Genomes on GRCh38,1000 Genomes 30x on GRCh38,1000 Genomes phase 3 release,1000 Genomes phase 1 release,Geuvadis
HG00740	female	SAME124834	PUR	Puerto Rican	AMR	American Ancestry	PUR	1000 Genomes on GRCh38,1000 Genomes 30x on GRCh38,1000 Genomes phase 3 release,1000 Genomes phase 1 release
HG00369	male	SAME124534	FIN	Finnish	EUR	European Ancestry	FIN	1000 Genomes on GRCh38,1000 Genomes 30x on GRCh38,1000 Genomes phase 3 release,1000 Genomes phase 1 release,Geuvadis
HG00978	female	SAME124727	CDX	Dai Chinese	EAS	East Asian Ancestry	CDX	1000 Genomes on GRCh38,1000 Genomes 30x on GRCh38,1000 Genomes phase 3 release
HG00371	male	SAME124747	FIN	Finnish	EUR	European Ancestry	FIN	1000 Genomes on GRCh38,1000 Genomes 30x on GRCh38,1000 Genomes phase 3 release,1000 Genomes phase 1 release,Geuvadis
HG01192	male	SAME124298	PUR	Puerto Rican	AMR	American Ancestry	PUR	1000 Genomes 30x on GRCh38,1000 Genomes phase 3 release
HG00376	female	SAME124751	FIN	Finnish	EUR	European Ancestry	FIN	1000 Genomes on GRCh38,1000 Genomes 30x on GRCh38,1000 Genomes phase 3 release,1000 Genomes phase 1 release,Geuvadis
HG01197	male	SAME124237	PUR	Puerto Rican	AMR	American Ancestry	PUR	1000 Genomes on GRCh38,1000 Genomes 30x on GRCh38,1000 Genomes phase 3 release,1000 Genomes phase 1 release
HG00383	female	SAME123032	FIN	Finnish	EUR	European Ancestry	FIN	1000 Genomes on GRCh38,1000 Genomes 30x on GRCh38,1000 Genomes phase 3 release,1000 Genomes phase 1 release,Geuvadis
HG01200	male	SAME1840109	PUR	Puerto Rican	AMR	American Ancestry	PUR	1000 Genomes on GRCh38,1000 Genomes 30x on GRCh38,1000 Genomes phase 3 release
HG00403	male	SAME123160	CHS	Southern Han Chinese	EAS	East Asian Ancestry	CHS	1000 Genomes on GRCh38,1000 Genomes 30x on GRCh38,1000 Genomes phase 3 release,1000 Genomes phase 1 release
HG01205	female	SAME124296	PUR	Puerto Rican	AMR	American Ancestry	PUR	1000 Genomes on GRCh38,1000 Genomes 30x on GRCh38,1000 Genomes phase 3 release,1000 Genomes phase 1 release
HG00408	female	SAME123155	CHS	Southern Han Chinese	EAS	East Asian Ancestry	CHS	1000 Genomes 30x on GRCh38,1000 Genomes phase 3 release
HG01243	male	SAME125063	PUR	Puerto Rican	AMR	American Ancestry	PUR	1000 Genomes 30x on GRCh38,1000 Genomes phase 3 release
HG00410	female	SAME1839734	CHS	Southern Han Chinese	EAS	East Asian Ancestry	CHS	1000 Genomes on GRCh38,1000 Genomes 30x on GRCh38,1000 Genomes phase 3 release
HG01248	female	SAME125058	PUR	Puerto Rican	AMR	American Ancestry	PUR	1000 Genomes on GRCh38,1000 Genomes 30x on GRCh38,1000 Genomes phase 3 release,1000 Genomes phase 1 release
HG00422	female	SAME122809	CHS	Southern Han Chinese	EAS	East Asian Ancestry	CHS	1000 Genomes on GRCh38,1000 Genomes 30x on GRCh38,1000 Genomes phase 3 release,1000 Genomes phase 1 release,90 Han Chinese high coverage genomes
HG01250	male	SAME125281	CLM	Colombian	AMR	American Ancestry	CLM	1000 Genomes on GRCh38,1000 Genomes 30x on GRCh38,1000 Genomes phase 3 release,1000 Genomes phase 1 release
HG00542	male	SAME124352	CHS	Southern Han Chinese	EAS	East Asian Ancestry	CHS	1000 Genomes on GRCh38,1000 Genomes 30x on GRCh38,1000 Genomes phase 3 release,1000 Genomes phase 1 release,90 Han Chinese high coverage genomes
HG01255	male	SAME125286	CLM	Colombian	AMR	American Ancestry	CLM	1000 Genomes 30x on GRCh38,1000 Genomes phase 3 release
HG00554	female	SAME123969	PUR	Puerto Rican	AMR	American Ancestry	PUR	1000 Genomes on GRCh38,1000 Genomes 30x on GRCh38,1000 Genomes phase 3 release,1000 Genomes phase 1 release
HG01274	male	SAME123001	CLM	Colombian	AMR	American Ancestry	CLM	1000 Genomes on GRCh38,1000 Genomes 30x on GRCh38,1000 Genomes phase 3 release,1000 Genomes phase 1 release
HG00559	male	SAME124248	CHS	Southern Han Chinese	EAS	East Asian Ancestry	CHS	1000 Genomes on GRCh38,1000 Genomes 30x on GRCh38,1000 Genomes phase 3 release,1000 Genomes phase 1 release
HG01279	female	SAME123004	CLM	Colombian	AMR	American Ancestry	CLM	1000 Genomes 30x on GRCh38,1000 Genomes phase 3 release
HG00561	female	SAME124455	CHS	Southern Han Chinese	EAS	East Asian Ancestry	CHS	1000 Genomes 30x on GRCh38,1000 Genomes phase 3 release
HG00620	female	SAME123081	CHS	Southern Han Chinese	EAS	East Asian Ancestry	CHS	1000 Genomes on GRCh38,1000 Genomes 30x on GRCh38,1000 Genomes phase 3 release,1000 Genomes phase 1 release,90 Han Chinese high coverage genomes
HG00566	female	SAME125048	CHS	Southern Han Chinese	EAS	East Asian Ancestry	CHS	1000 Genomes on GRCh38,1000 Genomes 30x on GRCh38,1000 Genomes phase 3 release,1000 Genomes phase 1 release
HG00625	male	SAME123080	CHS	Southern Han Chinese	EAS	East Asian Ancestry	CHS	1000 Genomes on GRCh38,1000 Genomes 30x on GRCh38,1000 Genomes phase 3 release,1000 Genomes phase 1 release
HG00578	female	SAME123528	CHS	Southern Han Chinese	EAS	East Asian Ancestry	CHS	1000 Genomes on GRCh38,1000 Genomes 30x on GRCh38,1000 Genomes phase 3 release,1000 Genomes phase 1 release

**HG00632**	female	SAME1839151	CHS	Southern Han Chinese	EAS	East Asian Ancestry	CHS	1000 Genomes on GRCh38,1000 Genomes 30x on GRCh38,1000 Genomes phase 3 release
**HG00580**	male	SAME124832	CHS	Southern Han Chinese	EAS	East Asian Ancestry	CHS	1000 Genomes on GRCh38,1000 Genomes 30x on GRCh38,1000 Genomes phase 3 release,1000 Genomes phase 1 release,90 Han Chinese high coverage genomes
**HG00637**	male	SAME123710	PUR	Puerto Rican	AMR	American Ancestry	PUR	1000 Genomes on GRCh38,1000 Genomes 30x on GRCh38,1000 Genomes phase 3 release,1000 Genomes phase 1 release
**HG00585**	female	SAME124722	CHS	Southern Han Chinese	EAS	East Asian Ancestry	CHS	1000 Genomes 30x on GRCh38,1000 Genomes phase 3 release
**HG00651**	female	SAME123966	CHS	Southern Han Chinese	EAS	East Asian Ancestry	CHS	1000 Genomes on GRCh38,1000 Genomes 30x on GRCh38,1000 Genomes phase 3 release,1000 Genomes phase 1 release
**HG00592**	male	SAME125011	CHS	Southern Han Chinese	EAS	East Asian Ancestry	CHS	1000 Genomes on GRCh38,1000 Genomes 30x on GRCh38,1000 Genomes phase 3 release,1000 Genomes phase 1 release
**HG00656**	male	SAME123233	CHS	Southern Han Chinese	EAS	East Asian Ancestry	CHS	1000 Genomes on GRCh38,1000 Genomes 30x on GRCh38,1000 Genomes phase 3 release,1000 Genomes phase 1 release,90 Han Chinese high coverage genomes
**HG00597**	female	SAME125008	CHS	Southern Han Chinese	EAS	East Asian Ancestry	CHS	1000 Genomes 30x on GRCh38,1000 Genomes phase 3 release
**HG00663**	female	SAME123813	CHS	Southern Han Chinese	EAS	East Asian Ancestry	CHS	1000 Genomes on GRCh38,1000 Genomes 30x on GRCh38,1000 Genomes phase 3 release,1000 Genomes phase 1 release,90 Han Chinese high coverage genomes
**HG00543**	female	SAME122857	CHS	Southern Han Chinese	EAS	East Asian Ancestry	CHS	1000 Genomes on GRCh38,1000 Genomes 30x on GRCh38,1000 Genomes phase 3 release,1000 Genomes phase 1 release
**HG00427**	male	SAME122806	CHS	Southern Han Chinese	EAS	East Asian Ancestry	CHS	1000 Genomes on GRCh38,1000 Genomes 30x on GRCh38,1000 Genomes phase 3 release,1000 Genomes phase 1 release,90 Han Chinese high coverage genomes
**HG00555**	female	SAME122856	PUR	Puerto Rican	AMR	American Ancestry	PUR	1000 Genomes 30x on GRCh38,1000 Genomes phase 3 release
**HG00446**	female	SAME123880	CHS	Southern Han Chinese	EAS	East Asian Ancestry	CHS	1000 Genomes on GRCh38,1000 Genomes 30x on GRCh38,1000 Genomes phase 3 release,1000 Genomes phase 1 release
**HG00567**	male	SAME124453	CHS	Southern Han Chinese	EAS	East Asian Ancestry	CHS	1000 Genomes 30x on GRCh38,1000 Genomes phase 3 release
**HG00453**	female	SAME123113	CHS	Southern Han Chinese	EAS	East Asian Ancestry	CHS	1000 Genomes 30x on GRCh38,1000 Genomes phase 3 release
**HG00579**	female	SAME123530	CHS	Southern Han Chinese	EAS	East Asian Ancestry	CHS	1000 Genomes 30x on GRCh38,1000 Genomes phase 3 release
**HG00458**	female	SAME125278	CHS	Southern Han Chinese	EAS	East Asian Ancestry	CHS	1000 Genomes on GRCh38,1000 Genomes 30x on GRCh38,1000 Genomes phase 3 release,1000 Genomes phase 1 release
**HG00581**	female	SAME124831	CHS	Southern Han Chinese	EAS	East Asian Ancestry	CHS	1000 Genomes on GRCh38,1000 Genomes 30x on GRCh38,1000 Genomes phase 3 release,1000 Genomes phase 1 release
**HG00465**	male	SAME123498	CHS	Southern Han Chinese	EAS	East Asian Ancestry	CHS	1000 Genomes 30x on GRCh38,1000 Genomes phase 3 release
**HG00593**	female	SAME125012	CHS	Southern Han Chinese	EAS	East Asian Ancestry	CHS	1000 Genomes on GRCh38,1000 Genomes 30x on GRCh38,1000 Genomes phase 3 release,1000 Genomes phase 1 release
**HG00472**	male	SAME123339	CHS	Southern Han Chinese	EAS	East Asian Ancestry	CHS	1000 Genomes on GRCh38,1000 Genomes 30x on GRCh38,1000 Genomes phase 3 release,1000 Genomes phase 1 release,90 Han Chinese high coverage genomes
**HG00598**	male	SAME1839713	CHS	Southern Han Chinese	EAS	East Asian Ancestry	CHS	1000 Genomes on GRCh38,1000 Genomes 30x on GRCh38,1000 Genomes phase 3 release
**HG00477**	male	SAME123340	CHS	Southern Han Chinese	EAS	East Asian Ancestry	CHS	1000 Genomes 30x on GRCh38,1000 Genomes phase 3 release
**HG00613**	male	SAME125196	CHS	Southern Han Chinese	EAS	East Asian Ancestry	CHS	1000 Genomes on GRCh38,1000 Genomes 30x on GRCh38,1000 Genomes phase 3 release,1000 Genomes phase 1 release
**HG00530**	male	SAME124356	CHS	Southern Han Chinese	EAS	East Asian Ancestry	CHS	1000 Genomes on GRCh38,1000 Genomes 30x on GRCh38,1000 Genomes phase 3 release,1000 Genomes phase 1 release,90 Han Chinese high coverage genomes
**HG01077**	female	SAME1839581	PUR	Puerto Rican	AMR	American Ancestry	PUR	1000 Genomes on GRCh38,1000 Genomes 30x on GRCh38,1000 Genomes phase 3 release
**HG00535**	male	SAME124348	CHS	Southern Han Chinese	EAS	East Asian Ancestry	CHS	1000 Genomes 30x on GRCh38,1000 Genomes phase 3 release
**HG01084**	female	SAME125392	PUR	Puerto Rican	AMR	American Ancestry	PUR	1000 Genomes 30x on GRCh38,1000 Genomes phase 3 release
**HG00732**	female	SAME125396	PUR	Puerto Rican	AMR	American Ancestry	PUR	1000 Genomes on GRCh38,1000 Genomes 30x on GRCh38,Human Genome Structural Variation Consortium, Phase 2,The Human Genome Structural Variation Consortiu
**HG01089**	female	SAME1839432	PUR	Puerto Rican	AMR	American Ancestry	PUR	1000 Genomes on GRCh38,1000 Genomes 30x on GRCh38,1000 Genomes phase 3 release
**HG00737**	female	SAME125416	PUR	Puerto Rican	AMR	American Ancestry	PUR	1000 Genomes on GRCh38,1000 Genomes 30x on GRCh38,1000 Genomes phase 3 release,1000 Genomes phase 1 release
**HG01096**	male	SAME123776	PUR	Puerto Rican	AMR	American Ancestry	PUR	1000 Genomes 30x on GRCh38,1000 Genomes phase 3 release
**HG00864**	female	SAME123547	CDX	Dai Chinese	EAS	East Asian Ancestry	CDX	1000 Genomes on GRCh38,1000 Genomes 30x on GRCh38,Human Genome Structural Variation Consortium, Phase 2,1000 Genomes phase 3 release
**HG01104**	male	SAME123672	PUR	Puerto Rican	AMR	American Ancestry	PUR	1000 Genomes on GRCh38,1000 Genomes 30x on GRCh38,1000 Genomes phase 3 release,1000 Genomes phase 1 release
**HG01109**	male	SAME123669	PUR	Puerto Rican	AMR	American Ancestry	PUR	1000 Genomes 30x on GRCh38,1000 Genomes phase 3 release
**HG00251**	male	SAME123053	GBR	British	EUR	European Ancestry	GBR	1000 Genomes on GRCh38,1000 Genomes 30x on GRCh38,1000 Genomes phase 3 release,1000 Genomes phase 1 release,Geuvadis
**HG01111**	female	SAME123486	PUR	Puerto Rican	AMR	American Ancestry	PUR	1000 Genomes on GRCh38,1000 Genomes 30x on GRCh38,1000 Genomes phase 3 release,1000 Genomes phase 1 release
**HG00256**	male	SAME123048	GBR	British	EUR	European Ancestry	GBR	1000 Genomes on GRCh38,1000 Genomes 30x on GRCh38,1000 Genomes phase 3 release,1000 Genomes phase 1 release,Geuvadis
**HG00263**	female	SAME123581	GBR	British	EUR	European Ancestry	GBR	1000 Genomes on GRCh38,1000 Genomes 30x on GRCh38,1000 Genomes phase 3 release,1000 Genomes phase 1 release,Geuvadis
**HG00675**	female	SAME1840076	CHS	Southern Han Chinese	EAS	East Asian Ancestry	CHS	1000 Genomes on GRCh38,1000 Genomes 30x on GRCh38,1000 Genomes phase 3 release
**HG00268**	female	SAME123574	FIN	Finnish	EUR	European Ancestry	FIN	1000 Genomes on GRCh38,1000 Genomes 30x on GRCh38,Human Genome Structural Variation Consortium, Phase 2,1000 Genomes phase 3 release,1000 Genomes ph
**HG00694**	male	SAME123154	CHS	Southern Han Chinese	EAS	East Asian Ancestry	CHS	1000 Genomes 30x on GRCh38,1000 Genomes phase 3 release
**HG00699**	female	SAME123165	CHS	Southern Han Chinese	EAS	East Asian Ancestry	CHS	1000 Genomes on GRCh38,1000 Genomes 30x on GRCh38,1000 Genomes phase 3 release,1000 Genomes phase 1 release,90 Han Chinese high coverage genomes
**HG00275**	female	SAME123421	FIN	Finnish	EUR	European Ancestry	FIN	1000 Genomes on GRCh38,1000 Genomes 30x on GRCh38,1000 Genomes phase 3 release,1000 Genomes phase 1 release,Geuvadis
**HG00702**	female	SAME122934	CHS	Southern Han Chinese	EAS	East Asian Ancestry	CHS	1000 Genomes on GRCh38,1000 Genomes 30x on GRCh38,1000 Genomes phase 3 release,1000 Genomes phase 1 release
**HG00282**	female	SAME122833	FIN	Finnish	EUR	European Ancestry	FIN	1000 Genomes on GRCh38,1000 Genomes 30x on GRCh38,1000 Genomes phase 3 release,1000 Genomes phase 1 release,Geuvadis
**HG00707**	male	SAME122929	CHS	Southern Han Chinese	EAS	East Asian Ancestry	CHS	1000 Genomes on GRCh38,1000 Genomes 30x on GRCh38,1000 Genomes phase 3 release,1000 Genomes phase 1 release
**HG01130**	male	SAME1839995	CLM	Colombian	AMR	American Ancestry	CLM	1000 Genomes on GRCh38,1000 Genomes 30x on GRCh38,1000 Genomes phase 3 release
**HG00612**	female	SAME125197	CHS	Southern Han Chinese	EAS	East Asian Ancestry	CHS	1000 Genomes 30x on GRCh38,1000 Genomes phase 3 release
**HG01135**	male	SAME123886	CLM	Colombian	AMR	American Ancestry	CLM	1000 Genomes 30x on GRCh38,1000 Genomes phase 3 release
**HG01142**	male	SAME1839731	CLM	Colombian	AMR	American Ancestry	CLM	1000 Genomes on GRCh38,1000 Genomes 30x on GRCh38,1000 Genomes phase 3 release
**HG00629**	female	SAME123086	CHS	Southern Han Chinese	EAS	East Asian Ancestry	CHS	1000 Genomes on GRCh38,1000 Genomes 30x on GRCh38,1000 Genomes phase 3 release,1000 Genomes phase 1 release
**HG01161**	male	SAME1839615	PUR	Puerto Rican	AMR	American Ancestry	PUR	1000 Genomes on GRCh38,1000 Genomes 30x on GRCh38,1000 Genomes phase 3 release
**HG00631**	male	SAME1839060	CHS	Southern Han Chinese	EAS	East Asian Ancestry	CHS	1000 Genomes on GRCh38,1000 Genomes 30x on GRCh38,1000 Genomes phase 3 release
**HG01173**	male	SAME124624	PUR	Puerto Rican	AMR	American Ancestry	PUR	1000 Genomes on GRCh38,1000 Genomes 30x on GRCh38,1000 Genomes phase 3 release,1000 Genomes phase 1 release
**HG00636**	male	SAME123711	CHS	Southern Han Chinese	EAS	East Asian Ancestry	CHS	1000 Genomes 30x on GRCh38,1000 Genomes phase 3 release
**HG01178**	male	SAME124616	PUR	Puerto Rican	AMR	American Ancestry	PUR	1000 Genomes 30x on GRCh38,1000 Genomes phase 3 release
**HG00650**	male	SAME123254	CHS	Southern Han Chinese	EAS	East Asian Ancestry	CHS	1000 Genomes on GRCh38,1000 Genomes 30x on GRCh38,1000 Genomes phase 3 release,1000 Genomes phase 1 release
**HG01046**	female	SAME123167	CDX	Dai Chinese	EAS	East Asian Ancestry	CDX	1000 Genomes on GRCh38,1000 Genomes 30x on GRCh38,1000 Genomes phase 3 release
**HG00655**	female	SAME123257	CHS	Southern Han Chinese	EAS	East Asian Ancestry	CHS	1000 Genomes 30x on GRCh38,1000 Genomes phase 3 release
**HG01053**	female	SAME124866	PUR	Puerto Rican	AMR	American Ancestry	PUR	1000 Genomes 30x on GRCh38,1000 Genomes phase 3 release
**HG00662**	male	SAME123812	CHS	Southern Han Chinese	EAS	East Asian Ancestry	CHS	1000 Genomes on GRCh38,1000 Genomes 30x on GRCh38,1000 Genomes phase 3 release,1000 Genomes phase 1 release
**HG01058**	female	SAME1840086	PUR	Puerto Rican	AMR	American Ancestry	PUR	1000 Genomes on GRCh38,1000 Genomes 30x on GRCh38,1000 Genomes phase 3 release
**HG00674**	male	SAME1840080	CHS	Southern Han Chinese	EAS	East Asian Ancestry	CHS	1000 Genomes on GRCh38,1000 Genomes 30x on GRCh38,1000 Genomes phase 3 release
**HG01060**	male	SAME123015	PUR	Puerto Rican	AMR	American Ancestry	PUR	1000 Genomes on GRCh38,1000 Genomes 30x on GRCh38,1000 Genomes phase 3 release,1000 Genomes phase 1 release
**HG00693**	female	SAME123683	CHS	Southern Han Chinese	EAS	East Asian Ancestry	CHS	1000 Genomes on GRCh38,1000 Genomes 30x on GRCh38,1000 Genomes phase 3 release,1000 Genomes phase 1 release
**HG01072**	male	SAME124091	PUR	Puerto Rican	AMR	American Ancestry	PUR	1000 Genomes on GRCh38,1000 Genomes 30x on GRCh38,1000 Genomes phase 3 release,1000 Genomes phase 1 release
**HG00698**	male	SAME123693	CHS	Southern Han Chinese	EAS	East Asian Ancestry	CHS	1000 Genomes on GRCh38,1000 Genomes 30x on GRCh38,1000 Genomes phase 3 release,1000 Genomes phase 1 release,90 Han Chinese high coverage genomes
**HG00701**	male	SAME122931	CHS	Southern Han Chinese	EAS	East Asian Ancestry	CHS	1000 Genomes on GRCh38,1000 Genomes 30x on GRCh38,1000 Genomes phase 3 release,1000 Genomes phase 1 release
**HG01488**	male	SAME123973	CLM	Colombian	AMR	American Ancestry	CLM	1000 Genomes on GRCh38,1000 Genomes 30x on GRCh38,1000 Genomes phase 3 release,1000 Genomes phase 1 release
**HG00706**	male	SAME122930	CHS	Southern Han Chinese	EAS	East Asian Ancestry	CHS	1000 Genomes 30x on GRCh38,1000 Genomes phase 3 release
**HG01490**	female	SAME124139	CLM	Colombian	AMR	American Ancestry	CLM	1000 Genomes 30x on GRCh38,1000 Genomes phase 3 release
**HG01495**	female	SAME124136	CLM	Colombian	AMR	American Ancestry	CLM	1000 Genomes on GRCh38,1000 Genomes 30x on GRCh38,1000 Genomes phase 3 release,1000 Genomes phase 1 release
**HG01052**	female	SAME123368	PUR	Puerto Rican	AMR	American Ancestry	PUR	1000 Genomes on GRCh38,1000 Genomes 30x on GRCh38,1000 Genomes phase 3 release,1000 Genomes phase 1 release
**HG01503**	male	SAME124944	IBS	Iberian,Spanish	EUR	European Ancestry,West Eurasia (SGDP)	IBS,SpanishSGDP	1000 Genomes on GRCh38,Simons Genome Diversity Project,1000 Genomes 30x on GRCh38,1000 Genomes phase 3 release
**HG01064**	female	SAME1839759	PUR	Puerto Rican	AMR	American Ancestry	PUR	1000 Genomes on GRCh38,1000 Genomes 30x on GRCh38,1000 Genomes phase 3 release
**HG01508**	male	SAME124939	IBS	Iberian	EUR	European Ancestry	IBS	1000 Genomes 30x on GRCh38,1000 Genomes phase 3 release
**HG01069**	male	SAME122825	PUR	Puerto Rican	AMR	American Ancestry	PUR	1000 Genomes on GRCh38,1000 Genomes 30x on GRCh38,1000 Genomes phase 3 release,1000 Genomes phase 1 release
**HG01565**	male	SAME124566	PEL	Peruvian	AMR	American Ancestry	PEL	1000 Genomes on GRCh38,1000 Genomes 30x on GRCh38,1000 Genomes phase 3 release
**HG01071**	female	SAME123009	PUR	Puerto Rican	AMR	American Ancestry	PUR	1000 Genomes 30x on GRCh38,1000 Genomes phase 3 release
**HG01572**	female	SAME124705	PEL	Peruvian	AMR	American Ancestry	PEL	1000 Genomes on GRCh38,1000 Genomes 30x on GRCh38,1000 Genomes phase 3 release
**HG01191**	female	SAME123651	PUR	Puerto Rican	AMR	American Ancestry	PUR	1000 Genomes on GRCh38,1000 Genomes 30x on GRCh38,1000 Genomes phase 3 release,1000 Genomes phase 1 release
**HG01577**	male	SAME124349	PEL	Peruvian	AMR	American Ancestry	PEL	1000 Genomes on GRCh38,1000 Genomes 30x on GRCh38,1000 Genomes phase 3 release
**HG01204**	male	SAME124297	PUR	Puerto Rican	AMR	American Ancestry	PUR	1000 Genomes on GRCh38,1000 Genomes 30x on GRCh38,1000 Genomes phase 3 release,1000 Genomes phase 1 release
**HG01589**	male	SAME1839859	PJL	Punjabi	SAS	South Asian Ancestry	PJL	1000 Genomes on GRCh38,1000 Genomes 30x on GRCh38,1000 Genomes phase 3 release
**HG01242**	female	SAME125064	PUR	Puerto Rican	AMR	American Ancestry	PUR	1000 Genomes on GRCh38,1000 Genomes 30x on GRCh38,1000 Genomes phase 3 release,1000 Genomes phase 1 release
**HG01596**	male	SAME125227	KHV	Kinh Vietnamese	EAS	East Asian Ancestry	KHV	1000 Genomes on GRCh38,1000 Genomes 30x on GRCh38,Human Genome Structural Variation Consortium, Phase 2,1000 Genomes phase 3 release
**HG01247**	male	SAME125067	PUR	Puerto Rican	AMR	American Ancestry	PUR	1000 Genomes on GRCh38,1000 Genomes 30x on GRCh38,1000 Genomes phase 3 release,1000 Genomes phase 1 release
**HG01349**	female	SAME124865	CLM	Colombian	AMR	American Ancestry	CLM	1000 Genomes 30x on GRCh38,1000 Genomes phase 3 release
**HG01254**	female	SAME125285	CLM	Colombian	AMR	American Ancestry	CLM	1000 Genomes on GRCh38,1000 Genomes 30x on GRCh38,1000 Genomes phase 3 release,1000 Genomes phase 1 release
**HG01351**	female	SAME124683	CLM	Colombian	AMR	American Ancestry	CLM	1000 Genomes on GRCh38,1000 Genomes 30x on GRCh38,1000 Genomes phase 3 release,1000 Genomes phase 1 release
**HG01259**	male	SAME125280	CLM	Colombian	AMR	American Ancestry	CLM	1000 Genomes on GRCh38,1000 Genomes 30x on GRCh38,1000 Genomes phase 3 release,1000 Genomes phase 1 release
**HG01356**	male	SAME124682	CLM	Colombian	AMR	American Ancestry	CLM	1000 Genomes on GRCh38,1000 Genomes 30x on GRCh38,1000 Genomes phase 3 release,1000 Genomes phase 1 release
**HG01261**	male	SAME122814	CLM	Colombian	AMR	American Ancestry	CLM	1000 Genomes 30x on GRCh38,1000 Genomes phase 3 release
**HG01363**	female	SAME1839059	CLM	Colombian	AMR	American Ancestry	CLM	1000 Genomes on GRCh38,1000 Genomes 30x on GRCh38,1000 Genomes phase 3 release
**HG01273**	male	SAME124046	CLM	Colombian	AMR	American Ancestry	CLM	1000 Genomes 30x on GRCh38,1000 Genomes phase 3 release
**HG01375**	female	SAME124290	CLM	Colombian	AMR	American Ancestry	CLM	1000 Genomes on GRCh38,1000 Genomes 30x on GRCh38,1000 Genomes phase 3 release,1000 Genomes phase 1 release
**HG01278**	female	SAME123003	CLM	Colombian	AMR	American Ancestry	CLM	1000 Genomes on GRCh38,1000 Genomes 30x on GRCh38,1000 Genomes phase 3 release,1000 Genomes phase 1 release
**HG01280**	male	SAME1839113	CLM	Colombian	AMR	American Ancestry	CLM	1000 Genomes on GRCh38,1000 Genomes 30x on GRCh38,1000 Genomes phase 3 release
**HG01402**	male	SAME1839873	PUR	Puerto Rican	AMR	American Ancestry	PUR	1000 Genomes on GRCh38,1000 Genomes 30x on GRCh38,1000 Genomes phase 3 release
**HG01083**	female	SAME125397	PUR	Puerto Rican	AMR	American Ancestry	PUR	1000 Genomes on GRCh38,1000 Genomes 30x on GRCh38,1000 Genomes phase 3 release,1000 Genomes phase 1 release
**HG01414**	female	SAME1839703	PUR	Puerto Rican	AMR	American Ancestry	PUR	1000 Genomes on GRCh38,1000 Genomes 30x on GRCh38,1000 Genomes phase 3 release
**HG01088**	male	SAME1839361	PUR	Puerto Rican	AMR	American Ancestry	PUR	1000 Genomes on GRCh38,1000 Genomes 30x on GRCh38,1000 Genomes phase 3 release
**HG01433**	male	SAME1839252	CLM	Colombian	AMR	American Ancestry	CLM	1000 Genomes 30x on GRCh38
**HG01438**	female	SAME124971	CLM	Colombian	AMR	American Ancestry	CLM	1000 Genomes on GRCh38,1000 Genomes 30x on GRCh38,1000 Genomes phase 3 release,1000 Genomes phase 1 release
**HG01095**	female	SAME122926	PUR	Puerto Rican	AMR	American Ancestry	PUR	1000 Genomes on GRCh38,1000 Genomes 30x on GRCh38,1000 Genomes phase 3 release,1000 Genomes phase 1 release
**HG01440**	male	SAME123237	CLM	Colombian	AMR	American Ancestry	CLM	1000 Genomes on GRCh38,1000 Genomes 30x on GRCh38,1000 Genomes phase 3 release,1000 Genomes phase 1 release
**HG01103**	female	SAME123679	PUR	Puerto Rican	AMR	American Ancestry	PUR	1000 Genomes 30x on GRCh38,1000 Genomes phase 3 release
**HG01281**	female	SAME1839107	CLM	Colombian	AMR	American Ancestry	CLM	1000 Genomes on GRCh38,1000 Genomes 30x on GRCh38,1000 Genomes phase 3 release
**HG01108**	female	SAME123103	PUR	Puerto Rican	AMR	American Ancestry	PUR	1000 Genomes on GRCh38,1000 Genomes 30x on GRCh38,1000 Genomes phase 3 release,1000 Genomes phase 1 release
**HG01286**	male	SAME1839176	PUR	Puerto Rican	AMR	American Ancestry	PUR	1000 Genomes on GRCh38,1000 Genomes 30x on GRCh38,1000 Genomes phase 3 release

**HG01110**	male	SAME123487	PUR	Puerto Rican	AMR	American Ancestry	PUR	1000 Genomes on GRCh38,1000 Genomes 30x on GRCh38,1000 Genomes phase 3 release,1000 Genomes phase 1 release
**HG01122**	female	SAME1840203	CLM	Colombian	AMR	American Ancestry	CLM	1000 Genomes on GRCh38,1000 Genomes 30x on GRCh38,1000 Genomes phase 3 release
**HG01325**	male	SAME1839604	PUR	Puerto Rican	AMR	American Ancestry	PUR	1000 Genomes on GRCh38,1000 Genomes 30x on GRCh38,1000 Genomes phase 3 release
**HG01350**	male	SAME124684	CLM	Colombian	AMR	American Ancestry	CLM	1000 Genomes on GRCh38,1000 Genomes 30x on GRCh38,1000 Genomes phase 3 release,1000 Genomes phase 1 release
**HG01344**	male	SAME124862	CLM	Colombian	AMR	American Ancestry	CLM	1000 Genomes on GRCh38,1000 Genomes 30x on GRCh38,1000 Genomes phase 3 release,1000 Genomes phase 1 release
**HG01355**	female	SAME124679	CLM	Colombian	AMR	American Ancestry	CLM	1000 Genomes 30x on GRCh38,1000 Genomes phase 3 release
**HG01604**	female	SAME123460	IBS	Iberian	EUR	European Ancestry	IBS	1000 Genomes 30x on GRCh38,1000 Genomes phase 3 release
**HG01362**	male	SAME1839066	CLM	Colombian	AMR	American Ancestry	CLM	1000 Genomes on GRCh38,1000 Genomes 30x on GRCh38,1000 Genomes phase 3 release
**HG01609**	male	SAME123461	IBS	Iberian	EUR	European Ancestry	IBS	1000 Genomes 30x on GRCh38,1000 Genomes phase 3 release
**HG01367**	female	SAME125266	CLM	Colombian	AMR	American Ancestry	CLM	1000 Genomes 30x on GRCh38,1000 Genomes phase 3 release
**HG01611**	male	SAME123624	IBS	Iberian	EUR	European Ancestry	IBS	1000 Genomes 30x on GRCh38,1000 Genomes phase 3 release
**HG01374**	male	SAME124291	CLM	Colombian	AMR	American Ancestry	CLM	1000 Genomes on GRCh38,1000 Genomes 30x on GRCh38,1000 Genomes phase 3 release,1000 Genomes phase 1 release
**HG01616**	female	SAME123629	IBS	Iberian	EUR	European Ancestry	IBS	1000 Genomes 30x on GRCh38,1000 Genomes phase 3 release
**HG01379**	female	SAME124283	CLM	Colombian	AMR	American Ancestry	CLM	1000 Genomes 30x on GRCh38,1000 Genomes phase 3 release
**HG01623**	female	SAME123845	IBS	Iberian	EUR	European Ancestry	IBS	1000 Genomes on GRCh38,1000 Genomes 30x on GRCh38,1000 Genomes phase 3 release,1000 Genomes phase 1 release
**HG01393**	female	SAME1840222	PUR	Puerto Rican	AMR	American Ancestry	PUR	1000 Genomes on GRCh38,1000 Genomes 30x on GRCh38,1000 Genomes phase 3 release
**HG01134**	female	SAME123887	CLM	Colombian	AMR	American Ancestry	CLM	1000 Genomes on GRCh38,1000 Genomes 30x on GRCh38,1000 Genomes phase 3 release,1000 Genomes phase 1 release
**HG01139**	male	SAME123876	CLM	Colombian	AMR	American Ancestry	CLM	1000 Genomes on GRCh38,1000 Genomes 30x on GRCh38,1000 Genomes phase 3 release,1000 Genomes phase 1 release
**HG01457**	male	SAME123428	CLM	Colombian	AMR	American Ancestry	CLM	1000 Genomes 30x on GRCh38,1000 Genomes phase 3 release
**HG01141**	female	SAME124423	CLM	Colombian	AMR	American Ancestry	CLM	1000 Genomes 30x on GRCh38,1000 Genomes phase 3 release
**HG01464**	male	SAME122883	CLM	Colombian	AMR	American Ancestry	CLM	1000 Genomes on GRCh38,1000 Genomes 30x on GRCh38,1000 Genomes phase 3 release,1000 Genomes phase 1 release
**HG01172**	male	SAME124625	PUR	Puerto Rican	AMR	American Ancestry	PUR	1000 Genomes 30x on GRCh38,1000 Genomes phase 3 release
**HG01177**	female	SAME124620	PUR	Puerto Rican	AMR	American Ancestry	PUR	1000 Genomes on GRCh38,1000 Genomes 30x on GRCh38,1000 Genomes phase 3 release,1000 Genomes phase 1 release
**HG01628**	female	SAME123837	IBS	Iberian	EUR	European Ancestry	IBS	1000 Genomes on GRCh38,1000 Genomes 30x on GRCh38,1000 Genomes phase 3 release
**HG01184**	female	SAME124321	PUR	Puerto Rican	AMR	American Ancestry	PUR	1000 Genomes 30x on GRCh38,1000 Genomes phase 3 release
**HG01630**	male	SAME124034	IBS	Iberian	EUR	European Ancestry	IBS	1000 Genomes on GRCh38,1000 Genomes 30x on GRCh38,1000 Genomes phase 3 release
**HG01189**	male	SAME124878	PUR	Puerto Rican	AMR	American Ancestry	PUR	1000 Genomes 30x on GRCh38,1000 Genomes phase 3 release
**HG01673**	female	SAME123289	IBS	Iberian	EUR	European Ancestry	IBS	1000 Genomes on GRCh38,1000 Genomes 30x on GRCh38,1000 Genomes phase 3 release
**HG01678**	male	SAME123292	IBS	Iberian	EUR	European Ancestry	IBS	1000 Genomes on GRCh38,1000 Genomes 30x on GRCh38,1000 Genomes phase 3 release
**HG01680**	male	SAME124656	IBS	Iberian	EUR	European Ancestry	IBS	1000 Genomes on GRCh38,1000 Genomes 30x on GRCh38,1000 Genomes phase 3 release
**HG01494**	male	SAME124135	CLM	Colombian	AMR	American Ancestry	CLM	1000 Genomes on GRCh38,1000 Genomes 30x on GRCh38,1000 Genomes phase 3 release,1000 Genomes phase 1 release
**HG01510**	female	SAME124771	IBS	Iberian	EUR	European Ancestry	IBS	1000 Genomes on GRCh38,1000 Genomes 30x on GRCh38,1000 Genomes phase 3 release
**HG01499**	male	SAME124143	CLM	Colombian	AMR	American Ancestry	CLM	1000 Genomes 30x on GRCh38,1000 Genomes phase 3 release
**HG01515**	male	SAME124774	IBS	Iberian	EUR	European Ancestry	IBS	1000 Genomes on GRCh38,1000 Genomes 30x on GRCh38,1000 Genomes phase 3 release,1000 Genomes phase 1 release
**HG01502**	male	SAME124943	IBS	Iberian	EUR	European Ancestry	IBS	1000 Genomes 30x on GRCh38,1000 Genomes phase 3 release
**HG01522**	female	SAME125321	IBS	Iberian	EUR	European Ancestry	IBS	1000 Genomes on GRCh38,1000 Genomes 30x on GRCh38,1000 Genomes phase 3 release,1000 Genomes phase 1 release
**HG01507**	female	SAME124948	IBS	Iberian	EUR	European Ancestry	IBS	1000 Genomes on GRCh38,1000 Genomes 30x on GRCh38,1000 Genomes phase 3 release
**HG01527**	male	SAME125326	IBS	Iberian	EUR	European Ancestry	IBS	1000 Genomes on GRCh38,1000 Genomes 30x on GRCh38,1000 Genomes phase 3 release
**HG01305**	male	SAME1840020	PUR	Puerto Rican	AMR	American Ancestry	PUR	1000 Genomes on GRCh38,1000 Genomes 30x on GRCh38,1000 Genomes phase 3 release
**HG01894**	female	SAME1840357	ACB	African Caribbean	AFR	African Ancestry	ACB	1000 Genomes on GRCh38,1000 Genomes 30x on GRCh38,1000 Genomes phase 3 release
**HG01312**	female	SAME1839791	PUR	Puerto Rican	AMR	American Ancestry	PUR	1000 Genomes on GRCh38,1000 Genomes 30x on GRCh38,1000 Genomes phase 3 release
**HG01914**	male	SAME1839768	ACB	African Caribbean	AFR	African Ancestry	ACB	1000 Genomes on GRCh38,1000 Genomes 30x on GRCh38,1000 Genomes phase 3 release
**HG01919**	female	SAME123239	PEL	Peruvian	AMR	American Ancestry	PEL	1000 Genomes 30x on GRCh38,1000 Genomes phase 3 release
**HG01343**	female	SAME124857	CLM	Colombian	AMR	American Ancestry	CLM	1000 Genomes 30x on GRCh38,1000 Genomes phase 3 release
**HG01921**	female	SAME123790	PEL	Peruvian	AMR	American Ancestry	PEL	1000 Genomes on GRCh38,1000 Genomes 30x on GRCh38,1000 Genomes phase 3 release
**HG01348**	female	SAME124864	CLM	Colombian	AMR	American Ancestry	CLM	1000 Genomes on GRCh38,1000 Genomes 30x on GRCh38,1000 Genomes phase 3 release
**HG01868**	female	SAME123193	KHV	Kinh Vietnamese	EAS	East Asian Ancestry	KHV	1000 Genomes on GRCh38,1000 Genomes 30x on GRCh38,1000 Genomes phase 3 release
**HG01398**	male	SAME1839950	PUR	Puerto Rican	AMR	American Ancestry	PUR	1000 Genomes on GRCh38,1000 Genomes 30x on GRCh38,1000 Genomes phase 3 release
**HG01870**	female	SAME123397	KHV	Kinh Vietnamese	EAS	East Asian Ancestry	KHV	1000 Genomes on GRCh38,1000 Genomes 30x on GRCh38,1000 Genomes phase 3 release
**HG01413**	male	SAME1839612	PUR	Puerto Rican	AMR	American Ancestry	PUR	1000 Genomes on GRCh38,1000 Genomes 30x on GRCh38,1000 Genomes phase 3 release
**HG01882**	male	SAME122849	ACB	African Caribbean	AFR	African Ancestry	ACB	1000 Genomes on GRCh38,1000 Genomes 30x on GRCh38,1000 Genomes phase 3 release
**HG01432**	female	SAME1839256	CLM	Colombian	AMR	American Ancestry	CLM	1000 Genomes on GRCh38,1000 Genomes 30x on GRCh38,1000 Genomes phase 3 release
**HG01887**	male	SAME122846	ACB	African Caribbean	AFR	African Ancestry	ACB	1000 Genomes 30x on GRCh38,1000 Genomes phase 3 release
**HG01437**	male	SAME124966	CLM	Colombian	AMR	American Ancestry	CLM	1000 Genomes on GRCh38,1000 Genomes 30x on GRCh38,1000 Genomes phase 3 release,1000 Genomes phase 1 release
**HG01926**	male	SAME123783	PEL	Peruvian	AMR	American Ancestry	PEL	1000 Genomes on GRCh38,1000 Genomes 30x on GRCh38,1000 Genomes phase 3 release
**HG01444**	female	SAME1839026	CLM	Colombian	AMR	American Ancestry	CLM	1000 Genomes on GRCh38,1000 Genomes 30x on GRCh38,1000 Genomes phase 3 release
**HG01933**	female	SAME123586	PEL	Peruvian	AMR	American Ancestry	PEL	1000 Genomes on GRCh38,1000 Genomes 30x on GRCh38,1000 Genomes phase 3 release
**HG01456**	female	SAME123427	CLM	Colombian	AMR	American Ancestry	CLM	1000 Genomes on GRCh38,1000 Genomes 30x on GRCh38,1000 Genomes phase 3 release,1000 Genomes phase 1 release
**HG01938**	male	SAME123593	PEL	Peruvian	AMR	American Ancestry	PEL	1000 Genomes on GRCh38,1000 Genomes 30x on GRCh38,1000 Genomes phase 3 release
**HG01463**	female	SAME123537	CLM	Colombian	AMR	American Ancestry	CLM	1000 Genomes 30x on GRCh38,1000 Genomes phase 3 release
**HG01940**	female	SAME124148	PEL	Peruvian	AMR	American Ancestry	PEL	1000 Genomes 30x on GRCh38,1000 Genomes phase 3 release
**HG01468**	female	SAME1840382	CLM	Colombian	AMR	American Ancestry	CLM	1000 Genomes on GRCh38,1000 Genomes 30x on GRCh38,1000 Genomes phase 3 release
**HG01945**	female	SAME124153	PEL	Peruvian	AMR	American Ancestry	PEL	1000 Genomes on GRCh38,1000 Genomes 30x on GRCh38,1000 Genomes phase 3 release
**HG01514**	female	SAME124775	IBS	Iberian	EUR	European Ancestry	IBS	1000 Genomes 30x on GRCh38,1000 Genomes phase 3 release
**HG01748**	male	SAME125191	IBS	Iberian	EUR	European Ancestry	IBS	1000 Genomes 30x on GRCh38,1000 Genomes phase 3 release
**HG01519**	female	SAME124765	IBS	Iberian	EUR	European Ancestry	IBS	1000 Genomes on GRCh38,1000 Genomes 30x on GRCh38,1000 Genomes phase 3 release,1000 Genomes phase 1 release
**HG01755**	female	SAME124986	IBS	Iberian	EUR	European Ancestry	IBS	1000 Genomes 30x on GRCh38,1000 Genomes phase 3 release
**HG01521**	male	SAME125324	IBS	Iberian	EUR	European Ancestry	IBS	1000 Genomes on GRCh38,1000 Genomes 30x on GRCh38,1000 Genomes phase 3 release,1000 Genomes phase 1 release
**HG01762**	female	SAME124811	IBS	Iberian	EUR	European Ancestry	IBS	1000 Genomes on GRCh38,1000 Genomes 30x on GRCh38,1000 Genomes phase 3 release
**HG01526**	male	SAME125325	IBS	Iberian	EUR	European Ancestry	IBS	1000 Genomes 30x on GRCh38,1000 Genomes phase 3 release
**HG01767**	male	SAME1839671	IBS	Iberian	EUR	European Ancestry	IBS	1000 Genomes on GRCh38,1000 Genomes 30x on GRCh38,1000 Genomes phase 3 release
**HG01538**	female	SAME125141	IBS	Iberian	EUR	European Ancestry	IBS	1000 Genomes 30x on GRCh38,1000 Genomes phase 3 release
**HG01774**	male	SAME124634	IBS	Iberian	EUR	European Ancestry	IBS	1000 Genomes 30x on GRCh38,1000 Genomes phase 3 release
**HG01552**	male	SAME124007	CLM	Colombian	AMR	American Ancestry	CLM	1000 Genomes 30x on GRCh38,1000 Genomes phase 3 release
**HG01779**	female	SAME124629	IBS	Iberian	EUR	European Ancestry	IBS	1000 Genomes on GRCh38,1000 Genomes 30x on GRCh38,1000 Genomes phase 3 release
**HG01571**	male	SAME124346	PEL	Peruvian	AMR	American Ancestry	PEL	1000 Genomes on GRCh38,1000 Genomes 30x on GRCh38,1000 Genomes phase 3 release
**HG01781**	male	SAME124921	IBS	Iberian	EUR	European Ancestry	IBS	1000 Genomes on GRCh38,1000 Genomes 30x on GRCh38,1000 Genomes phase 3 release
**HG01583**	male	SAME1839895	PJL	Punjabi	SAS	South Asian Ancestry	PJL	1000 Genomes on GRCh38,1000 Genomes 30x on GRCh38,1000 Genomes phase 3 release
**HG01786**	female	SAME1839312	IBS	Iberian	EUR	European Ancestry	IBS	1000 Genomes on GRCh38,1000 Genomes 30x on GRCh38,1000 Genomes phase 3 release
**HG01952**	male	SAME123975	PEL	Peruvian	AMR	American Ancestry	PEL	1000 Genomes 30x on GRCh38,1000 Genomes phase 3 release
**HG01595**	female	SAME123569	KHV	Kinh Vietnamese	EAS	East Asian Ancestry	KHV	1000 Genomes on GRCh38,1000 Genomes 30x on GRCh38,1000 Genomes phase 3 release
**HG01969**	female	SAME124533	PEL	Peruvian	AMR	American Ancestry	PEL	1000 Genomes 30x on GRCh38,1000 Genomes phase 3 release
**HG01867**	male	SAME123194	KHV	Kinh Vietnamese	EAS	East Asian Ancestry	KHV	1000 Genomes on GRCh38,1000 Genomes 30x on GRCh38,1000 Genomes phase 3 release
**HG01971**	female	SAME124326	PEL	Peruvian	AMR	American Ancestry	PEL	1000 Genomes on GRCh38,1000 Genomes 30x on GRCh38,1000 Genomes phase 3 release
**HG01874**	female	SAME123396	KHV	Kinh Vietnamese	EAS	East Asian Ancestry	KHV	1000 Genomes on GRCh38,1000 Genomes 30x on GRCh38,1000 Genomes phase 3 release
**HG01685**	female	SAME124659	IBS	Iberian	EUR	European Ancestry	IBS	1000 Genomes on GRCh38,1000 Genomes 30x on GRCh38,1000 Genomes phase 3 release
**HG01879**	male	SAME1839037	ACB	African Caribbean	AFR	African Ancestry	ACB	1000 Genomes on GRCh38,1000 Genomes 30x on GRCh38,1000 Genomes phase 3 release
**HG01697**	female	SAME1839223	IBS	Iberian	EUR	European Ancestry	IBS	1000 Genomes on GRCh38,1000 Genomes 30x on GRCh38,1000 Genomes phase 3 release
**HG01881**	female	SAME1839193	ACB	African Caribbean	AFR	African Ancestry	ACB	1000 Genomes 30x on GRCh38,1000 Genomes phase 3 release
**HG01700**	male	SAME123261	IBS	Iberian	EUR	European Ancestry	IBS	1000 Genomes on GRCh38,1000 Genomes 30x on GRCh38,1000 Genomes phase 3 release
**HG01886**	female	SAME122847	ACB	African Caribbean	AFR	African Ancestry	ACB	1000 Genomes on GRCh38,1000 Genomes 30x on GRCh38,1000 Genomes phase 3 release
**HG01705**	male	SAME123266	IBS	Iberian	EUR	European Ancestry	IBS	1000 Genomes on GRCh38,1000 Genomes 30x on GRCh38,1000 Genomes phase 3 release
**HG01709**	male	SAME123229	IBS	Iberian	EUR	European Ancestry	IBS	1000 Genomes on GRCh38,1000 Genomes 30x on GRCh38,1000 Genomes phase 3 release
**HG01844**	male	SAME123556	KHV	Kinh Vietnamese	EAS	East Asian Ancestry	KHV	1000 Genomes on GRCh38,1000 Genomes 30x on GRCh38,1000 Genomes phase 3 release
**HG01711**	female	SAME123083	IBS	Iberian	EUR	European Ancestry	IBS	1000 Genomes 30x on GRCh38,1000 Genomes phase 3 release
**HG01849**	male	SAME125110	KHV	Kinh Vietnamese	EAS	East Asian Ancestry	KHV	1000 Genomes on GRCh38,1000 Genomes 30x on GRCh38,1000 Genomes phase 3 release
**HG01747**	male	SAME125181	IBS	Iberian	EUR	European Ancestry	IBS	1000 Genomes on GRCh38,1000 Genomes 30x on GRCh38,1000 Genomes phase 3 release
**HG01851**	female	SAME123753	KHV	Kinh Vietnamese	EAS	East Asian Ancestry	KHV	1000 Genomes on GRCh38,1000 Genomes 30x on GRCh38,1000 Genomes phase 3 release
**HG01761**	male	SAME124810	IBS	Iberian	EUR	European Ancestry	IBS	1000 Genomes on GRCh38,1000 Genomes 30x on GRCh38,1000 Genomes phase 3 release
**HG01863**	female	SAME123198	KHV	Kinh Vietnamese	EAS	East Asian Ancestry	KHV	1000 Genomes on GRCh38,1000 Genomes 30x on GRCh38,1000 Genomes phase 3 release
**HG01766**	female	SAME1839673	IBS	Iberian	EUR	European Ancestry	IBS	1000 Genomes on GRCh38,1000 Genomes 30x on GRCh38,1000 Genomes phase 3 release
**HG01798**	female	SAME124228	CDX	Dai Chinese	EAS	East Asian Ancestry	CDX	1000 Genomes on GRCh38,1000 Genomes 30x on GRCh38,1000 Genomes phase 3 release
**HG01773**	female	SAME124635	IBS	Iberian	EUR	European Ancestry	IBS	1000 Genomes on GRCh38,1000 Genomes 30x on GRCh38,1000 Genomes phase 3 release
**HG01801**	female	SAME124305	CDX	Dai Chinese	EAS	East Asian Ancestry	CDX	1000 Genomes on GRCh38,1000 Genomes 30x on GRCh38,1000 Genomes phase 3 release
**HG01603**	male	SAME123453	IBS	Iberian	EUR	European Ancestry	IBS	1000 Genomes on GRCh38,1000 Genomes 30x on GRCh38,1000 Genomes phase 3 release
**HG01806**	female	SAME124304	CDX	Dai Chinese	EAS	East Asian Ancestry	CDX	1000 Genomes on GRCh38,1000 Genomes 30x on GRCh38,1000 Genomes phase 3 release
**HG01608**	male	SAME123462	IBS	Iberian	EUR	European Ancestry	IBS	1000 Genomes on GRCh38,1000 Genomes 30x on GRCh38,1000 Genomes phase 3 release
**HG01813**	female	SAME124518	CDX	Dai Chinese	EAS	East Asian Ancestry	CDX	1000 Genomes on GRCh38,1000 Genomes 30x on GRCh38,1000 Genomes phase 3 release
**HG01610**	male	SAME123623	IBS	Iberian	EUR	European Ancestry	IBS	1000 Genomes on GRCh38,1000 Genomes 30x on GRCh38,1000 Genomes phase 3 release
**HG02006**	female	SAME1840279	PEL	Peruvian	AMR	American Ancestry	PEL	1000 Genomes on GRCh38,1000 Genomes 30x on GRCh38,1000 Genomes phase 3 release
**HG01615**	male	SAME123628	IBS	Iberian	EUR	European Ancestry	IBS	1000 Genomes on GRCh38,1000 Genomes 30x on GRCh38,1000 Genomes phase 3 release

**HG02013**	male	SAME1840396	ACB	African Caribbean	AFR	African Ancestry	ACB	1000 Genomes on GRCh38,1000 Genomes 30x on GRCh38,1000 Genomes phase 3 release
**HG01622**	female	SAME123846	IBS	Iberian	EUR	European Ancestry	IBS	1000 Genomes 30x on GRCh38,1000 Genomes phase 3 release
**HG02018**	female	SAME123451	KHV	Kinh Vietnamese	EAS	East Asian Ancestry	KHV	1000 Genomes 30x on GRCh38,Human Genome Structural Variation Consortium, Phase 2,1000 Genomes phase 3 release
**HG01627**	male	SAME123841	IBS	Iberian	EUR	European Ancestry	IBS	1000 Genomes 30x on GRCh38,1000 Genomes phase 3 release
**HG02020**	male	SAME125168	KHV	Kinh Vietnamese	EAS	East Asian Ancestry	KHV	1000 Genomes on GRCh38,1000 Genomes 30x on GRCh38,1000 Genomes phase 3 release
**HG01778**	male	SAME124630	IBS	Iberian	EUR	European Ancestry	IBS	1000 Genomes 30x on GRCh38,1000 Genomes phase 3 release
**HG01976**	female	SAME124325	PEL	Peruvian	AMR	American Ancestry	PEL	1000 Genomes on GRCh38,1000 Genomes 30x on GRCh38,1000 Genomes phase 3 release
**HG01780**	male	SAME124428	IBS	Iberian	EUR	European Ancestry	IBS	1000 Genomes 30x on GRCh38,1000 Genomes phase 3 release
**HG01983**	female	SAME1840106	PEL	Peruvian	AMR	American Ancestry	PEL	1000 Genomes on GRCh38,1000 Genomes 30x on GRCh38,1000 Genomes phase 3 release
**HG01785**	male	SAME1839313	IBS	Iberian	EUR	European Ancestry	IBS	1000 Genomes on GRCh38,1000 Genomes 30x on GRCh38,1000 Genomes phase 3 release
**HG01988**	male	SAME1840175	ACB	African Caribbean	AFR	African Ancestry	ACB	1000 Genomes on GRCh38,1000 Genomes 30x on GRCh38,1000 Genomes phase 3 release
**HG01797**	female	SAME124219	CDX	Dai Chinese	EAS	East Asian Ancestry	CDX	1000 Genomes on GRCh38,1000 Genomes 30x on GRCh38,1000 Genomes phase 3 release
**HG01990**	male	SAME1840235	ACB	African Caribbean	AFR	African Ancestry	ACB	1000 Genomes on GRCh38,1000 Genomes 30x on GRCh38,1000 Genomes phase 3 release
**HG01684**	female	SAME124660	IBS	Iberian	EUR	European Ancestry	IBS	1000 Genomes on GRCh38,1000 Genomes 30x on GRCh38,1000 Genomes phase 3 release
**HG01696**	male	SAME124715	IBS	Iberian	EUR	European Ancestry	IBS	1000 Genomes 30x on GRCh38,1000 Genomes phase 3 release
**HG02025**	female	SAME125335	KHV	Kinh Vietnamese	EAS	East Asian Ancestry	KHV	1000 Genomes on GRCh38,1000 Genomes 30x on GRCh38,1000 Genomes phase 3 release
**HG01704**	female	SAME123265	IBS	Iberian	EUR	European Ancestry	IBS	1000 Genomes on GRCh38,1000 Genomes 30x on GRCh38,1000 Genomes phase 3 release
**HG02032**	male	SAME1839959	KHV	Kinh Vietnamese	EAS	East Asian Ancestry	KHV	1000 Genomes on GRCh38,1000 Genomes 30x on GRCh38,1000 Genomes phase 3 release
**HG01843**	female	SAME123553	KHV	Kinh Vietnamese	EAS	East Asian Ancestry	KHV	1000 Genomes on GRCh38,1000 Genomes 30x on GRCh38,1000 Genomes phase 3 release
**HG02049**	female	SAME123773	KHV	Kinh Vietnamese	EAS	East Asian Ancestry	KHV	1000 Genomes on GRCh38,1000 Genomes 30x on GRCh38,1000 Genomes phase 3 release
**HG01848**	female	SAME123548	KHV	Kinh Vietnamese	EAS	East Asian Ancestry	KHV	1000 Genomes on GRCh38,1000 Genomes 30x on GRCh38,1000 Genomes phase 3 release
**HG02051**	male	SAME1839549	ACB	African Caribbean	AFR	African Ancestry	ACB	1000 Genomes on GRCh38,1000 Genomes 30x on GRCh38,1000 Genomes phase 3 release
**HG01850**	female	SAME123752	KHV	Kinh Vietnamese	EAS	East Asian Ancestry	KHV	1000 Genomes on GRCh38,1000 Genomes 30x on GRCh38,1000 Genomes phase 3 release
**HG02082**	male	SAME124950	KHV	Kinh Vietnamese	EAS	East Asian Ancestry	KHV	1000 Genomes on GRCh38,1000 Genomes 30x on GRCh38,1000 Genomes phase 3 release
**HG01855**	female	SAME123749	KHV	Kinh Vietnamese	EAS	East Asian Ancestry	KHV	1000 Genomes on GRCh38,1000 Genomes 30x on GRCh38,1000 Genomes phase 3 release
**HG02087**	female	SAME124678	KHV	Kinh Vietnamese	EAS	East Asian Ancestry	KHV	1000 Genomes on GRCh38,1000 Genomes 30x on GRCh38,1000 Genomes phase 3 release
**HG01862**	female	SAME123199	KHV	Kinh Vietnamese	EAS	East Asian Ancestry	KHV	1000 Genomes on GRCh38,1000 Genomes 30x on GRCh38,1000 Genomes phase 3 release
**HG02102**	female	SAME1840155	PEL	Peruvian	AMR	American Ancestry	PEL	1000 Genomes on GRCh38,1000 Genomes 30x on GRCh38,1000 Genomes phase 3 release
**HG01672**	male	SAME123288	IBS	Iberian	EUR	European Ancestry	IBS	1000 Genomes on GRCh38,1000 Genomes 30x on GRCh38,1000 Genomes phase 3 release
**HG02107**	male	SAME1840206	ACB	African Caribbean	AFR	African Ancestry	ACB	1000 Genomes on GRCh38,1000 Genomes 30x on GRCh38,1000 Genomes phase 3 release
**HG01677**	female	SAME123285	IBS	Iberian	EUR	European Ancestry	IBS	1000 Genomes 30x on GRCh38,1000 Genomes phase 3 release
**HG02145**	male	SAME1839616	ACB	African Caribbean	AFR	African Ancestry	ACB	1000 Genomes 30x on GRCh38,1000 Genomes phase 3 release
**HG01800**	female	SAME124306	CDX	Dai Chinese	EAS	East Asian Ancestry	CDX	1000 Genomes on GRCh38,1000 Genomes 30x on GRCh38,1000 Genomes phase 3 release
**HG02152**	female	SAME124790	CDX	Dai Chinese	EAS	East Asian Ancestry	CDX	1000 Genomes on GRCh38,1000 Genomes 30x on GRCh38,1000 Genomes phase 3 release
**HG01805**	female	SAME124301	CDX	Dai Chinese	EAS	East Asian Ancestry	CDX	1000 Genomes on GRCh38,1000 Genomes 30x on GRCh38,1000 Genomes phase 3 release
**HG02164**	female	SAME124209	CDX	Dai Chinese	EAS	East Asian Ancestry	CDX	1000 Genomes on GRCh38,1000 Genomes 30x on GRCh38,1000 Genomes phase 3 release
**HG01812**	female	SAME124517	CDX	Dai Chinese	EAS	East Asian Ancestry	CDX	1000 Genomes on GRCh38,1000 Genomes 30x on GRCh38,1000 Genomes phase 3 release
**HG01817**	female	SAME124522	CDX	Dai Chinese	EAS	East Asian Ancestry	CDX	1000 Genomes on GRCh38,1000 Genomes 30x on GRCh38,1000 Genomes phase 3 release
**HG02121**	female	SAME123479	KHV	Kinh Vietnamese	EAS	East Asian Ancestry	KHV	1000 Genomes on GRCh38,1000 Genomes 30x on GRCh38,1000 Genomes phase 3 release
**HG02079**	male	SAME123098	KHV	Kinh Vietnamese	EAS	East Asian Ancestry	KHV	1000 Genomes on GRCh38,1000 Genomes 30x on GRCh38,1000 Genomes phase 3 release
**HG02126**	female	SAME123477	KHV	Kinh Vietnamese	EAS	East Asian Ancestry	KHV	1000 Genomes 30x on GRCh38,1000 Genomes phase 3 release
**HG02081**	female	SAME124826	KHV	Kinh Vietnamese	EAS	East Asian Ancestry	KHV	1000 Genomes on GRCh38,1000 Genomes 30x on GRCh38,1000 Genomes phase 3 release
**HG02133**	female	SAME1839882	KHV	Kinh Vietnamese	EAS	East Asian Ancestry	KHV	1000 Genomes on GRCh38,1000 Genomes 30x on GRCh38,1000 Genomes phase 3 release
**HG02086**	female	SAME124675	KHV	Kinh Vietnamese	EAS	East Asian Ancestry	KHV	1000 Genomes on GRCh38,1000 Genomes 30x on GRCh38,1000 Genomes phase 3 release
**HG02138**	male	SAME123659	KHV	Kinh Vietnamese	EAS	East Asian Ancestry	KHV	1000 Genomes on GRCh38,1000 Genomes 30x on GRCh38,1000 Genomes phase 3 release
**HG02106**	female	SAME123875	PEL	Peruvian	AMR	American Ancestry	PEL	1000 Genomes 30x on GRCh38,Human Genome Structural Variation Consortium, Phase 2,1000 Genomes phase 3 release
**HG02140**	female	SAME124612	KHV	Kinh Vietnamese	EAS	East Asian Ancestry	KHV	1000 Genomes on GRCh38,1000 Genomes 30x on GRCh38,1000 Genomes phase 3 release
**HG01949**	female	SAME124145	PEL	Peruvian	AMR	American Ancestry	PEL	1000 Genomes 30x on GRCh38,1000 Genomes phase 3 release
**HG02222**	female	SAME123717	IBS	Iberian	EUR	European Ancestry	IBS	1000 Genomes 30x on GRCh38,1000 Genomes phase 3 release
**HG01951**	female	SAME123976	PEL	Peruvian	AMR	American Ancestry	PEL	1000 Genomes on GRCh38,1000 Genomes 30x on GRCh38,1000 Genomes phase 3 release
**HG02234**	male	SAME1839367	IBS	Iberian	EUR	European Ancestry	IBS	1000 Genomes 30x on GRCh38,1000 Genomes phase 3 release
**HG01956**	female	SAME123978	ACB	African Caribbean	AFR	African Ancestry	ACB	1000 Genomes on GRCh38,1000 Genomes 30x on GRCh38,1000 Genomes phase 3 release
**HG02239**	female	SAME1839420	IBS	Iberian	EUR	European Ancestry	IBS	1000 Genomes on GRCh38,1000 Genomes 30x on GRCh38,1000 Genomes phase 3 release
**HG01968**	female	SAME124532	PEL	Peruvian	AMR	American Ancestry	PEL	1000 Genomes on GRCh38,1000 Genomes 30x on GRCh38,1000 Genomes phase 3 release
**HG02253**	male	SAME1839347	PEL	Peruvian	AMR	American Ancestry	PEL	1000 Genomes on GRCh38,1000 Genomes 30x on GRCh38,1000 Genomes phase 3 release
**HG01970**	male	SAME124327	PEL	Peruvian	AMR	American Ancestry	PEL	1000 Genomes on GRCh38,1000 Genomes 30x on GRCh38,1000 Genomes phase 3 release
**HG02056**	male	SAME124162	KHV	Kinh Vietnamese	EAS	East Asian Ancestry	KHV	1000 Genomes 30x on GRCh38,1000 Genomes phase 3 release
**HG02024**	female	SAME125182	KHV	Kinh Vietnamese	EAS	East Asian Ancestry	KHV	1000 Genomes on GRCh38,1000 Genomes 30x on GRCh38,1000 Genomes phase 3 release
**HG02068**	female	SAME123374	KHV	Kinh Vietnamese	EAS	East Asian Ancestry	KHV	1000 Genomes 30x on GRCh38,1000 Genomes phase 3 release
**HG02029**	male	SAME124021	KHV	Kinh Vietnamese	EAS	East Asian Ancestry	KHV	1000 Genomes on GRCh38,1000 Genomes 30x on GRCh38,1000 Genomes phase 3 release
**HG02070**	male	SAME124908	KHV	Kinh Vietnamese	EAS	East Asian Ancestry	KHV	1000 Genomes on GRCh38,1000 Genomes 30x on GRCh38,1000 Genomes phase 3 release
**HG02031**	female	SAME1839964	KHV	Kinh Vietnamese	EAS	East Asian Ancestry	KHV	1000 Genomes on GRCh38,1000 Genomes 30x on GRCh38,1000 Genomes phase 3 release
**HG02075**	female	SAME123106	KHV	Kinh Vietnamese	EAS	East Asian Ancestry	KHV	1000 Genomes on GRCh38,1000 Genomes 30x on GRCh38,1000 Genomes phase 3 release
**HG02048**	female	SAME123772	KHV	Kinh Vietnamese	EAS	East Asian Ancestry	KHV	1000 Genomes on GRCh38,1000 Genomes 30x on GRCh38,1000 Genomes phase 3 release
**HG02050**	male	SAME1839555	KHV	Kinh Vietnamese	EAS	East Asian Ancestry	KHV	1000 Genomes on GRCh38,1000 Genomes 30x on GRCh38,1000 Genomes phase 3 release
**HG02188**	female	SAME122829	CDX	Dai Chinese	EAS	East Asian Ancestry	CDX	1000 Genomes on GRCh38,1000 Genomes 30x on GRCh38,1000 Genomes phase 3 release
**HG02055**	male	SAME1839531	ACB	African Caribbean	AFR	African Ancestry	ACB	1000 Genomes 30x on GRCh38,1000 Genomes phase 3 release
**HG02190**	female	SAME125259	CDX	Dai Chinese	EAS	East Asian Ancestry	CDX	1000 Genomes on GRCh38,1000 Genomes 30x on GRCh38,1000 Genomes phase 3 release
**HG02067**	male	SAME1839388	KHV	Kinh Vietnamese	EAS	East Asian Ancestry	KHV	1000 Genomes on GRCh38,1000 Genomes 30x on GRCh38,1000 Genomes phase 3 release
**HG02215**	female	SAME123904	GBR	British	EUR	European Ancestry	GBR	1000 Genomes on GRCh38,1000 Genomes 30x on GRCh38,1000 Genomes phase 3 release,Geuvadis
**HG02074**	male	SAME123107	KHV	Kinh Vietnamese	EAS	East Asian Ancestry	KHV	1000 Genomes 30x on GRCh38,1000 Genomes phase 3 release
**HG02563**	male	SAME1839824	GWD	Gambian Mandinka	AFR	African Ancestry	GWD	1000 Genomes 30x on GRCh38
**HG01925**	female	SAME123786	PEL	Peruvian	AMR	American Ancestry	PEL	1000 Genomes 30x on GRCh38,1000 Genomes phase 3 release
**HG02568**	female	SAME1839899	GWD	Gambian Mandinka	AFR	African Ancestry	GWD	1000 Genomes on GRCh38,1000 Genomes 30x on GRCh38,1000 Genomes phase 3 release,Gambian Genome Variation Project (GRCh38)
**HG01932**	male	SAME123587	PEL	Peruvian	AMR	American Ancestry	PEL	1000 Genomes on GRCh38,1000 Genomes 30x on GRCh38,1000 Genomes phase 3 release
**HG02570**	male	SAME1839582	GWD	Gambian Mandinka	AFR	African Ancestry	GWD	1000 Genomes on GRCh38,1000 Genomes 30x on GRCh38,1000 Genomes phase 3 release,Gambian Genome Variation Project (GRCh38)
**HG01937**	male	SAME123590	PEL	Peruvian	AMR	American Ancestry	PEL	1000 Genomes 30x on GRCh38,1000 Genomes phase 3 release
**HG02575**	male	SAME1839650	GWD	Gambian Mandinka	AFR	African Ancestry	GWD	1000 Genomes 30x on GRCh38
**HG01944**	male	SAME124152	PEL	Peruvian	AMR	American Ancestry	PEL	1000 Genomes on GRCh38,1000 Genomes 30x on GRCh38,1000 Genomes phase 3 release
**HG02582**	male	SAME1839431	GWD	Gambian Mandinka	AFR	African Ancestry	GWD	1000 Genomes on GRCh38,1000 Genomes 30x on GRCh38,1000 Genomes phase 3 release,Gambian Genome Variation Project (GRCh38)
**HG01975**	female	SAME124322	PEL	Peruvian	AMR	American Ancestry	PEL	1000 Genomes 30x on GRCh38,1000 Genomes phase 3 release
**HG02587**	female	SAME1839409	GWD	Gambian Mandinka	AFR	African Ancestry	GWD	1000 Genomes 30x on GRCh38,Human Genome Structural Variation Consortium, Phase 2
**HG01982**	male	SAME1840111	PEL	Peruvian	AMR	American Ancestry	PEL	1000 Genomes on GRCh38,1000 Genomes 30x on GRCh38,1000 Genomes phase 3 release
**HG02594**	male	SAME1839208	GWD	Gambian Mandinka	AFR	African Ancestry	GWD	1000 Genomes on GRCh38,1000 Genomes 30x on GRCh38,1000 Genomes phase 3 release,Gambian Genome Variation Project (GRCh38)
**HG01987**	male	SAME1840089	ACB	African Caribbean	AFR	African Ancestry	ACB	1000 Genomes 30x on GRCh38,1000 Genomes phase 3 release
**HG02113**	female	SAME124044	KHV	Kinh Vietnamese	EAS	East Asian Ancestry	KHV	1000 Genomes on GRCh38,1000 Genomes 30x on GRCh38,1000 Genomes phase 3 release
**HG02602**	male	SAME1840290	PJL	Punjabi	SAS	South Asian Ancestry	PJL	1000 Genomes 30x on GRCh38,1000 Genomes phase 3 release
**HG02120**	male	SAME123480	KHV	Kinh Vietnamese	EAS	East Asian Ancestry	KHV	1000 Genomes 30x on GRCh38,1000 Genomes phase 3 release
**HG02429**	male	SAME1839021	ACB	African Caribbean	AFR	African Ancestry	ACB	1000 Genomes on GRCh38,1000 Genomes 30x on GRCh38,1000 Genomes phase 3 release
**HG02132**	male	SAME1839889	KHV	Kinh Vietnamese	EAS	East Asian Ancestry	KHV	1000 Genomes 30x on GRCh38,1000 Genomes phase 3 release
**HG02137**	male	SAME1839862	KHV	Kinh Vietnamese	EAS	East Asian Ancestry	KHV	1000 Genomes on GRCh38,1000 Genomes 30x on GRCh38,1000 Genomes phase 3 release
**HG02450**	female	SAME1840144	ACB	African Caribbean	AFR	African Ancestry	ACB	1000 Genomes on GRCh38,1000 Genomes 30x on GRCh38,1000 Genomes phase 3 release
**HG01893**	female	SAME123027	PEL	Peruvian	AMR	American Ancestry	PEL	1000 Genomes on GRCh38,1000 Genomes 30x on GRCh38,1000 Genomes phase 3 release
**HG02455**	male	SAME1840198	ACB	African Caribbean	AFR	African Ancestry	ACB	1000 Genomes on GRCh38,1000 Genomes 30x on GRCh38,1000 Genomes phase 3 release
**HG01898**	female	SAME124383	PEL	Peruvian	AMR	American Ancestry	PEL	1000 Genomes 30x on GRCh38,1000 Genomes phase 3 release
**HG02462**	female	SAME1839991	GWD	Gambian Mandinka	AFR	African Ancestry	GWD	1000 Genomes on GRCh38,1000 Genomes 30x on GRCh38,1000 Genomes phase 3 release,Gambian Genome Variation Project (GRCh38)
**HG01918**	female	SAME123240	PEL	Peruvian	AMR	American Ancestry	PEL	1000 Genomes on GRCh38,1000 Genomes 30x on GRCh38,1000 Genomes phase 3 release
**HG02258**	male	SAME1839322	ACB	African Caribbean	AFR	African Ancestry	ACB	1000 Genomes 30x on GRCh38,1000 Genomes phase 3 release
**HG01920**	male	SAME123789	PEL	Peruvian	AMR	American Ancestry	PEL	1000 Genomes on GRCh38,1000 Genomes 30x on GRCh38,1000 Genomes phase 3 release
**HG02260**	female	SAME1839139	PEL	Peruvian	AMR	American Ancestry	PEL	1000 Genomes on GRCh38,1000 Genomes 30x on GRCh38,1000 Genomes phase 3 release
**HG02012**	female	SAME1840403	ACB	African Caribbean	AFR	African Ancestry	ACB	1000 Genomes on GRCh38,1000 Genomes 30x on GRCh38,1000 Genomes phase 3 release
**HG02265**	male	SAME1839117	PEL	Peruvian	AMR	American Ancestry	PEL	1000 Genomes on GRCh38,1000 Genomes 30x on GRCh38,1000 Genomes phase 3 release
**HG02017**	male	SAME123445	KHV	Kinh Vietnamese	EAS	East Asian Ancestry	KHV	1000 Genomes on GRCh38,1000 Genomes 30x on GRCh38,1000 Genomes phase 3 release
**HG02272**	female	SAME1840314	PEL	Peruvian	AMR	American Ancestry	PEL	1000 Genomes on GRCh38,1000 Genomes 30x on GRCh38,1000 Genomes phase 3 release
**HG02277**	male	SAME1840370	PEL	Peruvian	AMR	American Ancestry	PEL	1000 Genomes on GRCh38,1000 Genomes 30x on GRCh38,1000 Genomes phase 3 release
**HG02442**	male	SAME1840352	ACB	African Caribbean	AFR	African Ancestry	ACB	1000 Genomes on GRCh38,1000 Genomes 30x on GRCh38,1000 Genomes phase 3 release
**HG02479**	female	SAME1839798	ACB	African Caribbean	AFR	African Ancestry	ACB	1000 Genomes on GRCh38,1000 Genomes 30x on GRCh38,1000 Genomes phase 3 release
**HG02461**	male	SAME1839993	GWD	Gambian Mandinka	AFR	African Ancestry	GWD	1000 Genomes on GRCh38,1000 Genomes 30x on GRCh38,1000 Genomes phase 3 release,Gambian Genome Variation Project (GRCh38)
**HG02481**	male	SAME1839870	ACB	African Caribbean	AFR	African Ancestry	ACB	1000 Genomes on GRCh38,1000 Genomes 30x on GRCh38,1000 Genomes phase 3 release
**HG02466**	male	SAME1839970	GWD	Gambian Mandinka	AFR	African Ancestry	GWD	1000 Genomes 30x on GRCh38
**HG02493**	male	SAME1839704	PJL	Punjabi	SAS	South Asian Ancestry	PJL	1000 Genomes on GRCh38,1000 Genomes 30x on GRCh38,1000 Genomes phase 3 release

**HG02182**	female	SAME125035	CDX	Dai Chinese	EAS	East Asian Ancestry	CDX	1000 Genomes on GRCh38,1000 Genomes 30x on GRCh38,1000 Genomes phase 3 release
**HG02501**	male	SAME1839281	ACB	African Caribbean	AFR	African Ancestry	ACB	1000 Genomes on GRCh38,1000 Genomes 30x on GRCh38,1000 Genomes phase 3 release
**HG02187**	female	SAME125038	CDX	Dai Chinese	EAS	East Asian Ancestry	CDX	1000 Genomes on GRCh38,1000 Genomes 30x on GRCh38,1000 Genomes phase 3 release
**HG02513**	female	SAME1839138	KHV	Kinh Vietnamese	EAS	East Asian Ancestry	KHV	1000 Genomes on GRCh38,1000 Genomes 30x on GRCh38,1000 Genomes phase 3 release
**HG02219**	male	SAME1839813	IBS	Iberian	EUR	European Ancestry	IBS	1000 Genomes on GRCh38,1000 Genomes 30x on GRCh38,1000 Genomes phase 3 release
**HG02525**	female	SAME1840313	KHV	Kinh Vietnamese	EAS	East Asian Ancestry	KHV	1000 Genomes on GRCh38,1000 Genomes 30x on GRCh38
**HG02221**	male	SAME123716	IBS	Iberian	EUR	European Ancestry	IBS	1000 Genomes on GRCh38,1000 Genomes 30x on GRCh38,1000 Genomes phase 3 release
**HG02537**	female	SAME1840165	ACB	African Caribbean	AFR	African Ancestry	ACB	1000 Genomes on GRCh38,1000 Genomes 30x on GRCh38,1000 Genomes phase 3 release
**HG02283**	male	SAME1840173	ACB	African Caribbean	AFR	African Ancestry	ACB	1000 Genomes on GRCh38,1000 Genomes 30x on GRCh38,1000 Genomes phase 3 release
**HG02549**	female	SAME1839917	ACB	African Caribbean	AFR	African Ancestry	ACB	1000 Genomes on GRCh38,1000 Genomes 30x on GRCh38,1000 Genomes phase 3 release
**HG02284**	male	SAME1840168	ACB	African Caribbean	AFR	African Ancestry	ACB	1000 Genomes on GRCh38,1000 Genomes 30x on GRCh38,1000 Genomes phase 3 release
**HG02303**	female	SAME124278	PEL	Peruvian	AMR	American Ancestry	PEL	1000 Genomes 30x on GRCh38,1000 Genomes phase 3 release
**HG02291**	male	SAME1839948	PEL	Peruvian	AMR	American Ancestry	PEL	1000 Genomes on GRCh38,1000 Genomes 30x on GRCh38,1000 Genomes phase 3 release
**HG02308**	female	SAME1839529	ACB	African Caribbean	AFR	African Ancestry	ACB	1000 Genomes on GRCh38,1000 Genomes 30x on GRCh38,1000 Genomes phase 3 release
**HG02304**	male	SAME1839548	PEL	Peruvian	AMR	American Ancestry	PEL	1000 Genomes on GRCh38,1000 Genomes 30x on GRCh38,1000 Genomes phase 3 release
**HG02353**	male	SAME1840276	CDX	Dai Chinese	EAS	East Asian Ancestry	CDX	1000 Genomes on GRCh38,1000 Genomes 30x on GRCh38,1000 Genomes phase 3 release
**HG02309**	female	SAME1839525	ACB	African Caribbean	AFR	African Ancestry	ACB	1000 Genomes on GRCh38,1000 Genomes 30x on GRCh38,1000 Genomes phase 3 release
**HG02316**	male	SAME1839661	ACB	African Caribbean	AFR	African Ancestry	ACB	1000 Genomes 30x on GRCh38,1000 Genomes phase 3 release
**HG02360**	male	SAME1840391	CDX	Dai Chinese	EAS	East Asian Ancestry	CDX	1000 Genomes on GRCh38,1000 Genomes 30x on GRCh38,1000 Genomes phase 3 release
**HG02323**	male	SAME1839440	ACB	African Caribbean	AFR	African Ancestry	ACB	1000 Genomes on GRCh38,1000 Genomes 30x on GRCh38,1000 Genomes phase 3 release
**HG02330**	male	SAME1839237	ACB	African Caribbean	AFR	African Ancestry	ACB	1000 Genomes on GRCh38,1000 Genomes 30x on GRCh38,1000 Genomes phase 3 release
**HG02384**	male	SAME1840011	CDX	Dai Chinese	EAS	East Asian Ancestry	CDX	1000 Genomes on GRCh38,1000 Genomes 30x on GRCh38,1000 Genomes phase 3 release
**HG02373**	male	SAME124104	CDX	Dai Chinese	EAS	East Asian Ancestry	CDX	1000 Genomes on GRCh38,1000 Genomes 30x on GRCh38,1000 Genomes phase 3 release
**HG02144**	female	SAME1839624	ACB	African Caribbean	AFR	African Ancestry	ACB	1000 Genomes on GRCh38,1000 Genomes 30x on GRCh38,1000 Genomes phase 3 release
**HG02380**	male	SAME1840045	CDX	Dai Chinese	EAS	East Asian Ancestry	CDX	1000 Genomes on GRCh38,1000 Genomes 30x on GRCh38,1000 Genomes phase 3 release
**HG02151**	female	SAME1839403	CDX	Dai Chinese	EAS	East Asian Ancestry	CDX	1000 Genomes on GRCh38,1000 Genomes 30x on GRCh38,1000 Genomes phase 3 release
**HG02385**	male	SAME1840006	CDX	Dai Chinese	EAS	East Asian Ancestry	CDX	1000 Genomes on GRCh38,1000 Genomes 30x on GRCh38,1000 Genomes phase 3 release
**HG02156**	female	SAME124793	CDX	Dai Chinese	EAS	East Asian Ancestry	CDX	1000 Genomes on GRCh38,1000 Genomes 30x on GRCh38,1000 Genomes phase 3 release
**HG02392**	male	SAME1839776	CDX	Dai Chinese	EAS	East Asian Ancestry	CDX	1000 Genomes on GRCh38,1000 Genomes 30x on GRCh38,1000 Genomes phase 3 release
**HG02397**	male	SAME123308	CDX	Dai Chinese	EAS	East Asian Ancestry	CDX	1000 Genomes on GRCh38,1000 Genomes 30x on GRCh38,1000 Genomes phase 3 release
**HG02233**	male	SAME1839370	IBS	Iberian	EUR	European Ancestry	IBS	1000 Genomes on GRCh38,1000 Genomes 30x on GRCh38,1000 Genomes phase 3 release
**HG02238**	male	SAME1839423	IBS	Iberian	EUR	European Ancestry	IBS	1000 Genomes on GRCh38,1000 Genomes 30x on GRCh38,1000 Genomes phase 3 release
**HG02789**	male	SAME1840280	PJL	Punjabi	SAS	South Asian Ancestry	PJL	1000 Genomes on GRCh38,1000 Genomes 30x on GRCh38,1000 Genomes phase 3 release
**HG02240**	female	SAME1839481	IBS	Iberian	EUR	European Ancestry	IBS	1000 Genomes 30x on GRCh38,1000 Genomes phase 3 release
**HG02791**	male	SAME1840342	PJL	Punjabi	SAS	South Asian Ancestry	PJL	1000 Genomes 30x on GRCh38,1000 Genomes phase 3 release
**HG02252**	female	SAME1839354	PEL	Peruvian	AMR	American Ancestry	PEL	1000 Genomes on GRCh38,1000 Genomes 30x on GRCh38,1000 Genomes phase 3 release
**HG02804**	male	SAME1840084	GWD	Gambian Mandinka	AFR	African Ancestry	GWD	1000 Genomes on GRCh38,1000 Genomes 30x on GRCh38,1000 Genomes phase 3 release,Gambian Genome Variation Project (GRCh38)
**HG02257**	female	SAME1839326	ACB	African Caribbean	AFR	African Ancestry	ACB	1000 Genomes 30x on GRCh38,1000 Genomes phase 3 release
**HG02809**	female	SAME1840067	GWD	Gambian Mandinka	AFR	African Ancestry	GWD	1000 Genomes 30x on GRCh38
**HG02271**	male	SAME1840319	PEL	Peruvian	AMR	American Ancestry	PEL	1000 Genomes on GRCh38,1000 Genomes 30x on GRCh38,1000 Genomes phase 3 release
**HG02811**	female	SAME1839849	GWD	Gambian Mandinka	AFR	African Ancestry	GWD	1000 Genomes on GRCh38,1000 Genomes 30x on GRCh38,1000 Genomes phase 3 release,Gambian Genome Variation Project (GRCh38)
**HG02816**	male	SAME1839822	GWD	Gambian Mandinka	AFR	African Ancestry	GWD	1000 Genomes on GRCh38,1000 Genomes 30x on GRCh38,1000 Genomes phase 3 release,Gambian Genome Variation Project (GRCh38)
**HG02315**	female	SAME1839666	ACB	African Caribbean	AFR	African Ancestry	ACB	1000 Genomes on GRCh38,1000 Genomes 30x on GRCh38,1000 Genomes phase 3 release
**HG02854**	male	SAME1839336	GWD	Gambian Mandinka	AFR	African Ancestry	GWD	1000 Genomes on GRCh38,1000 Genomes 30x on GRCh38,1000 Genomes phase 3 release,Gambian Genome Variation Project (GRCh38)
**HG02322**	female	SAME1839444	ACB	African Caribbean	AFR	African Ancestry	ACB	1000 Genomes on GRCh38,1000 Genomes 30x on GRCh38,1000 Genomes phase 3 release
**HG02861**	female	SAME1839125	GWD	Gambian Mandinka	AFR	African Ancestry	GWD	1000 Genomes on GRCh38,1000 Genomes 30x on GRCh38,1000 Genomes phase 3 release,Gambian Genome Variation Project (GRCh38)
**HG02334**	male	SAME1839307	ACB	African Caribbean	AFR	African Ancestry	ACB	1000 Genomes on GRCh38,1000 Genomes 30x on GRCh38,1000 Genomes phase 3 release
**HG02878**	male	SAME1840363	GWD	Gambian Mandinka	AFR	African Ancestry	GWD	1000 Genomes on GRCh38,1000 Genomes 30x on GRCh38,1000 Genomes phase 3 release,Gambian Genome Variation Project (GRCh38)
**HG02339**	female	SAME1839288	ACB	African Caribbean	AFR	African Ancestry	ACB	1000 Genomes on GRCh38,1000 Genomes 30x on GRCh38,1000 Genomes phase 3 release
**HG02880**	female	SAME1840090	GWD	Gambian Mandinka	AFR	African Ancestry	GWD	1000 Genomes 30x on GRCh38
**HG02389**	male	SAME1840085	CDX	Dai Chinese	EAS	East Asian Ancestry	CDX	1000 Genomes on GRCh38,1000 Genomes 30x on GRCh38,1000 Genomes phase 3 release
**HG02885**	female	SAME1840157	GWD	Gambian Mandinka	AFR	African Ancestry	GWD	1000 Genomes on GRCh38,1000 Genomes 30x on GRCh38,1000 Genomes phase 3 release,Gambian Genome Variation Project (GRCh38)
**HG02391**	male	SAME1839784	CDX	Dai Chinese	EAS	East Asian Ancestry	CDX	1000 Genomes on GRCh38,1000 Genomes 30x on GRCh38,1000 Genomes phase 3 release
**HG02892**	male	SAME1839935	GWD	Gambian Mandinka	AFR	African Ancestry	GWD	1000 Genomes 30x on GRCh38
**HG02396**	male	SAME125357	CDX	Dai Chinese	EAS	East Asian Ancestry	CDX	1000 Genomes on GRCh38,1000 Genomes 30x on GRCh38,1000 Genomes phase 3 release
**HG02897**	female	SAME1839910	GWD	Gambian Mandinka	AFR	African Ancestry	GWD	1000 Genomes 30x on GRCh38
**HG02409**	male	SAME1839457	CDX	Dai Chinese	EAS	East Asian Ancestry	CDX	1000 Genomes on GRCh38,1000 Genomes 30x on GRCh38,1000 Genomes phase 3 release
**HG02924**	male	SAME1839516	ESN	Esan	AFR	African Ancestry	ESN	1000 Genomes 30x on GRCh38
**HG02733**	male	SAME1839874	PJL	Punjabi	SAS	South Asian Ancestry	PJL	1000 Genomes on GRCh38,1000 Genomes 30x on GRCh38,1000 Genomes phase 3 release
**HG02974**	female	SAME1840241	ESN	Esan	AFR	African Ancestry	ESN	1000 Genomes on GRCh38,1000 Genomes 30x on GRCh38,1000 Genomes phase 3 release
**HG02738**	male	SAME1839560	PJL	Punjabi	SAS	South Asian Ancestry	PJL	1000 Genomes 30x on GRCh38
**HG02979**	female	SAME1840209	ESN	Esan	AFR	African Ancestry	ESN	1000 Genomes on GRCh38,1000 Genomes 30x on GRCh38,1000 Genomes phase 3 release
**HG02757**	female	SAME1839451	GWD	Gambian Mandinka	AFR	African Ancestry	GWD	1000 Genomes on GRCh38,1000 Genomes 30x on GRCh38,1000 Genomes phase 3 release,Gambian Genome Variation Project (GRCh38)
**HG02981**	male	SAME1840024	ESN	Esan	AFR	African Ancestry	ESN	1000 Genomes on GRCh38,1000 Genomes 30x on GRCh38,1000 Genomes phase 3 release
**HG02764**	male	SAME1839250	GWD	Gambian Mandinka	AFR	African Ancestry	GWD	1000 Genomes 30x on GRCh38
**HG03009**	male	SAME1840256	BEB	Bengali	SAS	South Asian Ancestry	BEB	1000 Genomes on GRCh38,1000 Genomes 30x on GRCh38,Human Genome Structural Variation Consortium, Phase 2,1000 Genomes phase 3 release
**HG02769**	female	SAME1839231	GWD	Gambian Mandinka	AFR	African Ancestry	GWD	1000 Genomes on GRCh38,1000 Genomes 30x on GRCh38,1000 Genomes phase 3 release,Gambian Genome Variation Project (GRCh38)
**HG03016**	female	SAME1839952	PJL	Punjabi	SAS	South Asian Ancestry	PJL	1000 Genomes on GRCh38,1000 Genomes 30x on GRCh38,1000 Genomes phase 3 release
**HG02771**	male	SAME1839045	GWD	Gambian Mandinka	AFR	African Ancestry	GWD	1000 Genomes on GRCh38,1000 Genomes 30x on GRCh38,1000 Genomes phase 3 release,Gambian Genome Variation Project (GRCh38)
**HG02776**	female	SAME1839017	PJL	Punjabi	SAS	South Asian Ancestry	PJL	1000 Genomes 30x on GRCh38
**HG02734**	female	SAME1839869	PJL	Punjabi	SAS	South Asian Ancestry	PJL	1000 Genomes on GRCh38,1000 Genomes 30x on GRCh38,1000 Genomes phase 3 release
**HG02783**	male	SAME1839162	PJL	Punjabi,Punjabi	SAS	South Asia (SGDP),South Asian Ancestry	PunjabiSGDP,PJL	1000 Genomes on GRCh38,Simons Genome Diversity Project,1000 Genomes 30x on GRCh38,1000 Genomes phase 3 release
**HG02562**	female	SAME1839832	GWD	Gambian Mandinka	AFR	African Ancestry	GWD	1000 Genomes on GRCh38,1000 Genomes 30x on GRCh38,1000 Genomes phase 3 release,Gambian Genome Variation Project (GRCh38)
**HG02760**	female	SAME1839267	GWD	Gambian Mandinka	AFR	African Ancestry	GWD	1000 Genomes on GRCh38,1000 Genomes 30x on GRCh38,1000 Genomes phase 3 release,Gambian Genome Variation Project (GRCh38)
**HG02772**	female	SAME1839039	GWD	Gambian Mandinka	AFR	African Ancestry	GWD	1000 Genomes on GRCh38,1000 Genomes 30x on GRCh38,1000 Genomes phase 3 release,Gambian Genome Variation Project (GRCh38)
**HG02574**	female	SAME1839652	GWD	Gambian Mandinka,Gar	AFR	African Ancestry,Africa (SGDP)	GWD,GambianSGDP	1000 Genomes on GRCh38,Simons Genome Diversity Project,1000 Genomes 30x on GRCh38,1000 Genomes phase 3 release,Gambian Genome Variation Project (GRCh
**HG02784**	female	SAME1839157	PJL	Punjabi	SAS	South Asian Ancestry	PJL	1000 Genomes on GRCh38,1000 Genomes 30x on GRCh38,1000 Genomes phase 3 release
**HG02586**	female	SAME1839414	GWD	Gambian Mandinka	AFR	African Ancestry	GWD	1000 Genomes on GRCh38,1000 Genomes 30x on GRCh38,1000 Genomes phase 3 release,Gambian Genome Variation Project (GRCh38)
**HG02690**	male	SAME1839088	PJL	Punjabi	SAS	South Asian Ancestry	PJL	1000 Genomes on GRCh38,1000 Genomes 30x on GRCh38,1000 Genomes phase 3 release
**HG02601**	female	SAME1840293	PJL	Punjabi	SAS	South Asian Ancestry	PJL	1000 Genomes on GRCh38,1000 Genomes 30x on GRCh38,1000 Genomes phase 3 release
**HG02703**	female	SAME1840142	GWD	Gambian Mandinka	AFR	African Ancestry	GWD	1000 Genomes on GRCh38,1000 Genomes 30x on GRCh38,1000 Genomes phase 3 release,Gambian Genome Variation Project (GRCh38)
**HG02788**	female	SAME1840285	PJL	Punjabi	SAS	South Asian Ancestry	PJL	1000 Genomes 30x on GRCh38
**HG02715**	male	SAME1839987	GWD	Gambian Mandinka	AFR	African Ancestry	GWD	1000 Genomes on GRCh38,1000 Genomes 30x on GRCh38,1000 Genomes phase 3 release,Gambian Genome Variation Project (GRCh38)
**HG02790**	female	SAME1840345	PJL	Punjabi,Punjabi	SAS	South Asia (SGDP),South Asian Ancestry	PunjabiSGDP,PJL	1000 Genomes on GRCh38,Simons Genome Diversity Project,1000 Genomes 30x on GRCh38,1000 Genomes phase 3 release
**HG02722**	female	SAME1839747	GWD	Gambian Mandinka	AFR	African Ancestry	GWD	1000 Genomes on GRCh38,1000 Genomes 30x on GRCh38,1000 Genomes phase 3 release,Gambian Genome Variation Project (GRCh38)
**HG02808**	female	SAME1840069	GWD	Gambian Mandinka	AFR	African Ancestry	GWD	1000 Genomes on GRCh38,1000 Genomes 30x on GRCh38,1000 Genomes phase 3 release,Gambian Genome Variation Project (GRCh38)
**HG02727**	male	SAME1839727	PJL	Punjabi	SAS	South Asian Ancestry	PJL	1000 Genomes on GRCh38,1000 Genomes 30x on GRCh38,1000 Genomes phase 3 release
**HG02810**	male	SAME1839854	GWD	Gambian Mandinka	AFR	African Ancestry	GWD	1000 Genomes on GRCh38,1000 Genomes 30x on GRCh38,1000 Genomes phase 3 release,Gambian Genome Variation Project (GRCh38)
**HG03028**	female	SAME1839729	GWD	Gambian Mandinka	AFR	African Ancestry	GWD	1000 Genomes on GRCh38,1000 Genomes 30x on GRCh38,1000 Genomes phase 3 release,Gambian Genome Variation Project (GRCh38)
**HG02815**	male	SAME1839829	GWD	Gambian Mandinka	AFR	African Ancestry	GWD	1000 Genomes 30x on GRCh38
**HG03035**	male	SAME1839573	GWD	Gambian Mandinka	AFR	African Ancestry	GWD	1000 Genomes 30x on GRCh38
**HG02839**	male	SAME1839413	GWD	Gambian Mandinka	AFR	African Ancestry	GWD	1000 Genomes on GRCh38,1000 Genomes 30x on GRCh38,1000 Genomes phase 3 release,Gambian Genome Variation Project (GRCh38)
**HG03047**	female	SAME1839644	GWD	Gambian Mandinka	AFR	African Ancestry	GWD	1000 Genomes 30x on GRCh38
**HG02841**	female	SAME1839470	GWD	Gambian Mandinka	AFR	African Ancestry	GWD	1000 Genomes 30x on GRCh38
**HG03054**	male	SAME1839507	MSL	Mende	AFR	African Ancestry	MSL	1000 Genomes on GRCh38,1000 Genomes 30x on GRCh38,1000 Genomes phase 3 release
**HG02853**	male	SAME1839342	GWD	Gambian Mandinka	AFR	African Ancestry	GWD	1000 Genomes 30x on GRCh38
**HG02860**	male	SAME1839132	GWD	Gambian Mandinka	AFR	African Ancestry	GWD	1000 Genomes on GRCh38,1000 Genomes 30x on GRCh38,1000 Genomes phase 3 release,Gambian Genome Variation Project (GRCh38)
**HG03061**	female	SAME1839215	MSL	Mende	AFR	African Ancestry	MSL	1000 Genomes on GRCh38,1000 Genomes 30x on GRCh38,1000 Genomes phase 3 release
**HG02884**	male	SAME1840161	GWD	Gambian Mandinka	AFR	African Ancestry	GWD	1000 Genomes on GRCh38,1000 Genomes 30x on GRCh38,1000 Genomes phase 3 release,Gambian Genome Variation Project (GRCh38)
**HG02657**	male	SAME1839600	PJL	Punjabi	SAS	South Asian Ancestry	PJL	1000 Genomes on GRCh38,1000 Genomes 30x on GRCh38,1000 Genomes phase 3 release
**HG02889**	female	SAME1840133	GWD	Gambian Mandinka	AFR	African Ancestry	GWD	1000 Genomes 30x on GRCh38
**HG02676**	female	SAME1839491	GWD	Gambian Mandinka	AFR	African Ancestry	GWD	1000 Genomes on GRCh38,1000 Genomes 30x on GRCh38,1000 Genomes phase 3 release,Gambian Genome Variation Project (GRCh38)
**HG02891**	female	SAME1839938	GWD	Gambian Mandinka	AFR	African Ancestry	GWD	1000 Genomes on GRCh38,1000 Genomes 30x on GRCh38,1000 Genomes phase 3 release,Gambian Genome Variation Project (GRCh38)
**HG02683**	female	SAME1839298	PJL	Punjabi	SAS	South Asian Ancestry	PJL	1000 Genomes 30x on GRCh38
**HG02896**	female	SAME1839916	GWD	Gambian Mandinka	AFR	African Ancestry	GWD	1000 Genomes on GRCh38,1000 Genomes 30x on GRCh38,1000 Genomes phase 3 release,Gambian Genome Variation Project (GRCh38)
**HG02688**	female	SAME1839358	PJL	Punjabi	SAS	South Asian Ancestry	PJL	1000 Genomes on GRCh38,1000 Genomes 30x on GRCh38,1000 Genomes phase 3 release
**HG02923**	male	SAME1839436	ESN	Esan	AFR	African Ancestry	ESN	1000 Genomes on GRCh38,1000 Genomes 30x on GRCh38,1000 Genomes phase 3 release
**HG02614**	female	SAME1840385	GWD	Gambian Mandinka	AFR	African Ancestry	GWD	1000 Genomes on GRCh38,1000 Genomes 30x on GRCh38,1000 Genomes phase 3 release,Gambian Genome Variation Project (GRCh38)
**HG02621**	female	SAME1840187	GWD	Gambian Mandinka	AFR	African Ancestry	GWD	1000 Genomes on GRCh38,1000 Genomes 30x on GRCh38,1000 Genomes phase 3 release,Gambian Genome Variation Project (GRCh38)
**HG02645**	male	SAME1839774	GWD	Gambian Mandinka	AFR	African Ancestry	GWD	1000 Genomes on GRCh38,1000 Genomes 30x on GRCh38,1000 Genomes phase 3 release,Gambian Genome Variation Project (GRCh38)
**HG02485**	female	SAME1839564	ACB	African Caribbean	AFR	African Ancestry	ACB	1000 Genomes on GRCh38,1000 Genomes 30x on GRCh38,1000 Genomes phase 3 release

**HG02652**	female	SAME1839541	PJL	Punjabi	SAS	South Asian Ancestry	PJL	1000 Genomes on GRCh38,1000 Genomes 30x on GRCh38,1000 Genomes phase 3 release
**HG02492**	male	SAME1839610	PJL	Punjabi	SAS	South Asian Ancestry	PJL	1000 Genomes 30x on GRCh38,Human Genome Structural Variation Consortium, Phase 2,1000 Genomes phase 3 release
**HG02943**	female	SAME1839085	ESN	Esan,Esan	AFR	African Ancestry,Africa (SGDP)	ESN,EsanSGDP	1000 Genomes on GRCh38,Simons Genome Diversity Project,1000 Genomes 30x on GRCh38,1000 Genomes phase 3 release
**HG02497**	female	SAME1839682	ACB	African Caribbean	AFR	African Ancestry	ACB	1000 Genomes on GRCh38,1000 Genomes 30x on GRCh38,1000 Genomes phase 3 release
**HG02948**	male	SAME1839153	ESN	Esan	AFR	African Ancestry	ESN	1000 Genomes 30x on GRCh38
**HG02505**	female	SAME1839351	ACB	African Caribbean	AFR	African Ancestry	ACB	1000 Genomes on GRCh38,1000 Genomes 30x on GRCh38,1000 Genomes phase 3 release
**HG03066**	male	SAME1839324	MSL	Mende	AFR	African Ancestry	MSL	1000 Genomes on GRCh38,1000 Genomes 30x on GRCh38,1000 Genomes phase 3 release
**HG02656**	male	SAME1839607	PJL	Punjabi	SAS	South Asian Ancestry	PJL	1000 Genomes 30x on GRCh38,1000 Genomes phase 3 release
**HG03073**	female	SAME1839019	MSL	Mende	AFR	African Ancestry	MSL	1000 Genomes on GRCh38,1000 Genomes 30x on GRCh38,1000 Genomes phase 3 release
**HG02668**	male	SAME1839377	GWD	Gambian Mandinka	AFR	African Ancestry	GWD	1000 Genomes 30x on GRCh38
**HG03078**	male	SAME1839047	MSL	Mende,Mende	AFR	African Ancestry,Africa (SGDP)	MSL,MendeSGDP	1000 Genomes on GRCh38,Simons Genome Diversity Project,1000 Genomes 30x on GRCh38,1000 Genomes phase 3 release
**HG02675**	male	SAME1839495	GWD	Gambian Mandinka	AFR	African Ancestry	GWD	1000 Genomes on GRCh38,1000 Genomes 30x on GRCh38,1000 Genomes phase 3 release,Gambian Genome Variation Project (GRCh38)
**HG02682**	female	SAME1839300	PJL	Punjabi	SAS	South Asian Ancestry	PJL	1000 Genomes on GRCh38,1000 Genomes 30x on GRCh38,1000 Genomes phase 3 release
**HG03085**	female	SAME1840328	MSL	Mende,Mende	AFR	African Ancestry,Africa (SGDP)	MSL,MendeSGDP	1000 Genomes on GRCh38,Simons Genome Diversity Project,1000 Genomes 30x on GRCh38,1000 Genomes phase 3 release
**HG02687**	male	SAME1839270	PJL	Punjabi	SAS	South Asian Ancestry	PJL	1000 Genomes on GRCh38,1000 Genomes 30x on GRCh38,1000 Genomes phase 3 release
**HG03097**	female	SAME1840130	MSL	Mende	AFR	African Ancestry	MSL	1000 Genomes on GRCh38,1000 Genomes 30x on GRCh38,1000 Genomes phase 3 release
**HG02613**	male	SAME1840390	GWD	Gambian Mandinka	AFR	African Ancestry	GWD	1000 Genomes on GRCh38,1000 Genomes 30x on GRCh38,1000 Genomes phase 3 release,Gambian Genome Variation Project (GRCh38)
**HG03249**	male	SAME1839183	GWD	Gambian Mandinka	AFR	African Ancestry	GWD	1000 Genomes on GRCh38,1000 Genomes 30x on GRCh38,Gambian Genome Variation Project (GRCh38)
**HG02620**	male	SAME1840191	GWD	Gambian Mandinka	AFR	African Ancestry	GWD	1000 Genomes on GRCh38,1000 Genomes 30x on GRCh38,1000 Genomes phase 3 release,Gambian Genome Variation Project (GRCh38)
**HG03251**	female	SAME1840266	GWD	Gambian Mandinka	AFR	African Ancestry	GWD	1000 Genomes 30x on GRCh38
**HG02625**	male	SAME1840257	GWD	Gambian Mandinka	AFR	African Ancestry	GWD	1000 Genomes 30x on GRCh38
**HG03268**	male	SAME1840093	ESN	Esan	AFR	African Ancestry	ESN	1000 Genomes on GRCh38,1000 Genomes 30x on GRCh38,1000 Genomes phase 3 release
**HG02644**	male	SAME1839782	GWD	Gambian Mandinka	AFR	African Ancestry	GWD	1000 Genomes 30x on GRCh38
**HG03270**	female	SAME1840194	ESN	Esan	AFR	African Ancestry	ESN	1000 Genomes on GRCh38,1000 Genomes 30x on GRCh38,1000 Genomes phase 3 release
**HG02649**	female	SAME1839852	PJL	Punjabi	SAS	South Asian Ancestry	PJL	1000 Genomes on GRCh38,1000 Genomes 30x on GRCh38,1000 Genomes phase 3 release
**HG03294**	female	SAME1839877	ESN	Esan	AFR	African Ancestry	ESN	1000 Genomes on GRCh38,1000 Genomes 30x on GRCh38,1000 Genomes phase 3 release
**HG02651**	male	SAME1839544	PJL	Punjabi	SAS	South Asian Ancestry	PJL	1000 Genomes on GRCh38,1000 Genomes 30x on GRCh38,1000 Genomes phase 3 release
**HG03100**	male	SAME1840003	ESN	Esan,Esan	AFR	African Ancestry,Africa (SGDP)	ESN,EsanSGDP	1000 Genomes on GRCh38,Simons Genome Diversity Project,1000 Genomes 30x on GRCh38,1000 Genomes phase 3 release
**HG02694**	female	SAME1839065	PJL	Punjabi	SAS	South Asian Ancestry	PJL	1000 Genomes on GRCh38,1000 Genomes 30x on GRCh38,1000 Genomes phase 3 release
**HG03105**	female	SAME1840041	ESN	Esan	AFR	African Ancestry	ESN	1000 Genomes on GRCh38,1000 Genomes 30x on GRCh38,1000 Genomes phase 3 release
**HG02699**	male	SAME1839129	PJL	Punjabi	SAS	South Asian Ancestry	PJL	1000 Genomes on GRCh38,1000 Genomes 30x on GRCh38,1000 Genomes phase 3 release
**HG03112**	male	SAME1839893	ESN	Esan	AFR	African Ancestry	ESN	1000 Genomes on GRCh38,1000 Genomes 30x on GRCh38,1000 Genomes phase 3 release
**HG02702**	male	SAME1840149	GWD	Gambian Mandinka	AFR	African Ancestry	GWD	1000 Genomes on GRCh38,1000 Genomes 30x on GRCh38,1000 Genomes phase 3 release,Gambian Genome Variation Project (GRCh38)
**HG03117**	female	SAME1839821	ESN	Esan	AFR	African Ancestry	ESN	1000 Genomes on GRCh38,1000 Genomes 30x on GRCh38,1000 Genomes phase 3 release
**HG02721**	male	SAME1839749	GWD	Gambian Mandinka	AFR	African Ancestry	GWD	1000 Genomes on GRCh38,1000 Genomes 30x on GRCh38,1000 Genomes phase 3 release,Gambian Genome Variation Project (GRCh38)
**HG03352**	male	SAME1840243	ESN	Esan	AFR	African Ancestry	ESN	1000 Genomes on GRCh38,1000 Genomes 30x on GRCh38,1000 Genomes phase 3 release
**HG02726**	female	SAME1839732	PJL	Punjabi	SAS	South Asian Ancestry	PJL	1000 Genomes 30x on GRCh38,1000 Genomes phase 3 release
**HG03369**	female	SAME1840078	ESN	Esan	AFR	African Ancestry	ESN	1000 Genomes on GRCh38,1000 Genomes 30x on GRCh38,1000 Genomes phase 3 release
**HG02512**	male	SAME1839146	KHV	Kinh Vietnamese	EAS	East Asian Ancestry	KHV	1000 Genomes on GRCh38,1000 Genomes 30x on GRCh38,1000 Genomes phase 3 release
**HG03371**	male	SAME1839712	ESN	Esan	AFR	African Ancestry	ESN	1000 Genomes 30x on GRCh38,Human Genome Structural Variation Consortium, Phase 2
**HG02524**	male	SAME1840317	KHV	Kinh Vietnamese	EAS	East Asian Ancestry	KHV	1000 Genomes on GRCh38,1000 Genomes 30x on GRCh38
**HG03376**	male	SAME1839736	MSL	Mende	AFR	African Ancestry	MSL	1000 Genomes on GRCh38,1000 Genomes 30x on GRCh38,1000 Genomes phase 3 release
**HG02536**	male	SAME1840172	ACB	African Caribbean	AFR	African Ancestry	ACB	1000 Genomes on GRCh38,1000 Genomes 30x on GRCh38,1000 Genomes phase 3 release
**HG02555**	female	SAME1840070	ACB	African Caribbean	AFR	African Ancestry	ACB	1000 Genomes on GRCh38,1000 Genomes 30x on GRCh38,1000 Genomes phase 3 release
**HG03388**	male	SAME1839524	MSL	Mende	AFR	African Ancestry	MSL	1000 Genomes on GRCh38,1000 Genomes 30x on GRCh38,1000 Genomes phase 3 release
**HG03027**	male	SAME1839725	GWD	Gambian Mandinka	AFR	African Ancestry	GWD	1000 Genomes on GRCh38,1000 Genomes 30x on GRCh38,1000 Genomes phase 3 release
**HG03034**	female	SAME1839568	GWD	Gambian Mandinka	AFR	African Ancestry	GWD	1000 Genomes on GRCh38,1000 Genomes 30x on GRCh38,Gambian Genome Variation Project (GRCh38)
**HG03039**	male	SAME1839593	GWD	Gambian Mandinka	AFR	African Ancestry	GWD	1000 Genomes on GRCh38,1000 Genomes 30x on GRCh38,1000 Genomes phase 3 release,Gambian Genome Variation Project (GRCh38)
**HG03410**	female	SAME1839182	MSL	Mende	AFR	African Ancestry	MSL	1000 Genomes on GRCh38,1000 Genomes 30x on GRCh38,1000 Genomes phase 3 release
**HG03041**	female	SAME1839620	GWD	Gambian Mandinka	AFR	African Ancestry	GWD	1000 Genomes 30x on GRCh38
**HG03198**	female	SAME1839933	ESN	Esan	AFR	African Ancestry	ESN	1000 Genomes on GRCh38,1000 Genomes 30x on GRCh38,1000 Genomes phase 3 release
**HG03046**	female	SAME1839639	GWD	Gambian Mandinka	AFR	African Ancestry	GWD	1000 Genomes on GRCh38,1000 Genomes 30x on GRCh38,1000 Genomes phase 3 release,Gambian Genome Variation Project (GRCh38)
**HG03225**	male	SAME1839460	MSL	Mende	AFR	African Ancestry	MSL	1000 Genomes on GRCh38,1000 Genomes 30x on GRCh38,1000 Genomes phase 3 release
**HG03237**	male	SAME1839282	PJL	Punjabi	SAS	South Asian Ancestry	PJL	1000 Genomes on GRCh38,1000 Genomes 30x on GRCh38,1000 Genomes phase 3 release
**HG03058**	female	SAME1839442	MSL	Mende	AFR	African Ancestry	MSL	1000 Genomes on GRCh38,1000 Genomes 30x on GRCh38,1000 Genomes phase 3 release
**HG03124**	male	SAME1839669	ESN	Esan	AFR	African Ancestry	ESN	1000 Genomes on GRCh38,1000 Genomes 30x on GRCh38,1000 Genomes phase 3 release
**HG03060**	male	SAME1839211	MSL	Mende	AFR	African Ancestry	MSL	1000 Genomes on GRCh38,1000 Genomes 30x on GRCh38,1000 Genomes phase 3 release
**HG03129**	female	SAME1839699	ESN	Esan	AFR	African Ancestry	ESN	1000 Genomes on GRCh38,1000 Genomes 30x on GRCh38,1000 Genomes phase 3 release
**HG03065**	male	SAME1839318	MSL	Mende	AFR	African Ancestry	MSL	1000 Genomes 30x on GRCh38,Human Genome Structural Variation Consortium, Phase 2
**HG03131**	male	SAME1839368	ESN	Esan	AFR	African Ancestry	ESN	1000 Genomes 30x on GRCh38
**HG03072**	male	SAME1839016	MSL	Mende	AFR	African Ancestry	MSL	1000 Genomes on GRCh38,1000 Genomes 30x on GRCh38,1000 Genomes phase 3 release
**HG03136**	male	SAME1839387	ESN	Esan	AFR	African Ancestry	ESN	1000 Genomes on GRCh38,1000 Genomes 30x on GRCh38,1000 Genomes phase 3 release
**HG03077**	male	SAME1839044	MSL	Mende	AFR	African Ancestry	MSL	1000 Genomes on GRCh38,1000 Genomes 30x on GRCh38,1000 Genomes phase 3 release
**HG03299**	male	SAME1839805	ESN	Esan	AFR	African Ancestry	ESN	1000 Genomes 30x on GRCh38
**HG03084**	male	SAME1840324	MSL	Mende	AFR	African Ancestry	MSL	1000 Genomes on GRCh38,1000 Genomes 30x on GRCh38,1000 Genomes phase 3 release
**HG03302**	male	SAME1839479	ESN	Esan	AFR	African Ancestry	ESN	1000 Genomes 30x on GRCh38
**HG03091**	female	SAME1840360	MSL	Mende	AFR	African Ancestry	MSL	1000 Genomes on GRCh38,1000 Genomes 30x on GRCh38,1000 Genomes phase 3 release
**HG03307**	male	SAME1839509	ESN	Esan	AFR	African Ancestry	ESN	1000 Genomes on GRCh38,1000 Genomes 30x on GRCh38
**HG03096**	male	SAME1840126	MSL	Mende	AFR	African Ancestry	MSL	1000 Genomes on GRCh38,1000 Genomes 30x on GRCh38,1000 Genomes phase 3 release
**HG03314**	male	SAME1839217	ESN	Esan	AFR	African Ancestry	ESN	1000 Genomes 30x on GRCh38
**HG03248**	male	SAME1839180	GWD	Gambian Mandinka	AFR	African Ancestry	GWD	1000 Genomes 30x on GRCh38
**HG03162**	female	SAME1839112	ESN	Esan	AFR	African Ancestry	ESN	1000 Genomes on GRCh38,1000 Genomes 30x on GRCh38,1000 Genomes phase 3 release
**HG03250**	female	SAME1840262	GWD	Gambian Mandinka	AFR	African Ancestry	GWD	1000 Genomes on GRCh38,1000 Genomes 30x on GRCh38,Gambian Genome Variation Project (GRCh38)
**HG03267**	female	SAME1840088	ESN	Esan	AFR	African Ancestry	ESN	1000 Genomes on GRCh38,1000 Genomes 30x on GRCh38,1000 Genomes phase 3 release
**HG03193**	male	SAME1839909	ESN	Esan	AFR	African Ancestry	ESN	1000 Genomes on GRCh38,1000 Genomes 30x on GRCh38,1000 Genomes phase 3 release
**HG03279**	female	SAME1839972	ESN	Esan	AFR	African Ancestry	ESN	1000 Genomes on GRCh38,1000 Genomes 30x on GRCh38,1000 Genomes phase 3 release
**HG03605**	female	SAME1840240	BEB	Bengali	SAS	South Asian Ancestry	BEB	1000 Genomes 30x on GRCh38
**HG03617**	female	SAME1840056	BEB	Bengali	SAS	South Asian Ancestry	BEB	1000 Genomes 30x on GRCh38
**HG03197**	male	SAME1839926	ESN	Esan	AFR	African Ancestry	ESN	1000 Genomes 30x on GRCh38
**HG03624**	male	SAME1839709	PJL	Punjabi	SAS	South Asian Ancestry	PJL	1000 Genomes on GRCh38,1000 Genomes 30x on GRCh38,1000 Genomes phase 3 release
**HG03200**	female	SAME1839589	ESN	Esan	AFR	African Ancestry	ESN	1000 Genomes 30x on GRCh38
**HG03629**	male	SAME1839738	PJL	Punjabi	SAS	South Asian Ancestry	PJL	1000 Genomes on GRCh38,1000 Genomes 30x on GRCh38,1000 Genomes phase 3 release
**HG03212**	female	SAME1839399	MSL	Mende	AFR	African Ancestry	MSL	1000 Genomes on GRCh38,1000 Genomes 30x on GRCh38,1000 Genomes phase 3 release
**HG03631**	female	SAME1839855	PJL	Punjabi	SAS	South Asian Ancestry	PJL	1000 Genomes on GRCh38,1000 Genomes 30x on GRCh38,1000 Genomes phase 3 release
**HG03224**	male	SAME1839455	MSL	Mende	AFR	African Ancestry	MSL	1000 Genomes on GRCh38,1000 Genomes 30x on GRCh38,1000 Genomes phase 3 release
**HG03636**	male	SAME1839586	PJL	Punjabi	SAS	South Asian Ancestry	PJL	1000 Genomes on GRCh38,1000 Genomes 30x on GRCh38,1000 Genomes phase 3 release
**HG03229**	female	SAME1839234	PJL	Punjabi	SAS	South Asian Ancestry	PJL	1000 Genomes on GRCh38,1000 Genomes 30x on GRCh38,1000 Genomes phase 3 release
**HG03698**	female	SAME1840145	STU	Tamil	SAS	South Asian Ancestry	STU	1000 Genomes on GRCh38,1000 Genomes 30x on GRCh38,1000 Genomes phase 3 release
**HG03236**	female	SAME1839276	PJL	Punjabi	SAS	South Asian Ancestry	PJL	1000 Genomes 30x on GRCh38
**HG03701**	male	SAME1840016	PJL	Punjabi	SAS	South Asian Ancestry	PJL	1000 Genomes 30x on GRCh38
**HG02973**	male	SAME1840246	ESN	Esan	AFR	African Ancestry	ESN	1000 Genomes on GRCh38,1000 Genomes 30x on GRCh38,1000 Genomes phase 3 release
**HG03706**	female	SAME1839758	PJL	Punjabi	SAS	South Asian Ancestry	PJL	1000 Genomes on GRCh38,1000 Genomes 30x on GRCh38,1000 Genomes phase 3 release
**HG02978**	female	SAME1840216	ESN	Esan	AFR	African Ancestry	ESN	1000 Genomes 30x on GRCh38
**HG03713**	male	SAME1839807	ITU	Telugu	SAS	South Asian Ancestry	ITU	1000 Genomes on GRCh38,1000 Genomes 30x on GRCh38,1000 Genomes phase 3 release
**HG02980**	male	SAME1840034	ESN	Esan	AFR	African Ancestry	ESN	1000 Genomes 30x on GRCh38
**HG03489**	male	SAME1839494	PJL	Punjabi	SAS	South Asian Ancestry	PJL	1000 Genomes 30x on GRCh38
**HG03008**	male	SAME1840253	BEB	Bengali	SAS	South Asian Ancestry	BEB	1000 Genomes 30x on GRCh38
**HG03491**	female	SAME1839264	PJL	Punjabi	SAS	South Asian Ancestry	PJL	1000 Genomes on GRCh38,1000 Genomes 30x on GRCh38,1000 Genomes phase 3 release
**HG03015**	male	SAME1839940	PJL	Punjabi	SAS	South Asian Ancestry	PJL	1000 Genomes on GRCh38,1000 Genomes 30x on GRCh38,1000 Genomes phase 3 release
**HG03511**	female	SAME1840131	ESN	Esan	AFR	African Ancestry	ESN	1000 Genomes on GRCh38,1000 Genomes 30x on GRCh38,1000 Genomes phase 3 release
**HG03022**	female	SAME1839795	PJL	Punjabi	SAS	South Asian Ancestry	PJL	1000 Genomes on GRCh38,1000 Genomes 30x on GRCh38,1000 Genomes phase 3 release
**HG03516**	female	SAME1840160	ESN	Esan	AFR	African Ancestry	ESN	1000 Genomes 30x on GRCh38
**HG03123**	female	SAME1839663	ESN	Esan	AFR	African Ancestry	ESN	1000 Genomes on GRCh38,1000 Genomes 30x on GRCh38,1000 Genomes phase 3 release
**HG03460**	male	SAME1839908	MSL	Mende	AFR	African Ancestry	MSL	1000 Genomes on GRCh38,1000 Genomes 30x on GRCh38,1000 Genomes phase 3 release
**HG03128**	male	SAME1839692	ESN	Esan	AFR	African Ancestry	ESN	1000 Genomes 30x on GRCh38
**HG03130**	male	SAME1839365	ESN	Esan	AFR	African Ancestry	ESN	1000 Genomes on GRCh38,1000 Genomes 30x on GRCh38,1000 Genomes phase 3 release
**HG03472**	male	SAME1839678	MSL	Mende	AFR	African Ancestry	MSL	1000 Genomes on GRCh38,1000 Genomes 30x on GRCh38,1000 Genomes phase 3 release
**HG03135**	female	SAME1839385	ESN	Esan	AFR	African Ancestry	ESN	1000 Genomes on GRCh38,1000 Genomes 30x on GRCh38,1000 Genomes phase 3 release
**HG03159**	female	SAME1839092	ESN	Esan	AFR	African Ancestry	ESN	1000 Genomes on GRCh38,1000 Genomes 30x on GRCh38,1000 Genomes phase 3 release
**HG03484**	male	SAME1839393	MSL	Mende	AFR	African Ancestry	MSL	1000 Genomes on GRCh38,1000 Genomes 30x on GRCh38,1000 Genomes phase 3 release
**HG03439**	male	SAME1840213	MSL	Mende	AFR	African Ancestry	MSL	1000 Genomes on GRCh38,1000 Genomes 30x on GRCh38,1000 Genomes phase 3 release

**HG03109**	male	SAME1839765	ESN	Esan	AFR	African Ancestry	ESN	1000 Genomes on GRCh38,1000 Genomes 30x on GRCh38,1000 Genomes phase 3 release
**HG03446**	female	SAME1839911	MSL	Mende	AFR	African Ancestry	MSL	1000 Genomes on GRCh38,1000 Genomes 30x on GRCh38,1000 Genomes phase 3 release
**HG03111**	female	SAME1839884	ESN	Esan	AFR	African Ancestry	ESN	1000 Genomes on GRCh38,1000 Genomes 30x on GRCh38,1000 Genomes phase 3 release
**HG03453**	female	SAME1839750	MSL	Mende	AFR	African Ancestry	MSL	1000 Genomes 30x on GRCh38
**HG03116**	female	SAME1839816	ESN	Esan	AFR	African Ancestry	ESN	1000 Genomes 30x on GRCh38
**HG03458**	female	SAME1839786	MSL	Mende	AFR	African Ancestry	MSL	1000 Genomes on GRCh38,1000 Genomes 30x on GRCh38,1000 Genomes phase 3 release
**HG03643**	female	SAME1839633	STU	Tamil	SAS	South Asian Ancestry	STU	1000 Genomes on GRCh38,1000 Genomes 30x on GRCh38,1000 Genomes phase 3 release
**HG02947**	male	SAME1839061	ESN	Esan	AFR	African Ancestry	ESN	1000 Genomes on GRCh38,1000 Genomes 30x on GRCh38,1000 Genomes phase 3 release
**HG03650**	female	SAME1839488	PJL	Punjabi	SAS	South Asian Ancestry	PJL	1000 Genomes on GRCh38,1000 Genomes 30x on GRCh38
**HG02954**	male	SAME1840264	ESN	Esan	AFR	African Ancestry	ESN	1000 Genomes 30x on GRCh38
**HG03667**	male	SAME1839331	PJL	Punjabi	SAS	South Asian Ancestry	PJL	1000 Genomes on GRCh38,1000 Genomes 30x on GRCh38,1000 Genomes phase 3 release
**HG02966**	male	SAME1840114	ESN	Esan	AFR	African Ancestry	ESN	1000 Genomes 30x on GRCh38
**HG03578**	female	SAME1839245	MSL	Mende	AFR	African Ancestry	MSL	1000 Genomes on GRCh38,1000 Genomes 30x on GRCh38,1000 Genomes phase 3 release
**HG03161**	male	SAME1839104	ESN	Esan	AFR	African Ancestry	ESN	1000 Genomes 30x on GRCh38
**HG03585**	male	SAME1839293	BEB	Bengali	SAS	South Asian Ancestry	BEB	1000 Genomes on GRCh38,1000 Genomes 30x on GRCh38,1000 Genomes phase 3 release
**HG03166**	male	SAME1839127	ESN	Esan	AFR	African Ancestry	ESN	1000 Genomes on GRCh38,1000 Genomes 30x on GRCh38,1000 Genomes phase 3 release
**HG03600**	male	SAME1840215	BEB	Bengali	SAS	South Asian Ancestry	BEB	1000 Genomes on GRCh38,1000 Genomes 30x on GRCh38,1000 Genomes phase 3 release
**HG03679**	male	SAME1839055	STU	Tamil	SAS	South Asian Ancestry	STU	1000 Genomes on GRCh38,1000 Genomes 30x on GRCh38,1000 Genomes phase 3 release
**HG03522**	female	SAME1840193	ESN	Esan	AFR	African Ancestry	ESN	1000 Genomes 30x on GRCh38
**HG03681**	male	SAME1840321	STU	Tamil	SAS	South Asian Ancestry	STU	1000 Genomes on GRCh38,1000 Genomes 30x on GRCh38,1000 Genomes phase 3 release
**HG03539**	female	SAME1840014	GWD	Gambian Mandinka	AFR	African Ancestry	GWD	1000 Genomes on GRCh38,1000 Genomes 30x on GRCh38,1000 Genomes phase 3 release,Gambian Genome Variation Project (GRCh38)
**HG03686**	male	SAME1840335	STU	Tamil	SAS	South Asian Ancestry	STU	1000 Genomes on GRCh38,1000 Genomes 30x on GRCh38,1000 Genomes phase 3 release
**HG03558**	female	SAME1839561	MSL	Mende	AFR	African Ancestry	MSL	1000 Genomes on GRCh38,1000 Genomes 30x on GRCh38,1000 Genomes phase 3 release
**HG03693**	male	SAME1840372	STU	Tamil	SAS	South Asian Ancestry	STU	1000 Genomes on GRCh38,1000 Genomes 30x on GRCh38,1000 Genomes phase 3 release
**HG03565**	male	SAME1839430	MSL	Mende	AFR	African Ancestry	MSL	1000 Genomes on GRCh38,1000 Genomes 30x on GRCh38,1000 Genomes phase 3 release
**HG03547**	male	SAME1839878	MSL	Mende	AFR	African Ancestry	MSL	1000 Genomes on GRCh38,1000 Genomes 30x on GRCh38,1000 Genomes phase 3 release
**HG03572**	female	SAME1839463	MSL	Mende	AFR	African Ancestry	MSL	1000 Genomes on GRCh38,1000 Genomes 30x on GRCh38,1000 Genomes phase 3 release
**HG03559**	male	SAME1839654	MSL	Mende	AFR	African Ancestry	MSL	1000 Genomes on GRCh38,1000 Genomes 30x on GRCh38,1000 Genomes phase 3 release
**HG03577**	male	SAME1839242	MSL	Mende	AFR	African Ancestry	MSL	1000 Genomes on GRCh38,1000 Genomes 30x on GRCh38,1000 Genomes phase 3 release
**HG03584**	female	SAME1839290	MSL	Mende	AFR	African Ancestry	MSL	1000 Genomes 30x on GRCh38,1000 Genomes phase 3 release
**HG03718**	male	SAME1839834	ITU	Telugu	SAS	South Asian Ancestry	ITU	1000 Genomes on GRCh38,1000 Genomes 30x on GRCh38,1000 Genomes phase 3 release
**HG03589**	female	SAME1839311	BEB	Bengali	SAS	South Asian Ancestry	BEB	1000 Genomes on GRCh38,1000 Genomes 30x on GRCh38,1000 Genomes phase 3 release
**HG03720**	male	SAME1839562	ITU	Telugu	SAS	South Asian Ancestry	ITU	1000 Genomes on GRCh38,1000 Genomes 30x on GRCh38,1000 Genomes phase 3 release
**HG03596**	male	SAME1839188	BEB	Bengali	SAS	South Asian Ancestry	BEB	1000 Genomes 30x on GRCh38
**HG03725**	male	SAME1839680	ITU	Telugu	SAS	South Asian Ancestry	ITU	1000 Genomes on GRCh38,1000 Genomes 30x on GRCh38
**HG03604**	female	SAME1840236	BEB	Bengali	SAS	South Asian Ancestry	BEB	1000 Genomes on GRCh38,1000 Genomes 30x on GRCh38,1000 Genomes phase 3 release
**HG03732**	male	SAME1839378	ITU	Telugu	SAS	South Asian Ancestry	ITU	1000 Genomes 30x on GRCh38,Human Genome Structural Variation Consortium, Phase 2
**HG03488**	female	SAME1839486	PJL	Punjabi	SAS	South Asian Ancestry	PJL	1000 Genomes on GRCh38,1000 Genomes 30x on GRCh38,1000 Genomes phase 3 release
**HG03744**	male	SAME1839265	STU	Tamil	SAS	South Asian Ancestry	STU	1000 Genomes on GRCh38,1000 Genomes 30x on GRCh38,1000 Genomes phase 3 release
**HG03490**	male	SAME1839259	PJL	Punjabi	SAS	South Asian Ancestry	PJL	1000 Genomes on GRCh38,1000 Genomes 30x on GRCh38,1000 Genomes phase 3 release
**HG03821**	male	SAME1839449	BEB	Bengali	SAS	South Asian Ancestry	BEB	1000 Genomes on GRCh38,1000 Genomes 30x on GRCh38,1000 Genomes phase 3 release
**HG03826**	female	SAME1839465	BEB	Bengali	SAS	South Asian Ancestry	BEB	1000 Genomes on GRCh38,1000 Genomes 30x on GRCh38,1000 Genomes phase 3 release
**HG03833**	male	SAME1839356	BEB	Bengali	SAS	South Asian Ancestry	BEB	1000 Genomes on GRCh38,1000 Genomes 30x on GRCh38,1000 Genomes phase 3 release
**HG03515**	male	SAME1840154	ESN	Esan	AFR	African Ancestry	ESN	1000 Genomes on GRCh38,1000 Genomes 30x on GRCh38,1000 Genomes phase 3 release
**HG03838**	female	SAME1839297	STU	Tamil	SAS	South Asian Ancestry	STU	1000 Genomes on GRCh38,1000 Genomes 30x on GRCh38,1000 Genomes phase 3 release
**HG03433**	male	SAME1840110	MSL	Mende	AFR	African Ancestry	MSL	1000 Genomes on GRCh38,1000 Genomes 30x on GRCh38,1000 Genomes phase 3 release
**HG03438**	female	SAME1840208	MSL	Mende	AFR	African Ancestry	MSL	1000 Genomes 30x on GRCh38
**HG03445**	male	SAME1839996	MSL	Mende	AFR	African Ancestry	MSL	1000 Genomes on GRCh38,1000 Genomes 30x on GRCh38,1000 Genomes phase 3 release
**HG03934**	female	SAME1840318	BEB	Bengali	SAS	South Asian Ancestry	BEB	1000 Genomes on GRCh38,1000 Genomes 30x on GRCh38,1000 Genomes phase 3 release
**HG03452**	female	SAME1840046	MSL	Mende	AFR	African Ancestry	MSL	1000 Genomes on GRCh38,1000 Genomes 30x on GRCh38,1000 Genomes phase 3 release
**HG03457**	male	SAME1839781	MSL	Mende	AFR	African Ancestry	MSL	1000 Genomes on GRCh38,1000 Genomes 30x on GRCh38,1000 Genomes phase 3 release
**HG03941**	male	SAME1840353	BEB	Bengali	SAS	South Asian Ancestry	BEB	1000 Genomes on GRCh38,1000 Genomes 30x on GRCh38,1000 Genomes phase 3 release
**HG03344**	male	SAME1840364	ESN	Esan	AFR	African Ancestry	ESN	1000 Genomes 30x on GRCh38
**HG03953**	male	SAME1840252	STU	Tamil	SAS	South Asian Ancestry	STU	1000 Genomes on GRCh38,1000 Genomes 30x on GRCh38,1000 Genomes phase 3 release
**HG03351**	female	SAME1840231	ESN	Esan	AFR	African Ancestry	ESN	1000 Genomes on GRCh38,1000 Genomes 30x on GRCh38,1000 Genomes phase 3 release
**HG03960**	male	SAME1839945	ITU	Telugu	SAS	South Asian Ancestry	ITU	1000 Genomes on GRCh38,1000 Genomes 30x on GRCh38,1000 Genomes phase 3 release
**HG03363**	female	SAME1839957	ESN	Esan	AFR	African Ancestry	ESN	1000 Genomes on GRCh38,1000 Genomes 30x on GRCh38,1000 Genomes phase 3 release
**HG03368**	male	SAME1840074	ESN	Esan	AFR	African Ancestry	ESN	1000 Genomes 30x on GRCh38
**HG03908**	male	SAME1839522	BEB	Bengali	SAS	South Asian Ancestry	BEB	1000 Genomes on GRCh38,1000 Genomes 30x on GRCh38,1000 Genomes phase 3 release
**HG03370**	male	SAME1839800	ESN	Esan	AFR	African Ancestry	ESN	1000 Genomes on GRCh38,1000 Genomes 30x on GRCh38,1000 Genomes phase 3 release
**HG03910**	female	SAME1839205	BEB	Bengali	SAS	South Asian Ancestry	BEB	1000 Genomes on GRCh38,1000 Genomes 30x on GRCh38,1000 Genomes phase 3 release
**HG03382**	male	SAME1839580	MSL	Mende	AFR	African Ancestry	MSL	1000 Genomes on GRCh38,1000 Genomes 30x on GRCh38,1000 Genomes phase 3 release
**HG03915**	female	SAME1839227	BEB	Bengali	SAS	South Asian Ancestry	BEB	1000 Genomes 30x on GRCh38
**HG03394**	male	SAME1839648	MSL	Mende	AFR	African Ancestry	MSL	1000 Genomes on GRCh38,1000 Genomes 30x on GRCh38,1000 Genomes phase 3 release
**HG03922**	female	SAME1839103	BEB	Bengali	SAS	South Asian Ancestry	BEB	1000 Genomes on GRCh38,1000 Genomes 30x on GRCh38,1000 Genomes phase 3 release
**HG03927**	female	SAME1839035	BEB	Bengali	SAS	South Asian Ancestry	BEB	1000 Genomes 30x on GRCh38
**HG03799**	female	SAME1839944	BEB	Bengali	SAS	South Asian Ancestry	BEB	1000 Genomes on GRCh38,1000 Genomes 30x on GRCh38
**HG03419**	female	SAME1839130	MSL	Mende	AFR	African Ancestry	MSL	1000 Genomes on GRCh38,1000 Genomes 30x on GRCh38,1000 Genomes phase 3 release
**HG03802**	female	SAME1839608	BEB	Bengali	SAS	South Asian Ancestry	BEB	1000 Genomes on GRCh38,1000 Genomes 30x on GRCh38,1000 Genomes phase 3 release
**HG03464**	female	SAME1839827	MSL	Mende	AFR	African Ancestry	MSL	1000 Genomes on GRCh38,1000 Genomes 30x on GRCh38,1000 Genomes phase 3 release
**HG03807**	female	SAME1839547	BEB	Bengali	SAS	South Asian Ancestry	BEB	1000 Genomes 30x on GRCh38,Human Genome Structural Variation Consortium, Phase 2
**HG03469**	male	SAME1839850	MSL	Mende	AFR	African Ancestry	MSL	1000 Genomes on GRCh38,1000 Genomes 30x on GRCh38,1000 Genomes phase 3 release
**HG03814**	female	SAME1839411	BEB	Bengali	SAS	South Asian Ancestry	BEB	1000 Genomes on GRCh38,1000 Genomes 30x on GRCh38,1000 Genomes phase 3 release
**HG03775**	male	SAME1840413	ITU	Telugu	SAS	South Asian Ancestry	ITU	1000 Genomes on GRCh38,1000 Genomes 30x on GRCh38,1000 Genomes phase 3 release
**HG03476**	female	SAME1839702	MSL	Mende	AFR	African Ancestry	MSL	1000 Genomes on GRCh38,1000 Genomes 30x on GRCh38,1000 Genomes phase 3 release
**HG03782**	female	SAME1840119	ITU	Telugu	SAS	South Asian Ancestry	ITU	1000 Genomes on GRCh38,1000 Genomes 30x on GRCh38,1000 Genomes phase 3 release
**HG03298**	male	SAME1839903	ESN	Esan	AFR	African Ancestry	ESN	1000 Genomes on GRCh38,1000 Genomes 30x on GRCh38,1000 Genomes phase 3 release
**HG03787**	female	SAME1840224	ITU	Telugu	SAS	South Asian Ancestry	ITU	1000 Genomes on GRCh38,1000 Genomes 30x on GRCh38,1000 Genomes phase 3 release
**HG03301**	male	SAME1839472	ESN	Esan	AFR	African Ancestry	ESN	1000 Genomes on GRCh38,1000 Genomes 30x on GRCh38,1000 Genomes phase 3 release
**HG03794**	male	SAME1839920	BEB	Bengali	SAS	South Asian Ancestry	BEB	1000 Genomes on GRCh38,1000 Genomes 30x on GRCh38
**HG03306**	female	SAME1839504	ESN	Esan	AFR	African Ancestry	ESN	1000 Genomes on GRCh38,1000 Genomes 30x on GRCh38
**HG03756**	female	SAME1839080	STU	Tamil	SAS	South Asian Ancestry	STU	1000 Genomes on GRCh38,1000 Genomes 30x on GRCh38,1000 Genomes phase 3 release
**HG03313**	male	SAME1839213	ESN	Esan	AFR	African Ancestry	ESN	1000 Genomes on GRCh38,1000 Genomes 30x on GRCh38,1000 Genomes phase 3 release
**HG03763**	male	SAME1839122	PJL	Punjabi	SAS	South Asian Ancestry	PJL	1000 Genomes 30x on GRCh38
**HG03611**	female	SAME1839929	BEB	Bengali	SAS	South Asian Ancestry	BEB	1000 Genomes on GRCh38,1000 Genomes 30x on GRCh38,1000 Genomes phase 3 release
**HG03770**	female	SAME1840388	ITU	Telugu	SAS	South Asian Ancestry	ITU	1000 Genomes on GRCh38,1000 Genomes 30x on GRCh38,1000 Genomes phase 3 release
**HG03616**	female	SAME1839955	BEB	Bengali	SAS	South Asian Ancestry	BEB	1000 Genomes on GRCh38,1000 Genomes 30x on GRCh38,1000 Genomes phase 3 release
**HG03635**	female	SAME1839578	PJL	Punjabi	SAS	South Asian Ancestry	PJL	1000 Genomes 30x on GRCh38
**HG03888**	female	SAME1840039	STU	Tamil	SAS	South Asian Ancestry	STU	1000 Genomes on GRCh38,1000 Genomes 30x on GRCh38,1000 Genomes phase 3 release
**HG03697**	male	SAME1840137	STU	Tamil	SAS	South Asian Ancestry	STU	1000 Genomes on GRCh38,1000 Genomes 30x on GRCh38,1000 Genomes phase 3 release
**HG03890**	male	SAME1839864	STU	Tamil	SAS	South Asian Ancestry	STU	1000 Genomes on GRCh38,1000 Genomes 30x on GRCh38,1000 Genomes phase 3 release
**HG03700**	female	SAME1840010	PJL	Punjabi	SAS	South Asian Ancestry	PJL	1000 Genomes on GRCh38,1000 Genomes 30x on GRCh38
**HG03895**	female	SAME1839888	STU	Tamil	SAS	South Asian Ancestry	STU	1000 Genomes on GRCh38,1000 Genomes 30x on GRCh38,1000 Genomes phase 3 release
**HG03705**	male	SAME1840051	PJL	Punjabi	SAS	South Asian Ancestry	PJL	1000 Genomes on GRCh38,1000 Genomes 30x on GRCh38,1000 Genomes phase 3 release
**HG03857**	female	SAME1840294	STU	Tamil	SAS	South Asian Ancestry	STU	1000 Genomes on GRCh38,1000 Genomes 30x on GRCh38,1000 Genomes phase 3 release
**HG03717**	female	SAME1839831	ITU	Telugu	SAS	South Asian Ancestry	ITU	1000 Genomes on GRCh38,1000 Genomes 30x on GRCh38,1000 Genomes phase 3 release
**HG03864**	male	SAME1840170	ITU	Telugu	SAS	South Asian Ancestry	ITU	1000 Genomes on GRCh38,1000 Genomes 30x on GRCh38,1000 Genomes phase 3 release
**HG03774**	female	SAME1840408	ITU	Telugu	SAS	South Asian Ancestry	ITU	1000 Genomes on GRCh38,1000 Genomes 30x on GRCh38,1000 Genomes phase 3 release
**HG03869**	male	SAME1840104	ITU	Telugu	SAS	South Asian Ancestry	ITU	1000 Genomes on GRCh38,1000 Genomes 30x on GRCh38,1000 Genomes phase 3 release
**HG03779**	male	SAME1840351	ITU	Telugu	SAS	South Asian Ancestry	ITU	1000 Genomes on GRCh38,1000 Genomes 30x on GRCh38,1000 Genomes phase 3 release
**HG03871**	male	SAME1840200	ITU	Telugu	SAS	South Asian Ancestry	ITU	1000 Genomes on GRCh38,1000 Genomes 30x on GRCh38,1000 Genomes phase 3 release
**HG03781**	female	SAME1840117	ITU	Telugu	SAS	South Asian Ancestry	ITU	1000 Genomes on GRCh38,1000 Genomes 30x on GRCh38,1000 Genomes phase 3 release
**HG04228**	male	SAME1839161	STU	Tamil	SAS	South Asian Ancestry	STU	1000 Genomes 30x on GRCh38
**HG03786**	male	SAME1840218	ITU	Telugu	SAS	South Asian Ancestry	ITU	1000 Genomes on GRCh38,1000 Genomes 30x on GRCh38,1000 Genomes phase 3 release
**HG04235**	male	SAME1840263	ITU	Telugu	SAS	South Asian Ancestry	ITU	1000 Genomes on GRCh38,1000 Genomes 30x on GRCh38,1000 Genomes phase 3 release
**HG03793**	female	SAME1839915	BEB	Bengali	SAS	South Asian Ancestry	BEB	1000 Genomes on GRCh38,1000 Genomes 30x on GRCh38,1000 Genomes phase 3 release
**NA06984**	male	SAME124763	CEU	CEPH	EUR	European Ancestry	CEU	1000 Genomes on GRCh38,1000 Genomes 30x on GRCh38,1000 Genomes phase 3 release,1000 Genomes phase 1 release,Geuvadis
**HG03825**	female	SAME1839462	BEB	Bengali	SAS	South Asian Ancestry	BEB	1000 Genomes 30x on GRCh38
**NA06989**	female	SAME124760	CEU	CEPH	EUR	European Ancestry	CEU	1000 Genomes on GRCh38,1000 Genomes 30x on GRCh38,1000 Genomes phase 3 release,1000 Genomes phase 1 release,Geuvadis
**HG03832**	female	SAME1839353	BEB	Bengali	SAS	South Asian Ancestry	BEB	1000 Genomes on GRCh38,1000 Genomes 30x on GRCh38,1000 Genomes phase 3 release
**NA06991**	female	SAME124936	CEU	CEPH	EUR	European Ancestry	CEU	1000 Genomes 30x on GRCh38,1000 Genomes phase 3 release

**HG03837**	male	SAME1839291	STU	Tamil	SAS	South Asian Ancestry	STU	1000 Genomes on GRCh38,1000 Genomes 30x on GRCh38,1000 Genomes phase 3 release
**NA07014**	female	SAME125162	CEU	CEPH	EUR	European Ancestry	CEU	1000 Genomes 30x on GRCh38,1000 Genomes phase 3 release
**HG03844**	male	SAME1839166	STU	Tamil	SAS	South Asian Ancestry	STU	1000 Genomes on GRCh38,1000 Genomes 30x on GRCh38,1000 Genomes phase 3 release
**NA07019**	female	SAME125152	CEU	CEPH	EUR	European Ancestry	CEU	1000 Genomes 30x on GRCh38,1000 Genomes phase 3 release
**HG03750**	male	SAME1839309	STU	Tamil	SAS	South Asian Ancestry	STU	1000 Genomes on GRCh38,1000 Genomes 30x on GRCh38,1000 Genomes phase 3 release
**HG04090**	female	SAME1839927	ITU	Telugu	SAS	South Asian Ancestry	ITU	1000 Genomes on GRCh38,1000 Genomes 30x on GRCh38,1000 Genomes phase 3 release
**HG03755**	male	SAME1839074	STU	Tamil	SAS	South Asian Ancestry	STU	1000 Genomes on GRCh38,1000 Genomes 30x on GRCh38,1000 Genomes phase 3 release
**HG04115**	male	SAME1839362	STU	Tamil	SAS	South Asian Ancestry	STU	1000 Genomes 30x on GRCh38
**HG03762**	female	SAME1839119	PJL	Punjabi	SAS	South Asian Ancestry	PJL	1000 Genomes on GRCh38,1000 Genomes 30x on GRCh38,1000 Genomes phase 3 release
**HG04122**	male	SAME1839230	STU	Tamil	SAS	South Asian Ancestry	STU	1000 Genomes 30x on GRCh38
**HG03767**	male	SAME1839141	PJL	Punjabi	SAS	South Asian Ancestry	PJL	1000 Genomes on GRCh38,1000 Genomes 30x on GRCh38,1000 Genomes phase 3 release
**HG04127**	male	SAME1839249	STU	Tamil	SAS	South Asian Ancestry	STU	1000 Genomes on GRCh38,1000 Genomes 30x on GRCh38
**HG03849**	female	SAME1839190	STU	Tamil	SAS	South Asian Ancestry	STU	1000 Genomes on GRCh38,1000 Genomes 30x on GRCh38,1000 Genomes phase 3 release
**HG04134**	male	SAME1839303	BEB	Bengali	SAS	South Asian Ancestry	BEB	1000 Genomes on GRCh38,1000 Genomes 30x on GRCh38,1000 Genomes phase 3 release
**HG03851**	male	SAME1840272	STU	Tamil	SAS	South Asian Ancestry	STU	1000 Genomes on GRCh38,1000 Genomes 30x on GRCh38,1000 Genomes phase 3 release
**NA07045**	female	SAME124187	CEU	CEPH	EUR	European Ancestry	CEU	1000 Genomes 30x on GRCh38,1000 Genomes phase 3 release
**HG03856**	male	SAME1840291	STU	Tamil	SAS	South Asian Ancestry	STU	1000 Genomes on GRCh38,1000 Genomes 30x on GRCh38,1000 Genomes phase 3 release
**NA07348**	female	SAME123567	CEU	CEPH	EUR	European Ancestry	CEU	1000 Genomes 30x on GRCh38,1000 Genomes phase 3 release
**HG03863**	female	SAME1840162	ITU	Telugu	SAS	South Asian Ancestry	ITU	1000 Genomes on GRCh38,1000 Genomes 30x on GRCh38,1000 Genomes phase 3 release
**NA10839**	female	SAME124551	CEU	CEPH	EUR	European Ancestry	CEU	1000 Genomes 30x on GRCh38,1000 Genomes phase 3 release
**HG03868**	female	SAME1840097	ITU	Telugu	SAS	South Asian Ancestry	ITU	1000 Genomes on GRCh38,1000 Genomes 30x on GRCh38,1000 Genomes phase 3 release
**NA10846**	male	SAME124758	CEU	CEPH	EUR	European Ancestry	CEU	1000 Genomes 30x on GRCh38,1000 Genomes phase 3 release
**HG03870**	male	SAME1840196	ITU	Telugu	SAS	South Asian Ancestry	ITU	1000 Genomes on GRCh38,1000 Genomes 30x on GRCh38,1000 Genomes phase 3 release
**HG04141**	female	SAME1839178	BEB	Bengali	SAS	South Asian Ancestry	BEB	1000 Genomes on GRCh38,1000 Genomes 30x on GRCh38,1000 Genomes phase 3 release
**HG03673**	female	SAME1839030	STU	Tamil	SAS	South Asian Ancestry	STU	1000 Genomes on GRCh38,1000 Genomes 30x on GRCh38,1000 Genomes phase 3 release
**HG04146**	male	SAME1839108	BEB	Bengali	SAS	South Asian Ancestry	BEB	1000 Genomes on GRCh38,1000 Genomes 30x on GRCh38,1000 Genomes phase 3 release
**HG03680**	male	SAME1840311	STU	Tamil	SAS	South Asian Ancestry	STU	1000 Genomes on GRCh38,1000 Genomes 30x on GRCh38,1000 Genomes phase 3 release
**HG04153**	female	SAME1840299	BEB	Bengali	SAS	South Asian Ancestry	BEB	1000 Genomes on GRCh38,1000 Genomes 30x on GRCh38,1000 Genomes phase 3 release
**HG03685**	male	SAME1840333	STU	Tamil	SAS	South Asian Ancestry	STU	1000 Genomes on GRCh38,1000 Genomes 30x on GRCh38,1000 Genomes phase 3 release
**HG04158**	male	SAME1840400	BEB	Bengali	SAS	South Asian Ancestry	BEB	1000 Genomes on GRCh38,1000 Genomes 30x on GRCh38,1000 Genomes phase 3 release
**HG03692**	female	SAME1840371	STU	Tamil	SAS	South Asian Ancestry	STU	1000 Genomes on GRCh38,1000 Genomes 30x on GRCh38,1000 Genomes phase 3 release
**HG04160**	male	SAME1840179	BEB	Bengali	SAS	South Asian Ancestry	BEB	1000 Genomes 30x on GRCh38
**HG03642**	female	SAME1839627	STU	Tamil	SAS	South Asian Ancestry	STU	1000 Genomes on GRCh38,1000 Genomes 30x on GRCh38,1000 Genomes phase 3 release
**HG04204**	male	SAME1839437	STU	Tamil	SAS	South Asian Ancestry	STU	1000 Genomes 30x on GRCh38
**HG03654**	male	SAME1839513	PJL	Punjabi	SAS	South Asian Ancestry	PJL	1000 Genomes 30x on GRCh38
**HG04209**	female	SAME1839452	ITU	Telugu	SAS	South Asian Ancestry	ITU	1000 Genomes on GRCh38,1000 Genomes 30x on GRCh38,1000 Genomes phase 3 release
**HG03798**	male	SAME1839941	BEB	Bengali	SAS	South Asian Ancestry	BEB	1000 Genomes 30x on GRCh38
**HG04211**	male	SAME1839321	ITU	Telugu	SAS	South Asian Ancestry	ITU	1000 Genomes on GRCh38,1000 Genomes 30x on GRCh38,1000 Genomes phase 3 release
**HG03801**	female	SAME1839606	BEB	Bengali	SAS	South Asian Ancestry	BEB	1000 Genomes 30x on GRCh38
**HG04216**	female	SAME1839339	ITU	Telugu	SAS	South Asian Ancestry	ITU	1000 Genomes on GRCh38,1000 Genomes 30x on GRCh38,1000 Genomes phase 3 release
**HG03806**	male	SAME1839542	BEB	Bengali	SAS	South Asian Ancestry	BEB	1000 Genomes on GRCh38,1000 Genomes 30x on GRCh38
**HG04177**	female	SAME1839992	BEB	Bengali	SAS	South Asian Ancestry	BEB	1000 Genomes on GRCh38,1000 Genomes 30x on GRCh38,1000 Genomes phase 3 release
**HG04184**	female	SAME1840049	BEB	Bengali	SAS	South Asian Ancestry	BEB	1000 Genomes 30x on GRCh38
**HG03729**	male	SAME1839701	ITU	Telugu	SAS	South Asian Ancestry	ITU	1000 Genomes on GRCh38,1000 Genomes 30x on GRCh38,1000 Genomes phase 3 release
**HG04189**	female	SAME1839783	BEB	Bengali	SAS	South Asian Ancestry	BEB	1000 Genomes on GRCh38,1000 Genomes 30x on GRCh38,1000 Genomes phase 3 release
**HG03731**	female	SAME1839372	ITU	Telugu	SAS	South Asian Ancestry	ITU	1000 Genomes on GRCh38,1000 Genomes 30x on GRCh38,1000 Genomes phase 3 release
**HG04191**	male	SAME1839897	BEB	Bengali	SAS	South Asian Ancestry	BEB	1000 Genomes on GRCh38,1000 Genomes 30x on GRCh38
**HG03736**	female	SAME1839389	STU	Tamil	SAS	South Asian Ancestry	STU	1000 Genomes on GRCh38,1000 Genomes 30x on GRCh38,1000 Genomes phase 3 release
**HG03965**	male	SAME1840063	ITU	Telugu	SAS	South Asian Ancestry	ITU	1000 Genomes on GRCh38,1000 Genomes 30x on GRCh38,1000 Genomes phase 3 release
**HG03743**	male	SAME1839261	STU	Tamil	SAS	South Asian Ancestry	STU	1000 Genomes on GRCh38,1000 Genomes 30x on GRCh38,1000 Genomes phase 3 release
**HG04094**	male	SAME1839953	ITU	Telugu	SAS	South Asian Ancestry	ITU	1000 Genomes on GRCh38,1000 Genomes 30x on GRCh38,1000 Genomes phase 3 release
**HG03977**	female	SAME1839746	ITU	Telugu	SAS	South Asian Ancestry	ITU	1000 Genomes on GRCh38,1000 Genomes 30x on GRCh38,1000 Genomes phase 3 release
**HG04099**	female	SAME1840065	STU	Tamil	SAS	South Asian Ancestry	STU	1000 Genomes on GRCh38,1000 Genomes 30x on GRCh38,1000 Genomes phase 3 release
**HG03989**	female	SAME1839532	STU	Tamil	SAS	South Asian Ancestry	STU	1000 Genomes on GRCh38,1000 Genomes 30x on GRCh38,1000 Genomes phase 3 release
**HG04107**	male	SAME1839660	STU	Tamil	SAS	South Asian Ancestry	STU	1000 Genomes on GRCh38,1000 Genomes 30x on GRCh38,1000 Genomes phase 3 release
**HG03991**	male	SAME1839640	STU	Tamil	SAS	South Asian Ancestry	STU	1000 Genomes on GRCh38,1000 Genomes 30x on GRCh38,1000 Genomes phase 3 release
**HG04038**	female	SAME1839499	STU	Tamil	SAS	South Asian Ancestry	STU	1000 Genomes on GRCh38,1000 Genomes 30x on GRCh38,1000 Genomes phase 3 release
**HG04133**	female	SAME1839294	BEB	Bengali	SAS	South Asian Ancestry	BEB	1000 Genomes 30x on GRCh38
**HG04076**	female	SAME1840358	ITU	Telugu	SAS	South Asian Ancestry	ITU	1000 Genomes on GRCh38,1000 Genomes 30x on GRCh38,1000 Genomes phase 3 release
**HG04056**	male	SAME1839102	ITU	Telugu	SAS	South Asian Ancestry	ITU	1000 Genomes on GRCh38,1000 Genomes 30x on GRCh38,1000 Genomes phase 3 release
**HG04002**	male	SAME1839769	ITU	Telugu	SAS	South Asian Ancestry	ITU	1000 Genomes on GRCh38,1000 Genomes 30x on GRCh38,1000 Genomes phase 3 release
**HG04063**	female	SAME1839155	ITU	Telugu	SAS	South Asian Ancestry	ITU	1000 Genomes on GRCh38,1000 Genomes 30x on GRCh38,1000 Genomes phase 3 release
**HG04014**	female	SAME1839844	ITU	Telugu	SAS	South Asian Ancestry	ITU	1000 Genomes on GRCh38,1000 Genomes 30x on GRCh38,1000 Genomes phase 3 release
**HG04070**	female	SAME1840411	ITU	Telugu	SAS	South Asian Ancestry	ITU	1000 Genomes on GRCh38,1000 Genomes 30x on GRCh38,1000 Genomes phase 3 release
**HG04019**	male	SAME1839572	ITU	Telugu	SAS	South Asian Ancestry	ITU	1000 Genomes on GRCh38,1000 Genomes 30x on GRCh38,1000 Genomes phase 3 release
**HG04075**	female	SAME1840359	STU	Tamil	SAS	South Asian Ancestry	STU	1000 Genomes on GRCh38,1000 Genomes 30x on GRCh38,1000 Genomes phase 3 release
**HG04026**	female	SAME1839622	ITU	Telugu	SAS	South Asian Ancestry	ITU	1000 Genomes on GRCh38,1000 Genomes 30x on GRCh38,1000 Genomes phase 3 release
**HG03875**	male	SAME1839965	ITU	Telugu	SAS	South Asian Ancestry	ITU	1000 Genomes on GRCh38,1000 Genomes 30x on GRCh38,1000 Genomes phase 3 release
**HG04033**	male	SAME1839466	STU	Tamil	SAS	South Asian Ancestry	STU	1000 Genomes on GRCh38,1000 Genomes 30x on GRCh38,1000 Genomes phase 3 release
**HG03882**	female	SAME1839999	ITU	Telugu	SAS	South Asian Ancestry	ITU	1000 Genomes on GRCh38,1000 Genomes 30x on GRCh38,1000 Genomes phase 3 release
**NA12878**	female	SAME123392	CEU	CEPH	EUR	European Ancestry	CEU	1000 Genomes on GRCh38,1000 Genomes 30x on GRCh38,Human Genome Structural Variation Consortium, Phase 2,Illumina Platinum pedigree,1000 Genomes phase 3
**HG03887**	male	SAME1840028	STU	Tamil	SAS	South Asian Ancestry	STU	1000 Genomes on GRCh38,1000 Genomes 30x on GRCh38,1000 Genomes phase 3 release
**NA12892**	female	SAME123181	CEU	CEPH	EUR	European Ancestry	CEU	1000 Genomes on GRCh38,1000 Genomes 30x on GRCh38,Illumina Platinum pedigree,1000 Genomes phase 3 release,1000 Genomes phase 1 release
**HG03894**	female	SAME1839885	STU	Tamil	SAS	South Asian Ancestry	STU	1000 Genomes on GRCh38,1000 Genomes 30x on GRCh38,1000 Genomes phase 3 release
**NA18484**	female	SAME124101	YRI	Yoruba	AFR	African Ancestry	YRI	1000 Genomes 30x on GRCh38,1000 Genomes phase 3 release
**HG03899**	male	SAME1839814	STU	Tamil	SAS	South Asian Ancestry	STU	1000 Genomes on GRCh38,1000 Genomes 30x on GRCh38,1000 Genomes phase 3 release
**NA18489**	female	SAME124102	YRI	Yoruba	AFR	African Ancestry	YRI	1000 Genomes on GRCh38,1000 Genomes 30x on GRCh38,1000 Genomes phase 3 release,1000 Genomes phase 1 release,Geuvadis
**HG03969**	male	SAME1840082	ITU	Telugu	SAS	South Asian Ancestry	ITU	1000 Genomes on GRCh38,1000 Genomes 30x on GRCh38,1000 Genomes phase 3 release
**NA18504**	male	SAME125061	YRI	Yoruba	AFR	African Ancestry	YRI	1000 Genomes on GRCh38,1000 Genomes 30x on GRCh38,1000 Genomes phase 3 release,1000 Genomes phase 1 release
**HG03971**	male	SAME1839718	ITU	Telugu	SAS	South Asian Ancestry	ITU	1000 Genomes on GRCh38,1000 Genomes 30x on GRCh38,1000 Genomes phase 3 release
**HG03976**	male	SAME1839739	ITU	Telugu	SAS	South Asian Ancestry	ITU	1000 Genomes on GRCh38,1000 Genomes 30x on GRCh38,1000 Genomes phase 3 release
**NA18511**	female	SAME124885	YRI	Yoruba	AFR	African Ancestry	YRI	1000 Genomes on GRCh38,1000 Genomes 30x on GRCh38,1000 Genomes phase 3 release,1000 Genomes phase 1 release,Geuvadis
**NA18516**	male	SAME124884	YRI	Yoruba	AFR	African Ancestry	YRI	1000 Genomes on GRCh38,1000 Genomes 30x on GRCh38,1000 Genomes phase 3 release,1000 Genomes phase 1 release
**HG03990**	male	SAME1839637	STU	Tamil	SAS	South Asian Ancestry	STU	1000 Genomes on GRCh38,1000 Genomes 30x on GRCh38,1000 Genomes phase 3 release
**NA18523**	female	SAME124670	YRI	Yoruba	AFR	African Ancestry	YRI	1000 Genomes on GRCh38,1000 Genomes 30x on GRCh38,1000 Genomes phase 3 release,1000 Genomes phase 1 release
**HG03995**	female	SAME1839395	STU	Tamil	SAS	South Asian Ancestry	STU	1000 Genomes on GRCh38,1000 Genomes 30x on GRCh38,1000 Genomes phase 3 release
**NA18528**	female	SAME124661	CHB	Han Chinese	EAS	East Asian Ancestry	CHB	1000 Genomes on GRCh38,1000 Genomes 30x on GRCh38,1000 Genomes phase 3 release,1000 Genomes phase 1 release
**HG03902**	male	SAME1839484	BEB	Bengali	SAS	South Asian Ancestry	BEB	1000 Genomes on GRCh38,1000 Genomes 30x on GRCh38,1000 Genomes phase 3 release
**NA18530**	male	SAME124484	CHB	Han Chinese	EAS	East Asian Ancestry	CHB	1000 Genomes on GRCh38,1000 Genomes 30x on GRCh38,1000 Genomes phase 3 release,1000 Genomes phase 1 release
**HG03907**	female	SAME1839517	BEB	Bengali	SAS	South Asian Ancestry	BEB	1000 Genomes on GRCh38,1000 Genomes 30x on GRCh38,1000 Genomes phase 3 release
**NA18535**	female	SAME124487	CHB	Han Chinese	EAS	East Asian Ancestry	CHB	1000 Genomes on GRCh38,1000 Genomes 30x on GRCh38,1000 Genomes phase 3 release,1000 Genomes phase 1 release
**HG03914**	male	SAME1839224	BEB	Bengali	SAS	South Asian Ancestry	BEB	1000 Genomes on GRCh38,1000 Genomes 30x on GRCh38,1000 Genomes phase 3 release
**NA18542**	female	SAME124267	CHB	Han Chinese	EAS	East Asian Ancestry	CHB	1000 Genomes on GRCh38,1000 Genomes 30x on GRCh38,1000 Genomes phase 3 release,1000 Genomes phase 1 release,90 Han Chinese high coverage genomes
**HG03919**	female	SAME1839329	BEB	Bengali	SAS	South Asian Ancestry	BEB	1000 Genomes on GRCh38,1000 Genomes 30x on GRCh38,1000 Genomes phase 3 release
**NA18547**	female	SAME124264	CHB	Han Chinese	EAS	East Asian Ancestry	CHB	1000 Genomes on GRCh38,1000 Genomes 30x on GRCh38,1000 Genomes phase 3 release,1000 Genomes phase 1 release,90 Han Chinese high coverage genomes
**HG03926**	male	SAME1839033	BEB	Bengali	SAS	South Asian Ancestry	BEB	1000 Genomes on GRCh38,1000 Genomes 30x on GRCh38,1000 Genomes phase 3 release
**NA18559**	male	SAME124093	CHB	Han Chinese	EAS	East Asian Ancestry	CHB	1000 Genomes on GRCh38,1000 Genomes 30x on GRCh38,1000 Genomes phase 3 release,1000 Genomes phase 1 release
**HG04164**	male	SAME1840102	BEB	Bengali	SAS	South Asian Ancestry	BEB	1000 Genomes on GRCh38,1000 Genomes 30x on GRCh38,1000 Genomes phase 3 release
**NA18561**	male	SAME123926	CHB	Han Chinese	EAS	East Asian Ancestry	CHB	1000 Genomes on GRCh38,1000 Genomes 30x on GRCh38,1000 Genomes phase 3 release,1000 Genomes phase 1 release,90 Han Chinese high coverage genomes
**HG04171**	female	SAME1839968	BEB	Bengali	SAS	South Asian Ancestry	BEB	1000 Genomes on GRCh38,1000 Genomes 30x on GRCh38,1000 Genomes phase 3 release
**NA18566**	female	SAME123923	CHB	Han Chinese	EAS	East Asian Ancestry	CHB	1000 Genomes on GRCh38,1000 Genomes 30x on GRCh38,1000 Genomes phase 3 release,1000 Genomes phase 1 release,90 Han Chinese high coverage genomes
**HG04176**	male	SAME1839986	BEB	Bengali	SAS	South Asian Ancestry	BEB	1000 Genomes on GRCh38,1000 Genomes 30x on GRCh38,1000 Genomes phase 3 release
**NA18573**	female	SAME123738	CHB	Han Chinese	EAS	East Asian Ancestry	CHB	1000 Genomes on GRCh38,1000 Genomes 30x on GRCh38,1000 Genomes phase 3 release,1000 Genomes phase 1 release,90 Han Chinese high coverage genomes
**HG04183**	female	SAME1840044	BEB	Bengali	SAS	South Asian Ancestry	BEB	1000 Genomes on GRCh38,1000 Genomes 30x on GRCh38,1000 Genomes phase 3 release
**NA18592**	female	SAME123721	CHB	Han Chinese	EAS	East Asian Ancestry	CHB	1000 Genomes on GRCh38,1000 Genomes 30x on GRCh38,1000 Genomes phase 3 release,1000 Genomes phase 1 release,90 Han Chinese high coverage genomes
**HG04188**	male	SAME1839771	BEB	Bengali	SAS	South Asian Ancestry	BEB	1000 Genomes on GRCh38,1000 Genomes 30x on GRCh38,1000 Genomes phase 3 release
**NA18597**	female	SAME123232	CHB	Han Chinese	EAS	East Asian Ancestry	CHB	1000 Genomes on GRCh38,1000 Genomes 30x on GRCh38,1000 Genomes phase 3 release,1000 Genomes phase 1 release
**HG04001**	female	SAME1839752	ITU	Telugu	SAS	South Asian Ancestry	ITU	1000 Genomes on GRCh38,1000 Genomes 30x on GRCh38,1000 Genomes phase 3 release
**NA18605**	male	SAME124877	CHB	Han Chinese	EAS	East Asian Ancestry	CHB	1000 Genomes on GRCh38,1000 Genomes 30x on GRCh38,1000 Genomes phase 3 release,1000 Genomes phase 1 release,90 Han Chinese high coverage genomes
**HG04006**	male	SAME1839787	STU	Tamil	SAS	South Asian Ancestry	STU	1000 Genomes on GRCh38,1000 Genomes 30x on GRCh38,1000 Genomes phase 3 release
**NA18612**	male	SAME125036	CHB	Han Chinese	EAS	East Asian Ancestry	CHB	1000 Genomes on GRCh38,1000 Genomes 30x on GRCh38,1000 Genomes phase 3 release,1000 Genomes phase 1 release,90 Han Chinese high coverage genomes

HG04018	female	SAME1839565	ITU	Telugu	SAS	South Asian Ancestry	ITU	1000 Genomes on GRCh38,1000 Genomes 30x on GRCh38,1000 Genomes phase 3 release
NA18617	female	SAME125033	CHB	Han Chinese	EAS	East Asian Ancestry	CHB	1000 Genomes on GRCh38,1000 Genomes 30x on GRCh38,1000 Genomes phase 3 release,1000 Genomes phase 1 release
HG04020	male	SAME1839685	ITU	Telugu	SAS	South Asian Ancestry	ITU	1000 Genomes on GRCh38,1000 Genomes 30x on GRCh38,1000 Genomes phase 3 release
NA18624	male	SAME124506	CHB	Han Chinese	EAS	East Asian Ancestry	CHB	1000 Genomes on GRCh38,1000 Genomes 30x on GRCh38,1000 Genomes phase 3 release,1000 Genomes phase 1 release,90 Han Chinese high coverage genomes
HG04025	female	SAME1839614	ITU	Telugu	SAS	South Asian Ancestry	ITU	1000 Genomes on GRCh38,1000 Genomes 30x on GRCh38,1000 Genomes phase 3 release
NA18629	male	SAME124501	CHB	Han Chinese	EAS	East Asian Ancestry	CHB	1000 Genomes on GRCh38,1000 Genomes 30x on GRCh38,1000 Genomes phase 3 release
HG04140	male	SAME1839173	BEB	Bengali	SAS	South Asian Ancestry	BEB	1000 Genomes on GRCh38,1000 Genomes 30x on GRCh38,1000 Genomes phase 3 release
NA18631	female	SAME124056	CHB	Han Chinese	EAS	East Asian Ancestry	CHB	1000 Genomes on GRCh38,1000 Genomes 30x on GRCh38,1000 Genomes phase 3 release,1000 Genomes phase 1 release
HG04152	male	SAME1840300	BEB	Bengali	SAS	South Asian Ancestry	BEB	1000 Genomes on GRCh38,1000 Genomes 30x on GRCh38,1000 Genomes phase 3 release
NA18636	male	SAME124320	CHB	Han Chinese	EAS	East Asian Ancestry	CHB	1000 Genomes on GRCh38,1000 Genomes 30x on GRCh38,1000 Genomes phase 3 release,1000 Genomes phase 1 release,90 Han Chinese high coverage genomes
HG04157	male	SAME1840395	BEB	Bengali	SAS	South Asian Ancestry	BEB	1000 Genomes 30x on GRCh38
NA18643	male	SAME122981	CHB	Han Chinese	EAS	East Asian Ancestry	CHB	1000 Genomes on GRCh38,1000 Genomes 30x on GRCh38,1000 Genomes phase 3 release,1000 Genomes phase 1 release
HG03940	female	SAME1840346	BEB	Bengali	SAS	South Asian Ancestry	BEB	1000 Genomes on GRCh38,1000 Genomes 30x on GRCh38,1000 Genomes phase 3 release
NA18648	male	SAME122974	CHB	Han Chinese	EAS	East Asian Ancestry	CHB	1000 Genomes on GRCh38,1000 Genomes 30x on GRCh38,1000 Genomes phase 3 release
HG03945	female	SAME1840369	STU	Tamil	SAS	South Asian Ancestry	STU	1000 Genomes on GRCh38,1000 Genomes 30x on GRCh38,1000 Genomes phase 3 release
NA18908	male	SAME123560	YRI	Yoruba	AFR	African Ancestry	YRI	1000 Genomes on GRCh38,1000 Genomes 30x on GRCh38,1000 Genomes phase 3 release,1000 Genomes phase 1 release,Geuvadis
NA18534	male	SAME124488	CHB	Han Chinese	EAS	East Asian Ancestry	CHB	1000 Genomes on GRCh38,1000 Genomes 30x on GRCh38,Human Genome Structural Variation Consortium, Phase 2,1000 Genomes phase 3 release,1000 Genomes ph
NA18910	male	SAME123402	YRI	Yoruba	AFR	African Ancestry	YRI	1000 Genomes on GRCh38,1000 Genomes 30x on GRCh38,1000 Genomes phase 3 release,1000 Genomes phase 1 release,Geuvadis
NA18539	female	SAME124475	CHB	Han Chinese	EAS	East Asian Ancestry	CHB	1000 Genomes on GRCh38,1000 Genomes 30x on GRCh38,1000 Genomes phase 3 release,1000 Genomes phase 1 release
NA18915	male	SAME123405	YRI	Yoruba	AFR	African Ancestry	YRI	1000 Genomes on GRCh38,1000 Genomes 30x on GRCh38,1000 Genomes phase 3 release
NA18541	female	SAME124270	CHB	Han Chinese	EAS	East Asian Ancestry	CHB	1000 Genomes on GRCh38,1000 Genomes 30x on GRCh38,1000 Genomes phase 3 release,1000 Genomes phase 1 release
NA18934	male	SAME123036	YRI	Yoruba	AFR	African Ancestry	YRI	1000 Genomes on GRCh38,1000 Genomes 30x on GRCh38,1000 Genomes phase 3 release,1000 Genomes phase 1 release,Geuvadis
NA18546	male	SAME124263	CHB	Han Chinese	EAS	East Asian Ancestry	CHB	1000 Genomes on GRCh38,1000 Genomes 30x on GRCh38,1000 Genomes phase 3 release,1000 Genomes phase 1 release
NA18939	female	SAME123041	JPT	Japanese	EAS	East Asian Ancestry	JPT	1000 Genomes on GRCh38,1000 Genomes 30x on GRCh38,Human Genome Structural Variation Consortium, Phase 2,1000 Genomes phase 3 release,1000 Genomes ph
NA18553	female	SAME124088	CHB	Han Chinese	EAS	East Asian Ancestry	CHB	1000 Genomes on GRCh38,1000 Genomes 30x on GRCh38,1000 Genomes phase 3 release,1000 Genomes phase 1 release
NA18941	female	SAME124311	JPT	Japanese	EAS	East Asian Ancestry	JPT	1000 Genomes on GRCh38,1000 Genomes 30x on GRCh38,1000 Genomes phase 3 release,1000 Genomes phase 1 release
NA18558	male	SAME124094	CHB	Han Chinese	EAS	East Asian Ancestry	CHB	1000 Genomes on GRCh38,1000 Genomes 30x on GRCh38,1000 Genomes phase 3 release,1000 Genomes phase 1 release,90 Han Chinese high coverage genomes
NA18946	female	SAME124312	JPT	Japanese	EAS	East Asian Ancestry	JPT	1000 Genomes on GRCh38,1000 Genomes 30x on GRCh38,1000 Genomes phase 3 release,1000 Genomes phase 1 release
NA18560	female	SAME123925	CHB	Han Chinese	EAS	East Asian Ancestry	CHB	1000 Genomes on GRCh38,1000 Genomes 30x on GRCh38,1000 Genomes phase 3 release,1000 Genomes phase 1 release
NA18953	male	SAME124113	JPT	Japanese	EAS	East Asian Ancestry	JPT	1000 Genomes on GRCh38,1000 Genomes 30x on GRCh38,1000 Genomes phase 3 release,1000 Genomes phase 1 release
NA18565	female	SAME123922	CHB	Han Chinese	EAS	East Asian Ancestry	CHB	1000 Genomes on GRCh38,1000 Genomes 30x on GRCh38,1000 Genomes phase 3 release,1000 Genomes phase 1 release
NA18960	male	SAME123956	JPT	Japanese	EAS	East Asian Ancestry	JPT	1000 Genomes on GRCh38,1000 Genomes 30x on GRCh38,1000 Genomes phase 3 release,1000 Genomes phase 1 release
NA18572	male	SAME123739	CHB	Han Chinese	EAS	East Asian Ancestry	CHB	1000 Genomes on GRCh38,1000 Genomes 30x on GRCh38,1000 Genomes phase 3 release,1000 Genomes phase 1 release,90 Han Chinese high coverage genomes
NA18965	male	SAME123961	JPT	Japanese	EAS	East Asian Ancestry	JPT	1000 Genomes on GRCh38,1000 Genomes 30x on GRCh38,1000 Genomes phase 3 release,1000 Genomes phase 1 release
NA18577	female	SAME123734	CHB	Han Chinese	EAS	East Asian Ancestry	CHB	1000 Genomes on GRCh38,1000 Genomes 30x on GRCh38,1000 Genomes phase 3 release,1000 Genomes phase 1 release,90 Han Chinese high coverage genomes
NA18591	female	SAME123719	CHB	Han Chinese	EAS	East Asian Ancestry	CHB	1000 Genomes on GRCh38,1000 Genomes 30x on GRCh38,1000 Genomes phase 3 release
NA10860	male	SAME125131	CEU	CEPH	EUR	European Ancestry	CEU	1000 Genomes 30x on GRCh38,1000 Genomes phase 3 release
NA18596	female	SAME123718	CHB	Han Chinese	EAS	East Asian Ancestry	CHB	1000 Genomes on GRCh38,1000 Genomes 30x on GRCh38,1000 Genomes phase 3 release,1000 Genomes phase 1 release
NA10865	male	SAME125128	CEU	CEPH	EUR	European Ancestry	CEU	1000 Genomes 30x on GRCh38,1000 Genomes phase 3 release
NA18609	male	SAME124867	CHB	Han Chinese	EAS	East Asian Ancestry	CHB	1000 Genomes on GRCh38,1000 Genomes 30x on GRCh38,1000 Genomes phase 3 release,1000 Genomes phase 1 release,90 Han Chinese high coverage genomes
NA11832	female	SAME124554	CEU	CEPH	EUR	European Ancestry	CEU	1000 Genomes on GRCh38,1000 Genomes 30x on GRCh38,1000 Genomes phase 3 release,1000 Genomes phase 1 release,Geuvadis
NA18611	male	SAME123225	CHB	Han Chinese	EAS	East Asian Ancestry	CHB	1000 Genomes on GRCh38,1000 Genomes 30x on GRCh38,1000 Genomes phase 3 release,1000 Genomes phase 1 release,90 Han Chinese high coverage genomes
NA11882	female	SAME123210	CEU	CEPH	EUR	European Ancestry	CEU	1000 Genomes 30x on GRCh38,1000 Genomes phase 3 release
NA18616	female	SAME125032	CHB	Han Chinese	EAS	East Asian Ancestry	CHB	1000 Genomes on GRCh38,1000 Genomes 30x on GRCh38,1000 Genomes phase 3 release,1000 Genomes phase 1 release
NA11894	female	SAME123043	CEU	CEPH	EUR	European Ancestry	CEU	1000 Genomes on GRCh38,1000 Genomes 30x on GRCh38,1000 Genomes phase 3 release,1000 Genomes phase 1 release,Geuvadis
NA18623	male	SAME124507	CHB	Han Chinese	EAS	East Asian Ancestry	CHB	1000 Genomes on GRCh38,1000 Genomes 30x on GRCh38,1000 Genomes phase 3 release,1000 Genomes phase 1 release,90 Han Chinese high coverage genomes
NA11919	male	SAME122834	CEU	CEPH	EUR	European Ancestry	CEU	1000 Genomes on GRCh38,1000 Genomes 30x on GRCh38,1000 Genomes phase 3 release,1000 Genomes phase 1 release
NA18628	female	SAME124502	CHB	Han Chinese	EAS	East Asian Ancestry	CHB	1000 Genomes on GRCh38,1000 Genomes 30x on GRCh38,1000 Genomes phase 3 release,1000 Genomes phase 1 release
NA11933	female	SAME125098	CEU	CEPH	EUR	European Ancestry	CEU	1000 Genomes on GRCh38,1000 Genomes 30x on GRCh38,1000 Genomes phase 3 release,1000 Genomes phase 1 release
NA18630	female	SAME124895	CHB	Han Chinese	EAS	East Asian Ancestry	CHB	1000 Genomes on GRCh38,1000 Genomes 30x on GRCh38,1000 Genomes phase 3 release,1000 Genomes phase 1 release
NA11995	female	SAME123968	CEU	CEPH	EUR	European Ancestry	CEU	1000 Genomes on GRCh38,1000 Genomes 30x on GRCh38,1000 Genomes phase 3 release,1000 Genomes phase 1 release,Geuvadis
NA18635	male	SAME123661	CHB	Han Chinese	EAS	East Asian Ancestry	CHB	1000 Genomes on GRCh38,1000 Genomes 30x on GRCh38,1000 Genomes phase 3 release,1000 Genomes phase 1 release,90 Han Chinese high coverage genomes
NA12006	female	SAME125145	CEU	CEPH	EUR	European Ancestry	CEU	1000 Genomes on GRCh38,1000 Genomes 30x on GRCh38,1000 Genomes phase 3 release,1000 Genomes phase 1 release,Geuvadis
NA18642	female	SAME122982	CHB	Han Chinese	EAS	East Asian Ancestry	CHB	1000 Genomes on GRCh38,1000 Genomes 30x on GRCh38,1000 Genomes phase 3 release,1000 Genomes phase 1 release
NA12044	female	SAME123212	CEU	CEPH	EUR	European Ancestry	CEU	1000 Genomes on GRCh38,1000 Genomes 30x on GRCh38,1000 Genomes phase 3 release,1000 Genomes phase 1 release,Geuvadis
NA18647	male	SAME122977	CHB	Han Chinese	EAS	East Asian Ancestry	CHB	1000 Genomes on GRCh38,1000 Genomes 30x on GRCh38,1000 Genomes phase 3 release,1000 Genomes phase 1 release
NA12056	male	SAME123414	CEU	CEPH	EUR	European Ancestry	CEU	1000 Genomes 30x on GRCh38,1000 Genomes phase 3 release
NA10852	female	SAME125320	CEU	CEPH	EUR	European Ancestry	CEU	1000 Genomes 30x on GRCh38,1000 Genomes phase 3 release
NA12145	female	SAME124157	CEU	CEPH	EUR	European Ancestry	CEU	1000 Genomes 30x on GRCh38,1000 Genomes phase 3 release
NA10857	male	SAME125332	CEU	CEPH	EUR	European Ancestry	CEU	1000 Genomes 30x on GRCh38,1000 Genomes phase 3 release
NA12234	female	SAME124003	CEU	CEPH	EUR	European Ancestry	CEU	1000 Genomes on GRCh38,1000 Genomes 30x on GRCh38,1000 Genomes phase 3 release,1000 Genomes phase 1 release,Geuvadis
NA10864	female	SAME125129	CEU	CEPH	EUR	European Ancestry	CEU	1000 Genomes 30x on GRCh38,1000 Genomes phase 3 release
NA12239	female	SAME123999	CEU	CEPH	EUR	European Ancestry	CEU	1000 Genomes 30x on GRCh38,1000 Genomes phase 3 release
NA11829	male	SAME124761	CEU	CEPH	EUR	European Ancestry	CEU	1000 Genomes on GRCh38,1000 Genomes 30x on GRCh38,1000 Genomes phase 3 release,1000 Genomes phase 1 release,Geuvadis
NA12272	male	SAME123231	CEU	CEPH	EUR	European Ancestry	CEU	1000 Genomes on GRCh38,1000 Genomes 30x on GRCh38,1000 Genomes phase 3 release,1000 Genomes phase 1 release,Geuvadis
NA11831	male	SAME124552	CEU	CEPH	EUR	European Ancestry	CEU	1000 Genomes on GRCh38,1000 Genomes 30x on GRCh38,1000 Genomes phase 3 release,1000 Genomes phase 1 release,Geuvadis
NA12335	male	SAME124097	CEU	CEPH	EUR	European Ancestry	CEU	1000 Genomes 30x on GRCh38,1000 Genomes phase 3 release
NA11843	male	SAME125140	CEU	CEPH	EUR	European Ancestry	CEU	1000 Genomes on GRCh38,1000 Genomes 30x on GRCh38,1000 Genomes phase 3 release,1000 Genomes phase 1 release,Geuvadis
NA12342	male	SAME125155	CEU	CEPH	EUR	European Ancestry	CEU	1000 Genomes on GRCh38,1000 Genomes 30x on GRCh38,1000 Genomes phase 3 release,1000 Genomes phase 1 release,Geuvadis
NA11881	male	SAME123209	CEU	CEPH	EUR	European Ancestry	CEU	1000 Genomes on GRCh38,1000 Genomes 30x on GRCh38,1000 Genomes phase 3 release,1000 Genomes phase 1 release,Geuvadis
NA12347	male	SAME125316	CEU	CEPH	EUR	European Ancestry	CEU	1000 Genomes on GRCh38,1000 Genomes 30x on GRCh38,1000 Genomes phase 3 release,1000 Genomes phase 1 release,Geuvadis
NA11893	male	SAME123044	CEU	CEPH	EUR	European Ancestry	CEU	1000 Genomes on GRCh38,1000 Genomes 30x on GRCh38,1000 Genomes phase 3 release,1000 Genomes phase 1 release,Geuvadis
NA12400	female	SAME123881	CEU	CEPH	EUR	European Ancestry	CEU	1000 Genomes on GRCh38,1000 Genomes 30x on GRCh38,1000 Genomes phase 3 release,1000 Genomes phase 1 release,Geuvadis
NA11918	female	SAME123135	CEU	CEPH	EUR	European Ancestry	CEU	1000 Genomes on GRCh38,1000 Genomes 30x on GRCh38,1000 Genomes phase 3 release,1000 Genomes phase 1 release,Geuvadis
NA11920	female	SAME124901	CEU	CEPH	EUR	European Ancestry	CEU	1000 Genomes on GRCh38,1000 Genomes 30x on GRCh38,1000 Genomes phase 3 release,1000 Genomes phase 1 release,Geuvadis
NA12739	male	SAME123529	CEU	CEPH	EUR	European Ancestry	CEU	1000 Genomes 30x on GRCh38,1000 Genomes phase 3 release
NA11932	male	SAME125097	CEU	CEPH	EUR	European Ancestry	CEU	1000 Genomes on GRCh38,1000 Genomes 30x on GRCh38,1000 Genomes phase 3 release,1000 Genomes phase 1 release
NA12753	female	SAME124647	CEU	CEPH	EUR	European Ancestry	CEU	1000 Genomes 30x on GRCh38,1000 Genomes phase 3 release
NA11994	male	SAME123967	CEU	CEPH	EUR	European Ancestry	CEU	1000 Genomes on GRCh38,1000 Genomes 30x on GRCh38,1000 Genomes phase 3 release,1000 Genomes phase 1 release,Geuvadis
NA12760	male	SAME124465	CEU	CEPH	EUR	European Ancestry	CEU	1000 Genomes on GRCh38,1000 Genomes 30x on GRCh38,1000 Genomes phase 3 release,1000 Genomes phase 1 release,Geuvadis
NA12005	male	SAME125143	CEU	CEPH	EUR	European Ancestry	CEU	1000 Genomes on GRCh38,1000 Genomes 30x on GRCh38,1000 Genomes phase 3 release,1000 Genomes phase 1 release,Geuvadis
NA12777	male	SAME125252	CEU	CEPH	EUR	European Ancestry	CEU	1000 Genomes on GRCh38,1000 Genomes 30x on GRCh38,1000 Genomes phase 3 release,1000 Genomes phase 1 release,Geuvadis
NA12877	male	SAME123391	CEU	CEPH	EUR	European Ancestry	CEU	1000 Genomes 30x on GRCh38,Illumina Platinum pedigree,1000 Genomes phase 3 release
NA12828	female	SAME125076	CEU	CEPH	EUR	European Ancestry	CEU	1000 Genomes on GRCh38,1000 Genomes 30x on GRCh38,1000 Genomes phase 3 release,1000 Genomes phase 1 release
NA12889	male	SAME123951	CEU	CEPH	EUR	European Ancestry	CEU	1000 Genomes on GRCh38,1000 Genomes 30x on GRCh38,Illumina Platinum pedigree,1000 Genomes phase 3 release,1000 Genomes phase 1 release,Geuvadis
NA12830	female	SAME125289	CEU	CEPH	EUR	European Ancestry	CEU	1000 Genomes on GRCh38,1000 Genomes 30x on GRCh38,1000 Genomes phase 3 release,1000 Genomes phase 1 release,Geuvadis
NA12891	male	SAME124117	CEU	CEPH	EUR	European Ancestry	CEU	1000 Genomes on GRCh38,1000 Genomes 30x on GRCh38,Illumina Platinum pedigree,1000 Genomes phase 3 release,1000 Genomes phase 1 release
NA12842	male	SAME123542	CEU	CEPH	EUR	European Ancestry	CEU	1000 Genomes on GRCh38,1000 Genomes 30x on GRCh38,1000 Genomes phase 3 release,1000 Genomes phase 1 release,Geuvadis
NA18488	female	SAME124103	YRI	Yoruba	AFR	African Ancestry	YRI	1000 Genomes on GRCh38,1000 Genomes 30x on GRCh38,1000 Genomes phase 3 release,1000 Genomes phase 1 release,Geuvadis
NA12873	female	SAME123388	CEU	CEPH	EUR	European Ancestry	CEU	1000 Genomes on GRCh38,1000 Genomes 30x on GRCh38,1000 Genomes phase 3 release,1000 Genomes phase 1 release,Geuvadis
NA18503	male	SAME125056	YRI	Yoruba	AFR	African Ancestry	YRI	1000 Genomes 30x on GRCh38,1000 Genomes phase 3 release
NA19026	male	SAME124849	LWK	Luhya	AFR	African Ancestry	LWK	1000 Genomes on GRCh38,1000 Genomes 30x on GRCh38,1000 Genomes phase 3 release
NA18508	female	SAME124318	YRI	Yoruba	AFR	African Ancestry	YRI	1000 Genomes on GRCh38,1000 Genomes 30x on GRCh38,1000 Genomes phase 3 release,1000 Genomes phase 1 release,Geuvadis
NA19038	female	SAME124668	LWK	Luhya	AFR	African Ancestry	LWK	1000 Genomes on GRCh38,1000 Genomes 30x on GRCh38,1000 Genomes phase 3 release,1000 Genomes phase 1 release
NA18510	male	SAME124886	YRI	Yoruba	AFR	African Ancestry	YRI	1000 Genomes on GRCh38,1000 Genomes 30x on GRCh38,1000 Genomes phase 3 release,1000 Genomes phase 1 release,Geuvadis
NA19057	female	SAME125025	JPT	Japanese	EAS	East Asian Ancestry	JPT	1000 Genomes on GRCh38,1000 Genomes 30x on GRCh38,1000 Genomes phase 3 release,1000 Genomes phase 1 release
NA18515	male	SAME124881	YRI	Yoruba	AFR	African Ancestry	YRI	1000 Genomes 30x on GRCh38,1000 Genomes phase 3 release
NA19064	female	SAME123010	JPT	Japanese	EAS	East Asian Ancestry	JPT	1000 Genomes on GRCh38,1000 Genomes 30x on GRCh38,1000 Genomes phase 3 release,1000 Genomes phase 1 release
NA18522	male	SAME124669	YRI	Yoruba	AFR	African Ancestry	YRI	1000 Genomes on GRCh38,1000 Genomes 30x on GRCh38,1000 Genomes phase 3 release,1000 Genomes phase 1 release
NA19076	male	SAME122819	JPT	Japanese	EAS	East Asian Ancestry	JPT	1000 Genomes on GRCh38,1000 Genomes 30x on GRCh38,1000 Genomes phase 3 release,1000 Genomes phase 1 release
NA19083	male	SAME123371	JPT	Japanese	EAS	East Asian Ancestry	JPT	1000 Genomes on GRCh38,1000 Genomes 30x on GRCh38,1000 Genomes phase 3 release,1000 Genomes phase 1 release
NA12707	male	SAME124080	CEU	CEPH	EUR	European Ancestry	CEU	1000 Genomes 30x on GRCh38,1000 Genomes phase 3 release
NA19088	male	SAME123360	JPT	Japanese	EAS	East Asian Ancestry	JPT	1000 Genomes on GRCh38,1000 Genomes 30x on GRCh38,1000 Genomes phase 3 release,1000 Genomes phase 1 release
NA12740	female	SAME124822	CEU	CEPH	EUR	European Ancestry	CEU	1000 Genomes 30x on GRCh38,1000 Genomes phase 3 release
NA19090	female	SAME123175	JPT	Japanese	EAS	East Asian Ancestry	JPT	1000 Genomes on GRCh38,1000 Genomes 30x on GRCh38,1000 Genomes phase 3 release
NA12752	male	SAME123518	CEU	CEPH	EUR	European Ancestry	CEU	1000 Genomes 30x on GRCh38,1000 Genomes phase 3 release
NA19095	female	SAME123170	YRI	Yoruba	AFR	African Ancestry	YRI	1000 Genomes on GRCh38,1000 Genomes 30x on GRCh38,1000 Genomes phase 3 release,1000 Genomes phase 1 release,Geuvadis
NA12776	female	SAME124256	CEU	CEPH	EUR	European Ancestry	CEU	1000 Genomes on GRCh38,1000 Genomes 30x on GRCh38,1000 Genomes phase 3 release,1000 Genomes phase 1 release,Geuvadis
NA18749	male	SAME123910	CHB	Han Chinese	EAS	East Asian Ancestry	CHB	1000 Genomes on GRCh38,1000 Genomes 30x on GRCh38,1000 Genomes phase 3 release,1000 Genomes phase 1 release
NA12815	female	SAME123022	CEU	CEPH	EUR	European Ancestry	CEU	1000 Genomes on GRCh38,1000 Genomes 30x on GRCh38,1000 Genomes phase 3 release,1000 Genomes phase 1 release,Geuvadis
NA12827	male	SAME125077	CEU	CEPH	EUR	European Ancestry	CEU	1000 Genomes on GRCh38,1000 Genomes 30x on GRCh38,1000 Genomes phase 3 release,1000 Genomes phase 1 release,Geuvadis

**NA18852**	female	SAME124732	YRI	Yoruba	AFR	African Ancestry	YRI	1000 Genomes 30x on GRCh38,1000 Genomes phase 3 release
**NA12865**	female	SAME123190	CEU	CEPH	EUR	European Ancestry	CEU	1000 Genomes 30x on GRCh38,1000 Genomes phase 3 release
**NA18857**	male	SAME124737	YRI	Yoruba	AFR	African Ancestry	YRI	1000 Genomes 30x on GRCh38,1000 Genomes phase 3 release
**NA12872**	male	SAME123387	CEU	CEPH	EUR	European Ancestry	CEU	1000 Genomes on GRCh38,1000 Genomes 30x on GRCh38,1000 Genomes phase 3 release,1000 Genomes phase 1 release,Geuvadis
**NA18864**	female	SAME124903	YRI	Yoruba	AFR	African Ancestry	YRI	1000 Genomes on GRCh38,1000 Genomes 30x on GRCh38,1000 Genomes phase 3 release
**NA12043**	male	SAME123214	CEU	CEPH	EUR	European Ancestry	CEU	1000 Genomes on GRCh38,1000 Genomes 30x on GRCh38,1000 Genomes phase 3 release,1000 Genomes phase 1 release,Geuvadis
**NA18869**	male	SAME124909	YRI	Yoruba	AFR	African Ancestry	YRI	1000 Genomes 30x on GRCh38,1000 Genomes phase 3 release
**NA12144**	male	SAME124156	CEU	CEPH	EUR	European Ancestry	CEU	1000 Genomes on GRCh38,1000 Genomes 30x on GRCh38,1000 Genomes phase 3 release,1000 Genomes phase 1 release,Geuvadis
**NA18871**	male	SAME125102	YRI	Yoruba	AFR	African Ancestry	YRI	1000 Genomes on GRCh38,1000 Genomes 30x on GRCh38,1000 Genomes phase 3 release,1000 Genomes phase 1 release
**NA12156**	female	SAME124952	CEU	CEPH	EUR	European Ancestry	CEU	1000 Genomes on GRCh38,1000 Genomes 30x on GRCh38,1000 Genomes phase 3 release,1000 Genomes phase 1 release,Geuvadis
**NA18876**	female	SAME125103	YRI	Yoruba	AFR	African Ancestry	YRI	1000 Genomes on GRCh38,1000 Genomes 30x on GRCh38,1000 Genomes phase 3 release
**NA12264**	male	SAME123087	CEU	CEPH	EUR	European Ancestry	CEU	1000 Genomes 30x on GRCh38,1000 Genomes phase 3 release
**NA18972**	female	SAME123762	JPT	Japanese	EAS	East Asian Ancestry	JPT	1000 Genomes on GRCh38,1000 Genomes 30x on GRCh38,1000 Genomes phase 3 release,1000 Genomes phase 1 release
**NA12283**	female	SAME124639	CEU	CEPH	EUR	European Ancestry	CEU	1000 Genomes on GRCh38,1000 Genomes 30x on GRCh38,1000 Genomes phase 3 release,1000 Genomes phase 1 release,Geuvadis
**NA18977**	male	SAME123765	JPT	Japanese	EAS	East Asian Ancestry	JPT	1000 Genomes on GRCh38,1000 Genomes 30x on GRCh38,1000 Genomes phase 3 release,1000 Genomes phase 1 release
**NA12341**	female	SAME125158	CEU	CEPH	EUR	European Ancestry	CEU	1000 Genomes on GRCh38,1000 Genomes 30x on GRCh38,1000 Genomes phase 3 release,1000 Genomes phase 1 release,Geuvadis
**NA18984**	male	SAME125071	JPT	Japanese	EAS	East Asian Ancestry	JPT	1000 Genomes on GRCh38,1000 Genomes 30x on GRCh38,1000 Genomes phase 3 release,1000 Genomes phase 1 release
**NA12485**	male	SAME125360	CEU	CEPH	EUR	European Ancestry	CEU	1000 Genomes 30x on GRCh38,1000 Genomes phase 3 release
**NA18989**	male	SAME125079	JPT	Japanese	EAS	East Asian Ancestry	JPT	1000 Genomes on GRCh38,1000 Genomes 30x on GRCh38,1000 Genomes phase 3 release,1000 Genomes phase 1 release
**NA07037**	female	SAME122862	CEU	CEPH	EUR	European Ancestry	CEU	1000 Genomes on GRCh38,1000 Genomes 30x on GRCh38,1000 Genomes phase 3 release,1000 Genomes phase 1 release,Geuvadis
**NA18991**	female	SAME124892	JPT	Japanese	EAS	East Asian Ancestry	JPT	1000 Genomes on GRCh38,1000 Genomes 30x on GRCh38,1000 Genomes phase 3 release,1000 Genomes phase 1 release
**NA07051**	male	SAME124378	CEU	CEPH	EUR	European Ancestry	CEU	1000 Genomes on GRCh38,1000 Genomes 30x on GRCh38,1000 Genomes phase 3 release,1000 Genomes phase 1 release,Geuvadis
**NA19002**	female	SAME124479	JPT	Japanese	EAS	East Asian Ancestry	JPT	1000 Genomes on GRCh38,1000 Genomes 30x on GRCh38,1000 Genomes phase 3 release,1000 Genomes phase 1 release
**NA07056**	female	SAME124375	CEU	CEPH	EUR	European Ancestry	CEU	1000 Genomes on GRCh38,1000 Genomes 30x on GRCh38,1000 Genomes phase 3 release,1000 Genomes phase 1 release,Geuvadis
**NA19007**	male	SAME124476	JPT	Japanese	EAS	East Asian Ancestry	JPT	1000 Genomes on GRCh38,1000 Genomes 30x on GRCh38,1000 Genomes phase 3 release,1000 Genomes phase 1 release
**NA07347**	male	SAME123572	CEU	CEPH	EUR	European Ancestry	CEU	1000 Genomes on GRCh38,1000 Genomes 30x on GRCh38,1000 Genomes phase 3 release,1000 Genomes phase 1 release,Geuvadis
**NA19019**	female	SAME124000	LWK	Luhya	AFR	African Ancestry	LWK	1000 Genomes on GRCh38,1000 Genomes 30x on GRCh38,1000 Genomes phase 3 release
**NA10838**	male	SAME124550	CEU	CEPH	EUR	European Ancestry	CEU	1000 Genomes 30x on GRCh38,1000 Genomes phase 3 release
**NA19153**	male	SAME123019	YRI	Yoruba	AFR	African Ancestry	YRI	1000 Genomes on GRCh38,1000 Genomes 30x on GRCh38,1000 Genomes phase 3 release,1000 Genomes phase 1 release,Geuvadis
**NA10840**	female	SAME124756	CEU	CEPH	EUR	European Ancestry	CEU	1000 Genomes 30x on GRCh38,1000 Genomes phase 3 release
**NA19160**	male	SAME123758	YRI	Yoruba	AFR	African Ancestry	YRI	1000 Genomes on GRCh38,1000 Genomes 30x on GRCh38,1000 Genomes phase 3 release,1000 Genomes phase 1 release,Geuvadis
**NA10845**	male	SAME124759	CEU	CEPH	EUR	European Ancestry	CEU	1000 Genomes 30x on GRCh38,1000 Genomes phase 3 release
**NA19172**	female	SAME124420	YRI	Yoruba	AFR	African Ancestry	YRI	1000 Genomes on GRCh38,1000 Genomes 30x on GRCh38,1000 Genomes phase 3 release,1000 Genomes phase 1 release,Geuvadis
**NA18748**	male	SAME123909	CHB	Han Chinese	EAS	East Asian Ancestry	CHB	1000 Genomes on GRCh38,1000 Genomes 30x on GRCh38,1000 Genomes phase 3 release,1000 Genomes phase 1 release
**NA19177**	male	SAME124417	YRI	Yoruba	AFR	African Ancestry	YRI	1000 Genomes 30x on GRCh38,1000 Genomes phase 3 release
**NA18856**	male	SAME124736	YRI	Yoruba	AFR	African Ancestry	YRI	1000 Genomes on GRCh38,1000 Genomes 30x on GRCh38,1000 Genomes phase 3 release,1000 Genomes phase 1 release
**NA19184**	male	SAME123859	YRI	Yoruba	AFR	African Ancestry	YRI	1000 Genomes on GRCh38,1000 Genomes 30x on GRCh38,1000 Genomes phase 3 release,1000 Genomes phase 1 release,Geuvadis
**NA18863**	male	SAME124907	YRI	Yoruba	AFR	African Ancestry	YRI	1000 Genomes 30x on GRCh38,1000 Genomes phase 3 release
**NA19189**	male	SAME123869	YRI	Yoruba	AFR	African Ancestry	YRI	1000 Genomes on GRCh38,1000 Genomes 30x on GRCh38,1000 Genomes phase 3 release,1000 Genomes phase 1 release,Geuvadis
**NA18868**	male	SAME124910	YRI	Yoruba	AFR	African Ancestry	YRI	1000 Genomes on GRCh38,1000 Genomes 30x on GRCh38,1000 Genomes phase 3 release,1000 Genomes phase 1 release,Geuvadis
**NA19191**	male	SAME124042	YRI	Yoruba	AFR	African Ancestry	YRI	1000 Genomes 30x on GRCh38,1000 Genomes phase 3 release
**NA18870**	female	SAME125101	YRI	Yoruba	AFR	African Ancestry	YRI	1000 Genomes on GRCh38,1000 Genomes 30x on GRCh38,1000 Genomes phase 3 release,1000 Genomes phase 1 release,Geuvadis
**NA18875**	female	SAME123545	YRI	Yoruba	AFR	African Ancestry	YRI	1000 Genomes 30x on GRCh38,1000 Genomes phase 3 release
**NA19204**	female	SAME122985	YRI	Yoruba	AFR	African Ancestry	YRI	1000 Genomes on GRCh38,1000 Genomes 30x on GRCh38,1000 Genomes phase 3 release,1000 Genomes phase 1 release,Geuvadis
**HG04222**	male	SAME1839040	ITU	Telugu	SAS	South Asian Ancestry	ITU	1000 Genomes on GRCh38,1000 Genomes 30x on GRCh38,1000 Genomes phase 3 release
**NA19209**	female	SAME122994	YRI	Yoruba	AFR	African Ancestry	YRI	1000 Genomes on GRCh38,1000 Genomes 30x on GRCh38,1000 Genomes phase 3 release,1000 Genomes phase 1 release,Geuvadis
**HG04227**	female	SAME1839056	STU	Tamil	SAS	South Asian Ancestry	STU	1000 Genomes on GRCh38,1000 Genomes 30x on GRCh38,1000 Genomes phase 3 release
**NA19211**	male	SAME125382	YRI	Yoruba	AFR	African Ancestry	YRI	1000 Genomes 30x on GRCh38,1000 Genomes phase 3 release
**HG04239**	male	SAME1840281	ITU	Telugu	SAS	South Asian Ancestry	ITU	1000 Genomes on GRCh38,1000 Genomes 30x on GRCh38,1000 Genomes phase 3 release
**NA19350**	male	SAME122853	LWK	Luhya	AFR	African Ancestry	LWK	1000 Genomes on GRCh38,1000 Genomes 30x on GRCh38,1000 Genomes phase 3 release,1000 Genomes phase 1 release
**NA06995**	male	SAME123994	CEU	CEPH	EUR	European Ancestry	CEU	1000 Genomes 30x on GRCh38,1000 Genomes phase 3 release
**NA19355**	female	SAME124077	LWK	Luhya	AFR	African Ancestry	LWK	1000 Genomes on GRCh38,1000 Genomes 30x on GRCh38,1000 Genomes phase 3 release,1000 Genomes phase 1 release
**HG04195**	female	SAME1839823	BEB	Bengali	SAS	South Asian Ancestry	BEB	1000 Genomes on GRCh38,1000 Genomes 30x on GRCh38,1000 Genomes phase 3 release
**NA19374**	male	SAME125115	LWK	Luhya	AFR	African Ancestry	LWK	1000 Genomes on GRCh38,1000 Genomes 30x on GRCh38,1000 Genomes phase 3 release,1000 Genomes phase 1 release
**NA19379**	female	SAME125092	LWK	Luhya	AFR	African Ancestry	LWK	1000 Genomes on GRCh38,1000 Genomes 30x on GRCh38,1000 Genomes phase 3 release,1000 Genomes phase 1 release
**HG04210**	male	SAME1839225	STU	Tamil	SAS	South Asian Ancestry	STU	1000 Genomes on GRCh38,1000 Genomes 30x on GRCh38,1000 Genomes phase 3 release
**HG04215**	male	SAME1839333	STU	Tamil	SAS	South Asian Ancestry	STU	1000 Genomes 30x on GRCh38
**NA19393**	male	SAME124496	LWK	Luhya	AFR	African Ancestry	LWK	1000 Genomes on GRCh38,1000 Genomes 30x on GRCh38,1000 Genomes phase 3 release,1000 Genomes phase 1 release
**NA19037**	female	SAME124665	LWK	Luhya	AFR	African Ancestry	LWK	1000 Genomes on GRCh38,1000 Genomes 30x on GRCh38,1000 Genomes phase 3 release
**NA19401**	female	SAME123634	LWK	Luhya	AFR	African Ancestry	LWK	1000 Genomes on GRCh38,1000 Genomes 30x on GRCh38,1000 Genomes phase 3 release,1000 Genomes phase 1 release
**NA19056**	male	SAME125026	JPT	Japanese	EAS	East Asian Ancestry	JPT	1000 Genomes on GRCh38,1000 Genomes 30x on GRCh38,1000 Genomes phase 3 release,1000 Genomes phase 1 release
**NA19063**	male	SAME123006	JPT	Japanese	EAS	East Asian Ancestry	JPT	1000 Genomes on GRCh38,1000 Genomes 30x on GRCh38,1000 Genomes phase 3 release,1000 Genomes phase 1 release
**NA19437**	female	SAME123851	LWK	Luhya	AFR	African Ancestry	LWK	1000 Genomes on GRCh38,1000 Genomes 30x on GRCh38,1000 Genomes phase 3 release,1000 Genomes phase 1 release
**NA19068**	male	SAME123012	JPT	Japanese	EAS	East Asian Ancestry	JPT	1000 Genomes on GRCh38,1000 Genomes 30x on GRCh38,1000 Genomes phase 3 release,1000 Genomes phase 1 release
**NA19070**	male	SAME122820	JPT	Japanese	EAS	East Asian Ancestry	JPT	1000 Genomes on GRCh38,1000 Genomes 30x on GRCh38,1000 Genomes phase 3 release,1000 Genomes phase 1 release
**NA19449**	female	SAME122946	LWK	Luhya	AFR	African Ancestry	LWK	1000 Genomes on GRCh38,1000 Genomes 30x on GRCh38,1000 Genomes phase 3 release,1000 Genomes phase 1 release
**NA19075**	male	SAME122816	JPT	Japanese	EAS	East Asian Ancestry	JPT	1000 Genomes on GRCh38,1000 Genomes 30x on GRCh38,1000 Genomes phase 3 release,1000 Genomes phase 1 release
**NA19451**	male	SAME125415	LWK	Luhya	AFR	African Ancestry	LWK	1000 Genomes on GRCh38,1000 Genomes 30x on GRCh38,1000 Genomes phase 3 release,1000 Genomes phase 1 release
**NA19082**	male	SAME123370	JPT	Japanese	EAS	East Asian Ancestry	JPT	1000 Genomes on GRCh38,1000 Genomes 30x on GRCh38,1000 Genomes phase 3 release,1000 Genomes phase 1 release
**NA19456**	female	SAME125410	LWK	Luhya	AFR	African Ancestry	LWK	1000 Genomes on GRCh38,1000 Genomes 30x on GRCh38,1000 Genomes phase 3 release,1000 Genomes phase 1 release
**NA19087**	female	SAME123367	JPT	Japanese	EAS	East Asian Ancestry	JPT	1000 Genomes on GRCh38,1000 Genomes 30x on GRCh38,1000 Genomes phase 3 release,1000 Genomes phase 1 release
**NA19463**	female	SAME123297	LWK	Luhya	AFR	African Ancestry	LWK	1000 Genomes on GRCh38,1000 Genomes 30x on GRCh38,1000 Genomes phase 3 release,1000 Genomes phase 1 release
**NA19094**	female	SAME123171	YRI	Yoruba	AFR	African Ancestry	YRI	1000 Genomes 30x on GRCh38,1000 Genomes phase 3 release
**NA19468**	female	SAME123299	LWK	Luhya	AFR	African Ancestry	LWK	1000 Genomes on GRCh38,1000 Genomes 30x on GRCh38,1000 Genomes phase 3 release,1000 Genomes phase 1 release
**NA19099**	female	SAME125240	YRI	Yoruba	AFR	African Ancestry	YRI	1000 Genomes on GRCh38,1000 Genomes 30x on GRCh38,1000 Genomes phase 3 release,1000 Genomes phase 1 release,Geuvadis
**NA19222**	female	SAME124147	YRI	Yoruba	AFR	African Ancestry	YRI	1000 Genomes on GRCh38,1000 Genomes 30x on GRCh38,1000 Genomes phase 3 release,1000 Genomes phase 1 release,Geuvadis
**NA19475**	female	SAME123121	LWK	Luhya	AFR	African Ancestry	LWK	1000 Genomes on GRCh38,1000 Genomes 30x on GRCh38,1000 Genomes phase 3 release
**NA19239**	male	SAME125081	YRI	Yoruba	AFR	African Ancestry	YRI	1000 Genomes on GRCh38,1000 Genomes 30x on GRCh38,Human Genome Structural Variation Consortium, Phase 2,The Human Genome Structural Variation Consortiu
**NA19653**	female	SAME123125	MXL	Mexican Ancestry	AMR	American Ancestry	MXL	1000 Genomes 30x on GRCh38,1000 Genomes phase 3 release
**NA19258**	male	SAME125045	YRI	Yoruba	AFR	African Ancestry	YRI	1000 Genomes 30x on GRCh38,1000 Genomes phase 3 release
**NA19658**	male	SAME123132	MXL	Mexican Ancestry	AMR	American Ancestry	MXL	1000 Genomes on GRCh38,1000 Genomes 30x on GRCh38,1000 Genomes phase 3 release,1000 Genomes phase 1 release
**NA19309**	male	SAME124984	LWK	Luhya	AFR	African Ancestry	LWK	1000 Genomes on GRCh38,1000 Genomes 30x on GRCh38,1000 Genomes phase 3 release,1000 Genomes phase 1 release
**NA19660**	female	SAME122958	MXL	Mexican Ancestry	AMR	American Ancestry	MXL	1000 Genomes on GRCh38,1000 Genomes 30x on GRCh38,1000 Genomes phase 3 release,1000 Genomes phase 1 release
**NA19665**	female	SAME122956	MXL	Mexican Ancestry	AMR	American Ancestry	MXL	1000 Genomes 30x on GRCh38,1000 Genomes phase 3 release
**NA19316**	female	SAME123311	LWK	Luhya	AFR	African Ancestry	LWK	1000 Genomes on GRCh38,1000 Genomes 30x on GRCh38,1000 Genomes phase 3 release,1000 Genomes phase 1 release
**NA19323**	female	SAME123471	LWK	Luhya	AFR	African Ancestry	LWK	1000 Genomes on GRCh38,1000 Genomes 30x on GRCh38,1000 Genomes phase 3 release
**NA19677**	female	SAME122789	MXL	Mexican Ancestry	AMR	American Ancestry	MXL	1000 Genomes 30x on GRCh38,1000 Genomes phase 3 release
**NA19328**	female	SAME123463	LWK	Luhya	AFR	African Ancestry	LWK	1000 Genomes on GRCh38,1000 Genomes 30x on GRCh38,1000 Genomes phase 3 release,1000 Genomes phase 1 release
**NA19684**	female	SAME122941	MXL	Mexican Ancestry	AMR	American Ancestry	MXL	1000 Genomes on GRCh38,1000 Genomes 30x on GRCh38,1000 Genomes phase 3 release,1000 Genomes phase 1 release
**NA19347**	male	SAME124340	LWK	Luhya	AFR	African Ancestry	LWK	1000 Genomes on GRCh38,1000 Genomes 30x on GRCh38,1000 Genomes phase 3 release,1000 Genomes phase 1 release
**NA19704**	female	SAME124236	ASW	African Ancestry SW	AFR	African Ancestry	ASW	1000 Genomes on GRCh38,1000 Genomes 30x on GRCh38,1000 Genomes phase 3 release,1000 Genomes phase 1 release
**NA19711**	male	SAME124446	ASW	African Ancestry SW	AFR	African Ancestry	ASW	1000 Genomes on GRCh38,1000 Genomes 30x on GRCh38,1000 Genomes phase 3 release,1000 Genomes phase 1 release
**NA19716**	female	SAME124450	MXL	Mexican Ancestry	AMR	American Ancestry	MXL	1000 Genomes on GRCh38,1000 Genomes 30x on GRCh38,1000 Genomes phase 3 release,1000 Genomes phase 1 release
**NA19378**	female	SAME125091	LWK	Luhya	AFR	African Ancestry	LWK	1000 Genomes on GRCh38,1000 Genomes 30x on GRCh38,1000 Genomes phase 3 release
**NA19723**	male	SAME124695	MXL	Mexican Ancestry	AMR	American Ancestry	MXL	1000 Genomes on GRCh38,1000 Genomes 30x on GRCh38,1000 Genomes phase 3 release,1000 Genomes phase 1 release
**NA19380**	male	SAME124276	LWK	Luhya	AFR	African Ancestry	LWK	1000 Genomes on GRCh38,1000 Genomes 30x on GRCh38,1000 Genomes phase 3 release,1000 Genomes phase 1 release
**NA19728**	female	SAME124702	MXL	Mexican Ancestry	AMR	American Ancestry	MXL	1000 Genomes on GRCh38,1000 Genomes 30x on GRCh38,1000 Genomes phase 3 release,1000 Genomes phase 1 release
**NA19385**	male	SAME124271	LWK	Luhya	AFR	African Ancestry	LWK	1000 Genomes on GRCh38,1000 Genomes 30x on GRCh38,1000 Genomes phase 3 release,1000 Genomes phase 1 release
**NA19730**	female	SAME124869	MXL	Mexican Ancestry	AMR	American Ancestry	MXL	1000 Genomes 30x on GRCh38,1000 Genomes phase 3 release
**NA19397**	male	SAME124492	LWK	Luhya	AFR	African Ancestry	LWK	1000 Genomes on GRCh38,1000 Genomes 30x on GRCh38,1000 Genomes phase 3 release,1000 Genomes phase 1 release
**NA19735**	male	SAME124874	MXL	Mexican Ancestry	AMR	American Ancestry	MXL	1000 Genomes on GRCh38,1000 Genomes 30x on GRCh38,1000 Genomes phase 3 release,1000 Genomes phase 1 release
**NA19429**	male	SAME124037	LWK	Luhya	AFR	African Ancestry	LWK	1000 Genomes on GRCh38,1000 Genomes 30x on GRCh38,1000 Genomes phase 3 release,1000 Genomes phase 1 release
**NA19785**	female	SAME123150	MXL	Mexican Ancestry	AMR	American Ancestry	MXL	1000 Genomes on GRCh38,1000 Genomes 30x on GRCh38,1000 Genomes phase 3 release,1000 Genomes phase 1 release
**NA19431**	female	SAME123853	LWK	Luhya	AFR	African Ancestry	LWK	1000 Genomes on GRCh38,1000 Genomes 30x on GRCh38,1000 Genomes phase 3 release,1000 Genomes phase 1 release
**NA19792**	male	SAME123327	MXL	Mexican Ancestry	AMR	American Ancestry	MXL	1000 Genomes on GRCh38,1000 Genomes 30x on GRCh38,1000 Genomes phase 3 release
**NA19436**	female	SAME123852	LWK	Luhya	AFR	African Ancestry	LWK	1000 Genomes on GRCh38,1000 Genomes 30x on GRCh38,1000 Genomes phase 3 release,1000 Genomes phase 1 release
**NA19443**	male	SAME122954	LWK	Luhya	AFR	African Ancestry	LWK	1000 Genomes on GRCh38,1000 Genomes 30x on GRCh38,1000 Genomes phase 3 release,1000 Genomes phase 1 release
**NA19836**	female	SAME125031	ASW	African Ancestry SW	AFR	African Ancestry	ASW	1000 Genomes 30x on GRCh38,1000 Genomes phase 3 release
**NA19448**	male	SAME122945	LWK	Luhya	AFR	African Ancestry	LWK	1000 Genomes on GRCh38,1000 Genomes 30x on GRCh38,1000 Genomes phase 3 release,1000 Genomes phase 1 release
**NA19901**	female	SAME125049	ASW	African Ancestry SW	AFR	African Ancestry	ASW	1000 Genomes on GRCh38,1000 Genomes 30x on GRCh38,1000 Genomes phase 3 release,1000 Genomes phase 1 release
**NA18907**	female	SAME123563	YRI	Yoruba	AFR	African Ancestry	YRI	1000 Genomes on GRCh38,1000 Genomes 30x on GRCh38,1000 Genomes phase 3 release,1000 Genomes phase 1 release,Geuvadis

**NA19913**	female	SAME124432	ASW	African Ancestry SW	AFR	African Ancestry	ASW	1000 Genomes on GRCh38,1000 Genomes 30x on GRCh38,1000 Genomes phase 3 release
**NA18914**	male	SAME123406	YRI	Yoruba	AFR	African Ancestry	YRI	1000 Genomes 30x on GRCh38,1000 Genomes phase 3 release
**NA19918**	male	SAME125275	ASW	African Ancestry SW	AFR	African Ancestry	ASW	1000 Genomes 30x on GRCh38,1000 Genomes phase 3 release
**NA18933**	female	SAME123037	YRI	Yoruba	AFR	African Ancestry	YRI	1000 Genomes on GRCh38,1000 Genomes 30x on GRCh38,1000 Genomes phase 3 release,1000 Genomes phase 1 release,Geuvadis
**NA19920**	male	SAME124713	ASW	African Ancestry SW	AFR	African Ancestry	ASW	1000 Genomes on GRCh38,1000 Genomes 30x on GRCh38,1000 Genomes phase 3 release,1000 Genomes phase 1 release
**NA18940**	male	SAME124310	JPT	Japanese,Japanese	EAS	East Asia (SGDP),East Asian Ancestry	JapaneseSGDP,JPT	1000 Genomes on GRCh38,Simons Genome Diversity Project,1000 Genomes 30x on GRCh38,1000 Genomes phase 3 release,1000 Genomes phase 1 release
**NA19982**	male	SAME124293	ASW	African Ancestry SW	AFR	African Ancestry	ASW	1000 Genomes on GRCh38,1000 Genomes 30x on GRCh38,1000 Genomes phase 3 release,1000 Genomes phase 1 release
**NA18945**	male	SAME124315	JPT	Japanese	EAS	East Asian Ancestry	JPT	1000 Genomes on GRCh38,1000 Genomes 30x on GRCh38,1000 Genomes phase 3 release,1000 Genomes phase 1 release
**NA19223**	male	SAME125223	YRI	Yoruba	AFR	African Ancestry	YRI	1000 Genomes on GRCh38,1000 Genomes 30x on GRCh38,1000 Genomes phase 3 release,1000 Genomes phase 1 release,Geuvadis
**NA18952**	male	SAME124114	JPT	Japanese	EAS	East Asian Ancestry	JPT	1000 Genomes on GRCh38,1000 Genomes 30x on GRCh38,1000 Genomes phase 3 release,1000 Genomes phase 1 release
**NA19235**	female	SAME125002	YRI	Yoruba	AFR	African Ancestry	YRI	1000 Genomes on GRCh38,1000 Genomes 30x on GRCh38,1000 Genomes phase 3 release,1000 Genomes phase 1 release,Geuvadis
**NA18957**	female	SAME124109	JPT	Japanese	EAS	East Asian Ancestry	JPT	1000 Genomes on GRCh38,1000 Genomes 30x on GRCh38,1000 Genomes phase 3 release,1000 Genomes phase 1 release
**NA19247**	female	SAME123691	YRI	Yoruba	AFR	African Ancestry	YRI	1000 Genomes on GRCh38,1000 Genomes 30x on GRCh38,1000 Genomes phase 3 release,1000 Genomes phase 1 release,Geuvadis
**NA18964**	female	SAME123960	JPT	Japanese	EAS	East Asian Ancestry	JPT	1000 Genomes on GRCh38,1000 Genomes 30x on GRCh38,1000 Genomes phase 3 release,1000 Genomes phase 1 release
**NA18969**	female	SAME123954	JPT	Japanese	EAS	East Asian Ancestry	JPT	1000 Genomes on GRCh38,1000 Genomes 30x on GRCh38,1000 Genomes phase 3 release,1000 Genomes phase 1 release
**NA19312**	male	SAME123315	LWK	Luhya	AFR	African Ancestry	LWK	1000 Genomes on GRCh38,1000 Genomes 30x on GRCh38,1000 Genomes phase 3 release,1000 Genomes phase 1 release
**NA18971**	male	SAME123763	JPT	Japanese	EAS	East Asian Ancestry	JPT	1000 Genomes on GRCh38,1000 Genomes 30x on GRCh38,1000 Genomes phase 3 release,1000 Genomes phase 1 release
**NA19317**	male	SAME123312	LWK	Luhya	AFR	African Ancestry	LWK	1000 Genomes on GRCh38,1000 Genomes 30x on GRCh38,1000 Genomes phase 3 release,1000 Genomes phase 1 release
**NA18976**	female	SAME123766	JPT	Japanese	EAS	East Asian Ancestry	JPT	1000 Genomes on GRCh38,1000 Genomes 30x on GRCh38,1000 Genomes phase 3 release,1000 Genomes phase 1 release
**NA19324**	female	SAME123472	LWK	Luhya	AFR	African Ancestry	LWK	1000 Genomes on GRCh38,1000 Genomes 30x on GRCh38,1000 Genomes phase 3 release,1000 Genomes phase 1 release
**NA18983**	male	SAME125074	JPT	Japanese	EAS	East Asian Ancestry	JPT	1000 Genomes on GRCh38,1000 Genomes 30x on GRCh38,1000 Genomes phase 3 release,1000 Genomes phase 1 release
**NA19331**	male	SAME123250	LWK	Luhya	AFR	African Ancestry	LWK	1000 Genomes on GRCh38,1000 Genomes 30x on GRCh38,1000 Genomes phase 3 release,1000 Genomes phase 1 release
**NA18988**	male	SAME125078	JPT	Japanese	EAS	East Asian Ancestry	JPT	1000 Genomes on GRCh38,1000 Genomes 30x on GRCh38,1000 Genomes phase 3 release,1000 Genomes phase 1 release
**NA19103**	male	SAME124330	YRI	Yoruba	AFR	African Ancestry	YRI	1000 Genomes 30x on GRCh38,1000 Genomes phase 3 release
**NA18990**	male	SAME124893	JPT	Japanese	EAS	East Asian Ancestry	JPT	1000 Genomes on GRCh38,1000 Genomes 30x on GRCh38,1000 Genomes phase 3 release,1000 Genomes phase 1 release
**NA19108**	female	SAME125378	YRI	Yoruba	AFR	African Ancestry	YRI	1000 Genomes on GRCh38,1000 Genomes 30x on GRCh38,1000 Genomes phase 3 release,1000 Genomes phase 1 release,Geuvadis
**NA18995**	male	SAME124896	JPT	Japanese	EAS	East Asian Ancestry	JPT	1000 Genomes on GRCh38,1000 Genomes 30x on GRCh38,1000 Genomes phase 3 release,1000 Genomes phase 1 release
**NA19115**	female	SAME123857	YRI	Yoruba	AFR	African Ancestry	YRI	1000 Genomes 30x on GRCh38,1000 Genomes phase 3 release
**NA19001**	female	SAME124478	JPT	Japanese	EAS	East Asian Ancestry	JPT	1000 Genomes on GRCh38,1000 Genomes 30x on GRCh38,1000 Genomes phase 3 release,1000 Genomes phase 1 release
**NA19122**	female	SAME125014	YRI	Yoruba	AFR	African Ancestry	YRI	1000 Genomes 30x on GRCh38,1000 Genomes phase 3 release
**NA19006**	male	SAME125119	JPT	Japanese	EAS	East Asian Ancestry	JPT	1000 Genomes on GRCh38,1000 Genomes 30x on GRCh38,1000 Genomes phase 3 release
**NA19127**	female	SAME125017	YRI	Yoruba	AFR	African Ancestry	YRI	1000 Genomes 30x on GRCh38,1000 Genomes phase 3 release
**NA19020**	male	SAME124855	LWK	Luhya	AFR	African Ancestry	LWK	1000 Genomes on GRCh38,1000 Genomes 30x on GRCh38,1000 Genomes phase 3 release,1000 Genomes phase 1 release
**NA19139**	male	SAME125237	YRI	Yoruba	AFR	African Ancestry	YRI	1000 Genomes 30x on GRCh38,1000 Genomes phase 3 release
**NA19025**	male	SAME124852	LWK	Luhya	AFR	African Ancestry	LWK	1000 Genomes on GRCh38,1000 Genomes 30x on GRCh38,1000 Genomes phase 3 release
**NA19141**	male	SAME124607	YRI	Yoruba	AFR	African Ancestry	YRI	1000 Genomes on GRCh38,1000 Genomes 30x on GRCh38,1000 Genomes phase 3 release,1000 Genomes phase 1 release,Geuvadis
**NA19152**	female	SAME123017	YRI	Yoruba	AFR	African Ancestry	YRI	1000 Genomes on GRCh38,1000 Genomes 30x on GRCh38,1000 Genomes phase 3 release,1000 Genomes phase 1 release,Geuvadis
**NA19146**	male	SAME125080	YRI	Yoruba	AFR	African Ancestry	YRI	1000 Genomes on GRCh38,1000 Genomes 30x on GRCh38,1000 Genomes phase 3 release,1000 Genomes phase 1 release,Geuvadis
**NA19171**	male	SAME124419	YRI	Yoruba	AFR	African Ancestry	YRI	1000 Genomes on GRCh38,1000 Genomes 30x on GRCh38,1000 Genomes phase 3 release,1000 Genomes phase 1 release,Geuvadis
**NA19176**	female	SAME124416	YRI	Yoruba	AFR	African Ancestry	YRI	1000 Genomes 30x on GRCh38,1000 Genomes phase 3 release
**NA19747**	male	SAME123501	MXL	Mexican Ancestry	AMR	American Ancestry	MXL	1000 Genomes on GRCh38,1000 Genomes 30x on GRCh38,1000 Genomes phase 3 release,1000 Genomes phase 1 release
**NA19190**	female	SAME124041	YRI	Yoruba	AFR	African Ancestry	YRI	1000 Genomes on GRCh38,1000 Genomes 30x on GRCh38,1000 Genomes phase 3 release,1000 Genomes phase 1 release,Geuvadis
**NA19759**	male	SAME123707	MXL	Mexican Ancestry	AMR	American Ancestry	MXL	1000 Genomes on GRCh38,1000 Genomes 30x on GRCh38,1000 Genomes phase 3 release,1000 Genomes phase 1 release
**NA19203**	male	SAME122988	YRI	Yoruba	AFR	African Ancestry	YRI	1000 Genomes 30x on GRCh38,1000 Genomes phase 3 release
**NA19761**	female	SAME123894	MXL	Mexican Ancestry	AMR	American Ancestry	MXL	1000 Genomes on GRCh38,1000 Genomes 30x on GRCh38,1000 Genomes phase 3 release,1000 Genomes phase 1 release
**NA19208**	male	SAME122993	YRI	Yoruba	AFR	African Ancestry	YRI	1000 Genomes 30x on GRCh38,1000 Genomes phase 3 release
**NA19210**	male	SAME125383	YRI	Yoruba	AFR	African Ancestry	YRI	1000 Genomes on GRCh38,1000 Genomes 30x on GRCh38,1000 Genomes phase 3 release,1000 Genomes phase 1 release,Geuvadis
**NA19773**	female	SAME124067	MXL	Mexican Ancestry	AMR	American Ancestry	MXL	1000 Genomes on GRCh38,1000 Genomes 30x on GRCh38,1000 Genomes phase 3 release,1000 Genomes phase 1 release
**NA19215**	female	SAME125385	YRI	Yoruba	AFR	African Ancestry	YRI	1000 Genomes 30x on GRCh38,1000 Genomes phase 3 release
**NA19778**	male	SAME124060	MXL	Mexican Ancestry	AMR	American Ancestry	MXL	1000 Genomes 30x on GRCh38,1000 Genomes phase 3 release
**NA19455**	male	SAME125411	LWK	Luhya	AFR	African Ancestry	LWK	1000 Genomes on GRCh38,1000 Genomes 30x on GRCh38,1000 Genomes phase 3 release,1000 Genomes phase 1 release
**NA19780**	male	SAME123147	MXL	Mexican Ancestry	AMR	American Ancestry	MXL	1000 Genomes on GRCh38,1000 Genomes 30x on GRCh38,1000 Genomes phase 3 release,1000 Genomes phase 1 release
**NA19462**	female	SAME123296	LWK	Luhya	AFR	African Ancestry	LWK	1000 Genomes on GRCh38,1000 Genomes 30x on GRCh38,1000 Genomes phase 3 release,1000 Genomes phase 1 release
**NA20129**	female	SAME122886	ASW	African Ancestry SW	AFR	African Ancestry	ASW	1000 Genomes 30x on GRCh38,1000 Genomes phase 3 release
**NA19467**	female	SAME123295	LWK	Luhya	AFR	African Ancestry	LWK	1000 Genomes on GRCh38,1000 Genomes 30x on GRCh38,1000 Genomes phase 3 release,1000 Genomes phase 1 release
**NA20282**	female	SAME124583	ASW	African Ancestry SW	AFR	African Ancestry	ASW	1000 Genomes on GRCh38,1000 Genomes 30x on GRCh38,1000 Genomes phase 3 release,1000 Genomes phase 1 release
**NA19474**	female	SAME123122	LWK	Luhya	AFR	African Ancestry	LWK	1000 Genomes on GRCh38,1000 Genomes 30x on GRCh38,1000 Genomes phase 3 release,1000 Genomes phase 1 release
**NA20287**	female	SAME124580	ASW	African Ancestry SW	AFR	African Ancestry	ASW	1000 Genomes on GRCh38,1000 Genomes 30x on GRCh38,1000 Genomes phase 3 release,1000 Genomes phase 1 release
**NA19652**	male	SAME124978	MXL	Mexican Ancestry	AMR	American Ancestry	MXL	1000 Genomes on GRCh38,1000 Genomes 30x on GRCh38,1000 Genomes phase 3 release,1000 Genomes phase 1 release
**NA20294**	female	SAME124776	ASW	African Ancestry SW	AFR	African Ancestry	ASW	1000 Genomes on GRCh38,1000 Genomes 30x on GRCh38,1000 Genomes phase 3 release,1000 Genomes phase 1 release
**NA19657**	female	SAME123129	MXL	Mexican Ancestry	AMR	American Ancestry	MXL	1000 Genomes on GRCh38,1000 Genomes 30x on GRCh38,1000 Genomes phase 3 release,1000 Genomes phase 1 release
**NA20299**	female	SAME124381	ASW	African Ancestry SW	AFR	African Ancestry	ASW	1000 Genomes on GRCh38,1000 Genomes 30x on GRCh38,1000 Genomes phase 3 release,1000 Genomes phase 1 release
**NA19664**	male	SAME122955	MXL	Mexican Ancestry	AMR	American Ancestry	MXL	1000 Genomes on GRCh38,1000 Genomes 30x on GRCh38,1000 Genomes phase 3 release,1000 Genomes phase 1 release
**NA19669**	female	SAME123903	MXL	Mexican Ancestry	AMR	American Ancestry	MXL	1000 Genomes on GRCh38,1000 Genomes 30x on GRCh38,1000 Genomes phase 3 release,1000 Genomes phase 1 release
**NA20314**	female	SAME125304	ASW	African Ancestry SW	AFR	African Ancestry	ASW	1000 Genomes on GRCh38,1000 Genomes 30x on GRCh38,1000 Genomes phase 3 release,1000 Genomes phase 1 release
**NA19671**	female	SAME122794	MXL	Mexican Ancestry	AMR	American Ancestry	MXL	1000 Genomes 30x on GRCh38,1000 Genomes phase 3 release
**NA19102**	female	SAME125112	YRI	Yoruba	AFR	African Ancestry	YRI	1000 Genomes on GRCh38,1000 Genomes 30x on GRCh38,1000 Genomes phase 3 release,1000 Genomes phase 1 release,Geuvadis
**NA19107**	male	SAME123393	YRI	Yoruba	AFR	African Ancestry	YRI	1000 Genomes on GRCh38,1000 Genomes 30x on GRCh38,1000 Genomes phase 3 release,1000 Genomes phase 1 release,Geuvadis
**NA20504**	female	SAME124360	TSI	Toscani	EUR	European Ancestry	TSI	1000 Genomes on GRCh38,1000 Genomes 30x on GRCh38,1000 Genomes phase 3 release,1000 Genomes phase 1 release,Geuvadis
**NA19114**	female	SAME122953	YRI	Yoruba	AFR	African Ancestry	YRI	1000 Genomes on GRCh38,1000 Genomes 30x on GRCh38,1000 Genomes phase 3 release,1000 Genomes phase 1 release,Geuvadis
**NA20509**	male	SAME124354	TSI	Toscani	EUR	European Ancestry	TSI	1000 Genomes on GRCh38,1000 Genomes 30x on GRCh38,Human Genome Structural Variation Consortium, Phase 2,1000 Genomes phase 3 release,1000 Genomes pha
**NA19119**	male	SAME122944	YRI	Yoruba	AFR	African Ancestry	YRI	1000 Genomes on GRCh38,1000 Genomes 30x on GRCh38,1000 Genomes phase 3 release,1000 Genomes phase 1 release,Geuvadis
**NA20511**	male	SAME124563	TSI	Toscani	EUR	European Ancestry	TSI	1000 Genomes on GRCh38,1000 Genomes 30x on GRCh38,1000 Genomes phase 3 release
**NA19121**	male	SAME125015	YRI	Yoruba	AFR	African Ancestry	YRI	1000 Genomes on GRCh38,1000 Genomes 30x on GRCh38,1000 Genomes phase 3 release,1000 Genomes phase 1 release,Geuvadis
**NA20516**	male	SAME124556	TSI	Toscani	EUR	European Ancestry	TSI	1000 Genomes on GRCh38,1000 Genomes 30x on GRCh38,1000 Genomes phase 3 release,1000 Genomes phase 1 release,Geuvadis
**NA19138**	male	SAME125236	YRI	Yoruba	AFR	African Ancestry	YRI	1000 Genomes on GRCh38,1000 Genomes 30x on GRCh38,1000 Genomes phase 3 release,1000 Genomes phase 1 release,Geuvadis
**NA20528**	male	SAME124012	TSI	Toscani	EUR	European Ancestry	TSI	1000 Genomes on GRCh38,1000 Genomes 30x on GRCh38,1000 Genomes phase 3 release,1000 Genomes phase 1 release,Geuvadis
**NA19140**	female	SAME124608	YRI	Yoruba	AFR	African Ancestry	YRI	1000 Genomes 30x on GRCh38,1000 Genomes phase 3 release
**NA20530**	female	SAME124169	TSI	Toscani	EUR	European Ancestry	TSI	1000 Genomes on GRCh38,1000 Genomes 30x on GRCh38,1000 Genomes phase 3 release,1000 Genomes phase 1 release,Geuvadis
**NA19145**	male	SAME123372	YRI	Yoruba	AFR	African Ancestry	YRI	1000 Genomes 30x on GRCh38,1000 Genomes phase 3 release
**NA20535**	female	SAME124166	TSI	Toscani	EUR	European Ancestry	TSI	1000 Genomes on GRCh38,1000 Genomes 30x on GRCh38,1000 Genomes phase 3 release,1000 Genomes phase 1 release,Geuvadis
**NA19676**	male	SAME122790	MXL	Mexican Ancestry	AMR	American Ancestry	MXL	1000 Genomes on GRCh38,1000 Genomes 30x on GRCh38,1000 Genomes phase 3 release,1000 Genomes phase 1 release
**NA20542**	female	SAME125150	TSI	Toscani	EUR	European Ancestry	TSI	1000 Genomes on GRCh38,1000 Genomes 30x on GRCh38,1000 Genomes phase 3 release,1000 Genomes phase 1 release,Geuvadis
**NA19683**	female	SAME122936	MXL	Mexican Ancestry	AMR	American Ancestry	MXL	1000 Genomes 30x on GRCh38,1000 Genomes phase 3 release
**NA20585**	female	SAME122861	TSI	Toscani	EUR	European Ancestry	TSI	1000 Genomes on GRCh38,1000 Genomes 30x on GRCh38,1000 Genomes phase 3 release,1000 Genomes phase 1 release,Geuvadis
**NA19703**	male	SAME124230	ASW	African Ancestry SW	AFR	African Ancestry	ASW	1000 Genomes on GRCh38,1000 Genomes 30x on GRCh38,1000 Genomes phase 3 release,1000 Genomes phase 1 release
**NA20756**	female	SAME124175	TSI	Toscani	EUR	European Ancestry	TSI	1000 Genomes on GRCh38,1000 Genomes 30x on GRCh38,1000 Genomes phase 3 release,1000 Genomes phase 1 release,Geuvadis
**NA20763**	male	SAME124365	TSI	Toscani	EUR	European Ancestry	TSI	1000 Genomes on GRCh38,1000 Genomes 30x on GRCh38,1000 Genomes phase 3 release
**NA19722**	female	SAME124696	MXL	Mexican Ancestry	AMR	American Ancestry	MXL	1000 Genomes on GRCh38,1000 Genomes 30x on GRCh38,1000 Genomes phase 3 release,1000 Genomes phase 1 release
**NA20768**	female	SAME124372	TSI	Toscani	EUR	European Ancestry	TSI	1000 Genomes on GRCh38,1000 Genomes 30x on GRCh38,1000 Genomes phase 3 release,1000 Genomes phase 1 release,Geuvadis
**NA19727**	male	SAME123639	MXL	Mexican Ancestry	AMR	American Ancestry	MXL	1000 Genomes 30x on GRCh38,1000 Genomes phase 3 release
**NA20770**	male	SAME124576	TSI	Toscani	EUR	European Ancestry	TSI	1000 Genomes on GRCh38,1000 Genomes 30x on GRCh38,1000 Genomes phase 3 release,1000 Genomes phase 1 release,Geuvadis
**NA19734**	female	SAME124873	MXL	Mexican Ancestry	AMR	American Ancestry	MXL	1000 Genomes on GRCh38,1000 Genomes 30x on GRCh38,1000 Genomes phase 3 release,1000 Genomes phase 1 release
**NA20775**	female	SAME124573	TSI	Toscani	EUR	European Ancestry	TSI	1000 Genomes on GRCh38,1000 Genomes 30x on GRCh38,1000 Genomes phase 3 release,1000 Genomes phase 1 release
**NA20787**	male	SAME123609	TSI	Toscani	EUR	European Ancestry	TSI	1000 Genomes on GRCh38,1000 Genomes 30x on GRCh38,1000 Genomes phase 3 release,1000 Genomes phase 1 release,Geuvadis
**NA20503**	female	SAME124357	TSI	Toscani	EUR	European Ancestry	TSI	1000 Genomes on GRCh38,1000 Genomes 30x on GRCh38,1000 Genomes phase 3 release,1000 Genomes phase 1 release,Geuvadis
**NA20799**	female	SAME123822	TSI	Toscani	EUR	European Ancestry	TSI	1000 Genomes on GRCh38,1000 Genomes 30x on GRCh38,1000 Genomes phase 3 release,1000 Genomes phase 1 release,Geuvadis
**NA20508**	female	SAME124355	TSI	Toscani	EUR	European Ancestry	TSI	1000 Genomes on GRCh38,1000 Genomes 30x on GRCh38,1000 Genomes phase 3 release,1000 Genomes phase 1 release,Geuvadis
**NA20802**	female	SAME125366	TSI	Toscani	EUR	European Ancestry	TSI	1000 Genomes on GRCh38,1000 Genomes 30x on GRCh38,1000 Genomes phase 3 release,1000 Genomes phase 1 release,Geuvadis
**NA20510**	male	SAME124562	TSI	Toscani	EUR	European Ancestry	TSI	1000 Genomes on GRCh38,1000 Genomes 30x on GRCh38,1000 Genomes phase 3 release,1000 Genomes phase 1 release,Geuvadis
**NA20807**	female	SAME125363	TSI	Toscani	EUR	European Ancestry	TSI	1000 Genomes on GRCh38,1000 Genomes 30x on GRCh38,1000 Genomes phase 3 release,1000 Genomes phase 1 release,Geuvadis
**NA20515**	male	SAME124559	TSI	Toscani	EUR	European Ancestry	TSI	1000 Genomes on GRCh38,1000 Genomes 30x on GRCh38,1000 Genomes phase 3 release,1000 Genomes phase 1 release,Geuvadis
**NA20814**	male	SAME125188	TSI	Toscani	EUR	European Ancestry	TSI	1000 Genomes on GRCh38,1000 Genomes 30x on GRCh38,1000 Genomes phase 3 release,1000 Genomes phase 1 release,Geuvadis
**NA20522**	female	SAME124017	TSI	Toscani	EUR	European Ancestry	TSI	1000 Genomes on GRCh38,1000 Genomes 30x on GRCh38,1000 Genomes phase 3 release,1000 Genomes phase 1 release
**NA20819**	female	SAME125183	TSI	Toscani	EUR	European Ancestry	TSI	1000 Genomes on GRCh38,1000 Genomes 30x on GRCh38,1000 Genomes phase 3 release,1000 Genomes phase 1 release,Geuvadis
**NA20527**	male	SAME124013	TSI	Toscani	EUR	European Ancestry	TSI	1000 Genomes on GRCh38,1000 Genomes 30x on GRCh38,1000 Genomes phase 3 release,1000 Genomes phase 1 release,Geuvadis
**NA20821**	female	SAME123075	TSI	Toscani	EUR	European Ancestry	TSI	1000 Genomes on GRCh38,1000 Genomes 30x on GRCh38,1000 Genomes phase 3 release
**NA20534**	male	SAME124165	TSI	Toscani	EUR	European Ancestry	TSI	1000 Genomes on GRCh38,1000 Genomes 30x on GRCh38,1000 Genomes phase 3 release,1000 Genomes phase 1 release,Geuvadis
**NA20826**	female	SAME123070	TSI	Toscani	EUR	European Ancestry	TSI	1000 Genomes on GRCh38,1000 Genomes 30x on GRCh38,1000 Genomes phase 3 release,1000 Genomes phase 1 release,Geuvadis
**NA20539**	male	SAME124164	TSI	Toscani	EUR	European Ancestry	TSI	1000 Genomes on GRCh38,1000 Genomes 30x on GRCh38,1000 Genomes phase 3 release,1000 Genomes phase 1 release,Geuvadis
**NA20845**	male	SAME123430	GIH	Gujarati	SAS	South Asian Ancestry	GIH	1000 Genomes on GRCh38,1000 Genomes 30x on GRCh38,1000 Genomes phase 3 release

**NA20541**	female	SAME125147	TSI	Toscani	EUR	European Ancestry	TSI	1000 Genomes on GRCh38,1000 Genomes 30x on GRCh38,1000 Genomes phase 3 release,1000 Genomes phase 1 release,Geuvadis
**NA20589**	female	SAME122865	TSI	Toscani	EUR	European Ancestry	TSI	1000 Genomes on GRCh38,1000 Genomes 30x on GRCh38,1000 Genomes phase 3 release,1000 Genomes phase 1 release,Geuvadis
**NA21141**	female	SAME125205	GIH	Gujarati	SAS	South Asian Ancestry	GIH	1000 Genomes on GRCh38,1000 Genomes 30x on GRCh38,1000 Genomes phase 3 release
**NA20755**	male	SAME124174	TSI	Toscani	EUR	European Ancestry	TSI	1000 Genomes on GRCh38,1000 Genomes 30x on GRCh38,1000 Genomes phase 3 release,1000 Genomes phase 1 release
**NA19784**	male	SAME123151	MXL	Mexican Ancestry	AMR	American Ancestry	MXL	1000 Genomes 30x on GRCh38,1000 Genomes phase 3 release
**NA19789**	male	SAME123152	MXL	Mexican Ancestry	AMR	American Ancestry	MXL	1000 Genomes on GRCh38,1000 Genomes 30x on GRCh38,1000 Genomes phase 3 release,1000 Genomes phase 1 release
**NA20321**	female	SAME123996	ASW	African Ancestry SW	AFR	African Ancestry	ASW	1000 Genomes on GRCh38,1000 Genomes 30x on GRCh38,1000 Genomes phase 3 release
**NA19796**	male	SAME123330	MXL	Mexican Ancestry	AMR	American Ancestry	MXL	1000 Genomes 30x on GRCh38,1000 Genomes phase 3 release
**NA19828**	male	SAME125246	ASW	African Ancestry SW	AFR	African Ancestry	ASW	1000 Genomes 30x on GRCh38,1000 Genomes phase 3 release
**NA20340**	male	SAME124400	ASW	African Ancestry SW	AFR	African Ancestry	ASW	1000 Genomes on GRCh38,1000 Genomes 30x on GRCh38,1000 Genomes phase 3 release,1000 Genomes phase 1 release
**NA19835**	female	SAME125029	ASW	African Ancestry SW	AFR	African Ancestry	ASW	1000 Genomes on GRCh38,1000 Genomes 30x on GRCh38,1000 Genomes phase 3 release,1000 Genomes phase 1 release
**NA19900**	male	SAME125050	ASW	African Ancestry SW	AFR	African Ancestry	ASW	1000 Genomes on GRCh38,1000 Genomes 30x on GRCh38,1000 Genomes phase 3 release,1000 Genomes phase 1 release
**NA20357**	female	SAME124599	ASW	African Ancestry SW	AFR	African Ancestry	ASW	1000 Genomes on GRCh38,1000 Genomes 30x on GRCh38,1000 Genomes phase 3 release,1000 Genomes phase 1 release
**NA20895**	male	SAME123666	GIH	Gujarati	SAS	South Asian Ancestry	GIH	1000 Genomes on GRCh38,1000 Genomes 30x on GRCh38,1000 Genomes phase 3 release
**NA19917**	female	SAME125272	ASW	African Ancestry SW	AFR	African Ancestry	ASW	1000 Genomes on GRCh38,1000 Genomes 30x on GRCh38,1000 Genomes phase 3 release,1000 Genomes phase 1 release
**NA20903**	male	SAME122921	GIH	Gujarati	SAS	South Asian Ancestry	GIH	1000 Genomes on GRCh38,1000 Genomes 30x on GRCh38,1000 Genomes phase 3 release
**NA19924**	female	SAME124714	ASW	African Ancestry SW	AFR	African Ancestry	ASW	1000 Genomes 30x on GRCh38,1000 Genomes phase 3 release
**NA20908**	female	SAME123777	GIH	Gujarati	SAS	South Asian Ancestry	GIH	1000 Genomes on GRCh38,1000 Genomes 30x on GRCh38,1000 Genomes phase 3 release
**NA20762**	male	SAME124744	TSI	Toscani	EUR	European Ancestry	TSI	1000 Genomes on GRCh38,1000 Genomes 30x on GRCh38,1000 Genomes phase 3 release
**NA20910**	female	SAME123100	GIH	Gujarati	SAS	South Asian Ancestry	GIH	1000 Genomes on GRCh38,1000 Genomes 30x on GRCh38,1000 Genomes phase 3 release
**NA20767**	male	SAME124369	TSI	Toscani	EUR	European Ancestry	TSI	1000 Genomes on GRCh38,1000 Genomes 30x on GRCh38,1000 Genomes phase 3 release
**NA21088**	female	SAME123143	GIH	Gujarati	SAS	South Asian Ancestry	GIH	1000 Genomes on GRCh38,1000 Genomes 30x on GRCh38,1000 Genomes phase 3 release
**NA20774**	female	SAME124572	TSI	Toscani	EUR	European Ancestry	TSI	1000 Genomes on GRCh38,1000 Genomes 30x on GRCh38,1000 Genomes phase 3 release,1000 Genomes phase 1 release,Geuvadis
**NA20852**	male	SAME123244	GIH	Gujarati	SAS	South Asian Ancestry	GIH	1000 Genomes on GRCh38,1000 Genomes 30x on GRCh38,1000 Genomes phase 3 release
**NA20786**	female	SAME123610	TSI	Toscani	EUR	European Ancestry	TSI	1000 Genomes on GRCh38,1000 Genomes 30x on GRCh38,1000 Genomes phase 3 release,1000 Genomes phase 1 release,Geuvadis
**NA20864**	male	SAME123944	GIH	Gujarati	SAS	South Asian Ancestry	GIH	1000 Genomes on GRCh38,1000 Genomes 30x on GRCh38,1000 Genomes phase 3 release
**NA20798**	male	SAME123821	TSI	Toscani	EUR	European Ancestry	TSI	1000 Genomes on GRCh38,1000 Genomes 30x on GRCh38,1000 Genomes phase 3 release,1000 Genomes phase 1 release,Geuvadis
**NA20869**	female	SAME123801	GIH	Gujarati	SAS	South Asian Ancestry	GIH	1000 Genomes on GRCh38,1000 Genomes 30x on GRCh38,1000 Genomes phase 3 release
**NA20801**	male	SAME125365	TSI	Toscani	EUR	European Ancestry	TSI	1000 Genomes on GRCh38,1000 Genomes 30x on GRCh38,1000 Genomes phase 3 release,1000 Genomes phase 1 release,Geuvadis
**NA20806**	male	SAME125362	TSI	Toscani	EUR	European Ancestry	TSI	1000 Genomes on GRCh38,1000 Genomes 30x on GRCh38,1000 Genomes phase 3 release,1000 Genomes phase 1 release,Geuvadis
**NA21108**	female	SAME124442	GIH	Gujarati	SAS	South Asian Ancestry	GIH	1000 Genomes on GRCh38,1000 Genomes 30x on GRCh38,1000 Genomes phase 3 release
**NA20813**	female	SAME125189	TSI	Toscani	EUR	European Ancestry	TSI	1000 Genomes on GRCh38,1000 Genomes 30x on GRCh38,1000 Genomes phase 3 release,1000 Genomes phase 1 release,Geuvadis
**NA21110**	female	SAME124180	GIH	Gujarati	SAS	South Asian Ancestry	GIH	1000 Genomes on GRCh38,1000 Genomes 30x on GRCh38,1000 Genomes phase 3 release
**NA20818**	female	SAME125184	TSI	Toscani	EUR	European Ancestry	TSI	1000 Genomes on GRCh38,1000 Genomes 30x on GRCh38,1000 Genomes phase 3 release,1000 Genomes phase 1 release
**NA21115**	male	SAME124183	GIH	Gujarati	SAS	South Asian Ancestry	GIH	1000 Genomes on GRCh38,1000 Genomes 30x on GRCh38,1000 Genomes phase 3 release
**NA20832**	female	SAME122894	TSI	Toscani	EUR	European Ancestry	TSI	1000 Genomes on GRCh38,1000 Genomes 30x on GRCh38,1000 Genomes phase 3 release
**NA21122**	female	SAME124030	GIH	Gujarati	SAS	South Asian Ancestry	GIH	1000 Genomes on GRCh38,1000 Genomes 30x on GRCh38,1000 Genomes phase 3 release
**NA20128**	female	SAME122887	ASW	African Ancestry SW	AFR	African Ancestry	ASW	1000 Genomes 30x on GRCh38,1000 Genomes phase 3 release
**NA21127**	male	SAME124027	GIH	Gujarati	SAS	South Asian Ancestry	GIH	1000 Genomes on GRCh38,1000 Genomes 30x on GRCh38,1000 Genomes phase 3 release
**NA20274**	female	SAME123615	ASW	African Ancestry SW	AFR	African Ancestry	ASW	1000 Genomes on GRCh38,1000 Genomes 30x on GRCh38,1000 Genomes phase 3 release
**NA20876**	female	SAME123599	GIH	Gujarati	SAS	South Asian Ancestry	GIH	1000 Genomes on GRCh38,1000 Genomes 30x on GRCh38,1000 Genomes phase 3 release
**NA20279**	male	SAME123616	ASW	African Ancestry SW	AFR	African Ancestry	ASW	1000 Genomes 30x on GRCh38,1000 Genomes phase 3 release
**NA20281**	male	SAME124584	ASW	African Ancestry SW	AFR	African Ancestry	ASW	1000 Genomes on GRCh38,1000 Genomes 30x on GRCh38,1000 Genomes phase 3 release,1000 Genomes phase 1 release
**NA20888**	female	SAME125258	GIH	Gujarati	SAS	South Asian Ancestry	GIH	1000 Genomes on GRCh38,1000 Genomes 30x on GRCh38,1000 Genomes phase 3 release
**NA20298**	male	SAME124382	ASW	African Ancestry SW	AFR	African Ancestry	ASW	1000 Genomes on GRCh38,1000 Genomes 30x on GRCh38,1000 Genomes phase 3 release,1000 Genomes phase 1 release
**NA20890**	male	SAME123671	GIH	Gujarati	SAS	South Asian Ancestry	GIH	1000 Genomes on GRCh38,1000 Genomes 30x on GRCh38,1000 Genomes phase 3 release
**NA21090**	male	SAME123320	GIH	Gujarati	SAS	South Asian Ancestry	GIH	1000 Genomes on GRCh38,1000 Genomes 30x on GRCh38,1000 Genomes phase 3 release
**NA21095**	male	SAME123318	GIH	Gujarati	SAS	South Asian Ancestry	GIH	1000 Genomes on GRCh38,1000 Genomes 30x on GRCh38,1000 Genomes phase 3 release
**NA20318**	male	SAME123802	ASW	African Ancestry SW	AFR	African Ancestry	ASW	1000 Genomes on GRCh38,1000 Genomes 30x on GRCh38,1000 Genomes phase 3 release
**NA21103**	female	SAME124439	GIH	Gujarati	SAS	South Asian Ancestry	GIH	1000 Genomes on GRCh38,1000 Genomes 30x on GRCh38,1000 Genomes phase 3 release
**NA20320**	female	SAME123997	ASW	African Ancestry SW	AFR	African Ancestry	ASW	1000 Genomes on GRCh38,1000 Genomes 30x on GRCh38,1000 Genomes phase 3 release
**NA20332**	female	SAME124198	ASW	African Ancestry SW	AFR	African Ancestry	ASW	1000 Genomes on GRCh38,1000 Genomes 30x on GRCh38,1000 Genomes phase 3 release,1000 Genomes phase 1 release
**NA20351**	male	SAME124596	ASW	African Ancestry SW	AFR	African Ancestry	ASW	1000 Genomes on GRCh38,1000 Genomes 30x on GRCh38,1000 Genomes phase 3 release,1000 Genomes phase 1 release
**NA20356**	male	SAME124598	ASW	African Ancestry SW	AFR	African Ancestry	ASW	1000 Genomes on GRCh38,1000 Genomes 30x on GRCh38,1000 Genomes phase 3 release,1000 Genomes phase 1 release
**NA19741**	male	SAME124718	MXL	Mexican Ancestry	AMR	American Ancestry	MXL	1000 Genomes on GRCh38,1000 Genomes 30x on GRCh38,1000 Genomes phase 3 release,1000 Genomes phase 1 release
**NA19746**	female	SAME123502	MXL	Mexican Ancestry	AMR	American Ancestry	MXL	1000 Genomes on GRCh38,1000 Genomes 30x on GRCh38,1000 Genomes phase 3 release,1000 Genomes phase 1 release
**NA19758**	female	SAME123706	MXL	Mexican Ancestry	AMR	American Ancestry	MXL	1000 Genomes on GRCh38,1000 Genomes 30x on GRCh38,1000 Genomes phase 3 release,1000 Genomes phase 1 release
**NA19760**	female	SAME123895	MXL	Mexican Ancestry	AMR	American Ancestry	MXL	1000 Genomes 30x on GRCh38,1000 Genomes phase 3 release
**NA19772**	male	SAME124066	MXL	Mexican Ancestry	AMR	American Ancestry	MXL	1000 Genomes 30x on GRCh38,1000 Genomes phase 3 release
**NA19777**	male	SAME124063	MXL	Mexican Ancestry	AMR	American Ancestry	MXL	1000 Genomes on GRCh38,1000 Genomes 30x on GRCh38,1000 Genomes phase 3 release,1000 Genomes phase 1 release
**NA20849**	female	SAME123433	GIH	Gujarati	SAS	South Asian Ancestry	GIH	1000 Genomes on GRCh38,1000 Genomes 30x on GRCh38,1000 Genomes phase 3 release
**NA20851**	female	SAME123245	GIH	Gujarati	SAS	South Asian Ancestry	GIH	1000 Genomes on GRCh38,1000 Genomes 30x on GRCh38,1000 Genomes phase 3 release
**NA20856**	female	SAME123247	GIH	Gujarati	SAS	South Asian Ancestry	GIH	1000 Genomes on GRCh38,1000 Genomes 30x on GRCh38,1000 Genomes phase 3 release
**NA20863**	male	SAME123805	GIH	Gujarati	SAS	South Asian Ancestry	GIH	1000 Genomes on GRCh38,1000 Genomes 30x on GRCh38,1000 Genomes phase 3 release
**NA20868**	female	SAME123800	GIH	Gujarati	SAS	South Asian Ancestry	GIH	1000 Genomes on GRCh38,1000 Genomes 30x on GRCh38,1000 Genomes phase 3 release
**NA20870**	male	SAME122839	GIH	Gujarati	SAS	South Asian Ancestry	GIH	1000 Genomes on GRCh38,1000 Genomes 30x on GRCh38,1000 Genomes phase 3 release
**NA20894**	female	SAME123667	GIH	Gujarati	SAS	South Asian Ancestry	GIH	1000 Genomes on GRCh38,1000 Genomes 30x on GRCh38,1000 Genomes phase 3 release
**NA20899**	female	SAME123140	GIH	Gujarati	SAS	South Asian Ancestry	GIH	1000 Genomes on GRCh38,1000 Genomes 30x on GRCh38,1000 Genomes phase 3 release
**NA20902**	female	SAME122922	GIH	Gujarati	SAS	South Asian Ancestry	GIH	1000 Genomes on GRCh38,1000 Genomes 30x on GRCh38,1000 Genomes phase 3 release
**NA21087**	male	SAME123138	GIH	Gujarati	SAS	South Asian Ancestry	GIH	1000 Genomes on GRCh38,1000 Genomes 30x on GRCh38,1000 Genomes phase 3 release
**NA20875**	female	SAME123596	GIH	Gujarati	SAS	South Asian Ancestry	GIH	1000 Genomes on GRCh38,1000 Genomes 30x on GRCh38,1000 Genomes phase 3 release
**NA20882**	female	SAME123882	GIH	Gujarati	SAS	South Asian Ancestry	GIH	1000 Genomes on GRCh38,1000 Genomes 30x on GRCh38,1000 Genomes phase 3 release
**NA20887**	male	SAME123879	GIH	Gujarati	SAS	South Asian Ancestry	GIH	1000 Genomes on GRCh38,1000 Genomes 30x on GRCh38,1000 Genomes phase 3 release
**NA21133**	male	SAME123831	GIH	Gujarati	SAS	South Asian Ancestry	GIH	1000 Genomes on GRCh38,1000 Genomes 30x on GRCh38,1000 Genomes phase 3 release
**NA21114**	male	SAME124184	GIH	Gujarati	SAS	South Asian Ancestry	GIH	1000 Genomes on GRCh38,1000 Genomes 30x on GRCh38,1000 Genomes phase 3 release
**NA21119**	male	SAME123584	GIH	Gujarati	SAS	South Asian Ancestry	GIH	1000 Genomes on GRCh38,1000 Genomes 30x on GRCh38,1000 Genomes phase 3 release
**NA21126**	male	SAME124026	GIH	Gujarati	SAS	South Asian Ancestry	GIH	1000 Genomes on GRCh38,1000 Genomes 30x on GRCh38,1000 Genomes phase 3 release
**NA21094**	male	SAME123317	GIH	Gujarati	SAS	South Asian Ancestry	GIH	1000 Genomes on GRCh38,1000 Genomes 30x on GRCh38,1000 Genomes phase 3 release
**NA21099**	male	SAME123325	GIH	Gujarati	SAS	South Asian Ancestry	GIH	1000 Genomes on GRCh38,1000 Genomes 30x on GRCh38,1000 Genomes phase 3 release
**NA21102**	female	SAME124438	GIH	Gujarati	SAS	South Asian Ancestry	GIH	1000 Genomes on GRCh38,1000 Genomes 30x on GRCh38,1000 Genomes phase 3 release
**NA21107**	male	SAME124435	GIH	Gujarati	SAS	South Asian Ancestry	GIH	1000 Genomes on GRCh38,1000 Genomes 30x on GRCh38,1000 Genomes phase 3 release
**HG00157**	male	SAME124590	GBR	British	EUR	European Ancestry	GBR	1000 Genomes on GRCh38,1000 Genomes 30x on GRCh38,1000 Genomes phase 3 release,1000 Genomes phase 1 release,Geuvadis
**HG00171**	female	SAME124961	FIN	Finnish	EUR	European Ancestry	FIN	1000 Genomes on GRCh38,1000 Genomes 30x on GRCh38,Human Genome Structural Variation Consortium, Phase 2,1000 Genomes phase 3 release,1000 Genomes pha
**HG00176**	female	SAME124956	FIN	Finnish	EUR	European Ancestry	FIN	1000 Genomes on GRCh38,1000 Genomes 30x on GRCh38,1000 Genomes phase 3 release,1000 Genomes phase 1 release,Geuvadis
**HG00183**	male	SAME123642	FIN	Finnish	EUR	European Ancestry	FIN	1000 Genomes on GRCh38,1000 Genomes 30x on GRCh38,1000 Genomes phase 3 release,1000 Genomes phase 1 release,Geuvadis
**HG00188**	male	SAME123641	FIN	Finnish	EUR	European Ancestry	FIN	1000 Genomes on GRCh38,1000 Genomes 30x on GRCh38,1000 Genomes phase 3 release,1000 Genomes phase 1 release,Geuvadis
**HG00190**	male	SAME123856	FIN	Finnish,Finnish	EUR	European Ancestry,West Eurasia (SGDP)	FIN,FinnishSGDP	1000 Genomes on GRCh38,Simons Genome Diversity Project,1000 Genomes 30x on GRCh38,1000 Genomes phase 3 release,1000 Genomes phase 1 release
**HG00234**	male	SAME124123	GBR	British	EUR	European Ancestry	GBR	1000 Genomes on GRCh38,1000 Genomes 30x on GRCh38,1000 Genomes phase 3 release,1000 Genomes phase 1 release,Geuvadis
**HG00239**	female	SAME124130	GBR	British	EUR	European Ancestry	GBR	1000 Genomes on GRCh38,1000 Genomes 30x on GRCh38,1000 Genomes phase 3 release,1000 Genomes phase 1 release,Geuvadis
**HG00246**	male	SAME123219	GBR	British	EUR	European Ancestry	GBR	1000 Genomes on GRCh38,1000 Genomes 30x on GRCh38,1000 Genomes phase 3 release,1000 Genomes phase 1 release,Geuvadis
**HG00253**	female	SAME123051	GBR	British	EUR	European Ancestry	GBR	1000 Genomes on GRCh38,1000 Genomes 30x on GRCh38,1000 Genomes phase 3 release,1000 Genomes phase 1 release,Geuvadis
**HG00258**	female	SAME123057	GBR	British	EUR	European Ancestry	GBR	1000 Genomes on GRCh38,1000 Genomes 30x on GRCh38,1000 Genomes phase 3 release,1000 Genomes phase 1 release,Geuvadis
**HG00260**	male	SAME123582	GBR	British	EUR	European Ancestry	GBR	1000 Genomes on GRCh38,1000 Genomes 30x on GRCh38,1000 Genomes phase 3 release,1000 Genomes phase 1 release,Geuvadis
**HG00265**	male	SAME123579	GBR	British	EUR	European Ancestry	GBR	1000 Genomes on GRCh38,1000 Genomes 30x on GRCh38,1000 Genomes phase 3 release,1000 Genomes phase 1 release,Geuvadis
**HG00272**	female	SAME123420	FIN	Finnish	EUR	European Ancestry	FIN	1000 Genomes on GRCh38,1000 Genomes 30x on GRCh38,1000 Genomes phase 3 release,1000 Genomes phase 1 release,Geuvadis
**HG00277**	male	SAME123423	FIN	Finnish	EUR	European Ancestry	FIN	1000 Genomes on GRCh38,1000 Genomes 30x on GRCh38,1000 Genomes phase 3 release,1000 Genomes phase 1 release,Geuvadis
**HG00284**	male	SAME122835	FIN	Finnish	EUR	European Ancestry	FIN	1000 Genomes on GRCh38,1000 Genomes 30x on GRCh38,1000 Genomes phase 3 release,1000 Genomes phase 1 release,Geuvadis
**HG00479**	female	SAME123334	CHS	Southern Han Chinese	EAS	East Asian Ancestry	CHS	1000 Genomes on GRCh38,1000 Genomes 30x on GRCh38,1000 Genomes phase 3 release,1000 Genomes phase 1 release,90 Han Chinese high coverage genomes
**HG00501**	female	SAME123362	CHS	Southern Han Chinese	EAS	East Asian Ancestry	CHS	1000 Genomes on GRCh38,1000 Genomes 30x on GRCh38,1000 Genomes phase 3 release,1000 Genomes phase 1 release
**HG00513**	female	SAME123526	CHS	Southern Han Chinese	EAS	East Asian Ancestry	CHS	1000 Genomes on GRCh38,1000 Genomes 30x on GRCh38,Human Genome Structural Variation Consortium, Phase 2,The Human Genome Structural Variation Consortiu
**HG00525**	female	SAME123242	CHS	Southern Han Chinese	EAS	East Asian Ancestry	CHS	1000 Genomes on GRCh38,1000 Genomes 30x on GRCh38,1000 Genomes phase 3 release,1000 Genomes phase 1 release,90 Han Chinese high coverage genomes
**HG00532**	male	SAME125268	CHS	Southern Han Chinese	EAS	East Asian Ancestry	CHS	1000 Genomes 30x on GRCh38,1000 Genomes phase 3 release
**HG00537**	female	SAME124345	CHS	Southern Han Chinese	EAS	East Asian Ancestry	CHS	1000 Genomes on GRCh38,1000 Genomes 30x on GRCh38,1000 Genomes phase 3 release,1000 Genomes phase 1 release,90 Han Chinese high coverage genomes
**HG00109**	male	SAME125160	GBR	British	EUR	European Ancestry	GBR	1000 Genomes on GRCh38,1000 Genomes 30x on GRCh38,1000 Genomes phase 3 release,1000 Genomes phase 1 release,Geuvadis
**HG00544**	female	SAME124086	CHS	Southern Han Chinese	EAS	East Asian Ancestry	CHS	1000 Genomes 30x on GRCh38,1000 Genomes phase 3 release
**HG00111**	female	SAME125340	GBR	British	EUR	European Ancestry	GBR	1000 Genomes on GRCh38,1000 Genomes 30x on GRCh38,1000 Genomes phase 3 release,1000 Genomes phase 1 release,Geuvadis
**HG00551**	female	SAME124252	PUR	Puerto Rican	AMR	American Ancestry	PUR	1000 Genomes on GRCh38,1000 Genomes 30x on GRCh38,1000 Genomes phase 3 release,1000 Genomes phase 1 release
**HG00116**	male	SAME125345	GBR	British	EUR	European Ancestry	GBR	1000 Genomes on GRCh38,1000 Genomes 30x on GRCh38,1000 Genomes phase 3 release,1000 Genomes phase 1 release,Geuvadis
**HG00556**	male	SAME123971	CHS	Southern Han Chinese	EAS	East Asian Ancestry	CHS	1000 Genomes on GRCh38,1000 Genomes 30x on GRCh38,1000 Genomes phase 3 release,1000 Genomes phase 1 release,90 Han Chinese high coverage genomes
**HG00123**	female	SAME122875	GBR	British	EUR	European Ancestry	GBR	1000 Genomes on GRCh38,1000 Genomes 30x on GRCh38,1000 Genomes phase 3 release,1000 Genomes phase 1 release,Geuvadis
**HG00582**	female	SAME124830	CHS	Southern Han Chinese	EAS	East Asian Ancestry	CHS	1000 Genomes 30x on GRCh38,1000 Genomes phase 3 release

**HG00128**	female	SAME122868	GBR	British,English	EUR	European Ancestry,West Eurasia (SGDP)	GBR,EnglishSGDP	1000 Genomes on GRCh38,Simons Genome Diversity Project,1000 Genomes 30x on GRCh38,1000 Genomes phase 3 release,1000 Genomes phase 1 release,Geuvadis
**HG00099**	female	SAME123271	GBR	British	EUR	European Ancestry	GBR	1000 Genomes on GRCh38,1000 Genomes 30x on GRCh38,1000 Genomes phase 3 release,1000 Genomes phase 1 release,Geuvadis
**HG00130**	female	SAME123063	GBR	British	EUR	European Ancestry	GBR	1000 Genomes on GRCh38,1000 Genomes 30x on GRCh38,1000 Genomes phase 3 release,1000 Genomes phase 1 release,Geuvadis
**HG00102**	female	SAME123945	GBR	British	EUR	European Ancestry	GBR	1000 Genomes on GRCh38,1000 Genomes 30x on GRCh38,1000 Genomes phase 3 release,1000 Genomes phase 1 release,Geuvadis
**HG00107**	male	SAME123947	GBR	British	EUR	European Ancestry	GBR	1000 Genomes on GRCh38,1000 Genomes 30x on GRCh38,1000 Genomes phase 3 release,1000 Genomes phase 1 release,Geuvadis
**HG00142**	male	SAME124818	GBR	British	EUR	European Ancestry	GBR	1000 Genomes on GRCh38,1000 Genomes 30x on GRCh38,1000 Genomes phase 3 release,1000 Genomes phase 1 release,Geuvadis
**HG00114**	male	SAME125343	GBR	British	EUR	European Ancestry	GBR	1000 Genomes on GRCh38,1000 Genomes 30x on GRCh38,1000 Genomes phase 3 release,1000 Genomes phase 1 release,Geuvadis
**HG00119**	male	SAME125348	GBR	British	EUR	European Ancestry	GBR	1000 Genomes on GRCh38,1000 Genomes 30x on GRCh38,1000 Genomes phase 3 release,1000 Genomes phase 1 release,Geuvadis
**HG00154**	female	SAME124587	GBR	British	EUR	European Ancestry	GBR	1000 Genomes on GRCh38,1000 Genomes 30x on GRCh38,1000 Genomes phase 3 release,1000 Genomes phase 1 release,Geuvadis
**HG00121**	female	SAME122873	GBR	British	EUR	European Ancestry	GBR	1000 Genomes on GRCh38,1000 Genomes 30x on GRCh38,1000 Genomes phase 3 release,1000 Genomes phase 1 release,Geuvadis
**HG00159**	male	SAME124586	GBR	British	EUR	European Ancestry	GBR	1000 Genomes on GRCh38,1000 Genomes 30x on GRCh38,1000 Genomes phase 3 release,1000 Genomes phase 1 release,Geuvadis
**HG00126**	male	SAME122872	GBR	British,English	EUR	European Ancestry,West Eurasia (SGDP)	GBR,EnglishSGDP	1000 Genomes on GRCh38,Simons Genome Diversity Project,1000 Genomes 30x on GRCh38,1000 Genomes phase 3 release,1000 Genomes phase 1 release,Geuvadis
**HG00173**	female	SAME124960	FIN	Finnish	EUR	European Ancestry	FIN	1000 Genomes on GRCh38,1000 Genomes 30x on GRCh38,1000 Genomes phase 3 release,1000 Genomes phase 1 release,Geuvadis
**HG00133**	female	SAME123062	GBR	British	EUR	European Ancestry	GBR	1000 Genomes on GRCh38,1000 Genomes 30x on GRCh38,1000 Genomes phase 3 release,1000 Genomes phase 1 release,Geuvadis
**HG00178**	female	SAME124964	FIN	Finnish	EUR	European Ancestry	FIN	1000 Genomes on GRCh38,1000 Genomes 30x on GRCh38,1000 Genomes phase 3 release,1000 Genomes phase 1 release,Geuvadis
**HG00138**	male	SAME123059	GBR	British	EUR	European Ancestry	GBR	1000 Genomes on GRCh38,1000 Genomes 30x on GRCh38,1000 Genomes phase 3 release,1000 Genomes phase 1 release,Geuvadis
**HG00180**	female	SAME123645	FIN	Finnish	EUR	European Ancestry	FIN	1000 Genomes on GRCh38,1000 Genomes 30x on GRCh38,1000 Genomes phase 3 release,1000 Genomes phase 1 release,Geuvadis
**HG00140**	male	SAME124396	GBR	British	EUR	European Ancestry	GBR	1000 Genomes on GRCh38,1000 Genomes 30x on GRCh38,1000 Genomes phase 3 release,1000 Genomes phase 1 release
**HG00185**	male	SAME123648	FIN	Finnish	EUR	European Ancestry	FIN	1000 Genomes on GRCh38,1000 Genomes 30x on GRCh38,1000 Genomes phase 3 release,1000 Genomes phase 1 release,Geuvadis
**HG00145**	male	SAME124391	GBR	British	EUR	European Ancestry	GBR	1000 Genomes on GRCh38,1000 Genomes 30x on GRCh38,1000 Genomes phase 3 release,1000 Genomes phase 1 release,Geuvadis
**HG00231**	female	SAME124126	GBR	British	EUR	European Ancestry	GBR	1000 Genomes on GRCh38,1000 Genomes 30x on GRCh38,1000 Genomes phase 3 release,1000 Genomes phase 1 release,Geuvadis
**HG00304**	female	SAME1840118	FIN	Finnish	EUR	European Ancestry	FIN	1000 Genomes on GRCh38,1000 Genomes 30x on GRCh38,1000 Genomes phase 3 release
**HG00236**	female	SAME123273	GBR	British	EUR	European Ancestry	GBR	1000 Genomes on GRCh38,1000 Genomes 30x on GRCh38,1000 Genomes phase 3 release,1000 Genomes phase 1 release,Geuvadis
**HG00309**	female	SAME124160	FIN	Finnish	EUR	European Ancestry	FIN	1000 Genomes on GRCh38,1000 Genomes 30x on GRCh38,1000 Genomes phase 3 release,1000 Genomes phase 1 release,Geuvadis
**HG00243**	male	SAME123224	GBR	British	EUR	European Ancestry	GBR	1000 Genomes on GRCh38,1000 Genomes 30x on GRCh38,1000 Genomes phase 3 release,1000 Genomes phase 1 release,Geuvadis
**HG00311**	male	SAME124339	FIN	Finnish	EUR	European Ancestry	FIN	1000 Genomes on GRCh38,1000 Genomes 30x on GRCh38,1000 Genomes phase 3 release,1000 Genomes phase 1 release,Geuvadis
**HG00318**	female	SAME124333	FIN	Finnish	EUR	European Ancestry	FIN	1000 Genomes on GRCh38,1000 Genomes 30x on GRCh38,1000 Genomes phase 3 release,1000 Genomes phase 1 release
**HG00323**	female	SAME123795	FIN	Finnish	EUR	European Ancestry	FIN	1000 Genomes on GRCh38,1000 Genomes 30x on GRCh38,1000 Genomes phase 3 release,1000 Genomes phase 1 release,Geuvadis
**HG00320**	female	SAME123797	FIN	Finnish	EUR	European Ancestry	FIN	1000 Genomes on GRCh38,1000 Genomes 30x on GRCh38,1000 Genomes phase 3 release,1000 Genomes phase 1 release,Geuvadis
**HG00328**	female	SAME123799	FIN	Finnish	EUR	European Ancestry	FIN	1000 Genomes on GRCh38,1000 Genomes 30x on GRCh38,1000 Genomes phase 3 release,1000 Genomes phase 1 release,Geuvadis
**HG00325**	male	SAME123793	FIN	Finnish	EUR	European Ancestry	FIN	1000 Genomes on GRCh38,1000 Genomes 30x on GRCh38,1000 Genomes phase 3 release,1000 Genomes phase 1 release,Geuvadis
**HG00330**	female	SAME123985	FIN	Finnish	EUR	European Ancestry	FIN	1000 Genomes on GRCh38,1000 Genomes 30x on GRCh38,1000 Genomes phase 3 release,1000 Genomes phase 1 release,Geuvadis
**HG00332**	female	SAME123987	FIN	Finnish	EUR	European Ancestry	FIN	1000 Genomes on GRCh38,1000 Genomes 30x on GRCh38,1000 Genomes phase 3 release,1000 Genomes phase 1 release,Geuvadis
**HG00335**	male	SAME123982	FIN	Finnish	EUR	European Ancestry	FIN	1000 Genomes on GRCh38,1000 Genomes 30x on GRCh38,1000 Genomes phase 3 release,1000 Genomes phase 1 release,Geuvadis
**HG00337**	female	SAME123984	FIN	Finnish	EUR	European Ancestry	FIN	1000 Genomes on GRCh38,1000 Genomes 30x on GRCh38,1000 Genomes phase 3 release,1000 Genomes phase 1 release,Geuvadis
**HG00342**	male	SAME124924	FIN	Finnish	EUR	European Ancestry	FIN	1000 Genomes on GRCh38,1000 Genomes 30x on GRCh38,1000 Genomes phase 3 release,1000 Genomes phase 1 release,Geuvadis
**HG00344**	female	SAME125255	FIN	Finnish	EUR	European Ancestry	FIN	1000 Genomes on GRCh38,1000 Genomes 30x on GRCh38,1000 Genomes phase 3 release,1000 Genomes phase 1 release,Geuvadis
**HG00349**	female	SAME124925	FIN	Finnish	EUR	European Ancestry	FIN	1000 Genomes on GRCh38,1000 Genomes 30x on GRCh38,1000 Genomes phase 3 release,1000 Genomes phase 1 release,Geuvadis
**HG00361**	female	SAME124538	FIN	Finnish	EUR	European Ancestry	FIN	1000 Genomes on GRCh38,1000 Genomes 30x on GRCh38,1000 Genomes phase 3 release,1000 Genomes phase 1 release,Geuvadis
**HG00351**	male	SAME125122	FIN	Finnish	EUR	European Ancestry	FIN	1000 Genomes on GRCh38,1000 Genomes 30x on GRCh38,1000 Genomes phase 3 release,1000 Genomes phase 1 release,Geuvadis
**HG00366**	male	SAME124541	FIN	Finnish	EUR	European Ancestry	FIN	1000 Genomes on GRCh38,1000 Genomes 30x on GRCh38,1000 Genomes phase 3 release,1000 Genomes phase 1 release,Geuvadis
**HG00356**	female	SAME125123	FIN	Finnish	EUR	European Ancestry	FIN	1000 Genomes on GRCh38,1000 Genomes 30x on GRCh38,1000 Genomes phase 3 release,1000 Genomes phase 1 release,Geuvadis
**HG00373**	female	SAME124749	FIN	Finnish	EUR	European Ancestry	FIN	1000 Genomes on GRCh38,1000 Genomes 30x on GRCh38,1000 Genomes phase 3 release,1000 Genomes phase 1 release,Geuvadis
**HG00368**	female	SAME124535	FIN	Finnish	EUR	European Ancestry	FIN	1000 Genomes on GRCh38,1000 Genomes 30x on GRCh38,1000 Genomes phase 3 release,1000 Genomes phase 1 release
**HG00378**	female	SAME124745	FIN	Finnish	EUR	European Ancestry	FIN	1000 Genomes on GRCh38,1000 Genomes 30x on GRCh38,1000 Genomes phase 3 release,1000 Genomes phase 1 release,Geuvadis
**HG00375**	male	SAME124750	FIN	Finnish	EUR	European Ancestry	FIN	1000 Genomes on GRCh38,1000 Genomes 30x on GRCh38,1000 Genomes phase 3 release,1000 Genomes phase 1 release,Geuvadis
**HG00380**	female	SAME123031	FIN	Finnish	EUR	European Ancestry	FIN	1000 Genomes on GRCh38,1000 Genomes 30x on GRCh38,1000 Genomes phase 3 release,1000 Genomes phase 1 release,Geuvadis
**HG00382**	male	SAME123033	FIN	Finnish	EUR	European Ancestry	FIN	1000 Genomes on GRCh38,1000 Genomes 30x on GRCh38,1000 Genomes phase 3 release,1000 Genomes phase 1 release,Geuvadis
**HG00594**	male	SAME125005	CHS	Southern Han Chinese	EAS	East Asian Ancestry	CHS	1000 Genomes 30x on GRCh38,1000 Genomes phase 3 release
**HG00407**	female	SAME125106	CHS	Southern Han Chinese	EAS	East Asian Ancestry	CHS	1000 Genomes on GRCh38,1000 Genomes 30x on GRCh38,1000 Genomes phase 3 release,1000 Genomes phase 1 release
**HG00599**	female	SAME1839802	CHS	Southern Han Chinese	EAS	East Asian Ancestry	CHS	1000 Genomes on GRCh38,1000 Genomes 30x on GRCh38,1000 Genomes phase 3 release
**HG00419**	female	SAME122991	CHS	Southern Han Chinese	EAS	East Asian Ancestry	CHS	1000 Genomes on GRCh38,1000 Genomes 30x on GRCh38,1000 Genomes phase 3 release,1000 Genomes phase 1 release
**HG00607**	male	SAME125373	CHS	Southern Han Chinese	EAS	East Asian Ancestry	CHS	1000 Genomes on GRCh38,1000 Genomes 30x on GRCh38,1000 Genomes phase 3 release,1000 Genomes phase 1 release
**HG00421**	male	SAME122808	CHS	Southern Han Chinese	EAS	East Asian Ancestry	CHS	1000 Genomes on GRCh38,1000 Genomes 30x on GRCh38,1000 Genomes phase 3 release,1000 Genomes phase 1 release,90 Han Chinese high coverage genomes
**HG00614**	female	SAME125195	CHS	Southern Han Chinese	EAS	East Asian Ancestry	CHS	1000 Genomes on GRCh38,1000 Genomes 30x on GRCh38,1000 Genomes phase 3 release,1000 Genomes phase 1 release
**HG00553**	male	SAME124254	PUR	Puerto Rican	AMR	American Ancestry	PUR	1000 Genomes on GRCh38,1000 Genomes 30x on GRCh38,1000 Genomes phase 3 release,1000 Genomes phase 1 release
**HG00619**	male	SAME125193	CHS	Southern Han Chinese	EAS	East Asian Ancestry	CHS	1000 Genomes on GRCh38,1000 Genomes 30x on GRCh38,1000 Genomes phase 3 release,1000 Genomes phase 1 release,90 Han Chinese high coverage genomes
**HG00558**	female	SAME124247	CHS	Southern Han Chinese	EAS	East Asian Ancestry	CHS	1000 Genomes 30x on GRCh38,1000 Genomes phase 3 release
**HG00621**	male	SAME123082	CHS	Southern Han Chinese	EAS	East Asian Ancestry	CHS	1000 Genomes 30x on GRCh38,1000 Genomes phase 3 release
**HG00560**	female	SAME124456	CHS	Southern Han Chinese	EAS	East Asian Ancestry	CHS	1000 Genomes on GRCh38,1000 Genomes 30x on GRCh38,1000 Genomes phase 3 release,1000 Genomes phase 1 release
**HG00626**	female	SAME123078	CHS	Southern Han Chinese	EAS	East Asian Ancestry	CHS	1000 Genomes on GRCh38,1000 Genomes 30x on GRCh38,1000 Genomes phase 3 release,1000 Genomes phase 1 release
**HG00565**	male	SAME124452	CHS	Southern Han Chinese	EAS	East Asian Ancestry	CHS	1000 Genomes on GRCh38,1000 Genomes 30x on GRCh38,1000 Genomes phase 3 release,1000 Genomes phase 1 release
**HG00577**	male	SAME123516	CHS	Southern Han Chinese	EAS	East Asian Ancestry	CHS	1000 Genomes on GRCh38,1000 Genomes 30x on GRCh38,1000 Genomes phase 3 release,1000 Genomes phase 1 release
**HG00405**	female	SAME123159	CHS	Southern Han Chinese	EAS	East Asian Ancestry	CHS	1000 Genomes 30x on GRCh38,1000 Genomes phase 3 release
**HG00584**	female	SAME124663	CHS	Southern Han Chinese	EAS	East Asian Ancestry	CHS	1000 Genomes on GRCh38,1000 Genomes 30x on GRCh38,1000 Genomes phase 3 release,1000 Genomes phase 1 release,90 Han Chinese high coverage genomes
**HG00429**	male	SAME122812	CHS	Southern Han Chinese	EAS	East Asian Ancestry	CHS	1000 Genomes 30x on GRCh38,1000 Genomes phase 3 release
**HG00589**	male	SAME124824	CHS	Southern Han Chinese	EAS	East Asian Ancestry	CHS	1000 Genomes on GRCh38,1000 Genomes 30x on GRCh38,1000 Genomes phase 3 release,1000 Genomes phase 1 release
**HG00436**	male	SAME125262	CHS	Southern Han Chinese	EAS	East Asian Ancestry	CHS	1000 Genomes on GRCh38,1000 Genomes 30x on GRCh38,1000 Genomes phase 3 release,1000 Genomes phase 1 release,90 Han Chinese high coverage genomes
**HG00591**	male	SAME125010	CHS	Southern Han Chinese	EAS	East Asian Ancestry	CHS	1000 Genomes 30x on GRCh38,1000 Genomes phase 3 release
**HG00443**	female	SAME124920	CHS	Southern Han Chinese	EAS	East Asian Ancestry	CHS	1000 Genomes on GRCh38,1000 Genomes 30x on GRCh38,1000 Genomes phase 3 release,1000 Genomes phase 1 release,90 Han Chinese high coverage genomes
**HG00596**	female	SAME125007	CHS	Southern Han Chinese	EAS	East Asian Ancestry	CHS	1000 Genomes on GRCh38,1000 Genomes 30x on GRCh38,1000 Genomes phase 3 release,1000 Genomes phase 1 release
**HG00448**	male	SAME124200	CHS	Southern Han Chinese	EAS	East Asian Ancestry	CHS	1000 Genomes on GRCh38,1000 Genomes 30x on GRCh38,1000 Genomes phase 3 release,1000 Genomes phase 1 release,90 Han Chinese high coverage genomes
**HG00609**	male	SAME125370	CHS	Southern Han Chinese	EAS	East Asian Ancestry	CHS	1000 Genomes 30x on GRCh38,1000 Genomes phase 3 release
**HG00450**	female	SAME125167	CHS	Southern Han Chinese	EAS	East Asian Ancestry	CHS	1000 Genomes 30x on GRCh38,1000 Genomes phase 3 release
**HG00611**	female	SAME125198	CHS	Southern Han Chinese	EAS	East Asian Ancestry	CHS	1000 Genomes on GRCh38,1000 Genomes 30x on GRCh38,1000 Genomes phase 3 release,1000 Genomes phase 1 release
**HG00474**	male	SAME123343	CHS	Southern Han Chinese	EAS	East Asian Ancestry	CHS	1000 Genomes 30x on GRCh38,1000 Genomes phase 3 release
**HG00638**	female	SAME123652	PUR	Puerto Rican	AMR	American Ancestry	PUR	1000 Genomes on GRCh38,1000 Genomes 30x on GRCh38,1000 Genomes phase 3 release,1000 Genomes phase 1 release
**HG00250**	female	SAME123054	GBR	British	EUR	European Ancestry	GBR	1000 Genomes on GRCh38,1000 Genomes 30x on GRCh38,1000 Genomes phase 3 release,1000 Genomes phase 1 release,Geuvadis
**HG00640**	male	SAME123434	PUR	Puerto Rican	AMR	American Ancestry	PUR	1000 Genomes on GRCh38,1000 Genomes 30x on GRCh38,1000 Genomes phase 3 release,1000 Genomes phase 1 release
**HG00255**	female	SAME123049	GBR	British	EUR	European Ancestry	GBR	1000 Genomes on GRCh38,1000 Genomes 30x on GRCh38,1000 Genomes phase 3 release,1000 Genomes phase 1 release,Geuvadis
**HG00652**	female	SAME123252	CHS	Southern Han Chinese	EAS	East Asian Ancestry	CHS	1000 Genomes 30x on GRCh38,1000 Genomes phase 3 release
**HG00262**	female	SAME123580	GBR	British	EUR	European Ancestry	GBR	1000 Genomes on GRCh38,1000 Genomes 30x on GRCh38,1000 Genomes phase 3 release,1000 Genomes phase 1 release,Geuvadis
**HG00657**	female	SAME123255	CHS	Southern Han Chinese	EAS	East Asian Ancestry	CHS	1000 Genomes on GRCh38,1000 Genomes 30x on GRCh38,1000 Genomes phase 3 release,1000 Genomes phase 1 release,90 Han Chinese high coverage genomes
**HG00267**	male	SAME123577	FIN	Finnish	EUR	European Ancestry	FIN	1000 Genomes on GRCh38,1000 Genomes 30x on GRCh38,1000 Genomes phase 3 release,1000 Genomes phase 1 release,Geuvadis
**HG00664**	male	SAME123814	CHS	Southern Han Chinese	EAS	East Asian Ancestry	CHS	1000 Genomes 30x on GRCh38,1000 Genomes phase 3 release
**HG00274**	female	SAME123422	FIN	Finnish	EUR	European Ancestry	FIN	1000 Genomes on GRCh38,1000 Genomes 30x on GRCh38,1000 Genomes phase 3 release,1000 Genomes phase 1 release,Geuvadis
**HG00671**	male	SAME123604	CHS	Southern Han Chinese	EAS	East Asian Ancestry	CHS	1000 Genomes on GRCh38,1000 Genomes 30x on GRCh38,1000 Genomes phase 3 release,1000 Genomes phase 1 release,90 Han Chinese high coverage genomes
**HG00281**	female	SAME122832	FIN	Finnish	EUR	European Ancestry	FIN	1000 Genomes on GRCh38,1000 Genomes 30x on GRCh38,1000 Genomes phase 3 release,1000 Genomes phase 1 release,Geuvadis
**HG00306**	female	SAME124159	FIN	Finnish	EUR	European Ancestry	FIN	1000 Genomes on GRCh38,1000 Genomes 30x on GRCh38,1000 Genomes phase 3 release,1000 Genomes phase 1 release,Geuvadis
**HG00683**	male	SAME123892	CHS	Southern Han Chinese	EAS	East Asian Ancestry	CHS	1000 Genomes on GRCh38,1000 Genomes 30x on GRCh38,1000 Genomes phase 3 release,1000 Genomes phase 1 release,90 Han Chinese high coverage genomes
**HG00313**	female	SAME124337	FIN	Finnish	EUR	European Ancestry	FIN	1000 Genomes on GRCh38,1000 Genomes 30x on GRCh38,1000 Genomes phase 3 release,1000 Genomes phase 1 release,Geuvadis
**HG00690**	female	SAME123686	CHS	Southern Han Chinese	EAS	East Asian Ancestry	CHS	1000 Genomes on GRCh38,1000 Genomes 30x on GRCh38,1000 Genomes phase 3 release,1000 Genomes phase 1 release,90 Han Chinese high coverage genomes
**HG00438**	female	SAME125267	CHS	Southern Han Chinese	EAS	East Asian Ancestry	CHS	1000 Genomes 30x on GRCh38,1000 Genomes phase 3 release
**HG01326**	female	SAME1839598	PUR	Puerto Rican	AMR	American Ancestry	PUR	1000 Genomes on GRCh38,1000 Genomes 30x on GRCh38,1000 Genomes phase 3 release
**HG00445**	male	SAME124975	CHS	Southern Han Chinese	EAS	East Asian Ancestry	CHS	1000 Genomes on GRCh38,1000 Genomes 30x on GRCh38,1000 Genomes phase 3 release,1000 Genomes phase 1 release
**HG01345**	female	SAME124863	CLM	Colombian	AMR	American Ancestry	CLM	1000 Genomes on GRCh38,1000 Genomes 30x on GRCh38,1000 Genomes phase 3 release,1000 Genomes phase 1 release
**HG00452**	female	SAME123115	CHS	Southern Han Chinese	EAS	East Asian Ancestry	CHS	1000 Genomes on GRCh38,1000 Genomes 30x on GRCh38,1000 Genomes phase 3 release,1000 Genomes phase 1 release
**HG01352**	female	SAME124686	CLM	Colombian	AMR	American Ancestry	CLM	1000 Genomes 30x on GRCh38,Human Genome Structural Variation Consortium, Phase 2,1000 Genomes phase 3 release
**HG00457**	male	SAME123095	CHS	Southern Han Chinese	EAS	East Asian Ancestry	CHS	1000 Genomes on GRCh38,1000 Genomes 30x on GRCh38,1000 Genomes phase 3 release,1000 Genomes phase 1 release
**HG01357**	female	SAME124681	CLM	Colombian	AMR	American Ancestry	CLM	1000 Genomes on GRCh38,1000 Genomes 30x on GRCh38,1000 Genomes phase 3 release,1000 Genomes phase 1 release
**HG00464**	female	SAME123497	CHS	Southern Han Chinese	EAS	East Asian Ancestry	CHS	1000 Genomes on GRCh38,1000 Genomes 30x on GRCh38,1000 Genomes phase 3 release,1000 Genomes phase 1 release
**HG00476**	female	SAME123341	CHS	Southern Han Chinese	EAS	East Asian Ancestry	CHS	1000 Genomes on GRCh38,1000 Genomes 30x on GRCh38,1000 Genomes phase 3 release,1000 Genomes phase 1 release,90 Han Chinese high coverage genomes
**HG01369**	female	SAME1839124	CLM	Colombian	AMR	American Ancestry	CLM	1000 Genomes on GRCh38,1000 Genomes 30x on GRCh38,1000 Genomes phase 3 release
**HG00534**	female	SAME123915	CHS	Southern Han Chinese	EAS	East Asian Ancestry	CHS	1000 Genomes on GRCh38,1000 Genomes 30x on GRCh38,1000 Genomes phase 3 release,1000 Genomes phase 1 release,90 Han Chinese high coverage genomes
**HG01376**	female	SAME124289	CLM	Colombian	AMR	American Ancestry	CLM	1000 Genomes 30x on GRCh38,1000 Genomes phase 3 release
**HG00096**	male	SAME123268	GBR	British	EUR	European Ancestry	GBR	1000 Genomes on GRCh38,1000 Genomes 30x on GRCh38,Human Genome Structural Variation Consortium, Phase 2,1000 Genomes phase 3 release,1000 Genomes ph
**HG01112**	male	SAME123485	CLM	Colombian	AMR	American Ancestry	CLM	1000 Genomes on GRCh38,1000 Genomes 30x on GRCh38,1000 Genomes phase 3 release,1000 Genomes phase 1 release
**HG00729**	female	SAME1840273	CHS	Southern Han Chinese	EAS	East Asian Ancestry	CHS	1000 Genomes on GRCh38,1000 Genomes 30x on GRCh38,1000 Genomes phase 3 release
**HG01124**	male	SAME124053	CLM	Colombian	AMR	American Ancestry	CLM	1000 Genomes on GRCh38,1000 Genomes 30x on GRCh38,1000 Genomes phase 3 release,1000 Genomes phase 1 release
**HG00731**	male	SAME125390	PUR	Puerto Rican	AMR	American Ancestry	PUR	1000 Genomes on GRCh38,1000 Genomes 30x on GRCh38,Human Genome Structural Variation Consortium, Phase 2,The Human Genome Structural Variation Consortiu
**HG01131**	female	SAME1839985	CLM	Colombian	AMR	American Ancestry	CLM	1000 Genomes on GRCh38,1000 Genomes 30x on GRCh38,1000 Genomes phase 3 release

**HG00736**	male	SAME123058	PUR	Puerto Rican	AMR	American Ancestry	PUR	1000 Genomes on GRCh38,1000 Genomes 30x on GRCh38,1000 Genomes phase 3 release,1000 Genomes phase 1 release
**HG01136**	male	SAME123889	CLM	Colombian	AMR	American Ancestry	CLM	1000 Genomes on GRCh38,1000 Genomes 30x on GRCh38,1000 Genomes phase 3 release,1000 Genomes phase 1 release
**HG00743**	female	SAME1840184	PUR	Puerto Rican	AMR	American Ancestry	PUR	1000 Genomes on GRCh38,1000 Genomes 30x on GRCh38,1000 Genomes phase 3 release
**HG01148**	male	SAME124424	CLM	Colombian	AMR	American Ancestry	CLM	1000 Genomes on GRCh38,1000 Genomes 30x on GRCh38,1000 Genomes phase 3 release,1000 Genomes phase 1 release
**HG00844**	male	SAME123952	CDX	Dai Chinese	EAS	East Asian Ancestry	CDX	1000 Genomes on GRCh38,1000 Genomes 30x on GRCh38,1000 Genomes phase 3 release
**HG01150**	female	SAME124216	CLM	Colombian	AMR	American Ancestry	CLM	1000 Genomes 30x on GRCh38,1000 Genomes phase 3 release
**HG00851**	female	SAME123756	CDX	Dai Chinese	EAS	East Asian Ancestry	CDX	1000 Genomes on GRCh38,1000 Genomes 30x on GRCh38,1000 Genomes phase 3 release
**HG01162**	female	SAME1839707	PUR	Puerto Rican	AMR	American Ancestry	PUR	1000 Genomes on GRCh38,1000 Genomes 30x on GRCh38,1000 Genomes phase 3 release
**HG01167**	male	SAME124798	PUR	Puerto Rican	AMR	American Ancestry	PUR	1000 Genomes on GRCh38,1000 Genomes 30x on GRCh38,1000 Genomes phase 3 release,1000 Genomes phase 1 release
**HG01082**	male	SAME125398	PUR	Puerto Rican	AMR	American Ancestry	PUR	1000 Genomes on GRCh38,1000 Genomes 30x on GRCh38,1000 Genomes phase 3 release,1000 Genomes phase 1 release
**HG01066**	male	SAME124294	PUR	Puerto Rican	AMR	American Ancestry	PUR	1000 Genomes on GRCh38,1000 Genomes 30x on GRCh38,1000 Genomes phase 3 release,1000 Genomes phase 1 release
**HG01087**	female	SAME125393	PUR	Puerto Rican	AMR	American Ancestry	PUR	1000 Genomes 30x on GRCh38,1000 Genomes phase 3 release
**HG01073**	female	SAME124095	PUR	Puerto Rican	AMR	American Ancestry	PUR	1000 Genomes on GRCh38,1000 Genomes 30x on GRCh38,1000 Genomes phase 3 release,1000 Genomes phase 1 release
**HG01094**	male	SAME122925	PUR	Puerto Rican	AMR	American Ancestry	PUR	1000 Genomes on GRCh38,1000 Genomes 30x on GRCh38,1000 Genomes phase 3 release,1000 Genomes phase 1 release
**HG01080**	female	SAME125333	PUR	Puerto Rican	AMR	American Ancestry	PUR	1000 Genomes on GRCh38,1000 Genomes 30x on GRCh38,1000 Genomes phase 3 release,1000 Genomes phase 1 release
**HG01099**	male	SAME123771	PUR	Puerto Rican	AMR	American Ancestry	PUR	1000 Genomes 30x on GRCh38,1000 Genomes phase 3 release
**HG01085**	male	SAME125391	PUR	Puerto Rican	AMR	American Ancestry	PUR	1000 Genomes on GRCh38,1000 Genomes 30x on GRCh38,1000 Genomes phase 3 release,1000 Genomes phase 1 release
**HG01102**	female	SAME123678	PUR	Puerto Rican	AMR	American Ancestry	PUR	1000 Genomes on GRCh38,1000 Genomes 30x on GRCh38,1000 Genomes phase 3 release,1000 Genomes phase 1 release
**HG01092**	female	SAME1839489	PUR	Puerto Rican	AMR	American Ancestry	PUR	1000 Genomes on GRCh38,1000 Genomes 30x on GRCh38,1000 Genomes phase 3 release
**HG01107**	male	SAME123675	PUR	Puerto Rican	AMR	American Ancestry	PUR	1000 Genomes on GRCh38,1000 Genomes 30x on GRCh38,1000 Genomes phase 3 release,1000 Genomes phase 1 release
**HG01097**	male	SAME122924	PUR	Puerto Rican	AMR	American Ancestry	PUR	1000 Genomes on GRCh38,1000 Genomes 30x on GRCh38,1000 Genomes phase 3 release,1000 Genomes phase 1 release
**HG01114**	female	SAME123483	CLM	Colombian	AMR	American Ancestry	CLM	1000 Genomes 30x on GRCh38,Human Genome Structural Variation Consortium, Phase 2,1000 Genomes phase 3 release
**HG01100**	female	SAME123676	PUR	Puerto Rican	AMR	American Ancestry	PUR	1000 Genomes 30x on GRCh38,1000 Genomes phase 3 release
**HG01119**	female	SAME1840402	CLM	Colombian	AMR	American Ancestry	CLM	1000 Genomes on GRCh38,1000 Genomes 30x on GRCh38,1000 Genomes phase 3 release
**HG01105**	female	SAME123673	PUR	Puerto Rican	AMR	American Ancestry	PUR	1000 Genomes on GRCh38,1000 Genomes 30x on GRCh38,1000 Genomes phase 3 release,1000 Genomes phase 1 release
**HG01121**	male	SAME1840127	CLM	Colombian	AMR	American Ancestry	CLM	1000 Genomes on GRCh38,1000 Genomes 30x on GRCh38,1000 Genomes phase 3 release
**HG01174**	female	SAME124619	PUR	Puerto Rican	AMR	American Ancestry	PUR	1000 Genomes on GRCh38,1000 Genomes 30x on GRCh38,1000 Genomes phase 3 release,1000 Genomes phase 1 release
**HG01126**	male	SAME124052	CLM	Colombian	AMR	American Ancestry	CLM	1000 Genomes 30x on GRCh38,1000 Genomes phase 3 release
**HG01198**	female	SAME125228	PUR	Puerto Rican	AMR	American Ancestry	PUR	1000 Genomes on GRCh38,1000 Genomes 30x on GRCh38,1000 Genomes phase 3 release,1000 Genomes phase 1 release
**HG01133**	male	SAME123884	CLM	Colombian	AMR	American Ancestry	CLM	1000 Genomes on GRCh38,1000 Genomes 30x on GRCh38,1000 Genomes phase 3 release,1000 Genomes phase 1 release
**HG01206**	male	SAME124295	PUR	Puerto Rican	AMR	American Ancestry	PUR	1000 Genomes 30x on GRCh38,1000 Genomes phase 3 release
**HG01138**	male	SAME123877	CLM	Colombian	AMR	American Ancestry	CLM	1000 Genomes 30x on GRCh38,1000 Genomes phase 3 release
**HG01249**	female	SAME125057	PUR	Puerto Rican	AMR	American Ancestry	PUR	1000 Genomes 30x on GRCh38,1000 Genomes phase 3 release
**HG01140**	female	SAME124422	CLM	Colombian	AMR	American Ancestry	CLM	1000 Genomes on GRCh38,1000 Genomes 30x on GRCh38,1000 Genomes phase 3 release,1000 Genomes phase 1 release
**HG01251**	female	SAME125282	CLM	Colombian	AMR	American Ancestry	CLM	1000 Genomes on GRCh38,1000 Genomes 30x on GRCh38,1000 Genomes phase 3 release,1000 Genomes phase 1 release
**HG01164**	male	SAME1839690	PUR	Puerto Rican	AMR	American Ancestry	PUR	1000 Genomes on GRCh38,1000 Genomes 30x on GRCh38,1000 Genomes phase 3 release
**HG01256**	male	SAME125287	CLM	Colombian	AMR	American Ancestry	CLM	1000 Genomes on GRCh38,1000 Genomes 30x on GRCh38,1000 Genomes phase 3 release,1000 Genomes phase 1 release
**HG01169**	female	SAME124797	PUR	Puerto Rican	AMR	American Ancestry	PUR	1000 Genomes 30x on GRCh38,1000 Genomes phase 3 release
**HG00703**	male	SAME122933	CHS	Southern Han Chinese	EAS	East Asian Ancestry	CHS	1000 Genomes 30x on GRCh38,1000 Genomes phase 3 release
**HG01171**	female	SAME124622	PUR	Puerto Rican	AMR	American Ancestry	PUR	1000 Genomes on GRCh38,1000 Genomes 30x on GRCh38,1000 Genomes phase 3 release,1000 Genomes phase 1 release
**HG00708**	female	SAME122940	CHS	Southern Han Chinese	EAS	East Asian Ancestry	CHS	1000 Genomes on GRCh38,1000 Genomes 30x on GRCh38,1000 Genomes phase 3 release,1000 Genomes phase 1 release
**HG01176**	male	SAME124621	PUR	Puerto Rican	AMR	American Ancestry	PUR	1000 Genomes on GRCh38,1000 Genomes 30x on GRCh38,1000 Genomes phase 3 release,1000 Genomes phase 1 release
**HG00734**	female	SAME123016	PUR	Puerto Rican	AMR	American Ancestry	PUR	1000 Genomes on GRCh38,1000 Genomes 30x on GRCh38,1000 Genomes phase 3 release,1000 Genomes phase 1 release
**HG01183**	female	SAME124792	PUR	Puerto Rican	AMR	American Ancestry	PUR	1000 Genomes on GRCh38,1000 Genomes 30x on GRCh38,1000 Genomes phase 3 release,1000 Genomes phase 1 release
**HG00739**	male	SAME125113	PUR	Puerto Rican	AMR	American Ancestry	PUR	1000 Genomes on GRCh38,1000 Genomes 30x on GRCh38,1000 Genomes phase 3 release,1000 Genomes phase 1 release
**HG01188**	female	SAME124927	PUR	Puerto Rican	AMR	American Ancestry	PUR	1000 Genomes on GRCh38,1000 Genomes 30x on GRCh38,1000 Genomes phase 3 release,1000 Genomes phase 1 release
**HG00741**	female	SAME124833	PUR	Puerto Rican	AMR	American Ancestry	PUR	1000 Genomes 30x on GRCh38,1000 Genomes phase 3 release
**HG01190**	male	SAME123654	PUR	Puerto Rican	AMR	American Ancestry	PUR	1000 Genomes on GRCh38,1000 Genomes 30x on GRCh38,1000 Genomes phase 3 release,1000 Genomes phase 1 release
**HG00623**	female	SAME1839285	CHS	Southern Han Chinese	EAS	East Asian Ancestry	CHS	1000 Genomes on GRCh38,1000 Genomes 30x on GRCh38,1000 Genomes phase 3 release
**HG01028**	male	SAME123524	CDX	Dai Chinese	EAS	East Asian Ancestry	CDX	1000 Genomes on GRCh38,1000 Genomes 30x on GRCh38,1000 Genomes phase 3 release
**HG00628**	male	SAME123085	CHS	Southern Han Chinese	EAS	East Asian Ancestry	CHS	1000 Genomes on GRCh38,1000 Genomes 30x on GRCh38,1000 Genomes phase 3 release,1000 Genomes phase 1 release
**HG01047**	male	SAME123166	PUR	Puerto Rican	AMR	American Ancestry	PUR	1000 Genomes on GRCh38,1000 Genomes 30x on GRCh38,1000 Genomes phase 3 release,1000 Genomes phase 1 release
**HG00630**	male	SAME122901	CHS	Southern Han Chinese	EAS	East Asian Ancestry	CHS	1000 Genomes 30x on GRCh38,1000 Genomes phase 3 release
**HG01054**	male	SAME123663	PUR	Puerto Rican	AMR	American Ancestry	PUR	1000 Genomes on GRCh38,1000 Genomes 30x on GRCh38,1000 Genomes phase 3 release,1000 Genomes phase 1 release
**HG00635**	female	SAME122902	CHS	Southern Han Chinese	EAS	East Asian Ancestry	CHS	1000 Genomes on GRCh38,1000 Genomes 30x on GRCh38,1000 Genomes phase 3 release,1000 Genomes phase 1 release
**HG01061**	female	SAME122821	PUR	Puerto Rican	AMR	American Ancestry	PUR	1000 Genomes on GRCh38,1000 Genomes 30x on GRCh38,1000 Genomes phase 3 release,1000 Genomes phase 1 release
**HG00642**	male	SAME123436	PUR	Puerto Rican	AMR	American Ancestry	PUR	1000 Genomes 30x on GRCh38,1000 Genomes phase 3 release
**HG01383**	male	SAME122971	CLM	Colombian	AMR	American Ancestry	CLM	1000 Genomes on GRCh38,1000 Genomes 30x on GRCh38,1000 Genomes phase 3 release,1000 Genomes phase 1 release
**HG00654**	female	SAME123234	CHS	Southern Han Chinese	EAS	East Asian Ancestry	CHS	1000 Genomes on GRCh38,1000 Genomes 30x on GRCh38,1000 Genomes phase 3 release,1000 Genomes phase 1 release
**HG01390**	female	SAME122805	CLM	Colombian	AMR	American Ancestry	CLM	1000 Genomes on GRCh38,1000 Genomes 30x on GRCh38,1000 Genomes phase 3 release,1000 Genomes phase 1 release
**HG01395**	male	SAME1840207	PUR	Puerto Rican	AMR	American Ancestry	PUR	1000 Genomes on GRCh38,1000 Genomes 30x on GRCh38,1000 Genomes phase 3 release
**HG01241**	male	SAME125065	PUR	Puerto Rican	AMR	American Ancestry	PUR	1000 Genomes on GRCh38,1000 Genomes 30x on GRCh38,1000 Genomes phase 3 release,1000 Genomes phase 1 release
**HG01403**	female	SAME1839867	PUR	Puerto Rican	AMR	American Ancestry	PUR	1000 Genomes on GRCh38,1000 Genomes 30x on GRCh38,1000 Genomes phase 3 release
**HG01253**	male	SAME125284	CLM	Colombian	AMR	American Ancestry	CLM	1000 Genomes on GRCh38,1000 Genomes 30x on GRCh38,1000 Genomes phase 3 release,1000 Genomes phase 1 release
**HG01258**	male	SAME125279	CLM	Colombian	AMR	American Ancestry	CLM	1000 Genomes 30x on GRCh38,1000 Genomes phase 3 release
**HG01275**	female	SAME123002	CLM	Colombian	AMR	American Ancestry	CLM	1000 Genomes on GRCh38,1000 Genomes 30x on GRCh38,1000 Genomes phase 3 release,1000 Genomes phase 1 release
**HG01260**	female	SAME122815	CLM	Colombian	AMR	American Ancestry	CLM	1000 Genomes on GRCh38,1000 Genomes 30x on GRCh38,1000 Genomes phase 3 release,1000 Genomes phase 1 release
**HG01302**	male	SAME1840038	PUR	Puerto Rican	AMR	American Ancestry	PUR	1000 Genomes on GRCh38,1000 Genomes 30x on GRCh38,1000 Genomes phase 3 release
**HG01272**	female	SAME122996	CLM	Colombian	AMR	American Ancestry	CLM	1000 Genomes on GRCh38,1000 Genomes 30x on GRCh38,1000 Genomes phase 3 release,1000 Genomes phase 1 release
**HG01504**	female	SAME124945	IBS	Iberian,Spanish	EUR	European Ancestry,West Eurasia (SGDP)	IBS,SpanishSGDP	1000 Genomes on GRCh38,Simons Genome Diversity Project,1000 Genomes 30x on GRCh38,1000 Genomes phase 3 release
**HG01277**	male	SAME123000	CLM	Colombian	AMR	American Ancestry	CLM	1000 Genomes on GRCh38,1000 Genomes 30x on GRCh38,1000 Genomes phase 3 release,1000 Genomes phase 1 release
**HG01509**	male	SAME124940	IBS	Iberian	EUR	European Ancestry	IBS	1000 Genomes on GRCh38,1000 Genomes 30x on GRCh38,1000 Genomes phase 3 release
**HG01354**	female	SAME124680	CLM	Colombian	AMR	American Ancestry	CLM	1000 Genomes on GRCh38,1000 Genomes 30x on GRCh38,1000 Genomes phase 3 release,1000 Genomes phase 1 release
**HG01511**	female	SAME124770	IBS	Iberian	EUR	European Ancestry	IBS	1000 Genomes 30x on GRCh38,1000 Genomes phase 3 release
**HG01359**	male	SAME124687	CLM	Colombian	AMR	American Ancestry	CLM	1000 Genomes on GRCh38,1000 Genomes 30x on GRCh38,1000 Genomes phase 3 release,1000 Genomes phase 1 release
**HG01516**	female	SAME124773	IBS	Iberian	EUR	European Ancestry	IBS	1000 Genomes on GRCh38,1000 Genomes 30x on GRCh38,1000 Genomes phase 3 release,1000 Genomes phase 1 release
**HG01361**	female	SAME124504	CLM	Colombian	AMR	American Ancestry	CLM	1000 Genomes 30x on GRCh38,1000 Genomes phase 3 release
**HG01523**	male	SAME125322	IBS	Iberian	EUR	European Ancestry	IBS	1000 Genomes 30x on GRCh38,1000 Genomes phase 3 release
**HG01366**	female	SAME125265	CLM	Colombian	AMR	American Ancestry	CLM	1000 Genomes on GRCh38,1000 Genomes 30x on GRCh38,1000 Genomes phase 3 release,1000 Genomes phase 1 release
**HG01528**	female	SAME125329	IBS	Iberian	EUR	European Ancestry	IBS	1000 Genomes on GRCh38,1000 Genomes 30x on GRCh38,1000 Genomes phase 3 release
**HG01378**	female	SAME124076	CLM	Colombian	AMR	American Ancestry	CLM	1000 Genomes on GRCh38,1000 Genomes 30x on GRCh38,1000 Genomes phase 3 release,1000 Genomes phase 1 release
**HG01597**	female	SAME125226	KHV	Kinh Vietnamese	EAS	East Asian Ancestry	KHV	1000 Genomes on GRCh38,1000 Genomes 30x on GRCh38,1000 Genomes phase 3 release
**HG01385**	female	SAME122970	CLM	Colombian	AMR	American Ancestry	CLM	1000 Genomes 30x on GRCh38,1000 Genomes phase 3 release
**HG01600**	female	SAME123456	KHV	Kinh,Kinh Vietnamese	EAS	East Asia (SGDP),East Asian Ancestry	KinhSGDP,KHV	1000 Genomes on GRCh38,Simons Genome Diversity Project,1000 Genomes 30x on GRCh38,1000 Genomes phase 3 release
**HG01392**	male	SAME1840234	PUR	Puerto Rican	AMR	American Ancestry	PUR	1000 Genomes on GRCh38,1000 Genomes 30x on GRCh38,1000 Genomes phase 3 release
**HG01605**	female	SAME123459	IBS	Iberian	EUR	European Ancestry	IBS	1000 Genomes on GRCh38,1000 Genomes 30x on GRCh38,1000 Genomes phase 3 release
**HG01049**	female	SAME125248	PUR	Puerto Rican	AMR	American Ancestry	PUR	1000 Genomes on GRCh38,1000 Genomes 30x on GRCh38,1000 Genomes phase 3 release,1000 Genomes phase 1 release
**HG01612**	female	SAME123625	IBS	Iberian	EUR	European Ancestry	IBS	1000 Genomes on GRCh38,1000 Genomes 30x on GRCh38,1000 Genomes phase 3 release
**HG01051**	male	SAME123712	PUR	Puerto Rican	AMR	American Ancestry	PUR	1000 Genomes on GRCh38,1000 Genomes 30x on GRCh38,1000 Genomes phase 3 release,1000 Genomes phase 1 release
**HG01530**	male	SAME125137	IBS	Iberian	EUR	European Ancestry	IBS	1000 Genomes on GRCh38,1000 Genomes 30x on GRCh38,1000 Genomes phase 3 release
**HG01056**	male	SAME123658	PUR	Puerto Rican	AMR	American Ancestry	PUR	1000 Genomes 30x on GRCh38,1000 Genomes phase 3 release
**HG01566**	female	SAME124564	PEL	Peruvian	AMR	American Ancestry	PEL	1000 Genomes on GRCh38,1000 Genomes 30x on GRCh38,1000 Genomes phase 3 release
**HG01063**	male	SAME1839767	PUR	Puerto Rican	AMR	American Ancestry	PUR	1000 Genomes on GRCh38,1000 Genomes 30x on GRCh38,1000 Genomes phase 3 release
**HG01573**	female	SAME124347	PEL	Peruvian	AMR	American Ancestry	PEL	1000 Genomes 30x on GRCh38,Human Genome Structural Variation Consortium, Phase 2,1000 Genomes phase 3 release
**HG01068**	female	SAME122826	PUR	Puerto Rican	AMR	American Ancestry	PUR	1000 Genomes 30x on GRCh38,1000 Genomes phase 3 release
**HG01578**	female	SAME124351	PEL	Peruvian	AMR	American Ancestry	PEL	1000 Genomes on GRCh38,1000 Genomes 30x on GRCh38,1000 Genomes phase 3 release
**HG01070**	female	SAME123993	PUR	Puerto Rican	AMR	American Ancestry	PUR	1000 Genomes on GRCh38,1000 Genomes 30x on GRCh38,1000 Genomes phase 3 release,1000 Genomes phase 1 release
**HG01617**	male	SAME123630	IBS	Iberian	EUR	European Ancestry	IBS	1000 Genomes on GRCh38,1000 Genomes 30x on GRCh38,1000 Genomes phase 3 release,1000 Genomes phase 1 release
**HG01075**	male	SAME124071	PUR	Puerto Rican	AMR	American Ancestry	PUR	1000 Genomes on GRCh38,1000 Genomes 30x on GRCh38,1000 Genomes phase 3 release,1000 Genomes phase 1 release
**HG01624**	male	SAME123840	IBS	Iberian	EUR	European Ancestry	IBS	1000 Genomes on GRCh38,1000 Genomes 30x on GRCh38,1000 Genomes phase 3 release,1000 Genomes phase 1 release
**HG00673**	male	SAME123605	CHS	Southern Han Chinese	EAS	East Asian Ancestry	CHS	1000 Genomes 30x on GRCh38,1000 Genomes phase 3 release
**HG01629**	male	SAME123836	IBS	Iberian	EUR	European Ancestry	IBS	1000 Genomes 30x on GRCh38,1000 Genomes phase 3 release
**HG00685**	male	SAME123891	CHS	Southern Han Chinese	EAS	East Asian Ancestry	CHS	1000 Genomes 30x on GRCh38,1000 Genomes phase 3 release
**HG01631**	male	SAME124035	IBS	Iberian	EUR	European Ancestry	IBS	1000 Genomes on GRCh38,1000 Genomes 30x on GRCh38,1000 Genomes phase 3 release
**HG00692**	male	SAME123684	CHS	Southern Han Chinese	EAS	East Asian Ancestry	CHS	1000 Genomes on GRCh38,1000 Genomes 30x on GRCh38,1000 Genomes phase 3 release,1000 Genomes phase 1 release
**HG01667**	male	SAME123116	IBS	Iberian	EUR	European Ancestry	IBS	1000 Genomes 30x on GRCh38,1000 Genomes phase 3 release
**HG00700**	female	SAME122932	CHS	Southern Han Chinese	EAS	East Asian Ancestry	CHS	1000 Genomes 30x on GRCh38,1000 Genomes phase 3 release
**HG01674**	male	SAME123286	IBS	Iberian	EUR	European Ancestry	IBS	1000 Genomes 30x on GRCh38,1000 Genomes phase 3 release
**HG00705**	female	SAME122927	CHS	Southern Han Chinese	EAS	East Asian Ancestry	CHS	1000 Genomes on GRCh38,1000 Genomes 30x on GRCh38,1000 Genomes phase 3 release,1000 Genomes phase 1 release
**HG00717**	female	SAME124926	CHS	Southern Han Chinese	EAS	East Asian Ancestry	CHS	1000 Genomes on GRCh38,1000 Genomes 30x on GRCh38,1000 Genomes phase 3 release
**HG01284**	female	SAME1839191	CLM	Colombian	AMR	American Ancestry	CLM	1000 Genomes on GRCh38,1000 Genomes 30x on GRCh38,1000 Genomes phase 3 release

**HG01489**	female	SAME123972	CLM	Colombian	AMR	American Ancestry	CLM	1000 Genomes on GRCh38,1000 Genomes 30x on GRCh38,1000 Genomes phase 3 release,1000 Genomes phase 1 release
**HG01311**	male	SAME1839796	PUR	Puerto Rican	AMR	American Ancestry	PUR	1000 Genomes on GRCh38,1000 Genomes 30x on GRCh38,1000 Genomes phase 3 release
**HG01491**	male	SAME124140	CLM	Colombian	AMR	American Ancestry	CLM	1000 Genomes on GRCh38,1000 Genomes 30x on GRCh38,1000 Genomes phase 3 release,1000 Genomes phase 1 release
**HG01323**	female	SAME1839534	PUR	Puerto Rican	AMR	American Ancestry	PUR	1000 Genomes on GRCh38,1000 Genomes 30x on GRCh38,1000 Genomes phase 3 release
**HG01496**	female	SAME124133	CLM	Colombian	AMR	American Ancestry	CLM	1000 Genomes 30x on GRCh38,1000 Genomes phase 3 release
**HG01342**	female	SAME124856	CLM	Colombian	AMR	American Ancestry	CLM	1000 Genomes on GRCh38,1000 Genomes 30x on GRCh38,1000 Genomes phase 3 release,1000 Genomes phase 1 release
**HG01679**	female	SAME123293	IBS	Iberian	EUR	European Ancestry	IBS	1000 Genomes on GRCh38,1000 Genomes 30x on GRCh38,1000 Genomes phase 3 release
**HG01347**	male	SAME124861	CLM	Colombian	AMR	American Ancestry	CLM	1000 Genomes on GRCh38,1000 Genomes 30x on GRCh38,1000 Genomes phase 3 release,1000 Genomes phase 1 release
**HG01681**	female	SAME124655	IBS	Iberian	EUR	European Ancestry	IBS	1000 Genomes 30x on GRCh38,1000 Genomes phase 3 release
**HG01599**	female	SAME125238	KHV	Kinh Vietnamese	EAS	East Asian Ancestry	KHV	1000 Genomes on GRCh38,1000 Genomes 30x on GRCh38,1000 Genomes phase 3 release
**HG01686**	male	SAME124658	IBS	Iberian	EUR	European Ancestry	IBS	1000 Genomes on GRCh38,1000 Genomes 30x on GRCh38,1000 Genomes phase 3 release
**HG01602**	female	SAME123454	IBS	Iberian	EUR	European Ancestry	IBS	1000 Genomes on GRCh38,1000 Genomes 30x on GRCh38,1000 Genomes phase 3 release
**HG01698**	male	SAME1839214	IBS	Iberian	EUR	European Ancestry	IBS	1000 Genomes 30x on GRCh38,1000 Genomes phase 3 release
**HG01607**	female	SAME123457	IBS	Iberian	EUR	European Ancestry	IBS	1000 Genomes on GRCh38,1000 Genomes 30x on GRCh38,1000 Genomes phase 3 release
**HG01701**	male	SAME123262	IBS	Iberian	EUR	European Ancestry	IBS	1000 Genomes 30x on GRCh38,1000 Genomes phase 3 release
**HG01614**	female	SAME123627	IBS	Iberian	EUR	European Ancestry	IBS	1000 Genomes 30x on GRCh38,1000 Genomes phase 3 release
**HG01768**	female	SAME1839665	IBS	Iberian	EUR	European Ancestry	IBS	1000 Genomes on GRCh38,1000 Genomes 30x on GRCh38,1000 Genomes phase 3 release
**HG01619**	male	SAME123632	IBS	Iberian	EUR	European Ancestry	IBS	1000 Genomes on GRCh38,1000 Genomes 30x on GRCh38,1000 Genomes phase 3 release,1000 Genomes phase 1 release
**HG01770**	female	SAME124638	IBS	Iberian	EUR	European Ancestry	IBS	1000 Genomes on GRCh38,1000 Genomes 30x on GRCh38,1000 Genomes phase 3 release
**HG01621**	male	SAME123843	IBS	Iberian	EUR	European Ancestry	IBS	1000 Genomes 30x on GRCh38,1000 Genomes phase 3 release
**HG01775**	male	SAME124633	IBS	Iberian	EUR	European Ancestry	IBS	1000 Genomes on GRCh38,1000 Genomes 30x on GRCh38,1000 Genomes phase 3 release
**HG01626**	female	SAME123842	IBS	Iberian	EUR	European Ancestry	IBS	1000 Genomes on GRCh38,1000 Genomes 30x on GRCh38,1000 Genomes phase 3 release,1000 Genomes phase 1 release
**HG01782**	female	SAME124426	IBS	Iberian	EUR	European Ancestry	IBS	1000 Genomes 30x on GRCh38,1000 Genomes phase 3 release
**HG01486**	female	SAME1840048	CLM	Colombian	AMR	American Ancestry	CLM	1000 Genomes on GRCh38,1000 Genomes 30x on GRCh38,1000 Genomes phase 3 release
**HG01493**	female	SAME124138	CLM	Colombian	AMR	American Ancestry	CLM	1000 Genomes 30x on GRCh38,1000 Genomes phase 3 release
**HG01439**	female	SAME124972	CLM	Colombian	AMR	American Ancestry	CLM	1000 Genomes 30x on GRCh38,1000 Genomes phase 3 release
**HG01498**	female	SAME124142	CLM	Colombian	AMR	American Ancestry	CLM	1000 Genomes on GRCh38,1000 Genomes 30x on GRCh38,1000 Genomes phase 3 release,1000 Genomes phase 1 release
**HG01441**	female	SAME123236	CLM	Colombian	AMR	American Ancestry	CLM	1000 Genomes on GRCh38,1000 Genomes 30x on GRCh38,1000 Genomes phase 3 release,1000 Genomes phase 1 release
**HG01501**	female	SAME124942	IBS	Iberian	EUR	European Ancestry	IBS	1000 Genomes on GRCh38,1000 Genomes 30x on GRCh38,1000 Genomes phase 3 release
**HG01506**	male	SAME124947	IBS	Iberian	EUR	European Ancestry	IBS	1000 Genomes on GRCh38,1000 Genomes 30x on GRCh38,1000 Genomes phase 3 release
**HG01465**	female	SAME122882	CLM	Colombian	AMR	American Ancestry	CLM	1000 Genomes on GRCh38,1000 Genomes 30x on GRCh38,1000 Genomes phase 3 release,1000 Genomes phase 1 release
**HG01513**	female	SAME124768	IBS	Iberian	EUR	European Ancestry	IBS	1000 Genomes on GRCh38,1000 Genomes 30x on GRCh38,1000 Genomes phase 3 release
**HG01706**	female	SAME123263	IBS	Iberian	EUR	European Ancestry	IBS	1000 Genomes 30x on GRCh38,1000 Genomes phase 3 release
**HG01518**	male	SAME124766	IBS	Iberian	EUR	European Ancestry	IBS	1000 Genomes on GRCh38,1000 Genomes 30x on GRCh38,1000 Genomes phase 3 release,1000 Genomes phase 1 release
**HG01756**	male	SAME124988	IBS	Iberian	EUR	European Ancestry	IBS	1000 Genomes on GRCh38,1000 Genomes 30x on GRCh38,1000 Genomes phase 3 release
**HG01520**	female	SAME125323	IBS	Iberian	EUR	European Ancestry	IBS	1000 Genomes 30x on GRCh38,1000 Genomes phase 3 release
**HG01763**	female	SAME124812	IBS	Iberian	EUR	European Ancestry	IBS	1000 Genomes 30x on GRCh38,1000 Genomes phase 3 release
**HG01525**	female	SAME125328	IBS	Iberian	EUR	European Ancestry	IBS	1000 Genomes on GRCh38,1000 Genomes 30x on GRCh38,1000 Genomes phase 3 release
**HG01946**	male	SAME124154	PEL	Peruvian	AMR	American Ancestry	PEL	1000 Genomes 30x on GRCh38,1000 Genomes phase 3 release
**HG01532**	male	SAME125138	IBS	Iberian	EUR	European Ancestry	IBS	1000 Genomes 30x on GRCh38,1000 Genomes phase 3 release
**HG01953**	male	SAME123974	PEL	Peruvian	AMR	American Ancestry	PEL	1000 Genomes on GRCh38,1000 Genomes 30x on GRCh38,1000 Genomes phase 3 release
**HG01537**	female	SAME125134	IBS	Iberian	EUR	European Ancestry	IBS	1000 Genomes on GRCh38,1000 Genomes 30x on GRCh38,1000 Genomes phase 3 release
**HG01958**	female	SAME1839349	ACB	African Caribbean	AFR	African Ancestry	ACB	1000 Genomes on GRCh38,1000 Genomes 30x on GRCh38,1000 Genomes phase 3 release
**HG01960**	female	SAME1839076	ACB	African Caribbean	AFR	African Ancestry	ACB	1000 Genomes 30x on GRCh38,1000 Genomes phase 3 release
**HG01405**	male	SAME1839863	PUR	Puerto Rican	AMR	American Ancestry	PUR	1000 Genomes on GRCh38,1000 Genomes 30x on GRCh38,1000 Genomes phase 3 release
**HG01965**	female	SAME1839145	PEL	Peruvian	AMR	American Ancestry	PEL	1000 Genomes on GRCh38,1000 Genomes 30x on GRCh38,1000 Genomes phase 3 release
**HG01412**	male	SAME1839619	PUR	Puerto Rican	AMR	American Ancestry	PUR	1000 Genomes on GRCh38,1000 Genomes 30x on GRCh38,1000 Genomes phase 3 release
**HG01972**	female	SAME124329	PEL	Peruvian	AMR	American Ancestry	PEL	1000 Genomes 30x on GRCh38,1000 Genomes phase 3 release
**HG01431**	male	SAME1839260	CLM	Colombian	AMR	American Ancestry	CLM	1000 Genomes on GRCh38,1000 Genomes 30x on GRCh38,1000 Genomes phase 3 release
**HG01977**	male	SAME124324	PEL	Peruvian	AMR	American Ancestry	PEL	1000 Genomes on GRCh38,1000 Genomes 30x on GRCh38,1000 Genomes phase 3 release
**HG01443**	male	SAME1839032	CLM	Colombian	AMR	American Ancestry	CLM	1000 Genomes on GRCh38,1000 Genomes 30x on GRCh38,1000 Genomes phase 3 release
**HG01984**	male	SAME1840105	PEL	Peruvian	AMR	American Ancestry	PEL	1000 Genomes 30x on GRCh38,1000 Genomes phase 3 release
**HG01455**	male	SAME123429	CLM	Colombian	AMR	American Ancestry	CLM	1000 Genomes on GRCh38,1000 Genomes 30x on GRCh38,1000 Genomes phase 3 release,1000 Genomes phase 1 release
**HG01989**	female	SAME1840166	ACB	African Caribbean	AFR	African Ancestry	ACB	1000 Genomes on GRCh38,1000 Genomes 30x on GRCh38,1000 Genomes phase 3 release
**HG01462**	female	SAME123539	CLM	Colombian	AMR	American Ancestry	CLM	1000 Genomes on GRCh38,1000 Genomes 30x on GRCh38,1000 Genomes phase 3 release,1000 Genomes phase 1 release
**HG01922**	female	SAME123787	PEL	Peruvian	AMR	American Ancestry	PEL	1000 Genomes 30x on GRCh38,1000 Genomes phase 3 release
**HG01474**	female	SAME1840189	CLM	Colombian	AMR	American Ancestry	CLM	1000 Genomes on GRCh38,1000 Genomes 30x on GRCh38,1000 Genomes phase 3 release
**HG01927**	female	SAME123784	PEL	Peruvian	AMR	American Ancestry	PEL	1000 Genomes on GRCh38,1000 Genomes 30x on GRCh38,1000 Genomes phase 3 release
**HG01479**	male	SAME1840251	CLM	Colombian	AMR	American Ancestry	CLM	1000 Genomes on GRCh38,1000 Genomes 30x on GRCh38,1000 Genomes phase 3 release
**HG01934**	male	SAME123589	PEL	Peruvian	AMR	American Ancestry	PEL	1000 Genomes 30x on GRCh38,1000 Genomes phase 3 release
**HG01939**	female	SAME123592	PEL	Peruvian	AMR	American Ancestry	PEL	1000 Genomes on GRCh38,1000 Genomes 30x on GRCh38,1000 Genomes phase 3 release
**HG01551**	female	SAME124008	CLM	Colombian	AMR	American Ancestry	CLM	1000 Genomes on GRCh38,1000 Genomes 30x on GRCh38,1000 Genomes phase 3 release,1000 Genomes phase 1 release
**HG01941**	male	SAME124149	PEL	Peruvian	AMR	American Ancestry	PEL	1000 Genomes on GRCh38,1000 Genomes 30x on GRCh38,1000 Genomes phase 3 release
**HG01556**	male	SAME1840141	CLM	Colombian	AMR	American Ancestry	CLM	1000 Genomes on GRCh38,1000 Genomes 30x on GRCh38,1000 Genomes phase 3 release
**HG01864**	male	SAME123197	KHV	Kinh Vietnamese	EAS	East Asian Ancestry	KHV	1000 Genomes on GRCh38,1000 Genomes 30x on GRCh38,1000 Genomes phase 3 release
**HG01869**	female	SAME123192	KHV	Kinh Vietnamese	EAS	East Asian Ancestry	KHV	1000 Genomes on GRCh38,1000 Genomes 30x on GRCh38,1000 Genomes phase 3 release
**HG01633**	male	SAME124033	IBS	Iberian	EUR	European Ancestry	IBS	1000 Genomes 30x on GRCh38,1000 Genomes phase 3 release
**HG01871**	female	SAME123398	KHV	Kinh Vietnamese	EAS	East Asian Ancestry	KHV	1000 Genomes on GRCh38,1000 Genomes 30x on GRCh38,1000 Genomes phase 3 release
**HG01669**	male	SAME123110	IBS	Iberian	EUR	European Ancestry	IBS	1000 Genomes on GRCh38,1000 Genomes 30x on GRCh38,1000 Genomes phase 3 release
**HG01883**	female	SAME122848	ACB	African Caribbean	AFR	African Ancestry	ACB	1000 Genomes on GRCh38,1000 Genomes 30x on GRCh38,1000 Genomes phase 3 release
**HG01671**	male	SAME123291	IBS	Iberian	EUR	European Ancestry	IBS	1000 Genomes 30x on GRCh38,1000 Genomes phase 3 release
**HG02019**	female	SAME123450	KHV	Kinh Vietnamese	EAS	East Asian Ancestry	KHV	1000 Genomes on GRCh38,1000 Genomes 30x on GRCh38,1000 Genomes phase 3 release
**HG01676**	female	SAME123284	IBS	Iberian	EUR	European Ancestry	IBS	1000 Genomes on GRCh38,1000 Genomes 30x on GRCh38,1000 Genomes phase 3 release
**HG01683**	male	SAME124653	IBS	Iberian	EUR	European Ancestry	IBS	1000 Genomes 30x on GRCh38,1000 Genomes phase 3 release
**HG02026**	male	SAME125315	KHV	Kinh Vietnamese	EAS	East Asian Ancestry	KHV	1000 Genomes on GRCh38,1000 Genomes 30x on GRCh38,1000 Genomes phase 3 release
**HG01695**	female	SAME124845	IBS	Iberian	EUR	European Ancestry	IBS	1000 Genomes on GRCh38,1000 Genomes 30x on GRCh38,1000 Genomes phase 3 release
**HG02040**	male	SAME1839803	KHV	Kinh Vietnamese	EAS	East Asian Ancestry	KHV	1000 Genomes on GRCh38,1000 Genomes 30x on GRCh38,1000 Genomes phase 3 release
**HG01703**	male	SAME123260	IBS	Iberian	EUR	European Ancestry	IBS	1000 Genomes 30x on GRCh38,1000 Genomes phase 3 release
**HG01888**	male	SAME122852	ACB	African Caribbean	AFR	African Ancestry	ACB	1000 Genomes 30x on GRCh38,1000 Genomes phase 3 release
**HG01708**	male	SAME123253	IBS	Iberian	EUR	European Ancestry	IBS	1000 Genomes on GRCh38,1000 Genomes 30x on GRCh38,1000 Genomes phase 3 release
**HG01890**	male	SAME123028	ACB	African Caribbean	AFR	African Ancestry	ACB	1000 Genomes on GRCh38,1000 Genomes 30x on GRCh38,1000 Genomes phase 3 release
**HG01710**	female	SAME123084	IBS	Iberian	EUR	European Ancestry	IBS	1000 Genomes on GRCh38,1000 Genomes 30x on GRCh38,1000 Genomes phase 3 release
**HG01895**	male	SAME1840349	ACB	African Caribbean	AFR	African Ancestry	ACB	1000 Genomes 30x on GRCh38,1000 Genomes phase 3 release
**HG01746**	female	SAME125180	IBS	Iberian	EUR	European Ancestry	IBS	1000 Genomes on GRCh38,1000 Genomes 30x on GRCh38,1000 Genomes phase 3 release
**HG01915**	female	SAME1839757	ACB	African Caribbean	AFR	African Ancestry	ACB	1000 Genomes on GRCh38,1000 Genomes 30x on GRCh38,1000 Genomes phase 3 release
**HG01765**	male	SAME1839681	IBS	Iberian	EUR	European Ancestry	IBS	1000 Genomes on GRCh38,1000 Genomes 30x on GRCh38,1000 Genomes phase 3 release
**HG01991**	male	SAME124743	PEL	Peruvian	AMR	American Ancestry	PEL	1000 Genomes on GRCh38,1000 Genomes 30x on GRCh38,1000 Genomes phase 3 release
**HG01772**	female	SAME124636	IBS	Iberian	EUR	European Ancestry	IBS	1000 Genomes 30x on GRCh38,1000 Genomes phase 3 release
**HG02002**	male	SAME123618	PEL	Peruvian	AMR	American Ancestry	PEL	1000 Genomes on GRCh38,1000 Genomes 30x on GRCh38,1000 Genomes phase 3 release
**HG01998**	male	SAME124739	PEL	Peruvian	AMR	American Ancestry	PEL	1000 Genomes 30x on GRCh38,1000 Genomes phase 3 release
**HG02014**	male	SAME123448	ACB	African Caribbean	AFR	African Ancestry	ACB	1000 Genomes on GRCh38,1000 Genomes 30x on GRCh38,1000 Genomes phase 3 release
**HG02004**	female	SAME123620	PEL	Peruvian	AMR	American Ancestry	PEL	1000 Genomes 30x on GRCh38,1000 Genomes phase 3 release
**HG01840**	male	SAME123552	KHV	Kinh Vietnamese	EAS	East Asian Ancestry	KHV	1000 Genomes on GRCh38,1000 Genomes 30x on GRCh38,1000 Genomes phase 3 release
**HG02009**	male	SAME123622	ACB	African Caribbean	AFR	African Ancestry	ACB	1000 Genomes on GRCh38,1000 Genomes 30x on GRCh38,1000 Genomes phase 3 release
**HG01845**	female	SAME123555	KHV	Kinh Vietnamese	EAS	East Asian Ancestry	KHV	1000 Genomes on GRCh38,1000 Genomes 30x on GRCh38,1000 Genomes phase 3 release
**HG02011**	male	SAME123443	ACB	African Caribbean	AFR	African Ancestry	ACB	1000 Genomes 30x on GRCh38,Human Genome Structural Variation Consortium, Phase 2,1000 Genomes phase 3 release
**HG01852**	male	SAME123750	KHV	Kinh Vietnamese	EAS	East Asian Ancestry	KHV	1000 Genomes on GRCh38,1000 Genomes 30x on GRCh38,1000 Genomes phase 3 release
**HG02016**	female	SAME123446	KHV	Kinh Vietnamese	EAS	East Asian Ancestry	KHV	1000 Genomes on GRCh38,1000 Genomes 30x on GRCh38,1000 Genomes phase 3 release
**HG01857**	female	SAME123748	KHV	Kinh Vietnamese	EAS	East Asian Ancestry	KHV	1000 Genomes on GRCh38,1000 Genomes 30x on GRCh38,1000 Genomes phase 3 release
**HG02023**	male	SAME125307	KHV	Kinh Vietnamese	EAS	East Asian Ancestry	KHV	1000 Genomes on GRCh38,1000 Genomes 30x on GRCh38,1000 Genomes phase 3 release
**HG01794**	female	SAME124218	CDX	Dai Chinese	EAS	East Asian Ancestry	CDX	1000 Genomes on GRCh38,1000 Genomes 30x on GRCh38,1000 Genomes phase 3 release
**HG01777**	male	SAME124631	IBS	Iberian	EUR	European Ancestry	IBS	1000 Genomes on GRCh38,1000 Genomes 30x on GRCh38,1000 Genomes phase 3 release
**HG01799**	female	SAME124227	CDX	Dai Chinese	EAS	East Asian Ancestry	CDX	1000 Genomes on GRCh38,1000 Genomes 30x on GRCh38,1000 Genomes phase 3 release
**HG01784**	female	SAME124431	IBS	Iberian	EUR	European Ancestry	IBS	1000 Genomes on GRCh38,1000 Genomes 30x on GRCh38,1000 Genomes phase 3 release
**HG01802**	female	SAME124307	CDX	Dai Chinese	EAS	East Asian Ancestry	CDX	1000 Genomes on GRCh38,1000 Genomes 30x on GRCh38,1000 Genomes phase 3 release
**HG01789**	male	SAME124433	GBR	British	EUR	European Ancestry	GBR	1000 Genomes on GRCh38,1000 Genomes 30x on GRCh38,1000 Genomes phase 3 release,Geuvadis
**HG01807**	female	SAME124303	CDX	Dai Chinese	EAS	East Asian Ancestry	CDX	1000 Genomes on GRCh38,1000 Genomes 30x on GRCh38,1000 Genomes phase 3 release
**HG01791**	male	SAME124221	GBR	British	EUR	European Ancestry	GBR	1000 Genomes on GRCh38,1000 Genomes 30x on GRCh38,1000 Genomes phase 3 release,Geuvadis
**HG02076**	male	SAME124913	KHV	Kinh Vietnamese	EAS	East Asian Ancestry	KHV	1000 Genomes on GRCh38,1000 Genomes 30x on GRCh38,1000 Genomes phase 3 release
**HG01796**	female	SAME124220	CDX	Dai Chinese	EAS	East Asian Ancestry	CDX	1000 Genomes on GRCh38,1000 Genomes 30x on GRCh38,1000 Genomes phase 3 release
**HG02083**	male	SAME124962	KHV	Kinh Vietnamese	EAS	East Asian Ancestry	KHV	1000 Genomes 30x on GRCh38,1000 Genomes phase 3 release

**HG01974**	male	SAME124323	PEL	Peruvian	AMR	American Ancestry	PEL	1000 Genomes on GRCh38,1000 Genomes 30x on GRCh38,1000 Genomes phase 3 release
**HG02088**	male	SAME124820	KHV	Kinh Vietnamese	EAS	East Asian Ancestry	KHV	1000 Genomes on GRCh38,1000 Genomes 30x on GRCh38,1000 Genomes phase 3 release
**HG01979**	male	SAME124331	PEL	Peruvian	AMR	American Ancestry	PEL	1000 Genomes on GRCh38,1000 Genomes 30x on GRCh38,1000 Genomes phase 3 release
**HG02090**	male	SAME124651	PEL	Peruvian	AMR	American Ancestry	PEL	1000 Genomes on GRCh38,1000 Genomes 30x on GRCh38,1000 Genomes phase 3 release
**HG01981**	female	SAME125247	PEL	Peruvian	AMR	American Ancestry	PEL	1000 Genomes 30x on GRCh38,1000 Genomes phase 3 release
**HG02095**	female	SAME1839064	ACB	African Caribbean	AFR	African Ancestry	ACB	1000 Genomes on GRCh38,1000 Genomes 30x on GRCh38,1000 Genomes phase 3 release
**HG01986**	male	SAME1840091	ACB	African Caribbean	AFR	African Ancestry	ACB	1000 Genomes on GRCh38,1000 Genomes 30x on GRCh38,1000 Genomes phase 3 release
**HG02278**	female	SAME1840367	PEL	Peruvian	AMR	American Ancestry	PEL	1000 Genomes on GRCh38,1000 Genomes 30x on GRCh38,1000 Genomes phase 3 release
**HG01993**	female	SAME124741	PEL	Peruvian	AMR	American Ancestry	PEL	1000 Genomes 30x on GRCh38,1000 Genomes phase 3 release
**HG02280**	female	SAME1840096	ACB	African Caribbean	AFR	African Ancestry	ACB	1000 Genomes 30x on GRCh38,1000 Genomes phase 3 release
**HG01892**	male	SAME123026	PEL	Peruvian	AMR	American Ancestry	PEL	1000 Genomes on GRCh38,1000 Genomes 30x on GRCh38,1000 Genomes phase 3 release
**HG02285**	male	SAME122914	PEL	Peruvian	AMR	American Ancestry	PEL	1000 Genomes on GRCh38,1000 Genomes 30x on GRCh38,1000 Genomes phase 3 release
**HG01897**	male	SAME1840343	ACB	African Caribbean	AFR	African Ancestry	ACB	1000 Genomes 30x on GRCh38,1000 Genomes phase 3 release
**HG02292**	female	SAME1839943	PEL	Peruvian	AMR	American Ancestry	PEL	1000 Genomes on GRCh38,1000 Genomes 30x on GRCh38,1000 Genomes phase 3 release
**HG01912**	male	SAME1839779	ACB	African Caribbean	AFR	African Ancestry	ACB	1000 Genomes on GRCh38,1000 Genomes 30x on GRCh38,1000 Genomes phase 3 release
**HG02300**	male	SAME1839856	PEL	Peruvian	AMR	American Ancestry	PEL	1000 Genomes 30x on GRCh38,1000 Genomes phase 3 release
**HG01917**	male	SAME123235	PEL	Peruvian	AMR	American Ancestry	PEL	1000 Genomes on GRCh38,1000 Genomes 30x on GRCh38,1000 Genomes phase 3 release
**HG02139**	female	SAME123660	KHV	Kinh Vietnamese	EAS	East Asian Ancestry	KHV	1000 Genomes on GRCh38,1000 Genomes 30x on GRCh38,1000 Genomes phase 3 release
**HG01924**	female	SAME123785	PEL	Peruvian	AMR	American Ancestry	PEL	1000 Genomes on GRCh38,1000 Genomes 30x on GRCh38,1000 Genomes phase 3 release
**HG02141**	male	SAME124611	KHV	Kinh Vietnamese	EAS	East Asian Ancestry	KHV	1000 Genomes on GRCh38,1000 Genomes 30x on GRCh38,1000 Genomes phase 3 release
**HG01866**	male	SAME123195	KHV	Kinh Vietnamese	EAS	East Asian Ancestry	KHV	1000 Genomes on GRCh38,1000 Genomes 30x on GRCh38,1000 Genomes phase 3 release
**HG02146**	male	SAME1839613	PEL	Peruvian	AMR	American Ancestry	PEL	1000 Genomes on GRCh38,1000 Genomes 30x on GRCh38,1000 Genomes phase 3 release
**HG01873**	male	SAME123400	KHV	Kinh Vietnamese	EAS	East Asian Ancestry	KHV	1000 Genomes on GRCh38,1000 Genomes 30x on GRCh38,1000 Genomes phase 3 release
**HG02153**	female	SAME124791	CDX	Dai Chinese	EAS	East Asian Ancestry	CDX	1000 Genomes on GRCh38,1000 Genomes 30x on GRCh38,1000 Genomes phase 3 release
**HG01878**	female	SAME123394	KHV	Kinh Vietnamese	EAS	East Asian Ancestry	KHV	1000 Genomes on GRCh38,1000 Genomes 30x on GRCh38,1000 Genomes phase 3 release
**HG02052**	female	SAME1839545	ACB	African Caribbean	AFR	African Ancestry	ACB	1000 Genomes on GRCh38,1000 Genomes 30x on GRCh38,1000 Genomes phase 3 release
**HG01880**	female	SAME1839109	ACB	African Caribbean	AFR	African Ancestry	ACB	1000 Genomes on GRCh38,1000 Genomes 30x on GRCh38,1000 Genomes phase 3 release
**HG02057**	female	SAME124146	KHV	Kinh Vietnamese	EAS	East Asian Ancestry	KHV	1000 Genomes on GRCh38,1000 Genomes 30x on GRCh38,1000 Genomes phase 3 release
**HG01885**	male	SAME122844	ACB	African Caribbean	AFR	African Ancestry	ACB	1000 Genomes on GRCh38,1000 Genomes 30x on GRCh38,1000 Genomes phase 3 release
**HG02064**	male	SAME1839658	KHV	Kinh Vietnamese	EAS	East Asian Ancestry	KHV	1000 Genomes on GRCh38,1000 Genomes 30x on GRCh38,1000 Genomes phase 3 release
**HG01948**	female	SAME124144	PEL	Peruvian	AMR	American Ancestry	PEL	1000 Genomes on GRCh38,1000 Genomes 30x on GRCh38,1000 Genomes phase 3 release
**HG02069**	female	SAME123375	KHV	Kinh Vietnamese	EAS	East Asian Ancestry	KHV	1000 Genomes on GRCh38,1000 Genomes 30x on GRCh38,1000 Genomes phase 3 release
**HG01950**	male	SAME123977	PEL	Peruvian	AMR	American Ancestry	PEL	1000 Genomes on GRCh38,1000 Genomes 30x on GRCh38,1000 Genomes phase 3 release
**HG02071**	male	SAME123102	KHV	Kinh Vietnamese	EAS	East Asian Ancestry	KHV	1000 Genomes 30x on GRCh38,1000 Genomes phase 3 release
**HG01955**	female	SAME123979	PEL	Peruvian	AMR	American Ancestry	PEL	1000 Genomes 30x on GRCh38,1000 Genomes phase 3 release
**HG01967**	male	SAME124530	PEL	Peruvian	AMR	American Ancestry	PEL	1000 Genomes on GRCh38,1000 Genomes 30x on GRCh38,1000 Genomes phase 3 release
**HG02259**	male	SAME1839319	PEL	Peruvian	AMR	American Ancestry	PEL	1000 Genomes on GRCh38,1000 Genomes 30x on GRCh38,1000 Genomes phase 3 release
**HG01936**	female	SAME123591	PEL	Peruvian	AMR	American Ancestry	PEL	1000 Genomes on GRCh38,1000 Genomes 30x on GRCh38,1000 Genomes phase 3 release
**HG02261**	male	SAME1839134	PEL	Peruvian	AMR	American Ancestry	PEL	1000 Genomes 30x on GRCh38,1000 Genomes phase 3 release
**HG00158**	female	SAME124585	GBR	British	EUR	European Ancestry	GBR	1000 Genomes on GRCh38,1000 Genomes 30x on GRCh38,1000 Genomes phase 3 release,1000 Genomes phase 1 release,Geuvadis
**HG01943**	male	SAME124151	PEL	Peruvian	AMR	American Ancestry	PEL	1000 Genomes 30x on GRCh38,1000 Genomes phase 3 release
**HG02266**	female	SAME1839110	PEL	Peruvian	AMR	American Ancestry	PEL	1000 Genomes on GRCh38,1000 Genomes 30x on GRCh38,1000 Genomes phase 3 release
**HG00160**	male	SAME124780	GBR	British	EUR	European Ancestry	GBR	1000 Genomes on GRCh38,1000 Genomes 30x on GRCh38,1000 Genomes phase 3 release,1000 Genomes phase 1 release,Geuvadis
**HG01842**	male	SAME123554	KHV	Kinh Vietnamese	EAS	East Asian Ancestry	KHV	1000 Genomes on GRCh38,1000 Genomes 30x on GRCh38,1000 Genomes phase 3 release
**HG02273**	female	SAME1840309	PEL	Peruvian	AMR	American Ancestry	PEL	1000 Genomes 30x on GRCh38,1000 Genomes phase 3 release
**HG00177**	female	SAME124957	FIN	Finnish	EUR	European Ancestry	FIN	1000 Genomes on GRCh38,1000 Genomes 30x on GRCh38,1000 Genomes phase 3 release,1000 Genomes phase 1 release,Geuvadis
**HG01847**	female	SAME123557	KHV	Kinh Vietnamese	EAS	East Asian Ancestry	KHV	1000 Genomes on GRCh38,1000 Genomes 30x on GRCh38,1000 Genomes phase 3 release
**HG02108**	female	SAME1840201	ACB	African Caribbean	AFR	African Ancestry	ACB	1000 Genomes on GRCh38,1000 Genomes 30x on GRCh38,1000 Genomes phase 3 release
**HG00189**	male	SAME123640	FIN	Finnish	EUR	European Ancestry	FIN	1000 Genomes on GRCh38,1000 Genomes 30x on GRCh38,1000 Genomes phase 3 release,1000 Genomes phase 1 release,Geuvadis
**HG01859**	female	SAME123535	KHV	Kinh Vietnamese	EAS	East Asian Ancestry	KHV	1000 Genomes on GRCh38,1000 Genomes 30x on GRCh38,1000 Genomes phase 3 release
**HG02122**	male	SAME123478	KHV	Kinh Vietnamese	EAS	East Asian Ancestry	KHV	1000 Genomes on GRCh38,1000 Genomes 30x on GRCh38,1000 Genomes phase 3 release
**HG00235**	female	SAME124122	GBR	British	EUR	European Ancestry	GBR	1000 Genomes on GRCh38,1000 Genomes 30x on GRCh38,1000 Genomes phase 3 release,1000 Genomes phase 1 release,Geuvadis
**HG01861**	male	SAME123200	KHV	Kinh Vietnamese	EAS	East Asian Ancestry	KHV	1000 Genomes on GRCh38,1000 Genomes 30x on GRCh38,1000 Genomes phase 3 release
**HG02127**	female	SAME123476	KHV	Kinh Vietnamese	EAS	East Asian Ancestry	KHV	1000 Genomes on GRCh38,1000 Genomes 30x on GRCh38,1000 Genomes phase 3 release
**HG00242**	male	SAME123223	GBR	British	EUR	European Ancestry	GBR	1000 Genomes on GRCh38,1000 Genomes 30x on GRCh38,1000 Genomes phase 3 release,1000 Genomes phase 1 release,Geuvadis
**HG01804**	female	SAME124302	CDX	Dai Chinese	EAS	East Asian Ancestry	CDX	1000 Genomes on GRCh38,1000 Genomes 30x on GRCh38,1000 Genomes phase 3 release
**HG02134**	male	SAME1839875	KHV	Kinh Vietnamese	EAS	East Asian Ancestry	KHV	1000 Genomes on GRCh38,1000 Genomes 30x on GRCh38,1000 Genomes phase 3 release
**HG01809**	female	SAME124299	CDX	Dai Chinese	EAS	East Asian Ancestry	CDX	1000 Genomes on GRCh38,1000 Genomes 30x on GRCh38,1000 Genomes phase 3 release
**HG02312**	female	SAME1839684	PEL	Peruvian	AMR	American Ancestry	PEL	1000 Genomes on GRCh38,1000 Genomes 30x on GRCh38,1000 Genomes phase 3 release
**HG00254**	female	SAME123050	GBR	British	EUR	European Ancestry	GBR	1000 Genomes on GRCh38,1000 Genomes 30x on GRCh38,1000 Genomes phase 3 release,1000 Genomes phase 1 release
**HG01811**	male	SAME125380	CDX	Dai Chinese	EAS	East Asian Ancestry	CDX	1000 Genomes on GRCh38,1000 Genomes 30x on GRCh38,1000 Genomes phase 3 release
**HG02317**	male	SAME1839656	ACB	African Caribbean	AFR	African Ancestry	ACB	1000 Genomes on GRCh38,1000 Genomes 30x on GRCh38,1000 Genomes phase 3 release
**HG00259**	female	SAME123056	GBR	British	EUR	European Ancestry	GBR	1000 Genomes on GRCh38,1000 Genomes 30x on GRCh38,1000 Genomes phase 3 release,1000 Genomes phase 1 release,Geuvadis
**HG01816**	male	SAME124521	CDX	Dai Chinese	EAS	East Asian Ancestry	CDX	1000 Genomes on GRCh38,1000 Genomes 30x on GRCh38,1000 Genomes phase 3 release
**HG02343**	male	SAME1839091	ACB	African Caribbean	AFR	African Ancestry	ACB	1000 Genomes on GRCh38,1000 Genomes 30x on GRCh38,1000 Genomes phase 3 release
**HG00261**	female	SAME123583	GBR	British	EUR	European Ancestry	GBR	1000 Genomes on GRCh38,1000 Genomes 30x on GRCh38,1000 Genomes phase 3 release,1000 Genomes phase 1 release,Geuvadis
**HG02282**	female	SAME1840177	ACB	African Caribbean	AFR	African Ancestry	ACB	1000 Genomes on GRCh38,1000 Genomes 30x on GRCh38,1000 Genomes phase 3 release
**HG02223**	female	SAME1839584	IBS	Iberian	EUR	European Ancestry	IBS	1000 Genomes on GRCh38,1000 Genomes 30x on GRCh38,1000 Genomes phase 3 release
**HG00266**	female	SAME123576	FIN	Finnish	EUR	European Ancestry	FIN	1000 Genomes on GRCh38,1000 Genomes 30x on GRCh38,1000 Genomes phase 3 release,1000 Genomes phase 1 release,Geuvadis
**HG02287**	female	SAME123755	PEL	Peruvian	AMR	American Ancestry	PEL	1000 Genomes 30x on GRCh38,1000 Genomes phase 3 release
**HG02230**	female	SAME1839383	IBS	Iberian	EUR	European Ancestry	IBS	1000 Genomes on GRCh38,1000 Genomes 30x on GRCh38,1000 Genomes phase 3 release
**HG00273**	male	SAME123419	FIN	Finnish	EUR	European Ancestry	FIN	1000 Genomes on GRCh38,1000 Genomes 30x on GRCh38,1000 Genomes phase 3 release,1000 Genomes phase 1 release,Geuvadis
**HG02299**	male	SAME1839994	PEL	Peruvian	AMR	American Ancestry	PEL	1000 Genomes on GRCh38,1000 Genomes 30x on GRCh38,1000 Genomes phase 3 release
**HG02235**	female	SAME123521	IBS	Iberian	EUR	European Ancestry	IBS	1000 Genomes on GRCh38,1000 Genomes 30x on GRCh38,1000 Genomes phase 3 release
**HG00278**	male	SAME123425	FIN	Finnish	EUR	European Ancestry	FIN	1000 Genomes on GRCh38,1000 Genomes 30x on GRCh38,1000 Genomes phase 3 release,1000 Genomes phase 1 release,Geuvadis
**HG02302**	male	SAME124279	PEL	Peruvian	AMR	American Ancestry	PEL	1000 Genomes 30x on GRCh38,1000 Genomes phase 3 release
**HG02165**	female	SAME124208	CDX	Dai Chinese	EAS	East Asian Ancestry	CDX	1000 Genomes on GRCh38,1000 Genomes 30x on GRCh38,1000 Genomes phase 3 release
**HG00280**	male	SAME122831	FIN	Finnish	EUR	European Ancestry	FIN	1000 Genomes on GRCh38,1000 Genomes 30x on GRCh38,1000 Genomes phase 3 release,1000 Genomes phase 1 release,Geuvadis
**HG02307**	male	SAME1839535	ACB	African Caribbean	AFR	African Ancestry	ACB	1000 Genomes on GRCh38,1000 Genomes 30x on GRCh38,1000 Genomes phase 3 release
**HG02184**	female	SAME123216	CDX	Dai Chinese	EAS	East Asian Ancestry	CDX	1000 Genomes on GRCh38,1000 Genomes 30x on GRCh38,1000 Genomes phase 3 release
**HG00285**	female	SAME122836	FIN	Finnish	EUR	European Ancestry	FIN	1000 Genomes on GRCh38,1000 Genomes 30x on GRCh38,1000 Genomes phase 3 release,1000 Genomes phase 1 release,Geuvadis
**HG02143**	male	SAME1839628	ACB	African Caribbean	AFR	African Ancestry	ACB	1000 Genomes on GRCh38,1000 Genomes 30x on GRCh38,1000 Genomes phase 3 release
**HG00103**	male	SAME125151	GBR	British	EUR	European Ancestry	GBR	1000 Genomes on GRCh38,1000 Genomes 30x on GRCh38,1000 Genomes phase 3 release,1000 Genomes phase 1 release,Geuvadis
**HG02148**	female	SAME1839697	PEL	Peruvian	AMR	American Ancestry	PEL	1000 Genomes 30x on GRCh38,1000 Genomes phase 3 release
**HG02689**	male	SAME1839355	PJL	Punjabi	SAS	South Asian Ancestry	PJL	1000 Genomes 30x on GRCh38,1000 Genomes phase 3 release
**HG00108**	male	SAME125161	GBR	British	EUR	European Ancestry	GBR	1000 Genomes on GRCh38,1000 Genomes 30x on GRCh38,1000 Genomes phase 3 release,1000 Genomes phase 1 release,Geuvadis
**HG02150**	male	SAME1839407	PEL	Peruvian	AMR	American Ancestry	PEL	1000 Genomes on GRCh38,1000 Genomes 30x on GRCh38,1000 Genomes phase 3 release
**HG02691**	female	SAME1839084	PJL	Punjabi	SAS	South Asian Ancestry	PJL	1000 Genomes on GRCh38,1000 Genomes 30x on GRCh38,1000 Genomes phase 3 release
**HG00110**	female	SAME125339	GBR	British	EUR	European Ancestry	GBR	1000 Genomes on GRCh38,1000 Genomes 30x on GRCh38,1000 Genomes phase 3 release,1000 Genomes phase 1 release,Geuvadis
**HG02155**	female	SAME124795	CDX	Dai Chinese	EAS	East Asian Ancestry	CDX	1000 Genomes on GRCh38,1000 Genomes 30x on GRCh38,1000 Genomes phase 3 release
**HG02696**	male	SAME1839149	PJL	Punjabi	SAS	South Asian Ancestry	PJL	1000 Genomes on GRCh38,1000 Genomes 30x on GRCh38,1000 Genomes phase 3 release
**HG00115**	male	SAME125344	GBR	British	EUR	European Ancestry	GBR	1000 Genomes on GRCh38,1000 Genomes 30x on GRCh38,1000 Genomes phase 3 release,1000 Genomes phase 1 release,Geuvadis
**HG02028**	female	SAME124020	KHV	Kinh Vietnamese	EAS	East Asian Ancestry	KHV	1000 Genomes on GRCh38,1000 Genomes 30x on GRCh38,1000 Genomes phase 3 release
**HG02704**	female	SAME1840134	GWD	Gambian Mandinka	AFR	African Ancestry	GWD	1000 Genomes 30x on GRCh38
**HG00122**	female	SAME122876	GBR	British	EUR	European Ancestry	GBR	1000 Genomes on GRCh38,1000 Genomes 30x on GRCh38,1000 Genomes phase 3 release,1000 Genomes phase 1 release,Geuvadis
**HG02030**	male	SAME1839966	KHV	Kinh Vietnamese	EAS	East Asian Ancestry	KHV	1000 Genomes 30x on GRCh38,1000 Genomes phase 3 release
**HG02716**	female	SAME1839981	GWD	Gambian Mandinka	AFR	African Ancestry	GWD	1000 Genomes on GRCh38,1000 Genomes 30x on GRCh38,1000 Genomes phase 3 release,Gambian Genome Variation Project (GRCh38)
**HG00127**	female	SAME122871	GBR	British	EUR	European Ancestry	GBR	1000 Genomes on GRCh38,1000 Genomes 30x on GRCh38,1000 Genomes phase 3 release,1000 Genomes phase 1 release,Geuvadis
**HG02035**	male	SAME1840035	KHV	Kinh Vietnamese	EAS	East Asian Ancestry	KHV	1000 Genomes on GRCh38,1000 Genomes 30x on GRCh38,1000 Genomes phase 3 release
**HG02723**	female	SAME1839742	GWD	Gambian Mandinka	AFR	African Ancestry	GWD	1000 Genomes 30x on GRCh38
**HG02047**	male	SAME1839772	KHV	Kinh Vietnamese	EAS	East Asian Ancestry	KHV	1000 Genomes on GRCh38,1000 Genomes 30x on GRCh38,1000 Genomes phase 3 release
**HG02728**	female	SAME1839722	PJL	Punjabi	SAS	South Asian Ancestry	PJL	1000 Genomes on GRCh38,1000 Genomes 30x on GRCh38,1000 Genomes phase 3 release
**HG00139**	male	SAME123060	GBR	British	EUR	European Ancestry	GBR	1000 Genomes on GRCh38,1000 Genomes 30x on GRCh38,1000 Genomes phase 3 release,1000 Genomes phase 1 release,Geuvadis
**HG02054**	female	SAME1839538	ACB	African Caribbean	AFR	African Ancestry	ACB	1000 Genomes on GRCh38,1000 Genomes 30x on GRCh38,1000 Genomes phase 3 release
**HG02603**	male	SAME1840287	PJL	Punjabi	SAS	South Asian Ancestry	PJL	1000 Genomes on GRCh38,1000 Genomes 30x on GRCh38,1000 Genomes phase 3 release
**HG00141**	male	SAME124395	GBR	British	EUR	European Ancestry	GBR	1000 Genomes on GRCh38,1000 Genomes 30x on GRCh38,1000 Genomes phase 3 release,1000 Genomes phase 1 release,Geuvadis
**HG02059**	female	SAME1839596	KHV	Kinh Vietnamese	EAS	East Asian Ancestry	KHV	1000 Genomes 30x on GRCh38,Human Genome Structural Variation Consortium, Phase 2,1000 Genomes phase 3 release
**HG02610**	male	SAME1840405	GWD	Gambian Mandinka	AFR	African Ancestry	GWD	1000 Genomes on GRCh38,1000 Genomes 30x on GRCh38,1000 Genomes phase 3 release,Gambian Genome Variation Project (GRCh38)
**HG00146**	female	SAME124390	GBR	British	EUR	European Ancestry	GBR	1000 Genomes on GRCh38,1000 Genomes 30x on GRCh38,1000 Genomes phase 3 release,1000 Genomes phase 1 release,Geuvadis
**HG02061**	male	SAME1839676	KHV	Kinh Vietnamese	EAS	East Asian Ancestry	KHV	1000 Genomes on GRCh38,1000 Genomes 30x on GRCh38,1000 Genomes phase 3 release
**HG02615**	female	SAME1840380	GWD	Gambian Mandinka	AFR	African Ancestry	GWD	1000 Genomes 30x on GRCh38

HG02073	male	SAME123104	KHV	Kinh Vietnamese	EAS	East Asian Ancestry	KHV	1000 Genomes on GRCh38,1000 Genomes 30x on GRCh38,1000 Genomes phase 3 release
HG02622	female	SAME1840185	GWD	Gambian Mandinka	AFR	African Ancestry	GWD	1000 Genomes 30x on GRCh38
HG00324	female	SAME123794	FIN	Finnish	EUR	European Ancestry	FIN	1000 Genomes on GRCh38,1000 Genomes 30x on GRCh38,1000 Genomes phase 3 release,1000 Genomes phase 1 release,Geuvadis
HG02078	female	SAME123099	KHV	Kinh Vietnamese	EAS	East Asian Ancestry	KHV	1000 Genomes on GRCh38,1000 Genomes 30x on GRCh38,1000 Genomes phase 3 release
HG02634	male	SAME1840031	GWD	Gambian Mandinka	AFR	African Ancestry	GWD	1000 Genomes on GRCh38,1000 Genomes 30x on GRCh38,1000 Genomes phase 3 release,Gambian Genome Variation Project (GRCh38)
HG00329	male	SAME123798	FIN	Finnish	EUR	European Ancestry	FIN	1000 Genomes on GRCh38,1000 Genomes 30x on GRCh38,1000 Genomes phase 3 release,1000 Genomes phase 1 release,Geuvadis
HG02179	female	SAME124415	CDX	Dai Chinese	EAS	East Asian Ancestry	CDX	1000 Genomes on GRCh38,1000 Genomes 30x on GRCh38,1000 Genomes phase 3 release
HG02646	female	SAME1839770	GWD	Gambian Mandinka	AFR	African Ancestry	GWD	1000 Genomes on GRCh38,1000 Genomes 30x on GRCh38,1000 Genomes phase 3 release,Gambian Genome Variation Project (GRCh38)
HG00331	female	SAME123986	FIN	Finnish	EUR	European Ancestry	FIN	1000 Genomes on GRCh38,1000 Genomes 30x on GRCh38,1000 Genomes phase 3 release,1000 Genomes phase 1 release,Geuvadis
HG02181	female	SAME123206	CDX	Dai Chinese	EAS	East Asian Ancestry	CDX	1000 Genomes on GRCh38,1000 Genomes 30x on GRCh38,1000 Genomes phase 3 release
HG02557	male	SAME1840061	ACB	African Caribbean	AFR	African Ancestry	ACB	1000 Genomes on GRCh38,1000 Genomes 30x on GRCh38,1000 Genomes phase 3 release
HG00336	male	SAME123983	FIN	Finnish	EUR	European Ancestry	FIN	1000 Genomes on GRCh38,1000 Genomes 30x on GRCh38,1000 Genomes phase 3 release,1000 Genomes phase 1 release,Geuvadis
HG02186	female	SAME125039	CDX	Dai Chinese	EAS	East Asian Ancestry	CDX	1000 Genomes on GRCh38,1000 Genomes 30x on GRCh38,1000 Genomes phase 3 release
HG02569	male	SAME1839892	GWD	Gambian Mandinka	AFR	African Ancestry	GWD	1000 Genomes 30x on GRCh38
HG00343	female	SAME124923	FIN	Finnish	EUR	European Ancestry	FIN	1000 Genomes on GRCh38,1000 Genomes 30x on GRCh38,1000 Genomes phase 3 release,1000 Genomes phase 1 release,Geuvadis
HG02571	female	SAME1839577	GWD	Gambian Mandinka	AFR	African Ancestry	GWD	1000 Genomes on GRCh38,1000 Genomes 30x on GRCh38,1000 Genomes phase 3 release,Gambian Genome Variation Project (GRCh38)
HG00350	female	SAME125121	FIN	Finnish	EUR	European Ancestry	FIN	1000 Genomes on GRCh38,1000 Genomes 30x on GRCh38,1000 Genomes phase 3 release,1000 Genomes phase 1 release,Geuvadis
HG02220	female	SAME123715	IBS	Iberian	EUR	European Ancestry	IBS	1000 Genomes on GRCh38,1000 Genomes 30x on GRCh38,1000 Genomes phase 3 release
HG02583	female	SAME1839429	GWD	Gambian Mandinka	AFR	African Ancestry	GWD	1000 Genomes on GRCh38,1000 Genomes 30x on GRCh38,1000 Genomes phase 3 release,Gambian Genome Variation Project (GRCh38)
HG00355	female	SAME125125	FIN	Finnish	EUR	European Ancestry	FIN	1000 Genomes on GRCh38,1000 Genomes 30x on GRCh38,1000 Genomes phase 3 release,1000 Genomes phase 1 release,Geuvadis
HG02080	female	SAME124825	KHV	Kinh Vietnamese	EAS	East Asian Ancestry	KHV	1000 Genomes 30x on GRCh38,1000 Genomes phase 3 release
HG02588	male	SAME1839404	GWD	Gambian Mandinka	AFR	African Ancestry	GWD	1000 Genomes on GRCh38,1000 Genomes 30x on GRCh38,1000 Genomes phase 3 release,Gambian Genome Variation Project (GRCh38)
HG00362	female	SAME124537	FIN	Finnish	EUR	European Ancestry	FIN	1000 Genomes on GRCh38,1000 Genomes 30x on GRCh38,1000 Genomes phase 3 release,1000 Genomes phase 1 release,Geuvadis
HG02085	male	SAME124979	KHV	Kinh Vietnamese	EAS	East Asian Ancestry	KHV	1000 Genomes on GRCh38,1000 Genomes 30x on GRCh38,1000 Genomes phase 3 release
HG02590	male	SAME1839222	GWD	Gambian Mandinka	AFR	African Ancestry	GWD	1000 Genomes 30x on GRCh38
HG00367	female	SAME124540	FIN	Finnish	EUR	European Ancestry	FIN	1000 Genomes on GRCh38,1000 Genomes 30x on GRCh38,1000 Genomes phase 3 release,1000 Genomes phase 1 release,Geuvadis
HG02105	female	SAME123873	PEL	Peruvian	AMR	American Ancestry	PEL	1000 Genomes on GRCh38,1000 Genomes 30x on GRCh38,1000 Genomes phase 3 release
HG02595	female	SAME1839204	GWD	Gambian Mandinka	AFR	African Ancestry	GWD	1000 Genomes on GRCh38,1000 Genomes 30x on GRCh38,1000 Genomes phase 3 release,Gambian Genome Variation Project (GRCh38)
HG00475	male	SAME123342	CHS	Southern Han Chinese	EAS	East Asian Ancestry	CHS	1000 Genomes on GRCh38,1000 Genomes 30x on GRCh38,1000 Genomes phase 3 release,1000 Genomes phase 1 release,90 Han Chinese high coverage genomes
HG02129	male	SAME1839724	KHV	Kinh Vietnamese	EAS	East Asian Ancestry	KHV	1000 Genomes 30x on GRCh38,1000 Genomes phase 3 release
HG02420	male	SAME1839320	ACB	African Caribbean	AFR	African Ancestry	ACB	1000 Genomes on GRCh38,1000 Genomes 30x on GRCh38,1000 Genomes phase 3 release
HG00502	male	SAME123361	CHS	Southern Han Chinese	EAS	East Asian Ancestry	CHS	1000 Genomes 30x on GRCh38,1000 Genomes phase 3 release
HG02131	male	SAME1839896	KHV	Kinh Vietnamese	EAS	East Asian Ancestry	KHV	1000 Genomes on GRCh38,1000 Genomes 30x on GRCh38,1000 Genomes phase 3 release
HG02425	female	SAME1839043	PEL	Peruvian	AMR	American Ancestry	PEL	1000 Genomes on GRCh38,1000 Genomes 30x on GRCh38,1000 Genomes phase 3 release
HG00514	female	SAME123522	CHS	Southern Han Chinese	EAS	East Asian Ancestry	CHS	1000 Genomes 30x on GRCh38,Human Genome Structural Variation Consortium, Phase 2,The Human Genome Structural Variation Consortium,1000 Genomes phase 3 re
HG02136	female	SAME1839865	KHV	Kinh Vietnamese	EAS	East Asian Ancestry	KHV	1000 Genomes on GRCh38,1000 Genomes 30x on GRCh38,1000 Genomes phase 3 release
HG02449	male	SAME1840404	ACB	African Caribbean	AFR	African Ancestry	ACB	1000 Genomes on GRCh38,1000 Genomes 30x on GRCh38,1000 Genomes phase 3 release
HG00526	male	SAME123395	CHS	Southern Han Chinese	EAS	East Asian Ancestry	CHS	1000 Genomes 30x on GRCh38,1000 Genomes phase 3 release
HG02225	female	SAME1839576	IBS	Iberian	EUR	European Ancestry	IBS	1000 Genomes 30x on GRCh38,1000 Genomes phase 3 release
HG02451	female	SAME1840138	ACB	African Caribbean	AFR	African Ancestry	ACB	1000 Genomes 30x on GRCh38,1000 Genomes phase 3 release
HG00533	male	SAME124353	CHS	Southern Han Chinese	EAS	East Asian Ancestry	CHS	1000 Genomes on GRCh38,1000 Genomes 30x on GRCh38,1000 Genomes phase 3 release,1000 Genomes phase 1 release,90 Han Chinese high coverage genomes
HG02232	female	SAME1839374	IBS	Iberian	EUR	European Ancestry	IBS	1000 Genomes on GRCh38,1000 Genomes 30x on GRCh38,1000 Genomes phase 3 release
HG02463	male	SAME1839983	GWD	Gambian Mandinka	AFR	African Ancestry	GWD	1000 Genomes 30x on GRCh38
HG00538	male	SAME124343	CHS	Southern Han Chinese	EAS	East Asian Ancestry	CHS	1000 Genomes 30x on GRCh38,1000 Genomes phase 3 release
HG02237	female	SAME123519	IBS	Iberian	EUR	European Ancestry	IBS	1000 Genomes 30x on GRCh38,1000 Genomes phase 3 release
HG02348	female	SAME1839063	PEL	Peruvian	AMR	American Ancestry	PEL	1000 Genomes on GRCh38,1000 Genomes 30x on GRCh38,1000 Genomes phase 3 release
HG00552	male	SAME124253	PUR	Puerto Rican	AMR	American Ancestry	PUR	1000 Genomes 30x on GRCh38,1000 Genomes phase 3 release
HG02256	female	SAME1839332	ACB	African Caribbean	AFR	African Ancestry	ACB	1000 Genomes on GRCh38,1000 Genomes 30x on GRCh38,1000 Genomes phase 3 release
HG02355	male	SAME1840269	CDX	Dai Chinese	EAS	East Asian Ancestry	CDX	1000 Genomes on GRCh38,1000 Genomes 30x on GRCh38,1000 Genomes phase 3 release
HG00557	female	SAME124258	CHS	Southern Han Chinese	EAS	East Asian Ancestry	CHS	1000 Genomes on GRCh38,1000 Genomes 30x on GRCh38,1000 Genomes phase 3 release,1000 Genomes phase 1 release,90 Han Chinese high coverage genomes
HG02275	female	SAME1840376	PEL	Peruvian	AMR	American Ancestry	PEL	1000 Genomes on GRCh38,1000 Genomes 30x on GRCh38,1000 Genomes phase 3 release
HG02367	male	SAME1840113	CDX	Dai Chinese	EAS	East Asian Ancestry	CDX	1000 Genomes on GRCh38,1000 Genomes 30x on GRCh38,1000 Genomes phase 3 release
HG00583	male	SAME124829	CHS	Southern Han Chinese	EAS	East Asian Ancestry	CHS	1000 Genomes on GRCh38,1000 Genomes 30x on GRCh38,1000 Genomes phase 3 release,1000 Genomes phase 1 release,90 Han Chinese high coverage genomes
HG02605	female	SAME1840277	PJL	Punjabi	SAS	South Asian Ancestry	PJL	1000 Genomes 30x on GRCh38,1000 Genomes phase 3 release
HG02374	male	SAME1840247	CDX	Dai Chinese	EAS	East Asian Ancestry	CDX	1000 Genomes on GRCh38,1000 Genomes 30x on GRCh38,1000 Genomes phase 3 release
HG00379	female	SAME124746	FIN	Finnish	EUR	European Ancestry	FIN	1000 Genomes on GRCh38,1000 Genomes 30x on GRCh38,1000 Genomes phase 3 release,1000 Genomes phase 1 release,Geuvadis
HG02612	male	SAME1840394	GWD	Gambian Mandinka	AFR	African Ancestry	GWD	1000 Genomes 30x on GRCh38
HG02379	male	SAME1840217	CDX	Dai Chinese	EAS	East Asian Ancestry	CDX	1000 Genomes on GRCh38,1000 Genomes 30x on GRCh38,1000 Genomes phase 3 release
HG00381	female	SAME123030	FIN	Finnish	EUR	European Ancestry	FIN	1000 Genomes on GRCh38,1000 Genomes 30x on GRCh38,1000 Genomes phase 3 release,1000 Genomes phase 1 release,Geuvadis
HG02624	female	SAME1840259	GWD	Gambian Mandinka	AFR	African Ancestry	GWD	1000 Genomes on GRCh38,1000 Genomes 30x on GRCh38,1000 Genomes phase 3 release,Gambian Genome Variation Project (GRCh38)
HG00406	male	SAME123156	CHS	Southern Han Chinese	EAS	East Asian Ancestry	CHS	1000 Genomes on GRCh38,1000 Genomes 30x on GRCh38,1000 Genomes phase 3 release,1000 Genomes phase 1 release
HG02629	female	SAME1840230	GWD	Gambian Mandinka	AFR	African Ancestry	GWD	1000 Genomes on GRCh38,1000 Genomes 30x on GRCh38,1000 Genomes phase 3 release,Gambian Genome Variation Project (GRCh38)
HG02653	female	SAME1839537	PJL	Punjabi	SAS	South Asian Ancestry	PJL	1000 Genomes 30x on GRCh38
HG00418	male	SAME122992	CHS	Southern Han Chinese	EAS	East Asian Ancestry	CHS	1000 Genomes on GRCh38,1000 Genomes 30x on GRCh38,1000 Genomes phase 3 release,1000 Genomes phase 1 release,90 Han Chinese high coverage genomes
HG02636	male	SAME1840017	GWD	Gambian Mandinka	AFR	African Ancestry	GWD	1000 Genomes 30x on GRCh38
HG02658	female	SAME1839597	PJL	Punjabi	SAS	South Asian Ancestry	PJL	1000 Genomes on GRCh38,1000 Genomes 30x on GRCh38,1000 Genomes phase 3 release
HG00420	male	SAME122807	CHS	Southern Han Chinese	EAS	East Asian Ancestry	CHS	1000 Genomes 30x on GRCh38,1000 Genomes phase 3 release
HG02643	female	SAME1839785	GWD	Gambian Mandinka	AFR	African Ancestry	GWD	1000 Genomes on GRCh38,1000 Genomes 30x on GRCh38,1000 Genomes phase 3 release,Gambian Genome Variation Project (GRCh38)
HG02660	male	SAME1839672	PJL	Punjabi	SAS	South Asian Ancestry	PJL	1000 Genomes on GRCh38,1000 Genomes 30x on GRCh38,1000 Genomes phase 3 release
HG00437	female	SAME125261	CHS	Southern Han Chinese	EAS	East Asian Ancestry	CHS	1000 Genomes on GRCh38,1000 Genomes 30x on GRCh38,1000 Genomes phase 3 release,1000 Genomes phase 1 release,90 Han Chinese high coverage genomes
HG02648	male	SAME1839751	PJL	Punjabi	SAS	South Asian Ancestry	PJL	1000 Genomes on GRCh38,1000 Genomes 30x on GRCh38,1000 Genomes phase 3 release
HG02677	female	SAME1839483	GWD	Gambian Mandinka	AFR	African Ancestry	GWD	1000 Genomes 30x on GRCh38
HG00444	male	SAME124190	CHS	Southern Han Chinese	EAS	East Asian Ancestry	CHS	1000 Genomes 30x on GRCh38,1000 Genomes phase 3 release
HG02650	male	SAME1839550	PJL	Punjabi	SAS	South Asian Ancestry	PJL	1000 Genomes 30x on GRCh38
HG02684	male	SAME1839292	PJL	Punjabi	SAS	South Asian Ancestry	PJL	1000 Genomes on GRCh38,1000 Genomes 30x on GRCh38,1000 Genomes phase 3 release
HG00449	female	SAME123885	CHS	Southern Han Chinese	EAS	East Asian Ancestry	CHS	1000 Genomes on GRCh38,1000 Genomes 30x on GRCh38,1000 Genomes phase 3 release,1000 Genomes phase 1 release
HG02655	female	SAME1839526	PJL	Punjabi	SAS	South Asian Ancestry	PJL	1000 Genomes on GRCh38,1000 Genomes 30x on GRCh38,1000 Genomes phase 3 release
HG02502	female	SAME1839272	ACB	African Caribbean	AFR	African Ancestry	ACB	1000 Genomes on GRCh38,1000 Genomes 30x on GRCh38,1000 Genomes phase 3 release
HG00451	male	SAME125159	CHS	Southern Han Chinese	EAS	East Asian Ancestry	CHS	1000 Genomes on GRCh38,1000 Genomes 30x on GRCh38,1000 Genomes phase 3 release,1000 Genomes phase 1 release
HG02514	male	SAME1839133	KHV	Kinh Vietnamese	EAS	East Asian Ancestry	KHV	1000 Genomes 30x on GRCh38,1000 Genomes phase 3 release
HG00463	male	SAME123496	CHS	Southern Han Chinese	EAS	East Asian Ancestry	CHS	1000 Genomes on GRCh38,1000 Genomes 30x on GRCh38,1000 Genomes phase 3 release,1000 Genomes phase 1 release
HG02561	male	SAME1839838	GWD	Gambian Mandinka	AFR	African Ancestry	GWD	1000 Genomes on GRCh38,1000 Genomes 30x on GRCh38,1000 Genomes phase 3 release,Gambian Genome Variation Project (GRCh38)
HG02521	male	SAME1840327	KHV	Kinh Vietnamese	EAS	East Asian Ancestry	KHV	1000 Genomes on GRCh38,1000 Genomes 30x on GRCh38,1000 Genomes phase 3 release
HG00639	female	SAME123650	PUR	Puerto Rican	AMR	American Ancestry	PUR	1000 Genomes 30x on GRCh38,1000 Genomes phase 3 release
HG02573	male	SAME1839567	GWD	Gambian Mandinka	AFR	African Ancestry	GWD	1000 Genomes on GRCh38,1000 Genomes 30x on GRCh38,1000 Genomes phase 3 release,Gambian Genome Variation Project (GRCh38)
HG02526	female	SAME1840308	KHV	Kinh Vietnamese	EAS	East Asian Ancestry	KHV	1000 Genomes 30x on GRCh38
HG00641	female	SAME123435	PUR	Puerto Rican	AMR	American Ancestry	PUR	1000 Genomes on GRCh38,1000 Genomes 30x on GRCh38,1000 Genomes phase 3 release,1000 Genomes phase 1 release
HG02580	female	SAME1839366	ACB	African Caribbean	AFR	African Ancestry	ACB	1000 Genomes on GRCh38,1000 Genomes 30x on GRCh38,1000 Genomes phase 3 release
HG02545	male	SAME1839939	ACB	African Caribbean	AFR	African Ancestry	ACB	1000 Genomes on GRCh38,1000 Genomes 30x on GRCh38,1000 Genomes phase 3 release
HG00653	male	SAME123251	CHS	Southern Han Chinese	EAS	East Asian Ancestry	CHS	1000 Genomes on GRCh38,1000 Genomes 30x on GRCh38,1000 Genomes phase 3 release,1000 Genomes phase 1 release,90 Han Chinese high coverage genomes
HG02585	male	SAME1839418	GWD	Gambian Mandinka	AFR	African Ancestry	GWD	1000 Genomes on GRCh38,1000 Genomes 30x on GRCh38,1000 Genomes phase 3 release,Gambian Genome Variation Project (GRCh38)
HG02470	male	SAME1839744	ACB	African Caribbean	AFR	African Ancestry	ACB	1000 Genomes on GRCh38,1000 Genomes 30x on GRCh38,1000 Genomes phase 3 release
HG00658	male	SAME123241	CHS	Southern Han Chinese	EAS	East Asian Ancestry	CHS	1000 Genomes 30x on GRCh38,1000 Genomes phase 3 release
HG02597	male	SAME1839199	PJL	Punjabi	SAS	South Asian Ancestry	PJL	1000 Genomes on GRCh38,1000 Genomes 30x on GRCh38,1000 Genomes phase 3 release
HG02494	female	SAME1839698	PJL	Punjabi,Punjabi	SAS	South Asia (SGDP),South Asian Ancestry	PunjabiSGDP,PJL	1000 Genomes on GRCh38,Simons Genome Diversity Project,1000 Genomes 30x on GRCh38,1000 Genomes phase 3 release
HG00672	female	SAME123606	CHS	Southern Han Chinese	EAS	East Asian Ancestry	CHS	1000 Genomes on GRCh38,1000 Genomes 30x on GRCh38,1000 Genomes phase 3 release,1000 Genomes phase 1 release,90 Han Chinese high coverage genomes
HG02600	male	SAME1840302	PJL	Punjabi	SAS	South Asian Ancestry	PJL	1000 Genomes on GRCh38,1000 Genomes 30x on GRCh38,1000 Genomes phase 3 release
HG02386	male	SAME1840001	CDX	Dai Chinese	EAS	East Asian Ancestry	CDX	1000 Genomes on GRCh38,1000 Genomes 30x on GRCh38,1000 Genomes phase 3 release
HG00684	female	SAME123890	CHS	Southern Han Chinese	EAS	East Asian Ancestry	CHS	1000 Genomes on GRCh38,1000 Genomes 30x on GRCh38,1000 Genomes phase 3 release,1000 Genomes phase 1 release,90 Han Chinese high coverage genomes
HG02662	female	SAME1839662	PJL	Punjabi	SAS	South Asian Ancestry	PJL	1000 Genomes 30x on GRCh38,1000 Genomes phase 3 release
HG02398	male	SAME1839845	CDX	Dai Chinese	EAS	East Asian Ancestry	CDX	1000 Genomes on GRCh38,1000 Genomes 30x on GRCh38,1000 Genomes phase 3 release
HG00689	male	SAME123893	CHS	Southern Han Chinese	EAS	East Asian Ancestry	CHS	1000 Genomes on GRCh38,1000 Genomes 30x on GRCh38,1000 Genomes phase 3 release,1000 Genomes phase 1 release
HG02667	female	SAME1839384	GWD	Gambian Mandinka	AFR	African Ancestry	GWD	1000 Genomes on GRCh38,1000 Genomes 30x on GRCh38,1000 Genomes phase 3 release,Gambian Genome Variation Project (GRCh38)
HG02401	male	SAME1839415	CDX	Dai Chinese	EAS	East Asian Ancestry	CDX	1000 Genomes on GRCh38,1000 Genomes 30x on GRCh38,1000 Genomes phase 3 release
HG00691	male	SAME123161	CHS	Southern Han Chinese	EAS	East Asian Ancestry	CHS	1000 Genomes 30x on GRCh38,1000 Genomes phase 3 release
HG02679	female	SAME1839471	GWD	Gambian Mandinka	AFR	African Ancestry	GWD	1000 Genomes on GRCh38,1000 Genomes 30x on GRCh38,1000 Genomes phase 3 release,Gambian Genome Variation Project (GRCh38)
HG02406	male	SAME1839394	CDX	Dai Chinese	EAS	East Asian Ancestry	CDX	1000 Genomes on GRCh38,1000 Genomes 30x on GRCh38,1000 Genomes phase 3 release
HG00867	female	SAME123550	CDX	Dai Chinese	EAS	East Asian Ancestry	CDX	1000 Genomes on GRCh38,1000 Genomes 30x on GRCh38,1000 Genomes phase 3 release
HG02681	male	SAME1839304	PJL	Punjabi	SAS	South Asian Ancestry	PJL	1000 Genomes on GRCh38,1000 Genomes 30x on GRCh38,1000 Genomes phase 3 release
HG02785	female	SAME1840298	PJL	Punjabi	SAS	South Asian Ancestry	PJL	1000 Genomes 30x on GRCh38,1000 Genomes phase 3 release
HG00879	female	SAME123399	CDX	Dai Chinese	EAS	East Asian Ancestry	CDX	1000 Genomes on GRCh38,1000 Genomes 30x on GRCh38,1000 Genomes phase 3 release

**HG02686**	male	SAME1839277	PJL	Punjabi	SAS	South Asian Ancestry	PJL	1000 Genomes 30x on GRCh38,1000 Genomes phase 3 release
**HG02792**	male	SAME1840416	PJL	Punjabi	SAS	South Asian Ancestry	PJL	1000 Genomes on GRCh38,1000 Genomes 30x on GRCh38,1000 Genomes phase 3 release
**HG00881**	male	SAME123540	CDX	Dai Chinese	EAS	East Asian Ancestry	CDX	1000 Genomes on GRCh38,1000 Genomes 30x on GRCh38,1000 Genomes phase 3 release
**HG02800**	male	SAME1840000	GWD	Gambian Mandinka	AFR	African Ancestry	GWD	1000 Genomes 30x on GRCh38
**HG00956**	female	SAME124319	CDX	Dai Chinese	EAS	East Asian Ancestry	CDX	1000 Genomes on GRCh38,1000 Genomes 30x on GRCh38,1000 Genomes phase 3 release
**HG02427**	female	SAME1839036	ACB	African Caribbean	AFR	African Ancestry	ACB	1000 Genomes on GRCh38,1000 Genomes 30x on GRCh38,1000 Genomes phase 3 release
**HG02805**	female	SAME1840081	GWD	Gambian Mandinka	AFR	African Ancestry	GWD	1000 Genomes on GRCh38,1000 Genomes 30x on GRCh38,1000 Genomes phase 3 release,Gambian Genome Variation Project (GRCh38)
**HG00982**	male	SAME123770	CDX	Dai Chinese	EAS	East Asian Ancestry	CDX	1000 Genomes on GRCh38,1000 Genomes 30x on GRCh38,1000 Genomes phase 3 release
**HG02439**	male	SAME1840297	ACB	African Caribbean	AFR	African Ancestry	ACB	1000 Genomes on GRCh38,1000 Genomes 30x on GRCh38,1000 Genomes phase 3 release
**HG02812**	male	SAME1839846	GWD	Gambian Mandinka	AFR	African Ancestry	GWD	1000 Genomes 30x on GRCh38
**HG01029**	female	SAME123523	CDX	Dai Chinese	EAS	East Asian Ancestry	CDX	1000 Genomes on GRCh38,1000 Genomes 30x on GRCh38,1000 Genomes phase 3 release
**HG02465**	female	SAME1839973	GWD	Gambian Mandinka	AFR	African Ancestry	GWD	1000 Genomes on GRCh38,1000 Genomes 30x on GRCh38,1000 Genomes phase 3 release,Gambian Genome Variation Project (GRCh38)
**HG02817**	female	SAME1839819	GWD	Gambian Mandinka	AFR	African Ancestry	GWD	1000 Genomes on GRCh38,1000 Genomes 30x on GRCh38,1000 Genomes phase 3 release,Gambian Genome Variation Project (GRCh38)
**HG01031**	male	SAME123728	CDX	Dai Chinese	EAS	East Asian Ancestry	CDX	1000 Genomes on GRCh38,1000 Genomes 30x on GRCh38,1000 Genomes phase 3 release
**HG02477**	female	SAME1839710	ACB	African Caribbean	AFR	African Ancestry	ACB	1000 Genomes on GRCh38,1000 Genomes 30x on GRCh38,1000 Genomes phase 3 release
**HG02735**	male	SAME1839866	PJL	Punjabi	SAS	South Asian Ancestry	PJL	1000 Genomes 30x on GRCh38,1000 Genomes phase 3 release
**HG01048**	male	SAME123169	PUR	Puerto Rican	AMR	American Ancestry	PUR	1000 Genomes on GRCh38,1000 Genomes 30x on GRCh38,1000 Genomes phase 3 release,1000 Genomes phase 1 release
**HG02484**	male	SAME1839858	ACB	African Caribbean	AFR	African Ancestry	ACB	1000 Genomes on GRCh38,1000 Genomes 30x on GRCh38,1000 Genomes phase 3 release
**HG02759**	male	SAME1839445	GWD	Gambian Mandinka	AFR	African Ancestry	GWD	1000 Genomes on GRCh38,1000 Genomes 30x on GRCh38,1000 Genomes phase 3 release,Gambian Genome Variation Project (GRCh38)
**HG01050**	female	SAME123180	PUR	Puerto Rican	AMR	American Ancestry	PUR	1000 Genomes 30x on GRCh38,1000 Genomes phase 3 release
**HG02489**	male	SAME1839546	ACB	African Caribbean	AFR	African Ancestry	ACB	1000 Genomes on GRCh38,1000 Genomes 30x on GRCh38,1000 Genomes phase 3 release
**HG02761**	female	SAME1839262	GWD	Gambian Mandinka	AFR	African Ancestry	GWD	1000 Genomes 30x on GRCh38
**HG01055**	female	SAME123638	PUR	Puerto Rican	AMR	American Ancestry	PUR	1000 Genomes on GRCh38,1000 Genomes 30x on GRCh38,1000 Genomes phase 3 release,1000 Genomes phase 1 release
**HG02491**	female	SAME1839617	PJL	Punjabi	SAS	South Asian Ancestry	PJL	1000 Genomes on GRCh38,1000 Genomes 30x on GRCh38,1000 Genomes phase 3 release
**HG02773**	male	SAME1839034	GWD	Gambian Mandinka	AFR	African Ancestry	GWD	1000 Genomes 30x on GRCh38
**HG01062**	female	SAME123014	PUR	Puerto Rican	AMR	American Ancestry	PUR	1000 Genomes 30x on GRCh38,1000 Genomes phase 3 release
**HG02496**	male	SAME1839688	ACB	African Caribbean	AFR	African Ancestry	ACB	1000 Genomes on GRCh38,1000 Genomes 30x on GRCh38,1000 Genomes phase 3 release
**HG02778**	female	SAME1839100	PJL	Punjabi	SAS	South Asian Ancestry	PJL	1000 Genomes on GRCh38,1000 Genomes 30x on GRCh38,1000 Genomes phase 3 release
**HG00704**	male	SAME122928	CHS	Southern Han Chinese	EAS	East Asian Ancestry	CHS	1000 Genomes on GRCh38,1000 Genomes 30x on GRCh38,1000 Genomes phase 3 release,1000 Genomes phase 1 release
**HG02511**	female	SAME1839150	ACB	African Caribbean	AFR	African Ancestry	ACB	1000 Genomes on GRCh38,1000 Genomes 30x on GRCh38,1000 Genomes phase 3 release
**HG02780**	male	SAME1839169	PJL	Punjabi	SAS	South Asian Ancestry	PJL	1000 Genomes on GRCh38,1000 Genomes 30x on GRCh38,1000 Genomes phase 3 release
**HG00709**	male	SAME125334	CHS	Southern Han Chinese	EAS	East Asian Ancestry	CHS	1000 Genomes 30x on GRCh38,1000 Genomes phase 3 release
**HG02523**	female	SAME1840322	KHV	Kinh Vietnamese	EAS	East Asian Ancestry	KHV	1000 Genomes 30x on GRCh38,1000 Genomes phase 3 release
**HG02975**	male	SAME1840232	ESN	Esan	AFR	African Ancestry	ESN	1000 Genomes 30x on GRCh38
**HG02982**	male	SAME1840015	GWD	Gambian Mandinka	AFR	African Ancestry	GWD	1000 Genomes on GRCh38,1000 Genomes 30x on GRCh38,1000 Genomes phase 3 release,Gambian Genome Variation Project (GRCh38)
**HG00728**	male	SAME1840267	CHS	Southern Han Chinese	EAS	East Asian Ancestry	CHS	1000 Genomes on GRCh38,1000 Genomes 30x on GRCh38,1000 Genomes phase 3 release
**HG02554**	male	SAME1840075	ACB	African Caribbean	AFR	African Ancestry	ACB	1000 Genomes on GRCh38,1000 Genomes 30x on GRCh38,1000 Genomes phase 3 release
**HG03012**	male	SAME1839924	BEB	Bengali	SAS	South Asian Ancestry	BEB	1000 Genomes on GRCh38,1000 Genomes 30x on GRCh38,1000 Genomes phase 3 release
**HG03017**	male	SAME1839956	PJL	Punjabi	SAS	South Asian Ancestry	PJL	1000 Genomes 30x on GRCh38
**HG00735**	female	SAME123042	PUR	Puerto Rican	AMR	American Ancestry	PUR	1000 Genomes 30x on GRCh38,1000 Genomes phase 3 release
**HG02390**	male	SAME1839788	CDX	Dai Chinese	EAS	East Asian Ancestry	CDX	1000 Genomes on GRCh38,1000 Genomes 30x on GRCh38,1000 Genomes phase 3 release
**HG02836**	male	SAME1839428	GWD	Gambian Mandinka	AFR	African Ancestry	GWD	1000 Genomes on GRCh38,1000 Genomes 30x on GRCh38,1000 Genomes phase 3 release,Gambian Genome Variation Project (GRCh38)
**HG00742**	male	SAME1840188	PUR	Puerto Rican	AMR	American Ancestry	PUR	1000 Genomes on GRCh38,1000 Genomes 30x on GRCh38,1000 Genomes phase 3 release
**HG02395**	male	SAME1839754	CDX	Dai Chinese	EAS	East Asian Ancestry	CDX	1000 Genomes on GRCh38,1000 Genomes 30x on GRCh38,1000 Genomes phase 3 release
**HG02855**	female	SAME1839330	GWD	Gambian Mandinka	AFR	African Ancestry	GWD	1000 Genomes on GRCh38,1000 Genomes 30x on GRCh38,1000 Genomes phase 3 release,Gambian Genome Variation Project (GRCh38)
**HG00759**	female	SAME125020	CDX	Dai Chinese	EAS	East Asian Ancestry	CDX	1000 Genomes on GRCh38,1000 Genomes 30x on GRCh38,1000 Genomes phase 3 release
**HG02408**	male	SAME1839461	CDX	Dai Chinese	EAS	East Asian Ancestry	CDX	1000 Genomes on GRCh38,1000 Genomes 30x on GRCh38,1000 Genomes phase 3 release
**HG02862**	female	SAME1839123	GWD	Gambian Mandinka	AFR	African Ancestry	GWD	1000 Genomes 30x on GRCh38
**HG00766**	female	SAME124458	CDX	Dai Chinese	EAS	East Asian Ancestry	CDX	1000 Genomes on GRCh38,1000 Genomes 30x on GRCh38,1000 Genomes phase 3 release
**HG02410**	male	SAME1839207	CDX	Dai Chinese	EAS	East Asian Ancestry	CDX	1000 Genomes on GRCh38,1000 Genomes 30x on GRCh38,1000 Genomes phase 3 release
**HG02879**	female	SAME1840355	GWD	Gambian Mandinka	AFR	African Ancestry	GWD	1000 Genomes on GRCh38,1000 Genomes 30x on GRCh38,1000 Genomes phase 3 release,Gambian Genome Variation Project (GRCh38)
**HG00590**	female	SAME125009	CHS	Southern Han Chinese	EAS	East Asian Ancestry	CHS	1000 Genomes on GRCh38,1000 Genomes 30x on GRCh38,1000 Genomes phase 3 release,1000 Genomes phase 1 release
**HG02314**	male	SAME1839674	ACB	African Caribbean	AFR	African Ancestry	ACB	1000 Genomes on GRCh38,1000 Genomes 30x on GRCh38,1000 Genomes phase 3 release
**HG02881**	male	SAME1840178	GWD	Gambian Mandinka	AFR	African Ancestry	GWD	1000 Genomes on GRCh38,1000 Genomes 30x on GRCh38,1000 Genomes phase 3 release,Gambian Genome Variation Project (GRCh38)
**HG00595**	male	SAME125006	CHS	Southern Han Chinese	EAS	East Asian Ancestry	CHS	1000 Genomes on GRCh38,1000 Genomes 30x on GRCh38,1000 Genomes phase 3 release,1000 Genomes phase 1 release
**HG02321**	female	SAME1839446	ACB	African Caribbean	AFR	African Ancestry	ACB	1000 Genomes 30x on GRCh38,1000 Genomes phase 3 release
**HG02886**	female	SAME1840151	GWD	Gambian Mandinka	AFR	African Ancestry	GWD	1000 Genomes 30x on GRCh38
**HG00608**	female	SAME125369	CHS	Southern Han Chinese	EAS	East Asian Ancestry	CHS	1000 Genomes on GRCh38,1000 Genomes 30x on GRCh38,1000 Genomes phase 3 release,1000 Genomes phase 1 release
**HG02345**	female	SAME1839081	PEL	Peruvian	AMR	American Ancestry	PEL	1000 Genomes on GRCh38,1000 Genomes 30x on GRCh38,1000 Genomes phase 3 release
**HG03024**	male	SAME1839799	GWD	Gambian Mandinka	AFR	African Ancestry	GWD	1000 Genomes on GRCh38,1000 Genomes 30x on GRCh38,1000 Genomes phase 3 release,Gambian Genome Variation Project (GRCh38)
**HG00610**	male	SAME125199	CHS	Southern Han Chinese	EAS	East Asian Ancestry	CHS	1000 Genomes on GRCh38,1000 Genomes 30x on GRCh38,1000 Genomes phase 3 release,1000 Genomes phase 1 release
**HG02364**	male	SAME1840125	CDX	Dai Chinese	EAS	East Asian Ancestry	CDX	1000 Genomes on GRCh38,1000 Genomes 30x on GRCh38,1000 Genomes phase 3 release
**HG03029**	male	SAME1839733	GWD	Gambian Mandinka	AFR	African Ancestry	GWD	1000 Genomes 30x on GRCh38
**HG00615**	female	SAME125194	CHS	Southern Han Chinese	EAS	East Asian Ancestry	CHS	1000 Genomes 30x on GRCh38,1000 Genomes phase 3 release
**HG02371**	male	SAME1840180	CDX	Dai Chinese	EAS	East Asian Ancestry	CDX	1000 Genomes on GRCh38,1000 Genomes 30x on GRCh38,1000 Genomes phase 3 release
**HG03048**	male	SAME1839647	GWD	Gambian Mandinka	AFR	African Ancestry	GWD	1000 Genomes on GRCh38,1000 Genomes 30x on GRCh38,1000 Genomes phase 3 release,Gambian Genome Variation Project (GRCh38)
**HG00622**	male	SAME1839278	CHS	Southern Han Chinese	EAS	East Asian Ancestry	CHS	1000 Genomes on GRCh38,1000 Genomes 30x on GRCh38,1000 Genomes phase 3 release
**HG02383**	male	SAME1840019	CDX	Dai Chinese	EAS	East Asian Ancestry	CDX	1000 Genomes on GRCh38,1000 Genomes 30x on GRCh38,1000 Genomes phase 3 release
**HG03050**	male	SAME1839482	GWD	Gambian Mandinka	AFR	African Ancestry	GWD	1000 Genomes 30x on GRCh38
**HG00627**	male	SAME123079	CHS	Southern Han Chinese	EAS	East Asian Ancestry	CHS	1000 Genomes 30x on GRCh38,1000 Genomes phase 3 release
**HG02819**	male	SAME1839804	GWD	Gambian Mandinka	AFR	African Ancestry	GWD	1000 Genomes on GRCh38,1000 Genomes 30x on GRCh38,1000 Genomes phase 3 release,Gambian Genome Variation Project (GRCh38)
**HG03055**	female	SAME1839514	MSL	Mende	AFR	African Ancestry	MSL	1000 Genomes on GRCh38,1000 Genomes 30x on GRCh38,1000 Genomes phase 3 release
**HG00634**	male	SAME122903	CHS	Southern Han Chinese	EAS	East Asian Ancestry	CHS	1000 Genomes on GRCh38,1000 Genomes 30x on GRCh38,1000 Genomes phase 3 release,1000 Genomes phase 1 release
**HG02821**	male	SAME1839588	GWD	Gambian Mandinka	AFR	African Ancestry	GWD	1000 Genomes 30x on GRCh38
**HG03098**	male	SAME1840136	MSL	Mende	AFR	African Ancestry	MSL	1000 Genomes 30x on GRCh38
**HG01170**	male	SAME124623	PUR	Puerto Rican	AMR	American Ancestry	PUR	1000 Genomes on GRCh38,1000 Genomes 30x on GRCh38,1000 Genomes phase 3 release,1000 Genomes phase 1 release
**HG02838**	male	SAME1839417	GWD	Gambian Mandinka	AFR	African Ancestry	GWD	1000 Genomes 30x on GRCh38
**HG03101**	male	SAME1840008	ESN	Esan	AFR	African Ancestry	ESN	1000 Genomes 30x on GRCh38
**HG01175**	female	SAME124618	PUR	Puerto Rican	AMR	American Ancestry	PUR	1000 Genomes 30x on GRCh38,1000 Genomes phase 3 release
**HG02840**	female	SAME1839475	GWD	Gambian Mandinka	AFR	African Ancestry	GWD	1000 Genomes on GRCh38,1000 Genomes 30x on GRCh38,1000 Genomes phase 3 release,Gambian Genome Variation Project (GRCh38)
**HG03113**	female	SAME1839901	ESN	Esan	AFR	African Ancestry	ESN	1000 Genomes 30x on GRCh38
**HG01182**	male	SAME124789	PUR	Puerto Rican	AMR	American Ancestry	PUR	1000 Genomes on GRCh38,1000 Genomes 30x on GRCh38,1000 Genomes phase 3 release
**HG02852**	female	SAME1839346	GWD	Gambian Mandinka	AFR	African Ancestry	GWD	1000 Genomes on GRCh38,1000 Genomes 30x on GRCh38,1000 Genomes phase 3 release,Gambian Genome Variation Project (GRCh38)
**HG03118**	male	SAME1839830	ESN	Esan	AFR	African Ancestry	ESN	1000 Genomes on GRCh38,1000 Genomes 30x on GRCh38,1000 Genomes phase 3 release
**HG01187**	male	SAME124783	PUR	Puerto Rican	AMR	American Ancestry	PUR	1000 Genomes on GRCh38,1000 Genomes 30x on GRCh38,1000 Genomes phase 3 release,1000 Genomes phase 1 release
**HG02869**	male	SAME1839184	GWD	Gambian Mandinka	AFR	African Ancestry	GWD	1000 Genomes on GRCh38,1000 Genomes 30x on GRCh38,Gambian Genome Variation Project (GRCh38)
**HG03120**	male	SAME1839557	ESN	Esan	AFR	African Ancestry	ESN	1000 Genomes on GRCh38,1000 Genomes 30x on GRCh38,1000 Genomes phase 3 release
**HG01199**	male	SAME124366	PUR	Puerto Rican	AMR	American Ancestry	PUR	1000 Genomes 30x on GRCh38,1000 Genomes phase 3 release
**HG02871**	female	SAME1840312	GWD	Gambian Mandinka	AFR	African Ancestry	GWD	1000 Genomes 30x on GRCh38
**HG03125**	female	SAME1839675	ESN	Esan	AFR	African Ancestry	ESN	1000 Genomes 30x on GRCh38,Human Genome Structural Variation Consortium, Phase 2
**HG01252**	male	SAME125283	CLM	Colombian	AMR	American Ancestry	CLM	1000 Genomes 30x on GRCh38,1000 Genomes phase 3 release
**HG02883**	male	SAME1840164	GWD	Gambian Mandinka	AFR	African Ancestry	GWD	1000 Genomes 30x on GRCh38
**HG03132**	female	SAME1839373	ESN	Esan	AFR	African Ancestry	ESN	1000 Genomes on GRCh38,1000 Genomes 30x on GRCh38,1000 Genomes phase 3 release
**HG01257**	female	SAME125288	CLM	Colombian	AMR	American Ancestry	CLM	1000 Genomes on GRCh38,1000 Genomes 30x on GRCh38,1000 Genomes phase 3 release,1000 Genomes phase 1 release
**HG02888**	female	SAME1840139	GWD	Gambian Mandinka	AFR	African Ancestry	GWD	1000 Genomes on GRCh38,1000 Genomes 30x on GRCh38,1000 Genomes phase 3 release,Gambian Genome Variation Project (GRCh38)
**HG03137**	male	SAME1839390	ESN	Esan	AFR	African Ancestry	ESN	1000 Genomes 30x on GRCh38
**HG01067**	female	SAME122997	PUR	Puerto Rican	AMR	American Ancestry	PUR	1000 Genomes on GRCh38,1000 Genomes 30x on GRCh38,1000 Genomes phase 3 release,1000 Genomes phase 1 release
**HG02890**	male	SAME1839947	GWD	Gambian Mandinka	AFR	African Ancestry	GWD	1000 Genomes on GRCh38,1000 Genomes 30x on GRCh38,1000 Genomes phase 3 release,Gambian Genome Variation Project (GRCh38)
**HG01074**	male	SAME124070	PUR	Puerto Rican	AMR	American Ancestry	PUR	1000 Genomes 30x on GRCh38,1000 Genomes phase 3 release
**HG02895**	male	SAME1839918	GWD	Gambian Mandinka	AFR	African Ancestry	GWD	1000 Genomes on GRCh38,1000 Genomes 30x on GRCh38,1000 Genomes phase 3 release,Gambian Genome Variation Project (GRCh38)
**HG03074**	male	SAME1839029	MSL	Mende	AFR	African Ancestry	MSL	1000 Genomes on GRCh38,1000 Genomes 30x on GRCh38,1000 Genomes phase 3 release
**HG01079**	male	SAME123013	PUR	Puerto Rican	AMR	American Ancestry	PUR	1000 Genomes on GRCh38,1000 Genomes 30x on GRCh38,1000 Genomes phase 3 release,1000 Genomes phase 1 release
**HG02922**	female	SAME1839441	ESN	Esan	AFR	African Ancestry	ESN	1000 Genomes on GRCh38,1000 Genomes 30x on GRCh38,1000 Genomes phase 3 release
**HG03079**	female	SAME1839051	MSL	Mende	AFR	African Ancestry	MSL	1000 Genomes on GRCh38,1000 Genomes 30x on GRCh38,1000 Genomes phase 3 release
**HG01081**	female	SAME125395	PUR	Puerto Rican	AMR	American Ancestry	PUR	1000 Genomes 30x on GRCh38,1000 Genomes phase 3 release
**HG02972**	male	SAME1840250	ESN	Esan	AFR	African Ancestry	ESN	1000 Genomes 30x on GRCh38
**HG03081**	male	SAME1840310	MSL	Mende	AFR	African Ancestry	MSL	1000 Genomes on GRCh38,1000 Genomes 30x on GRCh38,1000 Genomes phase 3 release
**HG01086**	female	SAME125394	PUR	Puerto Rican	AMR	American Ancestry	PUR	1000 Genomes on GRCh38,1000 Genomes 30x on GRCh38,1000 Genomes phase 3 release,1000 Genomes phase 1 release
**HG02977**	male	SAME1840221	ESN	Esan	AFR	African Ancestry	ESN	1000 Genomes on GRCh38,1000 Genomes 30x on GRCh38,1000 Genomes phase 3 release
**HG03086**	female	SAME1840331	MSL	Mende	AFR	African Ancestry	MSL	1000 Genomes on GRCh38,1000 Genomes 30x on GRCh38,1000 Genomes phase 3 release
**HG01098**	female	SAME123490	PUR	Puerto Rican	AMR	American Ancestry	PUR	1000 Genomes on GRCh38,1000 Genomes 30x on GRCh38,1000 Genomes phase 3 release,1000 Genomes phase 1 release

**HG02984**	male	SAME1840004	GWD	Gambian Mandinka	AFR	African Ancestry	GWD	1000 Genomes 30x on GRCh38
**HG02944**	male	SAME1839082	ESN	Esan	AFR	African Ancestry	ESN	1000 Genomes on GRCh38,1000 Genomes 30x on GRCh38,1000 Genomes phase 3 release
**HG01101**	male	SAME123677	PUR	Puerto Rican	AMR	American Ancestry	PUR	1000 Genomes on GRCh38,1000 Genomes 30x on GRCh38,1000 Genomes phase 3 release,1000 Genomes phase 1 release
**HG03007**	female	SAME1840245	BEB	Bengali,Bengali	SAS	South Asia (SGDP),South Asian Ancestry	BengaliSGDP,BEB	1000 Genomes on GRCh38,Simons Genome Diversity Project,1000 Genomes 30x on GRCh38,1000 Genomes phase 3 release
**HG02968**	male	SAME1840108	ESN	Esan	AFR	African Ancestry	ESN	1000 Genomes on GRCh38,1000 Genomes 30x on GRCh38,1000 Genomes phase 3 release
**HG01106**	male	SAME123674	PUR	Puerto Rican	AMR	American Ancestry	PUR	1000 Genomes 30x on GRCh38,1000 Genomes phase 3 release
**HG03019**	female	SAME1840060	PJL	Punjabi	SAS	South Asian Ancestry	PJL	1000 Genomes on GRCh38,1000 Genomes 30x on GRCh38,1000 Genomes phase 3 release
**HG02970**	female	SAME1840260	ESN	Esan	AFR	African Ancestry	ESN	1000 Genomes on GRCh38,1000 Genomes 30x on GRCh38,1000 Genomes phase 3 release
**HG01113**	female	SAME123484	CLM	Colombian	AMR	American Ancestry	CLM	1000 Genomes on GRCh38,1000 Genomes 30x on GRCh38,1000 Genomes phase 3 release,1000 Genomes phase 1 release
**HG03021**	male	SAME1839789	PJL	Punjabi	SAS	South Asian Ancestry	PJL	1000 Genomes on GRCh38,1000 Genomes 30x on GRCh38,1000 Genomes phase 3 release
**HG01125**	female	SAME124054	CLM	Colombian	AMR	American Ancestry	CLM	1000 Genomes on GRCh38,1000 Genomes 30x on GRCh38,1000 Genomes phase 3 release,1000 Genomes phase 1 release
**HG03026**	female	SAME1839714	GWD	Gambian Mandinka	AFR	African Ancestry	GWD	1000 Genomes 30x on GRCh38
**HG03461**	female	SAME1839808	MSL	Mende	AFR	African Ancestry	MSL	1000 Genomes on GRCh38,1000 Genomes 30x on GRCh38,1000 Genomes phase 3 release
**HG01137**	female	SAME123888	CLM	Colombian	AMR	American Ancestry	CLM	1000 Genomes on GRCh38,1000 Genomes 30x on GRCh38,1000 Genomes phase 3 release,1000 Genomes phase 1 release
**HG03033**	male	SAME1839566	GWD	Gambian Mandinka	AFR	African Ancestry	GWD	1000 Genomes on GRCh38,1000 Genomes 30x on GRCh38,Gambian Genome Variation Project (GRCh38)
**HG01149**	female	SAME124425	CLM	Colombian	AMR	American Ancestry	CLM	1000 Genomes on GRCh38,1000 Genomes 30x on GRCh38,1000 Genomes phase 3 release,1000 Genomes phase 1 release
**HG03040**	female	SAME1839611	GWD	Gambian Mandinka	AFR	African Ancestry	GWD	1000 Genomes on GRCh38,1000 Genomes 30x on GRCh38,1000 Genomes phase 3 release,Gambian Genome Variation Project (GRCh38)
**HG03473**	female	SAME1839687	MSL	Mende	AFR	African Ancestry	MSL	1000 Genomes on GRCh38,1000 Genomes 30x on GRCh38,1000 Genomes phase 3 release
**HG01168**	female	SAME124796	PUR	Puerto Rican	AMR	American Ancestry	PUR	1000 Genomes on GRCh38,1000 Genomes 30x on GRCh38,1000 Genomes phase 3 release,1000 Genomes phase 1 release
**HG03045**	male	SAME1839635	GWD	Gambian Mandinka	AFR	African Ancestry	GWD	1000 Genomes on GRCh38,1000 Genomes 30x on GRCh38,1000 Genomes phase 3 release,Gambian Genome Variation Project (GRCh38)
**HG03478**	male	SAME1839618	MSL	Mende	AFR	African Ancestry	MSL	1000 Genomes on GRCh38,1000 Genomes 30x on GRCh38,1000 Genomes phase 3 release
**HG03052**	female	SAME1839496	MSL	Mende	AFR	African Ancestry	MSL	1000 Genomes on GRCh38,1000 Genomes 30x on GRCh38,1000 Genomes phase 3 release
**HG03480**	female	SAME1839381	MSL	Mende	AFR	African Ancestry	MSL	1000 Genomes 30x on GRCh38
**HG01334**	male	SAME123533	GBR	British	EUR	European Ancestry	GBR	1000 Genomes on GRCh38,1000 Genomes 30x on GRCh38,1000 Genomes phase 3 release,1000 Genomes phase 1 release,Geuvadis
**HG03057**	male	SAME1839434	MSL	Mende	AFR	African Ancestry	MSL	1000 Genomes on GRCh38,1000 Genomes 30x on GRCh38,1000 Genomes phase 3 release
**HG03485**	female	SAME1839469	MSL	Mende	AFR	African Ancestry	MSL	1000 Genomes on GRCh38,1000 Genomes 30x on GRCh38,1000 Genomes phase 3 release
**HG01341**	male	SAME124859	CLM	Colombian	AMR	American Ancestry	CLM	1000 Genomes on GRCh38,1000 Genomes 30x on GRCh38,1000 Genomes phase 3 release,1000 Genomes phase 1 release
**HG02737**	female	SAME1839857	PJL	Punjabi	SAS	South Asian Ancestry	PJL	1000 Genomes on GRCh38,1000 Genomes 30x on GRCh38,1000 Genomes phase 3 release
**HG03240**	male	SAME1839050	GWD	Gambian Mandinka	AFR	African Ancestry	GWD	1000 Genomes on GRCh38,1000 Genomes 30x on GRCh38,1000 Genomes phase 3 release,Gambian Genome Variation Project (GRCh38)
**HG01346**	female	SAME124860	CLM	Colombian	AMR	American Ancestry	CLM	1000 Genomes 30x on GRCh38,1000 Genomes phase 3 release
**HG02756**	male	SAME1839454	GWD	Gambian Mandinka	AFR	African Ancestry	GWD	1000 Genomes on GRCh38,1000 Genomes 30x on GRCh38,1000 Genomes phase 3 release,Gambian Genome Variation Project (GRCh38)
**HG03269**	male	SAME1840100	ESN	Esan	AFR	African Ancestry	ESN	1000 Genomes 30x on GRCh38
**HG01353**	male	SAME124685	CLM	Colombian	AMR	American Ancestry	CLM	1000 Genomes on GRCh38,1000 Genomes 30x on GRCh38,1000 Genomes phase 3 release,1000 Genomes phase 1 release
**HG02763**	female	SAME1839254	GWD	Gambian Mandinka	AFR	African Ancestry	GWD	1000 Genomes on GRCh38,1000 Genomes 30x on GRCh38,1000 Genomes phase 3 release,Gambian Genome Variation Project (GRCh38)
**HG03271**	male	SAME1840195	ESN	Esan	AFR	African Ancestry	ESN	1000 Genomes on GRCh38,1000 Genomes 30x on GRCh38,1000 Genomes phase 3 release
**HG01358**	male	SAME124688	CLM	Colombian	AMR	American Ancestry	CLM	1000 Genomes 30x on GRCh38,1000 Genomes phase 3 release
**HG02768**	male	SAME1839235	GWD	Gambian Mandinka	AFR	African Ancestry	GWD	1000 Genomes on GRCh38,1000 Genomes 30x on GRCh38,1000 Genomes phase 3 release,Gambian Genome Variation Project (GRCh38)
**HG03295**	male	SAME1839881	ESN	Esan	AFR	African Ancestry	ESN	1000 Genomes on GRCh38,1000 Genomes 30x on GRCh38,1000 Genomes phase 3 release
**HG01360**	female	SAME122855	CLM	Colombian	AMR	American Ancestry	CLM	1000 Genomes on GRCh38,1000 Genomes 30x on GRCh38,1000 Genomes phase 3 release,1000 Genomes phase 1 release
**HG02770**	female	SAME1839049	GWD	Gambian Mandinka	AFR	African Ancestry	GWD	1000 Genomes 30x on GRCh38
**HG03303**	female	SAME1839485	ESN	Esan	AFR	African Ancestry	ESN	1000 Genomes on GRCh38,1000 Genomes 30x on GRCh38,1000 Genomes phase 3 release
**HG01365**	male	SAME124508	CLM	Colombian	AMR	American Ancestry	CLM	1000 Genomes on GRCh38,1000 Genomes 30x on GRCh38,1000 Genomes phase 3 release,1000 Genomes phase 1 release
**HG02775**	female	SAME1839022	PJL	Punjabi	SAS	South Asian Ancestry	PJL	1000 Genomes on GRCh38,1000 Genomes 30x on GRCh38,1000 Genomes phase 3 release
**HG03308**	male	SAME1839515	ESN	Esan	AFR	African Ancestry	ESN	1000 Genomes 30x on GRCh38
**HG01372**	female	SAME1840339	CLM	Colombian	AMR	American Ancestry	CLM	1000 Genomes on GRCh38,1000 Genomes 30x on GRCh38,1000 Genomes phase 3 release
**HG02787**	female	SAME1840288	PJL	Punjabi	SAS	South Asian Ancestry	PJL	1000 Genomes on GRCh38,1000 Genomes 30x on GRCh38,1000 Genomes phase 3 release
**HG03310**	female	SAME1839202	ESN	Esan	AFR	African Ancestry	ESN	1000 Genomes 30x on GRCh38
**HG01377**	male	SAME124288	CLM	Colombian	AMR	American Ancestry	CLM	1000 Genomes on GRCh38,1000 Genomes 30x on GRCh38,1000 Genomes phase 3 release,1000 Genomes phase 1 release
**HG02794**	male	SAME1840410	PJL	Punjabi	SAS	South Asian Ancestry	PJL	1000 Genomes 30x on GRCh38
**HG01529**	male	SAME125330	IBS	Iberian	EUR	European Ancestry	IBS	1000 Genomes 30x on GRCh38,1000 Genomes phase 3 release
**HG02799**	female	SAME1840384	GWD	Gambian Mandinka	AFR	African Ancestry	GWD	1000 Genomes on GRCh38,1000 Genomes 30x on GRCh38,1000 Genomes phase 3 release,Gambian Genome Variation Project (GRCh38)
**HG03163**	male	SAME1839118	ESN	Esan	AFR	African Ancestry	ESN	1000 Genomes on GRCh38,1000 Genomes 30x on GRCh38,1000 Genomes phase 3 release
**HG01531**	female	SAME125136	IBS	Iberian	EUR	European Ancestry	IBS	1000 Genomes on GRCh38,1000 Genomes 30x on GRCh38,1000 Genomes phase 3 release
**HG02807**	male	SAME1840073	GWD	Gambian Mandinka	AFR	African Ancestry	GWD	1000 Genomes on GRCh38,1000 Genomes 30x on GRCh38,1000 Genomes phase 3 release,Gambian Genome Variation Project (GRCh38)
**HG03168**	female	SAME1839143	ESN	Esan	AFR	African Ancestry	ESN	1000 Genomes on GRCh38,1000 Genomes 30x on GRCh38,1000 Genomes phase 3 release
**HG01536**	male	SAME125135	IBS	Iberian	EUR	European Ancestry	IBS	1000 Genomes on GRCh38,1000 Genomes 30x on GRCh38,1000 Genomes phase 3 release
**HG02814**	female	SAME1839837	GWD	Gambian Mandinka	AFR	African Ancestry	GWD	1000 Genomes on GRCh38,1000 Genomes 30x on GRCh38,1000 Genomes phase 3 release,Gambian Genome Variation Project (GRCh38)
**HG03170**	male	SAME1840383	ESN	Esan	AFR	African Ancestry	ESN	1000 Genomes 30x on GRCh38
**HG01550**	male	SAME124009	CLM	Colombian	AMR	American Ancestry	CLM	1000 Genomes on GRCh38,1000 Genomes 30x on GRCh38,1000 Genomes phase 3 release,1000 Genomes phase 1 release
**HG03064**	female	SAME1839228	MSL	Mende	AFR	African Ancestry	MSL	1000 Genomes on GRCh38,1000 Genomes 30x on GRCh38,1000 Genomes phase 3 release
**HG03175**	male	SAME1840407	ESN	Esan	AFR	African Ancestry	ESN	1000 Genomes on GRCh38,1000 Genomes 30x on GRCh38,1000 Genomes phase 3 release
**HG01567**	female	SAME124565	PEL	Peruvian	AMR	American Ancestry	PEL	1000 Genomes 30x on GRCh38,1000 Genomes phase 3 release
**HG03069**	male	SAME1839340	MSL	Mende	AFR	African Ancestry	MSL	1000 Genomes on GRCh38,1000 Genomes 30x on GRCh38,1000 Genomes phase 3 release
**HG01579**	male	SAME124350	PEL	Peruvian	AMR	American Ancestry	PEL	1000 Genomes 30x on GRCh38,1000 Genomes phase 3 release
**HG03199**	male	SAME1839937	ESN	Esan	AFR	African Ancestry	ESN	1000 Genomes on GRCh38,1000 Genomes 30x on GRCh38,1000 Genomes phase 3 release
**HG01586**	male	SAME1839879	PJL	Punjabi	SAS	South Asian Ancestry	PJL	1000 Genomes on GRCh38,1000 Genomes 30x on GRCh38,1000 Genomes phase 3 release
**HG03088**	female	SAME1840338	MSL	Mende	AFR	African Ancestry	MSL	1000 Genomes on GRCh38,1000 Genomes 30x on GRCh38,1000 Genomes phase 3 release
**HG03202**	male	SAME1839602	ESN	Esan	AFR	African Ancestry	ESN	1000 Genomes on GRCh38,1000 Genomes 30x on GRCh38,1000 Genomes phase 3 release
**HG01435**	female	SAME1839239	CLM	Colombian	AMR	American Ancestry	CLM	1000 Genomes on GRCh38,1000 Genomes 30x on GRCh38,1000 Genomes phase 3 release
**HG03095**	female	SAME1840374	MSL	Mende	AFR	African Ancestry	MSL	1000 Genomes on GRCh38,1000 Genomes 30x on GRCh38,1000 Genomes phase 3 release
**HG03238**	female	SAME1839289	PJL	Punjabi	SAS	South Asian Ancestry	PJL	1000 Genomes on GRCh38,1000 Genomes 30x on GRCh38,1000 Genomes phase 3 release
**HG03492**	male	SAME1839196	PJL	Punjabi	SAS	South Asian Ancestry	PJL	1000 Genomes 30x on GRCh38,1000 Genomes phase 3 release
**HG01447**	female	SAME1839099	CLM	Colombian	AMR	American Ancestry	CLM	1000 Genomes on GRCh38,1000 Genomes 30x on GRCh38,1000 Genomes phase 3 release
**HG02941**	male	SAME1839096	ESN	Esan	AFR	African Ancestry	ESN	1000 Genomes on GRCh38,1000 Genomes 30x on GRCh38,1000 Genomes phase 3 release
**HG03517**	female	SAME1840163	ESN	Esan	AFR	African Ancestry	ESN	1000 Genomes on GRCh38,1000 Genomes 30x on GRCh38,1000 Genomes phase 3 release
**HG02946**	female	SAME1839068	ESN	Esan	AFR	African Ancestry	ESN	1000 Genomes on GRCh38,1000 Genomes 30x on GRCh38,1000 Genomes phase 3 release
**HG03428**	female	SAME1840409	MSL	Mende	AFR	African Ancestry	MSL	1000 Genomes on GRCh38,1000 Genomes 30x on GRCh38,1000 Genomes phase 3 release
**HG01459**	female	SAME1840278	CLM	Colombian	AMR	American Ancestry	CLM	1000 Genomes on GRCh38,1000 Genomes 30x on GRCh38,1000 Genomes phase 3 release
**HG02953**	male	SAME1840271	ESN	Esan	AFR	African Ancestry	ESN	1000 Genomes on GRCh38,1000 Genomes 30x on GRCh38,1000 Genomes phase 3 release
**HG03442**	male	SAME1839979	MSL	Mende	AFR	African Ancestry	MSL	1000 Genomes on GRCh38,1000 Genomes 30x on GRCh38,1000 Genomes phase 3 release
**HG01461**	male	SAME122879	CLM	Colombian	AMR	American Ancestry	CLM	1000 Genomes on GRCh38,1000 Genomes 30x on GRCh38,1000 Genomes phase 3 release,1000 Genomes phase 1 release
**HG02965**	male	SAME1840116	ESN	Esan	AFR	African Ancestry	ESN	1000 Genomes on GRCh38,1000 Genomes 30x on GRCh38
**HG03454**	male	SAME1839764	MSL	Mende	AFR	African Ancestry	MSL	1000 Genomes on GRCh38,1000 Genomes 30x on GRCh38
**HG01466**	female	SAME122881	CLM	Colombian	AMR	American Ancestry	CLM	1000 Genomes 30x on GRCh38,1000 Genomes phase 3 release
**HG02698**	male	SAME1839137	PJL	Punjabi	SAS	South Asian Ancestry	PJL	1000 Genomes 30x on GRCh38,1000 Genomes phase 3 release
**HG01593**	female	SAME1839634	PJL	Punjabi	SAS	South Asian Ancestry	PJL	1000 Genomes on GRCh38,1000 Genomes 30x on GRCh38,1000 Genomes phase 3 release
**HG02701**	male	SAME1840152	PJL	Punjabi	SAS	South Asian Ancestry	PJL	1000 Genomes 30x on GRCh38
**HG01598**	female	SAME125239	KHV	Kinh Vietnamese	EAS	East Asian Ancestry	KHV	1000 Genomes on GRCh38,1000 Genomes 30x on GRCh38,1000 Genomes phase 3 release
**HG02725**	female	SAME1839737	PJL	Punjabi	SAS	South Asian Ancestry	PJL	1000 Genomes on GRCh38,1000 Genomes 30x on GRCh38,1000 Genomes phase 3 release
**HG03372**	female	SAME1839716	ESN	Esan	AFR	African Ancestry	ESN	1000 Genomes on GRCh38,1000 Genomes 30x on GRCh38,1000 Genomes phase 3 release
**HG01601**	male	SAME123455	IBS	Iberian	EUR	European Ancestry	IBS	1000 Genomes 30x on GRCh38,1000 Genomes phase 3 release
**HG03103**	male	SAME1840022	ESN	Esan	AFR	African Ancestry	ESN	1000 Genomes on GRCh38,1000 Genomes 30x on GRCh38,1000 Genomes phase 3 release
**HG01606**	male	SAME123458	IBS	Iberian	EUR	European Ancestry	IBS	1000 Genomes on GRCh38,1000 Genomes 30x on GRCh38,1000 Genomes phase 3 release
**HG03108**	female	SAME1839760	ESN	Esan	AFR	African Ancestry	ESN	1000 Genomes on GRCh38,1000 Genomes 30x on GRCh38,1000 Genomes phase 3 release
**HG03391**	male	SAME1839636	MSL	Mende	AFR	African Ancestry	MSL	1000 Genomes on GRCh38,1000 Genomes 30x on GRCh38,1000 Genomes phase 3 release
**HG01613**	female	SAME123626	IBS	Iberian	EUR	European Ancestry	IBS	1000 Genomes on GRCh38,1000 Genomes 30x on GRCh38,1000 Genomes phase 3 release
**HG03110**	female	SAME1839880	ESN	Esan	AFR	African Ancestry	ESN	1000 Genomes 30x on GRCh38
**HG01384**	female	SAME122969	CLM	Colombian	AMR	American Ancestry	CLM	1000 Genomes on GRCh38,1000 Genomes 30x on GRCh38,1000 Genomes phase 3 release,1000 Genomes phase 1 release
**HG03115**	male	SAME1839812	ESN	Esan	AFR	African Ancestry	ESN	1000 Genomes on GRCh38,1000 Genomes 30x on GRCh38,1000 Genomes phase 3 release
**HG03644**	male	SAME1839638	STU	Tamil	SAS	South Asian Ancestry	STU	1000 Genomes on GRCh38,1000 Genomes 30x on GRCh38,1000 Genomes phase 3 release
**HG01389**	male	SAME122972	CLM	Colombian	AMR	American Ancestry	CLM	1000 Genomes on GRCh38,1000 Genomes 30x on GRCh38,1000 Genomes phase 3 release,1000 Genomes phase 1 release
**HG03242**	male	SAME1839058	GWD	Gambian Mandinka	AFR	African Ancestry	GWD	1000 Genomes 30x on GRCh38
**HG03649**	male	SAME1839396	PJL	Punjabi	SAS	South Asian Ancestry	PJL	1000 Genomes on GRCh38,1000 Genomes 30x on GRCh38,1000 Genomes phase 3 release
**HG01391**	female	SAME122804	CLM	Colombian	AMR	American Ancestry	CLM	1000 Genomes 30x on GRCh38,1000 Genomes phase 3 release
**HG03247**	female	SAME1839174	GWD	Gambian Mandinka	AFR	African Ancestry	GWD	1000 Genomes on GRCh38,1000 Genomes 30x on GRCh38,1000 Genomes phase 3 release,Gambian Genome Variation Project (GRCh38)
**HG03651**	male	SAME1839498	PJL	Punjabi	SAS	South Asian Ancestry	PJL	1000 Genomes 30x on GRCh38
**HG01396**	female	SAME1839954	PUR	Puerto Rican	AMR	American Ancestry	PUR	1000 Genomes on GRCh38,1000 Genomes 30x on GRCh38,1000 Genomes phase 3 release
**HG03259**	female	SAME1840303	GWD	Gambian Mandinka	AFR	African Ancestry	GWD	1000 Genomes on GRCh38,1000 Genomes 30x on GRCh38,1000 Genomes phase 3 release,Gambian Genome Variation Project (GRCh38)
**HG03663**	male	SAME1839317	PJL	Punjabi	SAS	South Asian Ancestry	PJL	1000 Genomes on GRCh38,1000 Genomes 30x on GRCh38,1000 Genomes phase 3 release
**HG03280**	male	SAME1840083	ESN	Esan	AFR	African Ancestry	ESN	1000 Genomes on GRCh38,1000 Genomes 30x on GRCh38,1000 Genomes phase 3 release
**HG03668**	female	SAME1839338	PJL	Punjabi	SAS	South Asian Ancestry	PJL	1000 Genomes on GRCh38,1000 Genomes 30x on GRCh38,1000 Genomes phase 3 release

**HG01269**	female	SAME1839257	CLM	Colombian	AMR	American Ancestry	CLM	1000 Genomes on GRCh38,1000 Genomes 30x on GRCh38,1000 Genomes phase 3 release
**HG03196**	male	SAME1839921	ESN	Esan	AFR	African Ancestry	ESN	1000 Genomes on GRCh38,1000 Genomes 30x on GRCh38,1000 Genomes phase 3 release
**HG03719**	female	SAME1839843	ITU	Telugu	SAS	South Asian Ancestry	ITU	1000 Genomes 30x on GRCh38
**HG01271**	male	SAME122998	CLM	Colombian	AMR	American Ancestry	CLM	1000 Genomes on GRCh38,1000 Genomes 30x on GRCh38,1000 Genomes phase 3 release,1000 Genomes phase 1 release
**HG03209**	male	SAME1839551	MSL	Mende	AFR	African Ancestry	MSL	1000 Genomes on GRCh38,1000 Genomes 30x on GRCh38,1000 Genomes phase 3 release
**HG03721**	female	SAME1839659	ITU	Telugu	SAS	South Asian Ancestry	ITU	1000 Genomes on GRCh38,1000 Genomes 30x on GRCh38
**HG01276**	female	SAME122999	CLM	Colombian	AMR	American Ancestry	CLM	1000 Genomes 30x on GRCh38,1000 Genomes phase 3 release
**HG03228**	male	SAME1839232	PJL	Punjabi	SAS	South Asian Ancestry	PJL	1000 Genomes on GRCh38,1000 Genomes 30x on GRCh38,1000 Genomes phase 3 release
**HG03733**	female	SAME1839382	STU	Tamil	SAS	South Asian Ancestry	STU	1000 Genomes on GRCh38,1000 Genomes 30x on GRCh38,1000 Genomes phase 3 release
**HG01303**	female	SAME1840027	PUR	Puerto Rican	AMR	American Ancestry	PUR	1000 Genomes on GRCh38,1000 Genomes 30x on GRCh38,1000 Genomes phase 3 release
**HG03230**	female	SAME1839335	PJL	Punjabi	SAS	South Asian Ancestry	PJL	1000 Genomes 30x on GRCh38
**HG03738**	male	SAME1839473	STU	Tamil	SAS	South Asian Ancestry	STU	1000 Genomes on GRCh38,1000 Genomes 30x on GRCh38,1000 Genomes phase 3 release
**HG01308**	male	SAME1840002	PUR	Puerto Rican	AMR	American Ancestry	PUR	1000 Genomes on GRCh38,1000 Genomes 30x on GRCh38,1000 Genomes phase 3 release
**HG03235**	female	SAME1839360	PJL	Punjabi	SAS	South Asian Ancestry	PJL	1000 Genomes on GRCh38,1000 Genomes 30x on GRCh38,1000 Genomes phase 3 release
**HG03740**	male	SAME1839248	STU	Tamil	SAS	South Asian Ancestry	STU	1000 Genomes on GRCh38,1000 Genomes 30x on GRCh38,1000 Genomes phase 3 release
**HG03432**	male	SAME1840107	MSL	Mende	AFR	African Ancestry	MSL	1000 Genomes on GRCh38,1000 Genomes 30x on GRCh38,1000 Genomes phase 3 release
**HG03745**	male	SAME1839195	STU	Tamil	SAS	South Asian Ancestry	STU	1000 Genomes on GRCh38,1000 Genomes 30x on GRCh38,1000 Genomes phase 3 release
**HG03437**	female	SAME1840123	MSL	Mende	AFR	African Ancestry	MSL	1000 Genomes on GRCh38,1000 Genomes 30x on GRCh38,1000 Genomes phase 3 release
**HG03606**	male	SAME1840248	BEB	Bengali	SAS	South Asian Ancestry	BEB	1000 Genomes on GRCh38,1000 Genomes 30x on GRCh38,1000 Genomes phase 3 release
**HG03449**	female	SAME1839923	MSL	Mende	AFR	African Ancestry	MSL	1000 Genomes on GRCh38,1000 Genomes 30x on GRCh38,1000 Genomes phase 3 release
**HG03618**	male	SAME1840062	PJL	Punjabi	SAS	South Asian Ancestry	PJL	1000 Genomes on GRCh38,1000 Genomes 30x on GRCh38
**HG01485**	male	SAME1840047	CLM	Colombian	AMR	American Ancestry	CLM	1000 Genomes on GRCh38,1000 Genomes 30x on GRCh38,1000 Genomes phase 3 release
**HG03451**	male	SAME1840043	MSL	Mende	AFR	African Ancestry	MSL	1000 Genomes on GRCh38,1000 Genomes 30x on GRCh38,1000 Genomes phase 3 release
**HG03620**	female	SAME1839790	PJL	Punjabi	SAS	South Asian Ancestry	PJL	1000 Genomes 30x on GRCh38
**HG01492**	female	SAME124137	CLM	Colombian	AMR	American Ancestry	CLM	1000 Genomes on GRCh38,1000 Genomes 30x on GRCh38,1000 Genomes phase 3 release,1000 Genomes phase 1 release
**HG03456**	male	SAME1839778	MSL	Mende	AFR	African Ancestry	MSL	1000 Genomes 30x on GRCh38
**HG03625**	female	SAME1839719	PJL	Punjabi	SAS	South Asian Ancestry	PJL	1000 Genomes on GRCh38,1000 Genomes 30x on GRCh38,1000 Genomes phase 3 release
**HG01497**	male	SAME124134	CLM	Colombian	AMR	American Ancestry	CLM	1000 Genomes on GRCh38,1000 Genomes 30x on GRCh38,1000 Genomes phase 3 release,1000 Genomes phase 1 release
**HG03343**	male	SAME1840354	ESN	Esan	AFR	African Ancestry	ESN	1000 Genomes on GRCh38,1000 Genomes 30x on GRCh38,1000 Genomes phase 3 release
**HG03699**	male	SAME1840153	PJL	Punjabi	SAS	South Asian Ancestry	PJL	1000 Genomes on GRCh38,1000 Genomes 30x on GRCh38
**HG01500**	male	SAME124941	IBS	Iberian	EUR	European Ancestry	IBS	1000 Genomes on GRCh38,1000 Genomes 30x on GRCh38,1000 Genomes phase 3 release
**HG03350**	male	SAME1840225	ESN	Esan	AFR	African Ancestry	ESN	1000 Genomes 30x on GRCh38
**HG03702**	male	SAME1840029	PJL	Punjabi	SAS	South Asian Ancestry	PJL	1000 Genomes on GRCh38,1000 Genomes 30x on GRCh38,1000 Genomes phase 3 release
**HG01618**	female	SAME123631	IBS	Iberian	EUR	European Ancestry	IBS	1000 Genomes on GRCh38,1000 Genomes 30x on GRCh38,1000 Genomes phase 3 release,1000 Genomes phase 1 release
**HG03707**	male	SAME1839761	PJL	Punjabi	SAS	South Asian Ancestry	PJL	1000 Genomes 30x on GRCh38
**HG01620**	female	SAME123844	IBS	Iberian	EUR	European Ancestry	IBS	1000 Genomes on GRCh38,1000 Genomes 30x on GRCh38,1000 Genomes phase 3 release,1000 Genomes phase 1 release
**HG03367**	male	SAME1840068	ESN	Esan	AFR	African Ancestry	ESN	1000 Genomes on GRCh38,1000 Genomes 30x on GRCh38,1000 Genomes phase 3 release
**HG03714**	female	SAME1839809	ITU	Telugu	SAS	South Asian Ancestry	ITU	1000 Genomes on GRCh38,1000 Genomes 30x on GRCh38,1000 Genomes phase 3 release
**HG01625**	male	SAME123839	IBS	Iberian	EUR	European Ancestry	IBS	1000 Genomes on GRCh38,1000 Genomes 30x on GRCh38,1000 Genomes phase 3 release,1000 Genomes phase 1 release
**HG03374**	female	SAME1839730	ESN	Esan	AFR	African Ancestry	ESN	1000 Genomes 30x on GRCh38
**HG03752**	female	SAME1839314	STU	Tamil	SAS	South Asian Ancestry	STU	1000 Genomes on GRCh38,1000 Genomes 30x on GRCh38,1000 Genomes phase 3 release
**HG01632**	female	SAME124032	IBS	Iberian	EUR	European Ancestry	IBS	1000 Genomes on GRCh38,1000 Genomes 30x on GRCh38,1000 Genomes phase 3 release
**HG03122**	male	SAME1839657	ESN	Esan	AFR	African Ancestry	ESN	1000 Genomes 30x on GRCh38
**HG03757**	female	SAME1839089	STU	Tamil	SAS	South Asian Ancestry	STU	1000 Genomes on GRCh38,1000 Genomes 30x on GRCh38,1000 Genomes phase 3 release
**HG01668**	female	SAME123111	IBS	Iberian	EUR	European Ancestry	IBS	1000 Genomes on GRCh38,1000 Genomes 30x on GRCh38,1000 Genomes phase 3 release
**HG03127**	male	SAME1839686	ESN	Esan	AFR	African Ancestry	ESN	1000 Genomes on GRCh38,1000 Genomes 30x on GRCh38,1000 Genomes phase 3 release
**HG01670**	female	SAME123290	IBS	Iberian	EUR	European Ancestry	IBS	1000 Genomes on GRCh38,1000 Genomes 30x on GRCh38,1000 Genomes phase 3 release
**HG03134**	male	SAME1839380	ESN	Esan	AFR	African Ancestry	ESN	1000 Genomes 30x on GRCh38
**HG03771**	male	SAME1840393	ITU	Telugu	SAS	South Asian Ancestry	ITU	1000 Genomes on GRCh38,1000 Genomes 30x on GRCh38,1000 Genomes phase 3 release
**HG01675**	male	SAME123287	IBS	Iberian	EUR	European Ancestry	IBS	1000 Genomes on GRCh38,1000 Genomes 30x on GRCh38,1000 Genomes phase 3 release
**HG03139**	male	SAME1839468	ESN	Esan	AFR	African Ancestry	ESN	1000 Genomes on GRCh38,1000 Genomes 30x on GRCh38,1000 Genomes phase 3 release
**HG01505**	male	SAME124946	IBS	Iberian	EUR	European Ancestry	IBS	1000 Genomes 30x on GRCh38,Human Genome Structural Variation Consortium, Phase 2,1000 Genomes phase 3 release
**HG03788**	male	SAME1840227	ITU	Telugu	SAS	South Asian Ancestry	ITU	1000 Genomes on GRCh38,1000 Genomes 30x on GRCh38,1000 Genomes phase 3 release
**HG01512**	male	SAME124769	IBS	Iberian	EUR	European Ancestry	IBS	1000 Genomes on GRCh38,1000 Genomes 30x on GRCh38,1000 Genomes phase 3 release
**HG03297**	female	SAME1839900	ESN	Esan	AFR	African Ancestry	ESN	1000 Genomes on GRCh38,1000 Genomes 30x on GRCh38,1000 Genomes phase 3 release
**HG03790**	male	SAME1839989	ITU	Telugu	SAS	South Asian Ancestry	ITU	1000 Genomes on GRCh38,1000 Genomes 30x on GRCh38,1000 Genomes phase 3 release
**HG01517**	female	SAME124772	IBS	Iberian	EUR	European Ancestry	IBS	1000 Genomes 30x on GRCh38,1000 Genomes phase 3 release
**HG03300**	female	SAME1839467	ESN	Esan	AFR	African Ancestry	ESN	1000 Genomes on GRCh38,1000 Genomes 30x on GRCh38,1000 Genomes phase 3 release
**HG03795**	male	SAME1839925	BEB	Bengali	SAS	South Asian Ancestry	BEB	1000 Genomes 30x on GRCh38
**HG01524**	male	SAME125327	IBS	Iberian	EUR	European Ancestry	IBS	1000 Genomes on GRCh38,1000 Genomes 30x on GRCh38,1000 Genomes phase 3 release
**HG03305**	female	SAME1839497	ESN	Esan	AFR	African Ancestry	ESN	1000 Genomes 30x on GRCh38
**HG03579**	male	SAME1839247	MSL	Mende	AFR	African Ancestry	MSL	1000 Genomes 30x on GRCh38
**HG01815**	female	SAME124524	CDX	Dai Chinese	EAS	East Asian Ancestry	CDX	1000 Genomes on GRCh38,1000 Genomes 30x on GRCh38,1000 Genomes phase 3 release
**HG03312**	female	SAME1839209	ESN	Esan	AFR	African Ancestry	ESN	1000 Genomes on GRCh38,1000 Genomes 30x on GRCh38
**HG03593**	male	SAME1839171	BEB	Bengali	SAS	South Asian Ancestry	BEB	1000 Genomes on GRCh38,1000 Genomes 30x on GRCh38,1000 Genomes phase 3 release
**HG01841**	female	SAME123551	KHV	Kinh Vietnamese	EAS	East Asian Ancestry	KHV	1000 Genomes on GRCh38,1000 Genomes 30x on GRCh38,1000 Genomes phase 3 release
**HG03160**	male	SAME1839192	ESN	Esan	AFR	African Ancestry	ESN	1000 Genomes on GRCh38,1000 Genomes 30x on GRCh38,1000 Genomes phase 3 release
**HG03598**	female	SAME1839194	BEB	Bengali	SAS	South Asian Ancestry	BEB	1000 Genomes on GRCh38,1000 Genomes 30x on GRCh38,1000 Genomes phase 3 release
**HG01846**	male	SAME123558	KHV	Kinh,Kinh Vietnamese	EAS	East Asia (SGDP),East Asian Ancestry	KinhSGDP,KHV	1000 Genomes on GRCh38,Simons Genome Diversity Project,1000 Genomes 30x on GRCh38,1000 Genomes phase 3 release
**HG03172**	male	SAME1840392	ESN	Esan	AFR	African Ancestry	ESN	1000 Genomes on GRCh38,1000 Genomes 30x on GRCh38,1000 Genomes phase 3 release
**HG03548**	female	SAME1839883	MSL	Mende	AFR	African Ancestry	MSL	1000 Genomes on GRCh38,1000 Genomes 30x on GRCh38,1000 Genomes phase 3 release
**HG01853**	female	SAME123751	KHV	Kinh Vietnamese	EAS	East Asian Ancestry	KHV	1000 Genomes on GRCh38,1000 Genomes 30x on GRCh38,1000 Genomes phase 3 release
**HG03189**	female	SAME1840226	ESN	Esan	AFR	African Ancestry	ESN	1000 Genomes on GRCh38,1000 Genomes 30x on GRCh38,1000 Genomes phase 3 release
**HG03567**	female	SAME1839364	MSL	Mende	AFR	African Ancestry	MSL	1000 Genomes on GRCh38,1000 Genomes 30x on GRCh38,1000 Genomes phase 3 release
**HG01858**	female	SAME123754	KHV	Kinh Vietnamese	EAS	East Asian Ancestry	KHV	1000 Genomes on GRCh38,1000 Genomes 30x on GRCh38,1000 Genomes phase 3 release
**HG03191**	male	SAME1839988	ESN	Esan	AFR	African Ancestry	ESN	1000 Genomes 30x on GRCh38
**HG03574**	male	SAME1839229	MSL	Mende	AFR	African Ancestry	MSL	1000 Genomes on GRCh38,1000 Genomes 30x on GRCh38
**HG01860**	male	SAME123201	KHV	Kinh Vietnamese	EAS	East Asian Ancestry	KHV	1000 Genomes on GRCh38,1000 Genomes 30x on GRCh38,1000 Genomes phase 3 release
**HG03682**	male	SAME1840323	STU	Tamil	SAS	South Asian Ancestry	STU	1000 Genomes 30x on GRCh38
**HG03687**	male	SAME1840337	STU	Tamil	SAS	South Asian Ancestry	STU	1000 Genomes on GRCh38,1000 Genomes 30x on GRCh38,1000 Genomes phase 3 release
**HG01771**	male	SAME124637	IBS	Iberian	EUR	European Ancestry	IBS	1000 Genomes on GRCh38,1000 Genomes 30x on GRCh38,1000 Genomes phase 3 release
**HG03694**	male	SAME1840375	STU	Tamil	SAS	South Asian Ancestry	STU	1000 Genomes on GRCh38,1000 Genomes 30x on GRCh38,1000 Genomes phase 3 release
**HG01776**	female	SAME124632	IBS	Iberian	EUR	European Ancestry	IBS	1000 Genomes on GRCh38,1000 Genomes 30x on GRCh38,1000 Genomes phase 3 release
**HG03401**	female	SAME1839299	MSL	Mende	AFR	African Ancestry	MSL	1000 Genomes on GRCh38,1000 Genomes 30x on GRCh38,1000 Genomes phase 3 release
**HG03973**	female	SAME1839728	ITU	Telugu	SAS	South Asian Ancestry	ITU	1000 Genomes on GRCh38,1000 Genomes 30x on GRCh38,1000 Genomes phase 3 release
**HG01783**	male	SAME124427	IBS	Iberian	EUR	European Ancestry	IBS	1000 Genomes on GRCh38,1000 Genomes 30x on GRCh38,1000 Genomes phase 3 release
**HG03978**	male	SAME1839748	ITU	Telugu	SAS	South Asian Ancestry	ITU	1000 Genomes on GRCh38,1000 Genomes 30x on GRCh38,1000 Genomes phase 3 release
**HG01790**	female	SAME124222	GBR	British	EUR	European Ancestry	GBR	1000 Genomes on GRCh38,1000 Genomes 30x on GRCh38,1000 Genomes phase 3 release,Geuvadis
**HG03470**	female	SAME1839670	MSL	Mende	AFR	African Ancestry	MSL	1000 Genomes on GRCh38,1000 Genomes 30x on GRCh38,1000 Genomes phase 3 release
**HG03985**	male	SAME1839601	STU	Tamil	SAS	South Asian Ancestry	STU	1000 Genomes on GRCh38,1000 Genomes 30x on GRCh38,1000 Genomes phase 3 release
**HG01947**	male	SAME124155	PEL	Peruvian	AMR	American Ancestry	PEL	1000 Genomes on GRCh38,1000 Genomes 30x on GRCh38,1000 Genomes phase 3 release
**HG03538**	male	SAME1840007	GWD	Gambian Mandinka	AFR	African Ancestry	GWD	1000 Genomes on GRCh38,1000 Genomes 30x on GRCh38,1000 Genomes phase 3 release,Gambian Genome Variation Project (GRCh38)
**HG03992**	male	SAME1839646	STU	Tamil	SAS	South Asian Ancestry	STU	1000 Genomes 30x on GRCh38
**HG01954**	female	SAME123980	PEL	Peruvian	AMR	American Ancestry	PEL	1000 Genomes on GRCh38,1000 Genomes 30x on GRCh38,1000 Genomes phase 3 release
**HG03540**	female	SAME1839745	GWD	Gambian Mandinka	AFR	African Ancestry	GWD	1000 Genomes 30x on GRCh38
**HG03909**	male	SAME1839438	BEB	Bengali	SAS	South Asian Ancestry	BEB	1000 Genomes 30x on GRCh38
**HG01959**	female	SAME1839343	ACB	African Caribbean	AFR	African Ancestry	ACB	1000 Genomes 30x on GRCh38,1000 Genomes phase 3 release
**HG03557**	female	SAME1839556	MSL	Mende	AFR	African Ancestry	MSL	1000 Genomes on GRCh38,1000 Genomes 30x on GRCh38,1000 Genomes phase 3 release
**HG03911**	male	SAME1839212	BEB	Bengali	SAS	South Asian Ancestry	BEB	1000 Genomes on GRCh38,1000 Genomes 30x on GRCh38,1000 Genomes phase 3 release
**HG01961**	male	SAME1839067	PEL	Peruvian	AMR	American Ancestry	PEL	1000 Genomes on GRCh38,1000 Genomes 30x on GRCh38,1000 Genomes phase 3 release
**HG03916**	female	SAME1839316	BEB	Bengali	SAS	South Asian Ancestry	BEB	1000 Genomes on GRCh38,1000 Genomes 30x on GRCh38,1000 Genomes phase 3 release
**HG01682**	male	SAME124654	IBS	Iberian	EUR	European Ancestry	IBS	1000 Genomes on GRCh38,1000 Genomes 30x on GRCh38,1000 Genomes phase 3 release
**HG03928**	female	SAME1839042	BEB	Bengali	SAS	South Asian Ancestry	BEB	1000 Genomes on GRCh38,1000 Genomes 30x on GRCh38,1000 Genomes phase 3 release
**HG01687**	female	SAME124657	IBS	Iberian	EUR	European Ancestry	IBS	1000 Genomes 30x on GRCh38,1000 Genomes phase 3 release
**HG03571**	male	SAME1839458	MSL	Mende	AFR	African Ancestry	MSL	1000 Genomes on GRCh38,1000 Genomes 30x on GRCh38,1000 Genomes phase 3 release
**HG03884**	female	SAME1840005	STU	Tamil	SAS	South Asian Ancestry	STU	1000 Genomes on GRCh38,1000 Genomes 30x on GRCh38,1000 Genomes phase 3 release
**HG01694**	male	SAME124844	IBS	Iberian	EUR	European Ancestry	IBS	1000 Genomes on GRCh38,1000 Genomes 30x on GRCh38,1000 Genomes phase 3 release
**HG03576**	female	SAME1839238	MSL	Mende	AFR	African Ancestry	MSL	1000 Genomes 30x on GRCh38
**HG03896**	male	SAME1839898	STU	Tamil	SAS	South Asian Ancestry	STU	1000 Genomes on GRCh38,1000 Genomes 30x on GRCh38,1000 Genomes phase 3 release
**HG01699**	male	SAME1839210	IBS	Iberian	EUR	European Ancestry	IBS	1000 Genomes on GRCh38,1000 Genomes 30x on GRCh38,1000 Genomes phase 3 release
**HG03696**	male	SAME1840135	STU	Tamil	SAS	South Asian Ancestry	STU	1000 Genomes on GRCh38,1000 Genomes 30x on GRCh38,1000 Genomes phase 3 release

**HG03904**	female	SAME1839501	BEB	Bengali	SAS	South Asian Ancestry	BEB	1000 Genomes on GRCh38,1000 Genomes 30x on GRCh38
**HG01702**	female	SAME123259	IBS	Iberian	EUR	European Ancestry	IBS	1000 Genomes on GRCh38,1000 Genomes 30x on GRCh38,1000 Genomes phase 3 release
**HG03704**	female	SAME1840040	PJL	Punjabi	SAS	South Asian Ancestry	PJL	1000 Genomes 30x on GRCh38
**HG03803**	male	SAME1839527	BEB	Bengali	SAS	South Asian Ancestry	BEB	1000 Genomes on GRCh38,1000 Genomes 30x on GRCh38,1000 Genomes phase 3 release
**HG01865**	male	SAME123196	KHV	Kinh Vietnamese	EAS	East Asian Ancestry	KHV	1000 Genomes on GRCh38,1000 Genomes 30x on GRCh38,1000 Genomes phase 3 release
**HG03709**	female	SAME1839780	PJL	Punjabi	SAS	South Asian Ancestry	PJL	1000 Genomes on GRCh38,1000 Genomes 30x on GRCh38,1000 Genomes phase 3 release
**HG03808**	female	SAME1839552	BEB	Bengali	SAS	South Asian Ancestry	BEB	1000 Genomes on GRCh38,1000 Genomes 30x on GRCh38,1000 Genomes phase 3 release
**HG01872**	male	SAME123995	KHV	Kinh Vietnamese	EAS	East Asian Ancestry	KHV	1000 Genomes on GRCh38,1000 Genomes 30x on GRCh38,1000 Genomes phase 3 release
**HG03711**	male	SAME1839894	STU	Tamil	SAS	South Asian Ancestry	STU	1000 Genomes on GRCh38,1000 Genomes 30x on GRCh38,1000 Genomes phase 3 release
**HG03815**	male	SAME1839419	BEB	Bengali	SAS	South Asian Ancestry	BEB	1000 Genomes on GRCh38,1000 Genomes 30x on GRCh38,1000 Genomes phase 3 release
**HG01884**	female	SAME122845	ACB	African Caribbean	AFR	African Ancestry	ACB	1000 Genomes 30x on GRCh38,1000 Genomes phase 3 release
**HG03716**	male	SAME1839825	ITU	Telugu	SAS	South Asian Ancestry	ITU	1000 Genomes on GRCh38,1000 Genomes 30x on GRCh38,1000 Genomes phase 3 release
**HG01923**	male	SAME123788	PEL	Peruvian	AMR	American Ancestry	PEL	1000 Genomes on GRCh38,1000 Genomes 30x on GRCh38,1000 Genomes phase 3 release
**HG03487**	male	SAME1839480	PJL	Punjabi	SAS	South Asian Ancestry	PJL	1000 Genomes on GRCh38,1000 Genomes 30x on GRCh38
**HG03930**	female	SAME1839156	BEB	Bengali	SAS	South Asian Ancestry	BEB	1000 Genomes 30x on GRCh38
**HG01928**	male	SAME123782	PEL	Peruvian	AMR	American Ancestry	PEL	1000 Genomes 30x on GRCh38,1000 Genomes phase 3 release
**HG03499**	female	SAME1839219	ESN	Esan	AFR	African Ancestry	ESN	1000 Genomes on GRCh38,1000 Genomes 30x on GRCh38,1000 Genomes phase 3 release
**HG03942**	male	SAME1840356	BEB	Bengali	SAS	South Asian Ancestry	BEB	1000 Genomes 30x on GRCh38
**HG01935**	male	SAME123588	PEL	Peruvian	AMR	American Ancestry	PEL	1000 Genomes on GRCh38,1000 Genomes 30x on GRCh38,1000 Genomes phase 3 release
**HG03514**	female	SAME1840150	ESN	Esan	AFR	African Ancestry	ESN	1000 Genomes on GRCh38,1000 Genomes 30x on GRCh38,1000 Genomes phase 3 release
**HG03947**	female	SAME1840378	STU	Tamil	SAS	South Asian Ancestry	STU	1000 Genomes on GRCh38,1000 Genomes 30x on GRCh38,1000 Genomes phase 3 release
**HG01942**	female	SAME124150	PEL	Peruvian	AMR	American Ancestry	PEL	1000 Genomes on GRCh38,1000 Genomes 30x on GRCh38,1000 Genomes phase 3 release
**HG03519**	male	SAME1840176	ESN	Esan	AFR	African Ancestry	ESN	1000 Genomes 30x on GRCh38
**HG03834**	female	SAME1839269	BEB	Bengali	SAS	South Asian Ancestry	BEB	1000 Genomes 30x on GRCh38
**HG01889**	female	SAME122851	ACB	African Caribbean	AFR	African Ancestry	ACB	1000 Genomes on GRCh38,1000 Genomes 30x on GRCh38,1000 Genomes phase 3 release
**HG03521**	male	SAME1840190	ESN	Esan	AFR	African Ancestry	ESN	1000 Genomes on GRCh38,1000 Genomes 30x on GRCh38,1000 Genomes phase 3 release
**HG03846**	male	SAME1839172	STU	Tamil	SAS	South Asian Ancestry	STU	1000 Genomes on GRCh38,1000 Genomes 30x on GRCh38,1000 Genomes phase 3 release
**HG01891**	female	SAME123029	ACB	African Caribbean	AFR	African Ancestry	ACB	1000 Genomes 30x on GRCh38,1000 Genomes phase 3 release
**HG03639**	male	SAME1839603	PJL	Punjabi	SAS	South Asian Ancestry	PJL	1000 Genomes on GRCh38,1000 Genomes 30x on GRCh38
**HG04003**	male	SAME1839775	STU	Tamil	SAS	South Asian Ancestry	STU	1000 Genomes on GRCh38,1000 Genomes 30x on GRCh38,1000 Genomes phase 3 release
**HG01896**	female	SAME1840348	ACB	African Caribbean	AFR	African Ancestry	ACB	1000 Genomes on GRCh38,1000 Genomes 30x on GRCh38,1000 Genomes phase 3 release
**HG03641**	male	SAME1839626	PJL	Punjabi	SAS	South Asian Ancestry	PJL	1000 Genomes 30x on GRCh38
**HG04015**	male	SAME1839847	ITU	Telugu	SAS	South Asian Ancestry	ITU	1000 Genomes on GRCh38,1000 Genomes 30x on GRCh38,1000 Genomes phase 3 release
**HG01916**	female	SAME1839853	ACB	African Caribbean	AFR	African Ancestry	ACB	1000 Genomes 30x on GRCh38,1000 Genomes phase 3 release
**HG03646**	male	SAME1839643	STU	Tamil	SAS	South Asian Ancestry	STU	1000 Genomes on GRCh38,1000 Genomes 30x on GRCh38,1000 Genomes phase 3 release
**HG04022**	male	SAME1839691	ITU	Telugu	SAS	South Asian Ancestry	ITU	1000 Genomes on GRCh38,1000 Genomes 30x on GRCh38,1000 Genomes phase 3 release
**HG01707**	female	SAME123264	IBS	Iberian	EUR	European Ancestry	IBS	1000 Genomes on GRCh38,1000 Genomes 30x on GRCh38,1000 Genomes phase 3 release
**HG03653**	female	SAME1839508	PJL	Punjabi	SAS	South Asian Ancestry	PJL	1000 Genomes on GRCh38,1000 Genomes 30x on GRCh38,1000 Genomes phase 3 release
**HG04039**	male	SAME1839506	STU	Tamil	SAS	South Asian Ancestry	STU	1000 Genomes on GRCh38,1000 Genomes 30x on GRCh38,1000 Genomes phase 3 release
**HG01757**	female	SAME124987	IBS	Iberian	EUR	European Ancestry	IBS	1000 Genomes on GRCh38,1000 Genomes 30x on GRCh38,1000 Genomes phase 3 release
**HG03660**	male	SAME1839218	PJL	Punjabi	SAS	South Asian Ancestry	PJL	1000 Genomes on GRCh38,1000 Genomes 30x on GRCh38,1000 Genomes phase 3 release
**HG03754**	female	SAME1839073	STU	Tamil	SAS	South Asian Ancestry	STU	1000 Genomes on GRCh38,1000 Genomes 30x on GRCh38,1000 Genomes phase 3 release
**HG01795**	female	SAME124217	CDX	Dai Chinese	EAS	East Asian Ancestry	CDX	1000 Genomes on GRCh38,1000 Genomes 30x on GRCh38,1000 Genomes phase 3 release
**HG03761**	male	SAME1839114	PJL	Punjabi	SAS	South Asian Ancestry	PJL	1000 Genomes on GRCh38,1000 Genomes 30x on GRCh38
**HG04060**	male	SAME1839126	ITU	Telugu	SAS	South Asian Ancestry	ITU	1000 Genomes on GRCh38,1000 Genomes 30x on GRCh38,1000 Genomes phase 3 release
**HG01808**	female	SAME124300	CDX	Dai Chinese	EAS	East Asian Ancestry	CDX	1000 Genomes on GRCh38,1000 Genomes 30x on GRCh38,1000 Genomes phase 3 release
**HG03858**	female	SAME1840301	STU	Tamil	SAS	South Asian Ancestry	STU	1000 Genomes on GRCh38,1000 Genomes 30x on GRCh38,1000 Genomes phase 3 release
**HG01810**	male	SAME124519	CDX	Dai Chinese	EAS	East Asian Ancestry	CDX	1000 Genomes on GRCh38,1000 Genomes 30x on GRCh38,1000 Genomes phase 3 release
**HG03773**	male	SAME1840406	ITU	Telugu	SAS	South Asian Ancestry	ITU	1000 Genomes on GRCh38,1000 Genomes 30x on GRCh38,1000 Genomes phase 3 release
**HG03872**	male	SAME1840202	ITU	Telugu	SAS	South Asian Ancestry	ITU	1000 Genomes on GRCh38,1000 Genomes 30x on GRCh38,1000 Genomes phase 3 release
**HG01992**	female	SAME124742	PEL	Peruvian	AMR	American Ancestry	PEL	1000 Genomes on GRCh38,1000 Genomes 30x on GRCh38,1000 Genomes phase 3 release
**HG03583**	female	SAME1839283	MSL	Mende	AFR	African Ancestry	MSL	1000 Genomes on GRCh38,1000 Genomes 30x on GRCh38,1000 Genomes phase 3 release
**NA12146**	male	SAME124158	CEU	CEPH	EUR	European Ancestry	CEU	1000 Genomes 30x on GRCh38,1000 Genomes phase 3 release
**HG01997**	female	SAME124740	PEL	Peruvian	AMR	American Ancestry	PEL	1000 Genomes on GRCh38,1000 Genomes 30x on GRCh38,1000 Genomes phase 3 release
**NA12273**	female	SAME123256	CEU	CEPH	EUR	European Ancestry	CEU	1000 Genomes on GRCh38,1000 Genomes 30x on GRCh38,1000 Genomes phase 3 release,1000 Genomes phase 1 release,Geuvadis
**HG02003**	female	SAME123619	PEL	Peruvian	AMR	American Ancestry	PEL	1000 Genomes on GRCh38,1000 Genomes 30x on GRCh38,1000 Genomes phase 3 release
**HG03595**	female	SAME1839185	BEB	Bengali	SAS	South Asian Ancestry	BEB	1000 Genomes on GRCh38,1000 Genomes 30x on GRCh38,1000 Genomes phase 3 release
**NA12329**	female	SAME123531	CEU	CEPH	EUR	European Ancestry	CEU	1000 Genomes 30x on GRCh38,Human Genome Structural Variation Consortium, Phase 2,1000 Genomes phase 3 release
**HG02008**	male	SAME123621	PEL	Peruvian	AMR	American Ancestry	PEL	1000 Genomes on GRCh38,1000 Genomes 30x on GRCh38,1000 Genomes phase 3 release
**HG03603**	male	SAME1840229	BEB	Bengali	SAS	South Asian Ancestry	BEB	1000 Genomes on GRCh38,1000 Genomes 30x on GRCh38,1000 Genomes phase 3 release
**NA12336**	female	SAME125068	CEU	CEPH	EUR	European Ancestry	CEU	1000 Genomes 30x on GRCh38,1000 Genomes phase 3 release
**HG02010**	female	SAME123444	ACB	African Caribbean	AFR	African Ancestry	ACB	1000 Genomes on GRCh38,1000 Genomes 30x on GRCh38,1000 Genomes phase 3 release
**HG03672**	male	SAME1839020	STU	Tamil	SAS	South Asian Ancestry	STU	1000 Genomes on GRCh38,1000 Genomes 30x on GRCh38,1000 Genomes phase 3 release
**NA12343**	female	SAME125156	CEU	CEPH	EUR	European Ancestry	CEU	1000 Genomes 30x on GRCh38,1000 Genomes phase 3 release
**HG02015**	male	SAME123447	KHV	Kinh Vietnamese	EAS	East Asian Ancestry	KHV	1000 Genomes 30x on GRCh38,1000 Genomes phase 3 release
**HG03684**	female	SAME1840329	STU	Tamil	SAS	South Asian Ancestry	STU	1000 Genomes on GRCh38,1000 Genomes 30x on GRCh38,1000 Genomes phase 3 release
**NA12348**	female	SAME125163	CEU	CEPH	EUR	European Ancestry	CEU	1000 Genomes on GRCh38,1000 Genomes 30x on GRCh38,1000 Genomes phase 3 release,1000 Genomes phase 1 release,Geuvadis
**HG02077**	male	SAME123108	KHV	Kinh Vietnamese	EAS	East Asian Ancestry	KHV	1000 Genomes 30x on GRCh38,1000 Genomes phase 3 release
**HG03689**	female	SAME1840268	STU	Tamil	SAS	South Asian Ancestry	STU	1000 Genomes on GRCh38,1000 Genomes 30x on GRCh38,1000 Genomes phase 3 release
**NA12386**	female	SAME124386	CEU	CEPH	EUR	European Ancestry	CEU	1000 Genomes 30x on GRCh38,1000 Genomes phase 3 release
**HG02084**	female	SAME124973	KHV	Kinh Vietnamese	EAS	East Asian Ancestry	KHV	1000 Genomes on GRCh38,1000 Genomes 30x on GRCh38,1000 Genomes phase 3 release
**HG03691**	male	SAME1840366	STU	Tamil	SAS	South Asian Ancestry	STU	1000 Genomes on GRCh38,1000 Genomes 30x on GRCh38,1000 Genomes phase 3 release
**NA12413**	male	SAME124055	CEU	CEPH	EUR	European Ancestry	CEU	1000 Genomes on GRCh38,1000 Genomes 30x on GRCh38,1000 Genomes phase 3 release,1000 Genomes phase 1 release,Geuvadis
**HG02089**	female	SAME124821	PEL	Peruvian	AMR	American Ancestry	PEL	1000 Genomes on GRCh38,1000 Genomes 30x on GRCh38,1000 Genomes phase 3 release
**HG03615**	male	SAME1839951	BEB	Bengali	SAS	South Asian Ancestry	BEB	1000 Genomes on GRCh38,1000 Genomes 30x on GRCh38,1000 Genomes phase 3 release
**NA12716**	male	SAME123912	CEU	CEPH	EUR	European Ancestry	CEU	1000 Genomes on GRCh38,1000 Genomes 30x on GRCh38,1000 Genomes phase 3 release,1000 Genomes phase 1 release,Geuvadis
**HG02091**	female	SAME124650	PEL	Peruvian	AMR	American Ancestry	PEL	1000 Genomes 30x on GRCh38,1000 Genomes phase 3 release
**HG03634**	female	SAME1839575	PJL	Punjabi	SAS	South Asian Ancestry	PJL	1000 Genomes on GRCh38,1000 Genomes 30x on GRCh38,1000 Genomes phase 3 release
**NA12761**	female	SAME124466	CEU	CEPH	EUR	European Ancestry	CEU	1000 Genomes on GRCh38,1000 Genomes 30x on GRCh38,1000 Genomes phase 3 release,1000 Genomes phase 1 release,Geuvadis
**HG02104**	male	SAME123874	PEL	Peruvian	AMR	American Ancestry	PEL	1000 Genomes on GRCh38,1000 Genomes 30x on GRCh38,1000 Genomes phase 3 release
**NA12766**	male	SAME124471	CEU	CEPH	EUR	European Ancestry	CEU	1000 Genomes 30x on GRCh38,1000 Genomes phase 3 release
**HG02111**	female	SAME1839922	ACB	African Caribbean	AFR	African Ancestry	ACB	1000 Genomes on GRCh38,1000 Genomes 30x on GRCh38,1000 Genomes phase 3 release
**HG03730**	female	SAME1839369	ITU	Telugu	SAS	South Asian Ancestry	ITU	1000 Genomes on GRCh38,1000 Genomes 30x on GRCh38,1000 Genomes phase 3 release
**NA12778**	female	SAME124249	CEU	CEPH	EUR	European Ancestry	CEU	1000 Genomes on GRCh38,1000 Genomes 30x on GRCh38,1000 Genomes phase 3 release,1000 Genomes phase 1 release,Geuvadis
**HG02116**	male	SAME1839990	KHV	Kinh Vietnamese	EAS	East Asian Ancestry	KHV	1000 Genomes on GRCh38,1000 Genomes 30x on GRCh38,1000 Genomes phase 3 release
**HG03742**	male	SAME1839255	ITU	Telugu	SAS	South Asian Ancestry	ITU	1000 Genomes on GRCh38,1000 Genomes 30x on GRCh38,1000 Genomes phase 3 release
**NA12812**	male	SAME123023	CEU	CEPH	EUR	European Ancestry	CEU	1000 Genomes on GRCh38,1000 Genomes 30x on GRCh38,1000 Genomes phase 3 release,1000 Genomes phase 1 release,Geuvadis
**HG02128**	male	SAME123475	KHV	Kinh Vietnamese	EAS	East Asian Ancestry	KHV	1000 Genomes on GRCh38,1000 Genomes 30x on GRCh38,1000 Genomes phase 3 release
**HG03999**	male	SAME1839416	STU	Tamil	SAS	South Asian Ancestry	STU	1000 Genomes on GRCh38,1000 Genomes 30x on GRCh38,1000 Genomes phase 3 release
**NA12817**	male	SAME123020	CEU	CEPH	EUR	European Ancestry	CEU	1000 Genomes 30x on GRCh38,1000 Genomes phase 3 release
**HG02130**	female	SAME1839904	KHV	Kinh Vietnamese	EAS	East Asian Ancestry	KHV	1000 Genomes on GRCh38,1000 Genomes 30x on GRCh38,1000 Genomes phase 3 release
**HG04017**	male	SAME1839851	ITU	Telugu	SAS	South Asian Ancestry	ITU	1000 Genomes on GRCh38,1000 Genomes 30x on GRCh38,1000 Genomes phase 3 release
**NA12829**	male	SAME125075	CEU	CEPH	EUR	European Ancestry	CEU	1000 Genomes on GRCh38,1000 Genomes 30x on GRCh38,1000 Genomes phase 3 release,1000 Genomes phase 1 release,Geuvadis
**HG02135**	male	SAME1839872	KHV	Kinh Vietnamese	EAS	East Asian Ancestry	KHV	1000 Genomes 30x on GRCh38,1000 Genomes phase 3 release
**NA12843**	female	SAME123541	CEU	CEPH	EUR	European Ancestry	CEU	1000 Genomes on GRCh38,1000 Genomes 30x on GRCh38,1000 Genomes phase 3 release,1000 Genomes phase 1 release,Geuvadis
**HG02166**	female	SAME124207	CDX	Dai Chinese	EAS	East Asian Ancestry	CDX	1000 Genomes on GRCh38,1000 Genomes 30x on GRCh38,1000 Genomes phase 3 release
**HG04029**	female	SAME1839630	STU	Tamil	SAS	South Asian Ancestry	STU	1000 Genomes on GRCh38,1000 Genomes 30x on GRCh38,1000 Genomes phase 3 release
**NA12874**	male	SAME123389	CEU	CEPH	EUR	European Ancestry	CEU	1000 Genomes on GRCh38,1000 Genomes 30x on GRCh38,1000 Genomes phase 3 release,1000 Genomes phase 1 release,Geuvadis
**HG04036**	female	SAME1839492	STU	Tamil	SAS	South Asian Ancestry	STU	1000 Genomes 30x on GRCh38
**NA18485**	male	SAME124100	YRI	Yoruba	AFR	African Ancestry	YRI	1000 Genomes 30x on GRCh38,1000 Genomes phase 3 release
**HG02178**	female	SAME124414	CDX	Dai Chinese	EAS	East Asian Ancestry	CDX	1000 Genomes on GRCh38,1000 Genomes 30x on GRCh38,1000 Genomes phase 3 release
**HG04229**	male	SAME1839165	STU	Tamil	SAS	South Asian Ancestry	STU	1000 Genomes on GRCh38,1000 Genomes 30x on GRCh38,1000 Genomes phase 3 release
**HG02180**	female	SAME123208	CDX	Dai Chinese	EAS	East Asian Ancestry	CDX	1000 Genomes on GRCh38,1000 Genomes 30x on GRCh38,1000 Genomes phase 3 release
**HG03886**	female	SAME1840021	STU	Tamil	SAS	South Asian Ancestry	STU	1000 Genomes on GRCh38,1000 Genomes 30x on GRCh38,1000 Genomes phase 3 release
**HG02185**	female	SAME123215	CDX	Dai Chinese	EAS	East Asian Ancestry	CDX	1000 Genomes on GRCh38,1000 Genomes 30x on GRCh38,1000 Genomes phase 3 release
**HG03898**	female	SAME1839806	STU	Tamil	SAS	South Asian Ancestry	STU	1000 Genomes on GRCh38,1000 Genomes 30x on GRCh38,1000 Genomes phase 3 release
**NA06985**	female	SAME124762	CEU	CEPH	EUR	European Ancestry	CEU	1000 Genomes on GRCh38,1000 Genomes 30x on GRCh38,1000 Genomes phase 3 release,1000 Genomes phase 1 release,Geuvadis
**NA06997**	female	SAME124930	CEU	CEPH	EUR	European Ancestry	CEU	1000 Genomes 30x on GRCh38,1000 Genomes phase 3 release
**HG02224**	male	SAME1839579	IBS	Iberian	EUR	European Ancestry	IBS	1000 Genomes on GRCh38,1000 Genomes 30x on GRCh38,1000 Genomes phase 3 release
**HG03848**	male	SAME1839187	STU	Tamil	SAS	South Asian Ancestry	STU	1000 Genomes on GRCh38,1000 Genomes 30x on GRCh38,1000 Genomes phase 3 release
**NA07022**	male	SAME125336	CEU	CEPH	EUR	European Ancestry	CEU	1000 Genomes 30x on GRCh38,1000 Genomes phase 3 release

**HG02229**	female	SAME1839649	IBS	Iberian	EUR	European Ancestry	IBS	1000 Genomes 30x on GRCh38,1000 Genomes phase 3 release
**HG03850**	male	SAME1840270	STU	Tamil	SAS	South Asian Ancestry	STU	1000 Genomes on GRCh38,1000 Genomes 30x on GRCh38,1000 Genomes phase 3 release
**NA07034**	male	SAME122863	CEU	CEPH	EUR	European Ancestry	CEU	1000 Genomes 30x on GRCh38,1000 Genomes phase 3 release
**HG02231**	male	SAME1839376	IBS	Iberian	EUR	European Ancestry	IBS	1000 Genomes on GRCh38,1000 Genomes 30x on GRCh38,1000 Genomes phase 3 release
**HG03862**	female	SAME1840158	ITU	Telugu	SAS	South Asian Ancestry	ITU	1000 Genomes on GRCh38,1000 Genomes 30x on GRCh38,1000 Genomes phase 3 release
**NA07349**	male	SAME123568	CEU	CEPH	EUR	European Ancestry	CEU	1000 Genomes 30x on GRCh38,1000 Genomes phase 3 release
**HG02236**	male	SAME123520	IBS	Iberian	EUR	European Ancestry	IBS	1000 Genomes on GRCh38,1000 Genomes 30x on GRCh38,1000 Genomes phase 3 release
**HG03867**	male	SAME1840095	ITU	Telugu	SAS	South Asian Ancestry	ITU	1000 Genomes on GRCh38,1000 Genomes 30x on GRCh38,1000 Genomes phase 3 release
**NA10830**	male	SAME124548	CEU	CEPH	EUR	European Ancestry	CEU	1000 Genomes 30x on GRCh38,1000 Genomes phase 3 release
**HG02250**	male	SAME1839268	CDX	Dai Chinese	EAS	East Asian Ancestry	CDX	1000 Genomes on GRCh38,1000 Genomes 30x on GRCh38,1000 Genomes phase 3 release
**HG03874**	female	SAME1839961	ITU	Telugu	SAS	South Asian Ancestry	ITU	1000 Genomes on GRCh38,1000 Genomes 30x on GRCh38,1000 Genomes phase 3 release
**NA10835**	male	SAME124547	CEU	CEPH	EUR	European Ancestry	CEU	1000 Genomes 30x on GRCh38,1000 Genomes phase 3 release
**HG02142**	female	SAME124614	KHV	Kinh Vietnamese	EAS	East Asian Ancestry	KHV	1000 Genomes on GRCh38,1000 Genomes 30x on GRCh38,1000 Genomes phase 3 release
**HG03824**	male	SAME1839459	BEB	Bengali	SAS	South Asian Ancestry	BEB	1000 Genomes on GRCh38,1000 Genomes 30x on GRCh38,1000 Genomes phase 3 release
**NA10842**	male	SAME124755	CEU	CEPH	EUR	European Ancestry	CEU	1000 Genomes 30x on GRCh38,1000 Genomes phase 3 release
**HG02147**	female	SAME1839700	PEL	Peruvian	AMR	American Ancestry	PEL	1000 Genomes on GRCh38,1000 Genomes 30x on GRCh38,1000 Genomes phase 3 release
**HG03829**	female	SAME1839241	BEB	Bengali	SAS	South Asian Ancestry	BEB	1000 Genomes on GRCh38,1000 Genomes 30x on GRCh38,1000 Genomes phase 3 release
**NA10847**	female	SAME124757	CEU	CEPH	EUR	European Ancestry	CEU	1000 Genomes on GRCh38,1000 Genomes 30x on GRCh38,1000 Genomes phase 3 release,1000 Genomes phase 1 release,Geuvadis
**HG02154**	female	SAME124794	CDX	Dai Chinese	EAS	East Asian Ancestry	CDX	1000 Genomes on GRCh38,1000 Genomes 30x on GRCh38,1000 Genomes phase 3 release
**HG03831**	female	SAME1839348	BEB	Bengali	SAS	South Asian Ancestry	BEB	1000 Genomes 30x on GRCh38
**NA10854**	female	SAME124933	CEU	CEPH	EUR	European Ancestry	CEU	1000 Genomes 30x on GRCh38,1000 Genomes phase 3 release
**HG01973**	female	SAME124328	PEL	Peruvian	AMR	American Ancestry	PEL	1000 Genomes on GRCh38,1000 Genomes 30x on GRCh38,1000 Genomes phase 3 release
**HG03836**	female	SAME1839280	STU	Tamil	SAS	South Asian Ancestry	STU	1000 Genomes on GRCh38,1000 Genomes 30x on GRCh38,1000 Genomes phase 3 release
**NA10859**	female	SAME124937	CEU	CEPH	EUR	European Ancestry	CEU	1000 Genomes 30x on GRCh38,1000 Genomes phase 3 release
**HG01978**	female	SAME124332	PEL	Peruvian	AMR	American Ancestry	PEL	1000 Genomes 30x on GRCh38,1000 Genomes phase 3 release
**HG03937**	female	SAME1840330	BEB	Bengali	SAS	South Asian Ancestry	BEB	1000 Genomes on GRCh38,1000 Genomes 30x on GRCh38,1000 Genomes phase 3 release
**NA10861**	female	SAME125130	CEU	CEPH	EUR	European Ancestry	CEU	1000 Genomes 30x on GRCh38,1000 Genomes phase 3 release
**HG01980**	female	SAME124917	PEL	Peruvian	AMR	American Ancestry	PEL	1000 Genomes on GRCh38,1000 Genomes 30x on GRCh38,1000 Genomes phase 3 release
**HG03944**	female	SAME1840368	STU	Tamil	SAS	South Asian Ancestry	STU	1000 Genomes on GRCh38,1000 Genomes 30x on GRCh38,1000 Genomes phase 3 release
**NA11840**	female	SAME125139	CEU	CEPH	EUR	European Ancestry	CEU	1000 Genomes on GRCh38,1000 Genomes 30x on GRCh38,1000 Genomes phase 3 release,1000 Genomes phase 1 release,Geuvadis
**HG01985**	female	SAME1840099	ACB	African Caribbean	AFR	African Ancestry	ACB	1000 Genomes on GRCh38,1000 Genomes 30x on GRCh38,1000 Genomes phase 3 release
**HG03949**	female	SAME1840132	STU	Tamil	SAS	South Asian Ancestry	STU	1000 Genomes on GRCh38,1000 Genomes 30x on GRCh38,1000 Genomes phase 3 release
**NA12045**	male	SAME123211	CEU	CEPH	EUR	European Ancestry	CEU	1000 Genomes on GRCh38,1000 Genomes 30x on GRCh38,1000 Genomes phase 3 release,1000 Genomes phase 1 release,Geuvadis
**HG02053**	male	SAME1839543	ACB	African Caribbean	AFR	African Ancestry	ACB	1000 Genomes on GRCh38,1000 Genomes 30x on GRCh38,1000 Genomes phase 3 release
**HG03951**	female	SAME1840239	STU	Tamil	SAS	South Asian Ancestry	STU	1000 Genomes on GRCh38,1000 Genomes 30x on GRCh38,1000 Genomes phase 3 release
**NA12057**	female	SAME123415	CEU	CEPH	EUR	European Ancestry	CEU	1000 Genomes 30x on GRCh38,1000 Genomes phase 3 release
**HG02058**	male	SAME125319	KHV	Kinh Vietnamese	EAS	East Asian Ancestry	KHV	1000 Genomes on GRCh38,1000 Genomes 30x on GRCh38,1000 Genomes phase 3 release
**HG03797**	male	SAME1839931	BEB	Bengali	SAS	South Asian Ancestry	BEB	1000 Genomes on GRCh38,1000 Genomes 30x on GRCh38
**HG04173**	male	SAME1839977	BEB	Bengali	SAS	South Asian Ancestry	BEB	1000 Genomes on GRCh38,1000 Genomes 30x on GRCh38,1000 Genomes phase 3 release
**HG02060**	female	SAME1839679	KHV	Kinh Vietnamese	EAS	East Asian Ancestry	KHV	1000 Genomes on GRCh38,1000 Genomes 30x on GRCh38,1000 Genomes phase 3 release
**HG03800**	male	SAME1839595	BEB	Bengali	SAS	South Asian Ancestry	BEB	1000 Genomes on GRCh38,1000 Genomes 30x on GRCh38,1000 Genomes phase 3 release
**HG04180**	female	SAME1840012	BEB	Bengali	SAS	South Asian Ancestry	BEB	1000 Genomes on GRCh38,1000 Genomes 30x on GRCh38,1000 Genomes phase 3 release
**HG02072**	female	SAME123105	KHV	Kinh Vietnamese	EAS	East Asian Ancestry	KHV	1000 Genomes on GRCh38,1000 Genomes 30x on GRCh38,1000 Genomes phase 3 release
**HG03805**	female	SAME1839540	BEB	Bengali	SAS	South Asian Ancestry	BEB	1000 Genomes on GRCh38,1000 Genomes 30x on GRCh38,1000 Genomes phase 3 release
**HG04185**	male	SAME1839756	BEB	Bengali	SAS	South Asian Ancestry	BEB	1000 Genomes on GRCh38,1000 Genomes 30x on GRCh38,1000 Genomes phase 3 release
**HG02027**	male	SAME125318	KHV	Kinh Vietnamese	EAS	East Asian Ancestry	KHV	1000 Genomes 30x on GRCh38,1000 Genomes phase 3 release
**HG03812**	male	SAME1839405	BEB	Bengali	SAS	South Asian Ancestry	BEB	1000 Genomes on GRCh38,1000 Genomes 30x on GRCh38,1000 Genomes phase 3 release
**HG04192**	female	SAME1839902	BEB	Bengali	SAS	South Asian Ancestry	BEB	1000 Genomes on GRCh38,1000 Genomes 30x on GRCh38
**HG03817**	female	SAME1839426	BEB	Bengali	SAS	South Asian Ancestry	BEB	1000 Genomes on GRCh38,1000 Genomes 30x on GRCh38,1000 Genomes phase 3 release
**HG02419**	female	SAME1839244	ACB	African Caribbean	AFR	African Ancestry	ACB	1000 Genomes on GRCh38,1000 Genomes 30x on GRCh38,1000 Genomes phase 3 release
**HG03906**	male	SAME1839512	BEB	Bengali	SAS	South Asian Ancestry	BEB	1000 Genomes 30x on GRCh38
**HG04200**	female	SAME1839511	ITU	Telugu	SAS	South Asian Ancestry	ITU	1000 Genomes on GRCh38,1000 Genomes 30x on GRCh38,1000 Genomes phase 3 release
**HG02433**	male	SAME1839175	ACB	African Caribbean	AFR	African Ancestry	ACB	1000 Genomes on GRCh38,1000 Genomes 30x on GRCh38,1000 Genomes phase 3 release
**HG03913**	female	SAME1839216	BEB	Bengali	SAS	South Asian Ancestry	BEB	1000 Genomes on GRCh38,1000 Genomes 30x on GRCh38,1000 Genomes phase 3 release
**HG04212**	female	SAME1839325	ITU	Telugu	SAS	South Asian Ancestry	ITU	1000 Genomes on GRCh38,1000 Genomes 30x on GRCh38,1000 Genomes phase 3 release
**HG02445**	male	SAME1840341	ACB	African Caribbean	AFR	African Ancestry	ACB	1000 Genomes on GRCh38,1000 Genomes 30x on GRCh38,1000 Genomes phase 3 release
**HG03920**	male	SAME1839093	BEB	Bengali	SAS	South Asian Ancestry	BEB	1000 Genomes on GRCh38,1000 Genomes 30x on GRCh38,1000 Genomes phase 3 release
**HG04217**	female	SAME1839350	ITU	Telugu	SAS	South Asian Ancestry	ITU	1000 Genomes 30x on GRCh38,Human Genome Structural Variation Consortium, Phase 2
**HG02464**	male	SAME1839978	GWD	Gambian Mandinka,Gar	AFR	African Ancestry,Africa (SGDP)	GWD,GambianSGDP	1000 Genomes on GRCh38,Simons Genome Diversity Project,1000 Genomes 30x on GRCh38,1000 Genomes phase 3 release,Gambian Genome Variation Project (GRCh3
**HG03925**	female	SAME1839025	BEB	Bengali	SAS	South Asian Ancestry	BEB	1000 Genomes on GRCh38,1000 Genomes 30x on GRCh38,1000 Genomes phase 3 release
**HG04142**	female	SAME1839177	BEB	Bengali	SAS	South Asian Ancestry	BEB	1000 Genomes 30x on GRCh38
**HG02558**	female	SAME1840057	ACB	African Caribbean	AFR	African Ancestry	ACB	1000 Genomes on GRCh38,1000 Genomes 30x on GRCh38,1000 Genomes phase 3 release
**HG03963**	male	SAME1840055	ITU	Telugu	SAS	South Asian Ancestry	ITU	1000 Genomes on GRCh38,1000 Genomes 30x on GRCh38,1000 Genomes phase 3 release
**HG04147**	female	SAME1839116	BEB	Bengali	SAS	South Asian Ancestry	BEB	1000 Genomes on GRCh38,1000 Genomes 30x on GRCh38
**HG02572**	male	SAME1839570	GWD	Gambian Mandinka	AFR	African Ancestry	GWD	1000 Genomes 30x on GRCh38
**HG03968**	female	SAME1840077	ITU	Telugu	SAS	South Asian Ancestry	ITU	1000 Genomes on GRCh38,1000 Genomes 30x on GRCh38,1000 Genomes phase 3 release
**HG04159**	female	SAME1840399	BEB	Bengali	SAS	South Asian Ancestry	BEB	1000 Genomes on GRCh38,1000 Genomes 30x on GRCh38,1000 Genomes phase 3 release
**HG02577**	female	SAME1839642	ACB	African Caribbean	AFR	African Ancestry	ACB	1000 Genomes on GRCh38,1000 Genomes 30x on GRCh38,1000 Genomes phase 3 release
**HG04161**	male	SAME1840092	BEB	Bengali	SAS	South Asian Ancestry	BEB	1000 Genomes on GRCh38,1000 Genomes 30x on GRCh38,1000 Genomes phase 3 release
**HG02584**	male	SAME1839425	GWD	Gambian Mandinka	AFR	African Ancestry	GWD	1000 Genomes 30x on GRCh38
**HG03778**	male	SAME1840344	ITU	Telugu	SAS	South Asian Ancestry	ITU	1000 Genomes on GRCh38,1000 Genomes 30x on GRCh38,1000 Genomes phase 3 release
**HG04096**	male	SAME1840054	ITU	Telugu	SAS	South Asian Ancestry	ITU	1000 Genomes on GRCh38,1000 Genomes 30x on GRCh38,1000 Genomes phase 3 release
**HG02589**	female	SAME1839401	GWD	Gambian Mandinka	AFR	African Ancestry	GWD	1000 Genomes on GRCh38,1000 Genomes 30x on GRCh38,1000 Genomes phase 3 release,Gambian Genome Variation Project (GRCh38)
**HG03780**	female	SAME1840112	ITU	Telugu	SAS	South Asian Ancestry	ITU	1000 Genomes on GRCh38,1000 Genomes 30x on GRCh38,1000 Genomes phase 3 release
**HG02596**	male	SAME1839201	GWD	Gambian Mandinka	AFR	African Ancestry	GWD	1000 Genomes 30x on GRCh38
**HG03785**	male	SAME1840212	ITU	Telugu	SAS	South Asian Ancestry	ITU	1000 Genomes on GRCh38,1000 Genomes 30x on GRCh38,1000 Genomes phase 3 release
**HG04135**	female	SAME1839305	BEB	Bengali	SAS	South Asian Ancestry	BEB	1000 Genomes on GRCh38,1000 Genomes 30x on GRCh38
**HG02279**	male	SAME1840362	PEL	Peruvian	AMR	American Ancestry	PEL	1000 Genomes 30x on GRCh38,1000 Genomes phase 3 release
**HG03792**	male	SAME1839912	ITU	Telugu	SAS	South Asian Ancestry	ITU	1000 Genomes on GRCh38,1000 Genomes 30x on GRCh38,1000 Genomes phase 3 release
**NA18555**	female	SAME124090	CHB	Han Chinese	EAS	East Asian Ancestry	CHB	1000 Genomes on GRCh38,1000 Genomes 30x on GRCh38,1000 Genomes phase 3 release,1000 Genomes phase 1 release,90 Han Chinese high coverage genomes
**HG02281**	male	SAME1840094	ACB	African Caribbean	AFR	African Ancestry	ACB	1000 Genomes on GRCh38,1000 Genomes 30x on GRCh38,1000 Genomes phase 3 release
**NA12155**	male	SAME124955	CEU	CEPH	EUR	European Ancestry	CEU	1000 Genomes on GRCh38,1000 Genomes 30x on GRCh38,1000 Genomes phase 3 release,1000 Genomes phase 1 release,Geuvadis
**NA18562**	male	SAME123927	CHB	Han Chinese	EAS	East Asian Ancestry	CHB	1000 Genomes on GRCh38,1000 Genomes 30x on GRCh38,1000 Genomes phase 3 release,1000 Genomes phase 1 release,90 Han Chinese high coverage genomes
**HG02286**	female	SAME122912	PEL	Peruvian	AMR	American Ancestry	PEL	1000 Genomes on GRCh38,1000 Genomes 30x on GRCh38,1000 Genomes phase 3 release
**NA12249**	female	SAME125371	CEU	CEPH	EUR	European Ancestry	CEU	1000 Genomes on GRCh38,1000 Genomes 30x on GRCh38,1000 Genomes phase 3 release,1000 Genomes phase 1 release,Geuvadis
**NA18567**	female	SAME123924	CHB	Han Chinese	EAS	East Asian Ancestry	CHB	1000 Genomes on GRCh38,1000 Genomes 30x on GRCh38,1000 Genomes phase 3 release,1000 Genomes phase 1 release
**HG02293**	female	SAME1839934	PEL	Peruvian	AMR	American Ancestry	PEL	1000 Genomes 30x on GRCh38,1000 Genomes phase 3 release
**NA12275**	female	SAME125263	CEU	CEPH	EUR	European Ancestry	CEU	1000 Genomes on GRCh38,1000 Genomes 30x on GRCh38,1000 Genomes phase 3 release,1000 Genomes phase 1 release,Geuvadis
**NA18574**	female	SAME123737	CHB	Han Chinese	EAS	East Asian Ancestry	CHB	1000 Genomes on GRCh38,1000 Genomes 30x on GRCh38,1000 Genomes phase 3 release,1000 Genomes phase 1 release
**HG02298**	female	SAME1839913	PEL	Peruvian	AMR	American Ancestry	PEL	1000 Genomes on GRCh38,1000 Genomes 30x on GRCh38,1000 Genomes phase 3 release
**NA12282**	male	SAME124640	CEU	CEPH	EUR	European Ancestry	CEU	1000 Genomes on GRCh38,1000 Genomes 30x on GRCh38,1000 Genomes phase 3 release,1000 Genomes phase 1 release,Geuvadis
**NA18579**	female	SAME123742	CHB	Han Chinese	EAS	East Asian Ancestry	CHB	1000 Genomes on GRCh38,1000 Genomes 30x on GRCh38,1000 Genomes phase 3 release,1000 Genomes phase 1 release,90 Han Chinese high coverage genomes
**HG02301**	female	SAME124280	PEL	Peruvian	AMR	American Ancestry	PEL	1000 Genomes on GRCh38,1000 Genomes 30x on GRCh38,1000 Genomes phase 3 release
**NA12287**	female	SAME124641	CEU	CEPH	EUR	European Ancestry	CEU	1000 Genomes on GRCh38,1000 Genomes 30x on GRCh38,1000 Genomes phase 3 release,1000 Genomes phase 1 release,Geuvadis
**NA18593**	female	SAME123720	CHB	Han Chinese	EAS	East Asian Ancestry	CHB	1000 Genomes on GRCh38,1000 Genomes 30x on GRCh38,1000 Genomes phase 3 release,1000 Genomes phase 1 release,90 Han Chinese high coverage genomes
**HG02471**	female	SAME1839741	ACB	African Caribbean	AFR	African Ancestry	ACB	1000 Genomes on GRCh38,1000 Genomes 30x on GRCh38,1000 Genomes phase 3 release
**NA12340**	male	SAME125157	CEU	CEPH	EUR	European Ancestry	CEU	1000 Genomes on GRCh38,1000 Genomes 30x on GRCh38,1000 Genomes phase 3 release,1000 Genomes phase 1 release,Geuvadis
**NA18606**	male	SAME124879	CHB	Han Chinese	EAS	East Asian Ancestry	CHB	1000 Genomes on GRCh38,1000 Genomes 30x on GRCh38,1000 Genomes phase 3 release,1000 Genomes phase 1 release
**HG02476**	female	SAME1839717	ACB	African Caribbean	AFR	African Ancestry	ACB	1000 Genomes on GRCh38,1000 Genomes 30x on GRCh38,1000 Genomes phase 3 release
**NA12376**	female	SAME123275	CEU	CEPH	EUR	European Ancestry	CEU	1000 Genomes 30x on GRCh38,1000 Genomes phase 3 release
**NA18613**	male	SAME125037	CHB	Han Chinese	EAS	East Asian Ancestry	CHB	1000 Genomes on GRCh38,1000 Genomes 30x on GRCh38,1000 Genomes phase 3 release,1000 Genomes phase 1 release
**HG02490**	male	SAME1839625	PJL	Punjabi	SAS	South Asian Ancestry	PJL	1000 Genomes on GRCh38,1000 Genomes 30x on GRCh38,1000 Genomes phase 3 release
**NA12383**	female	SAME124384	CEU	CEPH	EUR	European Ancestry	CEU	1000 Genomes on GRCh38,1000 Genomes 30x on GRCh38,1000 Genomes phase 3 release,1000 Genomes phase 1 release,Geuvadis
**NA18618**	female	SAME125042	CHB	Han Chinese	EAS	East Asian Ancestry	CHB	1000 Genomes on GRCh38,1000 Genomes 30x on GRCh38,1000 Genomes phase 3 release,1000 Genomes phase 1 release
**HG02495**	female	SAME1839694	PJL	Punjabi	SAS	South Asian Ancestry	PJL	1000 Genomes 30x on GRCh38
**NA12489**	female	SAME125358	CEU	CEPH	EUR	European Ancestry	CEU	1000 Genomes on GRCh38,1000 Genomes 30x on GRCh38,1000 Genomes phase 3 release,1000 Genomes phase 1 release,Geuvadis
**NA18620**	male	SAME124510	CHB	Han Chinese	EAS	East Asian Ancestry	CHB	1000 Genomes on GRCh38,1000 Genomes 30x on GRCh38,1000 Genomes phase 3 release,1000 Genomes phase 1 release,90 Han Chinese high coverage genomes
**HG02318**	female	SAME1839391	ACB	African Caribbean	AFR	African Ancestry	ACB	1000 Genomes on GRCh38,1000 Genomes 30x on GRCh38,1000 Genomes phase 3 release
**NA10851**	male	SAME125317	CEU	CEPH	EUR	European Ancestry	CEU	1000 Genomes on GRCh38,1000 Genomes 30x on GRCh38,1000 Genomes phase 3 release,1000 Genomes phase 1 release,Geuvadis
**NA18625**	female	SAME125253	CHB	Han Chinese	EAS	East Asian Ancestry	CHB	1000 Genomes on GRCh38,1000 Genomes 30x on GRCh38,1000 Genomes phase 3 release,1000 Genomes phase 1 release
**HG02325**	female	SAME1839521	ACB	African Caribbean	AFR	African Ancestry	ACB	1000 Genomes on GRCh38,1000 Genomes 30x on GRCh38,1000 Genomes phase 3 release

**NA10856**	male	SAME124935	CEU	CEPH	EUR	European Ancestry	CEU		1000 Genomes 30x on GRCh38,1000 Genomes phase 3 release
**NA18632**	male	SAME124703	CHB	Han Chinese	EAS	East Asian Ancestry	CHB		1000 Genomes on GRCh38,1000 Genomes 30x on GRCh38,1000 Genomes phase 3 release,1000 Genomes phase 1 release,90 Han Chinese high coverage genomes
**HG02332**	male	SAME1839315	ACB	African Caribbean	AFR	African Ancestry	ACB		1000 Genomes on GRCh38,1000 Genomes 30x on GRCh38,1000 Genomes phase 3 release
**NA10863**	female	SAME125132	CEU	CEPH	EUR	European Ancestry	CEU		1000 Genomes 30x on GRCh38,1000 Genomes phase 3 release
**NA18637**	male	SAME124700	CHB	Han Chinese	EAS	East Asian Ancestry	CHB		1000 Genomes on GRCh38,1000 Genomes 30x on GRCh38,1000 Genomes phase 3 release,1000 Genomes phase 1 release,90 Han Chinese high coverage genomes
**HG02337**	female	SAME1839296	ACB	African Caribbean	AFR	African Ancestry	ACB		1000 Genomes on GRCh38,1000 Genomes 30x on GRCh38,1000 Genomes phase 3 release
**NA11830**	female	SAME124553	CEU	CEPH	EUR	European Ancestry	CEU		1000 Genomes on GRCh38,1000 Genomes 30x on GRCh38,1000 Genomes phase 3 release,1000 Genomes phase 1 release,Geuvadis
**NA18644**	female	SAME122976	CHB	Han Chinese	EAS	East Asian Ancestry	CHB		1000 Genomes on GRCh38,1000 Genomes 30x on GRCh38,1000 Genomes phase 3 release
**NA11892**	female	SAME123045	CEU	CEPH	EUR	European Ancestry	CEU		1000 Genomes on GRCh38,1000 Genomes 30x on GRCh38,1000 Genomes phase 3 release,1000 Genomes phase 1 release,Geuvadis
**NA18740**	male	SAME123917	CHB	Han Chinese	EAS	East Asian Ancestry	CHB		1000 Genomes on GRCh38,1000 Genomes 30x on GRCh38,1000 Genomes phase 3 release,1000 Genomes phase 1 release
**HG02508**	female	SAME1839334	ACB	African Caribbean	AFR	African Ancestry	ACB		1000 Genomes on GRCh38,1000 Genomes 30x on GRCh38,1000 Genomes phase 3 release
**NA11917**	male	SAME122838	CEU	CEPH	EUR	European Ancestry	CEU		1000 Genomes 30x on GRCh38,1000 Genomes phase 3 release
**NA18745**	male	SAME123914	CHB	Han Chinese	EAS	East Asian Ancestry	CHB		1000 Genomes on GRCh38,1000 Genomes 30x on GRCh38,1000 Genomes phase 3 release,1000 Genomes phase 1 release
**HG02522**	female	SAME1840326	KHV	Kinh Vietnamese	EAS	East Asian Ancestry	KHV		1000 Genomes on GRCh38,1000 Genomes 30x on GRCh38,1000 Genomes phase 3 release
**NA11931**	female	SAME125096	CEU	CEPH	EUR	European Ancestry	CEU		1000 Genomes on GRCh38,1000 Genomes 30x on GRCh38,1000 Genomes phase 3 release,1000 Genomes phase 1 release,Geuvadis
**NA18757**	male	SAME123727	CHB	Han Chinese	EAS	East Asian Ancestry	CHB		1000 Genomes on GRCh38,1000 Genomes 30x on GRCh38,1000 Genomes phase 3 release,1000 Genomes phase 1 release
**HG02541**	male	SAME1840214	ACB	African Caribbean	AFR	African Ancestry	ACB		1000 Genomes on GRCh38,1000 Genomes 30x on GRCh38,1000 Genomes phase 3 release
**NA11993**	female	SAME123965	CEU	CEPH	EUR	European Ancestry	CEU		1000 Genomes on GRCh38,1000 Genomes 30x on GRCh38,1000 Genomes phase 3 release,1000 Genomes phase 1 release,Geuvadis
**HG02546**	female	SAME1839932	ACB	African Caribbean	AFR	African Ancestry	ACB		1000 Genomes on GRCh38,1000 Genomes 30x on GRCh38,1000 Genomes phase 3 release
**NA12004**	female	SAME125144	CEU	CEPH	EUR	European Ancestry	CEU		1000 Genomes on GRCh38,1000 Genomes 30x on GRCh38,1000 Genomes phase 3 release,1000 Genomes phase 1 release,Geuvadis
**NA18853**	male	SAME124733	YRI	Yoruba	AFR	African Ancestry	YRI		1000 Genomes on GRCh38,1000 Genomes 30x on GRCh38,1000 Genomes phase 3 release,1000 Genomes phase 1 release
**HG02351**	male	SAME1840284	CDX	Dai Chinese	EAS	East Asian Ancestry	CDX		1000 Genomes on GRCh38,1000 Genomes 30x on GRCh38,1000 Genomes phase 3 release
**HG04238**	male	SAME1840274	ITU	Telugu	SAS	South Asian Ancestry	ITU		1000 Genomes on GRCh38,1000 Genomes 30x on GRCh38,1000 Genomes phase 3 release
**NA18916**	female	SAME123408	YRI	Yoruba	AFR	African Ancestry	YRI		1000 Genomes on GRCh38,1000 Genomes 30x on GRCh38,1000 Genomes phase 3 release,1000 Genomes phase 1 release,Geuvadis
**HG02356**	male	SAME1840261	CDX	Dai Chinese	EAS	East Asian Ancestry	CDX		1000 Genomes on GRCh38,1000 Genomes 30x on GRCh38,1000 Genomes phase 3 release
**NA18923**	male	SAME123205	YRI	Yoruba	AFR	African Ancestry	YRI		1000 Genomes on GRCh38,1000 Genomes 30x on GRCh38,1000 Genomes phase 3 release,1000 Genomes phase 1 release,Geuvadis
**NA06994**	male	SAME124931	CEU	CEPH	EUR	European Ancestry	CEU		1000 Genomes on GRCh38,1000 Genomes 30x on GRCh38,1000 Genomes phase 3 release,1000 Genomes phase 1 release,Geuvadis
**NA18930**	female	SAME123040	YRI	Yoruba	AFR	African Ancestry	YRI		1000 Genomes 30x on GRCh38,1000 Genomes phase 3 release
**HG02375**	male	SAME1840244	CDX	Dai Chinese	EAS	East Asian Ancestry	CDX		1000 Genomes on GRCh38,1000 Genomes 30x on GRCh38,1000 Genomes phase 3 release
**NA07000**	female	SAME124963	CEU	CEPH	EUR	European Ancestry	CEU		1000 Genomes on GRCh38,1000 Genomes 30x on GRCh38,1000 Genomes phase 3 release,1000 Genomes phase 1 release,Geuvadis
**NA18935**	male	SAME123035	YRI	Yoruba	AFR	African Ancestry	YRI		1000 Genomes 30x on GRCh38,1000 Genomes phase 3 release
**HG02382**	male	SAME1840025	CDX	Dai Chinese	EAS	East Asian Ancestry	CDX		1000 Genomes on GRCh38,1000 Genomes 30x on GRCh38,1000 Genomes phase 3 release
**NA07029**	male	SAME125338	CEU	CEPH	EUR	European Ancestry	CEU		1000 Genomes 30x on GRCh38,1000 Genomes phase 3 release
**NA18942**	female	SAME124308	JPT	Japanese	EAS	East Asian Ancestry	JPT		1000 Genomes on GRCh38,1000 Genomes 30x on GRCh38,1000 Genomes phase 3 release,1000 Genomes phase 1 release
**NA07031**	female	SAME122860	CEU	CEPH	EUR	European Ancestry	CEU		1000 Genomes 30x on GRCh38,1000 Genomes phase 3 release
**NA18947**	female	SAME124313	JPT	Japanese	EAS	East Asian Ancestry	JPT		1000 Genomes on GRCh38,1000 Genomes 30x on GRCh38,1000 Genomes phase 3 release,1000 Genomes phase 1 release
**HG02394**	male	SAME123305	CDX	Dai Chinese	EAS	East Asian Ancestry	CDX		1000 Genomes on GRCh38,1000 Genomes 30x on GRCh38,1000 Genomes phase 3 release
**NA07048**	male	SAME124188	CEU	CEPH	EUR	European Ancestry	CEU		1000 Genomes on GRCh38,1000 Genomes 30x on GRCh38,1000 Genomes phase 3 release,1000 Genomes phase 1 release,Geuvadis
**NA18954**	female	SAME124108	JPT	Japanese	EAS	East Asian Ancestry	JPT		1000 Genomes on GRCh38,1000 Genomes 30x on GRCh38,1000 Genomes phase 3 release,1000 Genomes phase 1 release
**HG02399**	male	SAME1839842	CDX	Dai Chinese	EAS	East Asian Ancestry	CDX		1000 Genomes on GRCh38,1000 Genomes 30x on GRCh38,1000 Genomes phase 3 release
**NA07055**	female	SAME124767	CEU	CEPH	EUR	European Ancestry	CEU		1000 Genomes 30x on GRCh38,1000 Genomes phase 3 release
**NA18959**	male	SAME124115	JPT	Japanese	EAS	East Asian Ancestry	JPT		1000 Genomes on GRCh38,1000 Genomes 30x on GRCh38,1000 Genomes phase 3 release,1000 Genomes phase 1 release
**HG02402**	male	SAME1839412	CDX	Dai Chinese	EAS	East Asian Ancestry	CDX		1000 Genomes on GRCh38,1000 Genomes 30x on GRCh38,1000 Genomes phase 3 release
**NA07346**	female	SAME123571	CEU	CEPH	EUR	European Ancestry	CEU		1000 Genomes on GRCh38,1000 Genomes 30x on GRCh38,1000 Genomes phase 3 release,1000 Genomes phase 1 release,Geuvadis
**NA18961**	male	SAME123957	JPT	Japanese	EAS	East Asian Ancestry	JPT		1000 Genomes on GRCh38,1000 Genomes 30x on GRCh38,1000 Genomes phase 3 release,1000 Genomes phase 1 release
**HG02407**	male	SAME1839464	CDX	Dai Chinese	EAS	East Asian Ancestry	CDX		1000 Genomes on GRCh38,1000 Genomes 30x on GRCh38,1000 Genomes phase 3 release
**NA07435**	male	SAME124342	CEU	CEPH	EUR	European Ancestry	CEU		1000 Genomes 30x on GRCh38,1000 Genomes phase 3 release
**NA18966**	male	SAME123962	JPT	Japanese	EAS	East Asian Ancestry	JPT		1000 Genomes on GRCh38,1000 Genomes 30x on GRCh38,1000 Genomes phase 3 release,1000 Genomes phase 1 release
**HG02255**	male	SAME1839337	ACB	African Caribbean	AFR	African Ancestry	ACB		1000 Genomes on GRCh38,1000 Genomes 30x on GRCh38,1000 Genomes phase 3 release
**NA10837**	male	SAME124546	CEU	CEPH	EUR	European Ancestry	CEU		1000 Genomes 30x on GRCh38,1000 Genomes phase 3 release
**NA18973**	female	SAME123761	JPT	Japanese	EAS	East Asian Ancestry	JPT		1000 Genomes on GRCh38,1000 Genomes 30x on GRCh38,1000 Genomes phase 3 release,1000 Genomes phase 1 release
**HG02262**	male	SAME1839128	PEL	Peruvian	AMR	American Ancestry	PEL		1000 Genomes on GRCh38,1000 Genomes 30x on GRCh38,1000 Genomes phase 3 release
**HG04194**	male	SAME1839818	BEB	Bengali	SAS	South Asian Ancestry	BEB		1000 Genomes on GRCh38,1000 Genomes 30x on GRCh38,1000 Genomes phase 3 release
**NA18978**	female	SAME123760	JPT	Japanese	EAS	East Asian Ancestry	JPT		1000 Genomes on GRCh38,1000 Genomes 30x on GRCh38,1000 Genomes phase 3 release,1000 Genomes phase 1 release
**HG04199**	male	SAME1839840	STU	Tamil	SAS	South Asian Ancestry	STU		1000 Genomes 30x on GRCh38
**HG02274**	male	SAME1840306	PEL	Peruvian	AMR	American Ancestry	PEL		1000 Genomes on GRCh38,1000 Genomes 30x on GRCh38,1000 Genomes phase 3 release
**HG04202**	female	SAME1839519	ITU	Telugu	SAS	South Asian Ancestry	ITU		1000 Genomes on GRCh38,1000 Genomes 30x on GRCh38,1000 Genomes phase 3 release
**NA19058**	male	SAME125024	JPT	Japanese	EAS	East Asian Ancestry	JPT		1000 Genomes on GRCh38,1000 Genomes 30x on GRCh38,1000 Genomes phase 3 release,1000 Genomes phase 1 release
**HG02604**	female	SAME1840282	PJL	Punjabi	SAS	South Asian Ancestry	PJL		1000 Genomes on GRCh38,1000 Genomes 30x on GRCh38,1000 Genomes phase 3 release
**HG04214**	female	SAME1839328	ITU	Telugu	SAS	South Asian Ancestry	ITU		1000 Genomes on GRCh38,1000 Genomes 30x on GRCh38,1000 Genomes phase 3 release
**NA19060**	male	SAME123007	JPT	Japanese	EAS	East Asian Ancestry	JPT		1000 Genomes on GRCh38,1000 Genomes 30x on GRCh38,1000 Genomes phase 3 release,1000 Genomes phase 1 release
**HG02611**	female	SAME1840401	GWD	Gambian Mandinka	AFR	African Ancestry	GWD		1000 Genomes on GRCh38,1000 Genomes 30x on GRCh38,1000 Genomes phase 3 release,Gambian Genome Variation Project (GRCh38)
**HG04219**	male	SAME1839359	ITU	Telugu	SAS	South Asian Ancestry	ITU		1000 Genomes on GRCh38,1000 Genomes 30x on GRCh38,1000 Genomes phase 3 release
**NA19065**	female	SAME123011	JPT	Japanese	EAS	East Asian Ancestry	JPT		1000 Genomes on GRCh38,1000 Genomes 30x on GRCh38,1000 Genomes phase 3 release,1000 Genomes phase 1 release
**HG02623**	male	SAME1840182	GWD	Gambian Mandinka	AFR	African Ancestry	GWD		1000 Genomes on GRCh38,1000 Genomes 30x on GRCh38,1000 Genomes phase 3 release,Gambian Genome Variation Project (GRCh38)
**HG04093**	male	SAME1839936	ITU	Telugu	SAS	South Asian Ancestry	ITU		1000 Genomes on GRCh38,1000 Genomes 30x on GRCh38,1000 Genomes phase 3 release
**NA19072**	male	SAME122822	JPT	Japanese	EAS	East Asian Ancestry	JPT		1000 Genomes on GRCh38,1000 Genomes 30x on GRCh38,1000 Genomes phase 3 release,1000 Genomes phase 1 release
**HG02628**	male	SAME1840238	GWD	Gambian Mandinka	AFR	African Ancestry	GWD		1000 Genomes on GRCh38,1000 Genomes 30x on GRCh38,1000 Genomes phase 3 release,Gambian Genome Variation Project (GRCh38)
**HG04098**	male	SAME1840059	ITU	Telugu	SAS	South Asian Ancestry	ITU		1000 Genomes on GRCh38,1000 Genomes 30x on GRCh38,1000 Genomes phase 3 release
**NA19077**	female	SAME122818	JPT	Japanese	EAS	East Asian Ancestry	JPT		1000 Genomes on GRCh38,1000 Genomes 30x on GRCh38,1000 Genomes phase 3 release,1000 Genomes phase 1 release
**HG02630**	female	SAME1839963	GWD	Gambian Mandinka	AFR	African Ancestry	GWD		1000 Genomes 30x on GRCh38
**HG04106**	female	SAME1839559	STU	Tamil	SAS	South Asian Ancestry	STU		1000 Genomes on GRCh38,1000 Genomes 30x on GRCh38,1000 Genomes phase 3 release
**NA19084**	female	SAME123364	JPT	Japanese	EAS	East Asian Ancestry	JPT		1000 Genomes on GRCh38,1000 Genomes 30x on GRCh38,1000 Genomes phase 3 release,1000 Genomes phase 1 release
**HG02635**	female	SAME1840023	GWD	Gambian Mandinka	AFR	African Ancestry	GWD		1000 Genomes on GRCh38,1000 Genomes 30x on GRCh38,1000 Genomes phase 3 release,Gambian Genome Variation Project (GRCh38)
**HG04118**	female	SAME1839379	ITU	Telugu	SAS	South Asian Ancestry	ITU		1000 Genomes on GRCh38,1000 Genomes 30x on GRCh38,1000 Genomes phase 3 release
**NA19089**	male	SAME123649	JPT	Japanese	EAS	East Asian Ancestry	JPT		1000 Genomes on GRCh38,1000 Genomes 30x on GRCh38,1000 Genomes phase 3 release
**HG02642**	male	SAME1839792	GWD	Gambian Mandinka	AFR	African Ancestry	GWD		1000 Genomes on GRCh38,1000 Genomes 30x on GRCh38,1000 Genomes phase 3 release,Gambian Genome Variation Project (GRCh38)
**HG04132**	female	SAME1839287	BEB	Bengali	SAS	South Asian Ancestry	BEB		1000 Genomes on GRCh38,1000 Genomes 30x on GRCh38
**NA19091**	male	SAME123174	JPT	Japanese	EAS	East Asian Ancestry	JPT		1000 Genomes on GRCh38,1000 Genomes 30x on GRCh38,1000 Genomes phase 3 release
**HG02647**	male	SAME1839762	GWD	Gambian Mandinka	AFR	African Ancestry	GWD		1000 Genomes 30x on GRCh38
**NA19096**	male	SAME123662	YRI	Yoruba	AFR	African Ancestry	YRI		1000 Genomes on GRCh38,1000 Genomes 30x on GRCh38,1000 Genomes phase 3 release,1000 Genomes phase 1 release,Geuvadis
**HG02786**	male	SAME1840292	PJL	Punjabi	SAS	South Asian Ancestry	PJL		1000 Genomes on GRCh38,1000 Genomes 30x on GRCh38,1000 Genomes phase 3 release
**HG04062**	female	SAME1839147	ITU	Telugu	SAS	South Asian Ancestry	ITU		1000 Genomes on GRCh38,1000 Genomes 30x on GRCh38,1000 Genomes phase 3 release
**NA19109**	female	SAME124544	YRI	Yoruba	AFR	African Ancestry	YRI		1000 Genomes 30x on GRCh38,1000 Genomes phase 3 release
**HG02793**	female	SAME1840415	PJL	Punjabi	SAS	South Asian Ancestry	PJL		1000 Genomes on GRCh38,1000 Genomes 30x on GRCh38,1000 Genomes phase 3 release
**NA18497**	male	SAME124277	YRI	Yoruba	AFR	African Ancestry	YRI		1000 Genomes 30x on GRCh38,1000 Genomes phase 3 release
**HG02798**	male	SAME1840387	GWD	Gambian Mandinka	AFR	African Ancestry	GWD		1000 Genomes on GRCh38,1000 Genomes 30x on GRCh38,1000 Genomes phase 3 release,Gambian Genome Variation Project (GRCh38)
**HG04144**	female	SAME1839189	BEB	Bengali	SAS	South Asian Ancestry	BEB		1000 Genomes on GRCh38,1000 Genomes 30x on GRCh38,1000 Genomes phase 3 release
**NA18500**	male	SAME123386	YRI	Yoruba	AFR	African Ancestry	YRI		1000 Genomes 30x on GRCh38,1000 Genomes phase 3 release
**HG02806**	female	SAME1840079	GWD	Gambian Mandinka	AFR	African Ancestry	GWD		1000 Genomes 30x on GRCh38
**HG04149**	male	SAME1839120	BEB	Bengali	SAS	South Asian Ancestry	BEB		1000 Genomes on GRCh38,1000 Genomes 30x on GRCh38
**NA18505**	female	SAME125062	YRI	Yoruba	AFR	African Ancestry	YRI		1000 Genomes on GRCh38,1000 Genomes 30x on GRCh38,1000 Genomes phase 3 release,1000 Genomes phase 1 release,Geuvadis
**HG02813**	male	SAME1839841	GWD	Gambian Mandinka	AFR	African Ancestry	GWD		1000 Genomes on GRCh38,1000 Genomes 30x on GRCh38,1000 Genomes phase 3 release,Gambian Genome Variation Project (GRCh38)
**HG04151**	female	SAME1840295	BEB	Bengali	SAS	South Asian Ancestry	BEB		1000 Genomes 30x on GRCh38
**NA18517**	female	SAME124883	YRI	Yoruba	AFR	African Ancestry	YRI		1000 Genomes on GRCh38,1000 Genomes 30x on GRCh38,1000 Genomes phase 3 release,1000 Genomes phase 1 release,Geuvadis
**HG02818**	female	SAME1839810	GWD	Gambian Mandinka	AFR	African Ancestry	GWD		1000 Genomes 30x on GRCh38,Human Genome Structural Variation Consortium, Phase 2
**HG04156**	female	SAME1840389	BEB	Bengali	SAS	South Asian Ancestry	BEB		1000 Genomes on GRCh38,1000 Genomes 30x on GRCh38,1000 Genomes phase 3 release
**NA18531**	female	SAME124483	CHB	Han Chinese	EAS	East Asian Ancestry	CHB		1000 Genomes on GRCh38,1000 Genomes 30x on GRCh38,1000 Genomes phase 3 release,1000 Genomes phase 1 release
**HG02971**	male	SAME1840255	ESN	Esan	AFR	African Ancestry	ESN		1000 Genomes on GRCh38,1000 Genomes 30x on GRCh38,1000 Genomes phase 3 release
**HG04175**	female	SAME1839980	BEB	Bengali	SAS	South Asian Ancestry	BEB		1000 Genomes 30x on GRCh38
**NA18536**	male	SAME124486	CHB	Han Chinese	EAS	East Asian Ancestry	CHB		1000 Genomes on GRCh38,1000 Genomes 30x on GRCh38,1000 Genomes phase 3 release,1000 Genomes phase 1 release
**HG02976**	female	SAME1840223	ESN	Esan	AFR	African Ancestry	ESN		1000 Genomes on GRCh38,1000 Genomes 30x on GRCh38,1000 Genomes phase 3 release
**HG04182**	male	SAME1840036	BEB	Bengali	SAS	South Asian Ancestry	BEB		1000 Genomes on GRCh38,1000 Genomes 30x on GRCh38,1000 Genomes phase 3 release
**NA18543**	male	SAME124268	CHB	Han Chinese	EAS	East Asian Ancestry	CHB		1000 Genomes on GRCh38,1000 Genomes 30x on GRCh38,1000 Genomes phase 3 release,1000 Genomes phase 1 release
**HG02983**	female	SAME1840009	GWD	Gambian Mandinka	AFR	African Ancestry	GWD		1000 Genomes on GRCh38,1000 Genomes 30x on GRCh38,1000 Genomes phase 3 release,Gambian Genome Variation Project (GRCh38)
**HG04187**	male	SAME1839763	BEB	Bengali	SAS	South Asian Ancestry	BEB		1000 Genomes 30x on GRCh38
**NA18548**	male	SAME124261	CHB	Han Chinese	EAS	East Asian Ancestry	CHB		1000 Genomes on GRCh38,1000 Genomes 30x on GRCh38,1000 Genomes phase 3 release,1000 Genomes phase 1 release
**HG03006**	male	SAME1840242	BEB	Bengali,Bengali	SAS	South Asia (SGDP),South Asian Ancestry	BengaliSGDP,BEB		1000 Genomes on GRCh38,Simons Genome Diversity Project,1000 Genomes 30x on GRCh38,1000 Genomes phase 3 release
**NA18538**	female	SAME123774	CHB	Han Chinese	EAS	East Asian Ancestry	CHB		1000 Genomes on GRCh38,1000 Genomes 30x on GRCh38,1000 Genomes phase 3 release,1000 Genomes phase 1 release
**NA18550**	female	SAME124087	CHB	Han Chinese	EAS	East Asian Ancestry	CHB		1000 Genomes on GRCh38,1000 Genomes 30x on GRCh38,1000 Genomes phase 3 release,1000 Genomes phase 1 release,90 Han Chinese high coverage genomes
**HG03018**	male	SAME1840053	PJL	Punjabi	SAS	South Asian Ancestry	PJL		1000 Genomes on GRCh38,1000 Genomes 30x on GRCh38,1000 Genomes phase 3 release

**NA18545**	female	SAME124266	CHB	Han Chinese	EAS	East Asian Ancestry	CHB	1000 Genomes on GRCh38,1000 Genomes 30x on GRCh38,1000 Genomes phase 3 release,1000 Genomes phase 1 release,90 Han Chinese high coverage genomes
**NA19116**	female	SAME124911	YRI	Yoruba	AFR	African Ancestry	YRI	1000 Genomes on GRCh38,1000 Genomes 30x on GRCh38,1000 Genomes phase 3 release,1000 Genomes phase 1 release,Geuvadis
**HG02654**	male	SAME1839530	PJL	Punjabi	SAS	South Asian Ancestry	PJL	1000 Genomes on GRCh38,1000 Genomes 30x on GRCh38,1000 Genomes phase 3 release
**NA18552**	female	SAME124089	CHB	Han Chinese	EAS	East Asian Ancestry	CHB	1000 Genomes on GRCh38,1000 Genomes 30x on GRCh38,1000 Genomes phase 3 release,1000 Genomes phase 1 release,90 Han Chinese high coverage genomes
**NA19123**	male	SAME125013	YRI	Yoruba	AFR	African Ancestry	YRI	1000 Genomes 30x on GRCh38,1000 Genomes phase 3 release
**HG02659**	female	SAME1839590	PJL	Punjabi	SAS	South Asian Ancestry	PJL	1000 Genomes 30x on GRCh38,1000 Genomes phase 3 release
**NA18557**	male	SAME124092	CHB	Han Chinese	EAS	East Asian Ancestry	CHB	1000 Genomes on GRCh38,1000 Genomes 30x on GRCh38,1000 Genomes phase 3 release,1000 Genomes phase 1 release
**NA19128**	male	SAME125022	YRI	Yoruba	AFR	African Ancestry	YRI	1000 Genomes 30x on GRCh38,1000 Genomes phase 3 release
**HG02661**	female	SAME1839667	PJL	Punjabi	SAS	South Asian Ancestry	PJL	1000 Genomes on GRCh38,1000 Genomes 30x on GRCh38,1000 Genomes phase 3 release
**NA18564**	female	SAME123921	CHB	Han Chinese	EAS	East Asian Ancestry	CHB	1000 Genomes on GRCh38,1000 Genomes 30x on GRCh38,1000 Genomes phase 3 release,1000 Genomes phase 1 release,90 Han Chinese high coverage genomes
**NA19130**	male	SAME125229	YRI	Yoruba	AFR	African Ancestry	YRI	1000 Genomes on GRCh38,1000 Genomes 30x on GRCh38,1000 Genomes phase 3 release,1000 Genomes phase 1 release,Geuvadis
**HG02666**	male	SAME1839386	GWD	Gambian Mandinka	AFR	African Ancestry	GWD	1000 Genomes on GRCh38,1000 Genomes 30x on GRCh38,1000 Genomes phase 3 release,Gambian Genome Variation Project (GRCh38)
**NA18571**	female	SAME123740	CHB	Han Chinese	EAS	East Asian Ancestry	CHB	1000 Genomes on GRCh38,1000 Genomes 30x on GRCh38,1000 Genomes phase 3 release,1000 Genomes phase 1 release,90 Han Chinese high coverage genomes
**NA19142**	male	SAME124610	YRI	Yoruba	AFR	African Ancestry	YRI	1000 Genomes 30x on GRCh38,1000 Genomes phase 3 release
**HG02678**	male	SAME1839477	GWD	Gambian Mandinka	AFR	African Ancestry	GWD	1000 Genomes on GRCh38,1000 Genomes 30x on GRCh38,1000 Genomes phase 3 release,Gambian Genome Variation Project (GRCh38)
**NA19147**	female	SAME124613	YRI	Yoruba	AFR	African Ancestry	YRI	1000 Genomes on GRCh38,1000 Genomes 30x on GRCh38,1000 Genomes phase 3 release,1000 Genomes phase 1 release,Geuvadis
**HG02680**	female	SAME1839308	GWD	Gambian Mandinka	AFR	African Ancestry	GWD	1000 Genomes 30x on GRCh38
**NA18595**	female	SAME123228	CHB	Han Chinese	EAS	East Asian Ancestry	CHB	1000 Genomes on GRCh38,1000 Genomes 30x on GRCh38,1000 Genomes phase 3 release,1000 Genomes phase 1 release
**NA19154**	male	SAME124835	YRI	Yoruba	AFR	African Ancestry	YRI	1000 Genomes 30x on GRCh38,1000 Genomes phase 3 release
**HG02685**	female	SAME1839286	PJL	Punjabi	SAS	South Asian Ancestry	PJL	1000 Genomes on GRCh38,1000 Genomes 30x on GRCh38,1000 Genomes phase 3 release
**NA18603**	male	SAME124875	CHB	Han Chinese	EAS	East Asian Ancestry	CHB	1000 Genomes on GRCh38,1000 Genomes 30x on GRCh38,1000 Genomes phase 3 release,1000 Genomes phase 1 release,90 Han Chinese high coverage genomes
**NA19159**	female	SAME124716	YRI	Yoruba	AFR	African Ancestry	YRI	1000 Genomes on GRCh38,1000 Genomes 30x on GRCh38,1000 Genomes phase 3 release,1000 Genomes phase 1 release,Geuvadis
**HG02692**	female	SAME1839078	PJL	Punjabi	SAS	South Asian Ancestry	PJL	1000 Genomes 30x on GRCh38
**NA18608**	male	SAME124779	CHB	Han Chinese	EAS	East Asian Ancestry	CHB	1000 Genomes on GRCh38,1000 Genomes 30x on GRCh38,1000 Genomes phase 3 release,1000 Genomes phase 1 release,90 Han Chinese high coverage genomes
**NA19161**	male	SAME123757	YRI	Yoruba	AFR	African Ancestry	YRI	1000 Genomes 30x on GRCh38,1000 Genomes phase 3 release
**HG02697**	female	SAME1839142	PJL	Punjabi	SAS	South Asian Ancestry	PJL	1000 Genomes on GRCh38,1000 Genomes 30x on GRCh38,1000 Genomes phase 3 release
**NA18610**	female	SAME123222	CHB	Han Chinese	EAS	East Asian Ancestry	CHB	1000 Genomes on GRCh38,1000 Genomes 30x on GRCh38,1000 Genomes phase 3 release,1000 Genomes phase 1 release
**HG02700**	female	SAME1840159	PJL	Punjabi	SAS	South Asian Ancestry	PJL	1000 Genomes on GRCh38,1000 Genomes 30x on GRCh38,1000 Genomes phase 3 release
**NA18615**	female	SAME123653	CHB	Han Chinese	EAS	East Asian Ancestry	CHB	1000 Genomes on GRCh38,1000 Genomes 30x on GRCh38,1000 Genomes phase 3 release,1000 Genomes phase 1 release
**NA19173**	male	SAME124421	YRI	Yoruba	AFR	African Ancestry	YRI	1000 Genomes 30x on GRCh38,1000 Genomes phase 3 release
**HG02717**	male	SAME1839976	GWD	Gambian Mandinka	AFR	African Ancestry	GWD	1000 Genomes 30x on GRCh38
**NA18622**	male	SAME125260	CHB	Han Chinese	EAS	East Asian Ancestry	CHB	1000 Genomes on GRCh38,1000 Genomes 30x on GRCh38,1000 Genomes phase 3 release,1000 Genomes phase 1 release,90 Han Chinese high coverage genomes
**NA18858**	female	SAME124730	YRI	Yoruba	AFR	African Ancestry	YRI	1000 Genomes on GRCh38,1000 Genomes 30x on GRCh38,1000 Genomes phase 3 release,1000 Genomes phase 1 release,Geuvadis
**HG02724**	male	SAME1839740	PJL	Punjabi,Punjabi	SAS	South Asia (SGDP),South Asian Ancestry	PunjabiSGDP,PJL	1000 Genomes on GRCh38,Simons Genome Diversity Project,1000 Genomes 30x on GRCh38,1000 Genomes phase 3 release
**NA18627**	female	SAME124106	CHB	Han Chinese	EAS	East Asian Ancestry	CHB	1000 Genomes on GRCh38,1000 Genomes 30x on GRCh38,1000 Genomes phase 3 release,1000 Genomes phase 1 release
**NA18860**	male	SAME124906	YRI	Yoruba	AFR	African Ancestry	YRI	1000 Genomes 30x on GRCh38,1000 Genomes phase 3 release
**HG02729**	male	SAME1839715	PJL	Punjabi	SAS	South Asian Ancestry	PJL	1000 Genomes 30x on GRCh38,1000 Genomes phase 3 release
**NA18634**	female	SAME124697	CHB	Han Chinese	EAS	East Asian Ancestry	CHB	1000 Genomes on GRCh38,1000 Genomes 30x on GRCh38,1000 Genomes phase 3 release,1000 Genomes phase 1 release
**NA18865**	male	SAME124902	YRI	Yoruba	AFR	African Ancestry	YRI	1000 Genomes on GRCh38,1000 Genomes 30x on GRCh38,1000 Genomes phase 3 release
**HG02731**	female	SAME1839886	PJL	Punjabi	SAS	South Asian Ancestry	PJL	1000 Genomes on GRCh38,1000 Genomes 30x on GRCh38,1000 Genomes phase 3 release
**NA18639**	male	SAME124694	CHB	Han Chinese	EAS	East Asian Ancestry	CHB	1000 Genomes on GRCh38,1000 Genomes 30x on GRCh38,1000 Genomes phase 3 release,1000 Genomes phase 1 release
**NA18872**	male	SAME125099	YRI	Yoruba	AFR	African Ancestry	YRI	1000 Genomes 30x on GRCh38,1000 Genomes phase 3 release
**HG02736**	male	SAME1839860	PJL	Punjabi	SAS	South Asian Ancestry	PJL	1000 Genomes on GRCh38,1000 Genomes 30x on GRCh38,1000 Genomes phase 3 release
**NA18641**	female	SAME122979	CHB	Han Chinese	EAS	East Asian Ancestry	CHB	1000 Genomes on GRCh38,1000 Genomes 30x on GRCh38,1000 Genomes phase 3 release,1000 Genomes phase 1 release
**NA18877**	male	SAME125104	YRI	Yoruba	AFR	African Ancestry	YRI	1000 Genomes on GRCh38,1000 Genomes 30x on GRCh38,1000 Genomes phase 3 release
**HG02762**	male	SAME1839258	GWD	Gambian Mandinka	AFR	African Ancestry	GWD	1000 Genomes on GRCh38,1000 Genomes 30x on GRCh38,Gambian Genome Variation Project (GRCh38)
**NA18646**	female	SAME122978	CHB	Han Chinese	EAS	East Asian Ancestry	CHB	1000 Genomes on GRCh38,1000 Genomes 30x on GRCh38,1000 Genomes phase 3 release
**NA18909**	female	SAME123561	YRI	Yoruba	AFR	African Ancestry	YRI	1000 Genomes on GRCh38,1000 Genomes 30x on GRCh38,1000 Genomes phase 3 release,1000 Genomes phase 1 release,Geuvadis
**HG02774**	male	SAME1839028	PJL	Punjabi	SAS	South Asian Ancestry	PJL	1000 Genomes on GRCh38,1000 Genomes 30x on GRCh38,1000 Genomes phase 3 release
**NA18747**	male	SAME123916	CHB	Han Chinese	EAS	East Asian Ancestry	CHB	1000 Genomes on GRCh38,1000 Genomes 30x on GRCh38,1000 Genomes phase 3 release,1000 Genomes phase 1 release
**NA18911**	male	SAME123401	YRI	Yoruba	AFR	African Ancestry	YRI	1000 Genomes 30x on GRCh38,1000 Genomes phase 3 release
**NA19031**	male	SAME123543	LWK	Luhya	AFR	African Ancestry	LWK	1000 Genomes on GRCh38,1000 Genomes 30x on GRCh38,1000 Genomes phase 3 release,1000 Genomes phase 1 release
**NA19248**	male	SAME123681	YRI	Yoruba	AFR	African Ancestry	YRI	1000 Genomes on GRCh38,1000 Genomes 30x on GRCh38,1000 Genomes phase 3 release,1000 Genomes phase 1 release,Geuvadis
**HG02820**	female	SAME1839591	GWD	Gambian Mandinka	AFR	African Ancestry	GWD	1000 Genomes on GRCh38,1000 Genomes 30x on GRCh38,1000 Genomes phase 3 release,Gambian Genome Variation Project (GRCh38)
**NA19036**	female	SAME124666	LWK	Luhya	AFR	African Ancestry	LWK	1000 Genomes on GRCh38,1000 Genomes 30x on GRCh38,Human Genome Structural Variation Consortium, Phase 2,1000 Genomes phase 3 release,1000 Genomes ph
**HG02837**	female	SAME1839422	GWD	Gambian Mandinka	AFR	African Ancestry	GWD	1000 Genomes on GRCh38,1000 Genomes 30x on GRCh38,1000 Genomes phase 3 release,Gambian Genome Variation Project (GRCh38)
**NA19043**	male	SAME125243	LWK	Luhya	AFR	African Ancestry	LWK	1000 Genomes on GRCh38,1000 Genomes 30x on GRCh38,1000 Genomes phase 3 release
**NA19318**	male	SAME124753	LWK	Luhya	AFR	African Ancestry	LWK	1000 Genomes on GRCh38,1000 Genomes 30x on GRCh38,1000 Genomes phase 3 release,1000 Genomes phase 1 release
**HG02851**	male	SAME1839352	GWD	Gambian Mandinka	AFR	African Ancestry	GWD	1000 Genomes on GRCh38,1000 Genomes 30x on GRCh38,1000 Genomes phase 3 release,Gambian Genome Variation Project (GRCh38)
**NA19055**	male	SAME125027	JPT	Japanese	EAS	East Asian Ancestry	JPT	1000 Genomes on GRCh38,1000 Genomes 30x on GRCh38,1000 Genomes phase 3 release,1000 Genomes phase 1 release
**NA19320**	female	SAME124570	LWK	Luhya	AFR	African Ancestry	LWK	1000 Genomes on GRCh38,1000 Genomes 30x on GRCh38,1000 Genomes phase 3 release
**HG02856**	male	SAME1839327	GWD	Gambian Mandinka	AFR	African Ancestry	GWD	1000 Genomes 30x on GRCh38
**NA19062**	male	SAME123005	JPT	Japanese	EAS	East Asian Ancestry	JPT	1000 Genomes on GRCh38,1000 Genomes 30x on GRCh38,1000 Genomes phase 3 release,1000 Genomes phase 1 release
**NA19332**	female	SAME123723	LWK	Luhya	AFR	African Ancestry	LWK	1000 Genomes on GRCh38,1000 Genomes 30x on GRCh38,1000 Genomes phase 3 release,1000 Genomes phase 1 release
**HG02870**	female	SAME1840320	GWD	Gambian Mandinka	AFR	African Ancestry	GWD	1000 Genomes on GRCh38,1000 Genomes 30x on GRCh38,1000 Genomes phase 3 release,Gambian Genome Variation Project (GRCh38)
**NA19067**	male	SAME123835	JPT	Japanese	EAS	East Asian Ancestry	JPT	1000 Genomes on GRCh38,1000 Genomes 30x on GRCh38,1000 Genomes phase 3 release,1000 Genomes phase 1 release
**NA19351**	female	SAME122854	LWK	Luhya	AFR	African Ancestry	LWK	1000 Genomes on GRCh38,1000 Genomes 30x on GRCh38,1000 Genomes phase 3 release,1000 Genomes phase 1 release
**HG02882**	female	SAME1840171	GWD	Gambian Mandinka	AFR	African Ancestry	GWD	1000 Genomes on GRCh38,1000 Genomes 30x on GRCh38,1000 Genomes phase 3 release,Gambian Genome Variation Project (GRCh38)
**NA19074**	female	SAME122817	JPT	Japanese	EAS	East Asian Ancestry	JPT	1000 Genomes on GRCh38,1000 Genomes 30x on GRCh38,1000 Genomes phase 3 release,1000 Genomes phase 1 release
**NA19375**	male	SAME125116	LWK	Luhya	AFR	African Ancestry	LWK	1000 Genomes on GRCh38,1000 Genomes 30x on GRCh38,1000 Genomes phase 3 release,1000 Genomes phase 1 release
**HG02887**	male	SAME1840147	GWD	Gambian Mandinka	AFR	African Ancestry	GWD	1000 Genomes on GRCh38,1000 Genomes 30x on GRCh38,1000 Genomes phase 3 release,Gambian Genome Variation Project (GRCh38)
**NA19079**	male	SAME122823	JPT	Japanese	EAS	East Asian Ancestry	JPT	1000 Genomes on GRCh38,1000 Genomes 30x on GRCh38,1000 Genomes phase 3 release,1000 Genomes phase 1 release
**NA18980**	female	SAME123527	JPT	Japanese	EAS	East Asian Ancestry	JPT	1000 Genomes on GRCh38,1000 Genomes 30x on GRCh38,1000 Genomes phase 3 release,1000 Genomes phase 1 release
**HG03025**	female	SAME1839711	GWD	Gambian Mandinka	AFR	African Ancestry	GWD	1000 Genomes on GRCh38,1000 Genomes 30x on GRCh38,1000 Genomes phase 3 release,Gambian Genome Variation Project (GRCh38)
**NA19081**	female	SAME123369	JPT	Japanese	EAS	East Asian Ancestry	JPT	1000 Genomes on GRCh38,1000 Genomes 30x on GRCh38,1000 Genomes phase 3 release,1000 Genomes phase 1 release
**NA18985**	male	SAME123413	JPT	Japanese	EAS	East Asian Ancestry	JPT	1000 Genomes on GRCh38,1000 Genomes 30x on GRCh38,1000 Genomes phase 3 release,1000 Genomes phase 1 release
**HG03049**	female	SAME1839653	GWD	Gambian Mandinka	AFR	African Ancestry	GWD	1000 Genomes on GRCh38,1000 Genomes 30x on GRCh38,1000 Genomes phase 3 release,Gambian Genome Variation Project (GRCh38)
**NA19086**	male	SAME123366	JPT	Japanese	EAS	East Asian Ancestry	JPT	1000 Genomes on GRCh38,1000 Genomes 30x on GRCh38,1000 Genomes phase 3 release,1000 Genomes phase 1 release
**NA18992**	female	SAME124983	JPT	Japanese	EAS	East Asian Ancestry	JPT	1000 Genomes on GRCh38,1000 Genomes 30x on GRCh38,1000 Genomes phase 3 release,1000 Genomes phase 1 release
**HG03056**	male	SAME1839518	MSL	Mende	AFR	African Ancestry	MSL	1000 Genomes 30x on GRCh38
**NA19093**	female	SAME123172	YRI	Yoruba	AFR	African Ancestry	YRI	1000 Genomes on GRCh38,1000 Genomes 30x on GRCh38,1000 Genomes phase 3 release,1000 Genomes phase 1 release,Geuvadis
**NA18997**	female	SAME124898	JPT	Japanese	EAS	East Asian Ancestry	JPT	1000 Genomes on GRCh38,1000 Genomes 30x on GRCh38,1000 Genomes phase 3 release,1000 Genomes phase 1 release
**HG02938**	male	SAME1839284	ESN	Esan	AFR	African Ancestry	ESN	1000 Genomes on GRCh38,1000 Genomes 30x on GRCh38,1000 Genomes phase 3 release
**NA19098**	male	SAME123633	YRI	Yoruba	AFR	African Ancestry	YRI	1000 Genomes on GRCh38,1000 Genomes 30x on GRCh38,1000 Genomes phase 3 release,1000 Genomes phase 1 release,Geuvadis
**NA19003**	female	SAME124480	JPT	Japanese	EAS	East Asian Ancestry	JPT	1000 Genomes on GRCh38,1000 Genomes 30x on GRCh38,1000 Genomes phase 3 release,1000 Genomes phase 1 release
**HG02945**	female	SAME1839075	ESN	Esan	AFR	African Ancestry	ESN	1000 Genomes 30x on GRCh38
**NA12890**	female	SAME124116	CEU	CEPH	EUR	European Ancestry	CEU	1000 Genomes on GRCh38,1000 Genomes 30x on GRCh38,Illumina Platinum pedigree,1000 Genomes phase 3 release,1000 Genomes phase 1 release,Geuvadis
**NA19010**	female	SAME123989	JPT	Japanese	EAS	East Asian Ancestry	JPT	1000 Genomes on GRCh38,1000 Genomes 30x on GRCh38,1000 Genomes phase 3 release,1000 Genomes phase 1 release
**HG02952**	female	SAME1840275	ESN	Esan	AFR	African Ancestry	ESN	1000 Genomes on GRCh38,1000 Genomes 30x on GRCh38,1000 Genomes phase 3 release
**NA18487**	male	SAME124098	YRI	Yoruba	AFR	African Ancestry	YRI	1000 Genomes on GRCh38,1000 Genomes 30x on GRCh38,1000 Genomes phase 3 release,1000 Genomes phase 1 release,Geuvadis
**NA19027**	male	SAME124850	LWK	Luhya	AFR	African Ancestry	LWK	1000 Genomes on GRCh38,1000 Genomes 30x on GRCh38,1000 Genomes phase 3 release,1000 Genomes phase 1 release
**HG02964**	female	SAME1840122	ESN	Esan	AFR	African Ancestry	ESN	1000 Genomes on GRCh38,1000 Genomes 30x on GRCh38
**NA18499**	female	SAME124059	YRI	Yoruba	AFR	African Ancestry	YRI	1000 Genomes on GRCh38,1000 Genomes 30x on GRCh38,1000 Genomes phase 3 release,1000 Genomes phase 1 release,Geuvadis
**NA19041**	male	SAME125241	LWK	Luhya	AFR	African Ancestry	LWK	1000 Genomes on GRCh38,1000 Genomes 30x on GRCh38,1000 Genomes phase 3 release,1000 Genomes phase 1 release
**NA18502**	female	SAME125055	YRI	Yoruba	AFR	African Ancestry	YRI	1000 Genomes on GRCh38,1000 Genomes 30x on GRCh38,1000 Genomes phase 3 release,1000 Genomes phase 1 release,Geuvadis
**NA19185**	female	SAME123858	YRI	Yoruba	AFR	African Ancestry	YRI	1000 Genomes on GRCh38,1000 Genomes 30x on GRCh38,1000 Genomes phase 3 release,1000 Genomes phase 1 release,Geuvadis
**HG03239**	female	SAME1839295	PJL	Punjabi	SAS	South Asian Ancestry	PJL	1000 Genomes 30x on GRCh38,1000 Genomes phase 3 release
**NA18507**	male	SAME125060	YRI	Yoruba	AFR	African Ancestry	YRI	1000 Genomes on GRCh38,1000 Genomes 30x on GRCh38,1000 Genomes phase 3 release,1000 Genomes phase 1 release
**NA19197**	female	SAME125309	YRI	Yoruba	AFR	African Ancestry	YRI	1000 Genomes on GRCh38,1000 Genomes 30x on GRCh38,1000 Genomes phase 3 release,1000 Genomes phase 1 release,Geuvadis
**HG03241**	female	SAME1839053	GWD	Gambian Mandinka	AFR	African Ancestry	GWD	1000 Genomes on GRCh38,1000 Genomes 30x on GRCh38,1000 Genomes phase 3 release,Gambian Genome Variation Project (GRCh38)
**NA18519**	male	SAME124888	YRI	Yoruba	AFR	African Ancestry	YRI	1000 Genomes on GRCh38,1000 Genomes 30x on GRCh38,1000 Genomes phase 3 release,1000 Genomes phase 1 release,Geuvadis
**NA19200**	male	SAME122989	YRI	Yoruba	AFR	African Ancestry	YRI	1000 Genomes on GRCh38,1000 Genomes 30x on GRCh38,1000 Genomes phase 3 release,1000 Genomes phase 1 release,Geuvadis
**HG03246**	male	SAME1839168	GWD	Gambian Mandinka	AFR	African Ancestry	GWD	1000 Genomes on GRCh38,1000 Genomes 30x on GRCh38,1000 Genomes phase 3 release,Gambian Genome Variation Project (GRCh38)
**NA18521**	male	SAME123566	YRI	Yoruba	AFR	African Ancestry	YRI	1000 Genomes 30x on GRCh38,1000 Genomes phase 3 release
**NA19205**	male	SAME122986	YRI	Yoruba	AFR	African Ancestry	YRI	1000 Genomes 30x on GRCh38,1000 Genomes phase 3 release
**HG03258**	male	SAME1840296	GWD	Gambian Mandinka	AFR	African Ancestry	GWD	1000 Genomes on GRCh38,1000 Genomes 30x on GRCh38,1000 Genomes phase 3 release,Gambian Genome Variation Project (GRCh38)
**NA18526**	female	SAME124673	CHB	Han Chinese	EAS	East Asian Ancestry	CHB	1000 Genomes on GRCh38,1000 Genomes 30x on GRCh38,1000 Genomes phase 3 release,1000 Genomes phase 1 release,90 Han Chinese high coverage genomes
**NA19224**	male	SAME125217	YRI	Yoruba	AFR	African Ancestry	YRI	1000 Genomes 30x on GRCh38,1000 Genomes phase 3 release
**HG03260**	male	SAME1840140	GWD	Gambian Mandinka	AFR	African Ancestry	GWD	1000 Genomes 30x on GRCh38
**NA18533**	female	SAME124481	CHB	Han Chinese	EAS	East Asian Ancestry	CHB	1000 Genomes on GRCh38,1000 Genomes 30x on GRCh38,1000 Genomes phase 3 release
**NA19236**	male	SAME125001	YRI	Yoruba	AFR	African Ancestry	YRI	1000 Genomes on GRCh38,1000 Genomes 30x on GRCh38,1000 Genomes phase 3 release,1000 Genomes phase 1 release,Geuvadis

**HG03265**	male	SAME1840167	ESN	Esan	AFR	African Ancestry	ESN	1000 Genomes on GRCh38,1000 Genomes 30x on GRCh38,1000 Genomes phase 3 release
**NA18968**	female	SAME123953	JPT	Japanese	EAS	East Asian Ancestry	JPT	1000 Genomes on GRCh38,1000 Genomes 30x on GRCh38,1000 Genomes phase 3 release,1000 Genomes phase 1 release
**NA20517**	female	SAME124557	TSI	Toscani	EUR	European Ancestry	TSI	1000 Genomes on GRCh38,1000 Genomes 30x on GRCh38,1000 Genomes phase 3 release,1000 Genomes phase 1 release,Geuvadis
**HG03272**	female	SAME1840199	ESN	Esan	AFR	African Ancestry	ESN	1000 Genomes 30x on GRCh38
**NA18970**	male	SAME123764	JPT	Japanese	EAS	East Asian Ancestry	JPT	1000 Genomes on GRCh38,1000 Genomes 30x on GRCh38,1000 Genomes phase 3 release,1000 Genomes phase 1 release
**NA20524**	male	SAME124016	TSI	Toscani	EUR	European Ancestry	TSI	1000 Genomes on GRCh38,1000 Genomes 30x on GRCh38,1000 Genomes phase 3 release,1000 Genomes phase 1 release,Geuvadis
**HG03291**	female	SAME1839861	ESN	Esan	AFR	African Ancestry	ESN	1000 Genomes on GRCh38,1000 Genomes 30x on GRCh38,1000 Genomes phase 3 release
**NA18975**	female	SAME123767	JPT	Japanese	EAS	East Asian Ancestry	JPT	1000 Genomes on GRCh38,1000 Genomes 30x on GRCh38,1000 Genomes phase 3 release,1000 Genomes phase 1 release
**NA20529**	female	SAME124011	TSI	Toscani	EUR	European Ancestry	TSI	1000 Genomes on GRCh38,1000 Genomes 30x on GRCh38,1000 Genomes phase 3 release,1000 Genomes phase 1 release,Geuvadis
**HG03342**	female	SAME1840350	ESN	Esan	AFR	African Ancestry	ESN	1000 Genomes on GRCh38,1000 Genomes 30x on GRCh38,1000 Genomes phase 3 release
**NA18982**	male	SAME125073	JPT	Japanese	EAS	East Asian Ancestry	JPT	1000 Genomes on GRCh38,1000 Genomes 30x on GRCh38,1000 Genomes phase 3 release,1000 Genomes phase 1 release
**NA20531**	female	SAME124170	TSI	Toscani	EUR	European Ancestry	TSI	1000 Genomes on GRCh38,1000 Genomes 30x on GRCh38,1000 Genomes phase 3 release,1000 Genomes phase 1 release,Geuvadis
**HG03354**	female	SAME1840249	ESN	Esan	AFR	African Ancestry	ESN	1000 Genomes on GRCh38,1000 Genomes 30x on GRCh38,1000 Genomes phase 3 release
**NA18987**	female	SAME125070	JPT	Japanese	EAS	East Asian Ancestry	JPT	1000 Genomes on GRCh38,1000 Genomes 30x on GRCh38,1000 Genomes phase 3 release,1000 Genomes phase 1 release
**NA20536**	male	SAME124167	TSI	Toscani	EUR	European Ancestry	TSI	1000 Genomes on GRCh38,1000 Genomes 30x on GRCh38,1000 Genomes phase 3 release,1000 Genomes phase 1 release,Geuvadis
**NA18994**	male	SAME124897	JPT	Japanese	EAS	East Asian Ancestry	JPT	1000 Genomes on GRCh38,1000 Genomes 30x on GRCh38,1000 Genomes phase 3 release,1000 Genomes phase 1 release
**NA20543**	male	SAME125149	TSI	Toscani	EUR	European Ancestry	TSI	1000 Genomes on GRCh38,1000 Genomes 30x on GRCh38,1000 Genomes phase 3 release,1000 Genomes phase 1 release,Geuvadis
**HG03366**	female	SAME1840066	ESN	Esan	AFR	African Ancestry	ESN	1000 Genomes on GRCh38,1000 Genomes 30x on GRCh38,1000 Genomes phase 3 release
**NA18999**	female	SAME124890	JPT	Japanese	EAS	East Asian Ancestry	JPT	1000 Genomes on GRCh38,1000 Genomes 30x on GRCh38,1000 Genomes phase 3 release,1000 Genomes phase 1 release
**NA20581**	male	SAME122859	TSI	Toscani	EUR	European Ancestry	TSI	1000 Genomes on GRCh38,1000 Genomes 30x on GRCh38,1000 Genomes phase 3 release,1000 Genomes phase 1 release,Geuvadis
**HG03373**	male	SAME1839720	ESN	Esan	AFR	African Ancestry	ESN	1000 Genomes on GRCh38,1000 Genomes 30x on GRCh38
**NA19000**	male	SAME124477	JPT	Japanese	EAS	East Asian Ancestry	JPT	1000 Genomes on GRCh38,1000 Genomes 30x on GRCh38,1000 Genomes phase 3 release,1000 Genomes phase 1 release
**NA20586**	male	SAME122864	TSI	Toscani	EUR	European Ancestry	TSI	1000 Genomes on GRCh38,1000 Genomes 30x on GRCh38,1000 Genomes phase 3 release,1000 Genomes phase 1 release,Geuvadis
**HG03164**	female	SAME1839121	ESN	Esan	AFR	African Ancestry	ESN	1000 Genomes 30x on GRCh38
**NA19005**	male	SAME124474	JPT	Japanese	EAS	East Asian Ancestry	JPT	1000 Genomes on GRCh38,1000 Genomes 30x on GRCh38,1000 Genomes phase 3 release,1000 Genomes phase 1 release
**NA20752**	male	SAME124177	TSI	Toscani	EUR	European Ancestry	TSI	1000 Genomes on GRCh38,1000 Genomes 30x on GRCh38,1000 Genomes phase 3 release,1000 Genomes phase 1 release,Geuvadis
**HG03169**	male	SAME1839148	ESN	Esan	AFR	African Ancestry	ESN	1000 Genomes on GRCh38,1000 Genomes 30x on GRCh38,1000 Genomes phase 3 release
**NA19012**	male	SAME124260	JPT	Japanese	EAS	East Asian Ancestry	JPT	1000 Genomes on GRCh38,1000 Genomes 30x on GRCh38,1000 Genomes phase 3 release,1000 Genomes phase 1 release
**NA20757**	female	SAME124176	TSI	Toscani	EUR	European Ancestry	TSI	1000 Genomes on GRCh38,1000 Genomes 30x on GRCh38,1000 Genomes phase 3 release,1000 Genomes phase 1 release,Geuvadis
**NA19017**	female	SAME124259	LWK	Luhya	AFR	African Ancestry	LWK	1000 Genomes on GRCh38,1000 Genomes 30x on GRCh38,1000 Genomes phase 3 release
**NA20764**	female	SAME124368	TSI	Toscani	EUR	European Ancestry	TSI	1000 Genomes on GRCh38,1000 Genomes 30x on GRCh38,1000 Genomes phase 3 release
**NA19024**	female	SAME124851	LWK	Luhya	AFR	African Ancestry	LWK	1000 Genomes on GRCh38,1000 Genomes 30x on GRCh38,1000 Genomes phase 3 release
**NA20769**	female	SAME124371	TSI	Toscani	EUR	European Ancestry	TSI	1000 Genomes on GRCh38,1000 Genomes 30x on GRCh38,1000 Genomes phase 3 release,1000 Genomes phase 1 release,Geuvadis
**HG03190**	male	SAME1839984	ESN	Esan	AFR	African Ancestry	ESN	1000 Genomes on GRCh38,1000 Genomes 30x on GRCh38,1000 Genomes phase 3 release
**NA12718**	female	SAME124454	CEU	CEPH	EUR	European Ancestry	CEU	1000 Genomes on GRCh38,1000 Genomes 30x on GRCh38,1000 Genomes phase 3 release,1000 Genomes phase 1 release,Geuvadis
**NA20771**	female	SAME124577	TSI	Toscani	EUR	European Ancestry	TSI	1000 Genomes on GRCh38,1000 Genomes 30x on GRCh38,1000 Genomes phase 3 release,1000 Genomes phase 1 release,Geuvadis
**HG03121**	female	SAME1839563	ESN	Esan	AFR	African Ancestry	ESN	1000 Genomes on GRCh38,1000 Genomes 30x on GRCh38,1000 Genomes phase 3 release
**NA12749**	female	SAME124828	CEU	CEPH	EUR	European Ancestry	CEU	1000 Genomes on GRCh38,1000 Genomes 30x on GRCh38,1000 Genomes phase 3 release,1000 Genomes phase 1 release,Geuvadis
**HG03126**	female	SAME1839683	ESN	Esan	AFR	African Ancestry	ESN	1000 Genomes on GRCh38,1000 Genomes 30x on GRCh38,1000 Genomes phase 3 release
**NA12751**	female	SAME123515	CEU	CEPH	EUR	European Ancestry	CEU	1000 Genomes on GRCh38,1000 Genomes 30x on GRCh38,1000 Genomes phase 3 release,1000 Genomes phase 1 release,Geuvadis
**NA19818**	male	SAME124677	ASW	African Ancestry SW	AFR	African Ancestry	ASW	1000 Genomes on GRCh38,1000 Genomes 30x on GRCh38,1000 Genomes phase 3 release,1000 Genomes phase 1 release
**HG03133**	male	SAME1839375	ESN	Esan	AFR	African Ancestry	ESN	1000 Genomes on GRCh38,1000 Genomes 30x on GRCh38,1000 Genomes phase 3 release
**NA12763**	female	SAME123872	CEU	CEPH	EUR	European Ancestry	CEU	1000 Genomes on GRCh38,1000 Genomes 30x on GRCh38,1000 Genomes phase 3 release,1000 Genomes phase 1 release,Geuvadis
**NA19902**	female	SAME125051	ASW	African Ancestry SW	AFR	African Ancestry	ASW	1000 Genomes 30x on GRCh38,1000 Genomes phase 3 release
**NA12775**	male	SAME124257	CEU	CEPH	EUR	European Ancestry	CEU	1000 Genomes on GRCh38,1000 Genomes 30x on GRCh38,1000 Genomes phase 3 release,1000 Genomes phase 1 release,Geuvadis
**NA19914**	female	SAME125273	ASW	African Ancestry SW	AFR	African Ancestry	ASW	1000 Genomes on GRCh38,1000 Genomes 30x on GRCh38,1000 Genomes phase 3 release,1000 Genomes phase 1 release
**HG03157**	male	SAME1839083	ESN	Esan	AFR	African Ancestry	ESN	1000 Genomes on GRCh38,1000 Genomes 30x on GRCh38,1000 Genomes phase 3 release
**NA12802**	female	SAME122828	CEU	CEPH	EUR	European Ancestry	CEU	1000 Genomes 30x on GRCh38,1000 Genomes phase 3 release
**NA19919**	male	SAME125276	ASW	African Ancestry SW	AFR	African Ancestry	ASW	1000 Genomes 30x on GRCh38,1000 Genomes phase 3 release
**HG03296**	male	SAME1839887	ESN	Esan	AFR	African Ancestry	ESN	1000 Genomes 30x on GRCh38
**NA12814**	male	SAME123021	CEU	CEPH	EUR	European Ancestry	CEU	1000 Genomes on GRCh38,1000 Genomes 30x on GRCh38,1000 Genomes phase 3 release,1000 Genomes phase 1 release,Geuvadis
**NA19921**	female	SAME124712	ASW	African Ancestry SW	AFR	African Ancestry	ASW	1000 Genomes on GRCh38,1000 Genomes 30x on GRCh38,1000 Genomes phase 3 release,1000 Genomes phase 1 release
**HG03304**	male	SAME1839490	ESN	Esan	AFR	African Ancestry	ESN	1000 Genomes on GRCh38,1000 Genomes 30x on GRCh38,1000 Genomes phase 3 release
**NA12864**	male	SAME123191	CEU	CEPH	EUR	European Ancestry	CEU	1000 Genomes 30x on GRCh38,1000 Genomes phase 3 release
**NA19983**	female	SAME123902	ASW	African Ancestry SW	AFR	African Ancestry	ASW	1000 Genomes 30x on GRCh38,Human Genome Structural Variation Consortium, Phase 2,1000 Genomes phase 3 release
**HG03309**	female	SAME1839520	ESN	Esan	AFR	African Ancestry	ESN	1000 Genomes on GRCh38,1000 Genomes 30x on GRCh38
**NA18879**	male	SAME125108	YRI	Yoruba	AFR	African Ancestry	YRI	1000 Genomes on GRCh38,1000 Genomes 30x on GRCh38,1000 Genomes phase 3 release
**NA20276**	female	SAME123614	ASW	African Ancestry SW	AFR	African Ancestry	ASW	1000 Genomes on GRCh38,1000 Genomes 30x on GRCh38,1000 Genomes phase 3 release,1000 Genomes phase 1 release
**HG03311**	male	SAME1839206	ESN	Esan	AFR	African Ancestry	ESN	1000 Genomes on GRCh38,1000 Genomes 30x on GRCh38,1000 Genomes phase 3 release
**NA18881**	female	SAME125308	YRI	Yoruba	AFR	African Ancestry	YRI	1000 Genomes on GRCh38,1000 Genomes 30x on GRCh38,1000 Genomes phase 3 release
**NA20783**	male	SAME123607	TSI	Toscani	EUR	European Ancestry	TSI	1000 Genomes on GRCh38,1000 Genomes 30x on GRCh38,1000 Genomes phase 3 release,1000 Genomes phase 1 release,Geuvadis
**HG03195**	female	SAME1839919	ESN	Esan	AFR	African Ancestry	ESN	1000 Genomes on GRCh38,1000 Genomes 30x on GRCh38,1000 Genomes phase 3 release
**NA18906**	female	SAME123562	YRI	Yoruba	AFR	African Ancestry	YRI	1000 Genomes 30x on GRCh38,1000 Genomes phase 3 release
**NA20790**	female	SAME123815	TSI	Toscani	EUR	European Ancestry	TSI	1000 Genomes on GRCh38,1000 Genomes 30x on GRCh38,1000 Genomes phase 3 release,1000 Genomes phase 1 release,Geuvadis
**NA18913**	male	SAME123403	YRI	Yoruba	AFR	African Ancestry	YRI	1000 Genomes 30x on GRCh38,1000 Genomes phase 3 release
**NA20795**	female	SAME123818	TSI	Toscani	EUR	European Ancestry	TSI	1000 Genomes on GRCh38,1000 Genomes 30x on GRCh38,1000 Genomes phase 3 release,1000 Genomes phase 1 release,Geuvadis
**HG03234**	male	SAME1839357	PJL	Punjabi	SAS	South Asian Ancestry	PJL	1000 Genomes on GRCh38,1000 Genomes 30x on GRCh38,1000 Genomes phase 3 release
**NA18925**	male	SAME123203	YRI	Yoruba	AFR	African Ancestry	YRI	1000 Genomes 30x on GRCh38,1000 Genomes phase 3 release
**NA20803**	male	SAME125367	TSI	Toscani	EUR	European Ancestry	TSI	1000 Genomes on GRCh38,1000 Genomes 30x on GRCh38,1000 Genomes phase 3 release,1000 Genomes phase 1 release,Geuvadis
**HG03099**	female	SAME1840146	ESN	Esan	AFR	African Ancestry	ESN	1000 Genomes on GRCh38,1000 Genomes 30x on GRCh38,1000 Genomes phase 3 release
**NA18944**	male	SAME124314	JPT	Japanese	EAS	East Asian Ancestry	JPT	1000 Genomes on GRCh38,1000 Genomes 30x on GRCh38,1000 Genomes phase 3 release,1000 Genomes phase 1 release
**NA20808**	female	SAME122813	TSI	Toscani	EUR	European Ancestry	TSI	1000 Genomes on GRCh38,1000 Genomes 30x on GRCh38,1000 Genomes phase 3 release,1000 Genomes phase 1 release,Geuvadis
**NA18949**	female	SAME124317	JPT	Japanese	EAS	East Asian Ancestry	JPT	1000 Genomes on GRCh38,1000 Genomes 30x on GRCh38,1000 Genomes phase 3 release,1000 Genomes phase 1 release
**NA20810**	male	SAME125192	TSI	Toscani	EUR	European Ancestry	TSI	1000 Genomes on GRCh38,1000 Genomes 30x on GRCh38,1000 Genomes phase 3 release,1000 Genomes phase 1 release,Geuvadis
**HG03114**	female	SAME1839907	ESN	Esan	AFR	African Ancestry	ESN	1000 Genomes on GRCh38,1000 Genomes 30x on GRCh38,1000 Genomes phase 3 release
**NA18951**	female	SAME124111	JPT	Japanese	EAS	East Asian Ancestry	JPT	1000 Genomes on GRCh38,1000 Genomes 30x on GRCh38,1000 Genomes phase 3 release,1000 Genomes phase 1 release
**NA20815**	male	SAME125187	TSI	Toscani	EUR	European Ancestry	TSI	1000 Genomes on GRCh38,1000 Genomes 30x on GRCh38,1000 Genomes phase 3 release,1000 Genomes phase 1 release,Geuvadis
**HG03119**	female	SAME1839833	ESN	Esan	AFR	African Ancestry	ESN	1000 Genomes 30x on GRCh38
**NA18956**	female	SAME124110	JPT	Japanese	EAS	East Asian Ancestry	JPT	1000 Genomes on GRCh38,1000 Genomes 30x on GRCh38,1000 Genomes phase 3 release,1000 Genomes phase 1 release
**NA20822**	female	SAME123073	TSI	Toscani	EUR	European Ancestry	TSI	1000 Genomes on GRCh38,1000 Genomes 30x on GRCh38,1000 Genomes phase 3 release
**HG03378**	female	SAME1839743	MSL	Mende	AFR	African Ancestry	MSL	1000 Genomes on GRCh38,1000 Genomes 30x on GRCh38,1000 Genomes phase 3 release
**NA18963**	female	SAME123959	JPT	Japanese	EAS	East Asian Ancestry	JPT	1000 Genomes on GRCh38,1000 Genomes 30x on GRCh38,1000 Genomes phase 3 release,1000 Genomes phase 1 release
**NA20827**	male	SAME123071	TSI	Toscani	EUR	European Ancestry	TSI	1000 Genomes on GRCh38,1000 Genomes 30x on GRCh38,1000 Genomes phase 3 release
**HG03380**	female	SAME1839569	MSL	Mende	AFR	African Ancestry	MSL	1000 Genomes on GRCh38,1000 Genomes 30x on GRCh38,1000 Genomes phase 3 release
**NA19101**	male	SAME123955	YRI	Yoruba	AFR	African Ancestry	YRI	1000 Genomes 30x on GRCh38,1000 Genomes phase 3 release
**NA20846**	male	SAME123431	GIH	Gujarati	SAS	South Asian Ancestry	GIH	1000 Genomes on GRCh38,1000 Genomes 30x on GRCh38,1000 Genomes phase 3 release
**HG03385**	male	SAME1839594	MSL	Mende	AFR	African Ancestry	MSL	1000 Genomes on GRCh38,1000 Genomes 30x on GRCh38,1000 Genomes phase 3 release
**NA19113**	male	SAME123838	YRI	Yoruba	AFR	African Ancestry	YRI	1000 Genomes on GRCh38,1000 Genomes 30x on GRCh38,1000 Genomes phase 3 release,1000 Genomes phase 1 release,Geuvadis
**NA20853**	female	SAME123243	GIH	Gujarati	SAS	South Asian Ancestry	GIH	1000 Genomes on GRCh38,1000 Genomes 30x on GRCh38,1000 Genomes phase 3 release
**HG03397**	male	SAME1839400	MSL	Mende	AFR	African Ancestry	MSL	1000 Genomes on GRCh38,1000 Genomes 30x on GRCh38,1000 Genomes phase 3 release
**NA19118**	female	SAME122943	YRI	Yoruba	AFR	African Ancestry	YRI	1000 Genomes on GRCh38,1000 Genomes 30x on GRCh38,1000 Genomes phase 3 release,1000 Genomes phase 1 release,Geuvadis
**HG03063**	male	SAME1839226	MSL	Mende	AFR	African Ancestry	MSL	1000 Genomes on GRCh38,1000 Genomes 30x on GRCh38,1000 Genomes phase 3 release
**NA19120**	male	SAME125016	YRI	Yoruba	AFR	African Ancestry	YRI	1000 Genomes 30x on GRCh38,1000 Genomes phase 3 release
**NA19471**	female	SAME123117	LWK	Luhya	AFR	African Ancestry	LWK	1000 Genomes on GRCh38,1000 Genomes 30x on GRCh38,1000 Genomes phase 3 release,1000 Genomes phase 1 release
**HG03082**	female	SAME1840316	MSL	Mende	AFR	African Ancestry	MSL	1000 Genomes on GRCh38,1000 Genomes 30x on GRCh38,1000 Genomes phase 3 release
**NA19132**	female	SAME125231	YRI	Yoruba	AFR	African Ancestry	YRI	1000 Genomes 30x on GRCh38,1000 Genomes phase 3 release
**NA19654**	female	SAME123128	MXL	Mexican Ancestry	AMR	American Ancestry	MXL	1000 Genomes on GRCh38,1000 Genomes 30x on GRCh38,1000 Genomes phase 3 release,1000 Genomes phase 1 release
**HG03486**	female	SAME1839474	MSL	Mende	AFR	African Ancestry	MSL	1000 Genomes 30x on GRCh38,Human Genome Structural Variation Consortium, Phase 2,1000 Genomes phase 3 release
**NA19137**	female	SAME125235	YRI	Yoruba	AFR	African Ancestry	YRI	1000 Genomes on GRCh38,1000 Genomes 30x on GRCh38,1000 Genomes phase 3 release,1000 Genomes phase 1 release,Geuvadis
**NA19659**	female	SAME123131	MXL	Mexican Ancestry	AMR	American Ancestry	MXL	1000 Genomes 30x on GRCh38,1000 Genomes phase 3 release
**NA19144**	male	SAME123373	YRI	Yoruba	AFR	African Ancestry	YRI	1000 Genomes on GRCh38,1000 Genomes 30x on GRCh38,1000 Genomes phase 3 release,1000 Genomes phase 1 release,Geuvadis
**NA19661**	male	SAME122959	MXL	Mexican Ancestry	AMR	American Ancestry	MXL	1000 Genomes on GRCh38,1000 Genomes 30x on GRCh38,1000 Genomes phase 3 release,1000 Genomes phase 1 release
**NA19149**	female	SAME124605	YRI	Yoruba	AFR	African Ancestry	YRI	1000 Genomes on GRCh38,1000 Genomes 30x on GRCh38,1000 Genomes phase 3 release,1000 Genomes phase 1 release,Geuvadis
**NA19678**	female	SAME122797	MXL	Mexican Ancestry	AMR	American Ancestry	MXL	1000 Genomes on GRCh38,1000 Genomes 30x on GRCh38,1000 Genomes phase 3 release,1000 Genomes phase 1 release
**HG03518**	male	SAME1840169	ESN	Esan	AFR	African Ancestry	ESN	1000 Genomes on GRCh38,1000 Genomes 30x on GRCh38,1000 Genomes phase 3 release
**NA19151**	female	SAME124839	YRI	Yoruba	AFR	African Ancestry	YRI	1000 Genomes 30x on GRCh38,1000 Genomes phase 3 release
**NA19680**	female	SAME122937	MXL	Mexican Ancestry	AMR	American Ancestry	MXL	1000 Genomes 30x on GRCh38,1000 Genomes phase 3 release
**HG03520**	female	SAME1840186	ESN	Esan	AFR	African Ancestry	ESN	1000 Genomes on GRCh38,1000 Genomes 30x on GRCh38,1000 Genomes phase 3 release
**NA19175**	male	SAME124887	YRI	Yoruba	AFR	African Ancestry	YRI	1000 Genomes on GRCh38,1000 Genomes 30x on GRCh38,1000 Genomes phase 3 release,1000 Genomes phase 1 release,Geuvadis
**NA19685**	male	SAME122942	MXL	Mexican Ancestry	AMR	American Ancestry	MXL	1000 Genomes on GRCh38,1000 Genomes 30x on GRCh38,1000 Genomes phase 3 release,1000 Genomes phase 1 release
**HG03607**	female	SAME1840254	BEB	Bengali	SAS	South Asian Ancestry	BEB	1000 Genomes on GRCh38,1000 Genomes 30x on GRCh38,1000 Genomes phase 3 release

**NA19199**	female	SAME124050	YRI	Yoruba	AFR	African Ancestry	YRI	1000 Genomes 30x on GRCh38,1000 Genomes phase 3 release
**NA19700**	male	SAME124233	ASW	African Ancestry SW	AFR	African Ancestry	ASW	1000 Genomes on GRCh38,1000 Genomes 30x on GRCh38,1000 Genomes phase 3 release,1000 Genomes phase 1 release
**HG03619**	female	SAME1840064	PJL	Punjabi	SAS	South Asian Ancestry	PJL	1000 Genomes on GRCh38,1000 Genomes 30x on GRCh38,1000 Genomes phase 3 release
**NA19202**	female	SAME122987	YRI	Yoruba	AFR	African Ancestry	YRI	1000 Genomes 30x on GRCh38,1000 Genomes phase 3 release
**NA19705**	male	SAME124235	ASW	African Ancestry SW	AFR	African Ancestry	ASW	1000 Genomes 30x on GRCh38,1000 Genomes phase 3 release
**NA19207**	male	SAME122984	YRI	Yoruba	AFR	African Ancestry	YRI	1000 Genomes on GRCh38,1000 Genomes 30x on GRCh38,1000 Genomes phase 3 release,1000 Genomes phase 1 release,Geuvadis
**NA19712**	female	SAME124447	ASW	African Ancestry SW	AFR	African Ancestry	ASW	1000 Genomes on GRCh38,1000 Genomes 30x on GRCh38,1000 Genomes phase 3 release,1000 Genomes phase 1 release
**HG03626**	female	SAME1839723	PJL	Punjabi	SAS	South Asian Ancestry	PJL	1000 Genomes 30x on GRCh38
**NA19214**	female	SAME125386	YRI	Yoruba	AFR	African Ancestry	YRI	1000 Genomes on GRCh38,1000 Genomes 30x on GRCh38,1000 Genomes phase 3 release,1000 Genomes phase 1 release,Geuvadis
**NA19717**	male	SAME124451	MXL	Mexican Ancestry	AMR	American Ancestry	MXL	1000 Genomes on GRCh38,1000 Genomes 30x on GRCh38,1000 Genomes phase 3 release,1000 Genomes phase 1 release
**HG03633**	male	SAME1839571	PJL	Punjabi	SAS	South Asian Ancestry	PJL	1000 Genomes on GRCh38,1000 Genomes 30x on GRCh38,1000 Genomes phase 3 release
**NA18855**	female	SAME124735	YRI	Yoruba	AFR	African Ancestry	YRI	1000 Genomes 30x on GRCh38,1000 Genomes phase 3 release
**NA19724**	male	SAME124690	MXL	Mexican Ancestry	AMR	American Ancestry	MXL	1000 Genomes 30x on GRCh38,1000 Genomes phase 3 release
**NA18862**	male	SAME125118	YRI	Yoruba	AFR	African Ancestry	YRI	1000 Genomes 30x on GRCh38,1000 Genomes phase 3 release
**NA19729**	male	SAME124701	MXL	Mexican Ancestry	AMR	American Ancestry	MXL	1000 Genomes on GRCh38,1000 Genomes 30x on GRCh38,1000 Genomes phase 3 release,1000 Genomes phase 1 release
**HG03683**	female	SAME1840325	STU	Tamil	SAS	South Asian Ancestry	STU	1000 Genomes 30x on GRCh38,Human Genome Structural Variation Consortium, Phase 2
**NA18867**	female	SAME124904	YRI	Yoruba	AFR	African Ancestry	YRI	1000 Genomes on GRCh38,1000 Genomes 30x on GRCh38,1000 Genomes phase 3 release,1000 Genomes phase 1 release,Geuvadis
**NA19731**	female	SAME124870	MXL	Mexican Ancestry	AMR	American Ancestry	MXL	1000 Genomes on GRCh38,1000 Genomes 30x on GRCh38,1000 Genomes phase 3 release,1000 Genomes phase 1 release
**HG03688**	male	SAME1840265	STU	Tamil	SAS	South Asian Ancestry	STU	1000 Genomes 30x on GRCh38
**NA18874**	male	SAME125105	YRI	Yoruba	AFR	African Ancestry	YRI	1000 Genomes on GRCh38,1000 Genomes 30x on GRCh38,1000 Genomes phase 3 release,1000 Genomes phase 1 release
**NA19748**	female	SAME124729	MXL	Mexican Ancestry	AMR	American Ancestry	MXL	1000 Genomes 30x on GRCh38,1000 Genomes phase 3 release
**HG03690**	female	SAME1840365	STU	Tamil	SAS	South Asian Ancestry	STU	1000 Genomes on GRCh38,1000 Genomes 30x on GRCh38,1000 Genomes phase 3 release
**NA19221**	female	SAME125221	YRI	Yoruba	AFR	African Ancestry	YRI	1000 Genomes 30x on GRCh38,1000 Genomes phase 3 release
**NA19750**	male	SAME123698	MXL	Mexican Ancestry	AMR	American Ancestry	MXL	1000 Genomes on GRCh38,1000 Genomes 30x on GRCh38,1000 Genomes phase 3 release,1000 Genomes phase 1 release
**HG03695**	male	SAME1840128	STU	Tamil	SAS	South Asian Ancestry	STU	1000 Genomes on GRCh38,1000 Genomes 30x on GRCh38,1000 Genomes phase 3 release
**NA19226**	male	SAME125219	YRI	Yoruba	AFR	African Ancestry	YRI	1000 Genomes 30x on GRCh38,1000 Genomes phase 3 release
**NA20339**	female	SAME124201	ASW	African Ancestry SW	AFR	African Ancestry	ASW	1000 Genomes on GRCh38,1000 Genomes 30x on GRCh38,1000 Genomes phase 3 release,1000 Genomes phase 1 release
**HG03640**	female	SAME1839621	PJL	Punjabi	SAS	South Asian Ancestry	PJL	1000 Genomes on GRCh38,1000 Genomes 30x on GRCh38,1000 Genomes phase 3 release
**NA19238**	female	SAME124999	YRI	Yoruba	AFR	African Ancestry	YRI	1000 Genomes on GRCh38,1000 Genomes 30x on GRCh38,Human Genome Structural Variation Consortium, Phase 2,The Human Genome Structural Variation Consortiu
**HG03645**	female	SAME1839641	STU	Tamil	SAS	South Asian Ancestry	STU	1000 Genomes on GRCh38,1000 Genomes 30x on GRCh38,1000 Genomes phase 3 release
**NA19240**	female	SAME123688	YRI	Yoruba	AFR	African Ancestry	YRI	1000 Genomes on GRCh38,1000 Genomes 30x on GRCh38,Human Genome Structural Variation Consortium, Phase 2,The Human Genome Structural Variation Consortiu
**NA20346**	male	SAME124402	ASW	African Ancestry SW	AFR	African Ancestry	ASW	1000 Genomes on GRCh38,1000 Genomes 30x on GRCh38,1000 Genomes phase 3 release,1000 Genomes phase 1 release
**HG03652**	male	SAME1839505	PJL	Punjabi	SAS	South Asian Ancestry	PJL	1000 Genomes on GRCh38,1000 Genomes 30x on GRCh38,1000 Genomes phase 3 release
**NA20358**	male	SAME124600	ASW	African Ancestry SW	AFR	African Ancestry	ASW	1000 Genomes 30x on GRCh38,1000 Genomes phase 3 release
**NA19257**	female	SAME123495	YRI	Yoruba	AFR	African Ancestry	YRI	1000 Genomes on GRCh38,1000 Genomes 30x on GRCh38,1000 Genomes phase 3 release,1000 Genomes phase 1 release,Geuvadis
**HG03669**	female	SAME1839345	PJL	Punjabi	SAS	South Asian Ancestry	PJL	1000 Genomes 30x on GRCh38
**NA19308**	male	SAME124985	LWK	Luhya	AFR	African Ancestry	LWK	1000 Genomes on GRCh38,1000 Genomes 30x on GRCh38,1000 Genomes phase 3 release,1000 Genomes phase 1 release
**NA20505**	female	SAME124359	TSI	Toscani	EUR	European Ancestry	TSI	1000 Genomes on GRCh38,1000 Genomes 30x on GRCh38,1000 Genomes phase 3 release,1000 Genomes phase 1 release,Geuvadis
**HG03582**	male	SAME1839271	MSL	Mende	AFR	African Ancestry	MSL	1000 Genomes on GRCh38,1000 Genomes 30x on GRCh38,1000 Genomes phase 3 release
**NA19310**	female	SAME123449	LWK	Luhya	AFR	African Ancestry	LWK	1000 Genomes on GRCh38,1000 Genomes 30x on GRCh38,1000 Genomes phase 3 release,1000 Genomes phase 1 release
**NA20512**	male	SAME124560	TSI	Toscani	EUR	European Ancestry	TSI	1000 Genomes on GRCh38,1000 Genomes 30x on GRCh38,1000 Genomes phase 3 release,1000 Genomes phase 1 release,Geuvadis
**NA19315**	female	SAME123310	LWK	Luhya	AFR	African Ancestry	LWK	1000 Genomes on GRCh38,1000 Genomes 30x on GRCh38,1000 Genomes phase 3 release,1000 Genomes phase 1 release
**HG03594**	male	SAME1839181	BEB	Bengali	SAS	South Asian Ancestry	BEB	1000 Genomes on GRCh38,1000 Genomes 30x on GRCh38,1000 Genomes phase 3 release
**NA19327**	female	SAME123473	LWK	Luhya	AFR	African Ancestry	LWK	1000 Genomes on GRCh38,1000 Genomes 30x on GRCh38,1000 Genomes phase 3 release,1000 Genomes phase 1 release
**NA19394**	male	SAME124489	LWK	Luhya	AFR	African Ancestry	LWK	1000 Genomes on GRCh38,1000 Genomes 30x on GRCh38,1000 Genomes phase 3 release,1000 Genomes phase 1 release
**NA19334**	male	SAME123722	LWK	Luhya	AFR	African Ancestry	LWK	1000 Genomes on GRCh38,1000 Genomes 30x on GRCh38,1000 Genomes phase 3 release,1000 Genomes phase 1 release
**NA19399**	female	SAME124498	LWK	Luhya	AFR	African Ancestry	LWK	1000 Genomes on GRCh38,1000 Genomes 30x on GRCh38,1000 Genomes phase 3 release,1000 Genomes phase 1 release
**NA19438**	female	SAME123848	LWK	Luhya	AFR	African Ancestry	LWK	1000 Genomes on GRCh38,1000 Genomes 30x on GRCh38,1000 Genomes phase 3 release,1000 Genomes phase 1 release
**HG03703**	female	SAME1840032	PJL	Punjabi	SAS	South Asian Ancestry	PJL	1000 Genomes on GRCh38,1000 Genomes 30x on GRCh38,1000 Genomes phase 3 release
**NA20502**	female	SAME124358	TSI	Toscani	EUR	European Ancestry	TSI	1000 Genomes on GRCh38,1000 Genomes 30x on GRCh38,1000 Genomes phase 3 release,1000 Genomes phase 1 release,Geuvadis
**NA19440**	female	SAME122951	LWK	Luhya	AFR	African Ancestry	LWK	1000 Genomes on GRCh38,1000 Genomes 30x on GRCh38,1000 Genomes phase 3 release,1000 Genomes phase 1 release
**HG03708**	male	SAME1839773	PJL	Punjabi	SAS	South Asian Ancestry	PJL	1000 Genomes on GRCh38,1000 Genomes 30x on GRCh38,1000 Genomes phase 3 release
**NA20507**	female	SAME124361	TSI	Toscani	EUR	European Ancestry	TSI	1000 Genomes on GRCh38,1000 Genomes 30x on GRCh38,1000 Genomes phase 3 release,1000 Genomes phase 1 release,Geuvadis
**NA19445**	female	SAME122948	LWK	Luhya	AFR	African Ancestry	LWK	1000 Genomes on GRCh38,1000 Genomes 30x on GRCh38,1000 Genomes phase 3 release,1000 Genomes phase 1 release
**HG03710**	male	SAME1839891	PJL	Punjabi	SAS	South Asian Ancestry	PJL	1000 Genomes 30x on GRCh38
**NA20514**	female	SAME124558	TSI	Toscani	EUR	European Ancestry	TSI	1000 Genomes on GRCh38,1000 Genomes 30x on GRCh38,1000 Genomes phase 3 release,1000 Genomes phase 1 release,Geuvadis
**NA19452**	male	SAME125414	LWK	Luhya	AFR	African Ancestry	LWK	1000 Genomes on GRCh38,1000 Genomes 30x on GRCh38,1000 Genomes phase 3 release,1000 Genomes phase 1 release
**NA20519**	male	SAME123179	TSI	Toscani	EUR	European Ancestry	TSI	1000 Genomes on GRCh38,1000 Genomes 30x on GRCh38,1000 Genomes phase 3 release,1000 Genomes phase 1 release,Geuvadis
**NA19457**	female	SAME125409	LWK	Luhya	AFR	African Ancestry	LWK	1000 Genomes on GRCh38,1000 Genomes 30x on GRCh38,1000 Genomes phase 3 release,1000 Genomes phase 1 release
**NA20521**	male	SAME124018	TSI	Toscani	EUR	European Ancestry	TSI	1000 Genomes on GRCh38,1000 Genomes 30x on GRCh38,1000 Genomes phase 3 release,1000 Genomes phase 1 release,Geuvadis
**NA19755**	female	SAME123695	MXL	Mexican Ancestry	AMR	American Ancestry	MXL	1000 Genomes on GRCh38,1000 Genomes 30x on GRCh38,1000 Genomes phase 3 release,1000 Genomes phase 1 release
**HG03556**	male	SAME1839553	MSL	Mende	AFR	African Ancestry	MSL	1000 Genomes on GRCh38,1000 Genomes 30x on GRCh38,1000 Genomes phase 3 release
**NA19762**	male	SAME123897	MXL	Mexican Ancestry	AMR	American Ancestry	MXL	1000 Genomes on GRCh38,1000 Genomes 30x on GRCh38,1000 Genomes phase 3 release,1000 Genomes phase 1 release
**HG03563**	female	SAME1839421	MSL	Mende	AFR	African Ancestry	MSL	1000 Genomes on GRCh38,1000 Genomes 30x on GRCh38,1000 Genomes phase 3 release
**NA20533**	female	SAME124172	TSI	Toscani	EUR	European Ancestry	TSI	1000 Genomes on GRCh38,1000 Genomes 30x on GRCh38,1000 Genomes phase 3 release,1000 Genomes phase 1 release
**NA19774**	male	SAME124064	MXL	Mexican Ancestry	AMR	American Ancestry	MXL	1000 Genomes on GRCh38,1000 Genomes 30x on GRCh38,1000 Genomes phase 3 release,1000 Genomes phase 1 release
**HG03575**	female	SAME1839236	MSL	Mende	AFR	African Ancestry	MSL	1000 Genomes on GRCh38,1000 Genomes 30x on GRCh38,1000 Genomes phase 3 release
**NA20538**	male	SAME124163	TSI	Toscani	EUR	European Ancestry	TSI	1000 Genomes on GRCh38,1000 Genomes 30x on GRCh38,1000 Genomes phase 3 release,1000 Genomes phase 1 release,Geuvadis
**NA19779**	female	SAME124061	MXL	Mexican Ancestry	AMR	American Ancestry	MXL	1000 Genomes on GRCh38,1000 Genomes 30x on GRCh38,1000 Genomes phase 3 release,1000 Genomes phase 1 release
**NA20540**	female	SAME125148	TSI	Toscani	EUR	European Ancestry	TSI	1000 Genomes on GRCh38,1000 Genomes 30x on GRCh38,1000 Genomes phase 3 release,1000 Genomes phase 1 release,Geuvadis
**NA19781**	female	SAME123146	MXL	Mexican Ancestry	AMR	American Ancestry	MXL	1000 Genomes 30x on GRCh38,1000 Genomes phase 3 release
**HG03436**	male	SAME1840120	MSL	Mende	AFR	African Ancestry	MSL	1000 Genomes on GRCh38,1000 Genomes 30x on GRCh38,1000 Genomes phase 3 release
**NA20588**	male	SAME122866	TSI	Toscani	EUR	European Ancestry	TSI	1000 Genomes on GRCh38,1000 Genomes 30x on GRCh38,1000 Genomes phase 3 release,1000 Genomes phase 1 release,Geuvadis
**NA19786**	male	SAME123149	MXL	Mexican Ancestry	AMR	American Ancestry	MXL	1000 Genomes on GRCh38,1000 Genomes 30x on GRCh38,1000 Genomes phase 3 release,1000 Genomes phase 1 release
**NA20754**	male	SAME124173	TSI	Toscani	EUR	European Ancestry	TSI	1000 Genomes on GRCh38,1000 Genomes 30x on GRCh38,1000 Genomes phase 3 release,1000 Genomes phase 1 release,Geuvadis
**HG03455**	female	SAME1839766	MSL	Mende	AFR	African Ancestry	MSL	1000 Genomes on GRCh38,1000 Genomes 30x on GRCh38,1000 Genomes phase 3 release
**NA20759**	male	SAME125094	TSI	Toscani	EUR	European Ancestry	TSI	1000 Genomes on GRCh38,1000 Genomes 30x on GRCh38,1000 Genomes phase 3 release,1000 Genomes phase 1 release,Geuvadis
**NA19346**	male	SAME124341	LWK	Luhya	AFR	African Ancestry	LWK	1000 Genomes on GRCh38,1000 Genomes 30x on GRCh38,1000 Genomes phase 3 release,1000 Genomes phase 1 release
**NA19360**	male	SAME124915	LWK	Luhya	AFR	African Ancestry	LWK	1000 Genomes on GRCh38,1000 Genomes 30x on GRCh38,1000 Genomes phase 3 release,1000 Genomes phase 1 release
**HG03479**	female	SAME1839623	MSL	Mende	AFR	African Ancestry	MSL	1000 Genomes on GRCh38,1000 Genomes 30x on GRCh38,1000 Genomes phase 3 release
**NA19372**	male	SAME125100	LWK	Luhya	AFR	African Ancestry	LWK	1000 Genomes on GRCh38,1000 Genomes 30x on GRCh38,1000 Genomes phase 3 release,1000 Genomes phase 1 release
**NA20334**	female	SAME124196	ASW	African Ancestry SW	AFR	African Ancestry	ASW	1000 Genomes on GRCh38,1000 Genomes 30x on GRCh38,1000 Genomes phase 3 release,1000 Genomes phase 1 release
**HG03931**	female	SAME1840305	BEB	Bengali	SAS	South Asian Ancestry	BEB	1000 Genomes on GRCh38,1000 Genomes 30x on GRCh38,1000 Genomes phase 3 release
**NA19377**	female	SAME124470	LWK	Luhya	AFR	African Ancestry	LWK	1000 Genomes on GRCh38,1000 Genomes 30x on GRCh38,1000 Genomes phase 3 release,1000 Genomes phase 1 release
**NA21142**	female	SAME123344	GIH	Gujarati	SAS	South Asian Ancestry	GIH	1000 Genomes on GRCh38,1000 Genomes 30x on GRCh38,1000 Genomes phase 3 release
**HG03943**	male	SAME1840361	STU	Tamil	SAS	South Asian Ancestry	STU	1000 Genomes on GRCh38,1000 Genomes 30x on GRCh38,1000 Genomes phase 3 release
**NA19384**	male	SAME124272	LWK	Luhya	AFR	African Ancestry	LWK	1000 Genomes on GRCh38,1000 Genomes 30x on GRCh38,1000 Genomes phase 3 release,1000 Genomes phase 1 release
**NA19391**	female	SAME124494	LWK	Luhya	AFR	African Ancestry	LWK	1000 Genomes on GRCh38,1000 Genomes 30x on GRCh38,1000 Genomes phase 3 release,1000 Genomes phase 1 release
**HG03950**	male	SAME1840233	STU	Tamil	SAS	South Asian Ancestry	STU	1000 Genomes on GRCh38,1000 Genomes 30x on GRCh38,1000 Genomes phase 3 release
**HG03955**	female	SAME1840181	STU	Tamil	SAS	South Asian Ancestry	STU	1000 Genomes on GRCh38,1000 Genomes 30x on GRCh38,1000 Genomes phase 3 release
**NA19404**	female	SAME123637	LWK	Luhya	AFR	African Ancestry	LWK	1000 Genomes on GRCh38,1000 Genomes 30x on GRCh38,1000 Genomes phase 3 release,1000 Genomes phase 1 release
**HG03823**	female	SAME1839456	BEB	Bengali	SAS	South Asian Ancestry	BEB	1000 Genomes on GRCh38,1000 Genomes 30x on GRCh38,1000 Genomes phase 3 release
**NA19428**	male	SAME124036	LWK	Luhya	AFR	African Ancestry	LWK	1000 Genomes on GRCh38,1000 Genomes 30x on GRCh38,1000 Genomes phase 3 release,1000 Genomes phase 1 release
**HG03830**	male	SAME1839341	BEB	Bengali	SAS	South Asian Ancestry	BEB	1000 Genomes on GRCh38,1000 Genomes 30x on GRCh38,1000 Genomes phase 3 release
**NA19430**	male	SAME123854	LWK	Luhya	AFR	African Ancestry	LWK	1000 Genomes on GRCh38,1000 Genomes 30x on GRCh38,1000 Genomes phase 3 release,1000 Genomes phase 1 release
**NA19435**	female	SAME123849	LWK	Luhya	AFR	African Ancestry	LWK	1000 Genomes on GRCh38,1000 Genomes 30x on GRCh38,1000 Genomes phase 3 release,1000 Genomes phase 1 release
**NA19454**	male	SAME125412	LWK	Luhya	AFR	African Ancestry	LWK	1000 Genomes on GRCh38,1000 Genomes 30x on GRCh38,1000 Genomes phase 3 release
**NA19461**	male	SAME123298	LWK	Luhya	AFR	African Ancestry	LWK	1000 Genomes on GRCh38,1000 Genomes 30x on GRCh38,1000 Genomes phase 3 release,1000 Genomes phase 1 release
**HG03753**	male	SAME1839062	STU	Tamil	SAS	South Asian Ancestry	STU	1000 Genomes on GRCh38,1000 Genomes 30x on GRCh38,1000 Genomes phase 3 release
**NA19466**	male	SAME123294	LWK	Luhya	AFR	African Ancestry	LWK	1000 Genomes on GRCh38,1000 Genomes 30x on GRCh38,1000 Genomes phase 3 release,1000 Genomes phase 1 release
**HG03760**	female	SAME1839105	STU	Tamil	SAS	South Asian Ancestry	STU	1000 Genomes on GRCh38,1000 Genomes 30x on GRCh38,1000 Genomes phase 3 release
**NA19473**	female	SAME123119	LWK	Luhya	AFR	African Ancestry	LWK	1000 Genomes on GRCh38,1000 Genomes 30x on GRCh38,1000 Genomes phase 3 release,1000 Genomes phase 1 release
**HG03765**	female	SAME1839131	PJL	Punjabi	SAS	South Asian Ancestry	PJL	1000 Genomes on GRCh38,1000 Genomes 30x on GRCh38,1000 Genomes phase 3 release
**NA19625**	female	SAME123655	ASW	African Ancestry SW	AFR	African Ancestry	ASW	1000 Genomes on GRCh38,1000 Genomes 30x on GRCh38,1000 Genomes phase 3 release,1000 Genomes phase 1 release
**HG03772**	female	SAME1840398	ITU	Telugu	SAS	South Asian Ancestry	ITU	1000 Genomes on GRCh38,1000 Genomes 30x on GRCh38,1000 Genomes phase 3 release
**NA19649**	male	SAME123442	MXL	Mexican Ancestry	AMR	American Ancestry	MXL	1000 Genomes on GRCh38,1000 Genomes 30x on GRCh38,1000 Genomes phase 3 release,1000 Genomes phase 1 release
**NA20904**	male	SAME122916	GIH	Gujarati	SAS	South Asian Ancestry	GIH	1000 Genomes on GRCh38,1000 Genomes 30x on GRCh38,1000 Genomes phase 3 release
**HG03722**	female	SAME1839664	ITU	Telugu	SAS	South Asian Ancestry	ITU	1000 Genomes on GRCh38,1000 Genomes 30x on GRCh38,1000 Genomes phase 3 release
**NA19651**	female	SAME123123	MXL	Mexican Ancestry	AMR	American Ancestry	MXL	1000 Genomes on GRCh38,1000 Genomes 30x on GRCh38,1000 Genomes phase 3 release,1000 Genomes phase 1 release
**HG03727**	male	SAME1839695	ITU	Telugu	SAS	South Asian Ancestry	ITU	1000 Genomes on GRCh38,1000 Genomes 30x on GRCh38,1000 Genomes phase 3 release
**NA19656**	female	SAME123130	MXL	Mexican Ancestry	AMR	American Ancestry	MXL	1000 Genomes 30x on GRCh38,1000 Genomes phase 3 release

**NA20911**	male	SAME123101	GIH	Gujarati	SAS	South Asian Ancestry	GIH	1000 Genomes on GRCh38,1000 Genomes 30x on GRCh38,1000 Genomes phase 3 release
**HG03741**	female	SAME1839251	STU	Tamil	SAS	South Asian Ancestry	STU	1000 Genomes on GRCh38,1000 Genomes 30x on GRCh38,1000 Genomes phase 3 release
**NA19663**	female	SAME122961	MXL	Mexican Ancestry	AMR	American Ancestry	MXL	1000 Genomes on GRCh38,1000 Genomes 30x on GRCh38,1000 Genomes phase 3 release,1000 Genomes phase 1 release
**NA21089**	female	SAME123142	GIH	Gujarati	SAS	South Asian Ancestry	GIH	1000 Genomes on GRCh38,1000 Genomes 30x on GRCh38,1000 Genomes phase 3 release
**HG03746**	male	SAME1839200	STU	Tamil	SAS	South Asian Ancestry	STU	1000 Genomes on GRCh38,1000 Genomes 30x on GRCh38,1000 Genomes phase 3 release
**NA19670**	male	SAME122795	MXL	Mexican Ancestry	AMR	American Ancestry	MXL	1000 Genomes on GRCh38,1000 Genomes 30x on GRCh38,1000 Genomes phase 3 release,1000 Genomes phase 1 release
**NA21091**	male	SAME123321	GIH	Gujarati	SAS	South Asian Ancestry	GIH	1000 Genomes on GRCh38,1000 Genomes 30x on GRCh38,1000 Genomes phase 3 release
**HG03777**	male	SAME1840340	ITU	Telugu	SAS	South Asian Ancestry	ITU	1000 Genomes on GRCh38,1000 Genomes 30x on GRCh38,1000 Genomes phase 3 release
**NA19788**	female	SAME123153	MXL	Mexican Ancestry	AMR	American Ancestry	MXL	1000 Genomes on GRCh38,1000 Genomes 30x on GRCh38,1000 Genomes phase 3 release,1000 Genomes phase 1 release
**NA20884**	male	SAME124514	GIH	Gujarati	SAS	South Asian Ancestry	GIH	1000 Genomes on GRCh38,1000 Genomes 30x on GRCh38,1000 Genomes phase 3 release
**HG03784**	female	SAME1840210	ITU	Telugu	SAS	South Asian Ancestry	ITU	1000 Genomes on GRCh38,1000 Genomes 30x on GRCh38,1000 Genomes phase 3 release
**NA19790**	female	SAME123326	MXL	Mexican Ancestry	AMR	American Ancestry	MXL	1000 Genomes 30x on GRCh38,1000 Genomes phase 3 release
**NA20889**	male	SAME125305	GIH	Gujarati	SAS	South Asian Ancestry	GIH	1000 Genomes on GRCh38,1000 Genomes 30x on GRCh38,1000 Genomes phase 3 release
**HG03789**	female	SAME1840237	ITU	Telugu	SAS	South Asian Ancestry	ITU	1000 Genomes on GRCh38,1000 Genomes 30x on GRCh38,1000 Genomes phase 3 release
**NA19795**	male	SAME123329	MXL	Mexican Ancestry	AMR	American Ancestry	MXL	1000 Genomes on GRCh38,1000 Genomes 30x on GRCh38,1000 Genomes phase 3 release,1000 Genomes phase 1 release
**NA20891**	male	SAME123670	GIH	Gujarati	SAS	South Asian Ancestry	GIH	1000 Genomes on GRCh38,1000 Genomes 30x on GRCh38,1000 Genomes phase 3 release
**HG03796**	female	SAME1839928	BEB	Bengali	SAS	South Asian Ancestry	BEB	1000 Genomes on GRCh38,1000 Genomes 30x on GRCh38,1000 Genomes phase 3 release
**NA19834**	male	SAME125030	ASW	African Ancestry SW	AFR	African Ancestry	ASW	1000 Genomes on GRCh38,1000 Genomes 30x on GRCh38,1000 Genomes phase 3 release,1000 Genomes phase 1 release
**NA20896**	female	SAME123665	GIH	Gujarati	SAS	South Asian Ancestry	GIH	1000 Genomes on GRCh38,1000 Genomes 30x on GRCh38,1000 Genomes phase 3 release
**HG03804**	female	SAME1839533	BEB	Bengali	SAS	South Asian Ancestry	BEB	1000 Genomes 30x on GRCh38
**NA19904**	male	SAME125047	ASW	African Ancestry SW	AFR	African Ancestry	ASW	1000 Genomes on GRCh38,1000 Genomes 30x on GRCh38,1000 Genomes phase 3 release,1000 Genomes phase 1 release
**NA21116**	male	SAME124186	GIH	Gujarati	SAS	South Asian Ancestry	GIH	1000 Genomes on GRCh38,1000 Genomes 30x on GRCh38,1000 Genomes phase 3 release
**HG03809**	male	SAME1839554	BEB	Bengali	SAS	South Asian Ancestry	BEB	1000 Genomes on GRCh38,1000 Genomes 30x on GRCh38,1000 Genomes phase 3 release
**NA19909**	female	SAME125052	ASW	African Ancestry SW	AFR	African Ancestry	ASW	1000 Genomes on GRCh38,1000 Genomes 30x on GRCh38,1000 Genomes phase 3 release,1000 Genomes phase 1 release
**NA21123**	male	SAME124031	GIH	Gujarati	SAS	South Asian Ancestry	GIH	1000 Genomes on GRCh38,1000 Genomes 30x on GRCh38,1000 Genomes phase 3 release
**NA19916**	male	SAME125271	ASW	African Ancestry SW	AFR	African Ancestry	ASW	1000 Genomes on GRCh38,1000 Genomes 30x on GRCh38,1000 Genomes phase 3 release,1000 Genomes phase 1 release
**NA21128**	male	SAME124022	GIH	Gujarati	SAS	South Asian Ancestry	GIH	1000 Genomes on GRCh38,1000 Genomes 30x on GRCh38,1000 Genomes phase 3 release
**HG03816**	female	SAME1839424	BEB	Bengali	SAS	South Asian Ancestry	BEB	1000 Genomes 30x on GRCh38
**NA19923**	female	SAME124710	ASW	African Ancestry SW	AFR	African Ancestry	ASW	1000 Genomes on GRCh38,1000 Genomes 30x on GRCh38,1000 Genomes phase 3 release,1000 Genomes phase 1 release
**NA21130**	male	SAME123834	GIH	Gujarati	SAS	South Asian Ancestry	GIH	1000 Genomes on GRCh38,1000 Genomes 30x on GRCh38,1000 Genomes phase 3 release
**HG03905**	male	SAME1839503	BEB	Bengali	SAS	South Asian Ancestry	BEB	1000 Genomes on GRCh38,1000 Genomes 30x on GRCh38,1000 Genomes phase 3 release
**NA21135**	male	SAME123829	GIH	Gujarati	SAS	South Asian Ancestry	GIH	1000 Genomes on GRCh38,1000 Genomes 30x on GRCh38,1000 Genomes phase 3 release
**HG03917**	male	SAME1839323	BEB	Bengali	SAS	South Asian Ancestry	BEB	1000 Genomes on GRCh38,1000 Genomes 30x on GRCh38,1000 Genomes phase 3 release
**NA20127**	female	SAME122884	ASW	African Ancestry SW	AFR	African Ancestry	ASW	1000 Genomes on GRCh38,1000 Genomes 30x on GRCh38,1000 Genomes phase 3 release,1000 Genomes phase 1 release
**NA20858**	male	SAME123207	GIH	Gujarati	SAS	South Asian Ancestry	GIH	1000 Genomes on GRCh38,1000 Genomes 30x on GRCh38,1000 Genomes phase 3 release
**NA19675**	female	SAME122791	MXL	Mexican Ancestry	AMR	American Ancestry	MXL	1000 Genomes on GRCh38,1000 Genomes 30x on GRCh38,1000 Genomes phase 3 release,1000 Genomes phase 1 release
**NA20872**	female	SAME122842	GIH	Gujarati	SAS	South Asian Ancestry	GIH	1000 Genomes on GRCh38,1000 Genomes 30x on GRCh38,1000 Genomes phase 3 release
**HG03929**	male	SAME1839046	BEB	Bengali	SAS	South Asian Ancestry	BEB	1000 Genomes on GRCh38,1000 Genomes 30x on GRCh38
**NA19682**	male	SAME122935	MXL	Mexican Ancestry	AMR	American Ancestry	MXL	1000 Genomes on GRCh38,1000 Genomes 30x on GRCh38,1000 Genomes phase 3 release,1000 Genomes phase 1 release
**NA20877**	female	SAME123598	GIH	Gujarati	SAS	South Asian Ancestry	GIH	1000 Genomes on GRCh38,1000 Genomes 30x on GRCh38,1000 Genomes phase 3 release
**HG03885**	male	SAME1840013	STU	Tamil	SAS	South Asian Ancestry	STU	1000 Genomes on GRCh38,1000 Genomes 30x on GRCh38,1000 Genomes phase 3 release
**NA19702**	male	SAME124231	ASW	African Ancestry SW	AFR	African Ancestry	ASW	1000 Genomes 30x on GRCh38,1000 Genomes phase 3 release
**NA21104**	male	SAME124436	GIH	Gujarati	SAS	South Asian Ancestry	GIH	1000 Genomes on GRCh38,1000 Genomes 30x on GRCh38,1000 Genomes phase 3 release
**HG03897**	female	SAME1839906	STU	Tamil	SAS	South Asian Ancestry	STU	1000 Genomes on GRCh38,1000 Genomes 30x on GRCh38,1000 Genomes phase 3 release
**NA19707**	female	SAME124234	ASW	African Ancestry SW	AFR	African Ancestry	ASW	1000 Genomes on GRCh38,1000 Genomes 30x on GRCh38,1000 Genomes phase 3 release,1000 Genomes phase 1 release
**NA21109**	male	SAME124443	GIH	Gujarati	SAS	South Asian Ancestry	GIH	1000 Genomes on GRCh38,1000 Genomes 30x on GRCh38,1000 Genomes phase 3 release
**HG03900**	male	SAME1839476	STU	Tamil	SAS	South Asian Ancestry	STU	1000 Genomes on GRCh38,1000 Genomes 30x on GRCh38,1000 Genomes phase 3 release
**NA21111**	male	SAME124179	GIH	Gujarati	SAS	South Asian Ancestry	GIH	1000 Genomes on GRCh38,1000 Genomes 30x on GRCh38,1000 Genomes phase 3 release
**HG03854**	male	SAME1840283	STU	Tamil	SAS	South Asian Ancestry	STU	1000 Genomes on GRCh38,1000 Genomes 30x on GRCh38,1000 Genomes phase 3 release
**NA19719**	female	SAME124445	MXL	Mexican Ancestry	AMR	American Ancestry	MXL	1000 Genomes on GRCh38,1000 Genomes 30x on GRCh38,1000 Genomes phase 3 release,1000 Genomes phase 1 release
**HG03861**	female	SAME1840156	ITU	Telugu	SAS	South Asian Ancestry	ITU	1000 Genomes on GRCh38,1000 Genomes 30x on GRCh38,1000 Genomes phase 3 release
**NA19721**	female	SAME125311	MXL	Mexican Ancestry	AMR	American Ancestry	MXL	1000 Genomes 30x on GRCh38,1000 Genomes phase 3 release
**HG03866**	male	SAME1840087	ITU	Telugu	SAS	South Asian Ancestry	ITU	1000 Genomes on GRCh38,1000 Genomes 30x on GRCh38,1000 Genomes phase 3 release
**NA19726**	male	SAME124692	MXL	Mexican Ancestry	AMR	American Ancestry	MXL	1000 Genomes on GRCh38,1000 Genomes 30x on GRCh38,1000 Genomes phase 3 release,1000 Genomes phase 1 release
**HG03873**	female	SAME1840205	ITU	Telugu	SAS	South Asian Ancestry	ITU	1000 Genomes on GRCh38,1000 Genomes 30x on GRCh38,1000 Genomes phase 3 release
**NA19733**	female	SAME124868	MXL	Mexican Ancestry	AMR	American Ancestry	MXL	1000 Genomes 30x on GRCh38,1000 Genomes phase 3 release
**NA07345**	female	SAME123570	CEU	CEPH	EUR	European Ancestry	CEU	1000 Genomes 30x on GRCh38,1000 Genomes phase 3 release
**NA19740**	female	SAME124719	MXL	Mexican Ancestry	AMR	American Ancestry	MXL	1000 Genomes on GRCh38,1000 Genomes 30x on GRCh38,1000 Genomes phase 3 release,1000 Genomes phase 1 release
**NA07357**	male	SAME123412	CEU	CEPH	EUR	European Ancestry	CEU	1000 Genomes on GRCh38,1000 Genomes 30x on GRCh38,1000 Genomes phase 3 release,1000 Genomes phase 1 release,Geuvadis
**NA19752**	female	SAME123700	MXL	Mexican Ancestry	AMR	American Ancestry	MXL	1000 Genomes on GRCh38,1000 Genomes 30x on GRCh38,1000 Genomes phase 3 release,1000 Genomes phase 1 release
**NA10831**	female	SAME124549	CEU	CEPH	EUR	European Ancestry	CEU	1000 Genomes 30x on GRCh38,1000 Genomes phase 3 release
**NA19757**	male	SAME123697	MXL	Mexican Ancestry	AMR	American Ancestry	MXL	1000 Genomes 30x on GRCh38,1000 Genomes phase 3 release
**NA10836**	female	SAME124545	CEU	CEPH	EUR	European Ancestry	CEU	1000 Genomes 30x on GRCh38,1000 Genomes phase 3 release
**NA19764**	female	SAME123898	MXL	Mexican Ancestry	AMR	American Ancestry	MXL	1000 Genomes on GRCh38,1000 Genomes 30x on GRCh38,1000 Genomes phase 3 release,1000 Genomes phase 1 release
**NA10843**	female	SAME124754	CEU	CEPH	EUR	European Ancestry	CEU	1000 Genomes 30x on GRCh38,1000 Genomes phase 3 release
**NA19771**	male	SAME124069	MXL	Mexican Ancestry	AMR	American Ancestry	MXL	1000 Genomes on GRCh38,1000 Genomes 30x on GRCh38,1000 Genomes phase 3 release,1000 Genomes phase 1 release
**NA19776**	female	SAME124062	MXL	Mexican Ancestry	AMR	American Ancestry	MXL	1000 Genomes on GRCh38,1000 Genomes 30x on GRCh38,1000 Genomes phase 3 release,1000 Genomes phase 1 release
**NA19783**	male	SAME123144	MXL	Mexican Ancestry	AMR	American Ancestry	MXL	1000 Genomes on GRCh38,1000 Genomes 30x on GRCh38,1000 Genomes phase 3 release,1000 Genomes phase 1 release
**NA06986**	male	SAME124764	CEU	CEPH	EUR	European Ancestry	CEU	1000 Genomes on GRCh38,1000 Genomes 30x on GRCh38,1000 Genomes phase 3 release,1000 Genomes phase 1 release,Geuvadis
**NA20278**	male	SAME123617	ASW	African Ancestry SW	AFR	African Ancestry	ASW	1000 Genomes on GRCh38,1000 Genomes 30x on GRCh38,1000 Genomes phase 3 release,1000 Genomes phase 1 release
**NA06993**	male	SAME124934	CEU	CEPH	EUR	European Ancestry	CEU	1000 Genomes 30x on GRCh38,1000 Genomes phase 3 release
**HG04042**	female	SAME1839203	STU	Tamil	SAS	South Asian Ancestry	STU	1000 Genomes on GRCh38,1000 Genomes 30x on GRCh38,1000 Genomes phase 3 release
**HG04047**	female	SAME1839221	STU	Tamil	SAS	South Asian Ancestry	STU	1000 Genomes on GRCh38,1000 Genomes 30x on GRCh38,1000 Genomes phase 3 release
**HG04054**	female	SAME1839094	ITU	Telugu	SAS	South Asian Ancestry	ITU	1000 Genomes on GRCh38,1000 Genomes 30x on GRCh38,1000 Genomes phase 3 release
**HG04059**	female	SAME1839031	ITU	Telugu	SAS	South Asian Ancestry	ITU	1000 Genomes on GRCh38,1000 Genomes 30x on GRCh38,1000 Genomes phase 3 release
**HG04061**	male	SAME1839140	ITU	Telugu	SAS	South Asian Ancestry	ITU	1000 Genomes on GRCh38,1000 Genomes 30x on GRCh38,1000 Genomes phase 3 release
**NA20317**	female	SAME123943	ASW	African Ancestry SW	AFR	African Ancestry	ASW	1000 Genomes on GRCh38,1000 Genomes 30x on GRCh38,1000 Genomes phase 3 release,1000 Genomes phase 1 release
**HG04080**	male	SAME1840211	ITU	Telugu	SAS	South Asian Ancestry	ITU	1000 Genomes on GRCh38,1000 Genomes 30x on GRCh38,1000 Genomes phase 3 release
**HG04148**	female	SAME1839115	BEB	Bengali	SAS	South Asian Ancestry	BEB	1000 Genomes 30x on GRCh38
**HG04150**	female	SAME1840289	BEB	Bengali	SAS	South Asian Ancestry	BEB	1000 Genomes on GRCh38,1000 Genomes 30x on GRCh38
**NA20348**	male	SAME124407	ASW	African Ancestry SW	AFR	African Ancestry	ASW	1000 Genomes on GRCh38,1000 Genomes 30x on GRCh38,1000 Genomes phase 3 release,1000 Genomes phase 1 release
**HG04155**	male	SAME1840381	BEB	Bengali	SAS	South Asian Ancestry	BEB	1000 Genomes on GRCh38,1000 Genomes 30x on GRCh38,1000 Genomes phase 3 release
**HG04162**	female	SAME1840098	BEB	Bengali	SAS	South Asian Ancestry	BEB	1000 Genomes on GRCh38,1000 Genomes 30x on GRCh38,1000 Genomes phase 3 release
**NA20355**	female	SAME124597	ASW	African Ancestry SW	AFR	African Ancestry	ASW	1000 Genomes on GRCh38,1000 Genomes 30x on GRCh38,1000 Genomes phase 3 release
**HG04174**	female	SAME1839982	BEB	Bengali	SAS	South Asian Ancestry	BEB	1000 Genomes on GRCh38,1000 Genomes 30x on GRCh38
**NA20362**	male	SAME124788	ASW	African Ancestry SW	AFR	African Ancestry	ASW	1000 Genomes on GRCh38,1000 Genomes 30x on GRCh38,1000 Genomes phase 3 release
**NA20761**	female	SAME124363	TSI	Toscani	EUR	European Ancestry	TSI	1000 Genomes on GRCh38,1000 Genomes 30x on GRCh38,1000 Genomes phase 3 release,1000 Genomes phase 1 release,Geuvadis
**HG04186**	female	SAME1839753	BEB	Bengali	SAS	South Asian Ancestry	BEB	1000 Genomes on GRCh38,1000 Genomes 30x on GRCh38,1000 Genomes phase 3 release
**NA20766**	female	SAME124370	TSI	Toscani	EUR	European Ancestry	TSI	1000 Genomes on GRCh38,1000 Genomes 30x on GRCh38,1000 Genomes phase 3 release,1000 Genomes phase 1 release,Geuvadis
**HG04193**	male	SAME1839811	BEB	Bengali	SAS	South Asian Ancestry	BEB	1000 Genomes 30x on GRCh38
**NA20773**	female	SAME124575	TSI	Toscani	EUR	European Ancestry	TSI	1000 Genomes on GRCh38,1000 Genomes 30x on GRCh38,1000 Genomes phase 3 release,1000 Genomes phase 1 release,Geuvadis
**HG04100**	male	SAME1839536	STU	Tamil	SAS	South Asian Ancestry	STU	1000 Genomes on GRCh38,1000 Genomes 30x on GRCh38,1000 Genomes phase 3 release
**NA20778**	male	SAME124571	TSI	Toscani	EUR	European Ancestry	TSI	1000 Genomes on GRCh38,1000 Genomes 30x on GRCh38,1000 Genomes phase 3 release,1000 Genomes phase 1 release,Geuvadis
**HG04131**	male	SAME1839279	BEB	Bengali	SAS	South Asian Ancestry	BEB	1000 Genomes on GRCh38,1000 Genomes 30x on GRCh38,1000 Genomes phase 3 release
**NA20785**	male	SAME123611	TSI	Toscani	EUR	European Ancestry	TSI	1000 Genomes on GRCh38,1000 Genomes 30x on GRCh38,1000 Genomes phase 3 release,1000 Genomes phase 1 release,Geuvadis
**HG04136**	female	SAME1839310	BEB	Bengali	SAS	South Asian Ancestry	BEB	1000 Genomes 30x on GRCh38
**NA20792**	male	SAME123817	TSI	Toscani	EUR	European Ancestry	TSI	1000 Genomes on GRCh38,1000 Genomes 30x on GRCh38,1000 Genomes phase 3 release,1000 Genomes phase 1 release,Geuvadis
**HG04198**	male	SAME1839835	ITU	Telugu	SAS	South Asian Ancestry	ITU	1000 Genomes on GRCh38,1000 Genomes 30x on GRCh38,1000 Genomes phase 3 release
**NA20797**	female	SAME123820	TSI	Toscani	EUR	European Ancestry	TSI	1000 Genomes on GRCh38,1000 Genomes 30x on GRCh38,1000 Genomes phase 3 release,1000 Genomes phase 1 release,Geuvadis
**HG04206**	male	SAME1839447	ITU	Telugu	SAS	South Asian Ancestry	ITU	1000 Genomes on GRCh38,1000 Genomes 30x on GRCh38,1000 Genomes phase 3 release
**NA20800**	female	SAME125364	TSI	Toscani	EUR	European Ancestry	TSI	1000 Genomes on GRCh38,1000 Genomes 30x on GRCh38,1000 Genomes phase 3 release,1000 Genomes phase 1 release,Geuvadis
**HG04225**	male	SAME1839048	ITU	Telugu	SAS	South Asian Ancestry	ITU	1000 Genomes on GRCh38,1000 Genomes 30x on GRCh38,1000 Genomes phase 3 release
**NA20805**	male	SAME125361	TSI	Toscani	EUR	European Ancestry	TSI	1000 Genomes on GRCh38,1000 Genomes 30x on GRCh38,1000 Genomes phase 3 release,1000 Genomes phase 1 release,Geuvadis
**HG03967**	male	SAME1840072	ITU	Telugu	SAS	South Asian Ancestry	ITU	1000 Genomes on GRCh38,1000 Genomes 30x on GRCh38,1000 Genomes phase 3 release
**NA20812**	male	SAME125190	TSI	Toscani	EUR	European Ancestry	TSI	1000 Genomes on GRCh38,1000 Genomes 30x on GRCh38,1000 Genomes phase 3 release,1000 Genomes phase 1 release,Geuvadis
**HG03974**	male	SAME1839735	ITU	Telugu	SAS	South Asian Ancestry	ITU	1000 Genomes on GRCh38,1000 Genomes 30x on GRCh38,1000 Genomes phase 3 release
**HG03986**	female	SAME1839605	STU	Tamil	SAS	South Asian Ancestry	STU	1000 Genomes on GRCh38,1000 Genomes 30x on GRCh38,1000 Genomes phase 3 release
**HG03998**	male	SAME1839408	STU	Tamil	SAS	South Asian Ancestry	STU	1000 Genomes on GRCh38,1000 Genomes 30x on GRCh38,1000 Genomes phase 3 release
**NA21137**	female	SAME124038	GIH	Gujarati	SAS	South Asian Ancestry	GIH	1000 Genomes on GRCh38,1000 Genomes 30x on GRCh38,1000 Genomes phase 3 release
**HG04023**	male	SAME1839696	ITU	Telugu	SAS	South Asian Ancestry	ITU	1000 Genomes on GRCh38,1000 Genomes 30x on GRCh38,1000 Genomes phase 3 release
**NA21144**	female	SAME124511	GIH	Gujarati	SAS	South Asian Ancestry	GIH	1000 Genomes on GRCh38,1000 Genomes 30x on GRCh38,1000 Genomes phase 3 release

**HG04035**	female	SAME1839487	STU	Tamil	SAS	South Asian Ancestry	STU	1000 Genomes on GRCh38,1000 Genomes 30x on GRCh38,1000 Genomes phase 3 release
**NA18486**	male	SAME124099	YRI	Yoruba	AFR	African Ancestry	YRI	1000 Genomes on GRCh38,1000 Genomes 30x on GRCh38,1000 Genomes phase 3 release,1000 Genomes phase 1 release,Geuvadis
**NA18498**	male	SAME124058	YRI	Yoruba	AFR	African Ancestry	YRI	1000 Genomes on GRCh38,1000 Genomes 30x on GRCh38,1000 Genomes phase 3 release,1000 Genomes phase 1 release,Geuvadis
**NA18501**	male	SAME124991	YRI	Yoruba	AFR	African Ancestry	YRI	1000 Genomes on GRCh38,1000 Genomes 30x on GRCh38,1000 Genomes phase 3 release,1000 Genomes phase 1 release
**NA20874**	female	SAME123597	GIH	Gujarati	SAS	South Asian Ancestry	GIH	1000 Genomes on GRCh38,1000 Genomes 30x on GRCh38,1000 Genomes phase 3 release
**NA18506**	male	SAME125059	YRI	Yoruba	AFR	African Ancestry	YRI	1000 Genomes 30x on GRCh38,1000 Genomes phase 3 release
**NA18518**	female	SAME124889	YRI	Yoruba	AFR	African Ancestry	YRI	1000 Genomes 30x on GRCh38,1000 Genomes phase 3 release
**NA20881**	female	SAME124531	GIH	Gujarati	SAS	South Asian Ancestry	GIH	1000 Genomes on GRCh38,1000 Genomes 30x on GRCh38,1000 Genomes phase 3 release
**NA18520**	female	SAME123565	YRI	Yoruba	AFR	African Ancestry	YRI	1000 Genomes on GRCh38,1000 Genomes 30x on GRCh38,1000 Genomes phase 3 release,1000 Genomes phase 1 release,Geuvadis
**NA20886**	female	SAME123878	GIH	Gujarati	SAS	South Asian Ancestry	GIH	1000 Genomes on GRCh38,1000 Genomes 30x on GRCh38,1000 Genomes phase 3 release
**NA18525**	female	SAME124672	CHB	Han Chinese	EAS	East Asian Ancestry	CHB	1000 Genomes on GRCh38,1000 Genomes 30x on GRCh38,1000 Genomes phase 3 release,1000 Genomes phase 1 release
**NA18532**	female	SAME124482	CHB	Han Chinese	EAS	East Asian Ancestry	CHB	1000 Genomes on GRCh38,1000 Genomes 30x on GRCh38,1000 Genomes phase 3 release,1000 Genomes phase 1 release,90 Han Chinese high coverage genomes
**NA18537**	female	SAME124485	CHB	Han Chinese	EAS	East Asian Ancestry	CHB	1000 Genomes on GRCh38,1000 Genomes 30x on GRCh38,1000 Genomes phase 3 release,1000 Genomes phase 1 release,90 Han Chinese high coverage genomes
**NA20901**	male	SAME122919	GIH	Gujarati	SAS	South Asian Ancestry	GIH	1000 Genomes on GRCh38,1000 Genomes 30x on GRCh38,1000 Genomes phase 3 release
**NA18544**	male	SAME124265	CHB	Han Chinese	EAS	East Asian Ancestry	CHB	1000 Genomes on GRCh38,1000 Genomes 30x on GRCh38,1000 Genomes phase 3 release,1000 Genomes phase 1 release
**NA20906**	female	SAME122918	GIH	Gujarati	SAS	South Asian Ancestry	GIH	1000 Genomes on GRCh38,1000 Genomes 30x on GRCh38,1000 Genomes phase 3 release
**NA18549**	male	SAME124262	CHB	Han Chinese	EAS	East Asian Ancestry	CHB	1000 Genomes on GRCh38,1000 Genomes 30x on GRCh38,1000 Genomes phase 3 release,1000 Genomes phase 1 release
**NA21086**	female	SAME123139	GIH	Gujarati	SAS	South Asian Ancestry	GIH	1000 Genomes on GRCh38,1000 Genomes 30x on GRCh38,1000 Genomes phase 3 release
**NA18563**	male	SAME123928	CHB	Han Chinese	EAS	East Asian Ancestry	CHB	1000 Genomes on GRCh38,1000 Genomes 30x on GRCh38,1000 Genomes phase 3 release,1000 Genomes phase 1 release,90 Han Chinese high coverage genomes
**NA18570**	female	SAME123741	CHB	Han Chinese	EAS	East Asian Ancestry	CHB	1000 Genomes on GRCh38,1000 Genomes 30x on GRCh38,1000 Genomes phase 3 release,1000 Genomes phase 1 release,90 Han Chinese high coverage genomes
**NA18582**	female	SAME123905	CHB	Han Chinese	EAS	East Asian Ancestry	CHB	1000 Genomes on GRCh38,1000 Genomes 30x on GRCh38,1000 Genomes phase 3 release,1000 Genomes phase 1 release,90 Han Chinese high coverage genomes
**NA18599**	female	SAME123713	CHB	Han Chinese	EAS	East Asian Ancestry	CHB	1000 Genomes on GRCh38,1000 Genomes 30x on GRCh38,1000 Genomes phase 3 release,1000 Genomes phase 1 release
**NA18602**	female	SAME124876	CHB	Han Chinese	EAS	East Asian Ancestry	CHB	1000 Genomes on GRCh38,1000 Genomes 30x on GRCh38,1000 Genomes phase 3 release,1000 Genomes phase 1 release
**NA18614**	female	SAME125034	CHB	Han Chinese	EAS	East Asian Ancestry	CHB	1000 Genomes on GRCh38,1000 Genomes 30x on GRCh38,1000 Genomes phase 3 release,1000 Genomes phase 1 release
**NA21113**	male	SAME124181	GIH	Gujarati	SAS	South Asian Ancestry	GIH	1000 Genomes on GRCh38,1000 Genomes 30x on GRCh38,1000 Genomes phase 3 release
**NA18619**	female	SAME125043	CHB	Han Chinese	EAS	East Asian Ancestry	CHB	1000 Genomes on GRCh38,1000 Genomes 30x on GRCh38,1000 Genomes phase 3 release,1000 Genomes phase 1 release
**NA21118**	male	SAME123585	GIH	Gujarati	SAS	South Asian Ancestry	GIH	1000 Genomes on GRCh38,1000 Genomes 30x on GRCh38,1000 Genomes phase 3 release
**NA18621**	male	SAME124509	CHB	Han Chinese	EAS	East Asian Ancestry	CHB	1000 Genomes on GRCh38,1000 Genomes 30x on GRCh38,1000 Genomes phase 3 release,1000 Genomes phase 1 release,90 Han Chinese high coverage genomes
**NA21120**	female	SAME124028	GIH	Gujarati	SAS	South Asian Ancestry	GIH	1000 Genomes on GRCh38,1000 Genomes 30x on GRCh38,1000 Genomes phase 3 release
**NA18626**	female	SAME125250	CHB	Han Chinese	EAS	East Asian Ancestry	CHB	1000 Genomes on GRCh38,1000 Genomes 30x on GRCh38,1000 Genomes phase 3 release,1000 Genomes phase 1 release
**NA21125**	female	SAME124025	GIH	Gujarati	SAS	South Asian Ancestry	GIH	1000 Genomes on GRCh38,1000 Genomes 30x on GRCh38,1000 Genomes phase 3 release
**NA18633**	male	SAME124704	CHB	Han Chinese	EAS	East Asian Ancestry	CHB	1000 Genomes on GRCh38,1000 Genomes 30x on GRCh38,1000 Genomes phase 3 release,1000 Genomes phase 1 release,90 Han Chinese high coverage genomes
**NA21093**	male	SAME123323	GIH	Gujarati	SAS	South Asian Ancestry	GIH	1000 Genomes on GRCh38,1000 Genomes 30x on GRCh38,1000 Genomes phase 3 release
**NA18638**	male	SAME124693	CHB	Han Chinese	EAS	East Asian Ancestry	CHB	1000 Genomes on GRCh38,1000 Genomes 30x on GRCh38,1000 Genomes phase 3 release,1000 Genomes phase 1 release
**NA21098**	male	SAME123324	GIH	Gujarati	SAS	South Asian Ancestry	GIH	1000 Genomes on GRCh38,1000 Genomes 30x on GRCh38,1000 Genomes phase 3 release
**NA18640**	female	SAME122980	CHB	Han Chinese	EAS	East Asian Ancestry	CHB	1000 Genomes on GRCh38,1000 Genomes 30x on GRCh38,1000 Genomes phase 3 release,1000 Genomes phase 1 release
**NA21101**	female	SAME124441	GIH	Gujarati	SAS	South Asian Ancestry	GIH	1000 Genomes on GRCh38,1000 Genomes 30x on GRCh38,1000 Genomes phase 3 release
**NA18645**	male	SAME122975	CHB	Han Chinese	EAS	East Asian Ancestry	CHB	1000 Genomes on GRCh38,1000 Genomes 30x on GRCh38,1000 Genomes phase 3 release,1000 Genomes phase 1 release
**NA21106**	female	SAME124434	GIH	Gujarati	SAS	South Asian Ancestry	GIH	1000 Genomes on GRCh38,1000 Genomes 30x on GRCh38,1000 Genomes phase 3 release
**NA20850**	male	SAME123246	GIH	Gujarati	SAS	South Asian Ancestry	GIH	1000 Genomes on GRCh38,1000 Genomes 30x on GRCh38,1000 Genomes phase 3 release
**NA18979**	female	SAME123759	JPT	Japanese	EAS	East Asian Ancestry	JPT	1000 Genomes on GRCh38,1000 Genomes 30x on GRCh38,1000 Genomes phase 3 release,1000 Genomes phase 1 release
**NA20862**	female	SAME123804	GIH	Gujarati	SAS	South Asian Ancestry	GIH	1000 Genomes on GRCh38,1000 Genomes 30x on GRCh38,1000 Genomes phase 3 release
**NA18981**	female	SAME123426	JPT	Japanese	EAS	East Asian Ancestry	JPT	1000 Genomes on GRCh38,1000 Genomes 30x on GRCh38,1000 Genomes phase 3 release,1000 Genomes phase 1 release
**NA20867**	male	SAME123809	GIH	Gujarati	SAS	South Asian Ancestry	GIH	1000 Genomes on GRCh38,1000 Genomes 30x on GRCh38,1000 Genomes phase 3 release
**NA18986**	male	SAME125069	JPT	Japanese	EAS	East Asian Ancestry	JPT	1000 Genomes on GRCh38,1000 Genomes 30x on GRCh38,1000 Genomes phase 3 release,1000 Genomes phase 1 release
**NA18993**	female	SAME124894	JPT	Japanese	EAS	East Asian Ancestry	JPT	1000 Genomes on GRCh38,1000 Genomes 30x on GRCh38,1000 Genomes phase 3 release,1000 Genomes phase 1 release
**NA18998**	female	SAME124891	JPT	Japanese	EAS	East Asian Ancestry	JPT	1000 Genomes on GRCh38,1000 Genomes 30x on GRCh38,1000 Genomes phase 3 release,1000 Genomes phase 1 release
**NA19004**	male	SAME124473	JPT	Japanese	EAS	East Asian Ancestry	JPT	1000 Genomes on GRCh38,1000 Genomes 30x on GRCh38,1000 Genomes phase 3 release,1000 Genomes phase 1 release
**NA19009**	male	SAME125133	JPT	Japanese	EAS	East Asian Ancestry	JPT	1000 Genomes on GRCh38,1000 Genomes 30x on GRCh38,1000 Genomes phase 3 release,1000 Genomes phase 1 release
**NA19011**	female	SAME124512	JPT	Japanese	EAS	East Asian Ancestry	JPT	1000 Genomes on GRCh38,1000 Genomes 30x on GRCh38,1000 Genomes phase 3 release
**NA19023**	female	SAME124854	LWK	Luhya,Luhya	AFR	African Ancestry,Africa (SGDP)	LWK,LuhyaSGDP	1000 Genomes on GRCh38,Simons Genome Diversity Project,1000 Genomes 30x on GRCh38,1000 Genomes phase 3 release
**NA19028**	male	SAME124848	LWK	Luhya	AFR	African Ancestry	LWK	1000 Genomes on GRCh38,1000 Genomes 30x on GRCh38,1000 Genomes phase 3 release,1000 Genomes phase 1 release
**NA19030**	female	SAME123544	LWK	Luhya	AFR	African Ancestry	LWK	1000 Genomes on GRCh38,1000 Genomes 30x on GRCh38,1000 Genomes phase 3 release
**NA19035**	male	SAME124664	LWK	Luhya	AFR	African Ancestry	LWK	1000 Genomes on GRCh38,1000 Genomes 30x on GRCh38,1000 Genomes phase 3 release,1000 Genomes phase 1 release
**NA19042**	female	SAME125242	LWK	Luhya	AFR	African Ancestry	LWK	1000 Genomes on GRCh38,1000 Genomes 30x on GRCh38,1000 Genomes phase 3 release
**NA19054**	female	SAME125028	JPT	Japanese	EAS	East Asian Ancestry	JPT	1000 Genomes on GRCh38,1000 Genomes 30x on GRCh38,1000 Genomes phase 3 release,1000 Genomes phase 1 release
**NA19059**	female	SAME125023	JPT	Japanese	EAS	East Asian Ancestry	JPT	1000 Genomes on GRCh38,1000 Genomes 30x on GRCh38,1000 Genomes phase 3 release,1000 Genomes phase 1 release
**NA19066**	male	SAME123008	JPT	Japanese	EAS	East Asian Ancestry	JPT	1000 Genomes on GRCh38,1000 Genomes 30x on GRCh38,1000 Genomes phase 3 release,1000 Genomes phase 1 release
**NA19078**	female	SAME122824	JPT	Japanese	EAS	East Asian Ancestry	JPT	1000 Genomes on GRCh38,1000 Genomes 30x on GRCh38,1000 Genomes phase 3 release,1000 Genomes phase 1 release
**NA19080**	female	SAME125313	JPT	Japanese	EAS	East Asian Ancestry	JPT	1000 Genomes on GRCh38,1000 Genomes 30x on GRCh38,1000 Genomes phase 3 release,1000 Genomes phase 1 release
**NA19085**	male	SAME123365	JPT	Japanese	EAS	East Asian Ancestry	JPT	1000 Genomes on GRCh38,1000 Genomes 30x on GRCh38,1000 Genomes phase 3 release,1000 Genomes phase 1 release
**NA19092**	male	SAME123173	YRI	Yoruba	AFR	African Ancestry	YRI	1000 Genomes on GRCh38,1000 Genomes 30x on GRCh38,1000 Genomes phase 3 release,1000 Genomes phase 1 release,Geuvadis
**NA19097**	female	SAME123168	YRI	Yoruba	AFR	African Ancestry	YRI	1000 Genomes 30x on GRCh38,1000 Genomes phase 3 release
**NA19100**	female	SAME125401	YRI	Yoruba	AFR	African Ancestry	YRI	1000 Genomes 30x on GRCh38,1000 Genomes phase 3 release
**NA12154**	male	SAME124959	CEU	CEPH	EUR	European Ancestry	CEU	1000 Genomes on GRCh38,1000 Genomes 30x on GRCh38,1000 Genomes phase 3 release,1000 Genomes phase 1 release,Geuvadis
**NA12248**	male	SAME125372	CEU	CEPH	EUR	European Ancestry	CEU	1000 Genomes 30x on GRCh38,1000 Genomes phase 3 release
**NA12274**	male	SAME125249	CEU	CEPH	EUR	European Ancestry	CEU	1000 Genomes 30x on GRCh38,1000 Genomes phase 3 release
**NA12286**	male	SAME124642	CEU	CEPH	EUR	European Ancestry	CEU	1000 Genomes on GRCh38,1000 Genomes 30x on GRCh38,1000 Genomes phase 3 release,1000 Genomes phase 1 release,Geuvadis
**NA12344**	male	SAME123946	CEU	CEPH	EUR	European Ancestry	CEU	1000 Genomes 30x on GRCh38,1000 Genomes phase 3 release
**NA12399**	male	SAME124193	CEU	CEPH	EUR	European Ancestry	CEU	1000 Genomes on GRCh38,1000 Genomes 30x on GRCh38,1000 Genomes phase 3 release,1000 Genomes phase 1 release,Geuvadis
**NA12414**	female	SAME124057	CEU	CEPH	EUR	European Ancestry	CEU	1000 Genomes on GRCh38,1000 Genomes 30x on GRCh38,1000 Genomes phase 3 release,1000 Genomes phase 1 release
**NA12546**	male	SAME124777	CEU	CEPH	EUR	European Ancestry	CEU	1000 Genomes on GRCh38,1000 Genomes 30x on GRCh38,1000 Genomes phase 3 release,1000 Genomes phase 1 release,Geuvadis
**NA12717**	female	SAME123911	CEU	CEPH	EUR	European Ancestry	CEU	1000 Genomes on GRCh38,1000 Genomes 30x on GRCh38,1000 Genomes phase 3 release,1000 Genomes phase 1 release,Geuvadis
**NA12748**	male	SAME124827	CEU	CEPH	EUR	European Ancestry	CEU	1000 Genomes on GRCh38,1000 Genomes 30x on GRCh38,1000 Genomes phase 3 release,1000 Genomes phase 1 release
**NA12750**	male	SAME124646	CEU	CEPH	EUR	European Ancestry	CEU	1000 Genomes on GRCh38,1000 Genomes 30x on GRCh38,1000 Genomes phase 3 release,1000 Genomes phase 1 release,Geuvadis
**NA12762**	male	SAME125109	CEU	CEPH	EUR	European Ancestry	CEU	1000 Genomes on GRCh38,1000 Genomes 30x on GRCh38,1000 Genomes phase 3 release,1000 Genomes phase 1 release,Geuvadis
**NA12767**	female	SAME124472	CEU	CEPH	EUR	European Ancestry	CEU	1000 Genomes 30x on GRCh38,1000 Genomes phase 3 release
**NA12801**	male	SAME122827	CEU	CEPH	EUR	European Ancestry	CEU	1000 Genomes 30x on GRCh38,1000 Genomes phase 3 release
**NA12813**	female	SAME123024	CEU	CEPH	EUR	European Ancestry	CEU	1000 Genomes on GRCh38,1000 Genomes 30x on GRCh38,1000 Genomes phase 3 release,1000 Genomes phase 1 release,Geuvadis
**NA12818**	female	SAME123025	CEU	CEPH	EUR	European Ancestry	CEU	1000 Genomes 30x on GRCh38,1000 Genomes phase 3 release
**NA12832**	female	SAME125290	CEU	CEPH	EUR	European Ancestry	CEU	1000 Genomes 30x on GRCh38,1000 Genomes phase 3 release
**NA12875**	female	SAME123390	CEU	CEPH	EUR	European Ancestry	CEU	1000 Genomes 30x on GRCh38,1000 Genomes phase 3 release
**NA18912**	female	SAME123404	YRI	Yoruba	AFR	African Ancestry	YRI	1000 Genomes on GRCh38,1000 Genomes 30x on GRCh38,1000 Genomes phase 3 release,1000 Genomes phase 1 release,Geuvadis
**NA18917**	male	SAME123407	YRI	Yoruba	AFR	African Ancestry	YRI	1000 Genomes on GRCh38,1000 Genomes 30x on GRCh38,1000 Genomes phase 3 release,1000 Genomes phase 1 release,Geuvadis
**NA18924**	female	SAME123202	YRI	Yoruba	AFR	African Ancestry	YRI	1000 Genomes on GRCh38,1000 Genomes 30x on GRCh38,1000 Genomes phase 3 release,1000 Genomes phase 1 release
**NA18943**	male	SAME124309	JPT	Japanese	EAS	East Asian Ancestry	JPT	1000 Genomes on GRCh38,1000 Genomes 30x on GRCh38,1000 Genomes phase 3 release,1000 Genomes phase 1 release
**NA18948**	male	SAME124316	JPT	Japanese	EAS	East Asian Ancestry	JPT	1000 Genomes on GRCh38,1000 Genomes 30x on GRCh38,1000 Genomes phase 3 release,1000 Genomes phase 1 release
**NA18950**	female	SAME124112	JPT	Japanese	EAS	East Asian Ancestry	JPT	1000 Genomes on GRCh38,1000 Genomes 30x on GRCh38,1000 Genomes phase 3 release,1000 Genomes phase 1 release
**NA18962**	male	SAME123958	JPT	Japanese	EAS	East Asian Ancestry	JPT	1000 Genomes on GRCh38,1000 Genomes 30x on GRCh38,1000 Genomes phase 3 release,1000 Genomes phase 1 release
**NA18967**	male	SAME123963	JPT	Japanese	EAS	East Asian Ancestry	JPT	1000 Genomes on GRCh38,1000 Genomes 30x on GRCh38,1000 Genomes phase 3 release,1000 Genomes phase 1 release
**NA18974**	male	SAME123768	JPT	Japanese	EAS	East Asian Ancestry	JPT	1000 Genomes on GRCh38,1000 Genomes 30x on GRCh38,1000 Genomes phase 3 release,1000 Genomes phase 1 release
**NA10855**	female	SAME125331	CEU	CEPH	EUR	European Ancestry	CEU	1000 Genomes 30x on GRCh38,1000 Genomes phase 3 release
**NA11839**	male	SAME124555	CEU	CEPH	EUR	European Ancestry	CEU	1000 Genomes 30x on GRCh38,1000 Genomes phase 3 release
**NA11891**	male	SAME123046	CEU	CEPH	EUR	European Ancestry	CEU	1000 Genomes 30x on GRCh38,1000 Genomes phase 3 release
**NA11930**	male	SAME125095	CEU	CEPH	EUR	European Ancestry	CEU	1000 Genomes on GRCh38,1000 Genomes 30x on GRCh38,1000 Genomes phase 3 release,1000 Genomes phase 1 release,Geuvadis
**NA11992**	male	SAME123964	CEU	CEPH	EUR	European Ancestry	CEU	1000 Genomes on GRCh38,1000 Genomes 30x on GRCh38,1000 Genomes phase 3 release,1000 Genomes phase 1 release,Geuvadis
**NA12003**	male	SAME125142	CEU	CEPH	EUR	European Ancestry	CEU	1000 Genomes on GRCh38,1000 Genomes 30x on GRCh38,1000 Genomes phase 3 release,1000 Genomes phase 1 release
**NA12046**	female	SAME123213	CEU	CEPH	EUR	European Ancestry	CEU	1000 Genomes on GRCh38,1000 Genomes 30x on GRCh38,1000 Genomes phase 3 release,1000 Genomes phase 1 release
**NA12058**	female	SAME123416	CEU	CEPH	EUR	European Ancestry	CEU	1000 Genomes on GRCh38,1000 Genomes 30x on GRCh38,1000 Genomes phase 3 release,1000 Genomes phase 1 release,Geuvadis
**NA18854**	male	SAME124734	YRI	Yoruba	AFR	African Ancestry	YRI	1000 Genomes 30x on GRCh38,1000 Genomes phase 3 release
**NA18859**	male	SAME124731	YRI	Yoruba	AFR	African Ancestry	YRI	1000 Genomes 30x on GRCh38,1000 Genomes phase 3 release
**NA18861**	female	SAME124905	YRI	Yoruba	AFR	African Ancestry	YRI	1000 Genomes on GRCh38,1000 Genomes 30x on GRCh38,1000 Genomes phase 3 release,1000 Genomes phase 1 release,Geuvadis
**NA18873**	female	SAME123536	YRI	Yoruba	AFR	African Ancestry	YRI	1000 Genomes on GRCh38,1000 Genomes 30x on GRCh38,1000 Genomes phase 3 release,1000 Genomes phase 1 release,Geuvadis
**NA18878**	female	SAME125107	YRI	Yoruba	AFR	African Ancestry	YRI	1000 Genomes on GRCh38,1000 Genomes 30x on GRCh38,1000 Genomes phase 3 release
**NA19472**	female	SAME123120	LWK	Luhya	AFR	African Ancestry	LWK	1000 Genomes on GRCh38,1000 Genomes 30x on GRCh38,1000 Genomes phase 3 release,1000 Genomes phase 1 release
**NA19648**	female	SAME123307	MXL	Mexican Ancestry	AMR	American Ancestry	MXL	1000 Genomes on GRCh38,1000 Genomes 30x on GRCh38,1000 Genomes phase 3 release,1000 Genomes phase 1 release
**NA19650**	male	SAME124976	MXL	Mexican Ancestry	AMR	American Ancestry	MXL	1000 Genomes 30x on GRCh38,Human Genome Structural Variation Consortium, Phase 2,1000 Genomes phase 3 release
**NA19655**	male	SAME124980	MXL	Mexican Ancestry	AMR	American Ancestry	MXL	1000 Genomes on GRCh38,1000 Genomes 30x on GRCh38,1000 Genomes phase 3 release,1000 Genomes phase 1 release
**NA19662**	female	SAME122960	MXL	Mexican Ancestry	AMR	American Ancestry	MXL	1000 Genomes 30x on GRCh38,1000 Genomes phase 3 release
**NA19679**	male	SAME122796	MXL	Mexican Ancestry	AMR	American Ancestry	MXL	1000 Genomes on GRCh38,1000 Genomes 30x on GRCh38,1000 Genomes phase 3 release,1000 Genomes phase 1 release
**NA19681**	female	SAME122938	MXL	Mexican Ancestry	AMR	American Ancestry	MXL	1000 Genomes on GRCh38,1000 Genomes 30x on GRCh38,1000 Genomes phase 3 release,1000 Genomes phase 1 release

**NA19249**	male	SAME123682	YRI	Yoruba	AFR	African Ancestry	YRI	1000 Genomes 30x on GRCh38,1000 Genomes phase 3 release
**NA19256**	male	SAME124707	YRI	Yoruba	AFR	African Ancestry	YRI	1000 Genomes on GRCh38,1000 Genomes 30x on GRCh38,1000 Genomes phase 3 release,1000 Genomes phase 1 release,Geuvadis
**NA19307**	male	SAME124981	LWK	Luhya	AFR	African Ancestry	LWK	1000 Genomes on GRCh38,1000 Genomes 30x on GRCh38,1000 Genomes phase 3 release,1000 Genomes phase 1 release
**NA19314**	female	SAME123309	LWK	Luhya	AFR	African Ancestry	LWK	1000 Genomes on GRCh38,1000 Genomes 30x on GRCh38,1000 Genomes phase 3 release,1000 Genomes phase 1 release
**NA19319**	male	SAME123438	LWK	Luhya	AFR	African Ancestry	LWK	1000 Genomes on GRCh38,1000 Genomes 30x on GRCh38,1000 Genomes phase 3 release,1000 Genomes phase 1 release
**NA19321**	female	SAME124569	LWK	Luhya	AFR	African Ancestry	LWK	1000 Genomes on GRCh38,1000 Genomes 30x on GRCh38,1000 Genomes phase 3 release,1000 Genomes phase 1 release
**NA19338**	female	SAME123274	LWK	Luhya	AFR	African Ancestry	LWK	1000 Genomes on GRCh38,1000 Genomes 30x on GRCh38,1000 Genomes phase 3 release,1000 Genomes phase 1 release
**NA19376**	male	SAME124469	LWK	Luhya	AFR	African Ancestry	LWK	1000 Genomes on GRCh38,1000 Genomes 30x on GRCh38,1000 Genomes phase 3 release,1000 Genomes phase 1 release
**NA19686**	female	SAME122939	MXL	Mexican Ancestry	AMR	American Ancestry	MXL	1000 Genomes 30x on GRCh38,1000 Genomes phase 3 release
**NA19701**	female	SAME124232	ASW	African Ancestry SW	AFR	African Ancestry	ASW	1000 Genomes on GRCh38,1000 Genomes 30x on GRCh38,1000 Genomes phase 3 release,1000 Genomes phase 1 release
**NA19713**	female	SAME124448	ASW	African Ancestry SW	AFR	African Ancestry	ASW	1000 Genomes on GRCh38,1000 Genomes 30x on GRCh38,1000 Genomes phase 3 release,1000 Genomes phase 1 release
**NA19718**	female	SAME124444	MXL	Mexican Ancestry	AMR	American Ancestry	MXL	1000 Genomes 30x on GRCh38,1000 Genomes phase 3 release
**NA19720**	male	SAME124118	MXL	Mexican Ancestry	AMR	American Ancestry	MXL	1000 Genomes on GRCh38,1000 Genomes 30x on GRCh38,1000 Genomes phase 3 release,1000 Genomes phase 1 release
**NA19725**	female	SAME124689	MXL	Mexican Ancestry	AMR	American Ancestry	MXL	1000 Genomes on GRCh38,1000 Genomes 30x on GRCh38,1000 Genomes phase 3 release,1000 Genomes phase 1 release
**NA19732**	male	SAME124778	MXL	Mexican Ancestry	AMR	American Ancestry	MXL	1000 Genomes on GRCh38,1000 Genomes 30x on GRCh38,1000 Genomes phase 3 release,1000 Genomes phase 1 release
**NA19749**	female	SAME124726	MXL	Mexican Ancestry	AMR	American Ancestry	MXL	1000 Genomes on GRCh38,1000 Genomes 30x on GRCh38,1000 Genomes phase 3 release,1000 Genomes phase 1 release
**NA19174**	male	SAME124882	YRI	Yoruba	AFR	African Ancestry	YRI	1000 Genomes 30x on GRCh38,1000 Genomes phase 3 release
**NA19186**	male	SAME123861	YRI	Yoruba	AFR	African Ancestry	YRI	1000 Genomes 30x on GRCh38,1000 Genomes phase 3 release
**NA19198**	male	SAME124049	YRI	Yoruba	AFR	African Ancestry	YRI	1000 Genomes on GRCh38,1000 Genomes 30x on GRCh38,1000 Genomes phase 3 release,1000 Genomes phase 1 release,Geuvadis
**NA19201**	female	SAME122990	YRI	Yoruba	AFR	African Ancestry	YRI	1000 Genomes on GRCh38,1000 Genomes 30x on GRCh38,1000 Genomes phase 3 release,1000 Genomes phase 1 release,Geuvadis
**NA19206**	female	SAME122983	YRI	Yoruba	AFR	African Ancestry	YRI	1000 Genomes on GRCh38,1000 Genomes 30x on GRCh38,1000 Genomes phase 3 release,1000 Genomes phase 1 release,Geuvadis
**NA19213**	male	SAME125384	YRI	Yoruba	AFR	African Ancestry	YRI	1000 Genomes on GRCh38,1000 Genomes 30x on GRCh38,1000 Genomes phase 3 release,1000 Genomes phase 1 release,Geuvadis
**NA19225**	female	SAME125218	YRI	Yoruba	AFR	African Ancestry	YRI	1000 Genomes on GRCh38,1000 Genomes 30x on GRCh38,1000 Genomes phase 3 release,1000 Genomes phase 1 release,Geuvadis
**NA19237**	female	SAME125000	YRI	Yoruba	AFR	African Ancestry	YRI	1000 Genomes 30x on GRCh38,1000 Genomes phase 3 release
**NA19383**	male	SAME124273	LWK	Luhya	AFR	African Ancestry	LWK	1000 Genomes on GRCh38,1000 Genomes 30x on GRCh38,1000 Genomes phase 3 release,1000 Genomes phase 1 release
**NA19390**	female	SAME124493	LWK	Luhya	AFR	African Ancestry	LWK	1000 Genomes on GRCh38,1000 Genomes 30x on GRCh38,1000 Genomes phase 3 release,1000 Genomes phase 1 release
**NA19395**	female	SAME124490	LWK	Luhya	AFR	African Ancestry	LWK	1000 Genomes on GRCh38,1000 Genomes 30x on GRCh38,1000 Genomes phase 3 release,1000 Genomes phase 1 release
**NA19403**	female	SAME123636	LWK	Luhya	AFR	African Ancestry	LWK	1000 Genomes on GRCh38,1000 Genomes 30x on GRCh38,1000 Genomes phase 3 release,1000 Genomes phase 1 release
**NA19434**	female	SAME123850	LWK	Luhya	AFR	African Ancestry	LWK	1000 Genomes on GRCh38,1000 Genomes 30x on GRCh38,Human Genome Structural Variation Consortium, Phase 2,1000 Genomes phase 3 release,1000 Genomes ph
**NA19439**	female	SAME123847	LWK	Luhya	AFR	African Ancestry	LWK	1000 Genomes on GRCh38,1000 Genomes 30x on GRCh38,1000 Genomes phase 3 release,1000 Genomes phase 1 release
**NA19446**	female	SAME122949	LWK	Luhya	AFR	African Ancestry	LWK	1000 Genomes on GRCh38,1000 Genomes 30x on GRCh38,1000 Genomes phase 3 release,1000 Genomes phase 1 release
**NA19117**	male	SAME124914	YRI	Yoruba	AFR	African Ancestry	YRI	1000 Genomes on GRCh38,1000 Genomes 30x on GRCh38,1000 Genomes phase 3 release,1000 Genomes phase 1 release,Geuvadis
**NA19129**	female	SAME125021	YRI	Yoruba	AFR	African Ancestry	YRI	1000 Genomes on GRCh38,1000 Genomes 30x on GRCh38,1000 Genomes phase 3 release,1000 Genomes phase 1 release,Geuvadis
**NA19131**	female	SAME125230	YRI	Yoruba	AFR	African Ancestry	YRI	1000 Genomes on GRCh38,1000 Genomes 30x on GRCh38,1000 Genomes phase 3 release,1000 Genomes phase 1 release,Geuvadis
**NA19143**	female	SAME124609	YRI	Yoruba	AFR	African Ancestry	YRI	1000 Genomes on GRCh38,1000 Genomes 30x on GRCh38,1000 Genomes phase 3 release,1000 Genomes phase 1 release,Geuvadis
**NA19148**	female	SAME124606	YRI	Yoruba	AFR	African Ancestry	YRI	1000 Genomes 30x on GRCh38,1000 Genomes phase 3 release
**NA19150**	male	SAME124838	YRI	Yoruba	AFR	African Ancestry	YRI	1000 Genomes on GRCh38,1000 Genomes 30x on GRCh38,1000 Genomes phase 3 release,1000 Genomes phase 1 release,Geuvadis
**NA19751**	male	SAME123699	MXL	Mexican Ancestry	AMR	American Ancestry	MXL	1000 Genomes 30x on GRCh38,1000 Genomes phase 3 release
**NA19756**	male	SAME123696	MXL	Mexican Ancestry	AMR	American Ancestry	MXL	1000 Genomes on GRCh38,1000 Genomes 30x on GRCh38,1000 Genomes phase 3 release,1000 Genomes phase 1 release
**NA19763**	female	SAME123896	MXL	Mexican Ancestry	AMR	American Ancestry	MXL	1000 Genomes 30x on GRCh38,1000 Genomes phase 3 release
**NA19770**	female	SAME124068	MXL	Mexican Ancestry	AMR	American Ancestry	MXL	1000 Genomes on GRCh38,1000 Genomes 30x on GRCh38,1000 Genomes phase 3 release,1000 Genomes phase 1 release
**NA19775**	female	SAME124065	MXL	Mexican Ancestry	AMR	American Ancestry	MXL	1000 Genomes 30x on GRCh38,1000 Genomes phase 3 release
**NA19782**	female	SAME123145	MXL	Mexican Ancestry	AMR	American Ancestry	MXL	1000 Genomes on GRCh38,1000 Genomes 30x on GRCh38,1000 Genomes phase 3 release,1000 Genomes phase 1 release
**NA19787**	male	SAME123148	MXL	Mexican Ancestry	AMR	American Ancestry	MXL	1000 Genomes 30x on GRCh38,1000 Genomes phase 3 release
**NA19794**	female	SAME123328	MXL	Mexican Ancestry	AMR	American Ancestry	MXL	1000 Genomes on GRCh38,1000 Genomes 30x on GRCh38,1000 Genomes phase 3 release,1000 Genomes phase 1 release
**NA19819**	female	SAME124676	ASW	African Ancestry SW	AFR	African Ancestry	ASW	1000 Genomes on GRCh38,1000 Genomes 30x on GRCh38,1000 Genomes phase 3 release,1000 Genomes phase 1 release
**NA19908**	male	SAME125053	ASW	African Ancestry SW	AFR	African Ancestry	ASW	1000 Genomes on GRCh38,1000 Genomes 30x on GRCh38,1000 Genomes phase 3 release,1000 Genomes phase 1 release
**NA19922**	male	SAME124711	ASW	African Ancestry SW	AFR	African Ancestry	ASW	1000 Genomes on GRCh38,1000 Genomes 30x on GRCh38,1000 Genomes phase 3 release,1000 Genomes phase 1 release
**NA19984**	male	SAME124287	ASW	African Ancestry SW	AFR	African Ancestry	ASW	1000 Genomes on GRCh38,1000 Genomes 30x on GRCh38,1000 Genomes phase 3 release,1000 Genomes phase 1 release
**NA20126**	male	SAME122885	ASW	African Ancestry SW	AFR	African Ancestry	ASW	1000 Genomes on GRCh38,1000 Genomes 30x on GRCh38,1000 Genomes phase 3 release,1000 Genomes phase 1 release
**NA20289**	female	SAME124579	ASW	African Ancestry SW	AFR	African Ancestry	ASW	1000 Genomes on GRCh38,1000 Genomes 30x on GRCh38,1000 Genomes phase 3 release,1000 Genomes phase 1 release
**NA20291**	male	SAME124373	ASW	African Ancestry SW	AFR	African Ancestry	ASW	1000 Genomes on GRCh38,1000 Genomes 30x on GRCh38,1000 Genomes phase 3 release,1000 Genomes phase 1 release
**NA20296**	female	SAME124380	ASW	African Ancestry SW	AFR	African Ancestry	ASW	1000 Genomes on GRCh38,1000 Genomes 30x on GRCh38,1000 Genomes phase 3 release,1000 Genomes phase 1 release
**NA20513**	male	SAME124561	TSI	Toscani	EUR	European Ancestry	TSI	1000 Genomes on GRCh38,1000 Genomes 30x on GRCh38,1000 Genomes phase 3 release,1000 Genomes phase 1 release,Geuvadis
**NA20518**	male	SAME123178	TSI	Toscani	EUR	European Ancestry	TSI	1000 Genomes on GRCh38,1000 Genomes 30x on GRCh38,1000 Genomes phase 3 release,1000 Genomes phase 1 release,Geuvadis
**NA20520**	male	SAME124019	TSI	Toscani	EUR	European Ancestry	TSI	1000 Genomes on GRCh38,1000 Genomes 30x on GRCh38,1000 Genomes phase 3 release,1000 Genomes phase 1 release,Geuvadis
**NA20525**	male	SAME124015	TSI	Toscani	EUR	European Ancestry	TSI	1000 Genomes on GRCh38,1000 Genomes 30x on GRCh38,1000 Genomes phase 3 release,1000 Genomes phase 1 release,Geuvadis
**NA20532**	male	SAME124171	TSI	Toscani	EUR	European Ancestry	TSI	1000 Genomes on GRCh38,1000 Genomes 30x on GRCh38,1000 Genomes phase 3 release,1000 Genomes phase 1 release,Geuvadis
**NA20544**	male	SAME125146	TSI	Toscani	EUR	European Ancestry	TSI	1000 Genomes on GRCh38,1000 Genomes 30x on GRCh38,1000 Genomes phase 3 release,1000 Genomes phase 1 release,Geuvadis
**NA20582**	female	SAME123474	TSI	Toscani	EUR	European Ancestry	TSI	1000 Genomes on GRCh38,1000 Genomes 30x on GRCh38,1000 Genomes phase 3 release,1000 Genomes phase 1 release,Geuvadis
**NA20587**	female	SAME123481	TSI	Toscani	EUR	European Ancestry	TSI	1000 Genomes on GRCh38,1000 Genomes 30x on GRCh38,1000 Genomes phase 3 release
**NA20753**	female	SAME124178	TSI	Toscani	EUR	European Ancestry	TSI	1000 Genomes on GRCh38,1000 Genomes 30x on GRCh38,1000 Genomes phase 3 release,1000 Genomes phase 1 release
**NA20758**	male	SAME125117	TSI	Toscani	EUR	European Ancestry	TSI	1000 Genomes on GRCh38,1000 Genomes 30x on GRCh38,1000 Genomes phase 3 release,1000 Genomes phase 1 release,Geuvadis
**NA20760**	female	SAME124364	TSI	Toscani	EUR	European Ancestry	TSI	1000 Genomes on GRCh38,1000 Genomes 30x on GRCh38,1000 Genomes phase 3 release,1000 Genomes phase 1 release,Geuvadis
**NA20765**	male	SAME124367	TSI	Toscani	EUR	European Ancestry	TSI	1000 Genomes on GRCh38,1000 Genomes 30x on GRCh38,1000 Genomes phase 3 release,1000 Genomes phase 1 release,Geuvadis
**NA20772**	female	SAME124574	TSI	Toscani	EUR	European Ancestry	TSI	1000 Genomes on GRCh38,1000 Genomes 30x on GRCh38,1000 Genomes phase 3 release,1000 Genomes phase 1 release,Geuvadis
**NA21143**	female	SAME123359	GIH	Gujarati	SAS	South Asian Ancestry	GIH	1000 Genomes on GRCh38,1000 Genomes 30x on GRCh38,1000 Genomes phase 3 release
**NA20796**	male	SAME123819	TSI	Toscani	EUR	European Ancestry	TSI	1000 Genomes on GRCh38,1000 Genomes 30x on GRCh38,1000 Genomes phase 3 release,1000 Genomes phase 1 release,Geuvadis
**NA20804**	female	SAME125224	TSI	Toscani	EUR	European Ancestry	TSI	1000 Genomes on GRCh38,1000 Genomes 30x on GRCh38,1000 Genomes phase 3 release,1000 Genomes phase 1 release,Geuvadis
**NA20809**	male	SAME125359	TSI	Toscani	EUR	European Ancestry	TSI	1000 Genomes on GRCh38,1000 Genomes 30x on GRCh38,1000 Genomes phase 3 release,1000 Genomes phase 1 release,Geuvadis
**NA20811**	male	SAME123069	TSI	Toscani	EUR	European Ancestry	TSI	1000 Genomes on GRCh38,1000 Genomes 30x on GRCh38,1000 Genomes phase 3 release,1000 Genomes phase 1 release,Geuvadis
**NA20828**	female	SAME123076	TSI	Toscani	EUR	European Ancestry	TSI	1000 Genomes on GRCh38,1000 Genomes 30x on GRCh38,1000 Genomes phase 3 release,1000 Genomes phase 1 release,Geuvadis
**NA20847**	female	SAME123432	GIH	Gujarati	SAS	South Asian Ancestry	GIH	1000 Genomes on GRCh38,1000 Genomes 30x on GRCh38,Human Genome Structural Variation Consortium, Phase 2,1000 Genomes phase 3 release
**NA20854**	female	SAME123248	GIH	Gujarati	SAS	South Asian Ancestry	GIH	1000 Genomes on GRCh38,1000 Genomes 30x on GRCh38,1000 Genomes phase 3 release
**NA20342**	male	SAME124398	ASW	African Ancestry SW	AFR	African Ancestry	ASW	1000 Genomes on GRCh38,1000 Genomes 30x on GRCh38,1000 Genomes phase 3 release,1000 Genomes phase 1 release
**NA20359**	female	SAME124601	ASW	African Ancestry SW	AFR	African Ancestry	ASW	1000 Genomes on GRCh38,1000 Genomes 30x on GRCh38,1000 Genomes phase 3 release,1000 Genomes phase 1 release
**NA20412**	female	SAME124002	ASW	African Ancestry SW	AFR	African Ancestry	ASW	1000 Genomes on GRCh38,1000 Genomes 30x on GRCh38,1000 Genomes phase 3 release,1000 Genomes phase 1 release
**NA20506**	female	SAME124362	TSI	Toscani	EUR	European Ancestry	TSI	1000 Genomes on GRCh38,1000 Genomes 30x on GRCh38,1000 Genomes phase 3 release,1000 Genomes phase 1 release,Geuvadis
**NA20859**	female	SAME123249	GIH	Gujarati	SAS	South Asian Ancestry	GIH	1000 Genomes on GRCh38,1000 Genomes 30x on GRCh38,1000 Genomes phase 3 release
**NA20861**	male	SAME123807	GIH	Gujarati	SAS	South Asian Ancestry	GIH	1000 Genomes on GRCh38,1000 Genomes 30x on GRCh38,1000 Genomes phase 3 release
**NA20866**	male	SAME123808	GIH	Gujarati	SAS	South Asian Ancestry	GIH	1000 Genomes on GRCh38,1000 Genomes 30x on GRCh38,1000 Genomes phase 3 release
**NA20878**	female	SAME123595	GIH	Gujarati	SAS	South Asian Ancestry	GIH	1000 Genomes on GRCh38,1000 Genomes 30x on GRCh38,1000 Genomes phase 3 release
**NA20885**	male	SAME124525	GIH	Gujarati	SAS	South Asian Ancestry	GIH	1000 Genomes on GRCh38,1000 Genomes 30x on GRCh38,1000 Genomes phase 3 release
**NA20892**	female	SAME123109	GIH	Gujarati	SAS	South Asian Ancestry	GIH	1000 Genomes on GRCh38,1000 Genomes 30x on GRCh38,1000 Genomes phase 3 release
**NA20897**	male	SAME123664	GIH	Gujarati	SAS	South Asian Ancestry	GIH	1000 Genomes on GRCh38,1000 Genomes 30x on GRCh38,1000 Genomes phase 3 release
**NA21097**	female	SAME123319	GIH	Gujarati	SAS	South Asian Ancestry	GIH	1000 Genomes on GRCh38,1000 Genomes 30x on GRCh38,1000 Genomes phase 3 release
**NA21100**	male	SAME124440	GIH	Gujarati	SAS	South Asian Ancestry	GIH	1000 Genomes on GRCh38,1000 Genomes 30x on GRCh38,1000 Genomes phase 3 release
**NA21105**	male	SAME124437	GIH	Gujarati	SAS	South Asian Ancestry	GIH	1000 Genomes on GRCh38,1000 Genomes 30x on GRCh38,1000 Genomes phase 3 release
**NA21112**	male	SAME124182	GIH	Gujarati	SAS	South Asian Ancestry	GIH	1000 Genomes on GRCh38,1000 Genomes 30x on GRCh38,1000 Genomes phase 3 release
**NA20900**	female	SAME122920	GIH	Gujarati	SAS	South Asian Ancestry	GIH	1000 Genomes on GRCh38,1000 Genomes 30x on GRCh38,1000 Genomes phase 3 release
**NA20905**	male	SAME122915	GIH	Gujarati	SAS	South Asian Ancestry	GIH	1000 Genomes on GRCh38,1000 Genomes 30x on GRCh38,1000 Genomes phase 3 release
**NA21092**	male	SAME123322	GIH	Gujarati	SAS	South Asian Ancestry	GIH	1000 Genomes on GRCh38,1000 Genomes 30x on GRCh38,1000 Genomes phase 3 release
**NA21117**	male	SAME124185	GIH	Gujarati	SAS	South Asian Ancestry	GIH	1000 Genomes on GRCh38,1000 Genomes 30x on GRCh38,1000 Genomes phase 3 release
**NA21124**	male	SAME124024	GIH	Gujarati	SAS	South Asian Ancestry	GIH	1000 Genomes on GRCh38,1000 Genomes 30x on GRCh38,1000 Genomes phase 3 release
**NA21129**	male	SAME124023	GIH	Gujarati	SAS	South Asian Ancestry	GIH	1000 Genomes on GRCh38,1000 Genomes 30x on GRCh38,1000 Genomes phase 3 release
**NA12236**	female	SAMEA6604124	CEU	CEPH	EUR	European Ancestry	CEU	1000 Genomes 30x on GRCh38
