# Supplementary material for: High-throughput complement component 4 genomic sequence analysis with C4Investigator
Source: bioRxiv. 2023 Jul 19:2023.07.18.549551. Preprint. [Version 1] doi: 10.1101/2023.07.18.549551 (PMC10370142; doi:10.1101/2023.07.18.549551)
Supplement: Supplement 4 [file media-4.pdf]

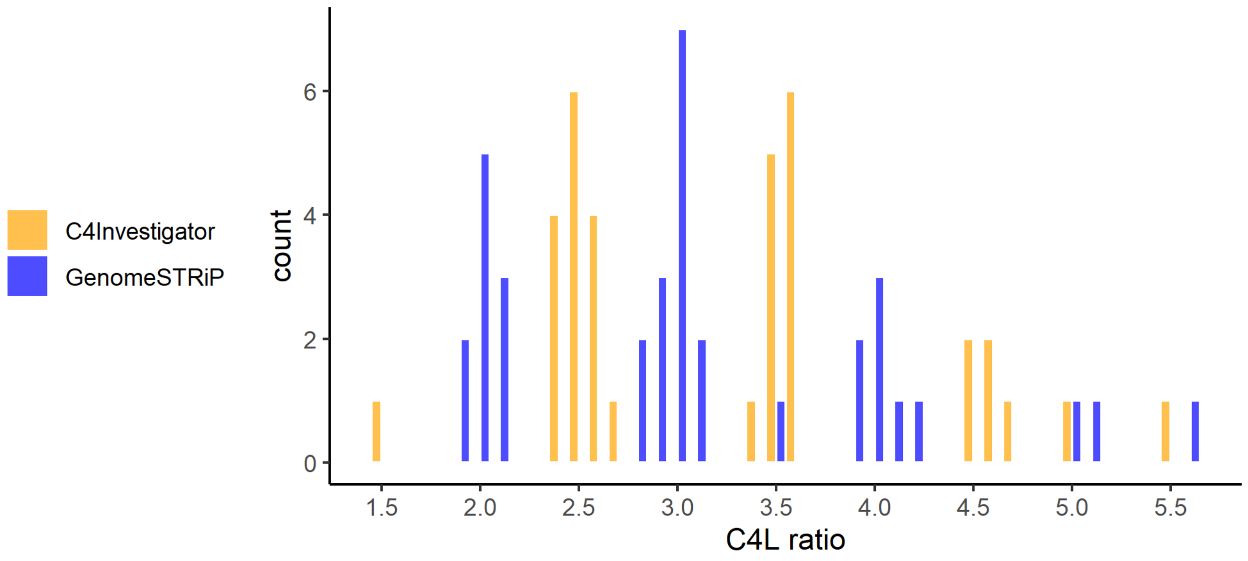

Figure S2. Histogram of normalized ratios for *C4(L)* read/k-mer counts for the C4Investigator and GenomeSTRiP workflows for discordant samples from the 1000 Genomes Project dataset.
